# Divergent Evolution and Local Establishment of Multidrug‐Resistant *Shigella sonnei* in China

**DOI:** 10.1002/mco2.70569

**Published:** 2026-01-07

**Authors:** Kangkang Liu, Jian Wang, Chaojie Yang, Yehong Yang, Xin Ge, Hongbo Liu, Xinying Du, Ying Xiang, Kaiyuan Min, Qi Wang, Hui Wang, Chao Wang, Huiqun Jia, Mingjuan Yang, Xiaoying Li, Ligui Wang, Yong Sun, Muti Mahe, Jiayong Zhao, Shijun Li, Deshan Yu, Stephen Baker, Jiangfeng Liu, Xuebin Xu, Hongbin Song, Shaofu Qiu, Juntao Yang

**Affiliations:** ^1^ Chinese PLA Center for Disease Control and Prevention Beijing China; ^2^ State Key Laboratory of Pathogen and Biosecurity Beijing Institute of Microbiology and Epidemiology Beijing China; ^3^ Beijing Key Laboratory of Surveillance Early Warning and Pathogen Research On Emerging Infectious Diseases Beijing Research Center for Respiratory Infectious Diseases Beijing Center for Disease Prevention and Control Beijing China; ^4^ State Key Laboratory of Common Mechanism Research for Major Diseases Department of Biochemistry and Molecular Biology, Institute of Basic Medical Sciences Chinese Academy of Medical Sciences & Peking Union Medical College Beijing China; ^5^ Department of Epidemiology and Biostatistics School of Public Health Anhui Medical University Hefei China; ^6^ Anhui Provincial Center for Disease Control and Prevention Hefei China; ^7^ Center For Disease Control and Prevention of Xinjiang Uygur Autonomous Region Urumqi China; ^8^ Henan Provincial Center for Disease Control and Prevention Zhengzhou China; ^9^ Guizhou Provincial Center for Disease Control and Prevention Guiyang China; ^10^ Gansu Provincial Center for Disease Control and Prevention Lanzhou China; ^11^ University of Cambridge School of Clinical Medicine Cambridge Biomedical Campus Cambridge UK; ^12^ National Institute of Pathogen Biology Chinese Academy of Medical Sciences & Peking Union Medical College Beijing China; ^13^ Shanghai Municipal Center for Disease Control and Prevention Shanghai China

**Keywords:** microevolution, multidrug resistance, ONPG‐negative variants, *Shigella sonnei*, whole‐genome sequencing

## Abstract

This study explored the microevolution of *Shigella sonnei* in China, focusing on 281 isolates exhibiting coresistance to ceftriaxone and azithromycin (cef^R^azi^R^) and 99 ONPG‐negative isolates. Phylogenetic analysis revealed that waterborne outbreak strains, characterized by multidrug resistance (MDR) and cef^R^azi^R^, clustered within the predominant domestic lineage I. In contrast, sporadic MDR strains harboring a wider array of antimicrobial resistance (AMR) genes were primarily associated with lineage II. The cef^R^azi^R^ phenotype in lineage I was mediated by an IncB/O/K/Z plasmid carrying *bla*_CTX‐M‐14_, *mphA*, *aac(3)‐IId*, *dfrA17, aadA5*, and *sul1* genes. Lineage II strains acquired cef^R^azi^R^ through a distinct IncFII plasmid possessing *bla*_CTX‐M‐15_, *ermB*, and *mphA* genes, and additionally carried a separate IncB/O/K/Z plasmid backbone with *bla*_TEM‐1_, *dfrA12*, *sul2*, *strA*, *strB*, *tet(A)*, and *aac(3)‐IId* genes. Conversion to the ONPG‐negative phenotype was linked to a deletion spanning approximately 10 kbp, which included two insertion sequences (IS1 and IS600), the *mhpBAR* operon, and the *lacIZY* operon. Genomic comparisons identified 66 SNPs and 9 accessory genes correlated with lineage II, and 23 SNPs with 9 accessory genes associated with ONPG‐negative variants. Ongoing surveillance of *S. sonnei* epidemic clones is essential to elucidate their microevolution, track transmission, and assess public health implications.

## Graphical abstract

• Evolutionary history and local establishment of Chinese *Shigella sonnei* are unclear.

• Chinese *S. sonnei* developed into four clades and ONPG‐negative variants.

• The antimicrobial resistance phenotype of Clade I was due to an IncB/O/K/Z plasmid.

• Antimicrobial resistance of Clade II was due to IncFII and IncB/O/K/Z plasmids.

• The ONPG‐negative phenotype was attributed to the deletion of a nucleotide region.

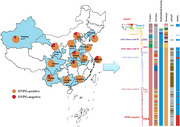

## 1 Introduction

*Shigella* species remain a significant cause of diarrheal disease globally, with *Shigella sonnei* emerging as a predominant pathogen in numerous settings [1, 2, 3]. Although typically considered less virulent than other *Shigella* species, *S. sonnei* exhibits efficient transmission via person‐to‐person contact as well as contaminated food and water sources. Its prevalence has risen notably in both industrialized and transitioning regions [4, 5]. Phylogenetic analyses suggest that *S. sonnei* originated relatively recently, with estimates placing its most recent common ancestor within the past five centuries, followed by dissemination from Europe to other parts of the world [4]. After years of evolution and spread, S. *sonnei* has developed into several distinct prevalent lineages (lineages I to V) [4, 6] and produced a variety of phenotypes adapted to the host or environment [7, 8].

The divergent evolution of S. *sonnei* is reflected by the acquisition or deletion of some virulence or metabolic genes, and more prominently, by the rapid emergence of antimicrobial resistance (AMR) [9, 10, 11, 12, 13, 14, 15, 16]. The evolution of AMR is mainly attributed to resistance to specific antibiotics, such as azithromycin, cephalosporin, and fluoroquinolone, due to the acquisition of different types of AMR determinants or the generation of specific mutation sites [17, 18]. Plasmids, integrons, and transposons usually play important roles in the horizontal transfer of AMR genes [13, 19, 20, 21, 22]. A pandemic clone of S. *sonnei* with multidrug resistance (MDR) has been found to be globally distributed in recent years [4, 23, 24, 25]. For instance, a single clone of ciprofloxacin‐resistant S. *sonnei* with classical chromosomal point mutations in the quinolone resistance‐determining regions (QRDRs) of the *gyrA* and *parC* genes was reported to cause travel‐associated infections and even large outbreaks in Australia, Bhutan, the USA, and Korea [10, 26, 27]. Moreover, macrolide‐resistant S. *sonnei* with *mphA* and/or *ermB* genes were discovered in several outbreaks, especially among men who have sex with men (MSM), which may limit the usefulness of azithromycin for the treatment of shigellosis [11, 20].

Current studies have revealed S. *sonnei* strains with different biological characteristics, including changes in virulence [7, 8, 17] and biochemical phenotype [18]. The divergent evolution of S. *sonnei* is also reflected by the acquisition or deletion of some virulence or metabolic genes. Two epidemiologically distinct clusters of S. *sonnei* with the *stx1* gene and thus producing active Stx1 toxin were identified in 56 cases in California during June 2014–April 2015 [7]. Encoding of the type VI secretion system in S. *sonnei* provided a competitive advantage over S. *flexneri*, which could potentially explain the increasing global prevalence of S. *sonnei* [17].

The above research indicates that S. *sonnei* populations have seemingly undergone local and divergent evolution in many countries and regions and have developed epidemic phenotypes with their own characteristics to adapt to different survival pressures. Despite considerable genomic diversity observed in the global S. *sonnei* population, relatively little is known about the evolutionary history and local establishment of Chinese S. *sonnei* isolates. We have reported an MDR clone of S. *sonnei* that caused six waterborne outbreaks of shigellosis with additional resistance to cephalosporin and azithromycin (cef^R^azi^R^) and the acquisition of *mphA* and *bla*_CTX‐M‐14_ genes [28, 29]. We also reported the emergence of ONPG (o‐nitrophenyl‐β‐D‐galactopyranoside)‐negative S. *sonnei* in China, with the deletion of *lac* operon genes, including *lacZ*, *lacY*, and *lacI* [18]. Here, we performed phylogenetic analysis based on a global collection of 743 S. *sonnei* genomes, including genomes from 587 Chinese isolates with different phenotypes and 156 globally distributed isolates described previously [4, 6], to investigate the microevolution of Chinese S. *sonnei* isolates within the global S. *sonnei* phylogeny. Our research provides valuable references for the evolutionary characteristics and drug resistance of S. *sonnei* strains in different regions, which is of great significance for global epidemic prevention and treatment.

## 2 Results

### 2.1 Characteristics of Chinese *S. sonnei* Isolates

**FIGURE 1:**
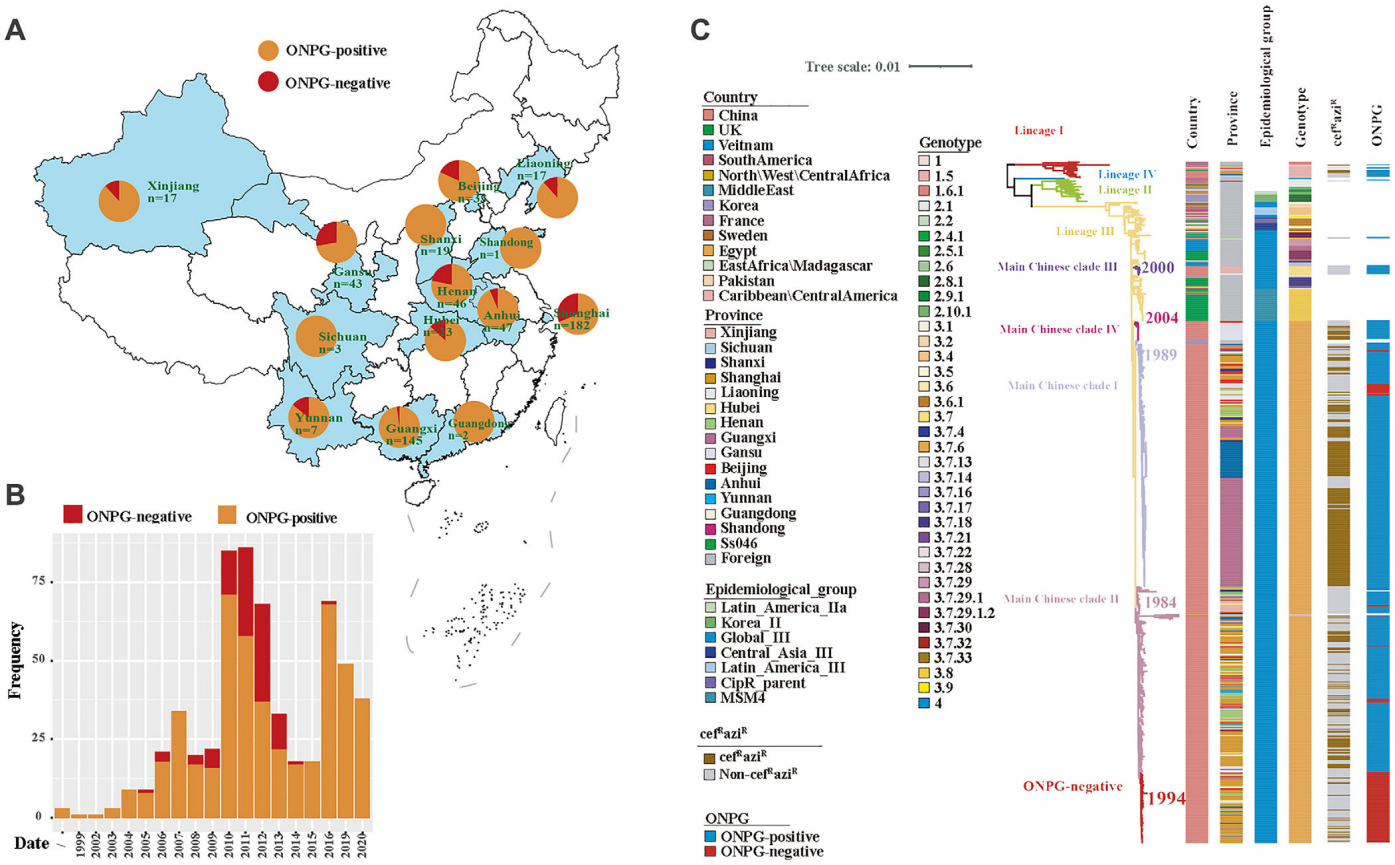
Spatiotemporal and phylogenetic characteristics of Chinese *S. sonnei* isolates. Geographic (A) and temporal (B) distributions of 587 Chinese strains, including ONPG‐negative and ONPG‐positive variants. (C) Maximum‐likelihood phylogeny of 743 global isolates, annotated with geographic origin, genotype, ONPG phenotype, and cef^R^azi^R^ status, highlighting four major Chinese clades within Lineage III.

The geographical distribution of the Chinese strains was shown in Figure 1A. Among the strains, 99 were identified as ONPG‐negative variants, which were detected in 10 of the 14 provinces of China (Figure 1A). The collection date of the Chinese strains ranged from 1999 to 2020, mainly from 2010 to 2012 (Figure 1B).

Antimicrobial susceptibility tests showed that the Chinese strains had high levels of resistance to older‐generation antimicrobials, including ticarcillin (89.95%), ampicillin (89.78%), trimethoprim (88.59%), tetracycline (81.77%), and piperacillin (79.56%). They also had high levels of resistance to the recommended first‐line drugs for shigellosis treatment, including cephalosporins such as cefazolin (75.81%), ceftriaxone (75.64%), cefoperazone (68.14%), and azithromycin (48.21%), and the rate of cef^R^azi^R^ coresistance reached 47.87% among all Chinese isolates (Table S1). In addition, 6.13% of the strains were resistant to colistin, the last‐resort antibiotic used for treating multidrug‐resistant Gram‐negative bacteria. The ONPG‐negative variants also had high levels of resistance to the older‐generation antimicrobials and developed alarming resistance to cephalosporins, including cefazolin (41.41%), ceftriaxone (40.40%), and cefoperazone (37.37%), and azithromycin (17.17%), with a cef^R^azi^R^ coresistance rate of 17.17% (Table S1). Moreover, the Chinese *S. sonnei* strains, irrespective of ONPG‐positive or negative status, showed high levels of MDR, and statistical analysis revealed that ONPG‐positive isolates had a higher rate of MDR than ONPG‐negative variants (91.2% vs. 79.8%, χ^2^ = 9.99, *p* = 0.0016). Furthermore, ONPG‐positive isolates exhibited significantly greater median numbers of resistant antibiotic classes (6 vs. 4, Mann–Whitney *U* = 13,539, *p* < 0.001) and carried more AMR genes (median: 6 vs. 5, Mann–Whitney *U* = 12,530, *p* < 0.001) (Table S2).

### 2.2 Phylogenetic Analysis of Chinese *S. sonnei* Isolates

In this section, we use Reddog and Snippy to analyze phylogeny, respectively. It can be seen that the phylogenetic trees generated by two pipelines, whether rectangular or circular, have the same lineage partitioning results (Figure S1). In addition, we provide a high‐resolution phylogenetic tree with each node having a bootstrap value (blue label) to evaluate the branch confidence score, as shown in Figure S2.

Phylogenetic analysis based on 21,307 single‐nucleotide polymorphisms (SNPs) detected in a global collection of 743 *S. sonnei* genomes showed that the global collections were divided into four lineages. The Chinese strains were distributed in Lineage III (*n* = 575), Lineage I (*n* = 11), and Lineage II (*n* = 1) (Figure 1C). Strains of Lineage III were also divided into several epidemiological clades and genotypes, and most of the Chinese strains were located in the Global III clade and belonged to genotype 3.7.6. Notably, the Chinese strains in Global III formed four distinct Chinese clades: main Chinese clade I (*n* = 265), main Chinese clade II (*n* = 280), main Chinese clade III (*n* = 10), and main Chinese clade IV (*n* = 21). Main Chinese clade I and main Chinese clade II were the two main Chinese groups, consisting of isolates from diverse regions of China, indicating local clonal expansions of *S. sonnei* within China. Main Chinese clade III and main Chinese clade IV mainly contained isolates from Xinjiang (*n* = 8) and Gansu (*n* = 17), respectively, and clustered into two localized clones. Using maximum likelihood analysis, we estimated that the main Chinese clades share a most recent common ancestor (MRCA), which evolved into recently derived and locally established clones in 1989 (main Chinese clade I), 1984 (main Chinese clade II), 2000 (main Chinese clade III), and 2004 (main Chinese clade IV) (Figure 1C). Moreover, the ONPG‐negative variants were distributed within two main Chinese clades (main Chinese clade II and main Chinese clade I), suggesting that the ONPG‐negative phenotype has evolved within the ONPG‐positive clones on multiple independent occasions. Most (*n* = 85, 85.86%) of the ONPG‐negative variants were distributed in the main Chinese clade II, making it the most prevalent ONPG‐negative cluster (*n* = 77, 77.78%) (Figure 1C). This most prevalent ONPG‐negative cluster emerged circa 1994, revealing a more recently derived clone of ONPG‐negative *S. sonnei* circulating in China (Figure 1C).

### 2.3 AMR Determinants of Chinese *S. sonnei* Isolates

**FIGURE 2:**
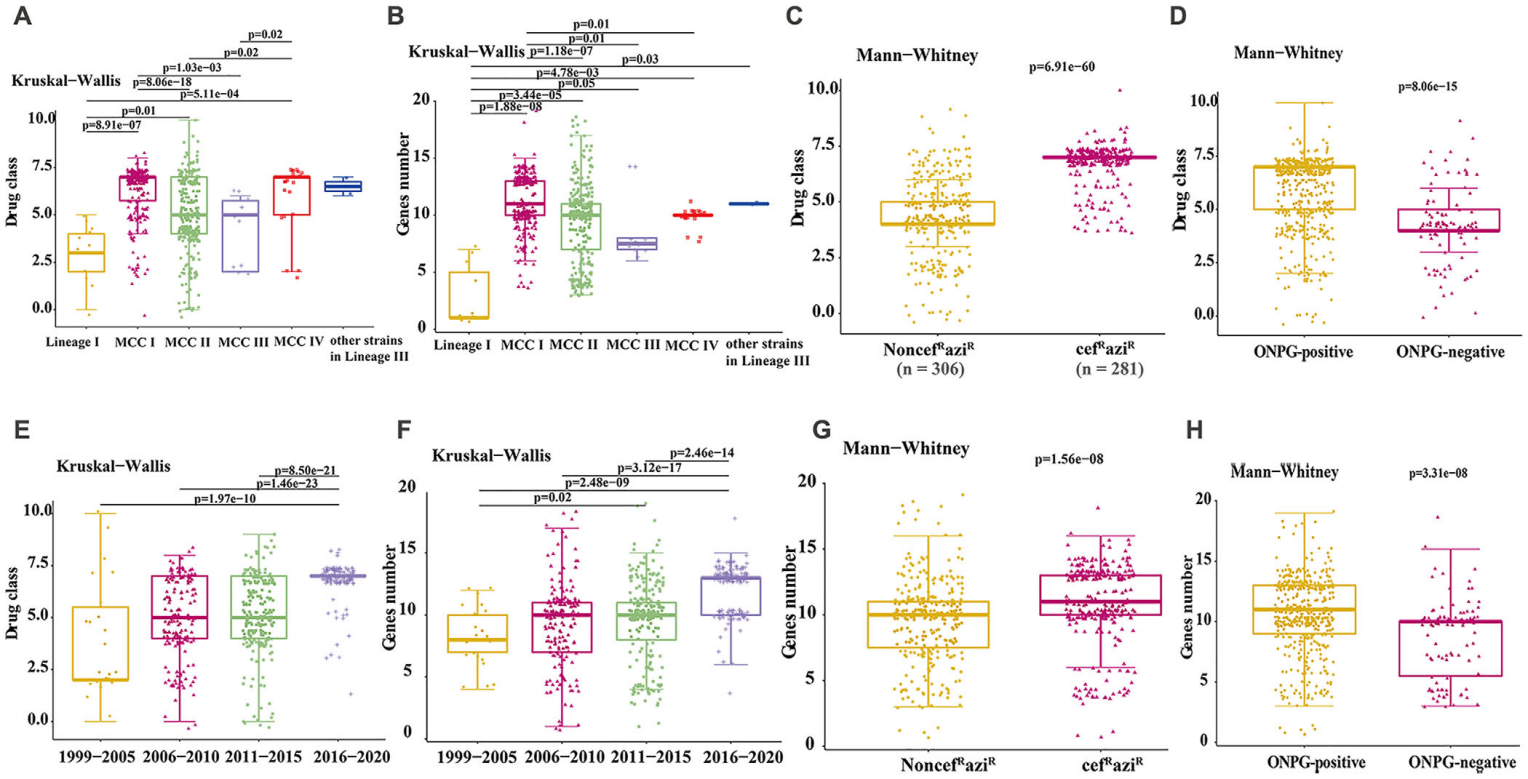
Antibiotic resistance profiles and AMR gene burden among Chinese *S. sonnei* strains. Boxplots compare the number of resistant drug classes (A–D) and AMR genes (E–H) across phylogenetic clades (A, E), collection periods (B, F), cef^R^azi^R^ status (C, G), and ONPG phenotype (D, H). Medians and quartiles are shown; points represent individual strains.

**FIGURE 3:**
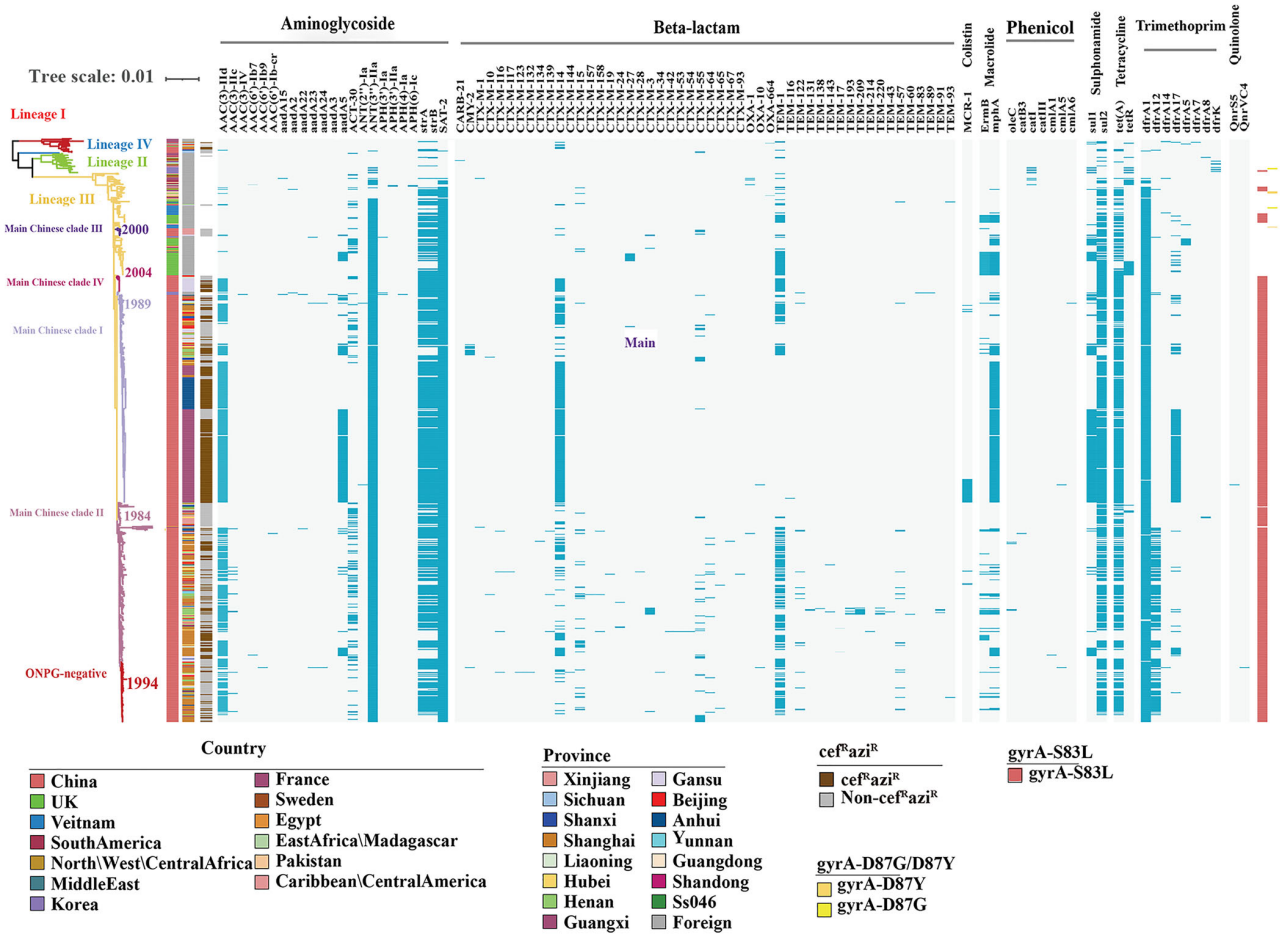
Global phylogeny and distribution of antimicrobial resistance determinants in *S. sonnei*. The tree is accompanied by a heatmap indicating geographic origin, cef^R^azi^R^ phenotype, classes of AMR genes (blue), and gyrA mutations associated with fluoroquinolone resistance.

We divided the Chinese strains into different groups according to the collection year or the branches where the strains were located, and analyzed the number of antibiotic classes and AMR genes carried by the different groups. Overall, the Chinese strains in Lineage III, including main Chinese clade I, main Chinese clade II, main Chinese clade III, and main Chinese clade VI, showed more serious antibiotic resistance and carried more AMR genes than those in other lineages (Figures 2 and 3). Significant differences were observed in antibiotic resistance patterns among the main Chinese clades (Kruskal–Wallis *H* = 59.45, *p* < 0.001), with clade I strains showing the highest resistance burden and, accordingly, containing more AMR genes (Figure 2A,B). Among the strains collected in the four time periods, temporal analysis indicated an increasing trend in antibiotic resistance, with strains collected from 2016 to 2020 showing higher numbers of resistant antibiotic classes and AMR genes compared with earlier collection periods (Figure 2E,F). This result indicated that the Chinese strains showed an increasing trend in the number of resistance drug classes and AMR genes in recent years. The cef^R^azi^R^ isolates demonstrated significantly higher numbers of resistant antibiotic classes compared with the non‐cef^R^azi^R^ isolates (median: 6 vs. 4, Mann–Whitney *U* = 24308, *p* < 0.001) (Figure 2C,G). Moreover, the ONPG‐positive isolates showed more serious antibiotic resistance and carried more AMR genes than the ONPG‐negative variants (Figure 2D,H). Analysis of individual antibiotic resistance patterns revealed significant differences between ONPG‐positive and ONPG‐negative isolates for several key antibiotics, including ceftriaxone, cefazolin, azithromycin, cefoperazone, tobramycin, gentamicin, ticarcillin, ampicillin, and colistin (all *p* < 0.05 after false discovery rate [FDR] correction).

Genomic investigation of acquired AMR genes showed that the isolates in Lineage III contained more AMR genes than those in other lineages (Figure 3). Within Lineage III, the Chinese isolates in the main Chinese clade I mainly included strains of six waterborne outbreaks of shigellosis, which are characterized by MDR, including cef^R^azi^R^ resistance. In the main Chinese clade I, coresistance to ceftriaxone and azithromycin (cef^R^azi^R^) was primarily mediated by an IncB/O/K/Z plasmid similar to pSH15sh99, which carries *bla*_CTX‐M‐14_ and mphA genes [29]. Furthermore, a subset of outbreak isolates within this clade also displayed colistin resistance, attributable to an acquired IncI2 plasmid harboring the *mcr‐1* gene [29] (Figure 3). Compared with those in main Chinese clade I, the isolates in main Chinese clade II carried more diverse AMR genes (Figure 3). The *dfrA12* gene was detected in most of the isolates (166/280, 59.29%) in the main Chinese clade II, while it was rarely detected in other clades (5/463, 1.07%). In particular, the cef^R^azi^R^ isolates in the main Chinese clade II harbored multiple extended‐spectrum β‐lactamases (ESBL) genes, such as *bla*_CTX‐M‐14_, *bla*_CTX‐M‐15_, *bla*_CTX‐M‐55_, and *bla*_TEM‐1_, and usually carried a combination of *mphA* and *ermB* genes conferring resistance to azithromycin (Figure 3).

### 2.4 Plasmids and Other Mobile Genetic Elements

**FIGURE 4:**
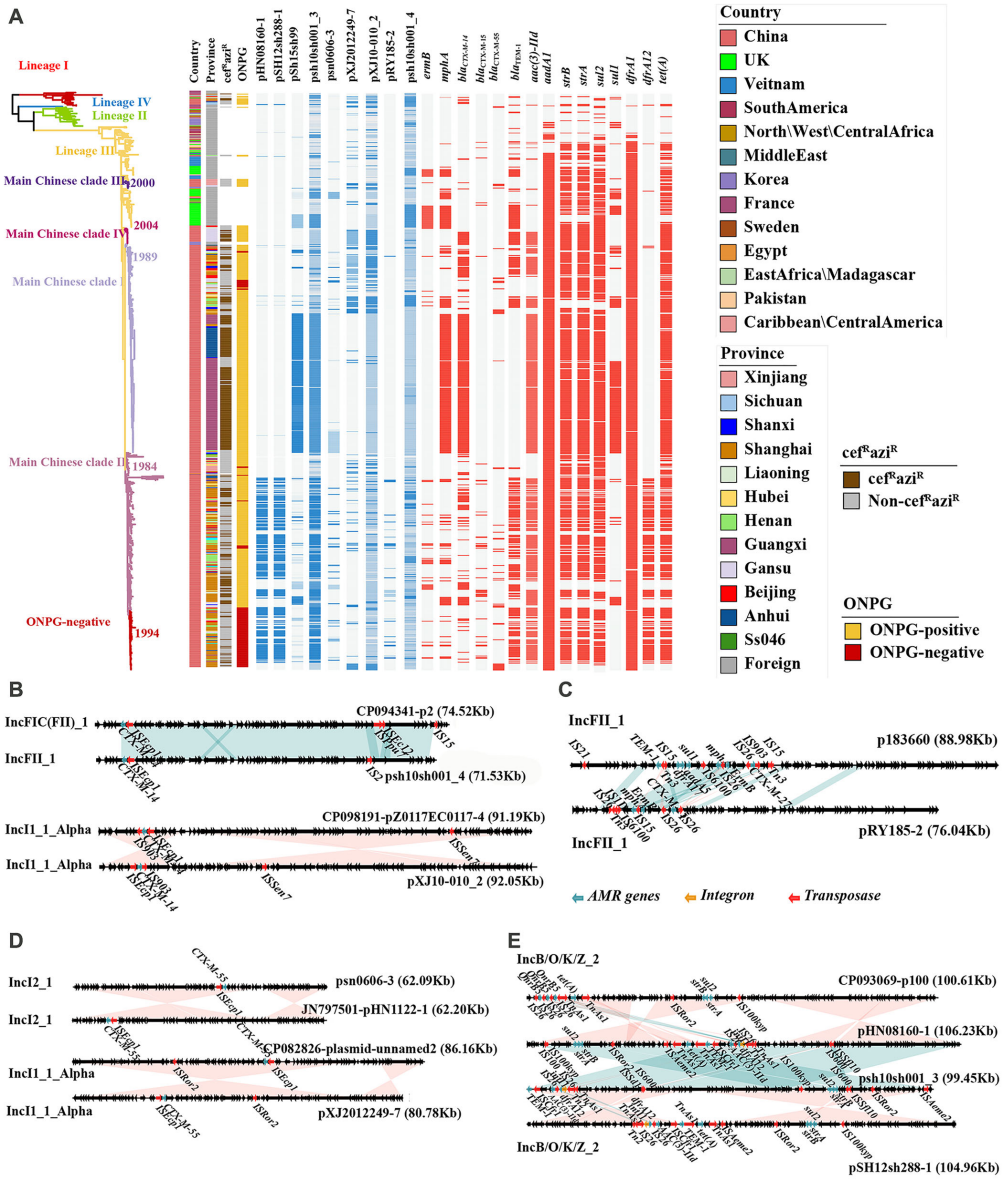
Plasmid diversity and structural comparisons of key AMR gene carriers. (A) Plasmid profiles annotated with strain metadata and AMR gene identity. (B–E) Pairwise alignments of plasmids harboring *bla*_CTX‐M_, *bla*_TEM‐1_, *mphA*, and/or *ermB* genes with reference plasmids from E. coli and S. *sonnei*.

To determine the key driver and relevant mechanism of the spread of AMR genes, we analyzed mobile genetic elements, including plasmids, integrons, and transposons. Here, we obtained eight newly completed plasmids by combining assembly of second‐ (Illumina short reads and third‐generation (Nanopore long reads) sequencing data. By integrating the plasmids sequenced in this study and the plasmids previously described [29], we found that the Chinese strains mainly harbored four dominant plasmid replicons: IncB/O/K/Z, IncFII, IncI1‐I(Alpha), and IncI2 (Figure 4). The cef^R^azi^R^ isolates in the main Chinese clade I mainly contained the pSH15sh99‐like IncB/O/K/Z plasmid (Figure 4A), which carried the *bla*_CTX‐M‐14_ and *mphA* genes conferring resistance to cephalosporins and macrolides and other AMR genes such as *aac(3)‐IId*, *dfrA17*, *aadA5*, and *sul1* associated with MDR. Within the main Chinese clade II, the *bla*_CTX‐M‐14_ gene was detected in 74 Chinese strains and was mainly located on two types of plasmids, the psh10sh001_4‐like IncFII plasmid and the pXJ10‐010_2‐like IncI1‐1(Alpha) plasmid (Figure 4A). The plasmid psh10sh001_4 is highly similar to plasmid p2 (accession CP094341) of the *Escherichia coli* W224N strain isolated in China, and plasmid pXJ10‐010_2 is highly similar to plasmid pZ0117EC0117‐4 (accession CP098191) of the *E. coli* Z0117EC0117 strain isolated in South Korea (Figure 4B). The *bla*_CTX‐M‐15_ gene was detected in 33 Chinese strains in the main Chinese clade II; this gene is mainly located on the pRY185‐2‐like IncFII plasmid carrying the *bla*_CTX‐M‐15_, *ermB*, and *mphA* genes (Figure 4C). This plasmid has a backbone similar to that of the plasmid p183660 (accession KX008967.1), which was identified in the *S. sonnei* strain 183660 isolated in the UK. The *bla*_CTX‐M‐55_ gene was detected in 28 Chinese strains in the main Chinese clade II, and it was mainly distributed in plasmids with the IncI2 (psn0606‐3) or IncI1‐1(Alpha) (pXJ2012249‐7) backbone. The plasmid psn0606‐3 is highly similar to the plasmid pHN1122‐1 (accession JN797501) of the *E. coli* 1122 strain isolated in China, and the plasmid pXJ2012249‐7 is highly similar to the plasmid unnamed2 (accession CP082826) of the *E. coli* SCAID URN1‐2021 (19/278) strain isolated in Kazakhstan. These two types of plasmids carried only the *bla*_CTX‐M‐55_ gene, with an IS*Ecp1* identified upstream of the *bla*_CTX‐M‐55_ gene (Figure 4D).

Notably, there are two types of plasmid replicons carrying the *mphA* and/or *ermB* gene, one of which belongs to the IncB/O/K/Z backbone, similar to pSH15sh99‐like plasmids found on the main Chinese clade I branch that contain both the *mphA* and *bla*_CTX‐M‐14_ genes. Another plasmid replicon, the pRY185‐2‐like IncFII plasmid found on the main Chinese clade II branch, carries the *mphA*, *ermB*, and *bla*_CTX‐M‐15_ genes, conferring cef^R^azi^R^ resistance (Figure 4C).

**FIGURE 5:**
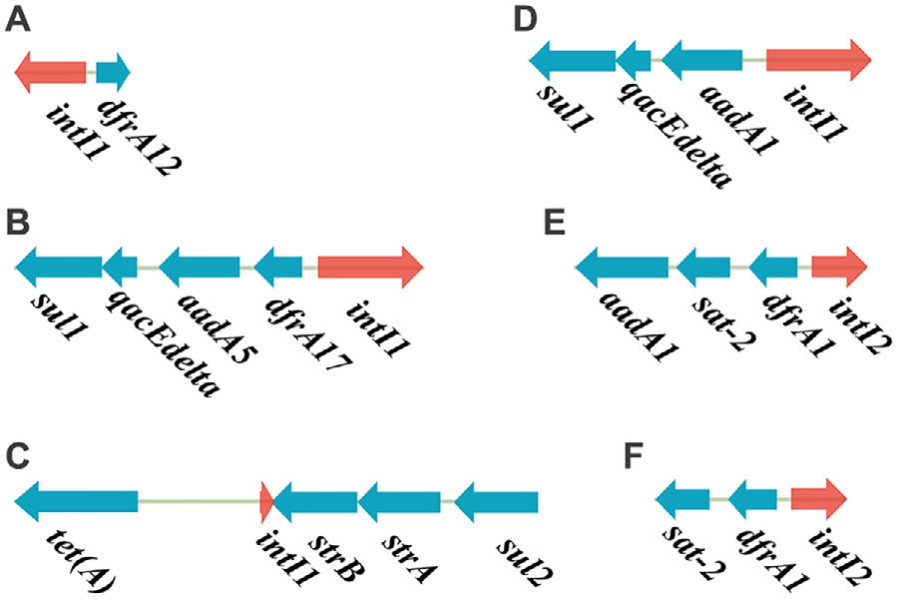
Structures of integrons identified in Chinese *S. sonnei* isolates. (A–F) show the genetic structures of the six distinct types of integrons, annotated with their respective gene cassettes.

Moreover, the *bla*_TEM‐1_, *dfrA12*, *sul2*, *strA*, *strB*, *tet(A)*, and *aac(3)‐IId* genes associated with MDR were mainly located on a plasmid with an IncB/O/K/Z backbone (Figure 4E). In this study, we obtained three plasmids with IncB/O/K/Z backbones, named pHN08160‐1, psh10sh001_3, and pSH12sh288‐1. After blast against the NCBI website, these plasmids showed the highest similarity to plasmid p100 (accession CP093069) of the *E. coli* BR1220 strain from the USA, which carried *sul2*, *strA*, *strB*, *tet(A)*, and *qnrB5* resistance genes. Compared with the p100 plasmid, the three plasmids had a fragment of approximately 17 kb, which contained *bla*_TEM‐1_, *dfrA12*, *aac(3)‐IId*, several transposons, and an *intI1* integrase gene. There is a Tn*3* family transposon, Tn*As1*, downstream of the *bla*_TEM‐1_ gene. Upstream of *dfrA12* was the *intI1* integron, and downstream of *dfrA12* and *intI1*, each had an IS*26* (Figure 4E). We then blasted the three IncB/O/K/Z plasmids against pSH15sh99, with an identity of 73.45%, 73.45%, and 73.44%, respectively. In addition, we identified six types of integrons in the Chinese strains, including five previously reported [29] and one new type of integron, *intI1*, along with the *dfrA12* gene (Figure 5).

Finally, we analyze the global distribution of the large virulence plasmid and the virulence genes of the 743 collected S. sonnei strains (Figure S3). By screening the plasmid pSs046 (Table S3), we find a total of 30.69% (228/743) strains with the plasmid coverage exceeds 90%, accounting for 41.67% (65/156) in overseas strains and 27.77% (163/587) in Chinese strains, especially 25.45% (28/110) of the six waterborne outbreaks (Table S3). The presence of the virulence plasmid (the green stripes) and the distribution of virulence genes (the red stripes) in 743 strains are shown in the heatmap (Figure S3).

### 2.5 The Molecular Mechanism of ONPG‐Negative Isolates

**FIGURE 6:**
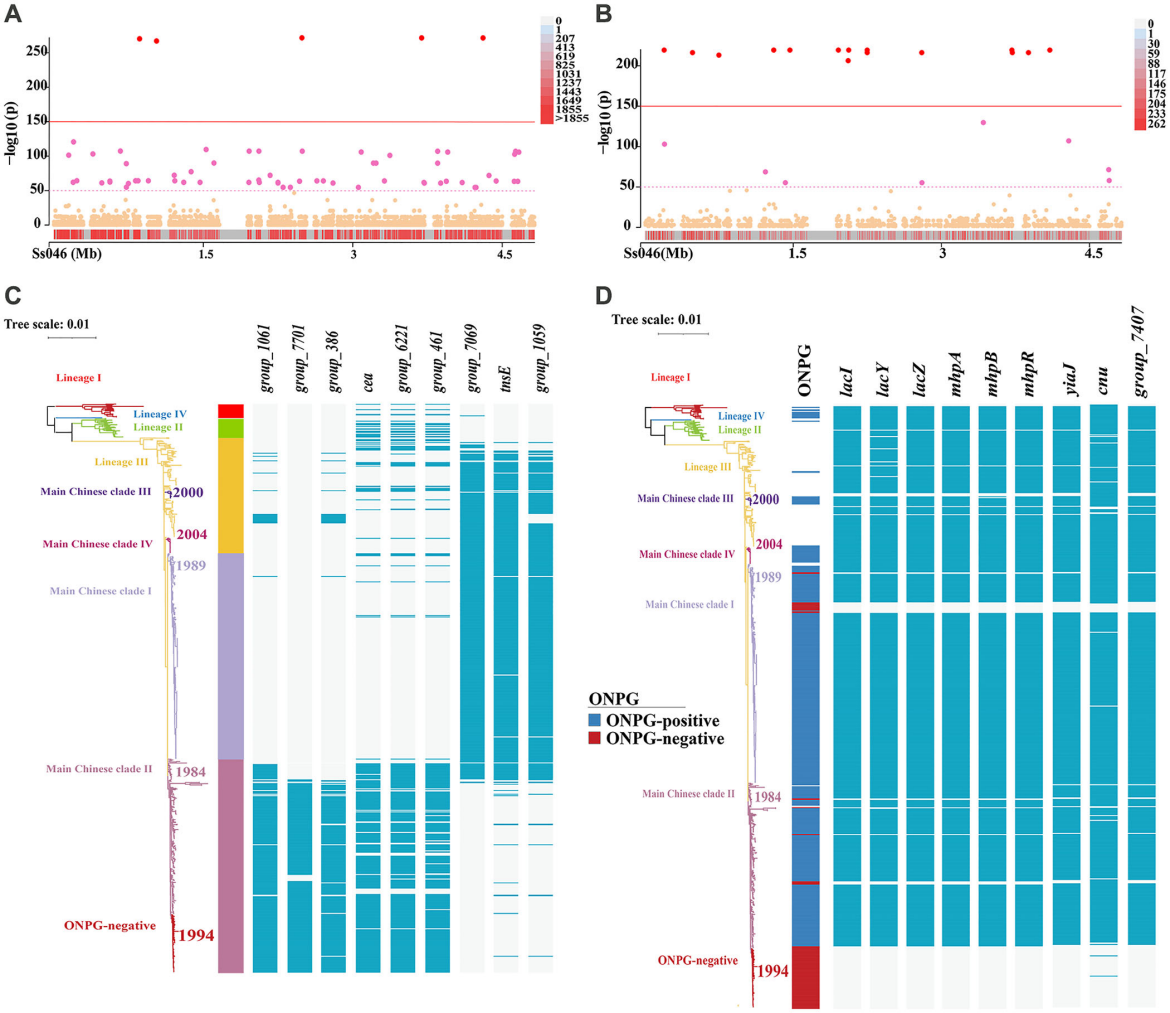
Genetic variants and accessory genes associated with main Chinese clade II and ONPG‐negative phenotypes. Manhattan plots from GWAS highlight SNPs significantly linked to (A) main Chinese clade II and (B) ONPG‐negative variants. (C, D) Accessory genes with clade‐specific distributions identified by pangenome analysis.

To determine the molecular mechanism underlying the ONPG‐negative phenotype, we performed a chi‐square test on the distributions of pangenome genes and mutations to identify genes that were significantly different between the ONPG‐negative and ONPG‐positive isolates. Finally, we identified nine genes associated with ONPG‐negative strains, namely, *lacI*, *lacY*, *lacZ*, *mhpA*, *mhpB*, *mhpC*, *cnu*, *group_7407*, and *yia*J (Figure 6D; Table S4). Then, we analyzed the mutations of the *lac* and *mhp* operons and their flanking regions by comparing the genomes of ONPG‐negative variants to the reference genome of *S. sonnei* Ss046. The results showed that the ONPG‐negative variants, including the most prevalent ONPG‐negative cluster, lacked a 10 kbp nucleotide region spanning the *mhp* operon, *lac* operon required for ONPG hydrolysis [30, 31], and twoIS elements (IS*1* and IS*600*) (Figure S4), consistent with IS*1*‐mediated deletion of the region between the two copies of IS*1* that flank the region in ONPG‐positive genomes. Then, we constructed the Δ*lac* operon mutant, and the mutant showed an ONPG‐negative phenotype when using API 20E strips for biochemical tests (Figure S5A,B). In addition, the Δ*lac* mutant showed no evidence of growth defects compared with the wild‐type strain in LB medium under three different temperature conditions (Figure S5C–E).

### 2.6 Potential Molecular Markers Associated with Main Chinese Clade II and the ONPG‐Negative Phenotype

Genome‐wide association study (GWAS) yielded 66 distinct SNPs (*p* < 1e‐50) significantly associated with the main Chinese clade II, including 61 in the coding regions and five in the intergenic regions (Figure 6A; Table S5). Of the coding region SNPs, 30 were missense mutations, one was an initiator codon variant, and 30 were synonymous mutations. The nonsynonymous SNPs affected 31 genes with various functions, and clusters of orthologous groups (COGs) analysis showed that the functions of those affected genes were mainly associated with carbohydrate transport and metabolism, inorganic ion transport and metabolism, energy production and conversion, and transcription (Figure S6). Genes associated with carbohydrate transport and metabolism include *gatY*, *gcd*, *frvB*, and *frwD*. Genes involved in inorganic ion transport and metabolism mainly included the putative inner membrane component for iron transport gene *ybdE* and nitrite extrusion protein gene *nark*. Genes for energy production and conversion included *pduS*, *yjeS*, and *ygiRQ*. The NADH dehydrogenase I chain L gene *nuoL* and the hydrogenase 4 membrane subunit gene *hyfB* belong to two of the classes. Genes associated with transcription included *yaeO*, *ttk*, and *iclR* (Table S5). Moreover, 23 specific SNPs were identified as being significantly (*p* < 1e‐50) associated with the ONPG‐negative phenotype, including 20 in the coding regions and 3 in the intergenic regions (Figure 6B; Table S6). Of the coding region SNPs, 10 were missense mutations, one was an initiator codon variant, and nine were synonymous mutations. The nonsynonymous SNPs affected five genes encoding conserved hypothetical proteins and six genes with known functions.

Pangenome analysis identified a total of 16,822 genes among the 743 strains, of which 3213 (19.1%) were clustered as core genes (Figure S7). To identify the genes that were significantly different between the strains in main Chinese clade II and on other branches, we performed a chi‐square test on the genes with a *p* value < 1e‐50 and those distributed in more than 80% of strains in main Chinese clade II but fewer than 20% on the other branches or fewer than 20% of strains in main Chinese clade II but more than 80% on other branches. Nine significantly different genes were identified, and the COGs analysis of these genes showed that only two of them had corresponding clusters, including the *cea* gene, which was mainly distributed in strains of the main Chinese clade II, and the *tnsE* gene, which was mainly distributed in strains of other clades (Figure 6C; Table S7).

## 3 Discussion

This work establishes the microevolution of global *S. sonnei* strains, which express different phenotypic and genetic characteristics, including distinct ONPG‐negative variants and a strong MDR phenotype, especially cef^R^azi^R^ coresistance due to different environmental adaptations or different modes of transmission.

The divergent evolution of *S. sonnei* is reflected by the acquisition or loss of some virulence or metabolic genes. In this work, the combination of pangenome analysis and gene editing experiments confirmed that the IS*1*‐mediated deletion of *lac* operon genes, including *lacZ*, *lacY*, and *lacI*, contributed to the ONPG‐negative phenotype. This discrete deletion reflected the ongoing transition of *Shigella* into an obligate pathogen [8], which may represent an evolutionary advantage for more cost‐effective host survival. The detection rate of ONPG‐negative strains is on the rise in China and is not limited to China [32]. Further epidemiological investigation is needed to investigate the spread of this variant.

Widespread antibiotic usage exerts strong selective pressure, favoring the survival and dissemination of strains harboring multiple antimicrobial resistance (AMR) determinants. Globally, antibiotic consumption measured in defined daily doses reached approximately 40.1 billion in 2018, representing a 46% increase since 2000 [33]. China is recognized as one of the world's leading consumers of antibiotics [34], a factor that likely contributes to the high prevalence of MDR *S. sonnei* observed in this study, where 86.37% (507/587) of isolates exhibited MDR. The resistance rate of Chinese strains to older‐generation antibiotics is high, and in recent years, strains resistant to first‐line antibiotics for the treatment of shigellosis, such as cephalosporin, azithromycin, and even colistin, have emerged [29]. Fortunately, the abuse of antibiotics has been monitored [35].

The resistance mechanism is closely related to movable genetic elements. The main Chinese clade I strains mainly acquired the pSH15sh99‐like IncB/O/K/Z plasmid harboring *bla*_CTX‐M‐14_ and *mphA* genes [29], while the main Chinese clade II strains acquired multiple dominant plasmids carrying diverse ESBL genes, and a combination of *mphA* and *ermB* genes conferring cef^R^azi^R^ coresistance. There are six types of integrons found in Chinese strains (Figure S1), and the integron‐related genes included *dfrA1*, *dfrA12*, and *dfrA17*, conferring resistance to trimethoprim; *aadA1*, *aadA5*, *strA*, *strB*, and *sat‐2* conferring resistance to aminoglycosides, and *sul1* and *sul2* conferring resistance to sulfonamide [4, 23]. Notably, the integron *intI1*, along with the *dfrA12* gene, was widely distributed in the main Chinese clade II.

However, this study also has some shortcomings. First, our work mainly focuses on bioinformatics analysis and lacks wet experiment validation. Second, the collected strains only cover 14 provinces of China, and are relatively concentrated in terms of time, which may lead to a certain sample bias. Our study suggested that continued surveillance and further genomic epidemiological studies are urgently required to detect epidemics, and it is one of our future directions.

In conclusion, our study delineates the divergent microevolution and local establishment of MDR S. *sonnei* in China. We identified two predominant clades with distinct antibiotic resistance profiles and plasmid types driving cef^R^azi^R^ coresistance. Furthermore, we elucidated the molecular mechanism behind the emerging ONPG‐negative phenotype, linked to an IS*1*‐mediated deletion. The coexistence of these successful clones, equipped with diverse resistance mechanisms and adaptive mutations, highlights a significant and evolving public health threat in China. Continuous national surveillance, combined with genomic epidemiology, is crucial to track the transmission and further evolution of these resistant clones and to inform effective control strategies.

## 4 Materials and Methods

### 4.1 Strain Collection

In this study, a total of 587 *S. sonnei* strains isolated from 1999 to 2020 in China were selected for whole‐genome sequencing (WGS), including 477 sporadic strains, which were randomly selected, and 110 outbreak strains of six outbreaks. These strains were recovered from 14 regions of China (Figure 1; Table S8). These samples contained previously reported strains from six cef^R^azi^R^ MDR waterborne outbreaks [29], and emergent ONPG‐negative MDR strains [18], alongside newly identified sporadic cef^R^azi^R^ MDR strains (Table S8). To determine their evolutionary relationships, we performed bioinformatics analysis on 587 genomes, including 281 cef^R^azi^R^ isolates and 99 ONPG‐negative isolates, along with 156 previously published reference genomes used by Holt et al. (Table S8) [4, 20].

### 4.2 High Throughput Sequencing

Bacterial genomic DNA was extracted with the QIAamp DNA Mini Kit (Qiagen, Germany). Whole‐genome sequencing was conducted on Illumina HiSeq or MiSeq platforms following standard library preparation protocols. Raw sequencing reads were subjected to quality trimming with Trimmomatic v0.39 [36] and subsequently assembled de novo using SPAdes v3.15.2 [37].

To obtain complete plasmid sequences, we also selected seven representative strains for Oxford Nanopore sequencing using a GridION device. Hybrid assembly of Illumina and Nanopore sequencing data was performed with Unicycler [38] to generate high‐quality genomes and plasmid assemblies. We use Bowtie2 [39] for aligning and mapping the sequencing reads, and SAMtools [40] to convert the format of result. The assembly details of all strains are provided in Table S9.

### 4.3 Microbiological Investigation

Strain identification involved biochemical characterization with the API 20E system (bioMérieux, France) coupled with serological typing using a commercial antiserum kit (Denka Seiken, Japan). Initial screening for the ONPG phenotype was conducted on CHROMagar chromogenic medium (CHROMagar Microbiology, France), with subsequent confirmation via API test strips (bioMérieux). For antimicrobial susceptibility, the broth microdilution method was employed in 96‐well microtiter plates (Sensititre, Trek Diagnostic Systems, Thermo Fisher Scientific Inc.), following the manufacturer's protocol. MICs were determined and interpreted in alignment with CLSI standards [41]. The reference strain ATCC 25922 was included in all MIC assays. Specifically, the MICs for colistin (0.5–32 µg/mL) and azithromycin (4–256 µg/mL) were established using the broth microdilution technique.

### 4.4 Phylogenetic Analysis

To ensure reliable lineage delineation given the genetic heterogeneity of *S. sonnei*, we used two pipelines, RedDog and Snippy (v4.6.0), to generate phylogeny, respectively. The complete genome of *S. sonnei* strain Ss046 (accession NC_007384), a widely used reference in prior studies, was employed for the SNP analysis [4, 20, 42].

Using the RedDog pipeline [4], SNPs were called with SAMtools [40] and filtered as previously described [4]. A maximum‐likelihood phylogenetic tree was constructed to explore population structure by using FastTree v2.1.8 [43]. To infer the evolutionary dynamics, we selected 371 strains with known isolation dates and regions for Bayesian phylogenetic analysis using Bayesian Evolutionary Analysis Sampling Trees (BEAST2) [44]. A Bayesian skyline model with a log‐normally distributed clock rate was chosen, and Markov chain Monte Carlo (MCMC) generations were set with 300 million steps and samples taken every 5000 generations in six independent MCMC chains. Finally, we burned in the first 10% steps of each chain and combined the log files of these chains to generate a maximum clade credibility (MCC) tree, visualized with Figtree (http://tree.bio.ed.ac.uk/software/figtree/). The genotypes of these strains were assigned using Mykrobe3 according to the framework of Hawkey et al. [45], and the phylogenetic tree was annotated using iTOL (https://itol.embl.de/) to display distributions of AMR genes, plasmid replicons, and other mobile elements. Similarly, for the Snippy pipeline (https://github.com/tseemann/snippy), we used SAMtools [40] and FreeBayes [46] for SNP identification, and used SNP‐sites [47] to extract the core SNPs, and finally constructed the phylogenetic tree by IQ‐TREE [48].

### 4.5 Identification of AMR Genes and Mobile Genetic Elements

In this work, MDR was defined as resistance to three or more antibiotic classes. We evaluated susceptibility against a panel of 20 antibiotics spanning 12 distinct classes (Table S1), categorized per CLSI guidelines. Following de novo assembly, the resulting contigs were annotated with Prokka [49]. To identify antimicrobial resistance genes, both chromosomal and plasmid‐derived sequences from the genome assemblies were analyzed using the Resistance Gene Identifier (RGI) v5.1.1, querying the Comprehensive Antibiotic Resistance Database (CARD) [50]. Fluoroquinolone resistance‐associated mutations in the *gyrA* and *parC* genes were extracted from the genome‐wide SNP calls. The horizontal transfer of AMR genes is usually associated with integrons, transposons, plasmids, or other elements, so we performed an analysis of the gene cassette arrays and transposons by mapping the assemblies to the INTEGRALL [51] database and MobileElementFinder [52] using BLAST v2.5.0 [53]. Complete plasmids harboring AMR genes such as *bla*_CTX‐M‐14_, *bla*_CTX‐M‐15_, *bla*_CTX‐M‐55_, *bla*_TEM‐1_, *ermB*, and *mphA* were resolved by hybrid assembly (Illumina MiSeq and Oxford Nanopore GridION) of seven representative strains.

Prokka was utilized for plasmid annotation, and replicon types were assigned with PlasmidFinder [54]. Insertion sequences (ISs) and AMR genes located on plasmids were characterized with ISfinder and ResFinder v4.0.1, respectively. Plasmid sequences harboring AMR genes were subjected to BLAST analysis against the NCBI database to identify closely related reference plasmids. For comparative visualization, custom Perl scripts processing GBK‐format files were developed. To ascertain plasmid carriage, genomic assemblies were aligned to the candidate plasmid sequences via BLAST, applying thresholds of >90% coverage and >70% nucleotide identity. Furthermore, the presence of plasmid‐associated AMR genes within the assemblies was taken as indicative of strains harboring identical or structurally analogous plasmids.

### 4.6 Identification of SNPs and Accessory Genes Associated with the Main Chinese Clades

To identify main Chinese clade‐related SNPs, we performed a GWAS using Plink [55]. SNPs were called from 743 strains against the chromosome of *S. sonnei* Ss046 using Snippy (v 4.6.0). The strains were divided into the main Chinese clade and the non‐main Chinese clade groups. Association significance was tested by the chi‐square test followed by FDR correction. We selected SNPs with a *p*‐value < 1e‐50 as the significant SNPs and a *p*‐value < 1e‐100 as the highly significant SNPs. Genes containing these SNPs were subjected to COGs analysis.

To identify main Chinese clade‐related accessory genes, pangenome analysis was performed using the Roary [56] pipeline on Prokka‐annotated genomes. Gene distribution differences between the main Chinese clade branch and other branches were evaluated by chi‐square tests (*p* < 0.01). Furthermore, we selected genes present in ≥80% of the main Chinese clade strains but ≤20% of others, or vice versa, as significant accessory genes. All identified genes from both analyses were mapped against the COGs database using BLAST.

### 4.7 Identification of Accessory Genes and SNPs Associated with the ONPG‐Negative Phenotype

To explore the genetic changes at the genomic level of strains with the ONPG‐negative phenotype, we performed pangenomic analysis and GWAS to determine the accessory genes and SNPs associated with the ONPG‐negative phenotype. The methods were the same as those described above.

### 4.8 Identification of the Molecular Mechanism Conferring the ONPG‐Negative Phenotype

We used Mummer [57] to compare the genomes of ONPG‐negative strains with Ss046 to identify structural variations associated with ONPG‐negative variants. To determine whether deletion of the *lac* operon is the main driver of the biochemical characteristics of ONPG‐negative variants, we constructed a *lac* operon deletion mutant in the wild‐type ONPG‐positive strain CICC 21535 by utilizing the pKD46 plasmid with the λ‐Red recombination system [58]. Briefly, a fusion fragment containing the FRT‐flanked kanamycin resistance gene and regions upstream and downstream of the *lac* operon was amplified, using the primers listed in Table S10, and then transferred into the recipient strain CICC 21535 carrying the pKD46 plasmid. The mutants were identified by screening transformants on kanamycin plates, and then the kanamycin resistance gene was eliminated by the pCP20 plasmid. The Δ*lac* operon mutants were further verified by PCR amplification and sequencing, and the biochemical phenotype of the mutant was confirmed by using CHROMagar chromogenic medium and API biochemical tests. The growth curve experiments of the wild‐type strain and its Δ*lac* operon mutant were conducted under different temperature conditions by using a previously described method [58].

### 4.9 Statistical Analysis

We calculated statistics for the classes of drugs to which the strains are resistant and the number of AMR genes carried by the strains. Between‐group data comparisons were performed using the Mann‒Whitney test for two‐group comparisons and the Kruskal‒Wallis test for multiple group comparisons. For categorical variables, including MDR rates and individual antibiotic resistance patterns, chi‐square tests were employed. Fisher's exact test was used when expected frequencies were small. To account for multiple testing in individual antibiotic resistance comparisons, the FDR procedure was applied using the Benjamini–Hochberg method. In addition, we performed a chi‐square test on the rate of pangenome detection in ONPG‐negative and positive bacteria and on the main Chinese clade II branch and other branches. Genes with *p*‐values below 1e‐50 were used as ONPG‐phenotype or branch‐related genes. *p*‐values less than 0.05 were considered statistically significant, with FDR‐adjusted *p*‐values < 0.05 considered significant for multiple comparisons. All statistical analyses were performed using R v4.2.0.

## Author Contributions

S.F.Q., J.T.Y., X.B.X., and H.B.S. conceived and designed the experiments. K.K.L., J.W., C.J.Y., X.G., Hb.L., X.Y.D., Y.X., K.Y.M., J.F.L., H.B.L., X.Y.L., H.W., C.W., Q.W., H.Q.J., M.J.Y., L.G.W., Y.S., M.M., J.Y.Z., S.J.L., D.S.Y., and X.B.X. performed the experiments. S.F.Q., K.K.L., J.W., C.J.Y., X.G., J.H., K.E.H., and S.B. analyzed the data. S.F.Q., K.K.L., J.W., C.J.Y., X.G., K.Y.M., J.F.L., S.B., and J.T.Y. wrote the paper. J.T.Y. is the last senior author of this paper. All authors reviewed the manuscript critically for content and approved the decision to submit for publication.

## Ethics Approval

Not required.

## Conflicts of Interest

The authors declare no conflicts of interest.

## Supporting information



Supporting File 1: mco270569‐sup‐0001‐SuppMat.pdf

Supporting File 2: mco270569‐sup‐0002‐FigureS4.pdf

Supporting File 3: mco270569‐sup‐0003‐FigureS5.pdf

Supporting File 4: mco270569‐sup‐0004‐FigureS6.pdf

Supporting File 5: mco270569‐sup‐0005‐FigureS7.pdf

Supporting File 6: mco270569‐sup‐0006‐TableS1.xlsx

Supporting File 7: mco270569‐sup‐0007‐TableS2.xlsx

Supporting File 8: mco270569‐sup‐0008‐TableS3.xlsx

Supporting File 9: mco270569‐sup‐0009‐TableS4.xlsx

Supporting File 10: mco270569‐sup‐0010‐tableS8.xlsx

Supporting File 11: mco270569‐sup‐0011‐TableS9.xlsx

Supporting File 12: mco270569‐sup‐0012‐TableS10.xlsx

## Data Availability

All sequence data in this study have been submitted to the NCBI Sequence Read Archive under BioProject number PRJNA835603, and the accession numbers for the data can be found in Table S8. The complete plasmid sequences have been deposited in NCBI under accessions OQ230385‐OQ230390 and OR506643‐OR506644.
