# Supplementary material for: Divergent Evolution and Local Establishment of Multidrug‐Resistant Shigella sonnei in China
Source: MedComm (2020). 2026 Jan 7;7(1):e70569. doi: 10.1002/mco2.70569 (PMC12778403; doi:10.1002/mco2.70569)
Supplement: Supplementary file 1 — Supporting File 1: mco270569‐sup‐0001‐SuppMat.pdf [file MCO2-7-e70569-s005.pdf]

Supplementary Information

Divergent evolution and local establishment of multi-drug-resistant *Shigella sonnei* in China

Kangkang Liu[1,2#], Jian Wang[3#], Chaojie Yang[1#], Yehong Yang[4#], Xin Ge[1,5], Hongbo Liu[1], Xinying Du[1], Ying Xiang[1], Kaiyuan Min[4], Qi Wang[1], Hui Wang[1], Chao Wang[1], Huiqun Jia[1], Mingjuan Yang[1], Xiaoying Li[1], Ligui Wang[1], Yong Sun[6], Muti Mahe[7], Jiayong Zhao[8], Shijun Li[9], Deshan Yu[10], Stephen Baker[11], Jiangfeng Liu[4,13]*, Xuebin Xu[12]*, Hongbin Song[1]*, Shaofu Qiu[1]*, Juntao Yang[4,13]

[1]Center for Disease Control and Prevention of Chinese PLA, Beijing, China.

[2]State Key Laboratory of Pathogen and Biosecurity, Beijing Institute of Microbiology and Epidemiology, Beijing, China.

[3]Beijing Key Laboratory of Surveillance, Early Warning and Pathogen Research on Emerging Infectious Diseases; Beijing Research Center for Respiratory Infectious Diseases; Beijing Center for Disease Prevention and Control, Beijing 100013, China.

[4]State Key Laboratory of Common Mechanism Research for Major Diseases, Institute of Basic Medical Sciences Chinese Academy of Medical Sciences, School of Basic Medicine Peking Union Medical College, Beijing, China.

[5]Department of Epidemiology and Biostatistics, School of Public Health, Anhui Medical University, Hefei, China.

[6]Anhui Provincial Center for Disease Control and Prevention, Hefei, China.

[7]Center for Disease Control and Prevention of Xinjiang Uygur Autonomous Region, Urumqi, China.

[8]Henan Provincial Center for Disease Control and Prevention, Zhengzhou, China.

[9]Guizhou Provincial Center for Disease Control and Prevention, Guiyang, China.

[10]Gansu Provincial Center for Disease Control and Prevention, Lanzhou, China.
[11]University of Cambridge School of Clinical Medicine, Cambridge Biomedical Campus, Cambridge, United Kingdom.
[12]Shanghai Municipal Center for Disease Control and Prevention, Shanghai, China.
[13]Institute of Pathogen Biology, Chinese Academy of Medical Sciences & Peking Union Medical College, Beijing, China.

[#]These authors contributed equally: Kangkang Liu, Jian Wang, Chaojie Yang, and Yehong Yang.

Juntao Yang is the last senior author

*Corresponding authors:
Xuebin Xu, Shanghai Municipal Center for Disease Control and Prevention, Shanghai 200336, China. E-mail: xxb72@sina.com.

Jiangfeng Liu, State Key Laboratory of Common Mechanism Research for Major Diseases, Institute of Basic Medical Sciences Chinese Academy of Medical Sciences, School of Basic Medicine Peking Union Medical College; Institute of Pathogen Biology, Chinese Academy of Medical Sciences & Peking Union Medical College, Beijing 100005, China. E-mail: ljf@pumc.edu.cn.

Hongbin Song, the Chinese PLA Center for Disease Control and Prevention, Beijing 100071, China. E-mail: hongbinsong@263.net.
Shaofu Qiu, the Chinese PLA Center for Disease Control and Prevention, Beijing 100071, China. E-mail: qiushf0613@hotmail.com.

SUPPLEMENTARY METHODS

Definition of Lineage

Lineages typically refer to the continuous evolutionary path from a common ancestor to modern species. They represent the continuity and temporal depth of biological evolution. On the evolutionary tree, a lineage can be seen as a continuous path from the root to the top, representing the evolutionary process of a species or a group of species. Lineages emphasize the continuity of species or genomes, as well as their direct evolutionary relationships.

Definition of Clusters

Clusters typically refer to a group of species or genes that are clustered together on an evolutionary tree due to a common ancestral relationship. These species or genes have maintained close phylogenetic relationships during the evolutionary process. In the topology of the evolutionary tree, clusters appear as a set of interconnected branches that share a closer common ancestor.

Comparison of Lineages and Clusters

By comparison, lineages mainly focus on the continuous evolutionary paths of individual species or genomes, while clusters emphasize the tight group formed by a group of species or genes due to common ancestral relationships. Clusters may contain multiple lines that diverge at some point in the evolutionary tree, but prior to this, their common ancestors tightly linked them together. Both play important roles in evolutionary analysis, helping researchers understand the origin and evolution of biodiversity.

Reference

[1] Didelot X, Maiden MCJ. Impact of recombination on bacterial evolution. Trends Microbiol. 2010; 18(7):315-22.

[2] Comas I, Coscolla M, Luo T, et al. Out-of-Africa migration and Neolithic coexpansion of *Mycobacterium tuberculosis* with modern humans. Nat Genet. 2013; 45(10):1176-82.

[3] Kaper JB, Nataro JP, Mobley HL. Pathogenic *Escherichia coli*. Nature Reviews Microbiology. 2004; 2(2):123-140.

SUPPLEMENTARY FIGURE LEGENDS

Figure S1. Phylogenetic tree of *S. sonnei* isolates.

A. Circular phylogenetic tree generated by Reddog. **B.** Circular phylogenetic tree generated by Snippy. **C.** Rectangular phylogenetic tree generated by Reddog. **D.** Rectangular phylogenetic tree generated by Snippy.

Figure S2. High-resolution phylogenetic tree of *S. sonnei* isolates with bootstrap values.

Figure S3. The global distribution of the large virulence plasmid and the virulence genes of the *S. sonnei* strains.

Figure S4. Comparison of the *lac* operon and its flanking regions among the ONPG-negative and ONPG-positive *S. sonnei* isolates.

The genome-sequenced isolate Ss046 was used as a reference. The isolate CICC 21535 represented the ONPG-positive isolates, and the isolate 1943 represented the ONPG-negative isolates. The Δlac operon mutant isolate was derived from the CICC

21535 wild-type strain. The coding genes, transposons and deletion regions are labeled in different colors.

Figure S5. Biological characteristics of the wild-type *S. sonnei* isolate and its Δ*lac* operon mutant.

A and **B** represent the biochemical characteristics of the wild-type *S. sonnei* isolate CICC 21535 and its Δ*lac* operon mutant, respectively, identified by API 20E test strips. **C** to **E** represent the growth curves of the wild-type *S. sonnei* strain CICC 21535 and its Δ*lac* operon mutant at 25 °C (**D**), 30 °C (**D**) and 37 °C (**E**). Data represent the mean from at least three separate experiments.

Figure S6. COG function classifications of SNPs with *P* < 1e-50 associated with MCC II.

The SNP-containing genes are mainly associated with carbohydrate transport and metabolism, inorganic ion transport and metabolism, energy production and conversion, transcription and so on.

Figure S7. Pangenome analysis of the global *S. sonnei* isolates.

A total of 16,822 genes were identified by pangenome analysis based on the 743 isolates, 13,609 of which were assigned to the accessory genome. **A** shows a heatmap displaying the distribution of the pangenome among isolates. Dark blue indicates the presence of a gene, and light blue indicates the absence of a gene. **B** shows the proportions of core and accessory genes.

SUPPLEMENTARY TABLES

Table S1. Antimicrobial resistance of the Chinese *S. sonnei* isolates, including the ONPG-negative variants

Table S2. Antibiotic resistance patterns of the Chinese *S. sonnei* isolates in this study

Table S3. The presence of the large virulence plasmid pSs046 in 743 *S. sonnei* strains.

Table S4. Accessory genes associated with ONPG-negative variants

Table S5. SNPs significantly associated with the MCC II clade

Table S6. SNPs significantly associated with ONPG-negative variants

Table S7. Accessory genes associated with MCC II and other branches

Table S8. Metadata of the *Shigella sonnei* strains used in this study

Table S9. Details of assembly statistics of the strains.

Table S10. The primers used in this study

Table S11. Abbreviations and their full names used in this study

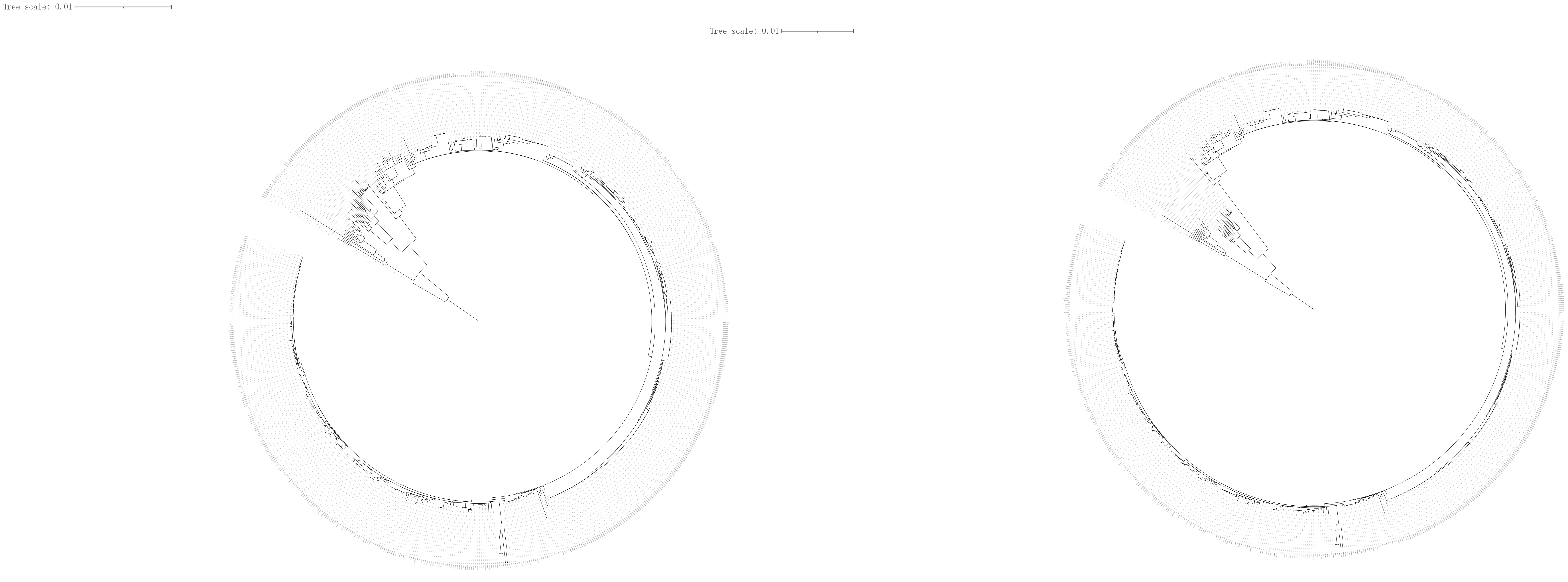

Reddog-tree

snippy-tree

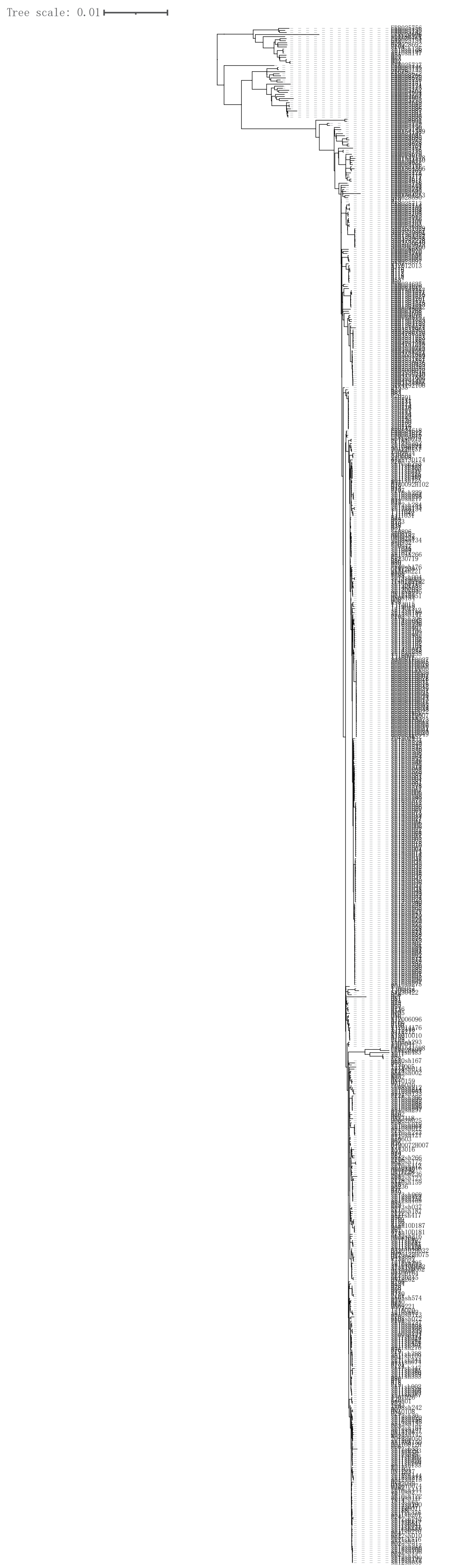

Reddog-tree

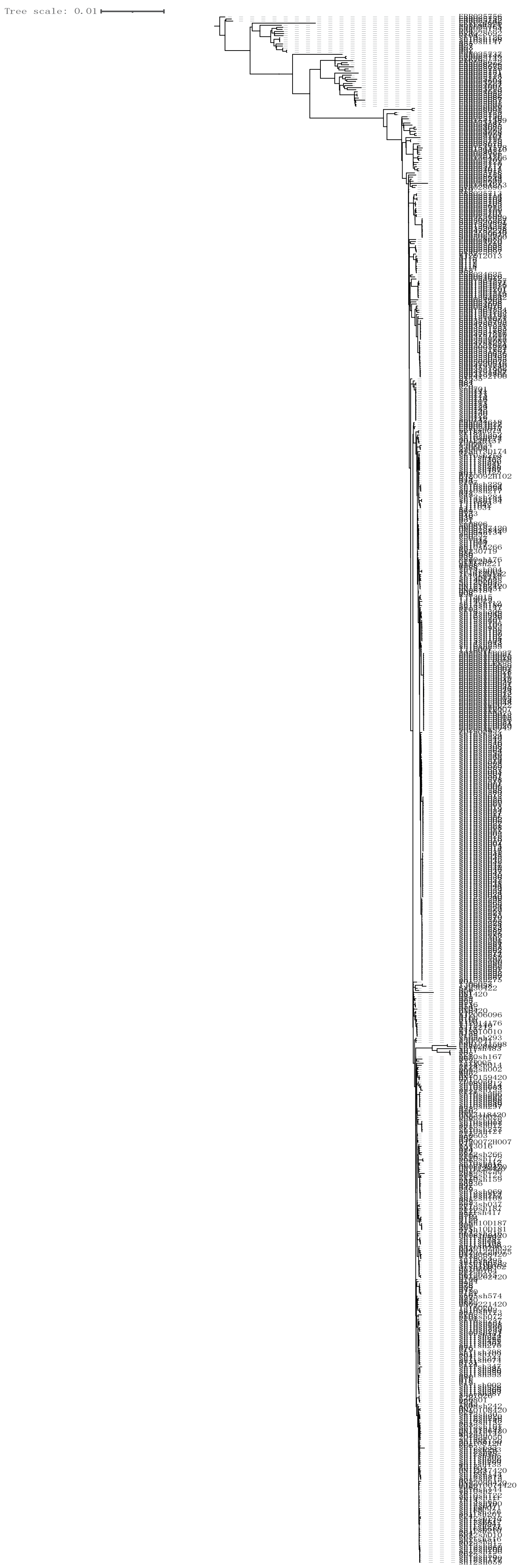

snippy-tree

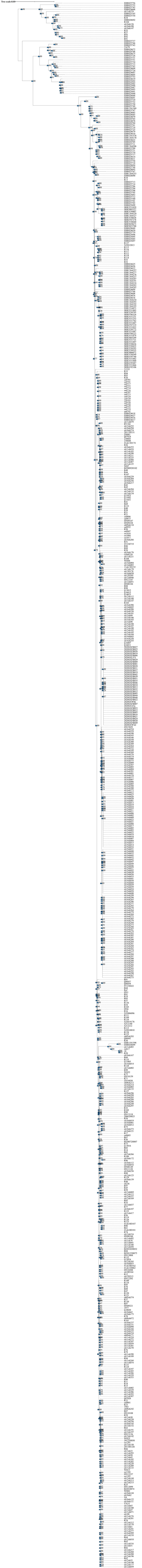
Tree scale:0.01
ERR025756
ERR025755
ERR025742
ERR024606
sh11sh374
ERR025765
ERR025754
R34
ERR028692
R104
sh10sh126
sh10sh108
sh10sh147
R55
R27
R93
R92
R91
ERR025737
ERR025746
ERR025743
21679
ERR028672
ERR028706
ERR028675
ERR025726
ERR025731
ERR025751
ERR025734
ERR025715
ERR025762
ERR024604
ERR025724
ERR024607
ERR024605
ERR025753
ERR024619
ERR025692
ERR025682
ERR025686
ERR025687
ERR025693
ERR025691
ERR025690
ERR028688
ERR028691
ERR025729
ERR025732
ERR025750
ERR025749
ERR1541589
ERR025747
ERR024085

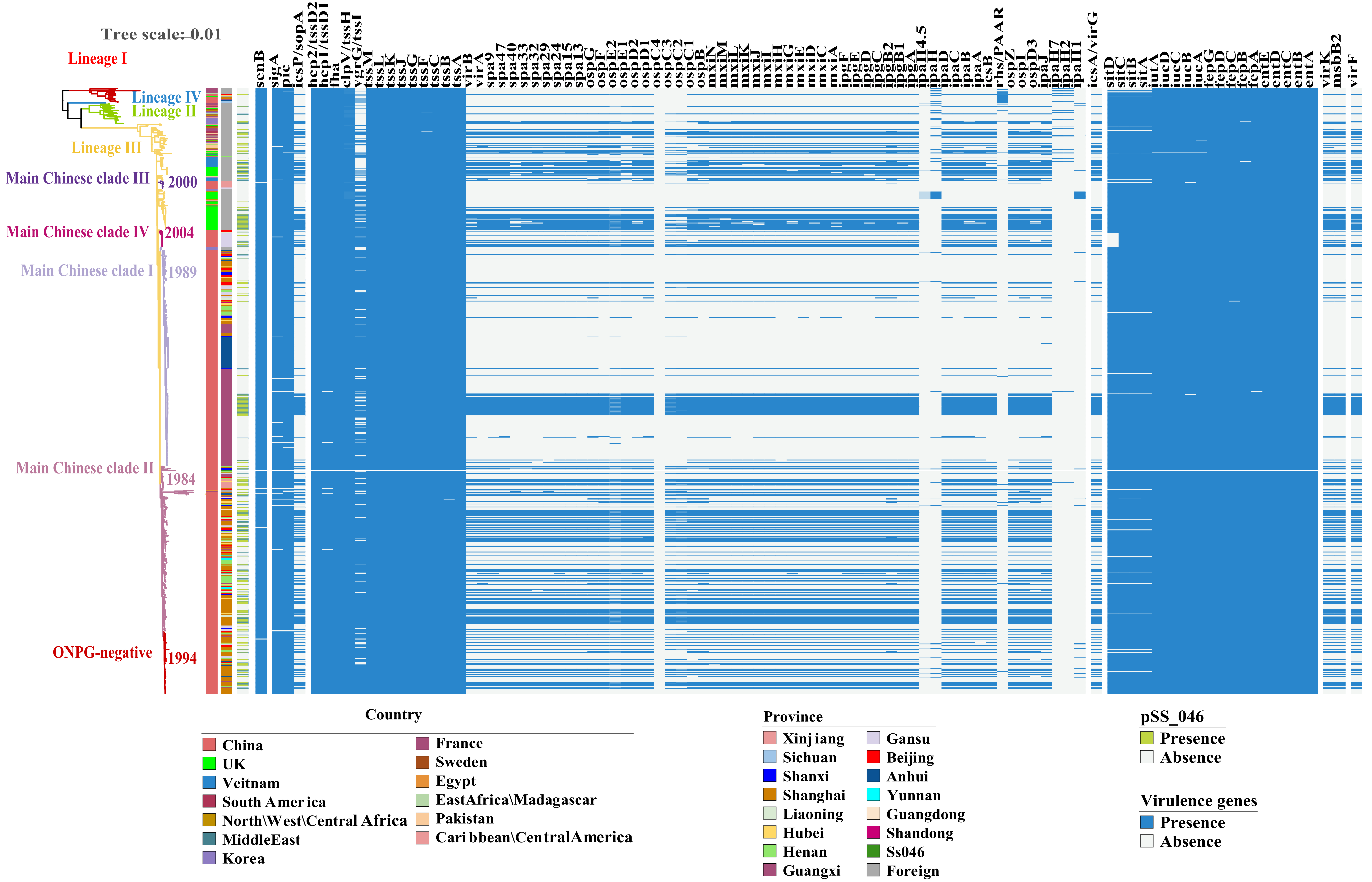

Tree scale: 0.01
Lineage I
Lineage IV
Lineage II
Lineage III
Main Chinese clade III
2000
Main Chinese clade IV
2004
Main Chinese clade I
1989
Main Chinese clade II
1984
ONPG-negative
1994
senB
sigA
pic
icsP/sopA
hcp2/tssD2
hcp1/tssD1
fha
clpV/tssH
vgrG/tssI
tssM
tssL
tssK
tssJ
tssG
tssF
tssC
tssB
tssA
virB
virA
spa9
spa47
spa40
spa33
spa32
spa29
spa24
spa15
spa13
ospG
ospF
ospE2
ospE1
ospD2
ospD1
ospC4
ospC3
ospC2
ospC1
ospB
mxiN
mxiM
mxiL
mxiK
mxiJ
mxiI
mxiH
mxiG
mxiE
mxiD
mxiC
mxiA
ipgF
ipgE
ipgD
ipgC
ipgB2
ipgB1
ipgA
ipaH4.5
ipaH
ipaD
ipaC
ipaB
ipaA
icsB
rhs/PAAR
ospZ
ospI
ospD3
ipaJ
ipaH7
ipaH2
ipaH1
icsA/virG
sitD
sitC
sitB
sitA
iutA
iucD
iucC
iucB
iucA
fepG
fepD
fepC
fepB
fepA
entE
entD
entC
entB
entA
virK
msbB2
virF
Country
China
UK
Veitnam
South America
North\West\Central Africa
MiddleEast
Korea
France
Sweden
Egypt
EastAfrica\Madagascar
Pakistan
Caribbean\CentralAmerica
Province
Xinjiang
Sichuan
Shanxi
Shanghai
Liaoning
Hubei
Henan
Guangxi
Gansu
Beijing
Anhui
Yunnan
Guangdong
Shandong
Ss046
Foreign
pSS_046
Presence
Absence
Virulence genes
Presence
Absence

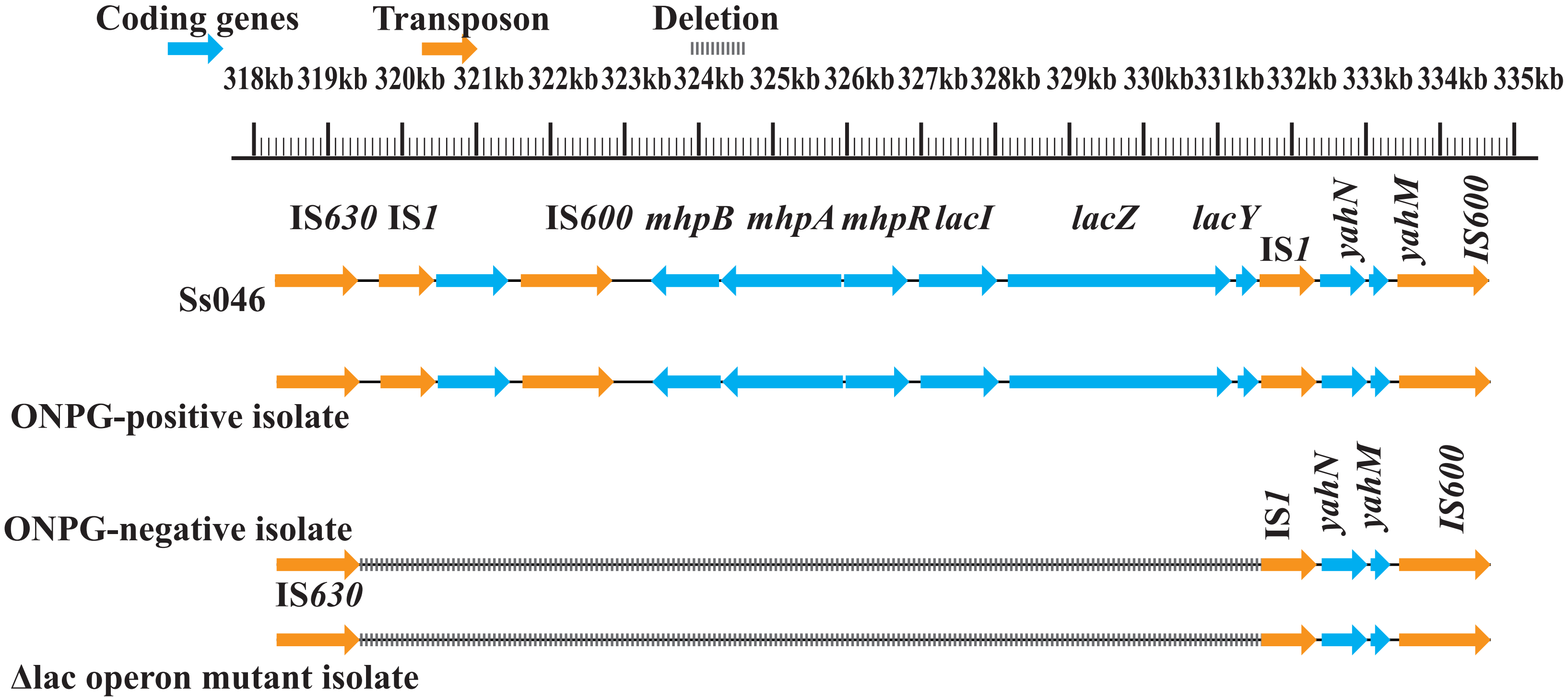

Coding genes
Transposon
Deletion
318kb 319kb 320kb 321kb 322kb 323kb 324kb 325kb 326kb 327kb 328kb 329kb 330kb 331kb 332kb 333kb 334kb 335kb
IS*630* IS*1* IS*600* *mhpB* *mhpA* *mhpR* *lacI* *lacZ* *lacY* IS*1* *yahN* *yahM* *IS600*
Ss046
ONPG-positive isolate
ONPG-negative isolate
IS*1* *yahN* *yahM* *IS600*
IS*630*
Δlac operon mutant isolate

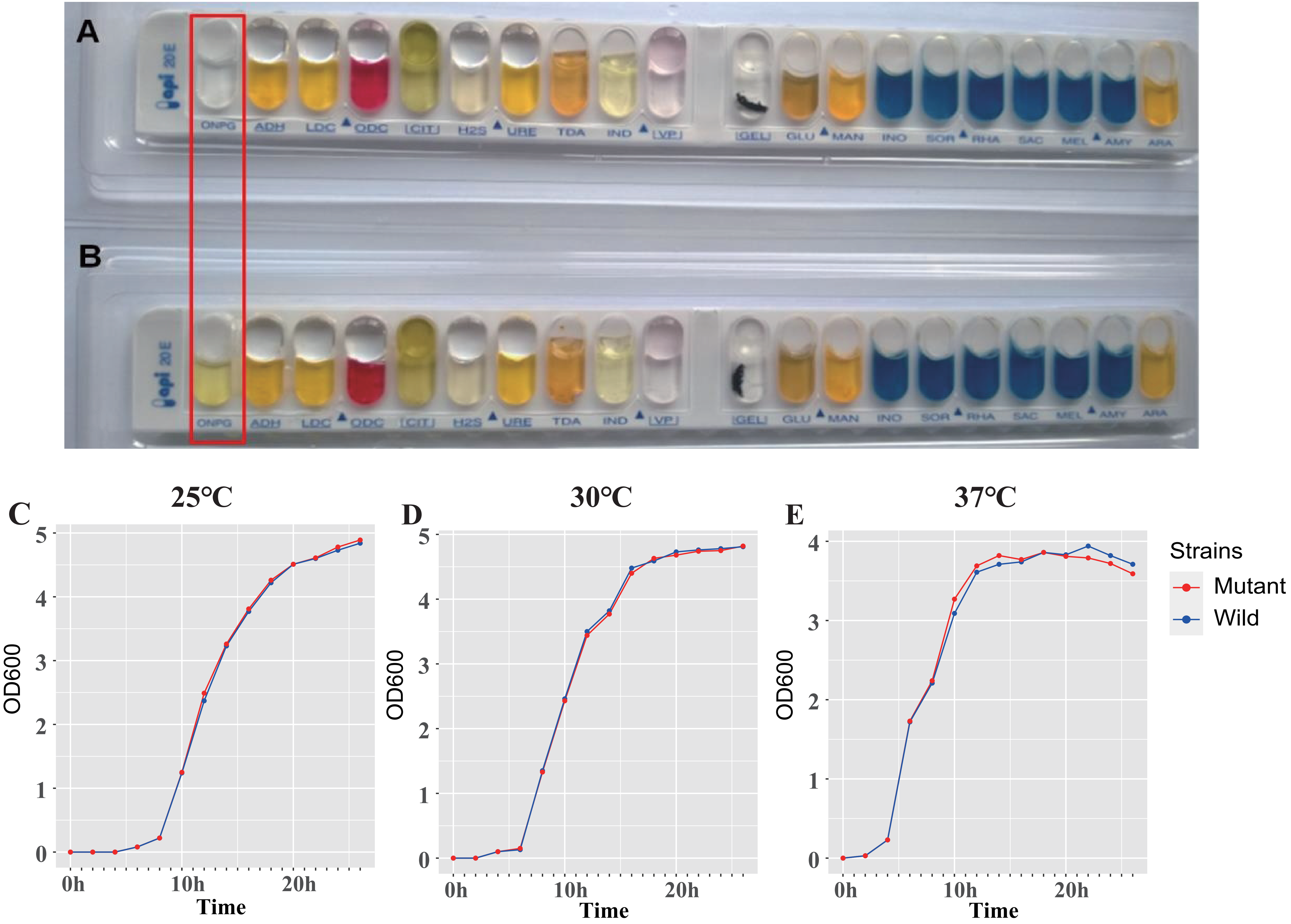

A
B
api 20E
ONPG
ADH
LDC
ODC
CIT
H2S
URE
TDA
IND
VP
GEL
GLU
MAN
INO
SOR
RHA
SAC
MEL
AMY
ARA
C
D
E
25°C
30°C
37°C
OD600
Time
0h
10h
20h
0
1
2
3
4
5
Strains
Mutant
Wild

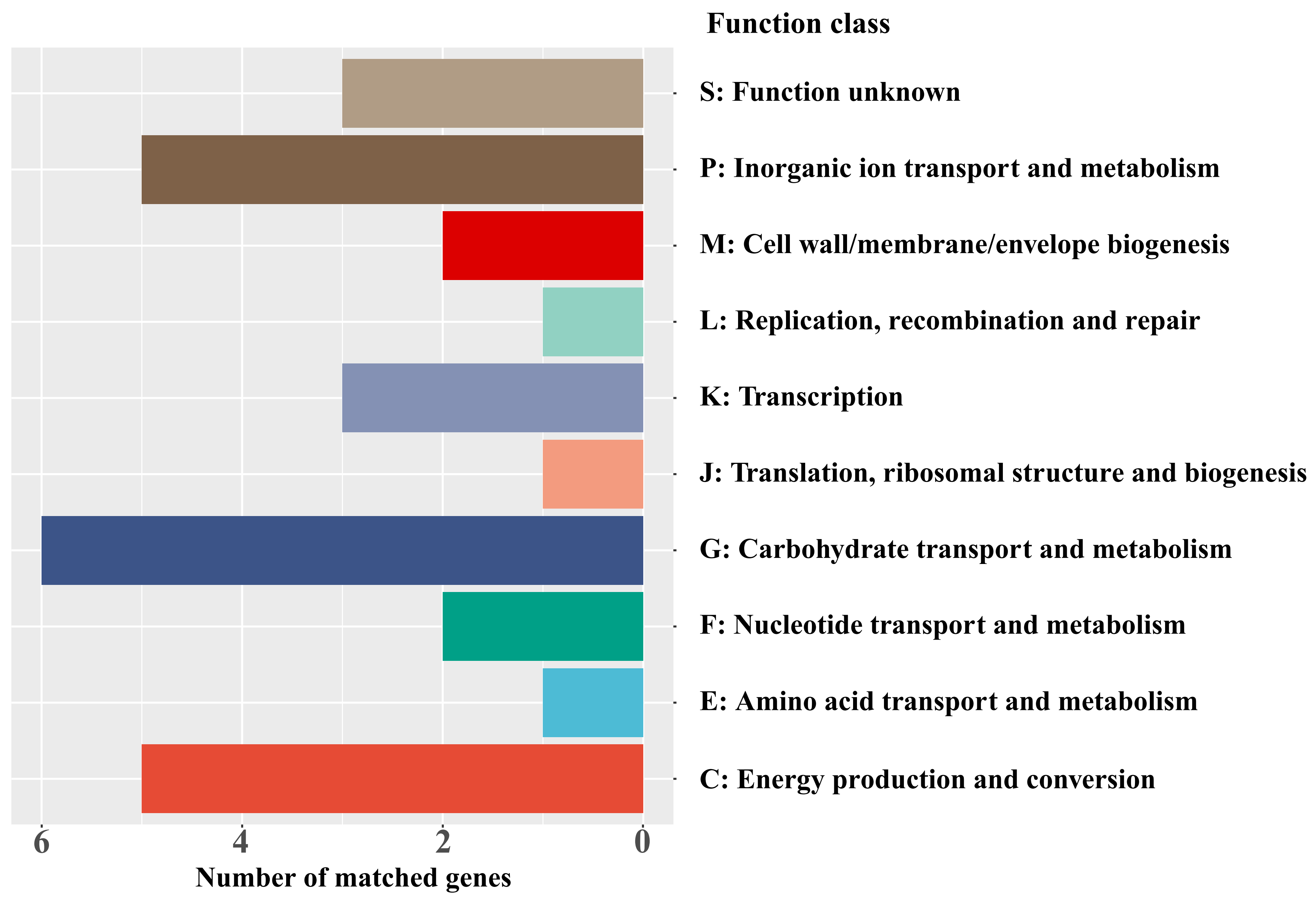

Function class
S: Function unknown
P: Inorganic ion transport and metabolism
M: Cell wall/membrane/envelope biogenesis
L: Replication, recombination and repair
K: Transcription
J: Translation, ribosomal structure and biogenesis
G: Carbohydrate transport and metabolism
F: Nucleotide transport and metabolism
E: Amino acid transport and metabolism
C: Energy production and conversion
6
4
2
0
Number of matched genes

A

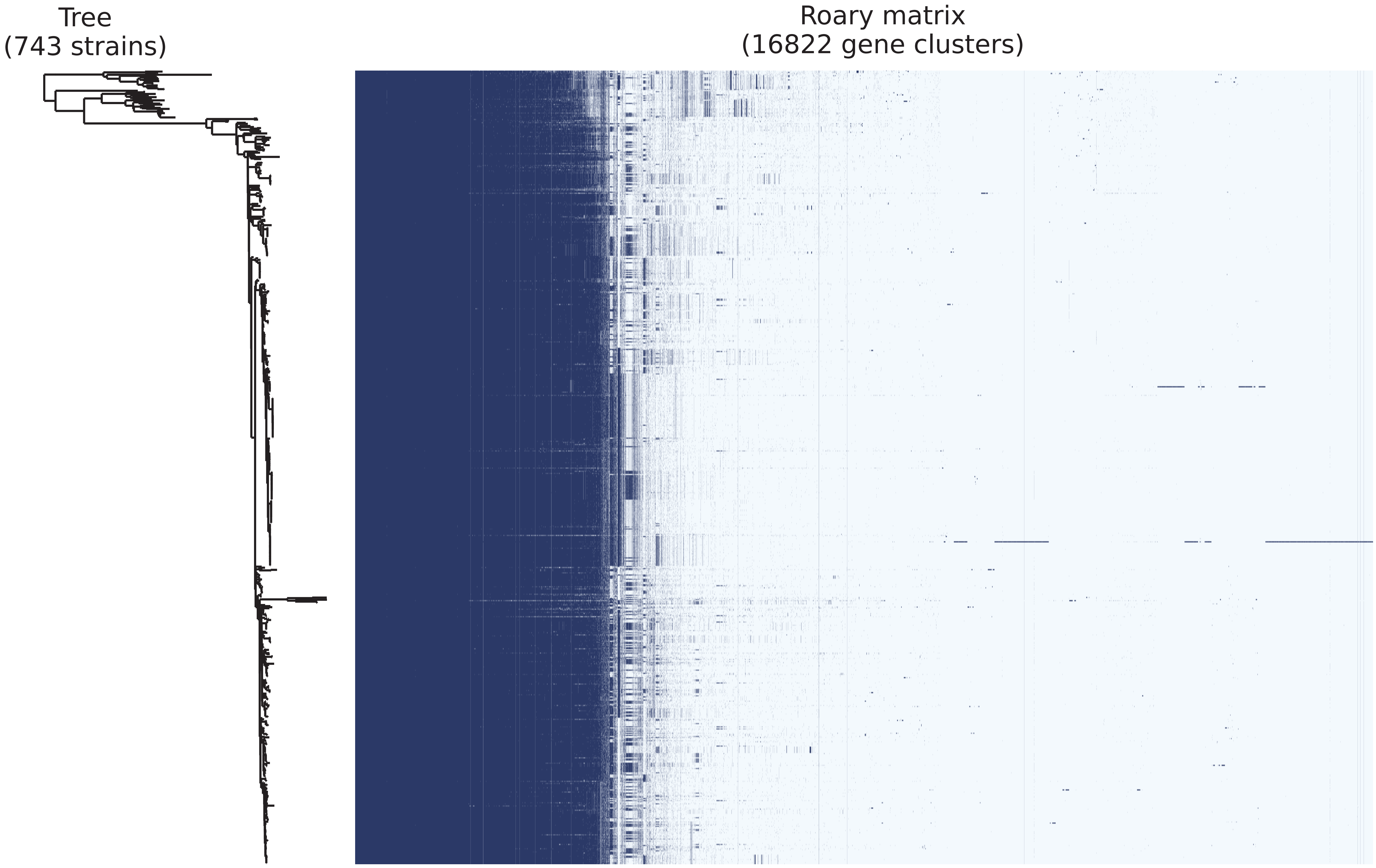

Tree
(743 strains)
Roary matrix
(16822 gene clusters)

B

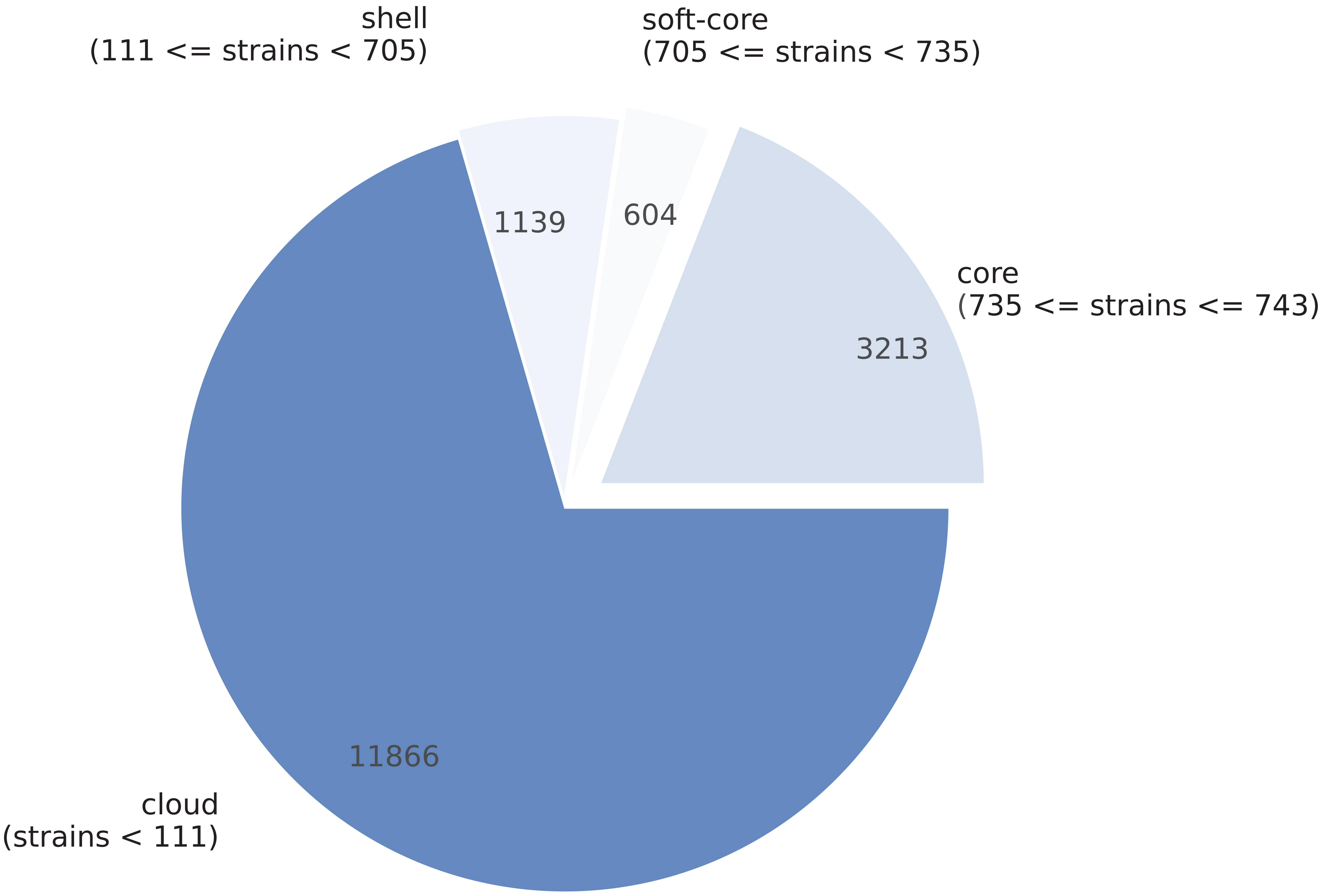

shell
(111 <= strains < 705)
soft-core
(705 <= strains < 735)
1139
604
core
(735 <= strains <= 743)
3213
11866
cloud
(strains < 111)

Table S1. Antimicrobial resistance of the Chinese *S. sonnei* isolates, including the ONPG-negative variants

Antimicrobials	Antimicrobial resistance (%)	
	All (n=587)	ONPG-negative (n=99)
**Cephalosporins**		
Ceftazidime	2.39% (14/587)	2.02% (2/99)
Ceftriaxone	75.64% (444/587)	40.40% (40/99)
Cefepime	1.19% (7/587)	2.02% (2/99)
Cefoperazone	68.14% (400/587)	37.37% (37/99)
Cefazolin	75.81% (445/587)	41.41% (41/99)
Cefoxitin	2.90% (17/587)	2.02% (2/99)
**Penicillins**		
Piperacillin	79.56% (467/587)	71.72% (71/99)
Ticarcillin	89.95% (528/587)	79.80% (79/99)
Ampicillin	89.78% (527/587)	79.80% (79/99)
**Aminoglycosides**		
Tobramycin	40.89% (240/587)	12.12% (12/99)
Gentamicin	74.79% (439/587)	55.56% (55/99)
**Carbapenems**		
Imipenem	1.02% (6/587)	1.01% (1/99)
**Nitrofurans**		
Nitrofurantoin	1.36% (8/587)	2.02% (2/99)
**Tetracyclines**		
Tetracycline	81.77% (480/587)	73.74% (73/99)
**β-lactam/β-lactamase inhibitor combinations**		
Ticarcillin/clavulaSc acid	14.48% (85/587)	18.18% (18/99)
**Monobactams**		
Aztreonam	16.70% (98/587)	24.24% (24/99)
**Phenicols**		
Chloramphenicol	2.56% (15/587)	2.02% (2/99)
**Folate pathway inhibitors**		
Trimethoprim/sulfamethoxazole	88.59% (520/587)	84.85% (84/99)
**Macrolide**		
Azithromycin	48.21% (283/587)	17.17% (17/99)
**Colistin**		
Colistin	6.13% (36/587)	0% (0/99)
**cef^{R}azi^{R}**	47.87% (281/587)	17.17% (17/99)

Table S2. Antibiotic resistance patterns of the Chinese *S. sonnei* isolates in this study

Resistant drug class	ONPG-negative *S. sonnei* (n=99)	ONPG-positive *S. sonnei* (n=488)
No resistance detected	3 (3.03%)	12 (2.46%)
Resistance ≥ 1 CLSI class	96 (96.97%)	476 (97.54%)
Resistance ≥ 2 CLSI classes	93 (93.94%)	473 (96.93%)
Resistance ≥ 3 CLSI classes	77 (77.78%)	430 (88.11%)
Resistance ≥ 4 CLSI classes	67 (67.68%)	408 (83.61%)
Resistance ≥ 5 CLSI classes	24 (24.24%)	343 (70.29%)
Resistance ≥ 6 CLSI classes	13 (13.13%)	77 (15.78%)

Table S3. The presence of the large virulence plasmid pSs046 in 743 S. sonnei strains.

Strains	pSs046 coverage (%)
sh19sh017	100
sh19sh008	100
sh19sh024	100
sh19sh010	100
sh19sh021	100
sh19sh009	100
sh19sh004	100
sh19sh019	100
sh19sh026	100
sh19sh014	100
sh19sh015	100
sh19sh003	100
sh19sh022	100
sh19sh012	100
sh19sh027	100
sh19sh020	100
sh19sh018	100
sh19sh005	100
sh19sh023	100
sh19sh025	100
sh19sh006	100
sh19sh007	100
sh19sh011	100
sh19sh002	100
sh19sh013	100
sh19sh016	100
sh16sh275	97
sh16sh292	97
sh16sh269	0
sh16sh265	0
sh16sh294	0
sh16sh283	0
sh16sh267	0
sh16sh262	0
sh16sh285	0
sh16sh281	0
sh16sh273	0
sh16sh300	0
sh16sh280	0
sh16sh277	0
sh16sh297	0
sh16sh290	0
sh16sh286	0

sh16sh287	0
sh16sh266	0
sh16sh289	0
sh16sh295	0
sh16sh278	0
sh16sh288	0
sh16sh274	0
sh16sh268	0
sh16sh284	0
sh16sh291	0
sh16sh276	0
sh16sh302	0
sh16sh296	0
sh16sh299	0
sh16sh272	0
sh16sh587	0
sh16sh004	0
sh16sh279	0
sh16sh588	0
sh16sh576	0
sh15sh102	0
sh16sh574	0
sh16sh581	0
sh16sh578	0
sh16sh298	0
sh15sh100	0
sh15sh99	0
sh15sh104	0
sh16sh270	0
sh15sh98	0
sh16sh264	0
sh16sh001	0
sh16sh580	0
sh16sh005	0
sh16sh573	0
sh15sh106	0
sh15sh103	0
sh16sh007	0
sh15sh105	0
sh15sh108	0
sh15sh107	0
sh16sh003	0
sh16sh008	0
sh16sh006	0
sh16sh271	0
sh16sh575	0

sh16sh293	0
sh15sh101	0
sh16sh009	0
sh16sh301	0
sh16sh577	0
sh16sh303	0
sh16sh309	100
R55	0
sh12sh252	0
sh16sh543	0
sh16sh534	0
sh16sh540	0
sh16sh308	0
sh16sh552	0
sh16sh529	0
sh16sh546	0
sh16sh550	0
sh16sh306	0
sh16sh568	0
sh16sh564	0
sh16sh549	0
R64	100
sn0815	100
R113	100
sh11sh376	100
sh10sh159	100
SRR4195740	100
41sh10D187	100
sh11sh393	100
sh10sh249	100
SRR4192106	100
ERR028685	100
R83	100
R03	100
R79	100
sn0603	100
R111	100
sh10sh112	100
sh12sh220	100
ZH061	100
sh11sh201	100
sh11sh516	100
sh10sh217	100
sh12sh100	100
HN13136	100
sh11sh386	100

sh10sh166	100
sh11sh002	100
ERR025718	100
sh12sh196	100
SRR3530549	100
R109	100
sh11sh347	100
sh10sh234	100
R19	100
R76	100
sh11sh387	100
sh12sh295	100
sh10sh176	100
R36	100
sh13sh65	100
R74	100
sh10D164	100
sh11sh483	100
sh10sh299	100
R110	100
sh10sh285	100
R73	100
sh12sh266	100
HN11128	100
HN12099	100
sh11sh105	100
sh11sh359	100
HN12202	100
sn0730	100
41sh10D162	100
SRR3531022	100
sh11sh071	100
sh11sh529	100
WH82	100
HN08160	100
R31	100
sh10sh172	100
sh10sh144	100
R95	100
R04	100
SRR3530773	100
sh10sh237	100
sh10sh273	100
R30	100
SRR3530807	100
XJ2006096	100

sh11sh306	100
R88	100
R50	100
R22	100
XJ12212	100
R105	100
sh11sh109	100
SY230422	100
R01	100
R63	100
R94	100
sh05sh574	100
sh10sh214	100
sh10sh288	100
SRR4195487	100
sh10sh001	100
20122H050	100
R101	100
sh11sh385	100
R29	100
sh11sh398	100
R25	100
SRR3531909	100
SRR3531087	100
R28	100
sh10sh187	100
sh11sh458	100
R34	100
BJ20102H032	100
230603	100
R71	100
20062H025	100
R23	100
SRR3531253	100
sh10sh297	100
R85	100
ERR025716	100
ERR028703	100
sh10sh019	100
SRR3531029	100
sh10sh286	100
sh13sh51	100
sh10sh098	100
sh13sh33	100
HN09221	100
SRR5005299	100

ERR028704	100
R12	100
sh10sh167	100
sh10sh235	100
sh10sh236	100
HN08149	100
R65	100
sh10sh296	100
sh11sh074	100
YN14	100
ERR028699	100
SRR3531866	100
SRR5006072	100
ERR025713	100
ERR025710	100
sh10sh62	100
ERR028695	100
HN13118	100
sh11sh202	100
XJ12140	100
ERR028689	100
ERR024079	100
sh13sh142	100
ERR024618	100
sh12sh010	100
SRR3531903	100
SRR3530636	100
ERR025709	100
SRR3531897	100
SRR4788222	100
SRR3530640	100
SRR4787278	100
lj10020	100
SRR3531874	99
R99	99
sh10sh266	99
ERR1541589	99
SRR3531882	99
1943	99
SRR3531231	99
ERR025705	99
ERR025762	99
ERR025691	99
ERR1364327	99
sh12sh260	99
R11	99

ERR025699	98
sh10sh012	98
SRR3530435	98
ZD15029	97
ERR025725	97
sh12sh144	97
R24	97
sn0729	97
SRR3530638	97
SRR4786326	97
ERR1364329	96
SRR5029673	96
ERR025693	95
SRR3530393	95
ERR025717	95
ERR025687	94
R37	94
sn0742	94
R41	94
R67	94
ERR025711	94
sn0717	94
sn0719	94
ERR025704	94
sn0743	94
ERR025685	94
ERR025712	94
ERR028694	94
ERR024608	93
ERR025701	93
ERR025683	93
SRR3530555	92
ERR025690	91
ERR025698	91
ERR028706	90
ERR025697	90
lj14023	0
ERR025700	0
lj06058	0
sh12sh50	0
ERR025748	0
ERR025708	0
ERR025721	0
lj12024	0
R115	0
ERR024085	0

ERR024078	0
WH52	0
R134	0
sh14sh014	0
ERR025695	0
SY230719	0
ERR028690	0
2020SXLYX	0
lj16007	0
ERR1541598	0
lj11005	0
sh10sh016	0
R27	0
R104	0
sh11sh374	0
sh10sh147	0
sh10sh108	0
sh10sh126	0
ERR025755	0
R91	0
R92	0
ERR028692	0
R93	0
ERR025765	0
ERR025754	0
R87	0
SRR3530795	0
2020SXXXj	0
R51	0
ERR028672	0
SRR5023700	0
ERR1364170	0
ERR025706	0
sn1103	0
ERR024606	0
lj13016	0
sh12D215	0
HN10208	0
sh10sh053	0
sh12sh133	0
41sh11D062	0
R129	0
R75	0
R58	0
R70	0
R127	0

R60	0
R17	0
ERR1541578	0
ERR025751	0
2020SXFB021	0
sh12D205	0
sh10sh121	0
R52	0
sn0720	0
sh10sh003	0
RY184	0
SRR3531721	0
sh11sh079	0
SRR3531887	0
SRR3531782	0
SRR4787219	0
SRR5005327	0
sh14sh038	0
sh13D178	0
2020SXFB024	0
2020SXFB044	0
2020SXFB015	0
2020SXFB029	0
2020SXFB012	0
2020SXFB025	0
2020SXFB005	0
2020SXWSZ	0
2020SXFB023	0
2020SXFB019	0
2020SXFB035	0
2020SXFB011	0
2020SXFB042	0
2020SXFB001	0
2020SXFB039	0
2020SXFB051	0
2020SXFB028	0
2020SXFB034	0
2020SXFB027	0
2020SXFB016	0
2020SXFB037	0
2020SXFB007	0
2020SXFB020	0
2020SXFB018	0
2020SXFB048	0
2020SXFB009	0
2020SXFB003	0

2020SXFB046	0
2020SXFB006	0
2020SXWbk	0
2020SXFB013	0
2020SXFB049	0
2020SXFB026	0
2020SXFB022	0
2020SXFB032	0
11sh13D152	0
WH88	0
sh12sh046	0
sh12sh051	0
sh13sh178	0
sh11sh221	0
sh14sh003	0
sh14sh004	0
HN12187	0
ERR1516071	0
R53	0
R108	0
ERR025734	0
ERR1364193	0
ERR024619	0
GIMI239	0
R57	0
sh11sh487	0
ERR025686	0
sn1016	0
ERR025749	0
ERR025682	0
ERR025719	0
HN09238	0
HN09187	0
ERR024614	0
sn1017	0
sh09sh134	0
ERR025746	0
18	0
ERR025743	0
sn0947	0
sn1004	0
sn0932	0
41sh13D174	0
R72	0
AH17	0
sh12sh216	0

sh13sh037	0
sh16sh589	0
sh11sh210	0
HN12237	0
R77	0
R61	0
R124	0
R131	0
R06	0
R47	0
sh10sh122	0
sh10sh111	0
sh13sh61	0
HN13068	0
BJ20072H007	0
sh11sh277	0
R32	0
sh11sh417	0
sh10sh173	0
1977	0
sh11sh244	0
R56	0
HN10108	0
BJ2010074	0
HN10159	0
R123	0
R128	0
R106	0
R14	0
R119	0
R45	0
R120	0
R35	0
R132	0
R48	0
R90	0
R121	0
R135	0
R126	0
R78	0
R09	0
R96	0
R02	0
R07	0
R100	0
R122	0

sh13sh47	0
lj07026	0
sh12sh123	0
sh236	0
X8	0
sh11sh179	0
sh11sh241	0
sh12sh212	0
sh08sh242	0
20110812H	0
sn1104	0
sh11sh133	0
sh12sh30	0
sn1201	0
sh188	0
WH13	0
sh11sh78	0
sh11sh108	0
sh11sh220	0
sh12sh45	0
sh11sh209	0
sh10sh145	0
sh12sh256	0
sh11sh293	0
sh13sh152	0
sn0801	0
sh13sh002	0
ZD16030	0
ERR028679	0
sh10sh256	0
sh10sh229	0
sh10sh304	0
R38	0
ERR1364242	0
sh12sh191	0
sh11sh270	0
sh11sh272	0
sh11sh297	0
sh11sh324	0
sh11sh301	0
sh11sh459	0
sh13sh147	0
sh13sh134	0
lj07024	0
ERR025767	0
R82	0

sh12sh197	0
ERR025732	0
sh19sh028	0
sh19sh048	0
sh19sh046	0
sh11sh353	0
sh19sh036	0
sh19sh033	0
sh19sh034	0
sh19sh030	0
sh19sh035	0
sh19sh047	0
sh19sh039	0
sh19sh049	0
sh10sh294	0
sh19sh031	0
sh19sh038	0
sh19sh041	0
sh13sh135	0
sh19sh029	0
sh19sh043	0
sh19sh037	0
sh19sh045	0
sh19sh042	0
sh14sh043	0
sh19sh040	0
sh19sh032	0
sh09sh171	0
sh10sh274	0
sh19sh044	0
sh16sh255	0
sh14sh045	0
sh16sh506	0
sh16sh258	0
ERR024605	0
R66	0
R80	0
ERR1364207	0
ERR1364191	0
ERR1364277	0
ERR1364227	0
ERR1364216	0
ERR1364257	0
ERR1364276	0
ERR024620	0
R103	0

20112H151	0
ERR024625	0
ERR1364263	0
lj06057	0
sh16sh336	0
lj16011	0
ERR024626	0
41sh10D181	0
sh08sh072	0
ERR1364293	0
ERR028675	0
sh12sh200	0
sh12sh177	0
sh12sh059	0
sh11sh125	0
WH7	0
XJ2010010	0
HN08184	0
sh12sh284	0
sh11sh488	0
sh10sh253	0
sh11sh290	0
lj11023	0
BJ20092H102	0
sh11sh211	0
lj11031	0
sh08sh212	0
sn0707	0
sn0713	0
sn0741	0
sn0714	0
sn0722	0
sn0723	0
sn0724	0
sn0716	0
lj11042	0
ERR1364253	0
R20	0
sh11sh225	0
sn0701	0
sh12sh190	0
ERR024616	0
ERR024617	0
R62	0
R138	0
R125	0

R08	0
R26	0
R42	0
R49	0
R43	0
R44	0
R107	0
R13	0
R10	0
ERR1364206	0
ERR1364234	0
sh12sh55	0
R116	0
ERR1364192	0
sh11sh103	0
R117	0
R137	0
HN6	0
sh13sh180	0
sh12sh112	0
sh12sh169	0
sh11sh492	0
R114	0
R84	0
ERR025731	0
R21	0
R16	0
R98	0
R15	0
R18	0
R54	0
R136	0
R102	0
ERR025737	0
ERR025761	0
ERR025726	0
WH25	0
sh11sh041	0
sh12sh217	0
sh11sh418	0
lj13015	0
lj16015	0
ERR025729	0
ERR025742	0
ERR028688	0
ERR028691	0

sh12sh113	0
sh13sh069	0
R69	0
R133	0
R40	0
ERR025758	0
ERR028676	0
ERR024076	0
ERR025703	0
BJ20122H075	0
sh10sh151	0
sh19sh001	0
lj06041	0
XJ2012013	0
HN1	0
R112	0
R33	0
ERR025714	0
ERR024607	0
sh13sh150	0
R118	0
ERR024611	0
ERR024610	0
21679	0
ERR024622	0
ERR024627	0
R89	0
21535	0
R68	0
R86	0
R97	0
R39	0
R59	0
R05	0
R81	0
sh12sh257	0
ERR024609	0
R130	0
ERR024612	0
ERR024604	0
ERR025715	0
sh10sh2	0
sh12sh215	0
230608	0
sh12sh213	0
ERR028705	0

sn0806	0
ERR025747	0
XJ2014176	0
ERR025768	0
ERR025750	0
ERR025756	0
ERR025724	0
ERR025692	0
ERR025753	0
sh05sh293	0

Table S4. Accessory genes associated with ONPG-negative variants

Gene	Presence in ONPG-negative strains	Presence in other strains	Absence in ONPG-negative strains	Absence in other strains	chiseq	*P* value
cnu	13(13.13%)	624(96.89%)	86(86.87%)	20(3.11%)	485.40769	1.42E-107
group_7407	0(0%)	632(98.14%)	99(100%)	12(1.86%)	642.62792	8.96E-142
lacI	0(0%)	631(97.98%)	99(100%)	13(2.02%)	635.87039	2.64E-140
lacY	0(0%)	626(97.2%)	99(100%)	18(2.8%)	603.81546	2.48E-133
lacZ	0(0%)	613(95.19%)	99(100%)	31(4.81%)	532.01244	1.03E-117
mhpA	0(0%)	632(98.14%)	99(100%)	12(1.86%)	642.62792	8.96E-142
mhpB	0(0%)	632(98.14%)	99(100%)	12(1.86%)	642.62792	8.96E-142
mhpR	0(0%)	632(98.15%)	99(100%)	12(1.87%)	642.62792	8.96E-142
yiaJ	0(0%)	632(98.14%)	99(100%)	12(1.86%)	642.62792	8.96E-142

Table S5. SNPs significantly associated with the MCC II clade

CHR	BP	Mutation	Type	Amino acid	Gene	Product	*P* (chi-seq, adj by fdr)	COG code
CP000038	2248896	T=>C	initiator codon variant	p.Met1-	gatY	tagatose-bisphosphate aldolase 1	5.81E-61	G
CP000038	819559	T=>C	intergenic				4.41E-64	
CP000038	1959401	G=>A	intergenic				3.24E-107	
CP000038	2248927	T=>C	intergenic				7.93E-62	
CP000038	3851577	A=>G	intergenic				3.24E-107	
CP000038	747483	A=>C	intergenic				4.55E-60	
CP000038	148125	C=>T	missense variant	p.Asp693Asn	gcd	glucose dehydrogenase	3.70E-101	G
CP000038	193167	C=>T	missense variant	p.Ala44Thr	pfs	conserved hypothetical protein	7.93E-62	F
CP000038	230238	T=>C	missense variant	p.Thr21Ala	yaeO	conserved hypothetical protein	4.41E-64	K
CP000038	391136	C=>T	missense variant	p.Ala107Val	aroM	protein of aro operon regulated by aroR	5.85E-103	
CP000038	562166	C=>T	missense variant	p.Arg1046Trp	ybdE	putative inner membrane component for iron transpo	3.22E-62	P
CP000038	726622	C=>A	missense variant	p.Ala44Asp	hrsA	protein modification enzyme induction of ompC	6.55E-89	G
CP000038	727336	T=>C	missense variant	p.Ile282Thr	hrsA	protein modification enzyme	1.06E-54	G
CP000038	1376659	C=>G	missense variant	p.Val254Leu	purT	phosphoribosylglycinamide formyltransferase 2	2.75E-77	F
CP000038	1527239	C=>T	missense variant	p.Arg225Cys	SSON_1453	conserved hypothetical protein	1.61E-109	S
CP000038	2056107	A=>C	missense variant	p.Phe232Val	narK	nitrite extrusion protein	3.27E-65	P
CP000038	2056926	A=>G	missense variant	p.Val63Ala	conserved	hypothetical protein	3.24E-107	
CP000038	2169250	T=>C	missense variant	p.Val133Ala	pduS	putative propanediol utilization protein:ferredoxin	4.43E-72	C
CP000038	2465186	GGG=>GCG	missense variant	p.Val286Gly	nuoL	NADH dehydrogenase I chain L	1.34E-63	CP
CP000038	2488116	G=>A	missense variant	p.Met22Ile	yfcC	putative S-transferase	1.01E-271	S
CP000038	2492128	T=>A	missense variant	p.Trp173Arg	yfcG	putative S-transferase	3.24E-107	O
CP000038	2704351	C=>A	missense variant	p.Leu161Ile	hyfB	hydrogenase 4 membrane subunit	4.41E-64	CP
CP000038	3083989	G=>A	missense variant	p.Pro247Ser	ygcA	putative enzyme	4.90E-106	J
CP000038	3317052	A=>G	missense variant	p.Ile141Thr	ygiRQ	Fe-S oxidoreductase	4.41E-64	C
CP000038	3371683	G=>C	missense variant	p.Thr160Arg	yqjH	conserved hypothetical protein	3.70E-101	P
CP000038	3689916	A=>G	missense variant	p.Arg268Gly	yrfF	putative dehydrogenase	1.01E-272	
CP000038	3881911	G=>A	missense variant	p.Asp84Asn	yhhF	conserved hypothetical protein	5.81E-61	L
CP000038	3938553	G=>A	missense variant	p.Ala40Thr	gltS	glutamate transport	1.34E-63	E
CP000038	3952595	G=>A	missense variant	p.Thr20Ile	ttk	putative transcriptional regulator	4.90E-106	K
CP000038	4097937	C=>T	missense variant	p.Ala250Val	glmU	N-acetyl glucosamine-1-phosphate uridyltransferase	7.93E-62	M
CP000038	4237501	C=>T	missense variant	p.Ser145Phe	yigP	conserved hypothetical protein	1.06E-54	S
CP000038	4306242	T=>C	missense variant	p.Ser128Gly	frvB	PTS system fructose-like enzyme IIBC component	1.01E-272	G
CP000038	4366788	T=>A	missense variant	p.Leu4Gln	frwD	PTS system fructose-like IIB component 2	4.43E-72	G
CP000038	4438780	A=>G	missense variant	p.Ser120Pro	iclR	repressor of aceBA operon	4.41E-64	K
CP000038	4620189	T=>G	missense variant	p.Ile243Leu	yjeS	conserved hypothetical protein	1.34E-63	C
CP000038	4655680	C=>T	missense variant	p.Asp31Asn	ytfB	conserved hypothetical protein	1.34E-63	M
CP000038	196757	G=>A	synonymous variant	p.Gln318Gln	htrA	periplasmic serine protease Do	1.76E-120	O
CP000038	480529	C=>T	synonymous variant	p.Gly600Gly	aefA	putative alpha helix protein	5.81E-61	M
CP000038	561422	C=>T	synonymous variant	p.Leu798Leu	ybdE	putative inner membrane component for iron transpo	1.34E-63	P
CP000038	667248	C=>T	synonymous variant	p.Leu142Leu	nagE	PTS system N-acetylglucosamine-specific enzyme I	3.24E-107	G
CP000038	824594	A=>C	synonymous variant	p.Gly159Gly	ybiB	putative enzyme	4.41E-64	E
CP000038	844182	C=>T	synonymous variant	p.Ile124Ile	ybiR	conserved hypothetical protein	4.41E-64	P
CP000038	858857	G=>A	synonymous variant	p.Leu46Leu	mipB	putative transaldolase	3.35E-270	G
CP000038	947726	T=>C	synonymous variant	p.His228His	ftsK	cell division protein	4.41E-64	D
CP000038	1029124	C=>T	synonymous variant	p.Cys356Cys	uup	putative ATP-binding component of a transport syst	5.00E-267	R
CP000038	1207876	C=>T	synonymous variant	p.Leu165Leu	ndh	respiratory NADH dehydrogenase	4.43E-72	C
CP000038	1213343	C=>T	synonymous variant	p.Leu644Leu	mfd	transcription-repair coupling factor	4.41E-64	LK
CP000038	1302728	C=>T	synonymous variant	p.Leu72Leu	cheA	sensory transducer kinase between chemo-signal rec	7.93E-62	NT
CP000038	1466776	G=>A	synonymous variant	p.Leu66Leu	topB	DNA topoisomerase III	7.93E-62	L

CP000038	1605976	G=>A	synonymous variant	p.Phe152Phe	SSON_1529	putative membrane protein	6.99E-90	C
CP000038	1956810	G=>A	synonymous variant	p.Ala24Ala	yciH	conserved hypothetical protein	7.93E-62	J
CP000038	2061352	G=>A	synonymous variant	p.Leu65Leu	ychN	conserved hypothetical protein	7.93E-62	P
CP000038	2300159	A=>G	synonymous variant	p.Gly15Gly	yohF	putative oxidoreductase	1.06E-54	IQR
CP000038	2373264	A=>G	synonymous variant	p.Leu26Leu	napB	cytochrome c-type protein	1.06E-54	C
CP000038	2639389	A=>G	synonymous variant	p.Val146Val	zipA	cell division protein	4.41E-64	D
CP000038	2800875	G=>A	synonymous variant	p.Val321Val	yfhD	putative periplasmic binding transport protein	5.81E-61	M
CP000038	3055920	A=>T	synonymous variant	p.Pro6Pro	cysI	sulfite reductase alpha subunit	1.06E-54	P
CP000038	3207169	G=>A	synonymous variant	p.Thr248Thr	recJ	ssDNA exonuclease	6.99E-90	L
CP000038	3232775	C=>T	synonymous variant	p.Phe577Phe	sbm	methylmalonyl-CoA mutase (MCM)	6.99E-90	I
CP000038	3713878	C=>T	synonymous variant	p.Ala661Ala	malP	maltodextrin phosphorylase	7.93E-62	G
CP000038	3718138	G=>A	synonymous variant	p.Gln556Gln	malT	positive regulator of mal regulon	5.81E-61	K
CP000038	3851359	G=>T	synonymous variant	p.Arg20Arg	asd	aspartate-semialdehyde dehydrogenase	6.99E-90	E
CP000038	4226472	A=>G	synonymous variant	p.Ser145Ser	metR	regulator for metE and metH	1.06E-54	K
CP000038	4624346	A=>G	synonymous variant	p.Glu29Glu	mutL	enzyme in methyl-directed mismatch repair	5.85E-103	L
CP000038	4634057	C=>T	synonymous variant	p.Gly199Gly	vacB	putative enzyme	3.24E-107	K
CP000038	4666728	G=>A	synonymous variant	p.Lys58Lys	ytfK	conserved hypothetical protein	4.90E-106	

Table S6. The SNPs detected significantly associated with ONPG-negative variants

CHR	BP	Mutation	Type	Amino acid	Gene	Product	*P* (chi-seq, adj by fdr)	COG
CP000038	2248896	T=>C	initiator codon variant	Met1-	gatY	tagatose-bisphosphate aldolase 1	5.24E-217	G
CP000038	193167	C=>T	missense variant	Ala44Thr	pfs	conserved hypothetical protein	3.99E-220	F
CP000038	2056107	A=>C	missense variant	Phe232Val	narK	nitrite extrusion protein	4.83E-207	P
CP000038	3881911	G=>A	missense variant	Asp84Asn	yhhF	conserved hypothetical protein	5.24E-217	L
CP000038	4097937	C=>T	missense variant	Ala250Val	glmU	N-acetyl glucosamine-1-phosphate uridyltransferase	3.99E-220	M
CP000038	3424710	G=>T	missense_variant	Gln134Lys		putative minor pilin and initiator	1.86E-130	
CP000038	4289050	G=>C	missense_variant	Trp124Cys	yihX	putative phosphatase	7.99E-108	
CP000038	4694244	C=>T	missense_variant	Thr56Met	mgtA	Mg2+ transport ATPase, P-type 1	4.21E-72	P
CP000038	1218335	A=>G	missense_variant	Glu131Gly	ycfV	putative ATP-binding component of a transport system	2.09E-69	
CP000038	1420433	T=>G	missense_variant	Ser169Arg		conserved hypothetical protein	4.94E-56	
CP000038	2802779	A=>T	missense_variant	Gln40Leu	yfhH	conserved hypothetical protein	4.94E-56	
CP000038	2248927	T=>C	intergenic				3.99E-220	
CP000038	747483	A=>C	intergenic				6.74E-214	
CP000038	4699034	G=>A	intergenic				1.08E-58	
CP000038	480529	C=>T	synonymous variant	Gly600Gly	aefA	putative alpha helix protein	5.24E-217	
CP000038	1302728	C=>T	synonymous variant	Leu72Leu	cheA	sensory transducer kinase between chemo-signal receptors and CheB and CheY	3.99E-220	
CP000038	1466776	G=>A	synonymous variant	Leu66Leu	topB	DNA topoisomerase III	3.99E-220	
CP000038	1956810	G=>A	synonymous variant	Ala24Ala	yciH	conserved hypothetical protein	3.99E-220	
CP000038	2061352	G=>A	synonymous variant	Leu65Leu	ychN	conserved hypothetical protein	3.99E-220	
CP000038	2800875	G=>A	synonymous variant	Val321Val	yfhD	putative periplasmic binding transport protein	5.24E-217	M
CP000038	3713878	C=>T	synonymous variant	Ala661Ala	malP	maltodextrin phosphorylase	3.99E-220	G
CP000038	3718138	G=>A	synonymous variant	Gln556Gln	malT	positive regulator of mal regulon	5.24E-217	K
CP000038	196757	G=>A	synonymous_variant	Gln318Gln	htrA	periplasmic serine protease Do	1.03E-103	O

Table S7. Accessory genes associated with MCC II and other branches

Group	Presence in other strains	Absence in other strains	Presence in MCC II strains	Absence in MCC II strains	chiseq	p value
group_1061	18(3.88%)	446(96.12%)	264(94.62%)	15(5.38%)	605.40908	1.115E-133
group_7701	0(0%)	464(100%)	243(87.1%)	36(12.9%)	596.58492	9.26E-132
group_386	17(3.66%)	447(96.34%)	238(85.3%)	41(14.7%)	511.57311	2.8837E-113
cea	52(11.21%)	412(88.79%)	250(89.61%)	29(10.39%)	440.65582	7.79317E-98
group_6221	55(11.85%)	409(88.15%)	251(89.96%)	28(10.04%)	435.64459	9.60202E-97
group_461	51(10.99%)	413(89.01%)	244(87.46%)	35(12.54%)	422.33489	7.57239E-94
group_7069	406(87.5%)	58(12.5%)	28(10.04%)	251(89.96%)	427.20929	6.58098E-95
tnsE	395(85.13%)	69(14.87%)	41(14.7%)	238(85.3%)	353.59355	6.99205E-79
group_1059	382(82.33%)	82(17.67%)	36(12.9%)	243(87.1%)	338.4366	1.39751E-75

Table S8. Metadata of the *Shigella sonnei* strains used in this study

Accession	Strains	Clade	ONPG phenotype	cefRaziR	Region	Date	Ceftazidime	Ceftriaxone	Imipenem	Strofurantoin	Piperacillin	Tetracycline	Cefepime	Cefoperazone	Cefazolin	Cefoxitin	Tobramycin	Levofloxacin	Gentamicin
SRR19126997	ZH061	Main Chinese clade I	Positive	cefRaziR	Beijing	2011	S	R	S	S	I	R	S	R	R	S	R	S	R
SRR22671215	18	Main Chinese clade II	Positive	cefRaziR	Anhui	2014	I	R	S	S	R	R	S	R	R	S	I	S	R
SRR22671216	1943	Main Chinese clade II	Negative	non-cefRaziR	Beijing	2005	R	R	R	R	R	R	R	S	R	R	R	S	R
SRR22671217	1977	Main Chinese clade II	Positive	cefRaziR	Beijing	2005	S	R	R	R	I	R	S	S	R	R	R	S	R
SRR19126766	21535	other strains in Lineage III	Positive	non-cefRaziR	China	/	S	S	S	S	S	R	S	S	S	S	S	S	S
SRR19126767	21679	Lineage II	Positive	non-cefRaziR	China	/	S	S	S	S	S	S	S	S	S	S	S	S	S
SRR19126768	230603	Main Chinese clade I	Negative	non-cefRaziR	Liaoning	2006	S	S	S	S	R	R	S	S	S	S	S	S	S
SRR19126769	230608	Main Chinese clade I	Negative	non-cefRaziR	Liaoning	2006	S	S	S	S	S	R	S	S	S	S	S	S	S
SRR19126770	11sh13D152	Main Chinese clade I	Positive	cefRaziR	Henan	2013	I	R	S	S	I	R	S	S	R	I	I	S	R
SRR22671218	20062H025	Main Chinese clade II	Negative	non-cefRaziR	Beijing	2006	S	S	S	S	S	S	S	S	S	S	R	S	R
SRR22671219	20110812H	Main Chinese clade II	Negative	non-cefRaziR	Beijing	2011	S	S	S	S	R	R	S	S	S	S	I	S	R
SRR19126771	20112H151	Main Chinese clade I	Positive	non-cefRaziR	Beijing	2011	S	R	S	S	R	R	S	R	R	S	R	S	R
SRR22671220	20122H050	Main Chinese clade II	Negative	non-cefRaziR	Beijing	2012	I	R	S	S	R	S	S	R	R	S	S	S	S
SRR19126772	2020SXFB001	Main Chinese clade I	Positive	cefRaziR	Anhui	2020	S	R	S	S	R	R	S	R	R	S	R	S	R
SRR19126775	2020SXFB003	Main Chinese clade I	Positive	cefRaziR	Anhui	2020	S	R	S	S	R	R	S	R	R	S	R	S	R
SRR19126776	2020SXFB005	Main Chinese clade I	Positive	cefRaziR	Anhui	2020	S	R	S	S	R	R	S	R	R	S	R	S	R
SRR19126777	2020SXFB006	Main Chinese clade I	Positive	cefRaziR	Anhui	2020	S	R	S	S	R	R	S	R	R	S	R	S	R
SRR19126778	2020SXFB007	Main Chinese clade I	Positive	cefRaziR	Anhui	2020	S	R	S	S	R	R	S	R	R	S	R	S	R
SRR19126779	2020SXFB009	Main Chinese clade I	Positive	cefRaziR	Anhui	2020	S	R	S	S	R	R	S	R	R	S	R	S	R
SRR19126780	2020SXFB011	Main Chinese clade I	Positive	cefRaziR	Anhui	2020	S	R	S	S	R	R	S	R	R	S	R	S	R
SRR19126781	2020SXFB012	Main Chinese clade I	Positive	cefRaziR	Anhui	2020	S	R	S	S	R	R	S	R	R	S	R	S	R
SRR19126890	2020SXFB013	Main Chinese clade I	Positive	cefRaziR	Anhui	2020	S	R	S	S	R	R	S	R	R	S	R	S	R
SRR19126891	2020SXFB015	Main Chinese clade I	Positive	cefRaziR	Anhui	2020	S	R	S	S	R	R	S	R	R	S	R	S	R
SRR19126892	2020SXFB016	Main Chinese clade I	Positive	cefRaziR	Anhui	2020	S	R	S	S	R	R	S	R	R	S	R	S	R
SRR19126894	2020SXFB018	Main Chinese clade I	Positive	cefRaziR	Anhui	2020	S	R	S	S	R	R	S	R	R	S	R	S	R
SRR19126895	2020SXFB019	Main Chinese clade I	Positive	cefRaziR	Anhui	2020	S	R	S	S	R	R	S	R	R	S	R	S	R
SRR19126896	2020SXFB020	Main Chinese clade I	Positive	cefRaziR	Anhui	2020	S	R	S	S	R	R	S	R	R	S	R	S	R
SRR19126897	2020SXFB021	Main Chinese clade I	Positive	cefRaziR	Anhui	2020	S	R	S	S	R	R	S	R	R	S	R	S	R
SRR19126898	2020SXFB022	Main Chinese clade I	Positive	cefRaziR	Anhui	2020	S	R	S	S	R	R	S	R	R	S	R	S	R
SRR19126899	2020SXFB023	Main Chinese clade I	Positive	cefRaziR	Anhui	2020	S	R	S	S	R	R	S	R	R	S	R	S	R
SRR19126900	2020SXFB024	Main Chinese clade I	Positive	cefRaziR	Anhui	2020	S	R	S	S	R	R	S	R	R	S	R	S	R
SRR19126901	2020SXFB025	Main Chinese clade I	Positive	cefRaziR	Anhui	2020	S	R	S	S	R	R	S	R	R	S	R	S	R
SRR19126902	2020SXFB026	Main Chinese clade I	Positive	cefRaziR	Anhui	2020	S	R	S	S	R	R	S	R	R	S	R	S	R
SRR19126903	2020SXFB027	Main Chinese clade I	Positive	cefRaziR	Anhui	2020	S	R	S	S	R	R	S	R	R	S	R	S	R
SRR19126905	2020SXFB028	Main Chinese clade I	Positive	cefRaziR	Anhui	2020	S	R	S	S	R	R	S	R	R	S	R	S	R
SRR19126906	2020SXFB029	Main Chinese clade I	Positive	cefRaziR	Anhui	2020	S	R	S	S	R	R	S	R	R	S	R	S	R
SRR19126907	2020SXFB032	Main Chinese clade I	Positive	cefRaziR	Anhui	2020	S	R	S	S	R	R	S	R	R	S	R	S	R
SRR19126908	2020SXFB034	Main Chinese clade I	Positive	cefRaziR	Anhui	2020	S	R	S	S	R	R	S	R	R	S	R	S	R
SRR19126909	2020SXFB035	Main Chinese clade I	Positive	cefRaziR	Anhui	2020	S	R	S	S	R	R	S	R	R	S	R	S	R
SRR19126910	2020SXFB037	Main Chinese clade I	Positive	cefRaziR	Anhui	2020	S	R	S	S	R	R	S	R	R	S	R	S	R
SRR19126911	2020SXFB039	Main Chinese clade I	Positive	cefRaziR	Anhui	2020	S	R	S	S	R	R	S	R	R	S	R	S	R
SRR19126912	2020SXFB042	Main Chinese clade I	Positive	cefRaziR	Anhui	2020	S	R	S	S	R	R	S	R	R	S	R	S	R
SRR19126913	2020SXFB044	Main Chinese clade I	Positive	cefRaziR	Anhui	2020	S	R	S	S	R	R	S	R	R	S	R	S	R
SRR19126914	2020SXFB046	Main Chinese clade I	Positive	cefRaziR	Anhui	2020	S	R	S	S	R	R	S	R	R	S	R	S	R
SRR19126916	2020SXFB048	Main Chinese clade I	Positive	cefRaziR	Anhui	2020	S	R	S	S	R	R	S	R	R	S	R	S	R
SRR19126917	2020SXFB049	Main Chinese clade I	Positive	cefRaziR	Anhui	2020	S	R	S	S	R	R	S	R	R	S	R	S	R
SRR19126918	2020SXFB051	Main Chinese clade I	Positive	cefRaziR	Anhui	2020	S	R	S	S	R	R	S	R	R	S	R	S	R
SRR19126919	2020SXLYX	Main Chinese clade I	Positive	cefRaziR	Anhui	2020	S	R	S	S	R	R	S	R	R	S	R	S	R
SRR19126920	2020SXWbk	Main Chinese clade I	Positive	cefRaziR	Anhui	2020	S	R	S	S	R	R	S	R	R	S	R	S	R
SRR19126921	2020SXWSZ	Main Chinese clade I	Positive	cefRaziR	Anhui	2020	S	R	S	S	R	R	S	R	R	S	R	S	R
SRR19126922	2020SXXXj	Main Chinese clade I	Positive	cefRaziR	Anhui	2020	S	R	S	S	R	R	S	R	R	S	R	S	R

SRR22671221	41sh10D162	Main Chinese clade II	Positive	cefRaziR	Henan	2010	S	R	S	S	R	R	S	I	R	S	R	S	R
SRR22671222	41sh10D181	Main Chinese clade II	Positive	cefRaziR	Henan	2010	I	R	S	S	R	S	S	R	R	S	I	S	R
SRR22671223	41sh10D187	Main Chinese clade II	Positive	non-cefRaziR	Henan	2010	I	R	R	S	R	R	S	R	R	S	R	S	R
SRR22671224	41sh11D062	Main Chinese clade II	Positive	cefRaziR	Henan	2011	S	R	S	S	R	R	S	R	R	S	I	S	R
SRR19126923	41sh13D174	Main Chinese clade I	Positive	non-cefRaziR	Henan	2013	I	R	S	S	R	R	S	R	R	S	S	S	S
SRR22671226	AH17	Main Chinese clade II	Positive	non-cefRaziR	Anhui	2014	S	R	S	S	I	R	S	I	R	S	I	S	R
SRR22671227	BJ20072H007	Main Chinese clade II	Positive	non-cefRaziR	Beijing	2007	S	S	S	S	S	R	S	S	S	S	I	S	R
SRR19126924	BJ20092H102	Main Chinese clade I	Positive	non-cefRaziR	Beijing	2009	S	R	S	S	R	R	S	R	R	S	I	S	R
SRR22671228	BJ2010074	Main Chinese clade II	Negative	non-cefRaziR	Beijing	2011	S	S	S	S	R	R	S	S	S	S	I	S	R
SRR22671229	BJ20102H032	Main Chinese clade II	Positive	non-cefRaziR	Beijing	2010	S	S	S	S	S	R	S	S	S	S	S	S	R
SRR22671230	BJ20122H075	Main Chinese clade II	Positive	non-cefRaziR	Beijing	2012	S	S	S	S	S	S	S	S	S	S	S	S	S
SRR22671023	GIMI239	Main Chinese clade I	Positive	non-cefRaziR	Shandong	/	S	S	S	S	S	S	S	S	S	S	S	S	S
SRR22671231	HN08149	Main Chinese clade II	Positive	non-cefRaziR	Henan	2008	S	S	S	S	S	R	S	S	S	S	I	S	R
SRR22671232	HN08160	Main Chinese clade II	Positive	non-cefRaziR	Henan	2008	R	R	S	S	R	R	S	R	R	S	S	S	R
SRR19126925	HN08184	Main Chinese clade I	Positive	non-cefRaziR	Henan	2008	S	R	S	S	R	R	S	R	R	S	S	S	S
SRR19126819	HN09187	Main Chinese clade I	Negative	non-cefRaziR	Henan	2009	S	S	S	S	S	R	S	S	S	S	S	S	S
SRR22671233	HN09221	Main Chinese clade II	Positive	non-cefRaziR	Henan	2009	S	S	S	S	R	R	S	S	S	S	I	S	R
SRR19126820	HN09238	Main Chinese clade I	Negative	non-cefRaziR	Henan	2009	S	S	S	S	S	R	S	S	S	S	S	S	S
SRR22671234	HN1	Main Chinese clade II	Positive	non-cefRaziR	Henan	1999	S	S	S	S	S	R	S	S	S	S	S	S	S
SRR22671235	HN10108	Main Chinese clade II	Negative	non-cefRaziR	Henan	2010	S	S	S	S	R	R	S	S	S	S	I	S	R
SRR22671237	HN10159	Main Chinese clade II	Positive	non-cefRaziR	Henan	2010	S	R	S	S	R	S	S	R	R	S	I	S	R
SRR22671097	HN10208	Main Chinese clade II	Positive	non-cefRaziR	Henan	2010	S	R	S	S	R	R	S	R	R	S	R	S	R
SRR22671099	HN11128	Main Chinese clade II	Positive	non-cefRaziR	Henan	2011	I	R	S	S	R	R	S	R	R	S	I	S	R
SRR22671100	HN12099	Main Chinese clade II	Negative	non-cefRaziR	Henan	2012	I	R	S	S	R	R	S	R	R	S	I	S	R
SRR19126821	HN12187	Main Chinese clade I	Positive	non-cefRaziR	Henan	2012	I	R	S	S	R	R	S	S	R	R	I	S	R
SRR22671101	HN12202	Main Chinese clade II	Positive	non-cefRaziR	Henan	2012	S	R	S	S	R	S	S	R	R	S	I	S	R
SRR22671102	HN12237	Main Chinese clade II	Negative	non-cefRaziR	Henan	2012	I	R	S	S	R	R	S	R	R	S	I	S	R
SRR22671103	HN13068	Main Chinese clade II	Positive	non-cefRaziR	Henan	2013	S	R	S	S	R	R	S	R	R	S	I	S	R
SRR22671104	HN13118	Main Chinese clade II	Positive	non-cefRaziR	Henan	2013	S	S	S	S	R	R	S	S	S	S	I	S	R
SRR22671105	HN13136	Main Chinese clade II	Negative	non-cefRaziR	Henan	2013	S	S	S	S	S	R	S	S	S	S	S	S	R
SRR22671106	HN6	Main Chinese clade II	Positive	non-cefRaziR	Henan	2003	S	S	S	S	S	R	S	S	S	S	S	S	S
SRR22671108	lj06041	Main Chinese clade II	Positive	non-cefRaziR	Shanxi	2006	S	S	S	S	S	R	S	S	S	S	S	S	S
SRR22671109	lj06057	Main Chinese clade II	Positive	non-cefRaziR	Shanxi	2006	S	R	S	S	I	R	S	R	R	S	S	S	S
SRR22671110	lj06058	Main Chinese clade II	Positive	non-cefRaziR	Shanxi	2006	S	R	S	S	I	R	S	R	R	S	S	S	S
SRR19126822	lj07024	Main Chinese clade I	Positive	non-cefRaziR	Shanxi	2007	S	R	S	S	R	R	S	R	R	S	I	S	R
SRR22671111	lj07026	Main Chinese clade II	Positive	non-cefRaziR	Shanxi	2007	S	R	S	S	R	R	S	R	R	S	I	S	R
SRR22671112	lj10020	Main Chinese clade II	Positive	non-cefRaziR	Shanxi	2010	S	R	S	S	I	R	S	I	R	S	S	S	R
SRR22671113	lj11005	Main Chinese clade II	Positive	non-cefRaziR	Shanxi	2011	I	R	S	S	R	R	S	R	R	S	R	S	R
SRR19126823	lj11023	Main Chinese clade I	Positive	non-cefRaziR	Shanxi	2011	S	R	S	S	I	R	S	I	R	S	I	S	R
SRR19126824	lj11031	Main Chinese clade I	Positive	non-cefRaziR	Shanxi	2011	S	R	S	S	I	R	S	I	R	S	I	S	R
SRR19126825	lj11042	Main Chinese clade I	Positive	non-cefRaziR	Shanxi	2011	S	R	S	S	I	R	S	I	R	S	I	S	R
SRR22671114	lj12024	Main Chinese clade II	Positive	non-cefRaziR	Shanxi	2012	S	S	S	S	S	R	S	S	S	S	S	S	R
SRR19126826	lj13015	Main Chinese clade I	Positive	non-cefRaziR	Shanxi	2013	I	R	S	S	R	R	S	R	R	S	S	S	R
SRR22671115	lj13016	Main Chinese clade II	Positive	non-cefRaziR	Shanxi	2013	I	R	S	S	R	R	S	R	R	S	S	S	R
SRR19126827	lj14023	Main Chinese clade I	Positive	non-cefRaziR	Shanxi	2014	I	R	S	S	R	R	S	R	R	S	S	S	R
SRR19126828	lj16007	Main Chinese clade I	Positive	non-cefRaziR	Shanxi	2016	S	R	S	S	I	R	S	S	R	S	R	S	R
SRR19126830	lj16011	Main Chinese clade I	Positive	non-cefRaziR	Shanxi	2016	S	R	S	S	I	R	S	I	R	S	R	S	R
SRR19126831	lj16015	Main Chinese clade I	Positive	non-cefRaziR	Shanxi	2016	S	R	S	S	I	S	S	I	R	S	S	I	R
SRR22671116	R01	Main Chinese clade II	Positive	non-cefRaziR	Henan	2006	S	S	S	S	S	R	S	S	S	S	S	S	S
SRR22671117	R02	Main Chinese clade II	Negative	non-cefRaziR	Henan	2011	S	S	S	S	R	R	S	S	S	S	I	S	R
SRR22671119	R03	Main Chinese clade II	Positive	non-cefRaziR	Henan	2010	S	S	S	S	R	R	S	S	S	S	I	S	R
SRR22671120	R04	Main Chinese clade II	Positive	non-cefRaziR	Henan	2011	S	S	S	S	S	R	S	S	S	S	I	S	R
SRR22671121	R05	Main Chinese clade II	Positive	non-cefRaziR	Henan	2004	S	S	S	S	S	R	S	S	S	S	S	S	S
SRR22671122	R06	Main Chinese clade II	Positive	non-cefRaziR	Henan	2010	I	R	S	S	R	R	S	R	R	S	R	S	R

SRR22671123	R07	Main Chinese clade II	Negative	non-cefRaziR	Henan	2010	S	S	S	S	R	R	S	S	S	S	I	S	R
SRR19126832	R08	Main Chinese clade I	Positive	non-cefRaziR	Henan	2008	S	R	S	S	R	R	S	R	S	S	S	S	S
SRR22671124	R09	Main Chinese clade II	Positive	non-cefRaziR	Henan	2012	S	S	S	S	R	R	S	S	S	S	I	S	R
SRR19126833	R10	other strains in Lineage III	Positive	non-cefRaziR	Henan	2009	S	R	S	S	R	R	S	R	R	S	I	S	R
SRR22671125	R100	Main Chinese clade II	Positive	non-cefRaziR	Hubei	2007	S	S	S	S	R	R	S	S	S	S	I	S	R
SRR22671126	R101	Main Chinese clade II	Positive	non-cefRaziR	Hubei	2010	S	S	S	S	R	R	S	S	S	S	I	S	R
SRR22671127	R102	Main Chinese clade II	Positive	non-cefRaziR	Hubei	2012	S	R	S	S	R	S	S	R	R	S	S	S	S
SRR19126834	R103	Main Chinese clade I	Positive	non-cefRaziR	Hubei	2013	S	R	S	S	R	R	S	R	R	S	I	S	R
SRR19126835	R104	Lineage I	Positive	non-cefRaziR	Hubei	2006	S	R	S	S	R	S	S	R	R	S	S	S	S
SRR22671128	R105	Main Chinese clade II	Positive	non-cefRaziR	Hubei	2007	I	R	S	S	R	R	S	R	R	S	S	S	S
SRR22671130	R106	Main Chinese clade II	Positive	non-cefRaziR	Hubei	2008	S	S	S	S	R	R	S	S	S	S	I	S	R
SRR19126836	R107	Main Chinese clade I	Positive	non-cefRaziR	Hubei	2009	S	R	S	S	R	R	S	R	R	S	I	S	R
SRR19126837	R108	Main Chinese clade I	Positive	non-cefRaziR	Hubei	2010	R	R	S	S	R	R	S	S	R	R	I	S	R
SRR22671131	R109	Main Chinese clade II	Positive	non-cefRaziR	Hubei	2010	R	R	S	S	R	S	S	R	R	S	S	S	S
SRR22671132	R11	Main Chinese clade II	Positive	non-cefRaziR	Gansu	2009	S	S	S	S	S	R	S	S	R	S	S	S	S
SRR22671133	R110	Main Chinese clade II	Positive	non-cefRaziR	Hubei	2010	I	R	S	S	R	S	S	R	R	S	S	S	S
SRR22670994	R111	Main Chinese clade II	Positive	non-cefRaziR	Xinjiang	2006	S	S	S	S	S	S	S	S	S	S	S	S	S
SRR19126838	R112	Main Chinese clade III	Positive	non-cefRaziR	Xinjiang	2009	S	S	S	S	S	R	S	S	S	S	S	S	S
SRR22670995	R113	Main Chinese clade II	Positive	non-cefRaziR	Xinjiang	2010	S	S	S	S	S	R	S	S	S	S	S	S	S
SRR19126839	R114	Main Chinese clade III	Positive	non-cefRaziR	Xinjiang	2012	I	R	S	S	R	R	S	R	R	S	S	S	S
SRR19126841	R115	Main Chinese clade III	Positive	non-cefRaziR	Xinjiang	2012	I	R	S	S	R	R	S	R	R	S	S	S	S
SRR19126842	R116	Main Chinese clade III	Positive	non-cefRaziR	Xinjiang	2012	I	R	S	S	R	R	S	R	R	S	S	S	S
SRR19126843	R117	Main Chinese clade III	Positive	non-cefRaziR	Xinjiang	2012	I	R	S	S	R	R	S	R	R	S	S	S	S
SRR19126844	R118	Main Chinese clade III	Positive	non-cefRaziR	Xinjiang	2012	S	S	S	S	S	R	S	S	S	S	S	S	S
SRR22670996	R119	Main Chinese clade II	Positive	non-cefRaziR	Yunnan	2012	S	S	S	S	R	R	S	S	S	S	I	S	R
SRR22670997	R12	Main Chinese clade II	Positive	non-cefRaziR	Henan	2011	S	R	S	S	R	S	S	R	R	S	I	S	R
SRR22670998	R120	Main Chinese clade II	Positive	non-cefRaziR	Yunnan	2013	S	S	S	S	R	R	S	S	S	S	I	S	R
SRR22670999	R121	Main Chinese clade II	Positive	non-cefRaziR	Yunnan	2012	S	S	S	S	R	R	S	S	S	S	I	S	R
SRR22671001	R122	Main Chinese clade II	Positive	non-cefRaziR	Yunnan	2009	I	R	S	S	R	R	S	R	R	S	I	S	R
SRR22671002	R123	Main Chinese clade II	Positive	non-cefRaziR	Yunnan	2012	S	S	S	S	R	R	S	S	S	S	S	S	R
SRR22671003	R124	Main Chinese clade II	Positive	non-cefRaziR	Liaoning	2011	R	R	R	R	R	R	S	R	R	R	R	S	R
SRR22671004	R125	Main Chinese clade II	Positive	non-cefRaziR	Xinjiang	2010	S	R	S	S	R	R	S	R	R	S	S	S	S
SRR22671005	R126	Main Chinese clade II	Positive	non-cefRaziR	Yunnan	2013	S	S	S	S	R	R	S	S	S	S	I	S	R
SRR22671006	R127	Main Chinese clade II	Positive	non-cefRaziR	Guangdong	2012	R	R	S	S	R	R	S	R	R	S	I	S	R
SRR22671007	R128	Main Chinese clade II	Positive	non-cefRaziR	Shanghai	2004	S	S	S	S	R	R	S	S	S	S	I	S	R
SRR22671008	R129	Main Chinese clade II	Positive	non-cefRaziR	Shanghai	2012	S	R	S	S	R	R	S	R	R	S	R	S	R
SRR19126845	R13	other strains in Lineage III	Positive	non-cefRaziR	Gansu	2011	I	R	S	S	R	R	S	R	R	S	S	S	R
SRR22671009	R130	Main Chinese clade II	Positive	non-cefRaziR	Liaoning	2007	S	S	S	S	S	S	S	S	S	S	S	S	S
SRR22671010	R131	Main Chinese clade II	Positive	non-cefRaziR	Liaoning	2011	R	R	R	R	R	R	S	R	R	R	R	S	R
SRR22671012	R132	Main Chinese clade II	Positive	non-cefRaziR	Liaoning	2012	S	S	S	S	R	R	S	S	S	S	I	S	R
SRR19126846	R133	Main Chinese clade I	Positive	non-cefRaziR	Liaoning	2006	R	R	S	S	R	R	S	R	R	S	S	S	S
SRR22671013	R134	Main Chinese clade II	Positive	non-cefRaziR	Gaungxi	2007	I	R	S	S	R	R	R	R	R	S	I	S	R
SRR22671014	R135	Main Chinese clade II	Positive	non-cefRaziR	Gaungxi	2009	S	S	S	S	R	R	S	S	S	S	S	S	R
SRR22671015	R136	Main Chinese clade II	Positive	non-cefRaziR	Gaungxi	2003	S	S	S	S	S	R	S	S	S	S	S	S	S
SRR19126847	R137	Main Chinese clade III	Positive	non-cefRaziR	Xinjiang	2008	S	R	S	S	R	S	S	R	R	S	S	S	S
SRR22671016	R138	Main Chinese clade II	Positive	non-cefRaziR	Xinjiang	2010	S	R	S	S	R	R	S	R	R	S	S	S	S
SRR22671017	R14	Main Chinese clade II	Positive	non-cefRaziR	Gansu	2011	S	S	S	S	R	R	S	S	S	S	I	S	R
SRR22671018	R15	Main Chinese clade II	Positive	non-cefRaziR	Gansu	2010	I	R	S	S	R	S	S	R	R	S	S	S	S
SRR22671019	R16	Main Chinese clade II	Positive	non-cefRaziR	Beijing	2004	S	S	S	S	S	R	S	S	S	S	S	S	S
SRR22671020	R17	Main Chinese clade II	Positive	non-cefRaziR	Gansu	2006	S	S	S	S	S	R	S	S	S	S	I	S	R
SRR22671021	R18	Main Chinese clade II	Positive	non-cefRaziR	Gansu	2010	I	R	S	S	R	S	S	R	R	S	S	S	S
SRR22671024	R19	Main Chinese clade II	Positive	non-cefRaziR	Gansu	2008	S	S	S	S	S	R	S	S	S	S	S	S	S
SRR19126848	R20	Main Chinese clade IV	Positive	non-cefRaziR	Gansu	2007	S	R	S	S	R	R	S	R	R	S	I	S	R
SRR19126849	R21	Main Chinese clade I	Positive	non-cefRaziR	Gansu	2006	I	R	S	S	R	R	S	R	R	S	S	S	S

SRR22671025	R22	Main Chinese clade II	Positive	non-cefRaziR	Gansu	2007	S	S	S	S	R	R	S	S	S	S	I	S	R
SRR22671026	R23	Main Chinese clade II	Positive	non-cefRaziR	Gansu	2008	S	S	S	S	R	R	S	S	S	S	I	S	R
SRR22671027	R24	Main Chinese clade II	Negative	non-cefRaziR	Anhui	2013	S	S	S	S	R	R	S	S	S	S	I	S	R
SRR22671028	R25	Main Chinese clade II	Negative	non-cefRaziR	Anhui	2014	I	R	S	S	R	R	S	R	R	S	I	S	R
SRR19126850	R26	Main Chinese clade I	Positive	non-cefRaziR	Anhui	2010	S	R	S	S	R	R	S	R	R	R	R	S	R
SRR19126852	R27	Lineage I	Positive	non-cefRaziR	Anhui	2013	S	S	S	S	R	R	S	S	S	S	I	S	R
SRR22671030	R28	Main Chinese clade II	Positive	non-cefRaziR	Anhui	2014	I	R	S	S	R	R	S	R	R	S	I	S	R
SRR22671062	R29	Main Chinese clade II	Negative	non-cefRaziR	Beijing	2010	S	S	S	S	S	R	S	S	S	S	S	S	S
SRR22671063	R30	Main Chinese clade II	Positive	non-cefRaziR	Beijing	2004	S	S	S	S	S	S	S	S	S	S	S	S	S
SRR19126853	R31	Main Chinese clade I	Positive	non-cefRaziR	Beijing	2005	I	R	S	S	R	R	S	R	R	S	S	S	S
SRR22671064	R32	Main Chinese clade II	Positive	non-cefRaziR	Beijing	2005	S	R	S	S	R	R	S	R	R	S	I	S	R
SRR19126782	R33	Main Chinese clade IV	Positive	non-cefRaziR	Beijing	2005	S	S	S	S	S	R	S	S	S	S	S	S	S
SRR19126783	R34	Lineage I	Positive	non-cefRaziR	Beijing	2006	S	R	S	S	R	S	S	R	R	S	S	S	S
SRR22671065	R35	Main Chinese clade II	Positive	non-cefRaziR	Beijing	2006	S	S	S	S	R	R	S	S	S	S	I	S	R
SRR22671067	R36	Main Chinese clade II	Positive	non-cefRaziR	Beijing	2007	S	S	S	S	R	R	S	S	S	S	I	S	R
SRR22671068	R37	Main Chinese clade II	Positive	non-cefRaziR	Beijing	2007	I	R	S	S	R	R	S	R	R	S	R	S	R
SRR19126784	R38	Main Chinese clade I	Positive	non-cefRaziR	Beijing	2007	I	R	S	S	R	R	S	R	R	S	S	S	S
SRR19126785	R39	Main Chinese clade I	Positive	non-cefRaziR	Beijing	2008	S	S	S	S	S	R	S	S	S	S	S	S	S
SRR19126786	R40	Main Chinese clade I	Positive	non-cefRaziR	Beijing	2008	I	R	S	S	R	R	S	R	R	S	S	S	S
SRR19126787	R41	Main Chinese clade I	Positive	non-cefRaziR	Beijing	2008	I	R	S	S	R	R	S	R	R	S	S	S	S
SRR19126788	R42	Main Chinese clade I	Positive	non-cefRaziR	Beijing	2009	S	S	S	S	R	R	S	S	S	S	I	S	R
SRR19126789	R43	Main Chinese clade I	Positive	non-cefRaziR	Beijing	2009	S	R	S	S	R	R	S	R	R	S	I	S	R
SRR19126791	R44	Main Chinese clade I	Positive	non-cefRaziR	Beijing	2010	S	R	S	S	R	R	S	R	R	S	I	S	R
SRR22671069	R45	Main Chinese clade II	Positive	non-cefRaziR	Beijing	2010	S	S	S	S	R	R	S	S	S	S	I	S	R
SRR22671070	R47	Main Chinese clade II	Positive	non-cefRaziR	Beijing	2012	I	R	S	S	R	R	S	R	R	S	I	S	R
SRR22671071	R48	Main Chinese clade II	Positive	non-cefRaziR	Beijing	2012	S	S	S	S	R	R	S	S	S	S	I	S	R
SRR19126792	R49	Main Chinese clade I	Positive	non-cefRaziR	Beijing	2012	S	R	S	S	R	R	S	R	R	S	I	S	R
SRR19126793	R50	Main Chinese clade I	Negative	non-cefRaziR	Gansu	2009	I	R	S	S	R	R	S	R	R	S	S	S	S
SRR19126794	R51	Main Chinese clade I	Negative	non-cefRaziR	Gansu	2010	S	R	S	S	R	R	S	R	R	S	S	S	S
SRR19126795	R52	Main Chinese clade III	Positive	non-cefRaziR	Gansu	2009	S	R	S	S	R	R	S	R	R	S	R	S	R
SRR19126796	R53	Main Chinese clade III	Positive	non-cefRaziR	Gansu	2009	S	R	S	S	R	R	S	R	R	S	R	S	R
SRR22671072	R54	Main Chinese clade II	Positive	non-cefRaziR	Gaungxi	2004	S	S	S	S	R	R	S	S	S	S	I	S	R
SRR19126797	R55	Lineage I	Positive	non-cefRaziR	Gaungxi	2007	S	S	S	S	S	R	S	S	S	S	S	S	S
SRR22671073	R56	Main Chinese clade II	Positive	non-cefRaziR	Gaungxi	2009	S	S	S	S	R	R	S	S	S	S	I	S	R
SRR22671074	R57	Main Chinese clade II	Positive	non-cefRaziR	Gaungxi	2003	S	R	S	S	R	R	S	R	R	S	S	S	S
SRR22671075	R58	Main Chinese clade II	Positive	non-cefRaziR	Gaungxi	2007	S	R	S	S	R	R	S	R	R	S	S	S	R
SRR22671076	R59	Main Chinese clade II	Positive	non-cefRaziR	Gaungxi	2012	S	S	S	S	S	S	S	S	S	S	S	S	S
SRR22671078	R60	Main Chinese clade II	Positive	non-cefRaziR	Guangdong	2012	S	S	S	S	R	R	S	S	S	S	I	S	R
SRR22671079	R61	Main Chinese clade II	Positive	non-cefRaziR	Henan	2010	I	R	S	S	R	R	S	R	R	S	R	S	R
SRR22671080	R62	Main Chinese clade II	Negative	non-cefRaziR	Shanghai	2012	I	R	S	S	R	S	S	R	R	S	S	S	S
SRR22671081	R63	Main Chinese clade II	Negative	non-cefRaziR	Shanghai	2012	I	R	S	S	R	S	S	R	R	S	S	S	S
SRR22671082	R64	Main Chinese clade II	Negative	non-cefRaziR	Shanghai	2011	S	R	S	S	R	R	S	R	R	S	I	S	R
SRR22671083	R65	Main Chinese clade II	Negative	non-cefRaziR	Shanghai	2011	I	R	S	S	R	R	S	R	R	S	I	S	R
SRR22671084	R66	Main Chinese clade II	Negative	non-cefRaziR	Shanghai	2012	I	R	S	S	R	S	S	R	R	S	S	S	S
SRR22671085	R67	Main Chinese clade II	Positive	non-cefRaziR	Shanghai	2005	I	R	S	S	R	R	S	R	R	S	S	S	R
SRR22671086	R68	Main Chinese clade II	Positive	non-cefRaziR	Shanghai	2006	S	R	S	S	R	S	S	R	R	S	S	S	S
SRR19126798	R69	Main Chinese clade I	Positive	non-cefRaziR	Shanghai	2007	I	R	S	S	R	R	S	R	R	S	S	S	S
SRR22671087	R70	Main Chinese clade II	Positive	non-cefRaziR	Shanghai	2008	I	R	S	S	R	R	R	R	R	S	I	S	R
SRR22671089	R71	Main Chinese clade II	Positive	non-cefRaziR	Shanghai	2008	R	R	R	R	R	S	R	R	R	R	R	S	R
SRR22671090	R72	Main Chinese clade II	Positive	non-cefRaziR	Shanghai	2008	I	R	S	S	R	R	R	R	R	S	I	S	R
SRR22671091	R73	Main Chinese clade II	Positive	non-cefRaziR	Shanghai	2009	I	R	S	S	R	R	S	R	R	S	I	S	R
SRR22671092	R74	Main Chinese clade II	Positive	non-cefRaziR	Shanghai	2010	I	R	S	S	R	R	R	R	R	S	S	S	R
SRR22671093	R75	Main Chinese clade II	Positive	non-cefRaziR	Shanghai	2010	S	R	S	S	R	R	S	R	R	S	S	S	R
SRR22671094	R76	Main Chinese clade II	Positive	non-cefRaziR	Shanghai	2010	I	R	S	S	R	R	S	R	R	S	I	S	R

SRR22671095	R77	Main Chinese clade II	Positive	non-cefRaziR	Shanghai	2011	I	R	S	S	R	R	S	R	R	S	I	S	R
SRR22671096	R78	Main Chinese clade II	Positive	non-cefRaziR	Shanghai	2011	S	S	S	S	R	R	S	S	S	S	I	S	R
SRR22671098	R79	Main Chinese clade II	Positive	non-cefRaziR	Shanghai	2011	I	R	S	S	R	R	S	R	R	S	I	S	R
SRR22671134	R80	Main Chinese clade II	Positive	non-cefRaziR	Shanghai	2011	I	R	S	S	R	S	S	R	R	S	R	S	R
SRR22671136	R81	Main Chinese clade II	Positive	non-cefRaziR	Shanghai	2002	S	S	S	S	S	S	S	S	S	S	S	S	S
SRR22671137	R82	Main Chinese clade II	Positive	non-cefRaziR	Shanghai	2013	S	R	S	S	R	S	S	R	R	S	R	S	R
SRR19126799	R83	Main Chinese clade IV	Positive	non-cefRaziR	Liaoning	2004	S	S	S	S	S	R	S	S	S	S	S	S	S
SRR19126800	R84	Main Chinese clade IV	Positive	non-cefRaziR	Liaoning	2004	S	S	S	S	S	R	S	S	S	S	S	S	S
SRR22671138	R85	Main Chinese clade II	Positive	non-cefRaziR	Liaoning	2005	S	R	S	S	R	R	S	R	R	S	I	S	R
SRR19126802	R86	Main Chinese clade I	Positive	non-cefRaziR	Liaoning	2006	S	S	S	S	S	R	S	S	S	S	S	S	S
SRR22671139	R87	Main Chinese clade II	Positive	non-cefRaziR	Liaoning	2006	S	R	S	S	R	R	S	R	R	S	R	S	R
SRR22671140	R88	Main Chinese clade II	Positive	non-cefRaziR	Liaoning	2011	S	R	S	R	R	R	S	R	R	R	R	S	R
SRR19126803	R89	Main Chinese clade I	Positive	non-cefRaziR	Liaoning	2007	S	R	S	R	S	R	S	S	R	R	R	S	R
SRR22671141	R90	Main Chinese clade II	Positive	non-cefRaziR	Liaoning	2007	S	S	S	S	R	R	S	S	S	S	S	S	R
SRR19126804	R91	Lineage I	Positive	non-cefRaziR	Sichuan	2013	R	R	S	S	R	S	S	R	R	S	S	S	S
SRR19126805	R92	Lineage I	Positive	non-cefRaziR	Sichuan	2013	S	R	S	S	R	S	S	R	R	S	S	S	S
SRR19126806	R93	Lineage I	Positive	non-cefRaziR	Sichuan	2013	S	S	S	S	S	S	S	S	S	S	S	S	S
SRR22671142	R94	Main Chinese clade II	Positive	non-cefRaziR	Hubei	2011	I	R	S	S	R	R	S	R	R	S	I	S	R
SRR22671143	R95	Main Chinese clade II	Positive	non-cefRaziR	Hubei	2011	R	R	S	S	R	R	S	R	R	S	R	S	R
SRR22671144	R96	Main Chinese clade II	Positive	non-cefRaziR	Hubei	2011	S	S	S	S	R	R	S	S	S	S	I	S	R
SRR22671145	R97	Main Chinese clade II	Positive	non-cefRaziR	Hubei	2013	S	S	S	S	S	S	S	S	S	S	S	S	S
SRR19126807	R98	Main Chinese clade I	Positive	non-cefRaziR	Hubei	2013	I	R	S	S	R	R	S	R	R	S	S	S	S
SRR22671147	R99	Main Chinese clade II	Positive	non-cefRaziR	Hubei	2006	S	S	S	S	R	R	S	S	S	S	I	S	R
SRR19126808	RY184	Main Chinese clade I	Positive	non-cefRaziR	Anhui	2014	I	R	S	S	R	R	S	R	R	S	I	S	R
SRR22671148	sh05sh293	Main Chinese clade II	Positive	non-cefRaziR	Shanghai	2005	S	R	S	S	R	S	S	R	R	S	S	S	S
SRR22671149	sh05sh574	Main Chinese clade II	Positive	cefRaziR	Shanghai	2005	S	R	S	S	R	R	S	R	R	S	R	S	R
SRR22671150	sh08sh072	Main Chinese clade II	Positive	non-cefRaziR	Shanghai	2008	I	R	S	S	R	S	S	R	R	S	S	S	S
SRR22671151	sh08sh212	Main Chinese clade II	Positive	cefRaziR	Shanghai	2008	S	R	S	S	R	S	S	R	R	S	S	S	S
SRR22671152	sh08sh242	Main Chinese clade II	Negative	non-cefRaziR	Shanghai	2008	S	S	S	S	R	R	S	S	S	S	I	S	R
SRR19126809	sh09sh134	Main Chinese clade I	Negative	non-cefRaziR	Shanghai	2009	S	S	S	S	S	R	S	S	S	S	S	S	S
SRR22671153	sh09sh171	Main Chinese clade II	Positive	cefRaziR	Shanghai	2009	S	R	S	S	R	S	S	R	R	S	S	S	S
SRR22671154	sh10D164	Main Chinese clade II	Positive	cefRaziR	Henan	2010	S	R	S	S	R	R	S	I	R	S	R	S	R
SRR22671155	sh10sh001	Main Chinese clade II	Positive	cefRaziR	Shanghai	2010	S	R	S	S	R	R	S	R	R	S	I	S	R
SRR22671156	sh10sh003	Main Chinese clade II	Positive	cefRaziR	Shanghai	2010	S	R	S	S	R	S	S	R	R	S	S	S	S
SRR22671158	sh10sh012	Main Chinese clade II	Positive	cefRaziR	Shanghai	2010	S	R	S	S	R	R	S	I	R	S	I	S	R
SRR22671159	sh10sh016	Main Chinese clade II	Positive	non-cefRaziR	Shanghai	2010	S	S	S	S	S	S	S	S	S	S	S	S	S
SRR22671160	sh10sh019	Main Chinese clade II	Positive	cefRaziR	Shanghai	2010	S	R	S	S	R	R	S	R	R	S	S	S	R
SRR22671161	sh10sh053	Main Chinese clade II	Positive	cefRaziR	Henan	2010	S	R	S	S	R	R	S	I	R	S	R	S	R
SRR22671162	sh10sh098	Main Chinese clade II	Positive	non-cefRaziR	Shanghai	2010	R	R	S	S	R	S	I	R	R	S	I	S	R
SRR19126810	sh10sh108	Lineage I	Positive	cefRaziR	Shanghai	2010	I	R	S	S	R	S	I	R	R	S	S	S	S
SRR22671163	sh10sh111	Main Chinese clade II	Negative	non-cefRaziR	Shanghai	2010	I	R	S	S	R	R	S	R	R	S	R	S	R
SRR22671164	sh10sh112	Main Chinese clade II	Positive	non-cefRaziR	Shanghai	2010	I	R	S	S	R	R	I	R	R	S	I	S	R
SRR22671165	sh10sh121	Main Chinese clade II	Positive	cefRaziR	Shanghai	2010	S	R	S	S	R	S	S	R	R	S	S	S	S
SRR22671166	sh10sh122	Main Chinese clade II	Negative	non-cefRaziR	Shanghai	2010	S	S	S	S	R	R	S	S	S	S	S	S	R
SRR19126811	sh10sh126	Lineage I	Positive	cefRaziR	Shanghai	2010	S	R	S	S	I	S	S	I	R	S	S	S	S
SRR22671167	sh10sh144	Main Chinese clade II	Negative	cefRaziR	Shanghai	2010	I	R	S	S	R	R	S	R	R	S	R	S	R
SRR22671169	sh10sh145	Main Chinese clade II	Negative	non-cefRaziR	Shanghai	2010	S	S	S	S	S	R	S	S	S	S	I	S	R
SRR19126814	sh10sh147	Lineage I	Positive	cefRaziR	Shanghai	2010	S	R	S	S	R	S	S	R	R	S	S	S	S
SRR22671170	sh10sh151	Main Chinese clade II	Positive	cefRaziR	Shanghai	2010	S	R	S	S	R	S	S	R	R	S	S	S	S
SRR22671171	sh10sh159	Main Chinese clade II	Positive	cefRaziR	Shanghai	2010	S	R	S	S	R	R	S	R	R	S	R	S	R
SRR22671172	sh10sh166	Main Chinese clade II	Positive	cefRaziR	Shanghai	2010	S	R	S	S	R	S	S	R	R	S	S	S	S
SRR22671173	sh10sh167	Main Chinese clade II	Positive	non-cefRaziR	Shanghai	2010	I	R	S	S	R	R	S	R	R	S	I	S	R
SRR22671174	sh10sh172	Main Chinese clade II	Positive	cefRaziR	Shanghai	2010	S	R	S	S	R	S	S	R	R	S	S	S	S
SRR22671175	sh10sh173	Main Chinese clade II	Positive	cefRaziR	Shanghai	2010	S	R	S	S	I	R	S	I	R	S	S	S	R

SRR19126815	sh10sh176	Main Chinese clade I	Positive	cefRaziR	Shanghai	2010	S	R	S	S	R	R	S	R	R	S	I	S	R
SRR22671176	sh10sh187	Main Chinese clade II	Positive	cefRaziR	Shanghai	2010	I	R	S	S	R	R	I	R	R	S	I	S	R
SRR22671177	sh10sh2	Main Chinese clade II	Negative	non-cefRaziR	Shanghai	2010	S	S	S	S	S	S	S	S	S	S	S	S	S
SRR22671178	sh10sh214	Main Chinese clade II	Positive	cefRaziR	Shanghai	2010	S	R	S	S	R	R	S	R	R	S	I	S	R
SRR19126816	sh10sh217	Main Chinese clade I	Positive	cefRaziR	Shanghai	2010	S	R	S	S	R	R	S	R	R	S	R	S	R
SRR19126817	sh10sh229	Main Chinese clade I	Positive	cefRaziR	Shanghai	2010	S	R	S	S	R	S	S	R	R	S	S	S	S
SRR22671180	sh10sh234	Main Chinese clade II	Positive	cefRaziR	Shanghai	2010	S	R	S	S	R	S	S	R	R	S	S	S	S
SRR22671181	sh10sh235	Main Chinese clade II	Positive	cefRaziR	Shanghai	2010	S	R	S	S	I	S	S	R	R	S	S	S	S
SRR22671182	sh10sh236	Main Chinese clade II	Positive	cefRaziR	Shanghai	2010	S	R	S	S	R	S	S	R	R	S	S	S	S
SRR22671183	sh10sh237	Main Chinese clade II	Positive	cefRaziR	Shanghai	2010	S	R	S	S	R	R	S	R	R	S	I	S	R
SRR22671184	sh10sh249	Main Chinese clade II	Positive	cefRaziR	Shanghai	2010	S	R	S	S	R	R	S	R	R	S	I	S	R
SRR19126854	sh10sh253	Main Chinese clade I	Positive	cefRaziR	Shanghai	2010	S	R	S	S	R	I	S	R	R	S	I	S	R
SRR19126855	sh10sh256	Main Chinese clade I	Positive	cefRaziR	Shanghai	2010	S	R	S	S	R	R	S	R	R	S	S	S	S
SRR19126856	sh10sh266	Main Chinese clade I	Positive	cefRaziR	Shanghai	2010	S	R	S	S	R	S	S	R	R	S	S	S	S
SRR22671185	sh10sh273	Main Chinese clade II	Positive	cefRaziR	Shanghai	2010	S	R	S	S	R	R	S	R	R	S	I	S	R
SRR19126857	sh10sh274	Main Chinese clade I	Positive	cefRaziR	Shanghai	2010	S	R	S	S	R	S	S	R	R	S	R	S	R
SRR22671186	sh10sh285	Main Chinese clade II	Positive	cefRaziR	Shanghai	2010	S	R	S	S	R	R	S	R	R	S	S	S	R
SRR22671187	sh10sh286	Main Chinese clade II	Positive	cefRaziR	Shanghai	2010	S	R	S	S	R	S	S	R	R	S	S	S	S
SRR22671188	sh10sh288	Main Chinese clade II	Positive	cefRaziR	Shanghai	2010	S	R	S	S	R	R	S	R	R	S	I	S	R
SRR19126858	sh10sh294	Main Chinese clade I	Positive	cefRaziR	Shanghai	2010	S	R	S	S	R	S	S	R	R	S	R	S	R
SRR22671189	sh10sh296	Main Chinese clade II	Positive	cefRaziR	Shanghai	2010	S	R	S	S	R	R	S	R	R	S	S	S	R
SRR22671191	sh10sh297	Main Chinese clade II	Positive	cefRaziR	Shanghai	2010	S	R	S	S	R	R	S	R	R	S	I	S	R
SRR22671192	sh10sh299	Main Chinese clade II	Positive	cefRaziR	Shanghai	2010	S	R	S	S	R	R	S	R	R	S	S	S	R
SRR19126859	sh10sh304	Main Chinese clade I	Positive	cefRaziR	Shanghai	2010	S	R	S	S	R	R	S	R	R	S	I	S	R
SRR22671193	sh10sh62	Main Chinese clade II	Positive	cefRaziR	Shanghai	2010	S	R	S	S	I	S	S	R	R	S	S	S	S
SRR22671194	sh11sh002	Main Chinese clade II	Positive	cefRaziR	Shanghai	2011	S	R	S	S	I	S	S	R	R	S	R	S	R
SRR22671195	sh11sh041	Main Chinese clade II	Negative	non-cefRaziR	Shanghai	2011	I	R	S	S	R	S	S	R	R	S	S	S	S
SRR22671196	sh11sh071	Main Chinese clade II	Negative	cefRaziR	Shanghai	2011	S	R	S	R	I	R	S	I	R	S	I	S	R
SRR22671197	sh11sh074	Main Chinese clade II	Positive	cefRaziR	Shanghai	2011	I	R	S	S	R	R	S	R	R	S	I	S	R
SRR19126861	sh11sh079	Main Chinese clade I	Positive	non-cefRaziR	Shanghai	2011	S	R	S	S	R	R	S	R	R	S	R	S	R
SRR19126862	sh11sh103	Main Chinese clade I	Positive	non-cefRaziR	Shanghai	2011	S	R	S	S	R	R	S	R	R	S	R	S	R
SRR22671198	sh11sh105	Main Chinese clade II	Negative	cefRaziR	Shanghai	2011	S	R	S	S	R	S	S	R	R	S	S	S	S
SRR22671200	sh11sh108	Main Chinese clade II	Negative	non-cefRaziR	Shanghai	2011	S	S	S	S	R	R	S	S	S	S	I	S	R
SRR22671238	sh11sh109	Main Chinese clade II	Positive	cefRaziR	Shanghai	2011	I	R	S	S	R	R	S	R	R	S	I	S	R
SRR19126863	sh11sh125	Main Chinese clade I	Positive	non-cefRaziR	Shanghai	2011	S	S	S	S	S	S	S	S	S	S	S	S	S
SRR22671199	sh11sh133	Main Chinese clade II	Negative	non-cefRaziR	Shanghai	2011	S	S	S	S	R	R	S	S	S	S	I	S	R
SRR22671201	sh11sh179	Main Chinese clade II	Negative	non-cefRaziR	Shanghai	2011	S	S	S	S	R	R	S	S	S	S	R	S	R
SRR22671202	sh11sh201	Main Chinese clade II	Negative	non-cefRaziR	Shanghai	2011	S	S	S	S	R	R	S	S	S	S	I	S	R
SRR22671203	sh11sh202	Main Chinese clade II	Negative	non-cefRaziR	Shanghai	2011	S	S	S	S	R	R	S	S	S	S	I	S	R
SRR22671029	sh11sh209	Main Chinese clade II	Negative	non-cefRaziR	Shanghai	2011	S	S	S	S	R	R	S	S	S	S	I	S	R
SRR22671031	sh11sh210	Main Chinese clade II	Negative	cefRaziR	Shanghai	2011	I	R	S	S	I	R	S	R	R	S	I	S	R
SRR19126864	sh11sh211	Main Chinese clade I	Positive	cefRaziR	Shanghai	2011	S	R	S	S	R	R	S	R	R	S	R	S	R
SRR22671032	sh11sh220	Main Chinese clade II	Negative	non-cefRaziR	Shanghai	2011	S	S	S	S	R	R	S	S	S	S	I	S	R
SRR19126865	sh11sh221	Main Chinese clade I	Positive	cefRaziR	Shanghai	2011	I	R	S	S	I	R	S	S	R	I	I	S	R
SRR19126866	sh11sh225	Main Chinese clade I	Positive	cefRaziR	Shanghai	2011	S	R	S	S	R	R	S	R	R	S	R	S	R
SRR22671033	sh11sh241	Main Chinese clade II	Negative	non-cefRaziR	Shanghai	2011	S	S	S	S	R	R	S	S	S	S	R	S	R
SRR22671034	sh11sh244	Main Chinese clade II	Positive	cefRaziR	Shanghai	2011	I	R	S	S	R	R	S	R	R	S	I	S	R
SRR22671035	sh11sh270	Main Chinese clade II	Positive	cefRaziR	Shanghai	2011	S	R	S	S	R	S	S	R	R	S	S	S	S
SRR22671037	sh11sh272	Main Chinese clade II	Positive	cefRaziR	Shanghai	2011	S	R	S	S	R	S	S	R	R	S	S	S	S
SRR22671038	sh11sh277	Main Chinese clade II	Negative	cefRaziR	Shanghai	2011	S	R	S	S	R	R	S	R	R	S	S	S	R
SRR19126867	sh11sh290	Main Chinese clade I	Positive	cefRaziR	Shanghai	2011	S	R	S	S	R	R	S	I	R	S	R	S	R
SRR22671039	sh11sh293	Main Chinese clade II	Negative	non-cefRaziR	Shanghai	2011	S	S	S	S	R	R	S	S	S	S	I	S	R
SRR22671040	sh11sh297	Main Chinese clade II	Positive	cefRaziR	Shanghai	2011	S	R	S	S	R	S	S	R	R	S	S	S	S
SRR22671041	sh11sh301	Main Chinese clade II	Positive	cefRaziR	Shanghai	2011	S	R	S	S	R	S	S	R	R	S	S	S	S

SRR22671042	sh11sh306	Main Chinese clade II	Positive	cefRaziR	Shanghai	2011	S	R	S	S	R	S	S	R	R	S	R	S	R
SRR22671043	sh11sh324	Main Chinese clade II	Positive	cefRaziR	Shanghai	2011	S	R	S	S	R	S	S	R	R	S	S	S	S
SRR22671044	sh11sh347	Main Chinese clade II	Positive	cefRaziR	Shanghai	2011	S	R	S	S	R	S	S	R	R	S	R	S	R
SRR22671045	sh11sh353	Main Chinese clade II	Positive	cefRaziR	Shanghai	2011	S	R	S	S	R	S	S	R	R	S	R	S	R
SRR22671046	sh11sh359	Main Chinese clade II	Positive	cefRaziR	Shanghai	2011	S	R	S	S	R	R	S	R	R	S	R	S	R
SRR19126868	sh11sh374	Lineage I	Positive	cefRaziR	Shanghai	2011	S	R	S	S	R	S	S	R	R	S	S	S	S
SRR22671048	sh11sh376	Main Chinese clade II	Negative	cefRaziR	Shanghai	2011	S	R	S	S	R	R	S	R	R	S	S	S	R
SRR22671049	sh11sh385	Main Chinese clade II	Positive	cefRaziR	Shanghai	2011	S	R	S	S	R	R	S	R	R	S	R	S	R
SRR22671050	sh11sh386	Main Chinese clade II	Positive	cefRaziR	Shanghai	2011	S	R	S	S	R	S	S	R	R	S	R	S	R
SRR22671051	sh11sh387	Main Chinese clade II	Positive	cefRaziR	Shanghai	2011	S	R	S	S	I	S	S	R	R	S	R	S	R
SRR22671052	sh11sh393	Main Chinese clade II	Positive	cefRaziR	Shanghai	2011	S	R	S	S	R	S	S	R	R	S	R	S	R
SRR22671053	sh11sh398	Main Chinese clade II	Positive	cefRaziR	Shanghai	2011	I	R	S	S	R	R	S	R	R	S	I	S	R
SRR22671054	sh11sh417	Main Chinese clade II	Positive	cefRaziR	Shanghai	2011	S	R	S	S	R	R	S	R	R	S	R	S	R
SRR19126869	sh11sh418	Main Chinese clade I	Positive	non-cefRaziR	Shanghai	2011	S	R	S	S	R	R	S	R	R	S	I	S	R
SRR22671055	sh11sh458	Main Chinese clade II	Positive	cefRaziR	Shanghai	2011	S	R	S	S	R	R	S	R	R	S	I	S	R
SRR22671056	sh11sh459	Main Chinese clade II	Positive	cefRaziR	Shanghai	2011	S	R	S	S	R	S	S	R	R	S	S	S	S
SRR22671057	sh11sh483	Main Chinese clade II	Negative	cefRaziR	Shanghai	2011	S	R	S	S	R	R	S	R	R	S	I	S	R
SRR19126870	sh11sh487	Main Chinese clade I	Positive	non-cefRaziR	Shanghai	2011	S	R	S	S	R	R	S	R	R	S	R	S	R
SRR19126872	sh11sh488	Main Chinese clade I	Positive	cefRaziR	Shanghai	2011	S	R	S	S	R	S	S	R	R	S	R	S	R
SRR22671059	sh11sh492	Main Chinese clade II	Negative	cefRaziR	Shanghai	2011	I	R	S	S	R	S	S	R	R	S	S	S	S
SRR22671060	sh11sh516	Main Chinese clade II	Negative	cefRaziR	Shanghai	2011	S	R	S	S	R	S	S	R	R	S	S	S	S
SRR22671061	sh11sh529	Main Chinese clade II	Positive	cefRaziR	Shanghai	2011	I	R	S	S	R	S	S	R	R	S	R	S	R
SRR22670962	sh11sh78	Main Chinese clade II	Negative	non-cefRaziR	Shanghai	2011	S	S	S	S	R	R	S	S	S	S	I	S	R
SRR19126873	sh12D205	Main Chinese clade I	Positive	cefRaziR	Henan	2012	S	R	S	S	R	R	S	R	R	S	I	S	R
SRR22670963	sh12D215	Main Chinese clade II	Positive	cefRaziR	Henan	2012	S	R	S	S	R	S	S	I	R	S	I	S	R
SRR22670964	sh12sh010	Main Chinese clade II	Negative	non-cefRaziR	Shanghai	2012	S	R	S	S	R	R	S	R	R	S	I	S	R
SRR19126874	sh12sh046	Main Chinese clade I	Positive	cefRaziR	Henan	2012	I	R	S	S	I	R	S	S	R	R	I	S	R
SRR19126875	sh12sh051	Main Chinese clade I	Positive	cefRaziR	Henan	2012	I	R	S	S	I	R	S	S	R	I	R	S	R
SRR22670965	sh12sh059	Main Chinese clade II	Negative	non-cefRaziR	Shanghai	2012	I	R	S	S	R	S	S	R	R	S	S	S	S
SRR22670966	sh12sh100	Main Chinese clade II	Negative	non-cefRaziR	Shanghai	2012	S	S	S	S	R	R	S	S	S	S	I	S	R
SRR19126876	sh12sh112	Main Chinese clade I	Positive	cefRaziR	Shanghai	2012	I	R	S	S	R	S	S	R	R	S	S	S	S
SRR22670967	sh12sh113	Main Chinese clade II	Positive	non-cefRaziR	Shanghai	2012	S	R	S	S	R	R	S	R	R	S	I	S	R
SRR22670968	sh12sh123	Main Chinese clade II	Positive	non-cefRaziR	Shanghai	2012	S	S	S	S	R	R	S	S	S	S	I	S	R
SRR22670970	sh12sh133	Main Chinese clade II	Positive	cefRaziR	Shanghai	2012	S	R	S	S	R	R	S	R	R	S	I	S	R
SRR22670971	sh12sh144	Main Chinese clade II	Negative	non-cefRaziR	Shanghai	2012	S	S	S	S	S	S	S	S	S	S	S	S	S
SRR22670972	sh12sh169	Main Chinese clade II	Positive	cefRaziR	Shanghai	2012	I	R	S	S	R	S	S	R	R	S	S	S	S
SRR22670973	sh12sh177	Main Chinese clade II	Negative	cefRaziR	Shanghai	2012	I	R	S	S	R	S	S	R	R	S	S	S	S
SRR22670974	sh12sh190	Main Chinese clade II	Negative	non-cefRaziR	Shanghai	2012	I	R	S	S	R	S	S	R	R	S	S	S	S
SRR22670975	sh12sh191	Main Chinese clade II	Negative	cefRaziR	Shanghai	2012	S	R	S	S	R	S	S	R	R	S	S	S	S
SRR22670976	sh12sh196	Main Chinese clade II	Negative	cefRaziR	Shanghai	2012	I	R	S	S	R	S	S	R	R	S	S	S	S
SRR22670977	sh12sh197	Main Chinese clade II	Negative	cefRaziR	Shanghai	2012	S	R	S	S	R	S	S	R	R	S	S	S	S
SRR22670978	sh12sh200	Main Chinese clade II	Negative	cefRaziR	Shanghai	2012	I	R	S	S	R	S	S	R	R	S	S	S	S
SRR22670979	sh12sh212	Main Chinese clade II	Negative	non-cefRaziR	Shanghai	2012	S	S	S	S	R	R	S	S	S	S	R	S	R
SRR22670981	sh12sh213	Main Chinese clade II	Negative	non-cefRaziR	Shanghai	2012	S	S	S	S	S	S	S	S	S	S	S	S	S
SRR22670982	sh12sh215	Main Chinese clade II	Negative	non-cefRaziR	Shanghai	2012	S	S	S	S	S	S	S	S	S	S	S	S	S
SRR22670983	sh12sh216	Main Chinese clade II	Positive	cefRaziR	Shanghai	2012	I	R	S	S	R	R	S	R	R	S	I	S	R
SRR22670984	sh12sh217	Main Chinese clade II	Negative	cefRaziR	Shanghai	2012	I	R	S	S	R	S	S	R	R	S	S	S	S
SRR22670985	sh12sh220	Main Chinese clade II	Negative	cefRaziR	Shanghai	2012	I	R	S	S	I	R	S	S	R	S	S	S	S
SRR19126877	sh12sh252	Main Chinese clade I	Positive	non-cefRaziR	Gaungxi	2009	S	S	S	S	I	R	S	S	S	S	R	S	R
SRR22670986	sh12sh256	Main Chinese clade II	Negative	non-cefRaziR	Gaungxi	2012	S	S	S	S	R	R	S	S	S	S	I	S	R
SRR22670987	sh12sh257	Main Chinese clade II	Positive	non-cefRaziR	Gaungxi	2012	S	S	S	S	S	S	S	S	S	S	S	S	S
SRR22670988	sh12sh260	Main Chinese clade II	Negative	non-cefRaziR	Gaungxi	2012	I	R	S	S	R	S	S	R	R	S	S	S	S
SRR22670989	sh12sh266	Main Chinese clade II	Positive	non-cefRaziR	Shanghai	2012	S	R	S	S	R	R	S	R	R	S	R	S	R
SRR19126878	sh12sh284	Main Chinese clade I	Positive	cefRaziR	Shanghai	2012	S	R	S	S	R	R	S	R	R	S	R	S	R

SRR22670990	sh12sh295	Main Chinese clade II	Positive	cefRaziR	Shanghai	2012	S	R	S	S	R	R	S	R	R	S	I	S	R
SRR22670992	sh12sh30	Main Chinese clade II	Negative	non-cefRaziR	Shanghai	2012	S	S	S	S	R	R	S	S	S	S	I	S	R
SRR22670993	sh12sh45	Main Chinese clade II	Negative	non-cefRaziR	Henan	2012	S	S	S	S	R	R	S	S	S	S	I	S	R
SRR22671204	sh12sh50	Main Chinese clade II	Negative	non-cefRaziR	Henan	2012	S	S	S	S	S	S	S	S	S	S	S	S	S
SRR22671205	sh12sh55	Main Chinese clade II	Negative	non-cefRaziR	Shanghai	2012	I	R	S	S	R	S	S	R	R	S	S	S	S
SRR19126879	sh13D178	Main Chinese clade I	Positive	cefRaziR	Henan	2013	I	R	S	S	I	R	S	S	R	I	I	S	R
SRR22671206	sh13sh002	Main Chinese clade II	Positive	non-cefRaziR	Shanghai	2004	S	S	S	S	I	R	S	S	S	S	I	S	R
SRR22671207	sh13sh037	Main Chinese clade II	Positive	cefRaziR	Shanghai	2010	I	R	S	S	R	R	S	R	R	S	I	S	R
SRR22671208	sh13sh069	Main Chinese clade II	Positive	non-cefRaziR	Shanghai	2012	S	R	S	S	R	R	S	R	R	S	R	S	R
SRR19126880	sh13sh134	Main Chinese clade I	Positive	non-cefRaziR	Shanghai	2013	R	R	S	S	R	R	I	R	R	S	R	S	R
SRR19126881	sh13sh135	Main Chinese clade I	Positive	cefRaziR	Shanghai	2013	S	R	S	S	R	R	S	R	R	S	R	S	R
SRR22671209	sh13sh142	Main Chinese clade II	Negative	non-cefRaziR	Shanghai	2013	R	R	S	S	R	R	R	R	R	S	R	S	R
SRR19126883	sh13sh147	Main Chinese clade I	Positive	cefRaziR	Shanghai	2013	S	R	S	S	R	R	S	R	R	S	R	S	R
SRR22671210	sh13sh150	Main Chinese clade II	Negative	non-cefRaziR	Shanghai	2013	S	S	S	S	S	S	S	S	S	S	S	S	S
SRR22671211	sh13sh152	Main Chinese clade II	Negative	non-cefRaziR	Shanghai	2013	S	S	S	S	R	R	S	S	S	S	R	S	R
SRR19126884	sh13sh178	Main Chinese clade I	Positive	cefRaziR	Shanghai	2013	I	R	S	S	I	R	S	S	R	R	R	S	R
SRR19126885	sh13sh180	Main Chinese clade I	Positive	cefRaziR	Shanghai	2013	I	R	S	S	R	S	S	R	R	S	S	S	S
SRR22671213	sh13sh33	Main Chinese clade II	Negative	non-cefRaziR	Shanghai	2013	S	S	S	S	S	R	S	S	S	S	I	S	R
SRR22671214	sh13sh47	Main Chinese clade II	Negative	non-cefRaziR	Shanghai	2013	S	S	S	S	R	R	S	S	S	S	S	S	R
SRR22671225	sh13sh51	Main Chinese clade II	Negative	non-cefRaziR	Shanghai	2013	S	S	S	S	R	R	S	S	S	S	R	S	R
SRR22671236	sh13sh61	Main Chinese clade II	Negative	non-cefRaziR	Shanghai	2013	I	R	S	S	R	R	S	R	R	S	I	S	R
SRR22671107	sh13sh65	Main Chinese clade II	Negative	non-cefRaziR	Shanghai	2013	S	S	S	S	R	R	S	S	S	S	S	S	R
SRR19126886	sh14sh003	Main Chinese clade I	Positive	cefRaziR	Shanghai	2014	I	R	S	S	I	R	S	S	R	R	I	S	R
SRR19126887	sh14sh004	Main Chinese clade I	Positive	cefRaziR	Shanghai	2014	I	R	S	S	I	R	S	S	R	I	I	S	R
SRR22671118	sh14sh014	Main Chinese clade II	Positive	cefRaziR	Shanghai	2014	S	R	S	S	R	R	S	R	R	S	S	S	S
SRR19126888	sh14sh038	Main Chinese clade I	Positive	cefRaziR	Shanghai	2014	I	R	S	S	I	R	S	S	R	R	I	S	R
SRR19126889	sh14sh043	Main Chinese clade I	Positive	cefRaziR	Shanghai	2014	S	R	S	S	R	R	S	R	R	S	R	S	R
SRR19126926	sh14sh045	Main Chinese clade I	Positive	non-cefRaziR	Shanghai	2014	S	R	S	S	R	R	S	R	R	S	R	S	R
SRR19126927	sh15sh100	Main Chinese clade I	Positive	cefRaziR	Gaungxi	2015	S	R	S	S	R	R	S	R	R	S	R	S	R
SRR19126928	sh15sh101	Main Chinese clade I	Positive	cefRaziR	Gaungxi	2015	S	R	S	S	R	R	S	R	R	S	R	S	R
SRR19126930	sh15sh102	Main Chinese clade I	Positive	cefRaziR	Gaungxi	2015	S	R	S	S	R	R	S	R	R	S	R	S	R
SRR19126931	sh15sh103	Main Chinese clade I	Positive	cefRaziR	Gaungxi	2015	S	R	S	S	R	R	S	R	R	S	R	S	R
SRR19126932	sh15sh104	Main Chinese clade I	Positive	cefRaziR	Gaungxi	2015	S	R	S	S	R	R	S	R	R	S	R	S	R
SRR19126933	sh15sh105	Main Chinese clade I	Positive	cefRaziR	Gaungxi	2015	S	R	S	S	R	R	S	R	R	S	R	S	R
SRR19126934	sh15sh106	Main Chinese clade I	Positive	cefRaziR	Gaungxi	2015	S	R	S	S	R	R	S	R	R	S	R	S	R
SRR19126935	sh15sh107	Main Chinese clade I	Positive	cefRaziR	Gaungxi	2015	S	R	S	S	R	R	S	R	R	S	R	S	R
SRR19126936	sh15sh108	Main Chinese clade I	Positive	cefRaziR	Gaungxi	2015	S	R	S	S	R	R	S	R	R	S	R	S	R
SRR19126937	sh15sh98	Main Chinese clade I	Positive	cefRaziR	Gaungxi	2015	S	R	S	S	R	R	S	R	R	S	R	S	R
SRR19126938	sh15sh99	Main Chinese clade I	Positive	cefRaziR	Gaungxi	2015	S	R	S	S	R	R	S	R	R	S	R	S	R
SRR19126939	sh16sh001	Main Chinese clade I	Positive	cefRaziR	Gaungxi	2016	S	R	S	S	R	R	S	R	R	S	R	S	R
SRR19126941	sh16sh003	Main Chinese clade I	Positive	cefRaziR	Gaungxi	2016	S	R	S	S	R	R	S	R	R	S	R	S	R
SRR19126942	sh16sh004	Main Chinese clade I	Positive	cefRaziR	Gaungxi	2016	S	R	S	S	R	R	S	R	R	S	R	S	R
SRR19126943	sh16sh005	Main Chinese clade I	Positive	cefRaziR	Gaungxi	2016	S	R	S	S	I	R	S	R	R	S	R	S	R
SRR19126944	sh16sh006	Main Chinese clade I	Positive	cefRaziR	Gaungxi	2016	S	R	S	S	R	R	S	R	R	S	I	S	R
SRR19126945	sh16sh007	Main Chinese clade I	Positive	cefRaziR	Gaungxi	2016	S	R	S	S	R	R	S	R	R	S	R	S	R
SRR19126946	sh16sh008	Main Chinese clade I	Positive	cefRaziR	Gaungxi	2016	S	R	S	S	R	R	S	R	R	S	R	S	R
SRR19126947	sh16sh009	Main Chinese clade I	Positive	cefRaziR	Gaungxi	2016	S	R	S	S	R	R	S	R	R	S	R	S	R
SRR19126948	sh16sh255	Main Chinese clade I	Positive	non-cefRaziR	Shanghai	2016	S	R	S	S	R	S	S	R	R	S	R	S	R
SRR19126949	sh16sh258	Main Chinese clade I	Positive	non-cefRaziR	Shanghai	2016	S	R	S	S	R	S	S	R	R	S	I	S	R
SRR19126950	sh16sh262	Main Chinese clade I	Positive	cefRaziR	Gaungxi	2016	S	R	S	S	R	R	S	R	R	S	I	S	R
SRR19126952	sh16sh264	Main Chinese clade I	Positive	cefRaziR	Gaungxi	2016	S	R	S	S	R	R	S	R	R	S	R	S	R
SRR19126953	sh16sh265	Main Chinese clade I	Positive	cefRaziR	Gaungxi	2016	S	R	S	S	R	R	S	R	R	S	R	S	R
SRR19126954	sh16sh266	Main Chinese clade I	Positive	cefRaziR	Gaungxi	2016	S	R	S	S	R	R	S	R	R	S	R	S	R
SRR19126955	sh16sh267	Main Chinese clade I	Positive	cefRaziR	Gaungxi	2016	S	R	S	S	R	R	S	R	R	S	R	S	R

SRR19126956	sh16sh268	Main Chinese clade I	Positive	cefRaziR	Gaungxi	2016	S	R	S	S	R	R	S	R	R	S	R	S	R
SRR19126957	sh16sh269	Main Chinese clade I	Positive	cefRaziR	Gaungxi	2016	S	R	S	I	R	R	S	R	R	S	R	S	R
SRR19126958	sh16sh270	Main Chinese clade I	Positive	cefRaziR	Gaungxi	2016	S	R	S	S	R	R	S	R	R	S	R	S	R
SRR19126959	sh16sh271	Main Chinese clade I	Positive	cefRaziR	Gaungxi	2016	S	R	S	S	R	R	S	R	R	S	R	S	R
SRR19126960	sh16sh272	Main Chinese clade I	Positive	cefRaziR	Gaungxi	2016	S	R	S	S	R	R	S	R	R	S	R	S	R
SRR19126961	sh16sh273	Main Chinese clade I	Positive	cefRaziR	Gaungxi	2016	S	R	S	S	R	R	S	R	R	S	R	S	R
SRR19126963	sh16sh274	Main Chinese clade I	Positive	cefRaziR	Gaungxi	2016	S	R	S	S	R	R	S	R	R	S	R	S	R
SRR19126964	sh16sh275	Main Chinese clade I	Positive	cefRaziR	Gaungxi	2016	S	R	S	S	R	R	S	R	R	S	R	S	R
SRR19126965	sh16sh276	Main Chinese clade I	Positive	cefRaziR	Gaungxi	2016	S	R	S	S	R	R	S	R	R	S	R	S	R
SRR19126966	sh16sh277	Main Chinese clade I	Positive	cefRaziR	Gaungxi	2016	S	R	S	S	R	R	S	R	R	S	R	S	R
SRR19126967	sh16sh278	Main Chinese clade I	Positive	cefRaziR	Gaungxi	2016	S	R	S	S	R	R	S	R	R	S	R	S	R
SRR19126968	sh16sh279	Main Chinese clade I	Positive	cefRaziR	Gaungxi	2016	S	R	S	S	R	R	S	R	R	S	I	S	R
SRR19126969	sh16sh280	Main Chinese clade I	Positive	cefRaziR	Gaungxi	2016	S	R	S	S	R	R	S	R	R	S	R	S	R
SRR19126970	sh16sh281	Main Chinese clade I	Positive	cefRaziR	Gaungxi	2016	S	R	S	S	R	R	S	R	R	S	I	S	R
SRR19126971	sh16sh283	Main Chinese clade I	Positive	cefRaziR	Gaungxi	2016	S	R	S	S	R	R	S	R	R	S	I	S	R
SRR19126972	sh16sh284	Main Chinese clade I	Positive	cefRaziR	Gaungxi	2016	S	R	S	S	R	R	S	R	R	S	R	S	R
SRR19126974	sh16sh285	Main Chinese clade I	Positive	cefRaziR	Gaungxi	2016	S	R	S	S	R	R	S	R	R	S	R	S	R
SRR19126975	sh16sh286	Main Chinese clade I	Positive	cefRaziR	Gaungxi	2016	S	R	S	S	R	R	S	R	R	S	R	S	R
SRR19126976	sh16sh287	Main Chinese clade I	Positive	cefRaziR	Gaungxi	2016	S	R	S	S	R	R	S	R	R	S	R	S	R
SRR19126977	sh16sh288	Main Chinese clade I	Positive	cefRaziR	Gaungxi	2016	S	R	S	S	R	R	S	R	R	S	R	S	R
SRR19126978	sh16sh289	Main Chinese clade I	Positive	cefRaziR	Gaungxi	2016	S	R	S	S	R	R	S	R	R	S	R	S	R
SRR19126979	sh16sh290	Main Chinese clade I	Positive	cefRaziR	Gaungxi	2016	S	R	S	S	R	R	S	R	R	S	R	S	R
SRR19126980	sh16sh291	Main Chinese clade I	Positive	cefRaziR	Gaungxi	2016	S	R	S	S	R	R	S	R	R	S	R	S	R
SRR19126981	sh16sh292	Main Chinese clade I	Positive	cefRaziR	Gaungxi	2016	S	R	S	S	R	R	S	R	R	S	R	S	R
SRR19126982	sh16sh293	Main Chinese clade I	Positive	cefRaziR	Gaungxi	2016	S	R	S	S	R	R	S	R	R	S	R	S	R
SRR19126983	sh16sh294	Main Chinese clade I	Positive	cefRaziR	Gaungxi	2016	S	R	S	S	R	R	S	R	R	S	R	S	R
SRR19126985	sh16sh295	Main Chinese clade I	Positive	cefRaziR	Gaungxi	2016	S	R	S	S	R	R	S	R	R	S	R	S	R
SRR19126986	sh16sh296	Main Chinese clade I	Positive	cefRaziR	Gaungxi	2016	S	R	S	S	R	R	S	R	R	R	R	S	R
SRR19126987	sh16sh297	Main Chinese clade I	Positive	cefRaziR	Gaungxi	2016	S	R	S	S	R	R	S	R	R	R	R	S	R
SRR19126988	sh16sh298	Main Chinese clade I	Positive	cefRaziR	Gaungxi	2016	S	R	S	S	R	R	S	R	R	S	R	S	R
SRR19126989	sh16sh299	Main Chinese clade I	Positive	cefRaziR	Gaungxi	2016	S	R	S	S	R	R	S	R	R	S	R	S	R
SRR19126990	sh16sh300	Main Chinese clade I	Positive	cefRaziR	Gaungxi	2016	S	R	S	S	R	R	S	R	R	S	R	S	R
SRR19126991	sh16sh301	Main Chinese clade I	Positive	cefRaziR	Gaungxi	2016	S	R	S	S	R	R	S	R	R	S	I	S	R
SRR19126992	sh16sh302	Main Chinese clade I	Positive	cefRaziR	Gaungxi	2016	S	R	S	S	R	R	S	R	R	S	R	S	R
SRR19126993	sh16sh303	Main Chinese clade I	Positive	cefRaziR	Gaungxi	2016	S	R	S	S	R	R	S	R	R	S	R	S	R
SRR19126994	sh16sh306	Main Chinese clade I	Positive	non-cefRaziR	Gaungxi	2016	S	R	S	S	R	R	S	R	R	S	R	S	R
SRR19126691	sh16sh308	Main Chinese clade I	Positive	non-cefRaziR	Gaungxi	2015	S	R	S	S	R	R	S	R	R	S	R	S	R
SRR19126692	sh16sh309	Main Chinese clade I	Positive	non-cefRaziR	Gaungxi	2015	S	R	S	S	R	R	S	R	R	S	I	S	R
SRR19126693	sh16sh336	Main Chinese clade I	Positive	non-cefRaziR	Shanghai	2016	S	R	S	S	R	R	S	R	R	S	R	S	R
SRR19126694	sh16sh506	Main Chinese clade I	Positive	non-cefRaziR	Shanghai	2016	S	R	S	S	R	R	S	R	R	S	I	S	R
SRR19126695	sh16sh529	Main Chinese clade I	Positive	non-cefRaziR	Gaungxi	2014	S	R	S	S	R	S	S	R	R	S	R	S	R
SRR19126696	sh16sh534	Main Chinese clade I	Positive	non-cefRaziR	Gaungxi	2014	S	R	S	S	I	R	S	R	R	S	R	S	R
SRR19126697	sh16sh540	Main Chinese clade I	Positive	non-cefRaziR	Gaungxi	2014	S	R	S	S	R	R	S	R	R	S	I	S	R
SRR19126698	sh16sh543	Main Chinese clade I	Positive	non-cefRaziR	Gaungxi	2014	S	R	S	S	R	R	S	R	R	S	R	S	R
SRR19126699	sh16sh546	Main Chinese clade I	Positive	non-cefRaziR	Gaungxi	2014	S	R	S	S	I	R	S	S	R	S	R	S	R
SRR19126700	sh16sh549	Main Chinese clade I	Positive	non-cefRaziR	Gaungxi	2015	S	R	S	S	R	R	S	R	R	S	I	S	R
SRR19126702	sh16sh550	Main Chinese clade I	Positive	non-cefRaziR	Gaungxi	2015	S	R	S	S	R	R	S	R	R	S	R	S	R
SRR19126703	sh16sh552	Main Chinese clade I	Positive	non-cefRaziR	Gaungxi	2015	S	R	S	S	R	R	S	R	R	S	I	S	R
SRR19126704	sh16sh564	Main Chinese clade I	Positive	non-cefRaziR	Gaungxi	2015	S	R	S	S	R	R	S	R	R	S	R	S	R
SRR19126705	sh16sh568	Main Chinese clade I	Positive	non-cefRaziR	Gaungxi	2015	S	R	S	S	R	R	S	R	R	S	I	S	R
SRR19126706	sh16sh573	Main Chinese clade I	Positive	non-cefRaziR	Gaungxi	2016	S	S	S	S	S	S	S	S	S	S	S	S	S
SRR19126707	sh16sh574	Main Chinese clade I	Positive	cefRaziR	Gaungxi	2016	S	R	S	S	R	R	S	R	R	S	R	S	R
SRR19126708	sh16sh575	Main Chinese clade I	Positive	cefRaziR	Gaungxi	2016	S	R	S	S	R	R	S	R	R	S	R	S	R
SRR19126709	sh16sh576	Main Chinese clade I	Positive	cefRaziR	Gaungxi	2016	S	R	S	S	R	R	S	R	R	S	I	S	R

SRR19126710	sh16sh577	Main Chinese clade I	Positive	cefRaziR	Gaungxi	2016	S	R	S	S	R	R	S	R	R	S	R	S	R
SRR19126711	sh16sh578	Main Chinese clade I	Positive	cefRaziR	Gaungxi	2016	S	R	S	S	R	R	S	R	R	S	R	S	R
SRR19126713	sh16sh580	Main Chinese clade I	Positive	cefRaziR	Gaungxi	2016	S	R	S	S	R	R	S	R	R	S	R	S	R
SRR19126714	sh16sh581	Main Chinese clade I	Positive	cefRaziR	Gaungxi	2016	S	R	S	S	I	R	S	R	R	S	I	S	R
SRR19126715	sh16sh587	Main Chinese clade I	Positive	cefRaziR	Gaungxi	2016	S	R	S	S	I	R	S	R	R	S	R	S	R
SRR19126716	sh16sh588	Main Chinese clade I	Positive	cefRaziR	Gaungxi	2016	S	R	S	S	I	R	S	R	R	S	R	S	R
SRR22671129	sh16sh589	Main Chinese clade II	Negative	non-cefRaziR	Gaungxi	2016	S	R	S	S	I	R	S	R	R	S	R	S	R
SRR22671000	sh188	Main Chinese clade II	Negative	non-cefRaziR	Shanghai	2011	S	S	S	S	R	R	S	S	S	S	I	S	R
SRR22671240	sh19sh001	Main Chinese clade I	Positive	non-cefRaziR	Gaungxi	2019	S	S	S	S	S	R	S	S	S	S	S	S	S
SRR19126717	sh19sh002	Main Chinese clade I	Positive	cefRaziR	Gaungxi	2019	S	R	S	S	R	R	S	R	R	S	R	S	R
SRR19126718	sh19sh003	Main Chinese clade I	Positive	cefRaziR	Gaungxi	2019	S	R	S	S	R	R	S	R	R	S	R	S	R
SRR19126719	sh19sh004	Main Chinese clade I	Positive	cefRaziR	Gaungxi	2019	S	R	S	S	R	R	S	R	R	S	R	S	R
SRR19126720	sh19sh005	Main Chinese clade I	Positive	cefRaziR	Gaungxi	2019	S	R	S	S	R	R	S	R	R	S	R	S	R
SRR19126721	sh19sh006	Main Chinese clade I	Positive	cefRaziR	Gaungxi	2019	S	R	S	S	R	R	S	R	R	S	R	S	R
SRR19126722	sh19sh007	Main Chinese clade I	Positive	cefRaziR	Gaungxi	2019	S	R	S	S	R	R	S	R	R	S	R	S	R
SRR19126724	sh19sh008	Main Chinese clade I	Positive	cefRaziR	Gaungxi	2019	S	R	S	S	R	R	S	R	R	S	R	S	R
SRR19126725	sh19sh009	Main Chinese clade I	Positive	cefRaziR	Gaungxi	2019	S	R	S	S	R	R	S	R	R	S	R	S	R
SRR19126726	sh19sh010	Main Chinese clade I	Positive	cefRaziR	Gaungxi	2019	S	R	S	S	R	R	S	R	R	S	R	S	R
SRR19126727	sh19sh011	Main Chinese clade I	Positive	cefRaziR	Gaungxi	2019	S	R	S	S	R	R	S	R	R	S	R	S	R
SRR19126728	sh19sh012	Main Chinese clade I	Positive	cefRaziR	Gaungxi	2019	S	R	S	S	R	R	S	R	R	S	R	S	R
SRR19126729	sh19sh013	Main Chinese clade I	Positive	cefRaziR	Gaungxi	2019	S	R	S	S	R	R	S	R	R	S	R	S	R
SRR19126730	sh19sh014	Main Chinese clade I	Positive	cefRaziR	Gaungxi	2019	S	R	S	S	R	R	S	R	R	S	R	S	R
SRR19126731	sh19sh015	Main Chinese clade I	Positive	cefRaziR	Gaungxi	2019	S	R	S	S	R	R	S	R	R	S	R	S	R
SRR19126732	sh19sh016	Main Chinese clade I	Positive	cefRaziR	Gaungxi	2019	S	R	S	S	R	R	S	R	R	S	R	S	R
SRR19126733	sh19sh017	Main Chinese clade I	Positive	cefRaziR	Gaungxi	2019	S	R	S	S	R	R	S	R	R	S	R	S	R
SRR19126735	sh19sh018	Main Chinese clade I	Positive	cefRaziR	Gaungxi	2019	S	R	S	S	R	R	S	R	R	S	R	S	R
SRR19126736	sh19sh019	Main Chinese clade I	Positive	cefRaziR	Gaungxi	2019	S	R	S	S	R	R	S	R	R	S	R	S	R
SRR19126737	sh19sh020	Main Chinese clade I	Positive	cefRaziR	Gaungxi	2019	S	R	S	S	R	R	S	R	R	S	R	S	R
SRR19126738	sh19sh021	Main Chinese clade I	Positive	cefRaziR	Gaungxi	2019	S	R	S	S	R	R	S	R	R	S	R	S	R
SRR19126739	sh19sh022	Main Chinese clade I	Positive	cefRaziR	Gaungxi	2019	S	R	S	S	R	R	S	R	R	S	R	S	R
SRR19126740	sh19sh023	Main Chinese clade I	Positive	cefRaziR	Gaungxi	2019	S	R	S	S	R	R	S	R	R	S	R	S	R
SRR19126741	sh19sh024	Main Chinese clade I	Positive	cefRaziR	Gaungxi	2019	S	R	S	S	R	R	S	R	R	S	R	S	R
SRR19126742	sh19sh025	Main Chinese clade I	Positive	cefRaziR	Gaungxi	2019	I	R	S	S	R	R	S	R	R	S	R	S	R
SRR19126743	sh19sh026	Main Chinese clade I	Positive	cefRaziR	Gaungxi	2019	S	R	S	S	R	R	S	R	R	S	R	S	R
SRR19126744	sh19sh027	Main Chinese clade I	Positive	cefRaziR	Gaungxi	2019	S	R	S	S	R	R	S	R	R	S	R	S	R
SRR19126746	sh19sh028	Main Chinese clade I	Positive	cefRaziR	Gaungxi	2019	S	R	S	S	R	R	S	R	R	S	R	S	R
SRR19126747	sh19sh029	Main Chinese clade I	Positive	cefRaziR	Gaungxi	2019	S	R	S	S	R	R	S	R	R	S	R	S	R
SRR19126748	sh19sh030	Main Chinese clade I	Positive	cefRaziR	Gaungxi	2019	S	R	S	S	R	R	S	R	R	S	R	S	R
SRR19126749	sh19sh031	Main Chinese clade I	Positive	cefRaziR	Gaungxi	2019	S	R	S	S	R	R	S	R	R	S	R	S	R
SRR19126750	sh19sh032	Main Chinese clade I	Positive	cefRaziR	Gaungxi	2019	S	R	S	S	R	R	S	R	R	S	R	S	R
SRR19126751	sh19sh033	Main Chinese clade I	Positive	cefRaziR	Gaungxi	2019	S	R	S	S	R	S	S	R	R	S	R	S	R
SRR19126752	sh19sh034	Main Chinese clade I	Positive	cefRaziR	Gaungxi	2019	S	R	S	S	R	R	S	R	R	S	R	S	R
SRR19126753	sh19sh035	Main Chinese clade I	Positive	cefRaziR	Gaungxi	2019	S	R	S	S	R	R	S	R	R	S	R	S	R
SRR19126754	sh19sh036	Main Chinese clade I	Positive	cefRaziR	Gaungxi	2019	S	R	S	S	R	R	S	R	R	S	R	S	R
SRR19126755	sh19sh037	Main Chinese clade I	Positive	cefRaziR	Gaungxi	2019	S	R	S	S	R	R	S	R	R	S	R	S	R
SRR19126757	sh19sh038	Main Chinese clade I	Positive	cefRaziR	Gaungxi	2019	S	R	S	S	R	R	S	R	R	S	R	S	R
SRR19126758	sh19sh039	Main Chinese clade I	Positive	cefRaziR	Gaungxi	2019	S	R	S	S	R	R	S	R	R	S	R	S	R
SRR19126759	sh19sh040	Main Chinese clade I	Positive	cefRaziR	Gaungxi	2019	S	R	S	S	R	R	S	R	R	S	R	S	R
SRR19126760	sh19sh041	Main Chinese clade I	Positive	cefRaziR	Gaungxi	2019	S	R	S	S	R	R	S	R	R	S	R	S	R
SRR19126761	sh19sh042	Main Chinese clade I	Positive	cefRaziR	Gaungxi	2019	S	R	S	S	R	R	S	R	R	S	R	S	R
SRR19126762	sh19sh043	Main Chinese clade I	Positive	cefRaziR	Gaungxi	2019	S	R	S	S	R	R	S	R	R	S	R	S	R
SRR19126763	sh19sh044	Main Chinese clade I	Positive	cefRaziR	Gaungxi	2019	S	R	S	S	R	R	S	R	R	S	R	S	R
SRR19126764	sh19sh045	Main Chinese clade I	Positive	cefRaziR	Gaungxi	2019	S	R	S	S	R	R	S	R	R	S	R	S	R
SRR19126765	sh19sh046	Main Chinese clade I	Positive	cefRaziR	Gaungxi	2019	S	R	S	S	R	R	S	R	R	S	R	S	R

SRR19126773	sh19sh047	Main Chinese clade I	Positive	cefRaziR	Gaungxi	2019	S	R	S	S	R	R	S	R	R	S	R	S	R
SRR19126893	sh19sh048	Main Chinese clade I	Positive	cefRaziR	Gaungxi	2019	S	R	S	S	R	R	S	R	R	S	R	S	R
SRR19126904	sh19sh049	Main Chinese clade I	Positive	cefRaziR	Gaungxi	2019	S	R	S	S	R	R	S	R	R	S	R	S	R
SRR22671011	sh236	Main Chinese clade II	Positive	non-cefRaziR	Shanghai	2011	S	S	S	S	R	R	S	S	S	S	I	S	R
SRR22671022	sn0603	Main Chinese clade II	Positive	non-cefRaziR	Gansu	2006	S	S	S	S	S	R	S	S	S	S	I	S	R
SRR19126915	sn0701	Main Chinese clade IV	Positive	non-cefRaziR	Gansu	2007	S	R	S	S	R	R	S	R	R	S	R	S	R
SRR19126818	sn0707	Main Chinese clade IV	Positive	cefRaziR	Gansu	2007	S	R	S	S	R	R	S	R	R	S	R	S	R
SRR19126829	sn0713	Main Chinese clade IV	Positive	cefRaziR	Gansu	2007	S	R	S	S	R	R	S	R	R	S	R	S	R
SRR19126840	sn0714	Main Chinese clade IV	Positive	cefRaziR	Gansu	2007	S	R	S	S	R	R	S	R	R	S	I	S	R
SRR19126851	sn0716	Main Chinese clade IV	Positive	non-cefRaziR	Gansu	2007	S	R	S	S	R	R	S	R	R	S	I	S	R
SRR19126790	sn0717	Main Chinese clade IV	Positive	cefRaziR	Gansu	2007	S	R	S	S	R	R	S	R	R	S	R	S	R
SRR19126801	sn0719	Main Chinese clade IV	Positive	cefRaziR	Gansu	2007	S	R	S	S	R	R	S	I	S	S	I	S	R
SRR19126812	sn0720	Main Chinese clade IV	Positive	non-cefRaziR	Gansu	2007	S	R	S	S	R	R	S	R	R	S	R	S	R
SRR19126860	sn0722	Main Chinese clade IV	Positive	cefRaziR	Gansu	2007	S	R	S	S	R	R	S	R	R	S	R	S	R
SRR19126871	sn0723	Main Chinese clade IV	Positive	non-cefRaziR	Gansu	2007	S	R	S	S	R	R	S	R	R	S	R	S	R
SRR19126882	sn0724	Main Chinese clade IV	Positive	cefRaziR	Gansu	2007	S	R	S	S	R	R	S	R	R	S	I	S	R
SRR19126929	sn0729	Main Chinese clade IV	Positive	cefRaziR	Gansu	2007	S	R	S	S	R	R	S	R	R	S	I	S	R
SRR19126940	sn0730	Main Chinese clade IV	Positive	cefRaziR	Gansu	2007	S	R	S	S	R	R	S	R	R	S	I	S	R
SRR19126951	sn0741	Main Chinese clade IV	Positive	cefRaziR	Gansu	2007	S	R	S	S	R	R	S	R	R	S	R	S	R
SRR19126962	sn0742	Main Chinese clade IV	Positive	cefRaziR	Gansu	2007	S	R	S	S	R	R	S	R	R	S	R	S	R
SRR19126973	sn0743	Main Chinese clade IV	Positive	cefRaziR	Gansu	2007	S	R	S	S	R	R	S	R	R	S	R	S	R
SRR22671066	sn0801	Main Chinese clade II	Positive	non-cefRaziR	Gansu	2008	S	S	S	S	S	R	S	S	S	S	I	S	R
SRR19126984	sn0806	Main Chinese clade I	Negative	non-cefRaziR	Gansu	2008	S	S	S	S	S	R	S	S	S	S	S	S	S
SRR19126995	sn0815	Main Chinese clade I	Negative	non-cefRaziR	Gansu	2008	S	S	S	S	S	R	S	S	R	S	S	S	S
SRR19126701	sn0932	Main Chinese clade I	Negative	non-cefRaziR	Gansu	2009	S	S	S	S	S	R	S	S	S	S	S	S	S
SRR19126712	sn0947	Main Chinese clade I	Negative	non-cefRaziR	Gansu	2009	S	S	S	S	S	R	S	S	S	S	S	S	S
SRR19126723	sn1004	Main Chinese clade I	Negative	non-cefRaziR	Gansu	2010	S	S	S	S	S	R	S	S	S	S	S	S	S
SRR19126734	sn1016	Main Chinese clade I	Negative	cefRaziR	Gansu	2010	S	R	S	S	R	R	S	R	R	S	S	S	S
SRR19126745	sn1017	Main Chinese clade I	Negative	non-cefRaziR	Gansu	2010	S	S	S	S	S	R	S	S	S	S	S	S	S
SRR22671077	sn1103	Main Chinese clade II	Negative	non-cefRaziR	Gansu	2011	S	S	S	S	R	R	S	S	S	S	R	S	R
SRR22671088	sn1104	Main Chinese clade II	Negative	non-cefRaziR	Gansu	2011	S	S	S	S	R	R	S	S	S	S	I	S	R
SRR22671135	sn1201	Main Chinese clade II	Negative	non-cefRaziR	Gansu	2012	S	S	S	S	S	R	S	S	S	S	S	S	S
SRR22671146	SY230422	Main Chinese clade II	Positive	non-cefRaziR	Liaoning	2004	S	S	S	S	S	R	S	S	S	S	S	S	S
SRR19126756	SY230719	Main Chinese clade I	Positive	non-cefRaziR	Liaoning	2007	S	S	S	S	S	R	S	S	S	S	S	S	S
SRR22671157	WH13	Main Chinese clade II	Negative	non-cefRaziR	Hubei	2011	S	S	S	S	R	R	S	S	S	S	I	S	R
SRR22671168	WH25	Main Chinese clade II	Negative	non-cefRaziR	Hubei	2012	S	R	S	S	R	S	S	R	R	S	S	S	S
SRR22671179	WH52	Main Chinese clade II	Positive	non-cefRaziR	Hubei	2006	S	R	S	S	R	S	S	R	R	S	S	S	S
SRR19126774	WH7	Main Chinese clade I	Positive	non-cefRaziR	Hubei	2011	S	R	S	S	R	R	S	R	R	S	R	S	R
SRR22671190	WH82	Main Chinese clade II	Negative	non-cefRaziR	Hubei	2010	S	S	S	S	R	R	S	S	S	S	I	S	R
SRR19126813	WH88	Main Chinese clade I	Positive	non-cefRaziR	Hubei	2010	I	R	S	S	I	R	S	S	R	I	I	S	R
SRR22671239	X8	Main Chinese clade II	Negative	non-cefRaziR	Anhui	2010	S	S	S	S	R	R	S	S	S	R	I	S	R
SRR22671036	XJ12140	Main Chinese clade II	Negative	non-cefRaziR	Xinjiang	2012	S	S	S	S	S	R	S	S	S	S	S	S	S
SRR22671047	XJ12212	Main Chinese clade II	Negative	non-cefRaziR	Xinjiang	2012	S	S	S	S	S	R	S	S	S	S	S	S	S
SRR22671058	XJ2006096	Main Chinese clade II	Positive	non-cefRaziR	Xinjiang	2006	S	S	S	S	S	R	S	S	S	S	S	S	S
SRR22670969	XJ2010010	Main Chinese clade II	Positive	non-cefRaziR	Xinjiang	2010	S	R	S	S	R	R	S	R	R	S	S	S	S
SRR19126996	XJ2012013	Main Chinese clade III	Positive	non-cefRaziR	Xinjiang	2011	S	S	S	S	S	R	S	S	S	S	S	S	S
SRR22670980	XJ2014176	Main Chinese clade II	Positive	non-cefRaziR	Xinjiang	2014	S	S	S	S	S	R	S	S	S	S	S	S	S
SRR22670991	YN14	Main Chinese clade II	Negative	non-cefRaziR	Yunnan	2013	S	S	S	S	R	R	S	S	S	S	I	S	R
SRR22671241	ZD15029	Main Chinese clade I	Positive	non-cefRaziR	Shanxi	2016	S	S	S	S	S	R	S	S	S	S	I	S	R
SRR22671212	ZD16030	Main Chinese clade II	Positive	non-cefRaziR	Shanxi	2016	S	S	S	S	S	R	S	S	S	S	I	S	R
CP000038.1	Ref	Main Chinese clade II		non-cefRaziR	China	/	-	-	-	-	-	-	-	-	-	-	-	-	-
SRR5029673	SRR5029673	other strains in Lineage III	-	non-cefRaziR	UK	2015	-	-	-	-	-	-	-	-	-	-	-	-	-
SRR5023700	SRR5023700	other strains in Lineage III	-	non-cefRaziR	UK	2015	-	-	-	-	-	-	-	-	-	-	-	-	-
SRR5006072	SRR5006072	other strains in Lineage III	-	non-cefRaziR	UK	2015	-	-	-	-	-	-	-	-	-	-	-	-	-

SRR5005327	SRR5005327	other strains in Lineage III	-	non-cefRaziR	UK	2015	-	-	-	-	-	-	-	-	-	-	-	-	-
SRR5005299	SRR5005299	other strains in Lineage III	-	non-cefRaziR	UK	2015	-	-	-	-	-	-	-	-	-	-	-	-	-
SRR4788222	SRR4788222	other strains in Lineage III	-	non-cefRaziR	UK	2015	-	-	-	-	-	-	-	-	-	-	-	-	-
SRR4787278	SRR4787278	other strains in Lineage III	-	non-cefRaziR	UK	2015	-	-	-	-	-	-	-	-	-	-	-	-	-
SRR4787219	SRR4787219	other strains in Lineage III	-	cefRaziR	UK	2015	-	-	-	-	-	-	-	-	-	-	-	-	-
SRR4786326	SRR4786326	other strains in Lineage III	-	cefRaziR	UK	2015	-	-	-	-	-	-	-	-	-	-	-	-	-
SRR4195740	SRR4195740	other strains in Lineage III	-	non-cefRaziR	UK	2016	-	-	-	-	-	-	-	-	-	-	-	-	-
SRR4195487	SRR4195487	other strains in Lineage III	-	non-cefRaziR	UK	2015	-	-	-	-	-	-	-	-	-	-	-	-	-
SRR4192106	SRR4192106	other strains in Lineage III	-	non-cefRaziR	UK	2015	-	-	-	-	-	-	-	-	-	-	-	-	-
SRR3531909	SRR3531909	other strains in Lineage III	-	non-cefRaziR	UK	2015	-	-	-	-	-	-	-	-	-	-	-	-	-
SRR3531903	SRR3531903	other strains in Lineage III	-	cefRaziR	UK	2015	-	-	-	-	-	-	-	-	-	-	-	-	-
SRR3531897	SRR3531897	other strains in Lineage III	-	non-cefRaziR	UK	2015	-	-	-	-	-	-	-	-	-	-	-	-	-
SRR3531887	SRR3531887	other strains in Lineage III	-	cefRaziR	UK	2016	-	-	-	-	-	-	-	-	-	-	-	-	-
SRR3531882	SRR3531882	other strains in Lineage III	-	non-cefRaziR	UK	2016	-	-	-	-	-	-	-	-	-	-	-	-	-
SRR3531874	SRR3531874	other strains in Lineage III	-	non-cefRaziR	UK	2015	-	-	-	-	-	-	-	-	-	-	-	-	-
SRR3531866	SRR3531866	other strains in Lineage III	-	non-cefRaziR	UK	2015	-	-	-	-	-	-	-	-	-	-	-	-	-
SRR3531782	SRR3531782	other strains in Lineage III	-	cefRaziR	UK	2015	-	-	-	-	-	-	-	-	-	-	-	-	-
SRR3531721	SRR3531721	other strains in Lineage III	-	non-cefRaziR	UK	2015	-	-	-	-	-	-	-	-	-	-	-	-	-
SRR3531253	SRR3531253	other strains in Lineage III	-	cefRaziR	UK	2015	-	-	-	-	-	-	-	-	-	-	-	-	-
SRR3531231	SRR3531231	other strains in Lineage III	-	cefRaziR	UK	2015	-	-	-	-	-	-	-	-	-	-	-	-	-
SRR3531087	SRR3531087	other strains in Lineage III	-	non-cefRaziR	UK	2015	-	-	-	-	-	-	-	-	-	-	-	-	-
SRR3531029	SRR3531029	other strains in Lineage III	-	non-cefRaziR	UK	2015	-	-	-	-	-	-	-	-	-	-	-	-	-
SRR3531022	SRR3531022	other strains in Lineage III	-	cefRaziR	UK	2015	-	-	-	-	-	-	-	-	-	-	-	-	-
SRR3530807	SRR3530807	other strains in Lineage III	-	non-cefRaziR	UK	2015	-	-	-	-	-	-	-	-	-	-	-	-	-
SRR3530795	SRR3530795	other strains in Lineage III	-	cefRaziR	UK	2015	-	-	-	-	-	-	-	-	-	-	-	-	-
SRR3530773	SRR3530773	other strains in Lineage III	-	cefRaziR	UK	2015	-	-	-	-	-	-	-	-	-	-	-	-	-
SRR3530640	SRR3530640	other strains in Lineage III	-	non-cefRaziR	UK	2015	-	-	-	-	-	-	-	-	-	-	-	-	-
SRR3530638	SRR3530638	other strains in Lineage III	-	non-cefRaziR	UK	2015	-	-	-	-	-	-	-	-	-	-	-	-	-
SRR3530636	SRR3530636	other strains in Lineage III	-	non-cefRaziR	UK	2015	-	-	-	-	-	-	-	-	-	-	-	-	-
SRR3530555	SRR3530555	other strains in Lineage III	-	non-cefRaziR	UK	2015	-	-	-	-	-	-	-	-	-	-	-	-	-
SRR3530549	SRR3530549	other strains in Lineage III	-	non-cefRaziR	UK	2015	-	-	-	-	-	-	-	-	-	-	-	-	-
SRR3530435	SRR3530435	other strains in Lineage III	-	non-cefRaziR	UK	2015	-	-	-	-	-	-	-	-	-	-	-	-	-
SRR3530393	SRR3530393	other strains in Lineage III	-	non-cefRaziR	UK	2015	-	-	-	-	-	-	-	-	-	-	-	-	-
ERR1541598	ERR1541598	Main Chinese clade II	-	non-cefRaziR	France	2013	-	-	-	-	-	-	-	-	-	-	-	-	-
ERR1541589	ERR1541589	other strains in Lineage III	-	non-cefRaziR	France	2013	-	-	-	-	-	-	-	-	-	-	-	-	-
ERR1541578	ERR1541578	other strains in Lineage III	-	non-cefRaziR	France	2012	-	-	-	-	-	-	-	-	-	-	-	-	-
ERR1516071	ERR1516071	other strains in Lineage III	-	non-cefRaziR	France	2010	-	-	-	-	-	-	-	-	-	-	-	-	-
ERR1364329	ERR1364329	other strains in Lineage III	-	non-cefRaziR	UK	2014	-	-	-	-	-	-	-	-	-	-	-	-	-
ERR1364327	ERR1364327	other strains in Lineage III	-	non-cefRaziR	UK	2014	-	-	-	-	-	-	-	-	-	-	-	-	-
ERR1364293	ERR1364293	other strains in Lineage III	-	non-cefRaziR	UK	2013	-	-	-	-	-	-	-	-	-	-	-	-	-
ERR1364277	ERR1364277	other strains in Lineage III	-	non-cefRaziR	UK	2012	-	-	-	-	-	-	-	-	-	-	-	-	-
ERR1364276	ERR1364276	other strains in Lineage III	-	non-cefRaziR	UK	2012	-	-	-	-	-	-	-	-	-	-	-	-	-
ERR1364263	ERR1364263	other strains in Lineage III	-	non-cefRaziR	UK	2012	-	-	-	-	-	-	-	-	-	-	-	-	-
ERR1364257	ERR1364257	other strains in Lineage III	-	non-cefRaziR	UK	2011	-	-	-	-	-	-	-	-	-	-	-	-	-
ERR1364253	ERR1364253	other strains in Lineage III	-	non-cefRaziR	UK	2011	-	-	-	-	-	-	-	-	-	-	-	-	-
ERR1364242	ERR1364242	other strains in Lineage III	-	non-cefRaziR	UK	2011	-	-	-	-	-	-	-	-	-	-	-	-	-
ERR1364234	ERR1364234	other strains in Lineage III	-	non-cefRaziR	UK	2011	-	-	-	-	-	-	-	-	-	-	-	-	-
ERR1364227	ERR1364227	other strains in Lineage III	-	non-cefRaziR	UK	2010	-	-	-	-	-	-	-	-	-	-	-	-	-
ERR1364216	ERR1364216	other strains in Lineage III	-	non-cefRaziR	UK	2010	-	-	-	-	-	-	-	-	-	-	-	-	-
ERR1364207	ERR1364207	other strains in Lineage III	-	non-cefRaziR	UK	2010	-	-	-	-	-	-	-	-	-	-	-	-	-
ERR1364206	ERR1364206	other strains in Lineage III	-	non-cefRaziR	UK	2010	-	-	-	-	-	-	-	-	-	-	-	-	-
ERR1364193	ERR1364193	other strains in Lineage III	-	non-cefRaziR	UK	2009	-	-	-	-	-	-	-	-	-	-	-	-	-
ERR1364192	ERR1364192	other strains in Lineage III	-	non-cefRaziR	UK	2009	-	-	-	-	-	-	-	-	-	-	-	-	-

ERR1364191	ERR1364191	other strains in Lineage III	-	non-cefRaziR	UK	2009	-	-	-	-	-	-	-	-	-	-	-	-	-
ERR1364170	ERR1364170	other strains in Lineage III	-	non-cefRaziR	UK	2009	-	-	-	-	-	-	-	-	-	-	-	-	-
ERR028706	ERR028706	Lineage II	-	non-cefRaziR	outhAmeric	1997	-	-	-	-	-	-	-	-	-	-	-	-	-
ERR028705	ERR028705	other strains in Lineage III	-	non-cefRaziR	outhAmeric	2000	-	-	-	-	-	-	-	-	-	-	-	-	-
ERR028704	ERR028704	other strains in Lineage III	-	non-cefRaziR	outhAmeric	2000	-	-	-	-	-	-	-	-	-	-	-	-	-
ERR028703	ERR028703	other strains in Lineage III	-	non-cefRaziR	Central Asia	2006	-	-	-	-	-	-	-	-	-	-	-	-	-
ERR028699	ERR028699	Main Chinese clade II	-	non-cefRaziR	Egypt	2006	-	-	-	-	-	-	-	-	-	-	-	-	-
ERR028695	ERR028695	other strains in Lineage III	-	non-cefRaziR	Europe	2001	-	-	-	-	-	-	-	-	-	-	-	-	-
ERR028694	ERR028694	other strains in Lineage III	-	non-cefRaziR	Europe	1990	-	-	-	-	-	-	-	-	-	-	-	-	-
ERR028692	ERR028692	Lineage I	-	non-cefRaziR	Europe	2004	-	-	-	-	-	-	-	-	-	-	-	-	-
ERR028691	ERR028691	other strains in Lineage III	-	non-cefRaziR	an/Central /	1998	-	-	-	-	-	-	-	-	-	-	-	-	-
ERR028690	ERR028690	other strains in Lineage III	-	non-cefRaziR	MiddleEast	2003	-	-	-	-	-	-	-	-	-	-	-	-	-
ERR028689	ERR028689	other strains in Lineage III	-	non-cefRaziR	Europe	2002	-	-	-	-	-	-	-	-	-	-	-	-	-
ERR028688	ERR028688	other strains in Lineage III	-	non-cefRaziR	Europe	2000	-	-	-	-	-	-	-	-	-	-	-	-	-
ERR028685	ERR028685	other strains in Lineage III	-	non-cefRaziR	outhAmeric	2002	-	-	-	-	-	-	-	-	-	-	-	-	-
ERR028679	ERR028679	other strains in Lineage III	-	non-cefRaziR	Central Asia	2004	-	-	-	-	-	-	-	-	-	-	-	-	-
ERR028676	ERR028676	other strains in Lineage III	-	non-cefRaziR	Europe	2004	-	-	-	-	-	-	-	-	-	-	-	-	-
ERR028675	ERR028675	Lineage II	-	non-cefRaziR	Europe	1991	-	-	-	-	-	-	-	-	-	-	-	-	-
ERR028672	ERR028672	Lineage II	-	non-cefRaziR	an/Central /	2006	-	-	-	-	-	-	-	-	-	-	-	-	-
ERR025768	ERR025768	other strains in Lineage III	-	non-cefRaziR	an/Central /	2003	-	-	-	-	-	-	-	-	-	-	-	-	-
ERR025767	ERR025767	other strains in Lineage III	-	non-cefRaziR	MiddleEast	2003	-	-	-	-	-	-	-	-	-	-	-	-	-
ERR025765	ERR025765	Lineage I	-	non-cefRaziR	rica & Mad	2000	-	-	-	-	-	-	-	-	-	-	-	-	-
ERR025762	ERR025762	Lineage II	-	non-cefRaziR	rica & Mad	1998	-	-	-	-	-	-	-	-	-	-	-	-	-
ERR025761	ERR025761	other strains in Lineage III	-	non-cefRaziR	rica & Mad	1998	-	-	-	-	-	-	-	-	-	-	-	-	-
ERR025758	ERR025758	other strains in Lineage III	-	non-cefRaziR	outhAmeric	1998	-	-	-	-	-	-	-	-	-	-	-	-	-
ERR025756	ERR025756	Lineage I	-	non-cefRaziR	Europe	1976	-	-	-	-	-	-	-	-	-	-	-	-	-
ERR025755	ERR025755	Lineage I	-	non-cefRaziR	Europe	1974	-	-	-	-	-	-	-	-	-	-	-	-	-
ERR025754	ERR025754	Lineage I	-	non-cefRaziR	Europe	1974	-	-	-	-	-	-	-	-	-	-	-	-	-
ERR025753	ERR025753	Lineage II	-	non-cefRaziR	Europe	1974	-	-	-	-	-	-	-	-	-	-	-	-	-
ERR025751	ERR025751	Lineage II	-	non-cefRaziR	West/Centra	1973	-	-	-	-	-	-	-	-	-	-	-	-	-
ERR025750	ERR025750	other strains in Lineage III	-	non-cefRaziR	West/Centra	1973	-	-	-	-	-	-	-	-	-	-	-	-	-
ERR025749	ERR025749	other strains in Lineage III	-	non-cefRaziR	Europe	1973	-	-	-	-	-	-	-	-	-	-	-	-	-
ERR025748	ERR025748	other strains in Lineage III	-	non-cefRaziR	Pacific	1997	-	-	-	-	-	-	-	-	-	-	-	-	-
ERR025747	ERR025747	other strains in Lineage III	-	non-cefRaziR	Europe	1983	-	-	-	-	-	-	-	-	-	-	-	-	-
ERR025746	ERR025746	Lineage II	-	non-cefRaziR	Europe	1973	-	-	-	-	-	-	-	-	-	-	-	-	-
ERR025743	ERR025743	Lineage II	-	non-cefRaziR	Europe	1966	-	-	-	-	-	-	-	-	-	-	-	-	-
ERR025742	ERR025742	Lineage I	-	non-cefRaziR	Europe	1965	-	-	-	-	-	-	-	-	-	-	-	-	-
ERR025737	ERR025737	Lineage IV	-	non-cefRaziR	Europe	1959	-	-	-	-	-	-	-	-	-	-	-	-	-
ERR025734	ERR025734	Lineage II	-	non-cefRaziR	Europe	1958	-	-	-	-	-	-	-	-	-	-	-	-	-
ERR025732	ERR025732	other strains in Lineage III	-	non-cefRaziR	Vietnam	2010	-	-	-	-	-	-	-	-	-	-	-	-	-
ERR025731	ERR025731	Lineage II	-	non-cefRaziR	Europe	1945	-	-	-	-	-	-	-	-	-	-	-	-	-
ERR025729	ERR025729	other strains in Lineage III	-	non-cefRaziR	Europe	1945	-	-	-	-	-	-	-	-	-	-	-	-	-
ERR025726	ERR025726	Lineage II	-	non-cefRaziR	Europe	1945	-	-	-	-	-	-	-	-	-	-	-	-	-
ERR025725	ERR025725	other strains in Lineage III	-	non-cefRaziR	Europe	1979	-	-	-	-	-	-	-	-	-	-	-	-	-
ERR025724	ERR025724	Lineage II	-	non-cefRaziR	Europe	1947	-	-	-	-	-	-	-	-	-	-	-	-	-
ERR025721	ERR025721	other strains in Lineage III	-	non-cefRaziR	Central Asia	2003	-	-	-	-	-	-	-	-	-	-	-	-	-
ERR025719	ERR025719	other strains in Lineage III	-	non-cefRaziR	Central Asia	2002	-	-	-	-	-	-	-	-	-	-	-	-	-
ERR025718	ERR025718	other strains in Lineage III	-	non-cefRaziR	Central Asia	2002	-	-	-	-	-	-	-	-	-	-	-	-	-
ERR025717	ERR025717	other strains in Lineage III	-	non-cefRaziR	Central Asia	2002	-	-	-	-	-	-	-	-	-	-	-	-	-
ERR025716	ERR025716	other strains in Lineage III	-	non-cefRaziR	Europe	1979	-	-	-	-	-	-	-	-	-	-	-	-	-
ERR025715	ERR025715	Lineage II	-	non-cefRaziR	Korea	2011	-	-	-	-	-	-	-	-	-	-	-	-	-
ERR025714	ERR025714	other strains in Lineage III	-	non-cefRaziR	Vietnam	2003	-	-	-	-	-	-	-	-	-	-	-	-	-
ERR025713	ERR025713	other strains in Lineage III	-	non-cefRaziR	Vietnam	2002	-	-	-	-	-	-	-	-	-	-	-	-	-
ERR025712	ERR025712	other strains in Lineage III	-	non-cefRaziR	Vietnam	2002	-	-	-	-	-	-	-	-	-	-	-	-	-

ERR025711	ERR025711	other strains in Lineage III	-	non-cefRaziR	Central Asia	2003	-	-	-	-	-	-	-	-	-	-	-	-	-
ERR025710	ERR025710	other strains in Lineage III	-	non-cefRaziR	Central Asia	2003	-	-	-	-	-	-	-	-	-	-	-	-	-
ERR025709	ERR025709	other strains in Lineage III	-	non-cefRaziR	Central Asia	2003	-	-	-	-	-	-	-	-	-	-	-	-	-
ERR025708	ERR025708	other strains in Lineage III	-	non-cefRaziR	Vietnam	2002	-	-	-	-	-	-	-	-	-	-	-	-	-
ERR025706	ERR025706	other strains in Lineage III	-	non-cefRaziR	Vietnam	2003	-	-	-	-	-	-	-	-	-	-	-	-	-
ERR025705	ERR025705	other strains in Lineage III	-	non-cefRaziR	Vietnam	2003	-	-	-	-	-	-	-	-	-	-	-	-	-
ERR025704	ERR025704	other strains in Lineage III	-	non-cefRaziR	Vietnam	2003	-	-	-	-	-	-	-	-	-	-	-	-	-
ERR025703	ERR025703	other strains in Lineage III	-	non-cefRaziR	Vietnam	2003	-	-	-	-	-	-	-	-	-	-	-	-	-
ERR025701	ERR025701	other strains in Lineage III	-	non-cefRaziR	Vietnam	2003	-	-	-	-	-	-	-	-	-	-	-	-	-
ERR025700	ERR025700	other strains in Lineage III	-	non-cefRaziR	Vietnam	2003	-	-	-	-	-	-	-	-	-	-	-	-	-
ERR025699	ERR025699	other strains in Lineage III	-	non-cefRaziR	Vietnam	2002	-	-	-	-	-	-	-	-	-	-	-	-	-
ERR025698	ERR025698	other strains in Lineage III	-	non-cefRaziR	Vietnam	2001	-	-	-	-	-	-	-	-	-	-	-	-	-
ERR025697	ERR025697	other strains in Lineage III	-	non-cefRaziR	Vietnam	2001	-	-	-	-	-	-	-	-	-	-	-	-	-
ERR025695	ERR025695	other strains in Lineage III	-	non-cefRaziR	Vietnam	2002	-	-	-	-	-	-	-	-	-	-	-	-	-
ERR025693	ERR025693	Lineage II	-	non-cefRaziR	Korea	1987	-	-	-	-	-	-	-	-	-	-	-	-	-
ERR025692	ERR025692	Lineage II	-	non-cefRaziR	Korea	1985	-	-	-	-	-	-	-	-	-	-	-	-	-
ERR025691	ERR025691	Lineage II	-	non-cefRaziR	Korea	1982	-	-	-	-	-	-	-	-	-	-	-	-	-
ERR025690	ERR025690	Lineage II	-	non-cefRaziR	Korea	1981	-	-	-	-	-	-	-	-	-	-	-	-	-
ERR025687	ERR025687	Lineage II	-	non-cefRaziR	Korea	1982	-	-	-	-	-	-	-	-	-	-	-	-	-
ERR025686	ERR025686	Lineage II	-	non-cefRaziR	Korea	1980	-	-	-	-	-	-	-	-	-	-	-	-	-
ERR025685	ERR025685	other strains in Lineage III	-	non-cefRaziR	Vietnam	2002	-	-	-	-	-	-	-	-	-	-	-	-	-
ERR025683	ERR025683	other strains in Lineage III	-	non-cefRaziR	Vietnam	2001	-	-	-	-	-	-	-	-	-	-	-	-	-
ERR025682	ERR025682	Lineage II	-	non-cefRaziR	Korea	1983	-	-	-	-	-	-	-	-	-	-	-	-	-
ERR024627	ERR024627	Main Chinese clade I	-	non-cefRaziR	Korea	1998	-	-	-	-	-	-	-	-	-	-	-	-	-
ERR024626	ERR024626	other strains in Lineage III	-	non-cefRaziR	Korea	1994	-	-	-	-	-	-	-	-	-	-	-	-	-
ERR024625	ERR024625	other strains in Lineage III	-	non-cefRaziR	Korea	1991	-	-	-	-	-	-	-	-	-	-	-	-	-
ERR024622	ERR024622	other strains in Lineage III	-	non-cefRaziR	Vietnam	2010	-	-	-	-	-	-	-	-	-	-	-	-	-
ERR024620	ERR024620	other strains in Lineage III	-	non-cefRaziR	Korea	2003	-	-	-	-	-	-	-	-	-	-	-	-	-
ERR024619	ERR024619	Lineage II	-	non-cefRaziR	Korea	1979	-	-	-	-	-	-	-	-	-	-	-	-	-
ERR024618	ERR024618	Main Chinese clade I	-	non-cefRaziR	Korea	1999	-	-	-	-	-	-	-	-	-	-	-	-	-
ERR024617	ERR024617	Main Chinese clade I	-	non-cefRaziR	Korea	2000	-	-	-	-	-	-	-	-	-	-	-	-	-
ERR024616	ERR024616	Main Chinese clade I	-	non-cefRaziR	Korea	2003	-	-	-	-	-	-	-	-	-	-	-	-	-
ERR024614	ERR024614	other strains in Lineage III	-	non-cefRaziR	Europe	2006	-	-	-	-	-	-	-	-	-	-	-	-	-
ERR024612	ERR024612	other strains in Lineage III	-	non-cefRaziR	West/Centra	2006	-	-	-	-	-	-	-	-	-	-	-	-	-
ERR024611	ERR024611	other strains in Lineage III	-	non-cefRaziR	West/Centra	2006	-	-	-	-	-	-	-	-	-	-	-	-	-
ERR024610	ERR024610	other strains in Lineage III	-	non-cefRaziR	West/Centra	2006	-	-	-	-	-	-	-	-	-	-	-	-	-
ERR024609	ERR024609	other strains in Lineage III	-	non-cefRaziR	West/Centra	2006	-	-	-	-	-	-	-	-	-	-	-	-	-
ERR024608	ERR024608	other strains in Lineage III	-	non-cefRaziR	outhAmeric	2006	-	-	-	-	-	-	-	-	-	-	-	-	-
ERR024607	ERR024607	Lineage II	-	non-cefRaziR	West/Centra	2005	-	-	-	-	-	-	-	-	-	-	-	-	-
ERR024606	ERR024606	Lineage I	-	non-cefRaziR	Europe	2007	-	-	-	-	-	-	-	-	-	-	-	-	-
ERR024605	ERR024605	Lineage II	-	non-cefRaziR	an/Central A	2006	-	-	-	-	-	-	-	-	-	-	-	-	-
ERR024604	ERR024604	Lineage II	-	non-cefRaziR	rica & Mad	2004	-	-	-	-	-	-	-	-	-	-	-	-	-
ERR024085	ERR024085	other strains in Lineage III	-	non-cefRaziR	outhAmeric	2005	-	-	-	-	-	-	-	-	-	-	-	-	-
ERR024079	ERR024079	other strains in Lineage III	-	non-cefRaziR	outhAmeric	2001	-	-	-	-	-	-	-	-	-	-	-	-	-
ERR024078	ERR024078	other strains in Lineage III	-	non-cefRaziR	outhAmeric	2000	-	-	-	-	-	-	-	-	-	-	-	-	-
ERR024076	ERR024076	other strains in Lineage III	-	non-cefRaziR	Egypt	2006	-	-	-	-	-	-	-	-	-	-	-	-	-

Ticarcillin	illin/clavulaS	Aztreonam	Ampicillin	hlorampheS	cprim/sulfame	Norfloxacin	Amikacin	Azithromycin	Colistin	AAC(3)-IId	AAC(3)-IIc	AAC(3)-IV	AAC(6')-Ib7	AAC(6')-Ib9	AAC(6')-Ib-	aadA15	aadA2	aadA22	aadA23
R	S	S	R	S	R	S	S	R	S	96.5	0	0	0	0	0	0	0	0	0
R	I	R	R	S	R	S	S	R	S	96.5	0	0	0	0	0	0	0	0	0
R	S	R	R	R	R	S	S	S	S	96.5	0	0	0	0	0	0	0	0	0
R	S	S	R	R	R	S	S	R	S	96.5	0	0	0	0	0	0	0	0	0
S	S	S	S	S	R	S	S	S	S	0	0	0	0	0	0	0	0	0	0
S	S	S	S	S	S	S	S	S	S	0	0	0	0	0	0	0	0	0	0
R	R	S	R	S	R	S	S	S	S	0	0	0	0	0	0	0	0	0	0
S	S	S	S	S	R	S	S	S	S	0	0	0	0	0	0	0	0	0	0
R	I	I	R	S	R	S	S	R	S	96.5	0	0	0	0	0	0	0	0	0
S	S	S	S	S	R	S	S	S	S	96.5	0	0	0	0	0	0	0	0	0
R	S	S	R	S	R	S	S	S	S	96.5	0	0	0	0	0	0	0	0	0
R	R	S	R	S	R	S	S	S	S	96.5	0	0	0	0	0	0	0	0	0
R	S	R	R	S	R	S	S	S	S	0	0	0	0	0	0	0	0	0	0
R	I	I	R	S	R	S	S	R	S	96.15	0	0	0	0	0	0	0	0	0
R	I	I	R	S	R	S	S	R	S	96.15	0	0	0	0	0	0	0	0	0
R	I	S	R	S	R	S	S	R	S	96.15	0	0	0	0	0	0	0	0	0
R	I	S	R	S	R	S	S	R	S	96.15	0	0	0	0	0	0	0	0	0
R	I	S	R	S	R	S	S	R	S	96.15	0	0	0	0	0	0	0	0	0
R	I	S	R	S	R	S	S	R	S	96.15	0	0	0	0	0	0	0	0	0
R	I	S	R	S	R	S	S	R	S	96.15	0	0	0	0	0	0	0	0	0
R	I	S	R	S	R	S	S	R	S	96.15	0	0	0	0	0	0	0	0	0
R	I	S	R	S	R	S	S	R	S	96.15	0	0	0	0	0	0	0	0	0
R	I	S	R	S	R	S	S	R	S	96.15	0	0	0	0	0	0	0	0	0
R	I	S	R	S	R	S	S	R	S	96.15	0	0	0	0	0	0	0	0	0
R	I	S	R	S	R	S	S	R	S	96.15	0	0	0	0	0	0	0	0	0
R	I	S	R	S	R	S	S	R	S	96.15	0	0	0	0	0	0	0	0	0
R	I	S	R	S	R	S	S	R	S	96.15	0	0	0	0	0	0	0	0	0
R	I	S	R	S	R	S	S	R	S	96.15	0	0	0	0	0	0	0	0	0
R	I	S	R	S	R	S	S	R	S	96.15	0	0	0	0	0	0	0	0	0
R	I	S	R	S	R	S	S	R	S	96.15	0	0	0	0	0	0	0	0	0
R	I	S	R	S	R	S	S	R	S	96.15	0	0	0	0	0	0	0	0	0
R	I	S	R	S	R	S	S	R	S	96.15	0	0	0	0	0	0	0	0	0
R	I	S	R	S	R	S	S	R	S	96.15	0	0	0	0	0	0	0	0	0
R	I	S	R	S	R	S	S	R	S	96.15	0	0	0	0	0	0	0	0	0
R	I	S	R	S	R	S	S	R	S	96.15	0	0	0	0	0	0	0	0	0
R	I	S	R	S	R	S	S	R	S	96.15	0	0	0	0	0	0	0	0	0
R	I	S	R	S	R	S	S	R	S	96.15	0	0	0	0	0	0	0	0	0
R	I	S	R	S	R	S	S	R	S	96.15	0	0	0	0	0	0	0	0	0
R	I	S	R	S	R	S	S	R	S	96.15	0	0	0	0	0	0	0	0	0
R	I	S	R	S	R	S	S	R	S	96.15	0	0	0	0	0	0	0	0	0
R	I	S	R	S	R	S	S	R	S	96.15	0	0	0	0	0	0	0	0	0
R	I	S	R	S	R	S	S	R	S	96.15	0	0	0	0	0	0	0	0	0
R	I	S	R	S	R	S	S	R	S	96.15	0	0	0	0	0	0	0	0	0
R	I	S	R	S	R	S	S	R	S	96.15	0	0	0	0	0	0	0	0	0
R	I	S	R	S	R	S	S	R	S	96.15	0	0	0	0	0	0	0	0	0
R	I	S	R	S	R	S	S	R	S	96.15	0	0	0	0	0	0	0	0	0
R	I	S	R	S	R	S	S	R	S	96.15	0	0	0	0	0	0	0	0	0
R	I	I	R	S	R	S	S	R	S	96.15	0	0	0	0	0	0	0	0	0
R	I	I	R	S	R	S	S	R	S	96.15	0	0	0	0	0	0	0	0	0
R	I	S	R	S	R	S	S	R	S	96.15	0	0	0	0	0	0	0	0	0
R	I	I	R	S	R	S	S	R	S	96.15	0	0	0	0	0	0	0	0	0

R	I	S	R	S	R	S	S	R	S	96.5	0	0	0	0	0	0	0	0	0
R	S	R	R	S	S	S	S	R	S	96.5	0	0	0	0	0	0	0	0	0
R	S	R	R	S	R	S	S	S	S	96.5	100	0	0	0	0	0	0	0	0
R	I	S	R	S	R	S	S	R	S	96.5	0	0	0	0	0	0	0	0	0
R	S	R	R	R	R	S	S	S	S	0	0	0	0	0	0	0	0	0	98.61
R	S	S	R	S	R	S	S	S	S	96.5	100	0	0	0	0	0	0	97.75	0
R	S	S	R	S	R	S	S	S	S	96.5	0	0	0	0	0	0	0	0	0
R	I	S	R	S	R	S	S	S	S	96.5	0	0	0	0	0	0	0	0	0
R	S	S	R	S	R	S	S	S	S	96.5	0	0	0	0	0	0	0	0	0
R	S	S	R	S	R	S	S	S	S	96.5	0	0	0	0	0	0	0	0	0
S	S	S	S	S	S	S	S	S	S	0	0	0	0	0	0	0	0	0	0
S	S	S	S	S	S	S	S	S	S	0	0	0	0	0	0	0	0	0	0
R	S	S	R	S	R	S	S	S	S	96.5	0	0	0	0	0	0	0	0	0
R	R	R	R	S	R	S	S	S	S	96.5	0	0	0	0	0	0	0	0	0
R	I	S	R	S	R	S	S	S	S	0	0	0	0	0	0	0	0	0	0
S	S	S	S	S	R	S	S	S	S	0	0	0	0	0	0	0	0	0	0
R	S	S	R	S	R	S	S	S	S	96.5	0	0	0	0	0	0	0	0	0
S	S	S	S	S	R	S	S	S	S	0	0	0	0	0	0	0	0	0	0
S	S	S	S	S	R	S	S	S	S	0	0	0	0	0	0	0	0	0	0
R	S	S	R	S	R	S	S	S	S	96.5	0	0	0	0	0	0	0	0	0
R	R	S	R	S	S	S	S	S	S	96.5	0	0	0	0	0	0	0	0	0
R	I	I	R	S	R	S	S	S	S	96.5	100	0	0	0	0	0	0	0	0
R	R	R	R	S	R	S	S	S	S	96.5	100	0	0	0	0	0	0	0	0
R	R	R	R	S	R	S	S	S	S	96.5	0	0	0	0	0	0	0	0	0
R	R	S	R	S	R	S	S	S	S	96.5	0	0	0	0	0	0	0	0	0
R	S	S	R	S	R	S	S	S	S	96.5	0	0	0	0	0	0	0	0	0
R	R	R	R	S	R	S	S	S	S	96.5	0	0	0	0	0	0	0	0	0
R	R	I	R	S	R	S	S	S	S	96.5	0	0	0	0	0	0	0	0	0
R	S	S	R	S	R	S	S	S	S	96.5	0	0	0	0	0	0	0	0	0
R	S	S	R	S	R	S	S	S	S	96.5	0	0	0	0	0	0	0	0	0
S	S	S	S	S	R	S	S	S	S	0	0	0	0	0	0	0	0	0	0
S	S	S	S	S	R	S	S	S	S	0	0	0	0	0	0	0	0	0	0
R	S	S	R	S	R	S	S	S	S	0	0	0	0	0	0	0	0	0	0
R	S	S	R	R	R	S	S	S	S	0	0	0	0	0	0	0	0	0	0
R	I	S	R	S	R	S	S	S	S	0	0	0	0	0	0	0	0	0	0
R	I	S	R	S	R	S	S	S	S	96.5	0	0	0	0	0	0	0	0	0
R	S	S	R	S	R	S	S	S	S	96.5	0	0	0	0	0	0	0	0	0
R	I	R	R	S	S	S	S	S	S	95.1	0	0	0	0	100	0	0	0	0
R	I	S	R	S	R	S	S	S	S	96.5	0	0	0	0	0	0	0	0	0
R	I	S	R	S	R	S	S	S	S	96.5	0	0	0	0	0	0	0	0	0
R	I	S	R	S	R	S	S	S	S	96.5	0	0	0	0	0	0	0	0	0
R	S	S	R	S	R	S	S	S	S	96.5	0	0	0	0	0	0	0	0	0
R	S	R	R	S	R	S	S	S	S	0	0	0	0	0	0	0	0	0	0
R	S	R	R	S	R	S	S	S	S	96.5	0	0	0	0	0	0	0	0	0
R	S	R	R	S	R	S	S	S	S	0	0	0	0	0	0	0	0	0	0
R	S	S	R	S	R	S	S	S	S	96.15	0	0	0	0	0	0	0	0	0
R	S	S	R	S	R	S	S	S	S	96.15	0	0	0	0	0	0	0	0	0
R	S	S	R	R	R	S	S	S	S	0	0	0	0	0	0	0	0	0	0
S	S	S	S	S	R	S	S	S	S	0	0	0	0	0	0	0	0	0	0
R	S	S	R	S	R	S	S	S	S	96.5	0	0	0	0	0	0	0	0	0
R	S	S	R	S	R	S	S	S	S	96.5	0	0	0	0	0	0	0	0	0
R	S	S	R	S	R	S	S	S	S	96.5	0	0	0	0	0	0	0	0	0
S	S	S	S	S	R	S	S	S	S	0	0	0	0	0	0	0	0	0	0
R	R	R	R	S	R	S	S	S	S	96.5	0	0	0	0	0	0	0	0	0

R	S	S	R	S	R	S	S	S	S	96.5	0	0	0	0	0	0	0	0	0
R	R	I	R	S	R	S	S	S	S	0	0	0	0	0	0	0	0	0	0
R	S	S	R	S	R	S	S	S	S	96.5	0	0	0	0	0	0	0	0	0
R	R	S	R	S	R	S	S	S	S	96.5	0	0	0	0	0	0	0	0	0
R	R	S	R	S	R	S	S	S	S	96.5	0	0	0	0	0	0	0	0	0
R	S	S	R	S	R	S	S	S	S	96.5	0	0	0	0	0	0	0	0	0
R	R	I	R	S	S	S	S	S	S	0	0	0	0	0	0	0	0	0	0
R	R	S	R	S	R	S	S	S	S	96.15	0	0	0	0	0	0	0	0	0
R	S	S	R	S	S	S	S	S	S	0	0	0	0	0	0	0	0	0	0
R	R	R	R	S	R	S	S	S	S	0	0	0	0	0	0	0	0	0	0
R	S	S	R	S	R	S	S	S	S	96.5	0	0	0	0	0	0	0	0	0
R	R	S	R	S	R	S	S	S	S	96.5	0	0	0	0	0	0	0	0	0
R	R	I	R	S	R	S	S	S	S	96.5	0	0	0	0	0	0	0	0	0
R	R	R	R	S	R	S	S	S	S	0	0	0	0	0	0	0	0	0	0
S	S	S	S	S	R	S	S	S	S	0	0	0	0	0	0	0	0	0	0
R	R	R	R	S	R	S	S	S	S	0	0	0	0	0	0	0	0	0	0
S	S	S	S	S	R	S	S	S	S	0	0	0	0	0	0	0	0	0	0
S	S	S	S	S	R	S	S	S	S	0	0	0	0	0	0	0	0	0	0
S	S	S	S	S	R	S	S	S	S	0	0	0	0	0	0	0	0	0	0
R	S	R	R	S	R	S	S	S	S	0	0	0	0	0	0	0	0	0	0
R	R	R	R	S	R	S	S	S	S	0	0	0	0	0	0	0	0	0	0
R	S	R	R	S	R	S	S	S	S	0	0	0	0	0	0	0	0	0	0
R	S	R	R	S	R	S	S	S	S	0	0	0	0	0	0	0	0	0	0
S	S	S	S	S	R	S	S	S	S	0	0	0	0	0	0	0	0	0	0
R	S	S	R	S	R	S	S	S	S	96.5	0	0	0	0	0	0	0	0	0
R	S	S	R	S	S	S	S	S	S	96.5	0	0	0	0	0	0	0	0	0
R	S	S	R	S	R	S	S	S	S	96.5	0	0	0	0	0	0	0	0	0
R	R	S	R	S	R	S	S	S	S	96.5	0	0	0	0	0	0	0	0	0
R	R	R	R	S	R	S	S	S	S	96.5	0	0	0	0	0	0	0	0	0
R	R	S	R	S	R	S	S	S	S	96.5	0	0	0	0	0	0	0	0	0
R	S	R	R	R	R	S	S	S	S	96.5	0	0	0	0	0	0	0	0	0
R	S	S	R	S	R	S	S	S	S	0	0	0	0	0	0	0	0	0	0
R	S	S	R	S	R	S	S	S	S	96.5	0	0	0	0	0	0	0	0	0
R	R	R	R	S	R	S	S	S	S	96.5	0	0	0	0	0	0	0	0	0
R	R	S	R	S	R	S	S	S	S	96.5	0	0	0	0	0	0	0	0	0
R	R	S	R	S	R	S	S	S	S	96.5	0	0	0	0	0	0	0	0	0
R	R	R	R	S	R	S	S	S	S	96.5	0	0	0	0	0	0	0	0	0
S	S	S	S	S	S	S	S	S	S	0	0	0	0	0	0	0	0	0	0
R	S	R	R	R	R	S	S	S	S	96.5	0	0	0	0	0	0	0	0	0
R	S	S	R	S	R	S	S	S	S	96.5	0	0	0	0	0	0	0	0	0
R	S	R	R	S	R	S	S	S	S	0	0	0	0	0	0	0	0	0	0
R	R	R	R	S	R	S	S	S	S	96.5	0	0	0	0	0	0	0	0	0
R	R	S	R	S	R	S	S	S	S	96.5	0	0	0	0	0	0	0	0	0
S	S	S	S	S	R	S	S	S	S	0	0	0	0	0	0	0	0	0	0
R	S	S	R	S	S	S	S	S	S	0	0	0	0	0	0	0	0	0	0
R	S	S	R	S	R	S	S	S	S	0	0	0	0	0	0	0	0	0	0
R	S	S	R	S	R	S	S	S	S	96.5	0	0	0	0	0	0	0	0	0
R	S	R	R	S	S	S	S	S	S	0	0	0	0	0	0	0	0	0	0
S	S	S	S	S	R	S	S	S	S	0	0	0	0	0	0	0	0	0	0
R	S	S	R	S	R	S	S	S	S	96.5	0	0	0	0	0	0	0	0	0
R	S	R	R	S	R	S	S	S	S	0	0	0	0	0	0	0	0	0	0
S	S	S	S	S	R	S	S	S	S	0	0	0	0	0	0	0	0	0	0
R	R	S	R	S	R	S	S	S	S	96.5	0	0	0	0	0	0	0	0	0
R	S	R	R	S	R	S	S	S	S	0	0	0	0	0	0	0	0	0	0

R	S	S	R	S	R	S	S	S	S	96.5	0	0	0	0	0	0	0	0	0
R	S	S	R	S	R	S	S	S	S	96.5	0	0	0	0	0	0	0	0	0
R	S	S	R	S	R	S	S	S	S	96.5	0	0	0	0	0	0	0	0	0
R	R	R	R	S	R	S	S	S	S	96.5	0	0	0	0	0	0	0	0	0
R	R	I	R	R	R	S	S	S	S	96.5	0	0	0	0	0	0	0	0	0
R	S	S	R	S	R	S	S	S	S	96.5	0	0	0	0	0	0	0	0	0
R	R	R	R	S	R	S	S	S	S	96.5	0	0	0	0	0	0	0	0	0
R	R	S	R	S	S	S	S	S	S	96.5	0	0	0	0	0	0	0	0	0
S	S	S	S	S	R	S	S	S	S	0	0	0	0	0	0	0	0	0	0
R	S	R	R	S	R	S	S	S	S	0	0	0	0	0	0	0	0	0	0
R	R	I	R	S	R	S	S	S	S	96.5	0	0	0	0	0	0	0	0	0
S	S	S	S	S	R	S	S	S	S	0	0	0	0	0	0	0	0	0	0
R	S	S	R	S	S	S	S	S	S	0	0	0	0	0	0	0	0	0	0
R	S	S	R	S	R	S	S	S	S	96.5	0	0	0	0	0	0	0	0	0
R	S	S	R	S	R	S	S	S	S	96.5	0	0	0	0	0	0	0	0	0
R	R	R	R	S	R	S	S	S	S	96.5	0	0	0	0	0	0	0	0	0
R	R	R	R	S	R	S	S	S	S	0	0	0	0	0	0	0	0	0	0
S	S	S	S	S	R	S	S	S	S	0	0	0	0	0	0	0	0	0	0
R	S	R	R	S	R	S	S	S	S	0	0	0	0	0	0	0	0	0	0
R	S	R	R	S	R	S	S	S	S	0	0	0	0	0	0	0	0	0	0
R	S	S	R	S	R	S	S	S	S	96.5	0	0	0	0	0	0	0	0	0
R	S	S	R	S	R	S	S	S	S	96.5	0	0	0	0	0	0	0	0	0
R	R	S	R	S	R	S	S	S	S	96.5	0	0	0	0	0	0	0	0	0
R	S	S	R	S	R	S	S	S	S	96.5	0	0	0	0	0	0	0	0	0
R	R	R	R	S	R	S	S	S	S	96.5	0	0	0	0	0	0	0	0	0
R	S	S	R	S	R	S	S	S	S	96.5	0	0	0	0	0	0	0	0	0
R	R	S	R	S	R	S	S	S	S	96.5	0	0	0	0	0	0	0	0	0
R	S	R	R	S	R	S	S	S	S	0	0	0	0	0	0	0	0	0	0
R	S	S	R	S	R	S	S	S	S	0	0	0	0	0	0	0	0	0	0
R	R	I	R	S	R	S	S	S	S	95.1	0	0	0	0	0	0	0	0	0
R	R	S	R	S	R	S	S	S	S	95.1	0	0	0	0	0	0	0	0	0
R	S	S	R	S	R	S	S	S	S	0	0	0	0	0	0	0	0	0	0
S	S	S	S	S	S	S	S	S	S	0	0	0	0	0	0	0	0	0	0
R	R	S	R	S	R	S	S	S	S	96.5	0	0	0	0	0	0	0	0	0
R	S	S	R	R	R	S	S	S	S	0	0	0	0	0	0	0	0	0	0
R	R	S	R	S	R	S	S	S	S	96.5	0	0	0	0	0	0	0	0	0
S	S	S	S	S	S	S	S	S	S	0	0	0	0	0	0	0	0	0	0
R	R	S	R	S	R	S	S	S	S	96.5	0	0	0	0	0	0	0	0	0
R	R	R	R	S	R	S	S	S	S	96.5	0	0	0	0	0	0	0	0	0
R	S	R	R	S	R	S	S	S	S	0	0	0	0	0	0	0	0	0	0
R	R	R	R	S	R	S	S	S	S	0	0	0	0	0	0	0	0	0	0
R	R	S	R	S	R	S	S	S	S	96.5	95.74	0	0	0	0	0	0	0	0
R	R	R	R	S	R	S	S	S	S	0	0	0	0	0	0	0	0	0	0
R	R	R	R	S	S	S	S	S	S	0	0	0	0	0	0	0	0	0	0
R	R	R	R	S	R	S	S	S	S	96.5	0	0	0	0	0	0	0	0	0
R	S	S	R	S	S	S	S	S	S	0	0	0	0	0	0	0	0	0	0
R	S	R	R	S	R	S	S	S	S	0	0	0	0	0	0	0	0	0	0
R	R	R	R	S	R	S	S	S	S	96.5	0	0	0	0	0	0	0	0	0
R	S	R	R	R	R	S	S	S	S	0	0	0	0	0	0	0	0	0	0
R	R	R	R	S	R	S	S	S	S	96.5	0	0	0	0	0	0	0	0	0
R	R	R	R	S	R	S	S	S	S	96.5	0	0	0	0	0	0	0	0	0
R	R	R	R	S	R	S	S	S	S	96.5	0	0	0	0	0	0	0	0	0
R	R	S	R	S	R	S	S	S	S	96.5	0	0	0	0	0	0	0	0	0
R	R	R	R	S	R	S	S	S	S	96.5	0	0	0	0	0	0	0	0	0

R	R	R	R	S	R	S	S	S	S	96.5	0	0	0	0	0	0	0	0	0
R	R	S	R	S	R	S	S	S	S	96.5	0	0	0	0	0	0	0	0	0
R	R	R	R	S	R	S	S	S	S	96.5	0	0	0	0	0	0	0	0	0
R	R	R	R	R	R	S	S	S	S	95.1	0	0	99.05	0	0	0	0	0	0
S	S	S	S	S	S	S	S	S	S	0	0	0	0	0	0	0	0	0	0
R	R	I	R	S	R	S	S	S	S	96.5	0	0	0	0	0	0	0	0	0
S	S	S	S	S	R	S	S	S	S	0	0	0	0	0	0	0	0	0	0
S	S	S	S	S	R	S	S	S	S	0	0	0	0	0	0	0	0	0	0
R	R	R	R	S	R	S	S	S	S	96.5	0	0	0	0	0	0	0	0	0
S	S	S	S	S	R	S	S	S	S	0	0	0	0	0	0	0	0	0	0
R	R	I	R	S	R	S	S	S	S	96.5	0	0	0	0	0	0	0	0	0
R	S	R	R	R	R	S	S	S	S	96.5	0	0	0	0	0	0	0	0	0
R	S	R	R	R	R	S	S	S	S	0	0	0	0	0	0	0	0	0	0
R	S	S	R	S	R	S	S	S	S	96.5	0	0	0	0	0	0	0	0	0
R	S	R	R	S	S	S	S	S	S	0	0	0	0	0	0	0	0	0	0
R	S	R	R	S	S	S	S	S	S	0	0	0	0	0	0	0	0	0	0
S	S	S	S	S	S	S	S	S	S	0	0	0	0	0	0	0	0	0	0
R	R	R	R	S	R	S	S	S	S	96.5	0	0	0	0	0	0	0	0	0
R	R	R	R	S	R	S	S	S	S	96.5	0	0	0	0	0	0	0	0	0
R	S	S	R	S	R	S	S	S	S	96.5	0	0	0	0	0	0	0	0	0
S	S	S	S	S	S	S	S	S	S	0	0	0	0	0	0	0	0	0	0
R	R	R	R	S	R	S	S	S	S	0	0	0	0	0	0	0	0	0	0
R	S	S	R	S	R	S	S	S	S	96.5	0	0	0	0	0	0	0	0	0
R	I	R	R	S	R	S	S	S	S	96.5	0	0	0	0	0	0	0	0	0
R	I	I	R	S	S	S	S	S	S	0	0	0	0	0	0	0	0	0	0
R	R	I	R	S	R	S	S	R	S	96.5	0	0	0	0	0	0	0	0	0
R	I	R	R	S	S	S	S	S	S	0	0	0	0	0	0	0	0	0	0
R	I	I	R	S	R	S	S	R	S	0	0	0	0	0	0	0	0	0	0
R	S	S	R	S	R	S	S	S	S	96.5	0	0	0	0	0	0	0	0	0
S	S	S	S	S	R	S	S	S	S	0	0	0	0	0	0	0	0	0	0
R	I	I	R	S	S	S	S	R	S	0	0	0	0	0	0	0	0	0	0
R	I	S	R	S	R	S	S	R	S	96.5	100	0	0	0	0	0	0	0	0
R	I	S	R	S	R	S	S	R	S	96.5	0	0	0	0	0	0	0	0	0
R	S	S	R	S	R	S	S	R	S	0	0	0	0	0	0	0	0	0	0
R	I	S	R	S	R	S	S	R	S	96.5	0	0	0	0	0	0	0	0	0
S	S	S	S	S	S	S	S	S	R	96.5	100	0	0	0	0	0	0	0	0
R	I	S	R	S	R	S	S	R	S	96.5	0	0	0	0	0	0	0	0	0
R	I	S	R	S	R	S	S	R	S	96.5	0	0	0	0	0	0	0	0	0
R	I	R	R	S	R	S	S	S	S	96.5	0	0	0	0	0	0	0	0	0
R	I	R	R	S	S	S	S	R	S	0	0	0	0	0	0	0	0	0	0
R	R	R	R	S	R	S	S	S	S	96.5	0	0	0	0	0	0	0	0	0
R	I	R	R	S	R	S	S	S	S	96.5	0	0	0	0	0	0	0	0	0
R	S	S	R	S	S	S	S	R	S	0	0	0	0	0	0	0	0	0	0
R	S	S	R	S	R	S	S	S	S	96.5	0	0	0	0	0	0	0	0	0
R	S	S	R	S	S	S	S	R	S	0	0	0	0	0	0	0	0	0	0
R	I	R	R	S	R	S	S	R	S	96.5	100	0	0	0	0	0	0	0	0
R	S	S	R	S	R	S	S	S	S	96.5	0	0	0	0	0	0	0	0	0
R	I	S	R	S	S	S	S	R	S	0	0	0	0	0	0	0	0	0	0
R	I	S	R	S	S	S	S	R	S	0	0	0	0	0	0	0	0	0	0
R	I	S	R	S	R	S	S	R	S	96.5	100	0	0	0	0	0	0	0	0
R	I	I	R	S	R	S	S	R	S	0	0	0	0	0	0	0	0	0	0
R	I	R	R	S	R	S	S	S	S	96.5	0	0	0	0	0	0	0	0	0
R	S	S	R	S	S	S	S	R	S	0	0	0	0	0	0	0	0	0	0
R	I	I	R	S	R	S	S	R	S	96.5	0	0	0	0	0	0	0	0	0

R	I	I	R	S	R	S	S	R	S	96.5	0	0	0	0	0	0	0	0	0
R	I	R	R	S	R	S	S	R	S	96.5	0	0	0	0	0	0	0	0	0
S	S	S	S	S	S	S	S	S	S	0	0	0	0	0	0	0	0	0	0
R	I	S	R	S	R	S	S	R	S	96.5	0	0	0	0	0	0	0	0	0
R	I	I	R	R	R	S	S	R	S	96.5	0	0	0	0	0	0	0	0	0
R	I	I	R	S	S	S	S	R	S	0	0	0	0	0	0	0	0	0	0
R	S	S	R	S	R	S	S	R	S	0	0	0	0	0	0	0	0	0	0
R	R	S	R	S	S	S	S	R	S	0	0	0	0	0	0	0	0	0	0
R	S	S	R	S	R	S	S	R	S	0	0	0	0	0	0	0	0	0	0
R	I	I	R	S	R	S	S	R	S	0	0	0	0	0	0	0	0	0	0
R	I	S	R	S	R	S	S	R	S	96.5	0	0	0	0	0	0	0	0	0
R	I	S	R	S	R	S	S	R	S	96.5	0	0	0	0	0	0	0	0	0
R	I	I	R	S	R	S	S	R	S	0	0	0	0	0	0	0	0	0	0
R	S	S	R	S	S	S	S	R	S	0	0	0	0	0	0	0	0	0	0
R	I	S	R	S	R	S	S	R	S	96.5	0	0	0	0	0	0	0	0	0
R	S	S	R	S	R	S	S	R	S	96.5	0	0	0	0	0	0	0	0	0
R	I	S	R	S	R	S	S	R	S	96.5	0	0	0	0	0	0	0	0	0
R	S	S	R	S	S	S	S	R	S	96.5	0	0	0	0	0	0	0	0	0
R	I	S	R	S	R	S	S	R	S	96.5	0	0	0	0	0	0	0	0	0
R	I	S	R	S	R	S	S	R	S	96.5	0	0	0	0	0	0	0	0	0
R	I	S	R	S	R	S	S	R	S	0	0	0	0	0	0	0	0	0	0
R	I	S	R	S	R	S	S	R	S	96.5	0	0	0	0	0	0	0	0	0
R	S	S	R	S	R	S	S	R	S	96.5	0	0	0	0	0	0	0	0	0
R	I	I	R	S	R	S	S	R	S	0	0	0	0	0	0	0	0	0	0
R	S	I	R	S	S	S	S	R	S	96.5	0	0	0	0	0	0	0	0	0
R	S	S	R	S	R	S	S	R	S	96.5	0	0	0	0	0	0	0	0	0
R	R	R	R	S	S	S	S	S	S	0	0	0	0	0	0	0	0	0	0
R	I	S	R	S	R	S	S	R	S	96.5	0	0	0	0	0	0	0	0	0
R	I	R	R	S	R	S	S	R	S	96.5	0	0	0	0	0	0	0	0	0
R	R	S	R	S	R	S	S	S	S	96.5	0	0	0	0	0	0	0	0	0
R	R	S	R	S	R	S	S	S	S	96.5	0	0	0	0	0	0	0	0	0
R	I	S	R	S	R	S	S	R	S	0	0	0	0	0	0	0	0	0	0
R	R	S	R	S	R	S	S	S	S	96.5	0	0	0	0	0	0	0	0	0
R	S	R	R	S	R	S	S	R	S	96.5	0	0	0	0	0	0	0	0	0
S	S	S	S	S	S	S	S	S	R	96.5	0	0	0	0	0	0	0	0	0
R	S	S	R	S	R	S	S	S	S	96.5	0	0	0	0	0	0	0	0	0
R	S	S	R	S	R	S	S	S	S	96.5	0	0	0	0	0	0	0	0	0
R	S	S	R	S	R	S	S	S	S	96.5	0	0	0	0	0	0	0	0	0
R	S	S	R	S	R	S	S	S	S	96.5	0	0	0	0	0	0	0	0	0
R	S	S	R	S	R	S	S	S	S	96.5	0	0	0	0	0	0	0	0	0
R	I	R	R	S	R	S	S	R	S	96.5	0	0	0	0	0	0	0	0	0
R	I	S	R	S	R	S	S	R	S	96.5	0	0	0	0	0	0	0	0	0
R	S	S	R	S	R	S	S	S	S	96.5	0	0	0	0	0	0	0	0	0
R	I	I	R	S	R	S	S	R	S	96.5	0	0	0	0	0	0	0	0	0
R	I	S	R	S	R	S	S	R	S	96.5	0	0	0	0	0	0	0	0	0
R	S	S	R	S	R	S	S	S	S	96.5	0	0	0	0	0	0	0	0	0
R	I	R	R	S	R	S	S	R	S	96.5	0	0	0	0	0	0	0	0	0
R	R	S	R	S	R	S	S	R	S	0	0	0	0	0	0	0	0	0	0
R	I	S	R	S	R	S	S	R	S	0	0	0	0	0	0	0	0	0	0
R	I	I	R	S	R	S	S	R	S	96.5	0	0	0	0	0	0	0	0	0
R	S	S	R	S	R	S	S	R	S	96.5	0	0	0	0	0	0	0	0	0
R	R	S	R	S	R	S	S	S	S	96.5	0	0	0	0	0	0	0	0	0
R	I	S	R	S	S	S	S	R	S	0	0	0	0	0	0	0	0	0	0
R	I	S	R	S	S	S	S	R	S	0	0	0	0	0	0	0	0	0	0

R	I	S	R	S	R	S	S	R	S	96.5	0	0	0	0	0	0	0	0	0
R	I	S	R	S	S	S	S	R	S	0	0	0	0	0	0	0	0	0	0
R	I	I	R	S	R	S	S	R	S	96.5	0	0	0	0	0	0	0	0	0
R	I	S	R	S	R	S	S	R	S	96.5	0	0	0	0	0	0	0	0	0
R	I	S	R	S	R	S	S	R	S	96.5	0	0	0	0	0	0	0	0	0
R	I	I	R	S	R	S	S	R	S	0	0	0	0	0	0	0	0	0	0
R	I	S	R	S	R	S	S	R	S	96.5	0	0	0	0	0	0	0	0	0
R	I	I	R	S	R	S	S	R	S	96.5	0	0	0	0	0	0	0	0	0
R	I	I	R	S	R	S	S	R	S	96.5	0	0	0	0	0	0	0	0	0
R	S	S	R	S	R	S	S	R	S	96.5	0	0	0	0	0	0	0	0	0
R	S	S	R	S	R	S	S	R	S	96.5	0	0	0	0	0	0	0	0	0
R	I	R	R	S	R	S	S	R	S	96.5	0	0	0	0	0	0	0	0	0
R	I	I	R	S	R	S	S	R	S	96.5	0	0	0	0	0	0	0	0	0
R	I	S	R	S	R	S	S	S	R	96.5	0	0	0	0	0	0	0	0	0
R	I	I	R	S	R	S	S	R	S	96.5	0	0	0	0	0	0	0	0	0
R	I	S	R	S	S	S	S	R	S	0	0	0	0	0	0	0	0	0	0
R	I	S	R	S	R	S	S	R	S	96.5	0	0	0	0	0	0	0	0	0
R	I	S	R	S	R	S	S	S	R	96.5	0	0	0	0	0	0	0	0	0
R	I	S	R	S	S	S	S	R	S	96.5	0	0	0	0	0	0	0	0	0
R	S	R	R	S	S	S	S	R	S	0	0	0	0	0	0	0	0	0	0
R	S	S	R	S	R	S	S	R	S	0	0	0	0	0	0	0	0	0	0
R	I	I	R	S	R	S	S	R	S	96.5	0	0	0	0	0	0	0	0	0
R	S	S	R	S	R	S	S	S	S	96.5	0	0	0	0	0	0	0	0	0
R	S	S	R	S	R	S	S	R	S	96.5	0	0	0	0	0	0	0	0	0
R	S	S	R	S	R	S	S	R	S	96.5	0	0	0	0	0	0	0	0	0
R	R	S	R	S	R	S	S	S	S	96.5	0	0	0	0	0	0	0	0	0
R	I	S	R	S	R	S	S	R	S	96.5	0	0	0	0	0	0	0	0	0
R	I	I	R	S	R	S	S	R	S	96.5	0	0	0	0	0	0	0	0	0
R	S	R	R	S	S	S	S	S	S	0	0	0	0	0	0	0	0	0	0
R	S	S	R	S	R	S	S	S	S	96.5	0	0	0	0	0	0	0	0	0
R	I	R	R	S	S	S	S	R	S	0	0	0	0	0	0	0	0	0	0
R	I	S	R	S	R	S	S	S	R	0	0	0	0	0	0	0	0	0	0
R	S	S	R	S	R	S	S	S	S	96.5	0	0	0	0	0	0	0	0	0
R	I	I	R	S	R	S	S	R	S	96.5	0	0	0	0	0	0	0	0	0
S	S	S	S	S	R	S	S	S	S	0	0	0	0	0	0	0	0	0	0
R	S	R	R	S	S	S	S	R	S	0	0	0	0	0	0	0	0	0	0
R	I	R	R	S	S	S	S	R	S	0	0	0	0	0	0	0	0	0	0
R	S	R	R	S	S	S	S	S	S	0	0	0	0	0	0	0	0	0	0
R	S	S	R	S	S	S	S	R	S	0	0	0	0	0	0	0	0	0	0
R	S	R	R	S	R	S	S	R	S	0	0	0	0	0	0	0	0	0	0
R	I	S	R	S	R	S	S	R	S	0	0	0	0	0	0	0	0	0	0
R	S	R	R	S	S	S	S	R	S	0	0	0	0	0	0	0	0	0	0
R	S	S	R	S	R	S	S	S	S	96.5	0	0	0	0	0	0	0	0	0
S	S	S	S	S	R	S	S	S	S	0	0	0	0	0	0	0	0	0	0
S	S	S	S	S	R	S	S	S	S	0	0	0	0	0	0	0	0	0	0
R	I	R	R	S	R	S	S	R	S	96.5	0	0	0	0	0	0	0	0	0
R	S	I	R	S	S	S	S	R	S	0	0	0	0	0	0	0	0	0	0
R	I	S	R	R	R	S	S	R	S	0	0	0	0	99.42	0	0	0	0	98.61
R	S	S	R	S	R	S	S	S	S	96.5	0	0	0	0	0	0	0	0	0
R	S	S	R	S	R	S	S	S	S	96.5	0	0	0	0	0	0	0	0	0
S	S	S	S	S	S	S	S	S	S	0	0	0	0	0	0	0	0	0	0
R	S	R	R	S	R	S	S	S	S	0	0	0	0	0	0	0	0	0	0
R	I	I	R	S	R	S	S	S	S	96.5	100	0	0	0	0	0	0	0	0
R	I	I	R	S	R	S	S	R	S	96.5	0	0	0	0	0	0	0	0	0

R	I	S	R	S	R	S	S	R	S	96.5	0	0	0	0	0	0	0	0	0
R	S	S	R	S	R	S	S	S	S	96.5	0	0	0	0	0	0	0	0	0
R	S	S	R	S	R	S	S	S	S	96.5	0	0	0	0	0	0	0	0	0
S	S	S	S	S	S	S	S	S	S	0	0	0	0	0	0	0	0	0	0
R	S	R	R	S	S	S	S	S	S	0	0	0	0	0	0	0	0	0	0
R	I	S	R	S	R	S	S	R	S	96.5	0	0	0	0	0	0	0	0	0
R	S	S	R	S	R	S	S	S	S	96.5	0	0	0	0	0	0	0	0	0
R	I	R	R	S	R	S	S	R	S	96.5	0	0	0	0	0	0	0	0	0
R	I	S	R	S	R	S	S	S	R	0	0	0	0	0	0	0	0	0	0
R	I	R	R	S	R	S	S	S	S	96.5	0	0	0	0	0	0	0	0	0
R	I	I	R	S	R	S	S	R	S	96.5	0	0	0	0	0	0	0	0	0
R	R	R	R	S	R	S	S	S	S	96.5	100	0	0	0	0	0	0	0	0
R	I	I	R	S	R	S	S	R	S	96.15	0	0	0	0	0	0	0	0	0
S	S	S	S	S	S	S	S	S	S	0	0	0	0	0	0	0	0	0	0
R	S	S	R	S	R	S	S	S	S	96.5	0	0	0	0	0	0	0	0	0
R	I	I	R	S	R	S	S	R	S	96.5	0	0	0	0	0	0	0	0	0
R	S	R	R	S	S	S	S	R	S	0	0	0	0	0	0	0	0	0	0
R	S	S	R	S	R	S	S	S	S	96.5	0	0	0	0	0	0	0	0	0
R	S	S	R	S	R	S	S	S	S	96.5	0	0	0	0	0	0	0	0	0
R	S	S	R	S	R	S	S	S	S	96.5	0	0	0	0	0	0	0	0	0
R	R	R	R	S	R	S	S	S	S	96.5	0	0	0	0	0	0	0	0	0
R	S	S	R	S	R	S	S	S	S	96.5	0	0	0	0	0	0	0	0	0
R	I	I	R	S	R	S	S	R	S	96.5	0	0	0	0	0	0	0	0	0
R	I	I	R	S	R	S	S	R	S	96.5	0	0	0	0	0	0	0	0	0
R	I	S	R	S	R	S	S	R	S	0	0	0	0	0	0	0	0	0	0
R	I	S	R	S	R	S	S	R	S	96.5	0	0	0	0	0	0	0	0	0
R	I	S	R	S	R	S	S	R	S	96.15	0	0	0	0	0	0	0	0	0
R	I	S	R	S	R	S	S	S	S	96.15	0	0	0	0	0	0	0	0	0
R	I	S	R	S	R	S	S	R	S	96.15	0	0	0	0	0	0	0	0	0
R	I	S	R	S	R	S	S	R	S	96.15	0	0	0	0	0	0	0	0	0
R	S	S	R	S	R	S	S	R	S	96.15	0	0	0	0	0	0	0	0	0
R	I	S	R	S	R	S	S	R	S	96.15	0	0	0	0	0	0	0	0	0
R	S	S	R	S	R	S	S	R	S	96.15	0	0	0	0	0	0	0	0	0
R	I	S	R	S	R	S	S	R	S	96.15	0	0	0	0	0	0	0	0	0
R	S	S	R	S	R	S	S	R	S	96.15	0	0	0	0	0	0	0	0	0
R	I	S	R	S	R	S	S	R	S	96.15	0	0	0	0	0	0	0	0	0
R	S	S	R	S	R	S	S	R	S	96.15	0	0	0	0	0	0	0	0	0
R	S	S	R	S	R	S	S	R	S	96.15	0	0	0	0	0	0	0	0	0
R	I	S	R	S	R	S	S	R	S	96.15	0	0	0	0	0	0	0	0	0
R	S	S	R	S	R	S	I	R	S	96.5	0	0	0	0	0	0	0	0	0
R	S	S	R	S	R	S	S	R	S	96.5	0	0	0	0	0	0	0	0	0
R	I	S	R	S	R	S	S	R	S	96.5	0	0	0	0	0	0	0	0	0
R	S	S	R	S	R	S	S	R	S	96.5	0	0	0	0	0	0	0	0	0
R	S	S	R	S	R	S	S	R	S	96.5	0	0	0	0	0	0	0	0	0
R	S	S	R	S	R	S	S	R	S	96.5	0	0	0	0	0	0	0	0	0
R	S	S	R	S	R	S	S	R	S	96.5	0	0	0	0	0	0	0	0	0
R	S	S	R	S	R	S	S	R	S	96.5	0	0	0	0	0	0	0	0	0
R	I	S	R	S	S	S	S	S	S	96.15	0	0	0	0	0	0	0	0	0
R	I	S	R	S	S	S	S	S	S	96.15	0	0	0	0	0	0	0	0	0
R	I	S	R	S	R	S	S	R	R	96.5	0	0	0	0	0	0	0	0	0
R	I	S	R	S	R	S	S	R	S	96.5	0	0	0	0	0	0	0	0	0
R	I	S	R	S	R	S	S	R	S	96.5	0	0	0	0	0	0	0	0	0
R	S	S	R	S	R	S	S	R	R	96.5	0	0	0	0	0	0	0	0	0
R	S	S	R	S	R	S	S	R	R	96.5	0	0	0	0	0	0	0	0	0

R	I	S	R	S	R	S	S	R	R	96.5	0	0	0	0	0	0	0	0	0
R	I	S	R	S	R	S	S	R	S	96.5	0	0	0	0	0	0	0	0	0
R	I	S	R	S	R	S	S	R	S	96.5	0	0	0	0	0	0	0	0	0
R	I	S	R	S	R	S	S	R	S	96.5	0	0	0	0	0	0	0	0	0
R	I	S	R	S	R	S	S	R	R	96.5	0	0	0	0	0	0	0	0	0
R	I	S	R	S	R	S	S	R	R	96.5	0	0	0	0	0	0	0	0	0
R	S	S	R	S	R	S	S	R	R	96.5	0	0	0	0	0	0	0	0	0
R	S	S	R	S	R	S	S	R	R	96.5	0	0	0	0	0	0	0	0	0
R	R	S	R	S	R	S	S	R	R	96.5	0	0	0	0	0	0	0	0	0
R	R	S	R	S	R	S	S	R	R	96.5	0	0	0	0	0	0	0	0	0
R	I	I	R	S	R	S	S	R	R	96.5	0	0	0	0	0	0	0	0	0
R	S	S	R	S	R	S	S	R	S	96.5	0	0	0	0	0	0	0	0	0
R	S	S	R	S	R	S	S	R	R	96.5	0	0	0	0	0	0	0	0	0
R	S	S	R	S	R	S	S	R	R	96.5	0	0	0	0	0	0	0	0	0
R	I	S	R	S	R	S	S	R	R	96.5	0	0	0	0	0	0	0	0	0
R	I	S	R	S	R	S	S	R	R	96.5	0	0	0	0	0	0	0	0	0
R	I	S	R	S	R	S	S	R	R	96.5	0	0	0	0	0	0	0	0	0
R	I	S	R	S	R	S	S	R	R	96.5	0	0	0	0	0	0	0	0	0
R	I	S	R	S	R	S	S	R	R	96.5	0	0	0	0	0	0	0	0	0
R	S	S	R	S	R	S	S	R	R	96.5	0	0	0	0	0	0	0	0	0
R	S	S	R	S	R	S	S	R	R	96.5	0	0	0	0	0	0	0	0	0
R	I	S	R	S	R	S	S	R	R	96.5	0	0	0	0	0	0	0	0	0
R	I	S	R	S	R	S	S	R	R	96.5	0	0	0	0	0	0	0	0	0
R	S	S	R	S	R	S	S	R	R	96.5	0	0	0	0	0	0	0	0	0
R	I	S	R	S	R	S	S	R	S	96.5	0	0	0	0	0	0	0	0	0
R	I	S	R	S	R	S	S	R	R	96.5	0	0	0	0	0	0	0	0	0
R	I	S	R	S	R	S	S	R	R	96.5	0	0	0	0	0	0	0	0	0
R	S	R	R	S	R	S	S	R	S	96.5	0	0	0	0	0	0	0	0	0
R	I	S	R	S	R	S	S	R	R	96.5	0	0	0	0	0	0	0	0	0
R	I	S	R	S	R	S	S	R	S	96.5	0	0	0	0	0	0	0	0	0
R	I	S	R	S	R	S	S	R	S	96.5	0	0	0	0	0	0	0	0	0
R	I	S	R	S	R	S	S	R	R	96.5	0	0	0	0	0	0	0	0	0
R	I	S	R	S	R	S	S	R	R	96.5	0	0	0	0	0	0	0	0	0
R	I	S	R	S	R	S	S	R	R	96.5	0	0	0	0	0	0	0	0	0
R	I	S	R	S	R	S	S	R	R	96.5	0	0	0	0	0	0	0	0	0
R	I	S	R	S	R	S	S	S	S	96.15	0	0	0	0	0	0	0	0	0
R	I	S	R	S	R	S	S	S	S	96.5	0	0	0	0	0	0	0	0	0
R	I	S	R	S	R	S	S	S	S	96.5	0	0	0	0	0	0	0	0	0
R	I	S	R	S	R	S	S	S	S	96.15	0	0	0	0	0	0	0	0	0
R	I	S	R	S	R	S	S	S	S	96.15	0	0	0	0	0	0	0	0	0
R	I	I	R	S	R	S	S	S	S	96.5	0	0	0	0	0	0	0	0	0
R	S	S	R	S	R	S	S	S	S	96.5	0	0	0	0	0	0	0	0	0
R	I	S	R	S	R	S	S	S	S	96.5	0	0	0	0	0	0	0	0	0
R	I	I	R	S	R	S	S	S	S	96.5	0	0	0	0	0	0	0	0	0
R	S	S	R	S	S	S	S	S	S	96.5	0	0	0	0	0	0	0	0	0
R	I	I	R	S	R	S	S	S	S	96.5	0	0	0	0	0	0	0	0	0
R	I	S	R	S	R	S	S	S	S	96.5	0	0	0	0	0	0	0	0	0
R	I	S	R	S	R	S	S	S	S	96.5	0	0	0	0	0	0	0	0	0
R	I	S	R	S	R	S	S	S	S	96.5	0	0	0	0	0	0	0	0	0
R	I	I	R	S	S	S	S	S	S	96.5	0	0	0	0	0	0	0	0	0
S	S	S	S	S	S	S	S	R	S	96.5	0	0	0	0	0	0	0	0	0
R	I	S	R	S	R	S	S	R	S	96.5	0	0	0	0	0	0	0	0	0
R	I	S	R	S	R	S	S	R	S	96.5	0	0	0	0	0	0	0	0	0
R	S	S	R	S	R	S	S	R	S	96.5	0	0	0	0	0	0	0	0	0

R	I	S	R	S	R	S	S	R	S	96.5	0	0	0	0	0	0	0	0	0
R	I	S	R	S	R	S	S	R	S	96.5	0	0	0	0	0	0	0	0	0
R	S	S	R	S	R	S	S	R	S	96.5	0	0	0	0	0	0	0	0	0
R	I	S	R	S	R	S	S	R	S	96.5	0	0	0	0	0	0	0	0	0
R	S	I	R	S	R	S	S	R	S	96.5	0	0	0	0	0	0	0	0	0
R	I	S	R	S	R	S	S	R	S	96.5	0	0	0	0	0	0	0	0	0
R	I	S	R	S	R	S	S	S	S	96.5	0	0	0	0	0	0	0	0	0
R	S	S	R	S	R	S	S	S	S	96.5	0	0	0	0	0	0	0	0	0
S	S	S	S	S	R	S	S	R	S	0	0	0	0	0	0	0	0	0	0
R	I	S	R	S	R	S	S	R	S	96.5	0	0	0	0	0	0	0	0	0
R	I	S	R	S	R	S	S	R	S	96.5	0	0	0	0	0	0	0	0	0
R	I	S	R	S	R	S	S	R	S	96.5	0	0	0	0	0	0	0	0	0
R	I	I	R	S	R	S	S	R	S	96.5	0	0	0	0	0	0	0	0	0
R	I	S	R	S	R	S	S	R	S	96.5	0	0	0	0	0	0	0	0	0
R	I	I	R	S	R	S	S	R	S	96.5	0	0	0	0	0	0	0	0	0
R	I	S	R	S	R	S	S	R	S	96.5	0	0	0	0	0	0	0	0	0
R	I	I	R	S	R	S	S	R	S	96.5	0	0	0	0	0	0	0	0	0
R	I	S	R	S	R	S	S	R	S	96.5	0	0	0	0	0	0	0	0	0
R	I	I	R	S	R	S	S	R	S	96.5	0	0	0	0	0	0	0	0	0
R	I	I	R	S	R	S	S	R	S	96.5	0	0	0	0	0	0	0	0	0
R	I	S	R	S	R	S	S	R	S	96.5	0	0	0	0	0	0	0	0	0
R	I	S	R	S	R	S	S	R	S	96.5	0	0	0	0	0	0	0	0	0
R	I	S	R	S	R	S	S	R	S	96.5	0	0	0	0	0	0	0	0	0
R	I	S	R	S	R	S	S	R	S	96.5	0	0	0	0	0	0	0	0	0
R	I	S	R	S	R	S	S	R	S	96.5	0	0	0	0	0	0	0	0	0
R	I	S	R	S	R	S	S	R	S	96.5	0	0	0	0	0	0	0	0	0
R	I	S	R	S	R	S	S	R	S	96.5	0	0	0	0	0	0	0	0	0
R	I	S	R	S	R	S	S	R	S	96.5	0	0	0	0	0	0	0	0	0
R	I	S	R	S	R	S	S	R	S	96.5	0	0	0	0	0	0	0	0	0
R	I	I	R	S	R	S	S	R	S	96.5	0	0	0	0	0	0	0	0	0
R	I	S	R	S	R	S	S	R	S	96.5	0	0	0	0	0	0	0	0	0
R	I	S	R	S	R	S	S	R	S	96.5	0	0	0	0	0	0	0	0	0
R	I	R	R	S	R	S	S	R	S	96.5	0	0	0	0	0	0	0	0	0
R	I	S	R	S	R	S	S	R	S	96.5	0	0	0	0	0	0	0	0	0
R	I	I	R	S	R	S	S	R	S	96.5	0	0	0	0	0	0	0	0	0
R	I	I	R	S	R	S	S	R	S	96.5	0	0	0	0	0	0	0	0	0
R	I	S	R	S	R	S	S	R	S	96.5	0	0	0	0	0	0	0	0	0
R	I	S	R	S	R	S	S	R	S	96.5	0	0	0	0	0	0	0	0	0
R	I	S	R	S	R	S	S	R	S	96.5	0	0	0	0	0	0	0	0	0
R	I	S	R	S	R	S	S	R	S	96.5	0	0	0	0	0	0	0	0	0
R	I	S	R	S	R	S	S	R	S	96.5	0	0	0	0	0	0	0	0	0
R	I	S	R	S	R	S	S	R	S	96.5	0	0	0	0	0	0	0	0	0
R	I	S	R	S	R	S	S	R	S	96.5	0	0	0	0	0	0	0	0	0
R	I	S	R	S	R	S	S	R	S	96.5	0	0	0	0	0	0	0	0	0
R	I	S	R	S	R	S	S	R	S	96.5	0	0	0	0	0	0	0	0	0
R	I	S	R	S	R	S	S	R	S	96.5	0	0	0	0	0	0	0	0	0
R	I	S	R	S	R	S	S	R	S	96.5	0	0	0	0	0	0	0	0	0
R	I	S	R	S	R	S	S	R	S	96.5	0	0	0	0	0	0	0	0	0
R	I	S	R	S	R	S	S	R	S	96.5	0	0	0	0	0	0	0	0	0
R	I	S	R	S	R	S	S	R	S	96.5	0	0	0	0	0	0	0	0	0
R	I	S	R	S	R	S	S	R	S	96.5	0	0	0	0	0	0	0	0	0
R	I	S	R	S	R	S	S	R	S	96.5	0	0	0	0	0	0	0	0	0
R	I	S	R	S	R	S	S	R	S	96.5	0	0	0	0	0	0	0	0	0
R	I	S	R	S	R	S	S	R	S	96.5	0	0	0	0	0	0	0	0	0

R	I	S	R	S	R	S	S	R	S	96.5	0	0	0	0	0	0	0	0	0
R	I	S	R	S	R	S	S	R	S	96.5	0	0	0	0	0	0	0	0	0
R	I	S	R	S	R	S	S	R	S	96.5	0	0	0	0	0	0	0	0	0
R	S	S	R	S	R	S	S	S	S	96.5	0	0	0	0	0	0	0	0	0
R	S	S	R	S	R	S	S	S	S	96.5	0	0	0	0	0	0	0	0	0
R	R	S	R	S	R	S	S	S	S	96.5	0	0	0	0	0	0	0	0	0
R	I	S	R	S	R	S	S	R	S	96.5	0	0	0	0	0	0	0	0	0
R	I	S	R	S	R	S	S	R	S	96.5	0	0	0	0	0	0	0	0	0
R	I	S	R	S	R	S	S	R	S	96.5	0	0	0	0	0	0	0	0	0
R	I	S	R	S	R	S	S	S	S	96.5	0	0	0	0	0	0	0	0	0
R	I	S	R	S	R	S	S	R	S	96.5	0	0	0	0	0	0	0	0	0
R	I	S	I	S	R	S	S	R	S	96.5	0	0	0	0	0	0	0	0	0
R	I	I	R	S	R	S	S	S	S	96.5	0	0	0	0	0	0	0	0	0
R	S	S	R	S	R	S	S	R	S	96.5	0	0	0	0	0	0	0	0	0
R	I	S	R	S	R	S	S	S	S	96.5	0	0	0	0	0	0	0	0	0
R	I	S	R	S	R	S	S	R	S	96.5	0	0	0	0	0	0	0	0	0
R	I	S	R	S	R	S	S	R	S	96.5	0	0	0	0	0	0	0	0	0
R	I	S	R	S	R	S	S	R	S	96.5	0	0	0	0	0	0	0	0	0
R	I	I	R	S	R	S	S	R	S	96.5	0	0	0	0	0	0	0	0	0
R	I	S	R	S	R	S	S	R	S	96.5	0	0	0	0	0	0	0	0	0
R	S	S	R	S	R	S	S	R	S	96.5	0	0	0	0	0	0	0	0	0
R	S	S	R	S	R	S	S	S	S	96.5	0	0	0	0	0	0	0	0	0
S	S	S	S	S	R	S	S	S	S	0	0	0	0	0	0	0	0	0	0
S	R	S	S	S	R	S	S	S	S	0	0	0	0	0	0	0	0	0	0
S	S	S	S	S	R	S	S	S	S	0	0	0	0	0	0	0	0	0	0
S	S	S	S	S	R	S	S	S	S	0	0	0	0	0	0	0	0	0	0
S	S	S	S	S	R	S	S	S	S	0	0	0	0	0	0	0	0	0	0
R	S	S	R	S	R	S	S	R	S	0	0	0	0	0	0	0	0	0	0
S	S	S	S	S	R	S	S	S	S	0	0	0	0	0	0	0	0	0	0
R	S	S	R	S	R	S	S	S	S	96.5	0	0	0	0	0	0	0	0	0
R	S	S	R	S	R	S	S	S	S	96.5	0	0	0	0	0	0	0	0	0
S	S	S	S	S	R	S	S	S	S	96.5	0	0	0	0	0	0	0	0	0
S	S	S	S	S	R	S	S	S	S	0	0	0	0	0	0	0	0	0	0
S	S	S	S	S	R	S	S	S	S	0	0	0	0	0	0	0	0	0	0
R	S	S	R	S	R	S	S	S	S	96.5	0	0	0	0	0	0	0	0	0
R	S	S	R	S	S	S	S	S	S	0	0	0	0	0	0	0	0	0	0
R	S	S	R	S	S	S	S	S	S	0	0	0	0	0	0	0	0	0	0
R	I	I	R	S	R	S	S	S	S	96.5	0	0	0	0	0	0	0	0	0
R	S	S	R	S	R	S	S	S	S	96.5	0	0	0	0	0	0	0	0	0
R	I	S	R	S	R	S	S	S	S	96.5	0	0	0	0	0	0	0	0	0
R	S	S	R	S	R	S	S	S	S	96.5	0	0	0	0	0	0	0	0	0
S	S	S	S	S	R	S	S	S	S	0	0	0	0	0	0	0	0	0	0
S	S	S	S	S	R	S	S	S	S	0	0	0	0	0	0	0	0	0	0
S	S	S	S	S	R	S	S	S	S	0	0	0	0	0	0	0	0	0	0
R	S	S	R	S	R	S	S	S	S	0	0	0	0	0	0	0	0	0	0
S	S	S	S	S	R	S	S	S	S	0	0	0	0	0	0	0	0	0	0
S	S	S	S	S	R	S	S	S	S	0	0	0	0	0	0	0	0	0	0
R	R	S	R	S	R	S	S	S	S	96.5	0	0	0	0	0	0	0	0	0
R	S	S	R	S	R	S	S	S	S	0	0	0	0	0	0	0	0	0	0
R	S	S	R	S	R	S	S	S	S	96.5	0	0	0	0	0	0	0	0	0
-	-	-	-	-	-	-	-	-	-	0	0	0	0	0	0	0	0	0	0
-	-	-	-	-	-	-	-	-	-	0	0	0	0	0	0	0	0	0	0
-	-	-	-	-	-	-	-	-	-	0	0	0	0	0	0	0	0	0	0
-	-	-	-	-	-	-	-	-	-	0	0	0	0	0	0	0	0	0	0

-	-	-	-	-	-	-	-	-	-	0	0	0	0	0	0	0	0	0	0
-	-	-	-	-	-	-	-	-	-	0	0	0	0	0	0	0	0	0	0
-	-	-	-	-	-	-	-	-	-	0	0	0	0	0	0	0	0	0	0
-	-	-	-	-	-	-	-	-	-	0	0	0	0	0	0	0	0	0	0
-	-	-	-	-	-	-	-	-	-	0	0	0	0	0	0	0	0	0	0
-	-	-	-	-	-	-	-	-	-	0	0	0	0	0	0	0	0	0	0
-	-	-	-	-	-	-	-	-	-	0	0	0	0	0	0	0	0	0	0
-	-	-	-	-	-	-	-	-	-	0	0	0	0	0	0	0	0	0	0
-	-	-	-	-	-	-	-	-	-	0	0	0	0	0	0	0	0	0	0
-	-	-	-	-	-	-	-	-	-	0	0	0	0	0	0	0	0	0	0
-	-	-	-	-	-	-	-	-	-	0	0	0	0	0	0	0	0	0	0
-	-	-	-	-	-	-	-	-	-	0	0	0	0	0	0	0	0	0	0
-	-	-	-	-	-	-	-	-	-	0	0	0	0	0	0	0	0	0	0
-	-	-	-	-	-	-	-	-	-	0	0	0	0	0	0	0	0	0	0
-	-	-	-	-	-	-	-	-	-	0	0	0	0	0	0	0	0	0	0
-	-	-	-	-	-	-	-	-	-	0	0	0	0	0	0	0	0	0	0
-	-	-	-	-	-	-	-	-	-	0	0	0	0	0	0	0	0	0	0
-	-	-	-	-	-	-	-	-	-	0	0	0	0	0	0	0	0	0	0
-	-	-	-	-	-	-	-	-	-	0	0	0	0	0	0	0	0	0	0
-	-	-	-	-	-	-	-	-	-	0	0	0	0	0	0	0	0	0	0
-	-	-	-	-	-	-	-	-	-	0	0	0	0	0	0	0	0	0	0
-	-	-	-	-	-	-	-	-	-	0	0	0	0	0	0	0	0	0	0
-	-	-	-	-	-	-	-	-	-	0	0	0	0	0	0	0	0	0	0
-	-	-	-	-	-	-	-	-	-	0	0	0	0	0	0	0	0	0	0
-	-	-	-	-	-	-	-	-	-	0	0	0	0	0	0	0	0	0	0
-	-	-	-	-	-	-	-	-	-	0	0	0	0	0	0	0	0	0	0
-	-	-	-	-	-	-	-	-	-	0	0	0	0	0	0	0	0	0	0
-	-	-	-	-	-	-	-	-	-	0	0	0	0	0	0	0	0	0	0
-	-	-	-	-	-	-	-	-	-	0	0	0	0	0	0	0	0	0	0
-	-	-	-	-	-	-	-	-	-	0	0	0	0	0	0	0	0	0	0
-	-	-	-	-	-	-	-	-	-	0	0	0	0	0	0	0	0	0	0
-	-	-	-	-	-	-	-	-	-	0	0	0	0	0	0	0	0	0	0
-	-	-	-	-	-	-	-	-	-	0	0	0	0	0	0	0	0	0	0
-	-	-	-	-	-	-	-	-	-	0	0	0	0	0	0	0	0	0	0
-	-	-	-	-	-	-	-	-	-	0	0	0	0	0	0	100	0	0	0
-	-	-	-	-	-	-	-	-	-	96.5	0	0	0	0	0	0	0	0	0
-	-	-	-	-	-	-	-	-	-	0	0	0	0	0	0	0	0	0	0
-	-	-	-	-	-	-	-	-	-	0	0	0	0	0	0	0	0	0	0
-	-	-	-	-	-	-	-	-	-	0	0	0	0	0	0	0	0	0	0
-	-	-	-	-	-	-	-	-	-	0	0	0	0	0	0	0	0	0	0
-	-	-	-	-	-	-	-	-	-	0	0	0	0	0	0	0	0	0	0
-	-	-	-	-	-	-	-	-	-	0	0	0	0	0	0	0	0	0	0
-	-	-	-	-	-	-	-	-	-	0	0	0	0	0	0	0	0	0	0
-	-	-	-	-	-	-	-	-	-	0	0	0	0	0	0	0	0	0	0
-	-	-	-	-	-	-	-	-	-	0	0	0	0	0	0	0	0	0	0
-	-	-	-	-	-	-	-	-	-	0	0	0	0	0	0	0	0	0	0
-	-	-	-	-	-	-	-	-	-	0	0	0	0	0	0	0	0	0	0
-	-	-	-	-	-	-	-	-	-	0	0	0	0	0	0	0	0	0	0
-	-	-	-	-	-	-	-	-	-	0	0	0	0	0	0	0	0	0	0
-	-	-	-	-	-	-	-	-	-	0	0	0	0	0	0	0	0	0	0
-	-	-	-	-	-	-	-	-	-	96.5	0	0	0	0	0	0	0	0	0
-	-	-	-	-	-	-	-	-	-	0	0	0	0	0	0	0	0	0	0
-	-	-	-	-	-	-	-	-	-	96.5	0	0	0	0	0	0	0	0	0

-	-	-	-	-	-	-	-	-	-	0	0	0	0	0	0	0	0	0	0
-	-	-	-	-	-	-	-	-	-	96.5	0	0	0	0	0	0	100	0	0
-	-	-	-	-	-	-	-	-	-	0	0	0	0	0	0	0	0	0	0
-	-	-	-	-	-	-	-	-	-	0	0	0	0	0	0	0	0	0	0
-	-	-	-	-	-	-	-	-	-	0	0	0	0	0	0	0	0	0	0
-	-	-	-	-	-	-	-	-	-	0	0	0	0	0	0	0	0	0	0
-	-	-	-	-	-	-	-	-	-	0	0	0	0	0	0	0	0	0	0
-	-	-	-	-	-	-	-	-	-	0	0	0	0	0	0	0	0	0	0
-	-	-	-	-	-	-	-	-	-	0	0	0	0	0	0	0	0	0	0
-	-	-	-	-	-	-	-	-	-	0	0	0	0	0	0	0	0	0	0
-	-	-	-	-	-	-	-	-	-	0	0	0	0	0	0	0	0	0	0
-	-	-	-	-	-	-	-	-	-	0	0	0	0	0	0	0	0	0	0
-	-	-	-	-	-	-	-	-	-	0	0	0	0	0	0	0	0	0	0
-	-	-	-	-	-	-	-	-	-	0	0	0	0	0	0	0	0	0	0
-	-	-	-	-	-	-	-	-	-	0	0	0	0	0	0	0	0	0	0
-	-	-	-	-	-	-	-	-	-	0	0	0	0	0	0	0	0	0	0
-	-	-	-	-	-	-	-	-	-	0	0	0	0	0	0	0	0	0	0
-	-	-	-	-	-	-	-	-	-	0	0	0	0	0	0	0	0	0	0
-	-	-	-	-	-	-	-	-	-	0	0	0	0	0	0	0	0	0	0
-	-	-	-	-	-	-	-	-	-	0	0	0	0	0	0	0	0	0	0
-	-	-	-	-	-	-	-	-	-	0	0	0	0	0	0	0	0	0	0
-	-	-	-	-	-	-	-	-	-	0	0	0	0	0	0	0	0	0	0
-	-	-	-	-	-	-	-	-	-	0	0	0	0	0	0	0	0	0	0
-	-	-	-	-	-	-	-	-	-	0	0	0	84.48	0	0	0	0	0	0
-	-	-	-	-	-	-	-	-	-	0	0	0	0	0	0	0	0	0	0
-	-	-	-	-	-	-	-	-	-	0	0	0	0	0	0	0	0	0	0
-	-	-	-	-	-	-	-	-	-	0	0	0	0	0	0	0	0	0	0
-	-	-	-	-	-	-	-	-	-	0	0	0	0	0	0	0	0	0	0
-	-	-	-	-	-	-	-	-	-	0	0	0	0	0	0	0	0	0	0
-	-	-	-	-	-	-	-	-	-	0	0	0	0	0	0	0	0	0	0
-	-	-	-	-	-	-	-	-	-	0	0	0	0	0	0	0	0	0	0
-	-	-	-	-	-	-	-	-	-	0	0	0	0	0	0	0	0	0	0
-	-	-	-	-	-	-	-	-	-	0	0	0	0	0	0	0	0	0	0
-	-	-	-	-	-	-	-	-	-	0	0	0	0	0	0	0	0	0	0
-	-	-	-	-	-	-	-	-	-	0	0	0	0	0	0	0	0	0	0
-	-	-	-	-	-	-	-	-	-	0	0	0	0	0	0	0	0	0	0
-	-	-	-	-	-	-	-	-	-	0	0	0	0	0	0	0	0	0	0
-	-	-	-	-	-	-	-	-	-	0	0	0	0	0	0	0	0	0	0
-	-	-	-	-	-	-	-	-	-	0	0	0	0	0	0	0	0	0	0
-	-	-	-	-	-	-	-	-	-	0	0	0	0	0	0	0	0	0	0
-	-	-	-	-	-	-	-	-	-	0	0	0	0	0	0	0	0	0	0
-	-	-	-	-	-	-	-	-	-	0	0	0	0	0	0	0	0	0	0
-	-	-	-	-	-	-	-	-	-	0	0	0	0	0	0	0	0	0	0
-	-	-	-	-	-	-	-	-	-	0	0	0	0	0	0	0	0	0	0
-	-	-	-	-	-	-	-	-	-	0	0	0	0	0	0	0	0	0	0
-	-	-	-	-	-	-	-	-	-	0	0	0	0	0	0	0	0	0	0
-	-	-	-	-	-	-	-	-	-	0	0	0	0	0	0	0	0	0	0
-	-	-	-	-	-	-	-	-	-	0	0	0	0	0	0	0	0	0	0
-	-	-	-	-	-	-	-	-	-	0	0	0	0	0	0	0	0	0	0
-	-	-	-	-	-	-	-	-	-	0	0	0	0	0	0	0	0	0	0
-	-	-	-	-	-	-	-	-	-	0	0	0	0	0	0	0	0	0	0
-	-	-	-	-	-	-	-	-	-	0	0	0	0	0	0	0	0	0	0
-	-	-	-	-	-	-	-	-	-	0	0	0	0	0	0	0	0	0	0
-	-	-	-	-	-	-	-	-	-	0	0	0	0	0	0	0	0	0	0

-	-	-	-	-	-	-	-	-	-	0	0	0	0	0	0	0	0	0	0
-	-	-	-	-	-	-	-	-	-	0	0	0	0	0	0	0	0	0	0
-	-	-	-	-	-	-	-	-	-	0	0	0	0	0	0	0	0	0	0
-	-	-	-	-	-	-	-	-	-	0	0	0	0	0	0	0	0	0	0
-	-	-	-	-	-	-	-	-	-	0	0	0	0	0	0	0	0	0	0
-	-	-	-	-	-	-	-	-	-	0	0	0	0	0	0	0	0	0	0
-	-	-	-	-	-	-	-	-	-	0	0	0	0	0	0	0	0	0	0
-	-	-	-	-	-	-	-	-	-	0	0	0	0	0	0	0	0	0	0
-	-	-	-	-	-	-	-	-	-	0	0	0	0	0	0	0	0	0	0
-	-	-	-	-	-	-	-	-	-	0	0	0	0	0	0	0	0	0	0
-	-	-	-	-	-	-	-	-	-	0	0	0	0	0	0	0	0	0	0
-	-	-	-	-	-	-	-	-	-	0	0	0	0	0	0	0	0	0	0
-	-	-	-	-	-	-	-	-	-	0	0	0	0	0	0	0	0	0	0
-	-	-	-	-	-	-	-	-	-	0	0	0	0	0	0	0	0	0	0
-	-	-	-	-	-	-	-	-	-	0	0	0	0	0	0	0	0	0	0
-	-	-	-	-	-	-	-	-	-	0	0	0	0	0	0	0	0	0	0
-	-	-	-	-	-	-	-	-	-	0	0	0	0	0	0	0	0	0	0
-	-	-	-	-	-	-	-	-	-	0	0	0	0	0	0	0	0	0	0
-	-	-	-	-	-	-	-	-	-	0	0	0	0	0	0	0	0	0	0
-	-	-	-	-	-	-	-	-	-	0	0	0	0	0	0	0	0	0	0
-	-	-	-	-	-	-	-	-	-	0	0	0	0	0	0	0	0	0	0
-	-	-	-	-	-	-	-	-	-	0	0	0	0	0	0	0	0	0	0
-	-	-	-	-	-	-	-	-	-	0	0	0	0	0	0	0	0	0	0
-	-	-	-	-	-	-	-	-	-	0	0	0	0	0	0	0	0	0	0
-	-	-	-	-	-	-	-	-	-	0	0	0	0	0	0	0	0	0	0
-	-	-	-	-	-	-	-	-	-	0	0	0	0	0	0	0	0	0	100
-	-	-	-	-	-	-	-	-	-	0	0	0	0	0	0	0	0	0	0
-	-	-	-	-	-	-	-	-	-	0	0	0	0	0	0	0	0	0	0
-	-	-	-	-	-	-	-	-	-	0	0	0	0	0	0	0	0	0	0
-	-	-	-	-	-	-	-	-	-	0	0	100	0	0	0	100	0	100	0
-	-	-	-	-	-	-	-	-	-	0	0	0	0	0	0	0	0	0	0
-	-	-	-	-	-	-	-	-	-	96.5	0	0	0	0	0	0	100	0	0
-	-	-	-	-	-	-	-	-	-	0	0	0	0	0	0	0	0	0	0
-	-	-	-	-	-	-	-	-	-	0	0	0	0	0	0	0	0	0	0
-	-	-	-	-	-	-	-	-	-	0	0	0	0	0	0	0	0	0	0
-	-	-	-	-	-	-	-	-	-	0	0	0	0	0	0	0	0	0	0
-	-	-	-	-	-	-	-	-	-	0	0	0	0	0	0	0	0	0	0
-	-	-	-	-	-	-	-	-	-	0	0	0	0	0	0	0	0	0	0
-	-	-	-	-	-	-	-	-	-	0	0	0	0	0	0	0	0	0	0
-	-	-	-	-	-	-	-	-	-	0	0	0	0	0	0	0	0	0	0
-	-	-	-	-	-	-	-	-	-	0	0	0	0	0	0	0	0	0	0
-	-	-	-	-	-	-	-	-	-	0	0	0	0	0	0	0	0	0	0
-	-	-	-	-	-	-	-	-	-	0	0	0	0	0	0	0	0	0	0
-	-	-	-	-	-	-	-	-	-	0	0	0	0	0	0	0	0	0	0
-	-	-	-	-	-	-	-	-	-	0	0	0	0	0	0	0	0	0	0
-	-	-	-	-	-	-	-	-	-	0	0	0	0	0	0	0	0	0	0

aadA24	aadA3	aadA5	ACT-30	ANT(2'')-Ia	aadA1	ANT(6)-Ib	APH(3')-Ia	APH(3')-Ib	strA	APH(3')-IIa	APH(3')-IVa	APH(4)-Ia	APH(6)-Ic	strB	SAT-2	CARB-21	CMY-2	CTX-M-1	CTX-M-10	CTX-M-116
0	0	100	0	0	99.62	0	0	0	99.63	0	0	0	0	99.64	100	0	0	0	0	0
0	0	0	0	0	99.62	0	0	0	99.63	0	0	0	0	99.64	100	0	0	0	0	0
0	0	0	0	0	99.62	0	0	0	99.63	0	0	0	0	99.64	100	0	0	0	0	0
0	0	0	0	0	99.62	0	0	0	99.63	0	0	0	0	99.64	100	0	0	0	0	0
0	0	0	0	0	99.62	0	0	0	100	0	0	0	0	99.64	100	0	0	0	0	0
0	0	0	0	0	0	0	0	0	0	0	0	0	0	0	0	0	0	0	0	0
0	0	0	0	0	99.62	0	0	0	100	0	0	0	0	99.64	100	0	0	0	0	0
0	0	0	0	0	99.62	0	0	0	99.63	0	0	0	0	99.64	100	0	0	0	0	0
0	0	100	0	0	99.62	0	0	0	0	0	0	0	0	0	100	0	100	0	0	0
0	0	0	0	0	99.62	0	0	0	0	0	0	0	0	0	100	0	0	0	0	0
0	0	0	0	0	99.62	0	0	0	99.63	0	0	0	0	99.64	100	0	0	0	0	0
0	0	100	0	0	99.62	0	0	0	99.63	0	0	0	0	99.64	100	0	0	0	0	0
0	0	0	0	0	99.62	0	0	0	0	0	0	0	0	0	100	0	0	0	0	0
0	0	0	0	0	99.62	0	0	0	99.63	0	0	0	0	99.64	100	0	0	0	0	0
0	0	0	0	0	99.62	0	0	0	99.63	0	0	0	0	99.64	100	0	0	0	0	0
0	0	0	0	0	99.62	0	0	0	99.63	0	0	0	0	99.64	100	0	0	0	0	0
0	0	0	0	0	99.62	0	0	0	99.63	0	0	0	0	99.64	100	0	0	0	0	0
0	0	0	0	0	99.62	0	0	0	99.63	0	0	0	0	99.64	100	0	0	0	0	0
0	0	0	0	0	99.62	0	0	0	99.63	0	0	0	0	99.64	100	0	0	0	0	0
0	0	0	0	0	99.62	0	0	0	99.63	0	0	0	0	99.64	100	0	0	0	0	0
0	0	0	0	0	99.62	0	0	0	99.63	0	0	0	0	99.64	100	0	0	0	0	0
0	0	0	0	0	99.62	0	0	0	99.63	0	0	0	0	99.64	100	0	0	0	0	0
0	0	0	0	0	99.62	0	0	0	99.63	0	0	0	0	99.64	100	0	0	0	0	0
0	0	0	0	0	99.62	0	0	0	99.63	0	0	0	0	99.64	100	0	0	0	0	0
0	0	0	0	0	99.62	0	0	0	99.63	0	0	0	0	99.64	100	0	0	0	0	0
0	0	0	0	0	99.62	0	0	0	99.63	0	0	0	0	99.64	100	0	0	0	0	0
0	0	0	0	0	99.62	0	0	0	99.63	0	0	0	0	99.64	100	0	0	0	0	0
0	0	0	0	0	99.62	0	0	0	99.63	0	0	0	0	99.64	100	0	0	0	0	0
0	0	0	0	0	99.62	0	0	0	99.63	0	0	0	0	99.64	100	0	0	0	0	0
0	0	0	0	0	99.62	0	0	0	99.63	0	0	0	0	99.64	100	0	0	0	0	0
0	0	0	0	0	99.62	0	0	0	99.63	0	0	0	0	99.64	100	0	0	0	0	0
0	0	0	0	0	99.62	0	0	0	99.63	0	0	0	0	99.64	100	0	0	0	0	0
0	0	0	0	0	99.62	0	0	0	99.63	0	0	0	0	99.64	100	0	0	0	0	0
0	0	0	0	0	99.62	0	0	0	99.63	0	0	0	0	99.64	100	0	0	0	0	0
0	0	0	0	0	99.62	0	0	0	99.63	0	0	0	0	99.64	100	0	0	0	0	0
0	0	0	0	0	99.62	0	0	0	99.63	0	0	0	0	99.64	100	0	0	0	0	0
0	0	0	0	0	99.62	0	0	0	99.63	0	0	0	0	99.64	100	0	0	0	0	0
0	0	0	0	0	99.62	0	0	0	99.63	0	0	0	0	99.64	100	0	0	0	0	0
0	0	0	0	0	99.62	0	0	0	99.63	0	0	0	0	99.64	100	0	0	0	0	0
0	0	0	0	0	99.62	0	0	0	99.63	0	0	0	0	99.64	100	0	0	0	0	0
0	0	0	0	0	99.62	0	0	0	99.63	0	0	0	0	99.64	100	0	0	0	0	0
0	0	0	0	0	99.62	0	0	0	99.63	0	0	0	0	99.64	100	0	0	0	0	0
0	0	0	0	0	99.62	0	0	0	99.63	0	0	0	0	99.64	100	0	0	0	0	0
0	0	0	0	0	99.62	0	0	0	99.63	0	0	0	0	99.64	100	0	0	0	0	0
0	0	0	0	0	99.62	0	0	0	99.63	0	0	0	0	99.64	100	0	0	0	0	0
0	0	0	0	0	99.62	0	0	0	99.63	0	0	0	0	99.64	100	0	0	0	0	0
0	0	0	0	0	99.62	0	0	0	99.63	0	0	0	0	99.64	100	0	0	0	0	0
0	0	0	0	0	99.62	0	0	0	99.63	0	0	0	0	99.64	100	0	0	0	0	0
0	0	0	0	0	99.62	0	0	0	99.63	0	0	0	0	99.64	100	0	0	0	0	0
0	0	0	0	0	99.62	0	0	0	99.63	0	0	0	0	99.64	100	0	0	0	0	0
0	0	0	0	0	99.62	0	0	0	99.63	0	0	0	0	99.64	100	0	0	0	0	0

0	0	0	0	0	99.62	0	0	0	99.63	0	0	0	0	99.64	100	0	0	0	0	0
0	0	0	0	0	99.62	0	0	0	0	0	0	0	0	0	100	0	0	0	0	0
0	0	0	0	0	99.62	0	0	0	99.63	0	0	0	0	99.64	100	0	0	0	0	0
0	0	0	0	0	99.62	0	0	0	99.63	0	0	0	0	99.64	100	0	0	0	0	0
100	0	100	0	100	100	0	0	0	99.63	0	0	0	0	99.64	100	0	0	0	0	0
0	0	100	0	0	100	0	0	0	99.63	0	0	0	0	99.64	100	0	0	0	0	0
0	0	0	0	0	99.62	0	0	0	99.63	0	0	0	0	99.64	100	0	0	0	0	0
0	0	0	0	0	99.62	0	0	0	99.63	0	0	0	0	99.64	100	0	0	0	0	0
0	0	0	0	0	99.62	0	0	0	99.63	0	0	0	0	99.64	100	0	0	0	0	0
0	0	0	0	0	99.62	0	0	0	99.63	0	0	0	0	99.64	100	0	0	0	0	0
0	0	0	0	0	99.62	0	0	0	0	0	0	0	0	0	100	0	0	0	0	0
0	0	0	0	0	0	0	0	0	0	0	0	0	0	0	0	0	0	0	0	0
0	0	0	0	0	99.62	0	0	0	99.63	0	0	0	0	99.64	100	0	0	0	0	0
0	0	0	0	0	99.62	0	0	0	99.63	0	0	0	0	99.64	100	0	0	0	0	0
0	0	0	0	0	99.62	0	0	0	99.63	0	0	0	0	99.64	100	0	0	0	0	0
0	0	0	0	0	99.62	0	0	0	99.63	0	0	0	0	99.64	100	0	0	0	0	0
0	0	0	0	0	99.62	0	0	0	99.63	0	0	0	0	99.64	100	0	0	0	0	0
0	0	0	0	0	99.62	0	0	0	99.63	0	0	0	0	99.64	100	0	0	0	0	0
0	0	0	0	0	99.62	0	0	0	99.63	0	0	0	0	99.64	100	0	0	0	0	0
0	0	0	0	0	99.62	0	0	0	99.63	0	0	0	0	99.64	100	0	0	0	0	0
0	0	0	0	0	99.62	0	0	0	0	0	0	0	0	0	99.43	0	0	0	0	0
0	0	0	0	0	99.62	0	0	0	99.63	0	0	0	0	99.64	100	0	0	0	0	0
0	0	100	0	0	99.62	0	0	0	99.63	0	0	0	0	99.64	100	0	0	0	0	0
0	0	0	0	0	99.62	0	0	0	99.63	0	0	0	0	99.64	100	0	0	0	0	0
0	0	100	0	0	99.62	0	0	0	0	0	0	0	0	0	100	0	100	0	0	0
0	0	0	0	0	99.62	0	0	0	99.63	0	0	0	0	99.64	100	0	0	0	0	0
0	0	0	0	0	99.62	0	0	0	99.63	0	0	0	0	99.64	100	0	0	0	0	0
0	0	0	0	0	99.62	0	0	0	99.63	0	0	0	0	99.64	100	0	0	0	0	0
0	0	0	0	0	99.62	0	0	0	99.63	0	0	0	0	99.64	100	0	0	0	0	0
0	0	0	0	0	99.62	0	0	0	99.63	0	0	0	0	99.64	100	0	0	0	0	0
0	0	0	0	0	99.62	0	0	0	99.63	0	0	0	0	99.64	100	0	0	0	0	0
0	0	0	0	0	99.62	0	0	0	99.63	0	0	0	0	99.64	100	0	0	0	0	0
0	0	0	0	0	99.62	0	0	0	99.63	0	0	0	0	99.64	100	0	0	0	0	0
0	0	0	0	0	99.62	0	0	0	99.63	0	0	0	0	99.64	100	0	0	0	0	0
0	0	0	0	0	99.62	0	0	0	99.63	0	0	0	0	99.64	100	0	0	0	0	0
0	0	0	0	0	99.62	0	0	0	99.63	0	0	0	0	99.64	100	0	0	0	0	0
0	0	0	0	0	99.62	0	0	0	99.63	0	0	0	0	99.64	100	0	0	0	0	0
0	0	100	0	0	99.62	0	0	0	99.63	0	0	0	0	99.64	100	0	0	0	0	0
0	0	0	0	0	99.62	0	0	0	99.63	0	0	0	0	99.64	100	0	0	0	0	0
0	0	0	0	0	99.62	0	0	0	99.63	0	0	0	0	99.64	100	0	0	0	0	0
0	0	0	0	0	99.62	0	0	0	99.63	0	0	0	0	99.64	100	0	0	0	0	0
0	0	0	0	0	99.62	0	0	0	99.63	0	0	0	0	99.64	100	0	0	0	0	0
0	0	0	0	0	99.62	0	0	0	99.63	0	0	0	0	99.64	100	0	0	0	0	0
0	0	0	0	0	99.62	0	0	0	99.63	0	0	0	0	99.64	100	0	0	0	0	0
0	0	0	0	0	99.62	0	0	0	99.63	0	0	0	0	99.64	100	0	0	0	0	0
0	0	0	0	0	99.62	0	0	0	100	0	0	0	0	99.64	100	0	0	0	0	0
0	0	0	0	0	99.62	0	0	0	99.63	0	0	0	0	99.64	100	0	0	0	0	0
0	0	0	0	0	99.62	0	0	0	99.63	0	0	0	0	99.64	100	0	0	0	0	0
0	0	0	100	0	99.62	0	0	0	99.63	0	0	0	0	99.64	100	0	0	0	0	0
0	0	0	100	0	99.62	0	0	0	99.63	0	0	0	0	99.64	100	0	0	0	0	0
0	0	0	100	0	99.62	0	0	0	99.63	0	0	0	0	99.64	100	0	0	0	0	0
0	0	0	100	0	99.62	0	0	0	99.63	0	0	0	0	99.64	100	0	0	0	0	0
0	0	0	100	0	99.62	0	0	0	99.63	0	0	0	0	99.64	100	0	0	0	0	0
0	0	0	100	0	99.62	0	0	0	99.63	0	0	0	0	99.64	100	0	0	0	100	0

0	0	0	100	0	99.62	0	0	0	99.63	0	0	0	0	99.64	100	0	0	0	0	0
0	0	0	100	0	99.62	0	0	0	99.63	0	0	0	0	99.64	100	0	0	0	0	0
0	0	0	100	0	99.62	0	0	0	99.63	0	0	0	0	99.64	100	0	0	0	0	0
0	0	0	100	0	99.62	0	0	0	100	0	0	0	0	99.64	100	0	0	0	0	0
0	0	0	100	0	99.62	0	0	0	99.63	0	0	0	0	99.64	100	0	0	0	0	0
0	0	0	100	0	99.62	0	0	0	99.63	0	0	0	0	99.64	100	0	0	0	0	0
0	0	0	100	0	99.62	0	0	0	0	0	0	0	0	0	100	0	0	0	0	0
0	0	0	100	0	99.62	0	0	0	99.63	0	0	0	0	99.64	100	0	0	0	0	0
0	0	0	0	0	0	0	0	0	0	0	0	0	0	0	0	0	0	0	0	0
0	0	0	100	0	99.62	0	0	0	99.63	0	0	0	0	99.64	100	0	0	0	0	0
0	0	0	100	0	99.62	0	0	0	99.63	0	0	0	0	99.64	100	0	0	0	0	0
0	0	0	100	0	99.62	0	0	0	99.63	0	0	0	0	99.64	100	0	0	0	0	0
0	0	100	100	0	99.62	0	0	0	99.63	0	0	0	0	99.64	100	0	100	0	0	0
0	0	0	100	0	99.62	0	0	0	99.63	0	0	0	0	99.28	100	0	0	0	0	0
0	0	0	100	0	99.62	0	0	0	0	0	0	0	0	0	100	0	0	0	0	0
0	0	0	100	0	99.62	0	0	0	99.63	0	0	0	0	99.28	100	0	0	0	0	0
0	0	0	100	0	99.62	0	0	0	0	0	0	0	0	0	100	0	0	0	0	0
0	0	0	0	0	99.62	0	0	0	99.63	0	0	0	0	99.64	100	0	0	0	0	0
0	0	0	100	0	99.62	0	0	0	99.63	0	0	0	0	99.64	100	0	0	0	0	0
0	0	0	0	0	99.62	0	0	0	99.63	0	0	0	0	99.64	100	0	0	0	0	0
0	0	0	0	0	99.62	0	0	0	99.63	0	0	0	0	99.64	100	0	0	0	0	0
0	0	0	0	0	99.62	0	0	0	99.63	0	0	0	0	99.64	100	0	0	0	0	0
0	0	0	0	0	99.62	0	0	0	99.63	0	0	0	0	99.64	100	0	0	0	0	0
0	0	0	0	0	99.62	0	0	0	99.63	0	0	0	0	99.64	100	0	0	0	0	0
0	0	0	100	0	99.62	0	0	0	99.63	0	0	0	0	99.64	100	0	0	0	0	0
0	0	0	100	0	99.62	0	0	0	0	0	0	0	0	0	100	0	0	0	0	0
0	0	0	100	0	99.62	0	0	0	99.63	0	0	0	0	99.64	100	0	0	0	0	0
0	0	0	100	0	99.62	0	0	0	99.63	0	0	0	0	99.64	100	0	0	0	0	0
0	0	0	100	0	99.62	0	0	0	99.63	0	0	0	0	99.64	100	0	0	0	0	0
0	0	0	100	0	99.62	0	0	0	99.63	0	0	0	0	99.64	100	0	0	0	0	0
0	0	0	100	0	99.62	0	0	0	99.63	0	0	0	0	99.64	100	0	0	0	0	0
0	0	0	100	0	99.62	0	0	0	99.63	0	0	0	0	99.64	100	0	0	0	0	0
0	0	0	100	0	99.62	0	0	0	99.63	0	0	0	0	99.64	100	0	0	0	0	0
0	0	0	100	0	99.62	0	0	0	99.63	0	0	0	0	99.64	100	0	0	0	0	0
0	0	0	100	0	99.62	0	0	0	99.63	0	0	0	0	99.64	100	0	0	0	0	0
0	0	0	100	0	99.62	0	0	0	99.63	0	0	0	0	99.64	100	0	0	0	0	0
0	0	0	100	0	99.62	0	0	0	100	0	0	0	0	99.64	100	0	0	0	0	0
0	0	0	100	0	99.62	0	0	0	0	0	0	0	0	0	100	0	0	0	0	0
0	0	0	100	0	99.62	0	0	0	99.63	0	0	0	0	99.64	100	0	0	0	0	0
0	0	0	100	0	99.62	0	0	0	99.63	0	0	0	0	99.64	100	0	0	0	0	0
0	0	0	100	0	99.62	0	0	0	99.63	0	0	0	0	99.64	100	0	0	0	0	0
0	0	0	100	0	99.62	0	0	0	99.63	0	0	0	0	99.64	100	0	0	0	0	0
0	0	0	100	0	99.62	0	0	0	99.63	0	0	0	0	99.64	100	0	0	0	0	0
0	0	0	100	0	99.62	0	0	0	0	0	0	0	0	0	100	0	0	0	0	0
0	0	0	100	0	99.62	0	0	0	0	0	0	0	0	0	100	0	0	0	0	0
0	0	0	100	0	99.62	0	0	0	99.63	0	0	0	0	99.64	100	0	0	0	0	0
0	0	0	100	0	99.62	0	0	0	99.63	0	0	0	0	99.64	100	0	0	0	0	0
0	0	0	100	0	99.62	0	0	0	0	0	0	0	0	0	100	0	0	0	0	0
0	0	0	100	0	99.62	0	0	0	99.63	0	0	0	0	99.64	100	0	0	0	0	0
0	0	0	100	0	99.62	0	0	0	99.63	0	0	0	0	99.64	100	0	0	0	0	0
0	0	0	100	0	99.62	0	0	0	0	0	0	0	0	0	100	0	0	0	0	0
0	0	0	100	0	99.62	0	0	0	99.25	0	0	0	0	99.64	100	0	0	0	0	0
0	0	0	100	0	99.62	0	0	0	99.63	0	0	0	0	99.64	100	0	0	0	0	0
0	0	0	100	0	99.62	0	0	0	99.63	0	0	0	0	99.64	100	0	0	0	0	0

0	0	0	100	0	99.62	0	0	0	99.63	0	0	0	0	99.64	100	0	0	0	0	0
0	0	0	100	0	99.62	0	0	0	99.63	0	0	0	0	99.64	100	0	0	0	0	0
0	0	0	100	0	99.62	0	0	0	99.63	0	0	0	0	99.64	100	0	0	0	0	0
0	0	0	100	0	99.62	0	0	0	99.63	0	0	0	0	99.64	100	0	0	0	0	0
0	0	0	100	0	99.62	0	0	0	99.63	0	0	0	0	99.64	100	0	0	0	0	0
0	0	0	0	0	0	0	0	0	99.63	0	0	0	0	99.64	0	0	0	0	0	0
0	0	0	100	0	99.62	0	0	0	99.63	0	0	0	0	99.64	100	0	0	0	0	0
0	0	0	100	0	99.62	0	0	0	99.63	0	0	0	0	99.64	100	0	0	0	0	0
0	0	0	100	0	99.62	0	0	0	0	0	0	0	0	0	100	0	0	0	0	0
0	0	0	100	0	99.62	0	0	0	99.63	0	0	0	0	99.64	100	0	0	0	0	0
0	0	0	100	0	99.62	0	0	0	99.63	0	0	0	0	99.64	100	0	0	0	0	0
0	0	0	100	0	99.62	0	0	0	99.63	0	0	0	0	99.64	100	0	0	0	0	0
0	0	0	0	0	0	0	0	0	0	0	0	0	0	0	0	0	0	0	0	0
0	0	0	100	0	99.62	0	0	0	99.63	0	0	0	0	99.64	100	0	0	0	0	0
0	0	0	100	0	99.62	0	0	0	99.63	0	0	0	0	99.64	100	0	0	0	0	0
0	0	0	100	0	99.62	0	0	0	99.63	0	0	0	0	99.64	100	0	0	0	0	0
0	0	0	100	0	99.62	0	0	0	99.63	0	0	0	0	99.64	100	0	0	0	0	0
0	0	0	100	0	99.62	0	0	0	99.63	0	0	0	0	99.64	100	0	0	0	0	0
0	0	0	100	0	99.62	0	0	0	99.63	0	0	0	0	99.64	100	0	0	0	0	0
0	0	0	100	0	99.62	0	0	0	99.63	0	0	0	0	99.64	100	0	0	0	0	0
0	0	0	100	0	99.62	0	0	0	99.63	0	0	0	0	99.64	100	0	0	0	0	0
0	0	0	100	0	99.62	0	0	0	99.63	0	0	0	0	99.64	100	0	0	0	0	0
0	0	0	100	0	99.62	0	0	0	99.63	0	0	0	0	99.64	100	0	0	0	0	0
0	0	0	100	0	99.62	0	0	0	99.63	0	0	0	0	99.64	100	0	0	0	0	0
0	0	0	100	0	99.62	0	0	0	99.63	0	0	0	0	99.64	100	0	0	0	0	0
0	0	0	100	0	99.62	0	0	0	99.63	0	0	0	0	99.64	100	0	0	0	0	0
0	0	0	100	0	99.62	0	0	0	99.63	0	0	0	0	99.64	100	0	0	0	0	0
0	0	0	100	0	99.62	0	0	0	99.63	0	0	0	0	99.64	100	0	0	0	0	0
0	0	0	100	0	99.62	0	0	0	99.63	0	0	0	0	99.64	100	0	0	0	0	0
0	0	100	0	0	99.62	0	0	0	99.63	0	0	0	0	99.64	100	0	0	0	0	0
0	0	100	0	0	99.62	0	0	0	99.63	0	0	0	0	99.64	100	0	0	0	0	0
0	0	0	100	0	99.62	0	0	0	0	0	0	0	0	0	100	0	0	0	0	0
0	0	0	0	0	0	0	0	0	100	0	0	0	0	99.64	0	0	0	0	0	0
0	0	0	100	0	99.62	0	0	0	99.63	0	0	0	0	99.64	100	0	0	0	0	0
0	0	0	100	0	99.62	0	0	0	0	0	0	0	0	0	100	0	0	0	0	0
0	0	0	0	0	99.62	0	0	0	99.63	0	0	0	0	99.64	100	0	0	0	0	0
0	0	0	100	0	99.62	0	0	0	0	0	0	0	0	0	100	0	0	0	0	0
0	0	0	100	0	99.62	0	0	0	99.63	0	0	0	0	99.64	100	0	0	0	0	0
0	0	0	100	0	99.62	0	0	0	99.63	0	0	0	0	99.64	100	0	0	0	0	0
0	0	0	100	0	99.62	0	0	0	0	0	0	0	0	0	100	0	0	0	0	0
0	0	0	100	0	99.62	0	0	0	0	0	0	0	0	0	100	0	0	0	0	0
0	0	0	100	0	99.62	0	0	0	99.63	0	0	0	0	99.64	100	0	0	0	0	0
0	0	0	100	0	99.62	0	0	0	0	0	0	0	0	0	100	0	0	0	0	0
0	0	0	100	0	99.62	0	0	0	0	0	0	0	0	0	100	0	0	0	0	0
0	0	0	100	0	99.62	0	0	0	99.63	0	0	0	0	99.64	100	0	0	0	0	0
0	0	0	100	0	99.62	0	0	0	0	0	0	0	0	0	100	0	0	0	0	0
0	0	0	100	0	99.62	0	0	0	99.63	0	0	0	0	99.64	100	0	0	0	0	0
0	0	0	100	0	99.62	0	0	0	99.63	0	0	0	0	99.64	100	0	0	0	0	0
0	0	0	100	0	99.62	0	0	0	0	0	0	0	0	0	99.43	0	0	0	0	0
0	0	0	100	0	99.62	0	0	0	99.63	0	0	0	0	99.64	99.43	0	0	0	0	0
0	0	0	100	0	99.62	0	0	0	99.63	0	0	0	0	99.64	100	0	0	0	0	0
0	0	0	100	0	99.62	0	0	0	99.63	0	0	0	0	99.64	100	0	0	0	0	0
0	0	0	0	0	99.62	0	0	0	99.63	0	0	0	0	99.64	100	0	0	0	0	0
0	0	0	100	0	99.62	0	0	0	99.63	0	0	0	0	99.64	100	0	0	0	0	0

0	0	0	100	0	99.62	0	0	0	99.63	0	0	0	0	99.64	100	0	0	0	0	0
0	0	0	100	0	99.62	0	0	0	99.63	0	0	0	0	99.64	100	0	0	0	0	0
0	0	0	100	0	99.62	0	0	0	99.63	0	0	0	0	99.64	100	0	0	0	0	0
0	0	100	100	0	99.62	0	0	0	0	0	0	0	0	0	100	0	0	0	0	0
0	0	0	100	0	99.62	0	0	0	0	0	0	0	0	0	100	0	0	0	0	0
0	0	100	100	0	99.62	0	0	0	0	0	0	0	0	0	100	0	0	0	0	0
0	0	0	100	0	99.62	0	0	0	99.63	0	0	0	0	99.64	100	0	0	0	0	0
0	0	0	100	0	99.62	0	0	0	99.63	0	0	0	0	99.64	100	0	0	0	0	0
0	0	0	100	0	99.62	0	0	0	99.63	0	0	0	0	99.64	100	0	0	0	0	0
0	0	0	100	0	99.62	0	0	0	99.63	0	0	0	0	99.64	100	0	0	0	0	0
0	0	0	100	0	99.62	0	0	0	99.63	0	0	0	0	99.64	100	0	0	0	0	0
0	0	0	100	0	99.62	0	0	0	99.63	0	0	0	0	99.64	100	0	0	0	0	0
0	0	0	100	0	99.62	0	0	0	99.63	0	0	0	0	99.64	100	0	0	0	0	0
0	0	0	100	0	99.62	0	0	0	99.63	0	0	0	0	99.64	100	0	0	0	0	0
0	0	0	0	0	0	0	0	0	0	0	0	0	0	0	0	0	0	0	0	0
0	0	0	0	0	0	0	0	0	0	0	0	0	0	0	0	0	0	0	0	0
0	0	0	0	0	99.62	0	0	0	99.63	0	0	0	0	99.64	100	0	0	0	0	0
0	0	0	100	0	99.62	0	0	0	99.63	0	0	0	0	99.64	100	0	0	0	0	0
0	0	0	100	0	99.62	0	0	0	99.63	0	0	0	0	99.64	100	0	0	0	0	0
0	0	0	100	0	99.62	0	0	0	99.63	0	0	0	0	99.64	100	0	0	0	0	0
0	0	0	100	0	99.62	0	0	0	0	0	0	0	0	0	100	0	0	0	0	0
0	0	0	100	0	99.62	0	0	0	99.63	0	0	0	0	99.64	100	0	0	0	100	0
0	0	0	100	0	99.62	0	0	0	99.63	0	0	0	0	99.64	100	0	0	0	0	0
0	0	100	0	0	99.62	0	0	0	99.63	0	0	0	0	99.64	100	0	0	0	0	0
0	0	0	0	0	99.62	0	0	0	0	0	0	0	0	0	100	0	0	0	0	0
0	0	0	0	0	99.62	0	0	0	99.63	0	0	0	0	99.64	100	0	0	0	0	0
0	0	0	0	0	99.62	0	0	0	0	0	0	0	0	0	100	0	0	0	0	0
0	0	0	0	0	99.62	0	0	0	0	0	0	0	0	0	100	0	0	0	0	0
0	0	0	0	0	99.62	0	0	0	99.63	0	0	0	0	99.64	100	0	0	0	0	0
0	0	0	0	0	99.62	0	0	0	100	0	0	0	0	99.64	100	0	0	0	0	0
0	0	0	0	0	99.62	0	0	0	0	0	0	0	0	0	100	0	0	0	0	0
0	0	0	0	0	99.62	0	0	0	99.63	0	0	0	0	99.64	100	0	0	0	0	0
0	0	0	0	0	99.62	0	0	0	99.63	0	0	0	0	99.64	100	0	0	0	0	0
0	0	0	0	0	99.62	0	0	0	0	0	0	0	0	0	100	0	0	0	0	0
0	0	0	0	0	99.62	0	0	0	99.63	0	0	0	0	99.64	100	0	0	0	0	0
0	0	100	0	0	99.62	0	0	0	99.63	0	0	0	0	99.64	100	0	0	0	0	0
0	0	0	0	0	99.62	0	0	0	99.63	0	0	0	0	99.64	100	0	0	0	0	0
0	0	0	0	0	99.62	0	0	0	99.63	0	0	0	0	99.64	100	0	0	0	0	0
0	0	100	0	0	99.62	0	0	0	0	0	0	0	0	0	100	0	0	0	0	100
0	0	0	0	0	0	0	0	0	0	0	0	0	0	0	0	0	0	0	0	0
0	0	0	0	0	99.62	0	0	0	99.63	0	0	0	0	99.64	100	0	0	0	0	0
0	0	0	0	0	99.62	0	0	0	99.63	0	0	0	0	99.64	100	0	0	0	0	100
0	0	0	0	0	99.62	0	0	0	0	0	0	0	0	0	100	0	0	0	0	0
0	0	0	0	0	99.62	0	0	0	99.63	0	0	0	0	99.64	100	0	0	0	0	0
0	0	0	0	0	0	0	0	0	0	0	0	0	0	0	0	0	0	0	0	0
0	0	0	0	0	99.62	0	0	0	99.63	0	0	0	0	99.64	100	0	0	0	0	0
0	0	0	0	0	99.62	0	0	0	99.63	0	0	0	0	99.64	100	0	0	0	0	0
0	0	0	0	0	0	0	0	0	0	0	0	0	0	0	0	0	0	0	0	0
0	0	0	0	0	99.62	0	0	0	0	0	0	0	0	0	100	0	0	0	0	0
0	0	0	0	0	99.62	0	0	0	99.63	0	0	0	0	99.64	100	0	0	0	0	0
0	0	0	0	0	99.62	0	0	0	0	0	0	0	0	0	100	0	0	0	0	0
0	0	100	0	0	99.62	0	0	0	99.63	0	0	0	0	99.64	100	0	0	0	0	100
0	0	0	0	0	99.62	0	0	0	0	0	0	0	0	0	100	0	0	0	0	0
0	0	0	0	0	99.62	0	0	0	99.63	0	0	0	0	99.64	100	0	0	0	0	0

0	0	100	0	0	99.62	0	0	0	99.63	0	0	0	0	99.64	100	0	0	0	0	0
0	0	0	0	0	99.62	0	0	0	99.63	0	0	0	0	99.64	100	0	0	0	0	0
0	0	0	0	0	99.62	0	0	0	0	0	0	0	0	0	100	0	0	0	0	0
0	0	0	0	0	99.62	0	0	0	99.63	0	0	0	0	99.64	100	0	0	0	0	0
0	0	0	0	0	99.62	0	0	0	0	0	0	0	0	0	100	0	0	0	0	0
0	0	0	0	0	99.62	0	0	0	0	0	0	0	0	0	100	0	0	0	0	0
0	0	0	0	0	99.62	0	0	0	0	0	0	0	0	0	100	0	0	0	0	0
0	0	0	0	0	99.62	0	0	0	0	0	0	0	0	0	100	0	0	0	0	0
0	0	0	0	0	99.62	0	0	0	0	0	0	0	0	0	100	0	0	0	0	0
0	0	0	0	0	99.62	0	0	0	0	0	0	0	0	0	100	0	0	0	0	0
0	0	0	0	0	99.62	0	0	0	99.63	0	0	0	0	99.64	100	0	0	0	0	0
0	0	0	0	0	99.62	0	0	0	99.63	0	0	0	0	99.64	100	0	0	0	0	0
0	0	0	0	0	99.62	0	0	0	0	0	0	0	0	0	100	0	0	0	0	0
0	0	0	0	0	99.62	0	0	0	0	0	0	0	0	0	100	0	0	0	0	0
0	0	0	0	0	99.62	0	0	0	99.63	0	0	0	0	99.64	100	0	0	0	0	0
0	0	100	0	0	99.62	0	0	0	0	0	0	0	0	0	100	0	0	0	0	0
0	0	0	0	0	99.62	0	0	0	99.63	0	0	0	0	99.64	100	0	0	0	0	0
0	0	0	0	0	99.62	0	0	0	99.63	0	0	0	0	99.64	100	0	0	0	0	0
0	0	0	0	0	99.62	0	0	0	99.63	0	0	0	0	99.64	100	0	0	0	0	0
0	0	100	0	0	99.62	0	0	0	0	0	0	0	0	0	100	0	0	0	0	0
0	0	0	0	0	99.62	0	0	0	0	0	0	0	0	0	100	0	0	0	0	0
0	0	0	0	0	99.62	0	0	0	99.63	0	0	0	0	99.64	100	0	0	0	0	0
0	0	0	0	0	99.62	0	0	0	99.63	0	0	0	0	99.64	100	0	0	0	0	0
0	0	0	0	0	99.62	0	0	0	99.63	0	0	0	0	99.64	100	0	0	0	0	0
0	0	0	0	0	99.62	0	0	0	99.63	0	0	0	0	99.64	100	0	0	0	0	0
0	0	100	0	0	99.62	0	0	0	0	0	0	0	0	0	100	0	0	0	0	0
0	0	0	0	0	99.62	0	0	0	0	0	0	0	0	0	100	0	0	0	0	0
0	0	0	0	0	99.62	0	0	0	99.63	0	0	0	0	99.64	100	0	0	0	0	0
0	0	0	0	0	99.62	0	0	0	99.63	0	0	0	0	99.64	100	0	0	0	0	0
0	0	100	0	0	99.62	0	0	0	99.63	0	0	0	0	99.64	100	0	0	0	0	0
0	0	0	0	0	99.62	0	0	0	99.63	0	0	0	0	99.64	100	0	0	0	0	0
0	0	0	0	0	99.62	0	0	0	0	0	0	0	0	0	100	0	0	0	0	0
0	0	0	0	0	99.62	0	0	0	99.63	0	0	0	0	99.64	100	0	0	0	0	0
0	0	0	0	0	99.62	0	0	0	99.63	0	0	0	0	99.64	100	0	0	0	0	0
0	0	0	0	0	99.62	0	0	0	99.63	0	0	0	0	99.64	100	0	0	0	0	0
0	0	0	0	0	99.62	0	0	0	99.63	0	0	0	0	99.64	100	0	0	0	0	0
0	0	0	0	0	99.62	0	0	0	99.63	0	0	0	0	99.64	100	0	0	0	0	0
0	0	0	0	0	99.62	0	0	0	99.63	0	0	0	0	99.64	100	0	0	0	0	0
0	0	0	0	0	99.62	0	0	0	99.63	0	0	0	0	99.64	100	0	0	0	0	0
0	0	0	0	0	99.62	0	0	0	99.63	0	0	0	0	99.64	100	0	0	0	0	0
0	0	0	0	0	99.62	0	0	0	99.63	0	0	0	0	99.64	100	0	0	0	0	0
0	0	0	0	0	99.62	0	0	0	99.63	0	0	0	0	99.64	100	0	0	0	0	0
0	0	0	0	0	99.62	0	0	0	99.63	0	0	0	0	99.64	100	0	0	0	0	0
0	0	100	0	0	99.62	0	0	0	99.63	0	0	0	0	99.64	100	0	100	0	0	0
0	0	0	0	0	99.62	0	0	0	99.63	0	0	0	0	99.64	100	0	0	0	0	0
0	0	0	0	0	99.62	0	0	0	99.63	0	0	0	0	99.64	100	0	0	0	0	0
0	0	0	0	0	99.62	0	0	0	99.63	0	0	0	0	99.64	100	0	0	0	0	0
0	0	0	0	0	99.62	0	0	0	0	0	0	0	0	0	100	0	0	0	0	0
0	0	0	0	0	99.62	0	0	0	0	0	0	0	0	0	100	0	0	0	0	0
0	0	0	0	0	99.62	0	0	0	99.63	0	0	0	0	99.64	100	0	0	0	0	0
0	0	0	0	0	99.62	0	0	0	99.63	0	0	0	0	99.64	100	0	0	0	0	0
0	0	0	0	0	99.62	0	0	0	99.63	0	0	0	0	99.64	100	0	0	0	0	0
0	0	0	0	0	99.62	0	0	0	0	0	0	0	0	0	100	0	0	0	0	0
0	0	0	0	0	99.62	0	0	0	0	0	0	0	0	0	100	0	0	0	0	0

0	0	100	0	0	99.62	0	0	0	0	0	0	0	0	0	100	0	0	0	0	0
0	0	0	0	0	99.62	0	0	0	0	0	0	0	0	0	100	0	0	0	0	0
0	0	100	0	0	99.62	0	0	0	0	0	0	0	0	0	100	0	0	0	0	0
0	0	100	0	0	99.62	0	0	0	0	0	0	0	0	0	100	0	0	0	0	0
0	0	100	0	0	99.62	0	0	0	99.63	0	0	0	0	99.64	100	0	0	0	0	0
0	0	0	0	0	0	0	0	0	0	0	0	0	0	0	0	0	0	0	0	0
0	0	0	0	0	99.62	0	0	0	99.63	0	0	0	0	99.64	100	0	0	0	0	0
0	0	100	0	0	99.62	0	0	0	0	0	0	0	0	0	100	0	0	0	0	0
0	0	100	0	0	99.62	0	0	0	0	0	0	0	0	0	100	0	0	0	0	0
0	0	100	0	0	99.62	0	0	0	0	0	0	0	0	0	100	0	0	0	0	0
0	0	100	0	0	99.62	0	0	0	0	0	0	0	0	0	100	0	0	0	0	0
0	0	0	0	0	99.62	0	0	0	99.63	0	0	0	0	99.64	100	0	0	0	0	0
0	0	0	0	0	99.62	0	0	0	99.63	0	0	0	0	99.64	100	0	0	0	0	0
0	0	0	0	0	99.62	0	0	0	99.63	0	0	0	0	99.64	100	0	0	0	0	0
0	0	0	0	0	99.62	0	0	0	99.63	0	0	0	0	99.64	100	0	0	0	0	0
0	0	0	0	0	99.62	0	0	0	0	0	0	0	0	0	100	0	0	0	0	0
0	0	0	0	0	99.62	0	0	0	99.63	0	0	0	0	99.64	100	0	0	0	0	0
0	0	0	0	0	99.62	0	0	0	99.63	0	0	0	0	99.64	100	0	0	0	0	0
0	0	0	0	0	99.62	0	0	0	0	0	0	0	0	0	100	0	0	0	0	0
0	0	0	0	0	99.62	0	0	0	0	0	0	0	0	0	100	0	0	0	0	0
0	0	0	0	0	99.62	0	0	0	0	0	0	0	0	0	100	0	0	0	0	0
0	0	100	0	0	99.62	0	0	0	0	0	0	0	0	0	100	0	0	0	0	0
0	0	0	0	0	99.62	0	0	0	99.63	0	0	0	0	99.64	100	0	0	0	0	0
0	0	100	0	0	99.62	0	0	0	0	0	0	0	0	0	100	0	0	0	0	0
0	0	0	0	0	99.62	0	0	0	99.63	0	0	0	0	99.64	100	0	0	0	0	0
0	0	0	0	0	99.62	0	0	0	99.63	0	0	0	0	99.64	100	0	0	0	0	0
0	0	100	0	0	99.62	0	0	0	0	0	0	0	0	0	100	0	100	0	0	0
0	0	100	0	0	99.62	0	0	0	0	0	0	0	0	0	100	0	100	0	0	0
0	0	0	0	0	99.62	0	0	0	0	0	0	0	0	0	100	0	0	0	0	0
0	0	0	0	0	99.62	0	0	0	99.63	0	0	0	0	99.64	100	0	0	0	0	0
0	0	0	0	0	99.62	0	0	0	0	0	0	0	0	0	100	0	0	0	0	0
0	0	0	0	0	99.62	0	0	0	0	0	0	0	0	0	100	0	0	0	0	0
0	0	0	0	0	99.62	0	0	0	99.63	0	0	0	0	99.64	100	0	0	0	0	0
0	0	0	0	0	99.62	0	0	0	99.63	0	0	0	0	99.64	100	0	0	0	0	0
0	0	0	0	0	99.62	0	0	0	0	0	0	0	0	0	100	0	0	0	0	0
0	0	0	0	0	99.62	0	0	0	0	0	0	0	0	0	100	0	0	0	0	0
0	0	0	0	0	99.62	0	0	0	0	0	0	0	0	0	100	0	0	0	0	0
0	0	0	0	0	99.62	0	0	0	0	0	0	0	0	0	100	0	0	0	0	0
0	0	0	0	0	99.62	0	0	0	0	0	0	0	0	0	100	0	0	0	0	0
0	0	0	0	0	99.62	0	0	0	0	0	0	0	0	0	100	0	0	0	0	0
0	0	0	0	0	99.62	0	0	0	0	0	0	0	0	0	100	0	0	0	0	0
0	0	0	0	0	99.62	0	0	0	0	0	0	0	0	0	100	0	0	0	0	0
0	0	0	0	0	99.62	0	0	0	99.63	0	0	0	0	99.64	100	0	0	0	0	0
0	0	0	0	0	99.62	0	0	0	0	0	0	0	0	0	100	0	0	0	0	0
0	0	0	0	0	99.62	0	0	0	0	0	0	0	0	100	100	0	0	0	0	0
0	0	0	0	0	99.62	0	0	0	99.63	0	0	0	0	99.64	100	0	0	0	0	0
0	0	0	0	0	99.62	0	0	0	0	0	0	0	0	0	100	0	0	0	0	0
100	0	0	0	0	97.83	0	0	0	99.63	0	0	0	0	99.64	100	0	100	0	0	0
0	0	0	0	0	99.62	0	0	0	99.63	0	0	0	0	100	100	0	0	0	0	0
0	0	0	0	0	99.62	0	0	0	99.63	0	0	0	0	99.64	100	0	0	0	0	0
0	0	0	0	0	99.62	0	0	0	0	0	0	0	0	0	100	0	0	0	0	0
0	0	0	0	0	99.62	0	0	0	0	0	0	0	0	0	100	0	0	0	0	0
0	0	100	0	0	99.62	0	0	0	99.63	0	0	0	0	99.64	100	0	0	0	0	0
0	0	0	0	0	99.62	0	0	0	99.63	0	0	0	0	99.64	100	0	0	0	0	0

0	0	0	0	0	99.62	0	0	0	99.63	0	0	0	0	99.64	100	0	0	0	0	0
0	0	0	0	0	99.62	0	0	0	99.63	0	0	0	0	99.64	100	0	0	0	0	0
0	0	0	0	0	99.62	0	0	0	99.63	0	0	0	0	99.64	100	0	0	0	0	0
0	0	0	0	0	99.62	0	0	0	0	0	0	0	0	0	100	0	0	0	0	0
0	0	0	0	0	99.62	0	0	0	0	0	0	0	0	0	100	0	0	0	0	0
0	0	100	0	0	99.62	0	0	0	0	0	0	0	0	0	100	0	100	0	0	0
0	0	0	0	0	99.62	0	0	0	99.63	0	0	0	0	99.64	100	0	0	0	0	0
0	0	0	0	0	99.62	0	0	0	99.63	0	0	0	0	99.64	100	0	0	0	0	0
0	0	0	0	0	99.62	0	0	0	0	0	0	0	0	0	100	0	0	0	0	0
0	0	100	0	0	99.62	0	0	0	99.63	0	0	0	0	99.64	100	0	0	0	0	0
0	0	100	0	0	99.62	0	0	0	99.63	0	0	0	0	99.64	100	0	0	0	0	0
0	0	0	0	0	99.62	0	0	0	99.63	0	0	0	0	99.64	100	0	0	0	0	100
0	0	0	0	0	99.62	0	0	0	99.63	0	0	0	0	99.64	100	0	0	0	0	0
0	0	0	0	0	99.62	0	0	0	0	0	0	0	0	0	100	0	0	0	0	0
0	0	0	0	0	99.62	0	0	0	99.63	0	0	0	0	99.64	100	0	0	0	0	0
0	0	100	0	0	99.62	0	0	0	0	0	0	0	0	0	100	0	100	0	0	0
0	0	0	0	0	99.62	0	0	0	0	0	0	0	0	0	100	0	0	0	0	0
0	0	0	0	0	99.62	0	0	0	99.63	0	0	0	0	99.64	100	0	0	0	0	0
0	0	0	0	0	99.62	0	0	0	99.63	0	0	0	0	99.64	100	0	0	0	0	0
0	0	0	0	0	99.62	0	0	0	99.63	0	0	0	0	99.64	100	0	0	0	0	0
0	0	0	0	0	99.62	0	0	0	99.63	0	0	0	0	99.64	100	0	0	0	0	0
0	0	0	0	0	99.62	0	0	0	99.63	0	0	0	0	99.64	100	0	0	0	0	0
0	0	100	0	0	99.62	0	0	0	0	0	0	0	0	0	100	0	100	0	0	0
0	0	100	0	0	99.62	0	0	0	0	0	0	0	0	0	100	0	100	0	0	0
0	0	0	0	0	99.62	0	0	0	100	0	0	0	0	99.64	100	0	0	0	0	0
0	0	100	0	0	99.62	0	0	0	0	0	0	0	0	0	100	0	100	0	0	0
0	0	0	0	0	99.62	0	0	0	99.63	0	0	0	0	99.64	100	0	0	0	0	0
0	0	0	0	0	99.62	0	0	0	99.63	0	0	0	0	99.64	100	0	0	0	0	0
0	0	0	0	0	99.62	0	0	0	99.63	0	0	0	0	99.64	100	0	0	0	0	0
0	0	0	0	0	99.62	0	0	0	99.63	0	0	0	0	99.64	100	0	0	0	0	0
0	0	0	0	0	99.62	0	0	0	99.63	0	0	0	0	99.64	100	0	0	0	0	0
0	0	0	0	0	99.62	0	0	0	99.63	0	0	0	0	99.64	100	0	0	0	0	0
0	0	0	0	0	99.62	0	0	0	99.63	0	0	0	0	99.64	100	0	0	0	0	0
0	0	0	0	0	99.62	0	0	0	99.63	0	0	0	0	99.64	100	0	0	0	0	0
0	0	0	0	0	99.62	0	0	0	99.63	0	0	0	0	99.64	100	0	0	0	0	0
0	0	0	0	0	99.62	0	0	0	99.63	0	0	0	0	99.64	100	0	0	0	0	0
0	0	0	0	0	99.62	0	0	0	99.63	0	0	0	0	99.64	100	0	0	0	0	0
0	0	0	0	0	99.62	0	0	0	99.63	0	0	0	0	99.64	100	0	0	0	0	0
0	0	0	0	0	99.62	0	0	0	99.63	0	0	0	0	99.64	100	0	0	0	0	0
0	0	100	0	0	99.62	0	0	0	99.63	0	0	0	0	99.64	100	0	0	0	0	0
0	0	100	0	0	99.62	0	0	0	99.63	0	0	0	0	99.64	100	0	0	0	0	0
0	0	100	0	0	99.62	0	0	0	99.63	0	0	0	0	99.64	100	0	0	0	0	0
0	0	100	0	0	99.62	0	0	0	99.63	0	0	0	0	99.64	100	0	0	0	0	0
0	0	100	0	0	99.62	0	0	0	99.63	0	0	0	0	99.64	100	0	0	0	0	0
0	0	100	0	0	99.62	0	0	0	99.63	0	0	0	0	99.64	100	0	0	0	0	0
0	0	100	0	0	99.62	0	0	0	99.63	0	0	0	0	99.64	100	0	0	0	0	0
0	0	100	0	0	99.62	0	0	0	99.63	0	0	0	0	99.64	100	0	0	0	0	0
0	0	0	0	0	99.62	0	0	0	0	0	0	0	0	0	100	0	0	0	0	0
0	0	0	0	0	99.62	0	0	0	0	0	0	0	0	0	100	0	0	0	0	0
0	0	99.62	0	0	99.62	0	0	0	99.63	0	0	0	0	99.64	100	0	0	0	0	0
0	0	99.62	0	0	99.62	0	0	0	99.63	0	0	0	0	99.64	100	0	0	0	0	0
0	0	99.62	0	0	99.62	0	0	0	99.63	0	0	0	0	99.64	100	0	0	0	0	0
0	0	99.62	0	0	99.62	0	0	0	99.63	0	0	0	0	99.64	100	0	0	0	0	0
0	0	99.62	0	0	99.62	0	0	0	99.63	0	0	0	0	99.64	100	0	0	0	0	0

0	0	99.62	0	0	99.62	0	0	0	99.63	0	0	0	0	99.64	100	0	0	0	0	0
0	0	99.62	0	0	99.62	0	0	0	99.63	0	0	0	0	99.64	100	0	0	0	0	0
0	0	99.62	0	0	99.62	0	0	0	99.63	0	0	0	0	99.64	100	0	0	0	0	0
0	0	99.62	0	0	99.62	0	0	0	99.63	0	0	0	0	99.64	100	0	0	0	0	0
0	0	99.62	0	0	99.62	0	0	0	99.63	0	0	0	0	99.64	100	0	0	0	0	0
0	0	99.62	0	0	99.62	0	0	0	99.63	0	0	0	0	99.64	100	0	0	0	0	0
0	0	99.62	0	0	99.62	0	0	0	99.63	0	0	0	0	99.64	100	0	0	0	0	0
0	0	99.62	0	0	99.62	0	0	0	99.63	0	0	0	0	99.64	100	0	0	0	0	0
0	0	99.62	0	0	99.62	0	0	0	99.63	0	0	0	0	99.64	100	0	0	0	0	0
0	0	99.62	0	0	99.62	0	0	0	99.63	0	0	0	0	99.64	100	0	0	0	0	0
0	0	99.62	0	0	99.62	0	0	0	99.63	0	0	0	0	99.64	100	0	0	0	0	0
0	0	99.62	0	0	99.62	0	0	0	99.63	0	0	0	0	99.64	100	0	0	0	0	0
0	0	99.62	0	0	99.62	0	0	0	99.63	0	0	0	0	99.64	100	0	0	0	0	0
0	0	99.62	0	0	99.62	0	0	0	99.63	0	0	0	0	99.64	100	0	0	0	0	0
0	0	99.62	0	95.92	99.62	0	0	0	99.63	0	0	0	0	99.64	100	0	0	0	0	0
0	0	99.62	0	0	99.62	0	0	0	99.63	0	0	0	0	99.64	100	0	0	0	0	0
0	0	99.62	0	0	99.62	0	0	0	99.63	0	0	0	0	99.64	100	0	0	0	0	0
0	0	99.62	0	0	99.62	0	0	0	99.63	0	0	0	0	99.64	100	0	0	0	0	0
0	0	99.62	0	0	99.62	0	0	0	99.63	0	0	0	0	99.64	100	0	0	0	0	0
0	0	99.62	0	0	99.62	0	0	0	99.63	0	0	0	0	99.64	100	0	0	0	0	0
0	0	99.62	0	0	99.62	0	0	0	99.63	0	0	0	0	99.64	100	0	0	0	0	0
0	0	99.62	0	0	99.62	0	0	0	99.63	0	0	0	0	99.64	100	0	0	0	0	0
0	0	99.62	0	0	99.62	0	0	0	99.63	0	0	0	0	99.64	100	0	0	0	0	0
0	0	99.62	0	0	99.62	0	0	0	99.63	0	0	0	0	99.64	100	0	0	0	0	0
0	0	99.62	0	0	99.62	0	0	0	99.63	0	0	0	0	99.64	100	0	0	0	0	0
0	0	99.62	0	0	99.62	0	0	0	99.63	0	0	0	0	99.64	100	0	0	0	0	0
0	0	99.62	0	0	99.62	0	0	0	99.63	0	0	0	0	99.64	100	0	0	0	0	0
0	0	99.62	0	0	99.62	0	0	0	99.63	0	0	0	0	99.64	100	0	0	0	0	0
0	0	99.62	0	0	99.62	0	0	0	99.63	0	0	0	0	99.64	100	0	0	0	0	0
0	0	99.62	0	0	99.62	0	0	0	99.63	0	0	0	0	99.64	100	0	0	0	0	0
0	0	99.62	0	0	99.62	0	0	0	99.63	0	0	0	0	99.64	100	0	0	0	0	0
0	0	99.62	0	0	99.62	0	0	0	99.63	0	0	0	0	99.64	100	0	0	0	0	0
0	0	99.62	0	0	99.62	0	0	0	99.63	0	0	0	0	99.64	100	0	0	0	0	0
0	0	99.62	0	0	99.62	0	0	0	99.63	0	0	0	0	99.64	100	0	0	0	0	0
0	0	99.62	0	0	99.62	0	0	0	99.63	0	0	0	0	99.64	100	0	0	0	0	0
0	0	0	0	0	99.62	0	0	0	99.63	0	0	0	0	99.64	100	0	0	0	0	0
0	0	100	0	0	99.62	0	0	0	99.63	0	0	0	0	99.64	100	0	0	0	0	0
0	0	100	0	0	99.62	0	0	0	99.61	0	0	0	0	99.64	100	0	0	0	0	0
0	0	0	0	0	99.62	0	0	0	99.63	0	0	0	0	99.64	100	0	0	0	0	0
0	0	0	0	0	99.62	0	0	0	99.63	0	0	0	0	99.64	100	0	0	0	0	0
0	0	100	0	0	99.62	0	0	0	0	0	0	0	0	0	100	0	0	0	0	0
0	0	100	0	0	99.62	0	0	0	99.63	0	0	0	0	99.64	100	0	0	0	0	0
0	0	100	0	0	99.62	0	0	0	99.63	0	0	0	0	99.64	100	0	0	0	0	0
0	0	100	0	0	99.62	0	0	0	99.63	0	0	0	0	99.64	100	0	0	0	0	0
0	0	100	0	0	99.62	0	0	0	99.63	0	0	0	0	99.64	100	0	0	0	0	0
0	0	100	0	0	99.62	0	0	0	99.63	0	0	0	0	99.64	100	0	0	0	0	0
0	0	100	0	0	99.62	0	0	0	99.63	0	0	0	0	99.64	100	0	0	0	0	0
0	0	100	0	0	99.62	0	0	0	100	0	0	0	0	99.64	100	0	0	0	0	0
0	0	100	0	0	99.62	0	0	0	0	0	0	0	0	0	100	0	0	0	0	0
0	0	100	0	0	99.62	0	0	0	99.63	0	0	0	0	99.64	100	0	0	0	0	0
0	0	100	0	0	99.62	0	0	0	99.63	0	0	0	0	99.64	100	0	0	0	0	0
0	0	100	0	0	99.62	0	0	0	99.63	0	0	0	0	99.64	100	0	0	0	0	0
0	0	100	0	0	99.62	0	0	0	99.63	0	0	0	0	99.64	100	0	0	0	0	0
0	0	100	0	0	99.62	0	0	0	99.63	0	0	0	0	99.64	100	0	0	0	0	0

0	0	100	0	0	99.62	0	0	0	99.63	0	0	0	0	99.64	100	0	0	0	0	0
0	0	100	0	0	99.62	0	0	0	99.63	0	0	0	0	99.64	100	0	0	0	0	0
0	0	100	0	0	99.62	0	0	0	0	0	0	0	0	0	100	0	0	0	0	0
0	0	100	0	0	99.62	0	0	0	99.63	0	0	0	0	99.64	100	0	0	0	0	0
0	0	100	0	0	99.62	0	0	0	99.63	0	0	0	0	99.64	100	0	0	0	0	0
0	0	100	0	0	99.62	0	0	0	99.63	0	0	0	0	99.64	100	0	0	0	0	0
0	0	0	0	0	99.62	0	0	0	99.63	0	0	0	0	99.64	100	0	0	0	0	0
0	0	0	0	0	99.62	0	0	0	99.63	0	0	0	0	99.64	100	0	0	0	0	0
0	0	0	0	0	99.62	0	0	0	99.63	0	0	0	0	99.64	100	0	0	0	0	0
0	0	100	0	0	99.62	0	0	0	99.63	0	0	0	0	99.64	100	0	0	0	0	0
0	0	100	0	0	99.62	0	0	0	99.63	0	0	0	0	99.64	100	0	0	0	0	0
0	0	100	0	0	99.62	0	0	0	99.63	0	0	0	0	99.64	100	0	0	0	0	0
0	0	100	0	0	99.62	0	0	0	99.63	0	0	0	0	99.64	100	0	0	0	0	0
0	0	100	0	0	99.62	0	0	0	99.63	0	0	0	0	99.64	100	0	0	0	0	0
0	0	100	0	0	99.62	0	0	0	99.63	0	0	0	0	99.64	100	0	0	0	0	0
0	0	100	0	0	99.62	0	0	0	99.63	0	0	0	0	99.64	100	0	0	0	0	0
0	0	100	0	0	99.62	0	0	0	99.63	0	0	0	0	99.64	100	0	0	0	0	0
0	0	100	0	0	99.62	0	0	0	99.63	0	0	0	0	99.64	100	0	0	0	0	0
0	0	100	0	0	99.62	0	0	0	99.63	0	0	0	0	99.64	100	0	0	0	0	0
0	0	100	0	0	99.62	0	0	0	99.63	0	0	0	0	99.64	100	0	0	0	0	0
0	0	100	0	0	99.62	0	0	0	99.63	0	0	0	0	99.64	100	0	0	0	0	0
0	0	100	0	0	99.62	0	0	0	99.63	0	0	0	0	99.64	100	0	0	0	0	0
0	0	100	0	0	99.62	0	0	0	99.63	0	0	0	0	99.64	100	0	0	0	0	0
0	0	100	0	0	99.62	0	0	0	99.63	0	0	0	0	99.64	100	0	0	0	0	0
0	0	100	0	0	99.62	0	0	0	99.63	0	0	0	0	99.64	100	0	0	0	0	0
0	0	100	0	0	99.62	0	0	0	99.63	0	0	0	0	99.64	100	0	0	0	0	0
0	0	100	0	0	99.62	0	0	0	99.63	0	0	0	0	99.64	100	0	0	0	0	0
0	0	100	0	0	99.62	0	0	0	99.63	0	0	0	0	99.64	100	0	0	0	0	0
0	0	100	0	0	99.62	0	0	0	99.63	0	0	0	0	99.64	100	0	0	0	0	0
0	0	100	0	0	99.62	0	0	0	99.63	0	0	0	0	99.64	100	0	0	0	0	0
0	0	100	0	0	99.62	0	0	0	99.63	0	0	0	0	99.64	100	0	0	0	0	0
0	0	100	0	0	99.62	0	0	0	99.63	0	0	0	0	99.64	100	0	0	0	0	0
0	0	100	0	0	99.62	0	0	0	99.63	0	0	0	0	99.64	100	0	0	0	0	0
0	0	100	0	0	99.62	0	0	0	99.63	0	0	0	0	99.64	100	0	0	0	0	0
0	0	100	0	0	99.62	0	0	0	99.63	0	0	0	0	99.64	100	0	0	0	0	0
0	0	99.62	0	0	99.62	0	0	0	99.63	0	0	0	0	99.64	100	0	0	0	0	0
0	0	99.62	0	0	99.62	0	0	0	99.63	0	0	0	0	99.64	100	0	0	0	0	0
0	0	99.62	0	0	99.62	0	0	0	99.63	0	0	0	0	99.64	100	0	0	0	0	0
0	0	99.62	0	0	99.62	0	0	0	99.63	0	0	0	0	99.64	100	0	0	0	0	0
0	0	99.62	0	0	99.62	0	0	0	99.63	0	0	0	0	99.64	100	0	0	0	0	0
0	0	99.62	0	0	99.62	0	0	0	0	0	0	0	0	0	100	0	0	0	0	0
0	0	99.62	0	0	99.62	0	0	0	99.63	0	0	0	0	99.64	100	0	0	0	0	0
0	0	99.62	0	0	99.62	0	0	0	99.63	0	0	0	0	99.64	100	0	0	0	0	0
0	0	99.62	0	0	99.62	0	0	0	99.63	0	0	0	0	99.64	100	0	0	0	0	0
0	0	99.62	0	0	99.62	0	0	0	99.63	0	0	0	0	99.64	100	0	0	0	0	0
0	0	99.62	0	0	99.62	0	0	0	99.63	0	0	0	0	99.64	100	0	0	0	0	0
0	0	99.62	0	0	99.62	0	0	0	99.63	0	0	0	0	99.64	100	0	0	0	0	0
0	0	99.62	0	0	99.62	0	0	0	99.63	0	0	0	0	99.64	100	0	0	0	0	0
0	0	99.62	0	0	99.62	0	0	0	99.63	0	0	0	0	99.64	100	0	0	0	0	0
0	0	99.62	0	0	99.62	0	0	0	99.63	0	0	0	0	99.64	100	0	0	0	0	0
0	0	99.62	0	0	99.62	0	0	0	99.63	0	0	0	0	99.64	100	0	0	0	0	0
0	0	99.62	0	0	99.62	0	0	0	99.63	0	0	0	0	99.64	100	0	0	0	0	0
0	0	99.62	0	0	99.62	0	0	0	99.63	0	0	0	0	99.64	100	0	0	0	0	0
0	0	99.62	0	0	99.62	0	0	0	99.63	0	0	0	0	99.64	100	0	0	0	0	0

0	0	99.62	0	0	99.62	0	0	0	99.63	0	0	0	0	99.64	100	0	0	0	0	0
0	0	99.62	0	0	99.62	0	0	0	99.63	0	0	0	0	99.64	100	0	0	0	0	0
0	0	99.62	0	0	99.62	0	0	0	99.63	0	0	0	0	99.64	100	0	0	0	0	0
0	0	0	0	0	99.62	0	0	0	99.63	0	0	0	0	99.64	100	0	0	0	0	0
0	0	0	0	0	99.62	0	0	0	99.63	0	0	0	0	99.64	100	0	0	0	0	0
0	0	0	0	0	99.62	0	0	0	99.63	0	0	0	0	99.64	100	0	0	0	0	0
0	0	0	0	0	99.62	0	0	0	99.63	0	0	0	0	99.64	100	0	0	0	0	0
0	0	0	0	0	99.62	0	0	0	99.63	0	0	0	0	99.64	100	0	0	0	0	0
0	0	0	0	0	99.62	0	0	0	99.63	0	0	0	0	99.64	100	0	0	0	0	0
0	0	0	0	0	99.62	0	0	0	99.63	0	0	0	0	99.64	100	0	0	0	0	0
0	0	0	0	0	99.62	0	0	0	99.63	0	0	0	0	99.64	100	0	0	0	0	0
0	0	0	0	0	99.62	0	0	0	99.63	0	0	0	0	99.64	100	0	0	0	0	0
0	0	0	0	0	99.62	0	0	0	99.63	0	0	0	0	99.64	100	0	0	0	0	0
0	0	0	0	0	99.62	0	0	0	99.63	0	0	0	0	99.64	100	0	0	0	0	0
0	0	0	0	0	99.62	0	0	0	99.63	0	0	0	0	99.64	100	0	0	0	0	0
0	0	0	0	0	99.62	0	0	0	99.63	0	0	0	0	99.64	100	0	0	0	0	0
0	0	0	0	0	99.62	0	0	0	99.63	0	0	0	0	99.64	100	0	0	0	0	0
0	0	0	0	0	99.62	0	0	0	99.63	0	0	0	0	99.64	100	0	0	0	0	0
0	0	0	0	0	99.62	0	0	0	99.63	0	0	0	0	99.64	100	0	0	0	0	0
0	0	0	0	0	99.62	0	0	0	99.63	0	0	0	0	99.64	100	0	0	0	0	0
0	0	0	0	0	99.62	0	0	0	99.63	0	0	0	0	99.64	100	0	0	0	0	0
0	0	0	0	0	99.62	0	0	0	99.63	0	0	0	0	99.64	100	0	0	0	0	0
0	0	0	0	0	99.62	0	0	0	99.62	0	0	0	0	99.64	100	0	0	0	0	0
0	0	0	0	0	99.62	0	0	0	100	0	0	0	0	99.64	100	0	0	0	0	0
0	0	0	0	0	99.62	0	0	0	99.63	0	0	0	0	99.64	100	0	0	0	0	0
0	0	0	0	0	99.62	0	0	0	99.63	0	0	0	0	99.64	100	0	0	0	0	0
0	0	0	0	0	99.62	0	0	0	99.63	0	0	0	0	99.64	100	0	0	0	0	0
0	0	0	0	0	99.62	0	0	0	99.63	0	0	0	0	99.64	100	0	0	0	0	0
0	0	0	0	0	99.62	0	0	0	100	0	0	0	0	99.64	100	0	0	0	0	0
0	0	0	0	0	99.62	0	0	0	99.63	0	0	0	0	99.64	100	0	0	0	0	0
0	0	0	0	0	99.62	0	0	0	99.63	0	0	0	0	99.64	100	0	0	0	0	0
0	0	0	0	0	99.62	0	0	0	99.63	0	0	0	0	99.64	100	0	0	0	0	0
0	0	0	0	0	99.62	0	0	0	99.63	0	0	0	0	99.64	100	0	0	0	0	0
0	0	0	0	0	99.62	0	0	0	99.63	0	0	0	0	99.64	100	0	0	0	0	0
0	0	0	0	0	99.62	0	0	0	99.63	0	0	0	0	99.64	100	0	0	0	0	0
0	0	0	0	0	99.62	0	0	0	0	0	0	0	0	0	100	0	0	0	0	0
0	0	0	0	0	99.62	0	0	0	99.53	0	0	0	0	99.62	100	0	0	0	0	0
0	0	0	0	0	99.62	0	0	0	99.63	0	0	0	0	99.64	100	0	0	0	0	0
0	0	0	0	0	99.62	0	0	0	99.63	0	0	0	0	99.64	100	0	0	0	0	0
0	0	100	0	0	99.62	0	0	0	99.63	0	0	0	0	99.64	100	0	100	0	0	0
0	0	0	0	0	99.62	0	0	0	99.63	0	0	0	0	99.64	100	0	0	0	0	0
0	0	0	0	0	99.62	0	0	0	99.53	0	0	0	0	99.64	100	0	0	0	0	0
0	0	0	0	0	99.62	0	0	0	99.63	0	0	0	0	99.64	100	0	0	0	0	0
0	0	0	0	0	99.62	0	0	0	99.63	0	0	0	0	99.64	100	0	0	0	0	0
0	0	0	0	0	99.62	0	0	0	99.63	0	0	0	0	99.64	100	0	0	0	0	0
0	0	0	0	0	99.62	0	0	0	99.63	0	0	0	0	99.64	100	0	0	0	0	0
0	0	0	0	0	99.62	0	0	0	99.63	0	0	0	0	99.64	98.05	0	0	0	0	0
0	0	0	0	0	99.62	0	0	0	99.63	0	0	0	0	99.64	100	0	0	0	0	0
0	0	0	0	0	99.62	0	0	0	0	0	0	0	0	0	100	0	0	0	0	0
0	0	0	0	0	99.62	0	0	0	99.63	0	0	0	0	99.64	100	0	0	0	0	0
0	0	0	0	0	99.62	0	0	0	99.63	0	0	0	0	99.64	100	0	0	0	0	0
0	0	0	0	0	99.62	0	0	0	99.63	0	0	0	0	99.64	100	0	0	0	0	0
0	0	0	0	0	99.62	0	0	0	0	0	0	0	0	0	100	0	0	0	0	0
0	0	0	0	0	99.62	0	0	0	0	0	0	0	0	0	100	0	0	0	0	0

0	0	0	0	0	99.62	0	0	0	99.63	0	0	0	0	99.64	100	0	0	0	0	0
0	0	0	0	0	99.62	0	0	0	0	0	0	0	0	0	100	0	0	0	0	0
0	0	0	0	0	99.62	0	0	0	0	0	0	0	0	0	100	0	0	0	0	0
0	0	0	0	0	99.62	0	0	0	99.63	0	0	0	0	99.64	100	0	0	0	0	0
0	0	100	0	0	99.62	0	0	0	0	0	0	0	0	0	100	0	0	0	0	0
0	0	100	0	0	99.62	0	0	0	99.63	0	0	0	0	99.64	100	0	0	0	0	0
0	0	0	0	0	99.62	0	0	0	99.63	0	0	0	0	99.64	100	0	0	0	0	0
0	0	0	0	0	99.62	0	0	0	99.63	0	0	0	0	99.64	100	0	0	0	0	0
0	0	0	0	0	99.62	0	0	0	99.63	0	0	0	0	99.64	100	0	0	0	0	0
0	0	0	0	0	99.62	0	0	0	99.63	0	0	0	0	99.64	100	0	0	0	0	0
0	0	100	0	0	99.62	0	0	0	99.63	0	0	0	0	99.64	100	0	0	0	0	0
0	0	0	0	0	99.62	0	0	0	0	0	0	0	0	0	100	0	0	0	0	0
0	0	100	0	0	99.62	0	0	0	0	0	0	0	0	0	100	0	0	0	0	0
0	0	0	0	0	99.62	0	0	0	99.63	0	0	0	0	99.64	100	0	0	0	0	0
0	0	0	0	0	99.62	0	0	0	0	0	0	0	0	0	100	0	0	0	0	0
0	0	0	0	0	99.62	0	0	0	99.63	0	0	0	0	99.64	100	0	0	0	0	0
0	0	100	0	0	99.62	0	0	0	99.63	0	0	0	0	99.64	100	0	0	0	0	0
0	0	0	0	0	99.62	0	0	0	0	0	0	0	0	0	100	0	0	0	0	0
0	0	100	0	0	99.62	0	0	0	99.63	0	0	0	0	99.64	100	0	0	0	0	0
0	0	100	0	0	99.62	0	0	0	99.63	0	0	0	0	99.64	100	0	0	0	0	0
0	0	0	0	0	99.62	0	0	0	0	0	0	0	0	0	100	0	0	0	0	0
0	0	0	0	0	99.62	0	0	0	0	0	0	0	0	0	100	0	0	0	0	0
0	0	100	0	0	99.62	0	0	0	99.63	0	0	0	0	99.64	100	0	0	0	0	0
0	0	0	0	0	99.62	0	0	0	99.63	0	0	0	0	99.64	100	0	0	0	0	0
0	0	100	0	0	99.62	0	0	0	0	0	0	0	0	0	100	0	0	0	0	0
0	0	100	0	0	99.62	0	0	0	99.63	0	0	0	0	99.64	100	0	0	0	0	0
0	0	0	0	0	99.62	0	0	0	99.63	0	0	0	0	99.64	100	0	0	0	0	0
0	0	0	0	0	99.62	0	0	0	99.63	0	0	0	0	99.64	100	0	0	0	0	0
0	0	0	0	0	99.62	0	0	0	0	0	0	0	0	0	100	0	0	0	0	0
0	0	0	0	0	99.62	0	0	0	99.63	0	0	0	0	99.64	100	0	0	0	0	0
0	0	0	0	0	99.62	0	0	0	99.63	0	0	0	0	99.64	100	0	0	0	0	0
0	0	0	0	0	99.62	0	0	0	0	0	0	0	0	0	100	0	0	0	0	0
0	0	0	0	0	99.62	0	0	0	0	0	0	0	0	0	100	0	0	0	0	0
0	0	0	0	0	99.62	0	0	0	99.25	0	0	0	0	99.64	100	0	0	100	0	0
0	0	0	100	0	100	0	0	0	100	0	0	0	0	99.64	100	0	0	100	0	0
0	0	0	100	0	99.62	0	0	0	99.25	0	0	0	0	99.64	100	0	0	0	0	0
0	0	100	100	0	99.62	0	0	0	0	0	0	0	0	0	100	0	0	0	0	0
0	0	0	100	0	99.62	0	0	0	99.63	0	0	0	0	97.22	100	0	0	0	0	0
0	0	0	100	0	99.62	0	0	0	99.63	0	0	0	0	99.64	100	0	0	0	0	0
0	0	0	100	0	99.62	0	0	0	99.63	0	0	0	0	99.64	100	0	0	0	0	0
0	0	0	100	0	99.62	0	0	0	99.63	0	0	0	0	99.64	100	0	0	0	0	0
0	0	0	100	0	99.62	0	0	0	99.63	0	0	0	0	99.64	100	0	0	0	0	0
0	0	0	100	0	99.62	0	0	0	0	0	0	0	0	0	100	0	0	0	0	0
0	0	0	100	0	99.62	0	0	0	99.54	0	0	0	0	99.64	100	0	0	0	0	0
0	0	0	100	0	99.62	0	0	0	99.63	0	0	0	0	99.64	100	0	0	0	0	0
0	0	0	100	0	99.62	0	0	0	99.63	0	0	0	0	99.64	100	0	0	0	0	0
0	0	0	100	0	99.62	0	0	0	99.63	0	0	0	0	99.64	100	0	0	0	0	0
0	0	0	100	0	99.62	0	0	0	99.63	0	0	0	0	99.64	100	0	0	0	0	0
0	0	0	100	0	99.62	0	0	0	99.63	0	0	0	0	99.64	100	0	0	0	0	0
0	0	0	100	0	99.62	0	0	0	0	0	0	0	0	0	100	0	0	0	0	0
0	0	0	0	0	0	0	0	0	99.25	0	0	0	0	99.64	100	0	0	0	0	0
0	0	100	100	0	99.62	0	0	0	99.63	0	0	0	0	99.64	100	0	0	0	0	0
0	0	0	100	0	99.62	0	0	0	99.63	0	0	0	0	99.64	100	0	0	0	0	0

0	0	0	100	0	99.62	0	0	0	99.63	0	0	0	0	99.64	100	0	0	0	0	0
0	0	0	100	0	0	0	0	0	99.23	0	0	0	0	99.64	100	0	0	0	0	0
0	0	0	0	0	0	0	0	0	0	0	0	0	0	0	0	0	0	0	0	0
0	0	0	0	0	0	0	0	0	99.25	0	0	0	0	99.64	100	0	0	0	0	0
0	0	0	0	0	99.62	0	0	0	0	0	0	0	0	0	100	0	0	0	0	0
0	0	0	0	0	99.62	0	0	0	0	0	0	0	0	0	100	0	0	0	0	0
0	0	0	100	0	99.62	0	0	0	0	0	0	0	0	0	100	0	0	0	0	0
0	0	0	100	0	99.62	0	0	0	99.63	0	0	0	0	99.64	100	0	0	0	0	0
0	0	0	0	0	99.62	0	0	0	99.63	0	0	0	0	99.59	100	0	0	0	0	0
0	0	0	0	0	0	0	0	0	0	0	0	0	0	0	0	0	0	0	0	0
0	0	0	0	0	0	0	0	0	0	0	0	0	0	0	0	0	0	0	0	0
0	0	0	0	0	99.62	0	0	0	99.63	0	0	0	0	99.64	100	0	0	0	0	0
0	0	0	0	0	99.62	0	0	0	99.63	0	0	0	0	99.64	100	0	0	0	0	0
0	0	0	100	0	0	0	0	0	0	0	0	0	0	0	0	0	0	0	0	0
0	0	0	0	0	99.62	0	0	0	0	0	0	0	0	0	100	0	0	0	0	0
0	0	0	0	0	0	0	0	0	0	0	0	0	0	0	100	0	0	0	0	0
0	0	0	0	0	99.62	0	0	0	99.63	0	0	0	0	99.64	100	0	0	0	0	0
0	0	0	0	0	0	0	0	0	0	0	0	0	0	99.59	0	0	0	0	0	0
0	0	0	0	0	0	0	0	0	99.63	0	0	0	0	99.28	0	0	0	0	0	0
0	0	0	100	0	99.62	0	0	0	99.63	0	0	0	0	99.64	100	0	0	0	0	0
0	0	0	0	0	99.62	0	0	0	99.63	0	0	0	0	99.59	100	0	0	0	0	0
0	0	0	0	0	0	0	0	0	0	0	0	0	0	0	0	0	0	0	0	0
0	0	0	0	0	0	0	0	0	0	0	0	0	0	0	0	0	0	0	0	0
0	0	0	0	0	99.61	0	0	0	0	0	0	0	0	0	0	0	0	0	0	0
0	0	0	0	0	0	0	0	0	0	0	0	0	0	0	0	0	0	0	0	0
0	0	0	0	0	0	0	0	0	0	0	0	0	0	0	0	0	0	0	0	0
0	0	0	0	0	0	0	0	0	0	0	0	0	0	0	0	0	0	0	0	0
0	0	0	0	0	0	0	0	0	0	0	0	0	0	0	0	0	0	0	0	0
0	0	0	0	0	0	42.11	0	0	100	0	36.84	0	0	99.28	0	0	0	0	0	0
0	0	0	0	0	0	0	0	0	0	0	0	0	0	0	0	100	0	0	0	0
0	0	0	0	0	0	0	0	0	0	0	0	0	0	0	0	0	0	0	0	0
0	0	0	0	0	0	0	0	0	0	0	0	0	0	0	0	0	0	0	0	0
0	0	0	0	0	99.62	0	0	0	0	0	0	0	0	0	100	0	0	0	0	0
0	0	0	0	0	0	0	0	0	0	0	0	0	0	0	0	0	0	0	0	0
0	0	0	0	0	0	0	0	0	0	0	0	0	0	0	0	0	0	0	0	0
0	0	0	0	0	0	0	0	0	0	0	0	0	0	0	0	0	0	0	0	0
0	0	0	0	0	0	0	0	0	0	0	0	0	0	0	0	0	0	0	0	0
0	0	0	100	0	0	0	0	0	0	0	0	0	0	0	0	0	0	0	0	0
0	0	0	0	0	0	0	0	0	0	0	0	0	0	0	0	0	0	0	0	0
0	0	0	100	0	0	0	0	0	0	0	0	0	0	0	0	0	0	0	0	0
0	0	0	0	0	0	0	0	0	0	0	0	0	0	0	0	0	0	0	0	0
0	0	0	0	0	0	0	0	0	0	0	0	0	0	0	0	0	0	0	0	0
0	0	0	0	0	0	0	0	0	0	0	0	0	0	0	0	0	0	0	0	0
0	0	0	0	0	0	0	0	0	0	100	0	0	100	0	0	0	0	0	0	0
0	0	0	0	0	0	0	0	0	0	0	0	0	0	0	0	0	0	0	0	0
0	0	0	0	0	0	0	0	0	99.25	0	0	0	0	99.64	100	0	0	0	0	0
0	0	0	0	0	99.62	0	0	0	99.63	0	0	0	0	99.64	100	0	0	0	0	0
0	0	0	0	0	99.62	0	0	0	99.63	0	0	0	0	99.64	100	0	0	0	0	0
0	0	0	0	0	0	0	0	0	99.25	0	0	0	0	99.64	100	0	0	0	0	0
0	0	0	0	0	0	0	0	0	0	100	0	0	100	0	0	0	0	0	0	0
0	0	0	0	0	0	0	0	0	0	0	0	0	0	0	0	0	0	0	0	0
0	0	0	100	0	99.62	0	0	0	0	0	0	0	0	0	100	0	0	0	0	0
0	0	0	0	0	99.62	0	0	0	0	0	0	0	0	0	100	0	0	0	0	0
0	0	0	0	0	99.62	0	0	0	0	0	0	0	0	0	100	0	0	0	0	0

0	0	0	0	0	0	0	0	0	99.25	0	0	0	0	99.64	100	0	0	0	0	0
0	0	0	0	0	0	0	0	0	99.25	0	0	0	0	99.64	100	0	0	0	0	0
0	0	0	0	0	0	0	0	0	99.25	0	0	0	0	99.64	100	0	0	0	0	0
0	0	0	0	0	99.62	0	0	0	99.63	0	0	0	0	99.64	100	0	0	0	0	0
0	0	0	0	0	99.62	0	0	0	0	0	0	0	0	0	100	0	0	0	0	0
0	0	0	0	0	99.62	0	0	0	0	0	0	0	0	0	100	0	0	0	0	0
0	0	0	0	0	99.62	0	0	0	0	0	0	0	0	0	100	0	0	0	0	0
0	0	0	0	0	99.62	0	0	0	99.63	0	0	0	0	99.64	100	0	0	0	0	0
0	0	0	0	0	99.62	0	0	0	0	0	0	0	0	0	100	0	0	0	0	0
0	0	0	0	0	99.62	0	0	47.06	99.25	0	0	0	0	99.28	100	0	0	0	0	0
0	0	0	0	0	99.62	0	0	0	99.63	0	0	0	0	99.64	100	0	0	0	0	0
0	0	0	0	0	99.62	0	0	0	99.63	0	0	0	0	99.64	100	0	0	0	0	0
0	0	0	0	0	99.62	0	0	0	99.63	0	0	0	0	99.64	100	0	0	0	0	0
0	0	0	0	0	99.62	0	0	0	99.63	0	0	0	0	99.64	100	0	0	0	0	0
0	0	0	0	0	99.61	0	0	0	0	0	0	0	0	0	0	0	0	0	0	0
0	0	0	0	0	0	42.11	0	0	0	0	0	0	0	0	0	0	0	0	0	0
0	0	0	0	0	0	0	0	0	0	0	0	0	0	0	0	0	0	0	0	0
0	0	0	0	0	99.61	0	0	0	0	0	0	0	0	0	0	0	0	0	0	0
0	0	0	0	0	99.61	0	0	0	0	0	0	0	0	0	0	0	0	0	0	0
0	0	0	0	0	99.61	0	0	0	0	0	0	0	0	0	0	0	0	0	0	0
0	0	0	0	0	99.62	0	0	0	0	0	0	0	0	0	100	0	0	0	0	0
0	0	0	0	0	99.62	0	0	0	0	0	0	0	0	0	100	0	0	0	0	0
0	0	0	0	0	99.61	0	0	0	0	0	0	0	0	0	0	0	0	0	0	0
0	0	0	100	0	99.62	0	0	0	99.63	0	0	0	0	99.64	100	0	0	0	0	0
0	0	0	0	0	99.62	0	0	0	99.63	0	0	0	0	99.64	100	0	0	0	0	0
0	0	0	0	0	99.62	0	98.52	0	0	0	0	0	0	0	100	0	0	0	0	0
0	0	0	0	0	0	0	0	0	99.25	0	0	0	0	99.64	100	0	0	0	0	0
0	0	0	0	0	99.62	0	0	0	99.63	0	0	0	0	99.64	100	0	0	0	0	0
0	0	0	0	0	99.61	0	0	0	0	0	0	0	0	0	0	0	0	0	0	0
0	100	0	0	0	98.41	0	98.52	0	100	0	0	100	0	99.64	100	0	0	0	0	0
0	0	0	0	0	99.62	0	0	0	99.63	0	0	0	0	99.64	100	0	0	0	0	0
0	0	0	0	0	99.62	0	98.52	0	99.63	0	0	0	0	99.64	100	0	0	0	0	0
0	0	0	0	0	0	0	0	0	0	0	0	0	0	0	100	0	0	0	0	0
0	0	0	0	0	99.62	0	0	0	0	0	0	0	0	0	100	0	0	0	0	0
0	0	0	0	0	0	0	0	0	0	0	0	0	0	0	100	0	0	0	0	0
0	0	0	0	0	0	0	0	0	0	0	0	0	0	0	100	0	0	0	0	0
0	0	0	0	0	99.62	0	0	0	99.63	0	0	0	0	99.64	100	0	0	0	0	0
0	0	0	0	0	99.62	0	0	0	99.63	0	0	0	0	99.64	100	0	0	0	0	0
0	0	0	0	0	0	0	0	0	0	0	0	0	0	0	0	0	0	0	0	0
0	0	0	0	0	0	0	0	0	100	0	0	0	0	99.6	0	0	0	0	0	0
0	0	0	0	0	0	0	0	0	0	0	0	0	0	0	0	0	0	0	0	0
0	0	0	0	0	0	0	0	0	0	0	0	0	0	0	0	0	0	0	0	0
0	0	0	0	0	99.61	0	0	0	0	0	0	0	0	0	0	0	0	0	0	0
0	0	0	0	0	99.62	0	0	0	0	0	0	0	0	0	100	0	0	0	0	0
0	0	0	0	0	99.6	0	0	0	0	0	0	0	0	0	100	0	0	0	0	0
0	0	0	0	0	99.62	0	0	0	99.63	0	0	0	0	99.64	100	0	0	0	0	0

CTX-M-117	CTX-M-123	CTX-M-132	CTX-M-134	CTX-M-139	CTX-M-14	CTX-M-144	CTX-M-15	CTX-M-157	CTX-M-158	CTX-M-19	CTX-M-24	CTX-M-27	CTX-M-28	CTX-M-3	CTX-M-34	CTX-M-35	CTX-M-42	CTX-M-53	CTX-M-54	CTX-M-55
0	0	0	0	0	100	0	0	0	0	0	0	0	0	0	0	0	0	0	0	0
0	0	0	0	0	0	0	100	0	0	0	0	0	0	0	0	0	0	0	0	0
0	0	0	0	0	0	0	0	0	0	0	0	0	0	0	0	0	0	0	0	0
0	0	0	0	0	0	0	0	0	0	0	0	0	0	0	0	0	0	0	0	0
0	0	0	0	0	0	0	0	0	0	0	0	0	0	0	0	0	0	0	0	0
0	0	0	0	0	0	0	0	0	0	0	0	0	0	0	0	0	0	0	0	0
0	0	0	0	0	0	0	0	0	0	0	0	0	0	0	0	0	0	0	0	0
0	0	0	0	0	0	0	0	0	0	0	0	0	0	0	0	0	0	0	0	0
0	0	0	0	0	0	0	0	0	0	0	0	0	0	0	0	0	0	0	0	0
0	0	0	0	0	0	0	0	0	0	0	0	0	0	0	0	0	0	0	0	0
0	0	0	0	0	0	0	0	0	0	0	0	0	0	0	0	0	0	0	0	0
0	0	0	0	0	100	0	0	0	0	0	0	0	0	0	0	0	0	0	0	0
0	0	0	0	0	0	0	0	0	0	0	0	0	0	0	0	0	0	0	0	100
0	0	0	0	0	100	0	0	0	0	0	0	0	0	0	0	0	0	0	0	0
0	0	0	0	0	100	0	0	0	0	0	0	0	0	0	0	0	0	0	0	0
0	0	0	0	0	100	0	0	0	0	0	0	0	0	0	0	0	0	0	0	0
0	0	0	0	0	100	0	0	0	0	0	0	0	0	0	0	0	0	0	0	0
0	0	0	0	0	100	0	0	0	0	0	0	0	0	0	0	0	0	0	0	0
0	0	0	0	0	100	0	0	0	0	0	0	0	0	0	0	0	0	0	0	0
0	0	0	0	0	100	0	0	0	0	0	0	0	0	0	0	0	0	0	0	0
0	0	0	0	0	100	0	0	0	0	0	0	0	0	0	0	0	0	0	0	0
0	0	0	0	0	100	0	0	0	0	0	0	0	0	0	0	0	0	0	0	0
0	0	0	0	0	100	0	0	0	0	0	0	0	0	0	0	0	0	0	0	0
0	0	0	0	0	100	0	0	0	0	0	0	0	0	0	0	0	0	0	0	0
0	0	0	0	0	100	0	0	0	0	0	0	0	0	0	0	0	0	0	0	0
0	0	0	0	0	100	0	0	0	0	0	0	0	0	0	0	0	0	0	0	0
0	0	0	0	0	100	0	0	0	0	0	0	0	0	0	0	0	0	0	0	0
0	0	0	0	0	100	0	0	0	0	0	0	0	0	0	0	0	0	0	0	0
0	0	0	0	0	100	0	0	0	0	0	0	0	0	0	0	0	0	0	0	0
0	0	0	0	0	100	0	0	0	0	0	0	0	0	0	0	0	0	0	0	0
0	0	0	0	0	100	0	0	0	0	0	0	0	0	0	0	0	0	0	0	0
0	0	0	0	0	100	0	0	0	0	0	0	0	0	0	0	0	0	0	0	0
0	0	0	0	0	100	0	0	0	0	0	0	0	0	0	0	0	0	0	0	0
0	0	0	0	0	100	0	0	0	0	0	0	0	0	0	0	0	0	0	0	0
0	0	0	0	0	100	0	0	0	0	0	0	0	0	0	0	0	0	0	0	0
0	0	0	0	0	100	0	0	0	0	0	0	0	0	0	0	0	0	0	0	0
0	0	0	0	0	100	0	0	0	0	0	0	0	0	0	0	0	0	0	0	0
0	0	0	0	0	100	0	0	0	0	0	0	0	0	0	0	0	0	0	0	0
0	0	0	0	0	100	0	0	0	0	0	0	0	0	0	0	0	0	0	0	0
0	0	0	0	0	100	0	0	0	0	0	0	0	0	0	0	0	0	0	0	0
0	0	0	0	0	100	0	0	0	0	0	0	0	0	0	0	0	0	0	0	0
0	0	0	0	0	100	0	0	0	0	0	0	0	0	0	0	0	0	0	0	0
0	0	0	0	0	100	0	0	0	0	0	0	0	0	0	0	0	0	0	0	0
0	0	0	0	0	100	0	0	0	0	0	0	0	0	0	0	0	0	0	0	0
0	0	0	0	0	100	0	0	0	0	0	0	0	0	0	0	0	0	0	0	0
0	0	0	0	0	100	0	0	0	0	0	0	0	0	0	0	0	0	0	0	0
0	0	0	0	0	100	0	0	0	0	0	0	0	0	0	0	0	0	0	0	0
0	0	0	0	0	100	0	0	0	0	0	0	0	0	0	0	0	0	0	0	0
0	0	0	0	0	100	0	0	0	0	0	0	0	0	0	0	0	0	0	0	0
0	0	0	0	0	100	0	0	0	0	0	0	0	0	0	0	0	0	0	0	0
0	0	0	0	0	100	0	0	0	0	0	0	0	0	0	0	0	0	0	0	0

0	0	0	0	0	0	0	0	0	0	0	0	0	0	100	0	0	0	0	0	0
0	0	0	0	0	0	0	100	0	0	0	0	0	0	0	0	0	0	0	0	0
0	0	0	0	0	0	0	100	0	0	0	0	0	0	0	0	0	0	0	0	0
0	0	0	0	0	0	0	0	0	0	0	0	0	0	100	0	0	0	0	0	0
0	0	0	0	0	0	0	100	0	0	0	0	0	0	0	0	0	0	0	0	0
0	0	0	0	0	100	0	0	0	0	0	0	0	0	0	0	0	0	0	0	0
0	0	0	0	0	0	0	0	0	0	0	0	0	0	0	0	0	0	0	0	0
0	0	0	0	0	100	0	0	0	0	0	0	0	0	0	0	0	0	0	0	0
0	0	0	0	0	0	0	0	0	0	0	0	0	0	0	0	0	0	0	0	0
0	0	0	0	0	0	0	0	0	0	0	0	0	0	0	0	0	0	0	0	0
0	0	0	0	0	0	0	0	0	0	0	0	0	0	0	0	0	0	0	0	0
0	0	0	0	0	0	0	0	0	0	0	0	0	0	0	0	0	0	0	0	0
0	0	0	0	0	0	0	0	0	0	0	0	0	0	0	0	0	0	0	0	0
0	0	0	0	0	0	0	0	0	0	0	0	0	0	0	0	0	0	0	0	0
0	0	0	0	0	100	0	0	0	0	0	0	0	0	0	0	0	0	0	0	0
0	0	0	0	0	0	0	0	0	0	0	0	0	0	0	0	0	0	0	0	0
0	0	0	0	0	0	0	0	0	0	0	0	0	0	0	0	0	0	0	0	0
0	0	0	0	0	0	0	0	0	0	0	0	0	0	0	0	0	0	0	0	0
0	0	0	0	0	0	0	0	0	0	0	0	0	0	0	0	0	0	0	0	0
0	0	0	0	0	0	0	0	0	0	0	0	0	0	0	0	0	0	0	0	0
0	0	0	0	0	100	0	0	0	0	0	0	0	0	0	0	0	0	0	0	0
0	0	0	0	0	0	0	0	0	0	0	0	0	0	100	0	0	0	0	0	0
0	0	0	0	0	0	0	0	0	0	100	0	0	0	0	0	0	0	0	0	0
0	0	0	0	0	0	0	0	0	0	0	0	0	0	0	0	0	0	0	0	0
0	0	0	0	0	0	0	0	0	0	0	0	0	0	0	0	0	0	0	0	0
0	0	0	0	0	0	0	0	0	0	0	0	0	0	100	0	0	0	0	0	0
0	0	0	0	0	0	0	100	0	0	0	0	0	0	0	0	0	0	0	0	0
0	0	0	0	0	100	0	0	0	0	0	0	0	0	0	0	0	0	0	0	0
0	0	0	0	0	0	0	0	0	0	0	0	0	0	0	0	0	0	0	0	0
0	0	0	0	0	0	0	0	0	0	0	0	0	0	0	0	0	0	0	0	0
0	0	0	0	0	0	0	0	0	0	0	0	0	0	0	0	0	0	0	0	0
0	0	0	0	0	0	0	0	0	0	0	0	0	0	0	0	0	0	0	0	0
0	0	0	0	0	100	0	0	0	0	0	0	0	0	0	0	0	0	0	0	0
0	0	0	0	0	100	0	0	0	0	0	0	0	0	0	0	0	0	0	0	0
0	0	0	0	0	100	0	0	0	0	0	0	0	0	0	0	0	0	0	0	0
0	0	0	0	0	0	0	0	0	0	0	0	0	0	0	0	0	0	0	0	0
0	0	0	0	0	100	0	0	0	0	0	0	0	0	0	0	0	0	0	0	0
0	0	0	0	0	0	0	100	0	0	0	0	0	0	0	0	0	0	0	0	0
0	0	0	0	0	100	0	0	0	0	0	0	0	0	0	0	0	0	0	0	0
0	0	0	0	0	100	0	0	0	0	0	0	0	0	0	0	0	0	0	0	0
0	0	0	0	0	100	0	0	0	0	0	0	0	0	0	0	0	0	0	0	0
0	0	0	0	0	0	0	0	0	0	0	0	0	0	0	0	0	0	0	0	0
0	0	0	0	0	0	0	0	0	0	0	0	0	0	0	0	0	0	0	0	100
0	0	0	0	0	0	0	100	0	0	0	0	0	0	0	0	0	0	0	0	0
0	0	0	0	0	0	0	0	0	0	0	0	0	0	0	0	0	0	0	0	100
0	0	0	0	0	100	0	0	0	0	0	0	0	0	0	0	0	0	0	0	0
0	0	0	0	0	100	0	0	0	0	0	0	0	0	0	0	0	0	0	0	0
0	0	0	0	0	0	0	0	0	0	0	0	0	0	0	0	0	0	0	0	100
0	0	0	0	0	0	0	0	0	0	0	0	0	0	0	0	0	0	0	0	0
0	0	0	0	0	0	0	0	0	0	0	0	0	0	0	0	0	0	0	0	0
0	0	0	0	0	0	0	0	0	0	0	0	0	0	0	0	0	0	0	0	0
0	0	0	0	0	0	0	0	0	0	0	0	0	0	0	0	0	0	0	0	0
0	0	0	0	0	0	0	0	0	0	0	0	0	0	0	0	0	0	0	0	0
0	0	0	0	100	0	0	0	100	100	0	0	0	0	0	0	0	0	0	0	0

0	0	0	0	0	0	0	0	0	0	0	0	0	0	0	0	0	0	0	0	0
0	0	0	0	0	100	0	0	0	0	0	0	0	0	0	0	0	0	0	0	0
0	0	0	0	0	0	0	0	0	0	0	0	0	0	0	0	0	0	0	0	0
0	0	0	0	0	100	0	0	0	0	0	0	0	0	0	0	0	0	0	0	0
0	0	0	0	0	0	0	0	0	0	0	0	0	0	0	0	0	0	0	0	0
0	0	0	0	0	0	0	0	0	0	0	0	0	0	0	0	0	0	0	0	0
0	0	0	0	0	100	0	0	0	0	0	0	0	0	0	0	0	0	0	0	0
0	0	0	0	0	100	0	0	0	0	0	0	0	0	0	0	0	0	0	0	0
0	0	0	0	0	100	0	0	0	0	0	0	0	0	0	0	0	0	0	0	0
0	0	0	0	0	0	0	100	0	0	0	0	0	0	0	0	0	0	0	0	0
0	0	0	0	0	0	0	0	0	0	0	0	0	0	0	0	0	0	0	0	0
0	0	0	0	0	100	0	0	0	0	0	0	0	0	0	0	0	0	0	0	0
0	0	0	0	0	0	0	0	0	0	0	0	0	0	0	0	0	0	0	0	0
0	0	0	0	0	0	0	0	0	0	0	0	0	0	0	0	0	0	0	0	100
0	0	0	0	0	0	0	0	0	0	0	0	0	0	0	0	0	0	0	0	100
0	0	0	0	0	0	0	0	0	0	0	0	0	0	0	0	0	0	0	0	100
0	0	0	0	0	0	0	0	0	0	0	0	0	0	0	0	0	0	0	0	0
0	0	0	0	0	0	0	0	0	0	0	0	0	0	0	0	0	0	0	0	0
0	0	0	0	0	0	0	0	0	0	0	0	0	0	0	0	0	0	0	0	0
0	0	0	0	0	0	0	0	0	0	0	0	0	0	0	0	0	0	0	0	100
0	0	0	0	0	0	0	0	0	0	0	0	0	0	0	0	0	0	0	0	100
0	0	0	0	0	0	0	0	0	0	0	0	0	0	0	0	0	0	0	0	100
0	0	0	0	0	0	0	0	0	0	0	0	0	0	0	0	0	0	0	0	100
0	0	0	0	0	0	0	0	0	0	0	0	0	0	0	0	0	0	0	0	0
0	0	0	0	0	0	0	0	0	0	0	0	0	0	0	0	0	0	0	0	0
0	0	0	0	0	0	0	0	0	0	0	0	0	0	100	0	0	0	0	0	0
0	0	0	0	0	0	0	0	0	0	0	0	0	0	0	0	0	0	0	0	0
0	0	0	0	0	0	0	0	0	0	0	0	0	0	0	0	0	0	0	0	0
0	0	0	0	0	0	0	0	0	0	0	0	0	0	0	0	0	0	0	0	100
0	0	0	0	0	0	0	0	0	0	0	0	0	0	0	0	0	0	0	0	0
0	0	0	0	0	0	0	100	0	0	0	0	0	0	0	0	0	0	0	0	0
0	0	0	0	0	100	0	0	0	0	0	0	0	0	0	0	0	0	0	0	0
0	0	0	0	0	0	0	0	0	0	0	0	0	0	0	0	0	0	0	0	0
0	0	0	0	0	0	0	0	0	0	0	0	0	0	0	0	0	0	0	0	0
0	0	0	0	0	0	0	0	0	0	0	0	0	0	0	0	0	0	0	0	0
0	0	0	0	0	100	0	0	0	0	0	0	0	0	0	0	0	0	0	0	0
0	0	0	0	0	100	0	0	0	0	0	0	0	0	0	0	0	0	0	0	0
0	0	0	0	0	0	0	0	0	0	0	0	0	0	0	0	0	0	0	0	0
0	0	0	0	0	0	0	100	0	0	0	0	0	0	0	0	0	0	0	0	0
0	0	0	0	0	0	0	0	0	0	0	0	0	0	0	0	0	0	0	0	0
0	0	0	0	0	0	0	0	0	0	0	0	0	0	0	0	0	0	0	0	100
0	0	0	0	0	0	0	0	0	0	0	0	0	0	0	0	0	0	0	0	100
0	0	0	0	0	0	0	0	0	0	0	0	0	0	0	0	0	0	0	0	0
0	0	0	0	0	0	0	0	0	0	0	0	0	0	0	0	0	0	0	0	0
0	0	0	0	0	100	0	0	0	0	0	0	0	0	0	0	0	0	0	0	0
0	0	0	0	0	100	0	0	0	0	0	0	0	0	0	0	0	0	0	0	0
0	0	0	0	0	0	0	0	0	0	0	0	0	0	0	0	0	0	0	0	0
0	0	0	0	0	0	0	0	0	0	0	0	0	0	0	0	0	0	0	0	100
0	0	0	0	0	0	0	0	0	0	0	0	0	0	0	0	0	0	0	0	0
0	0	0	0	0	0	0	0	0	0	0	0	0	0	0	0	0	0	0	0	0
0	0	0	0	0	0	0	0	0	0	0	0	0	0	0	0	0	0	0	0	100
0	0	0	0	0	0	0	0	0	0	0	0	0	0	0	0	0	0	0	0	0
0	0	0	0	0	100	0	0	0	0	0	0	0	0	0	0	0	0	0	0	0
0	0	0	0	0	0	0	0	0	0	0	0	0	0	0	0	0	0	0	0	100

0	0	0	0	0	0	0	0	0	0	0	0	0	0	0	0	0	0	0	0	0
0	0	0	0	0	0	0	0	0	0	0	0	0	0	0	0	0	0	0	0	0
0	0	0	0	0	0	0	0	0	0	0	0	0	0	0	0	0	0	0	0	0
0	0	0	0	0	0	0	0	0	0	0	0	0	0	0	0	0	0	0	0	100
0	0	0	0	0	100	0	0	0	0	0	0	0	0	0	0	0	0	0	0	0
0	0	0	0	0	0	0	0	0	0	0	0	0	0	0	0	0	0	0	0	0
0	0	0	0	0	0	0	100	0	0	0	0	0	0	0	0	0	0	0	0	0
0	0	0	0	0	0	0	0	0	0	0	0	0	0	0	0	0	0	0	0	0
0	0	0	0	0	0	0	0	0	0	0	0	0	0	0	0	0	0	0	0	0
0	0	0	0	0	0	0	0	0	0	0	0	100	0	0	0	0	0	0	0	0
0	0	0	0	0	100	0	0	0	0	0	0	0	0	0	0	0	0	0	0	0
0	0	0	0	0	0	0	0	0	0	0	0	0	0	0	0	0	0	0	0	0
0	0	0	0	0	100	0	0	0	0	0	0	0	0	0	0	0	0	0	0	0
0	0	0	0	0	0	0	0	0	0	0	0	0	0	0	0	0	0	0	0	0
0	0	0	0	0	0	0	0	0	0	0	0	0	0	0	0	0	0	0	0	0
0	0	0	0	0	0	0	0	0	0	0	0	0	0	0	0	0	0	0	0	100
0	0	0	0	0	100	0	0	0	0	0	0	0	0	0	0	0	0	0	0	0
0	0	0	0	0	0	0	0	0	0	0	0	0	0	0	0	0	0	0	0	0
0	0	0	0	0	0	0	0	0	0	0	0	0	0	0	0	0	0	0	0	100
0	0	0	0	0	0	0	0	0	0	0	0	0	0	0	0	0	0	0	0	100
0	0	0	0	0	0	0	0	0	0	0	0	0	0	0	0	0	0	0	0	0
0	0	0	0	0	100	0	0	0	0	0	0	0	0	0	0	0	0	0	0	0
0	0	0	0	0	100	0	0	0	0	0	0	0	0	0	0	0	0	0	0	0
0	0	0	0	0	0	0	0	0	0	0	0	0	0	0	0	0	0	0	0	0
0	0	0	0	0	0	0	100	0	0	0	0	0	0	0	0	0	0	0	0	0
0	0	0	0	0	0	0	0	0	0	0	0	0	0	0	0	0	0	0	0	0
0	0	0	0	0	100	0	0	0	0	0	0	0	0	0	0	0	0	0	0	0
0	0	0	0	0	0	0	0	0	0	0	0	0	0	0	0	0	0	0	0	100
0	0	0	0	0	100	0	0	0	0	0	0	0	0	0	0	0	0	0	0	0
0	0	0	0	0	100	0	0	0	0	0	0	0	0	100	0	0	0	0	0	0
0	0	0	0	0	0	0	0	0	0	0	0	0	0	100	0	0	0	0	0	0
0	0	0	0	0	0	0	0	0	0	0	0	0	0	0	0	0	0	0	0	0
0	0	0	0	0	0	0	0	0	0	0	0	0	0	0	0	0	0	0	0	0
0	0	0	0	0	0	0	0	0	0	0	0	0	0	0	0	0	0	0	0	0
0	0	0	0	0	100	0	0	0	0	0	0	0	0	0	0	0	0	0	0	0
0	0	0	0	0	100	0	0	0	0	0	0	0	0	0	0	0	0	0	0	0
0	0	0	0	0	0	0	0	0	0	0	0	0	0	0	0	0	0	0	0	0
0	0	0	0	0	0	0	0	0	0	0	0	0	0	0	0	0	0	0	0	0
0	0	0	0	0	0	0	100	0	0	0	0	0	0	0	0	0	0	0	0	0
0	0	0	0	0	0	0	0	0	0	0	0	0	0	0	0	0	0	0	0	100
0	0	0	0	0	100	0	100	0	0	0	0	0	0	0	0	0	0	0	0	0
0	0	0	0	0	0	0	0	0	0	0	0	0	0	100	0	0	0	0	0	0
0	0	0	0	0	0	0	0	0	0	0	0	0	0	0	0	0	0	0	0	0
0	0	0	0	0	100	0	0	0	0	0	0	0	0	0	0	0	0	0	0	0
0	0	0	0	0	0	0	100	0	0	0	0	0	0	0	0	0	0	0	0	0
0	0	0	0	0	100	0	0	0	0	0	0	0	0	0	0	0	0	0	0	0
0	0	0	0	0	0	0	0	0	0	0	0	0	0	0	0	0	0	0	0	100
0	0	0	0	0	100	0	0	0	0	0	0	0	0	0	0	0	0	0	0	100
0	0	0	0	0	100	0	0	0	0	0	0	0	0	0	0	0	0	0	0	0
0	0	0	0	0	0	0	0	0	0	0	0	0	0	0	0	0	0	0	0	0
0	0	0	0	0	0	0	0	0	0	0	0	0	0	0	0	0	0	0	0	0
0	0	0	0	0	0	0	100	0	0	0	0	0	0	0	0	0	0	0	0	0
0	0	0	0	0	100	0	0	0	0	0	0	0	0	0	0	0	0	0	0	0
0	0	0	0	0	0	0	100	0	0	0	0	0	0	0	0	0	0	0	0	0

0	0	0	0	0	0	0	100	0	0	0	0	0	0	0	0	0	0	0	0	0
0	0	0	0	0	0	0	0	0	0	0	0	0	0	0	0	0	0	0	0	0
0	0	0	0	0	0	0	100	0	0	0	0	0	0	0	0	0	0	0	0	0
0	0	0	0	0	100	0	0	0	0	0	0	0	0	0	0	0	0	0	0	100
0	0	0	0	0	0	0	0	0	0	0	0	0	0	0	0	0	0	0	0	0
0	0	0	0	0	100	0	0	0	0	0	0	0	0	0	0	0	0	0	0	0
0	0	0	0	0	0	0	0	0	0	0	0	0	0	0	0	0	0	0	0	0
0	0	0	0	0	0	0	0	0	0	0	0	0	0	0	0	0	0	0	0	0
0	0	0	0	0	0	0	0	0	0	0	0	0	0	0	0	0	0	0	0	0
0	0	0	0	0	0	0	0	0	0	0	0	0	0	0	0	0	0	0	0	0
0	0	0	0	0	0	0	0	0	0	0	0	0	0	0	0	0	0	0	0	0
0	0	0	0	0	0	0	0	0	0	0	0	0	0	0	0	0	0	0	0	100
0	0	0	0	0	0	0	0	0	0	0	0	0	0	0	0	0	0	0	0	0
0	0	0	0	0	0	0	0	0	0	0	0	0	0	0	0	0	0	0	0	0
0	0	0	0	0	0	0	0	0	0	0	0	0	0	0	0	0	0	0	0	100
0	0	0	0	0	0	0	0	0	0	0	0	0	0	0	0	0	0	0	0	100
0	0	0	0	0	0	0	0	0	0	0	0	0	0	0	0	0	0	0	0	0
0	0	0	0	0	0	0	100	0	0	0	0	0	0	0	0	0	0	0	0	0
0	0	0	0	0	0	0	0	0	0	0	0	0	0	0	0	0	0	0	0	0
0	0	0	0	0	0	0	0	0	0	0	0	0	0	0	0	0	0	0	0	0
0	0	0	0	0	0	0	0	0	0	0	0	0	0	0	0	0	0	0	0	0
0	0	0	0	0	0	0	0	0	0	0	0	0	0	0	0	0	0	0	0	100
0	0	0	0	0	0	0	0	0	0	0	0	0	0	0	0	0	0	0	0	0
0	0	0	0	0	0	0	0	0	0	0	0	0	0	0	0	0	0	0	0	100
0	0	0	0	0	100	0	0	0	0	0	0	0	0	0	0	0	0	0	0	0
0	0	0	0	0	100	0	0	0	0	0	0	0	0	0	0	0	0	0	0	0
0	0	0	0	0	0	0	100	0	0	0	0	0	0	0	0	0	0	0	0	0
0	0	0	100	0	0	0	0	0	0	0	0	0	0	0	0	0	0	0	0	0
0	0	0	0	0	0	0	0	0	0	0	0	0	0	0	0	0	0	0	0	0
0	0	0	0	0	0	0	0	0	0	0	0	0	0	0	0	0	0	0	0	0
0	0	0	0	0	100	0	0	0	0	0	0	0	0	0	0	0	0	0	0	0
0	0	0	0	0	0	0	0	0	0	0	0	0	0	100	0	0	0	0	0	0
0	0	0	0	0	100	0	0	0	0	0	0	0	0	0	0	0	0	0	0	0
0	0	0	0	0	100	0	0	0	0	0	0	0	0	0	0	0	0	0	0	0
0	0	0	0	0	100	0	0	0	0	0	0	0	0	0	0	0	0	0	0	0
0	0	0	0	0	100	0	0	0	0	0	0	0	0	0	0	0	0	0	0	0
0	0	0	0	0	100	0	0	0	0	0	0	0	0	0	0	0	0	0	0	0
0	0	0	0	0	0	0	0	0	0	0	0	0	0	100	0	0	0	0	0	0
0	0	0	0	100	0	0	0	0	100	0	0	0	0	0	0	0	100	100	100	0
0	0	0	0	0	100	0	0	0	0	0	0	0	0	0	0	0	0	0	0	0
0	0	0	0	0	0	0	100	0	0	0	0	0	0	0	0	0	0	0	0	0
100	0	0	0	0	0	0	100	0	0	0	0	0	100	0	0	0	0	0	0	0
0	0	0	0	0	100	0	0	0	0	0	0	0	0	0	0	0	0	0	0	0
0	0	0	0	0	0	0	100	0	0	0	0	0	0	0	0	0	0	0	0	0
0	0	0	0	0	100	0	0	0	0	0	0	0	0	0	0	0	0	0	0	0
0	0	0	0	0	0	0	100	0	0	0	0	0	0	0	0	0	0	0	0	0
0	0	0	0	0	0	0	0	0	0	0	0	0	0	0	0	0	0	0	0	0
0	0	0	0	0	100	0	0	0	0	0	0	0	0	0	0	0	0	0	0	0
0	0	0	0	0	0	0	0	0	0	0	0	0	0	0	0	0	0	0	0	0
0	0	0	0	0	100	0	0	0	0	0	0	0	0	0	0	0	0	0	0	0
0	0	0	0	0	100	0	0	0	0	0	0	0	0	0	0	0	0	0	0	0
0	0	0	0	100	0	0	0	0	100	0	0	0	0	0	0	0	0	0	0	0
0	0	0	0	0	100	0	0	0	0	0	0	0	0	0	0	0	0	0	0	0
0	0	0	0	0	0	0	0	0	0	0	100	0	0	0	0	0	0	0	0	0

0	0	0	0	0	100	0	0	0	0	0	0	0	0	0	0	0	0	0	0	0
0	0	0	0	0	0	0	0	0	0	0	0	0	0	0	0	0	0	0	0	100
0	0	0	0	0	0	0	0	0	0	0	0	0	0	0	0	0	0	0	0	0
0	0	0	0	0	100	0	0	0	0	0	0	0	0	0	0	0	0	0	0	0
0	0	0	0	0	100	0	0	0	0	0	0	0	0	0	0	0	0	0	0	0
0	0	0	0	0	100	0	0	0	0	0	0	0	0	0	0	0	0	0	0	0
0	0	0	0	0	100	0	0	0	0	0	0	0	0	0	0	0	0	0	0	0
0	0	0	0	0	100	0	0	0	0	0	0	0	0	0	0	0	0	0	0	0
0	0	0	0	0	100	0	0	0	0	0	0	0	0	0	0	0	0	0	0	0
0	0	0	0	0	0	0	0	0	0	0	0	0	0	0	0	0	0	0	0	100
0	0	0	0	0	100	0	0	0	0	0	0	0	0	0	0	0	0	0	0	0
0	0	0	0	0	100	0	0	0	0	0	0	0	0	0	0	0	0	0	0	0
0	0	0	0	0	100	0	0	0	0	0	0	0	0	0	0	0	0	0	0	0
0	0	0	0	0	100	0	0	0	0	0	0	0	0	0	0	0	0	0	0	0
0	0	0	0	0	100	0	0	0	0	0	0	0	0	0	0	0	0	0	0	0
0	0	0	0	0	100	0	0	0	0	0	0	0	0	0	0	0	0	0	0	0
0	0	0	0	0	100	0	0	0	0	0	0	0	0	0	0	0	0	0	0	0
0	0	0	0	0	100	0	0	0	0	0	0	0	0	0	0	0	0	0	0	0
0	0	0	0	0	100	0	0	0	0	0	0	0	0	0	0	0	0	0	0	0
0	0	0	0	0	100	0	0	0	0	0	0	0	0	0	0	0	0	0	0	0
0	0	0	0	0	100	0	0	0	0	0	0	0	0	0	0	0	0	0	0	0
0	0	0	0	0	100	0	0	0	0	0	0	0	0	0	0	0	0	0	0	0
0	0	0	0	0	100	0	0	0	0	0	0	0	0	0	0	0	0	0	0	0
0	0	0	0	0	100	0	0	0	0	0	0	0	0	0	0	0	0	0	0	0
0	0	0	0	0	0	0	0	0	0	0	100	0	0	0	0	0	0	0	0	0
0	0	0	0	0	100	0	0	0	0	0	0	0	0	0	0	0	0	0	0	0
0	0	0	0	0	0	0	0	0	0	0	0	0	0	0	0	0	0	0	0	100
0	0	0	0	0	0	0	0	0	0	0	0	0	0	0	0	0	0	0	0	0
0	0	0	0	0	0	0	100	0	0	0	0	0	0	0	0	0	0	0	0	0
0	0	0	0	0	100	0	0	0	0	0	0	0	0	0	0	0	0	0	0	0
0	0	0	0	0	100	0	0	0	0	0	0	0	0	0	0	0	0	0	0	0
0	0	0	0	0	100	0	0	0	0	0	0	0	0	0	0	0	0	0	0	0
0	0	0	0	0	0	0	0	0	0	0	0	0	0	0	0	0	0	0	0	0
0	0	0	0	0	0	0	100	0	0	0	0	0	0	0	0	0	0	0	0	0
0	0	0	0	0	100	0	0	0	0	0	0	0	0	0	0	0	0	0	0	0
0	0	0	0	0	0	0	0	0	0	0	0	0	0	0	0	0	0	0	0	0
0	0	0	0	0	0	0	0	0	0	0	0	0	0	0	0	0	0	0	0	0
0	0	0	0	0	0	0	0	0	0	0	0	0	0	0	0	0	0	0	0	0
0	0	0	0	0	0	0	0	0	0	0	0	0	0	0	0	0	0	0	0	0
0	0	0	0	0	0	0	0	0	0	0	0	0	0	0	0	0	0	0	0	0
0	0	0	0	0	0	0	100	0	0	0	0	0	0	0	0	0	0	0	0	0
0	0	0	0	0	100	0	0	0	0	0	0	0	0	0	0	0	0	0	0	0
0	0	0	0	0	0	0	0	0	0	0	0	0	0	0	0	0	0	0	0	0
0	0	0	0	0	0	0	0	0	0	0	0	0	0	0	0	0	0	0	0	0
0	0	0	0	0	100	0	0	0	0	0	0	0	0	0	0	0	0	0	0	0
0	0	0	0	0	0	0	0	0	0	0	0	0	0	0	0	0	0	0	0	0
0	0	0	0	0	0	0	0	0	0	0	0	0	0	0	0	0	0	0	0	0
0	0	0	0	0	100	0	0	0	0	0	0	0	0	0	0	0	0	0	0	0
0	0	0	0	0	100	0	0	0	0	0	0	0	0	0	0	0	0	0	0	0
0	0	0	0	0	0	0	0	0	0	0	0	0	0	0	0	0	0	0	0	0
0	0	0	0	0	100	0	0	0	0	0	0	0	0	0	0	0	0	0	0	0
0	0	0	0	0	0	0	0	0	0	0	0	0	0	0	0	0	0	0	0	0
0	0	0	0	0	100	0	0	0	0	0	0	0	0	0	0	0	0	0	0	0
0	0	0	0	0	100	0	0	0	0	0	0	0	0	0	0	0	0	0	0	0

0	0	0	0	0	100	0	0	0	0	0	0	0	0	0	0	0	0	0	0	0
0	0	0	0	0	100	0	0	0	0	0	0	0	0	0	0	0	0	0	0	0
0	0	0	0	0	100	0	0	0	0	0	0	0	0	0	0	0	0	0	0	0
0	0	0	0	0	100	0	0	0	0	0	0	0	0	0	0	0	0	0	0	0
0	0	0	0	0	100	0	0	0	0	0	0	0	0	0	0	0	0	0	0	0
0	0	0	0	0	100	0	0	0	0	0	0	0	0	0	0	0	0	0	0	0
0	0	0	0	0	100	0	0	0	0	0	0	0	0	0	0	0	0	0	0	0
0	0	0	0	0	100	0	0	0	0	0	0	0	0	0	0	0	0	0	0	0
0	0	0	0	0	100	0	0	0	0	0	0	0	0	0	0	0	0	0	0	0
0	0	0	0	0	100	0	0	0	0	0	0	0	0	0	0	0	0	0	0	0
0	0	0	0	0	100	0	0	0	0	0	0	0	0	0	0	0	0	0	0	0
0	0	0	0	0	0	0	100	0	0	0	0	0	0	0	0	0	0	0	0	0
0	0	0	0	0	100	0	0	0	0	0	0	0	0	0	0	0	0	0	0	0
0	0	0	0	0	100	0	0	0	0	0	0	0	0	0	0	0	0	0	0	0
0	0	0	0	0	0	0	0	0	0	0	100	0	0	0	0	0	0	0	0	0
0	0	0	0	0	100	0	0	0	0	0	0	0	0	0	0	0	0	0	0	0
0	0	0	0	0	100	0	0	0	0	0	0	0	0	0	0	0	0	0	0	0
0	0	0	0	0	100	0	0	0	0	0	0	0	0	0	0	0	0	0	0	0
0	0	0	0	0	100	0	0	0	0	0	0	0	0	0	0	0	0	0	0	0
0	0	0	0	0	0	0	0	0	0	0	0	0	0	0	0	0	0	0	0	100
0	0	0	0	0	100	0	0	0	0	0	0	0	0	0	0	0	0	0	0	0
0	0	0	0	0	100	0	100	0	0	0	0	0	0	0	0	0	0	0	0	0
0	0	0	0	0	0	0	0	0	0	0	0	0	0	0	0	0	0	0	0	0
0	0	0	0	0	100	0	0	0	0	0	0	0	0	0	0	0	0	0	0	0
0	0	0	0	0	0	0	0	0	0	0	0	0	0	100	0	0	0	0	0	0
0	0	0	0	0	100	0	0	0	0	0	0	0	0	0	0	0	0	0	0	0
0	0	0	0	0	0	0	0	0	0	0	0	0	0	0	0	0	0	0	0	0
0	0	0	0	0	0	0	0	0	0	0	0	0	0	0	0	0	0	0	0	0
0	0	0	0	0	0	0	0	0	0	0	0	0	0	0	0	0	0	0	0	100
0	0	0	0	0	0	0	0	0	0	0	0	0	0	0	0	0	0	0	0	0
0	0	0	0	0	0	0	0	0	0	0	0	0	0	0	0	0	0	0	0	100
0	0	0	0	0	0	0	0	0	0	0	0	0	0	0	0	0	0	0	0	0
0	0	0	0	0	0	0	0	0	0	0	0	0	0	0	0	0	0	0	0	0
0	0	0	0	0	100	0	0	0	0	0	0	0	0	0	0	0	0	0	0	0
0	0	0	0	0	0	0	0	0	0	0	0	0	0	0	0	0	0	0	0	0
0	0	0	0	0	0	0	0	0	0	0	0	0	0	0	0	0	0	0	0	100
0	0	0	0	0	0	0	0	0	0	0	0	0	0	0	0	0	0	0	0	100
0	0	0	0	0	0	0	0	0	0	0	0	0	0	0	0	0	0	0	0	100
0	0	0	0	0	100	0	0	0	0	0	0	0	0	0	0	0	0	0	0	0
0	0	0	0	0	0	0	0	0	0	0	0	0	0	0	0	0	0	0	0	100
0	0	0	0	0	100	0	0	0	0	0	0	0	0	0	0	0	0	0	0	0
0	0	0	0	0	0	0	0	0	0	0	0	0	0	0	0	0	0	0	0	100
0	0	0	0	0	0	0	0	0	0	0	0	0	0	0	0	0	0	0	0	0
0	0	0	0	0	0	0	0	0	0	0	0	0	0	0	0	0	0	0	0	0
0	0	0	0	0	0	0	0	0	0	0	0	0	0	0	0	0	0	0	0	0
0	0	0	0	0	0	0	100	0	0	0	0	0	0	0	0	0	0	0	0	0
0	0	0	0	0	0	0	0	0	0	0	0	0	0	0	0	0	0	0	0	100
0	0	0	0	0	0	0	0	0	0	0	0	0	0	0	0	0	0	0	0	0
0	0	0	0	0	0	0	0	0	0	0	0	0	0	0	0	0	0	0	0	0
0	0	0	0	0	0	0	0	0	0	0	0	0	0	0	0	0	0	0	0	0
0	0	0	0	0	0	0	0	0	0	0	0	0	0	0	0	0	0	0	0	0
0	0	0	0	0	0	0	0	0	0	0	0	0	0	0	0	0	0	0	0	100
0	0	0	0	0	100	0	0	0	0	0	0	0	0	0	0	0	0	0	0	0
0	0	0	0	0	100	0	0	0	0	0	0	0	0	0	0	0	0	0	0	0

0	0	0	0	0	0	0	0	0	0	0	0	0	0	100	0	0	0	0	0	0
0	0	0	0	0	0	0	0	0	0	0	0	0	0	0	0	0	0	0	0	0
0	0	0	0	0	0	0	0	0	0	0	0	0	0	0	0	0	0	0	0	0
0	0	0	0	0	0	0	0	0	0	0	0	0	0	0	0	0	0	0	0	0
0	0	0	0	0	0	0	0	0	0	0	0	0	0	0	0	0	0	0	0	100
0	0	0	0	0	0	0	0	0	0	0	0	0	0	0	0	0	0	0	0	0
0	0	0	0	0	0	0	0	0	0	0	0	0	0	0	0	0	0	0	0	0
0	0	0	0	0	0	0	100	0	0	0	0	0	0	0	0	0	0	0	0	0
0	0	0	0	0	0	0	0	0	0	0	0	0	0	0	0	0	0	0	0	0
0	0	0	0	0	100	0	0	0	0	0	0	0	0	0	0	0	0	0	0	0
0	0	0	0	0	100	0	0	0	0	0	0	0	0	0	0	0	0	0	0	0
0	100	100	0	0	0	100	0	0	0	0	0	0	0	0	100	0	0	0	0	0
0	0	0	0	0	100	0	0	0	0	0	0	0	0	0	0	0	0	0	0	0
0	0	0	0	0	0	0	0	0	0	0	0	0	0	0	0	0	0	0	0	0
0	0	0	0	0	0	0	0	0	0	0	0	0	0	0	0	0	0	0	0	0
0	0	0	0	0	0	0	0	0	0	0	0	0	0	0	0	0	0	0	0	0
0	0	0	0	0	0	0	0	0	0	0	0	0	0	0	0	0	0	0	0	100
0	0	0	0	0	0	0	0	0	0	0	0	0	0	0	0	0	0	0	0	0
0	0	0	0	0	0	0	0	0	0	0	0	0	0	0	0	0	0	0	0	0
0	0	0	0	0	0	0	0	0	0	0	0	0	0	0	0	0	0	0	0	0
0	0	0	0	0	0	0	100	0	0	0	0	0	0	0	0	0	0	0	0	0
0	0	0	0	0	0	0	0	0	0	0	0	0	0	0	0	0	0	0	0	0
0	0	0	0	0	0	0	0	0	0	0	0	0	0	0	0	0	0	0	0	0
0	0	0	0	0	0	0	0	0	0	0	0	0	0	0	0	0	0	0	0	0
0	0	0	0	0	100	0	0	0	0	0	0	0	0	0	0	0	0	0	0	0
0	0	0	0	0	0	0	0	0	0	0	0	0	0	0	0	0	0	0	0	0
0	0	0	0	0	100	0	0	0	0	0	0	0	0	0	0	0	0	0	0	0
0	0	0	0	0	100	0	0	0	0	0	0	0	0	0	0	0	0	0	0	0
0	0	0	0	0	100	0	0	0	0	0	0	0	0	0	0	0	0	0	0	0
0	0	0	0	0	100	0	0	0	0	0	0	0	0	0	0	0	0	0	0	0
0	0	0	0	0	100	0	0	0	0	0	0	0	0	0	0	0	0	0	0	0
0	0	0	0	0	100	0	0	0	0	0	0	0	0	0	0	0	0	0	0	0
0	0	0	0	0	100	0	0	0	0	0	0	0	0	0	0	0	0	0	0	0
0	0	0	0	0	100	0	0	0	0	0	0	0	0	0	0	0	0	0	0	0
0	0	0	0	0	100	0	0	0	0	0	0	0	0	0	0	0	0	0	0	0
0	0	0	0	0	100	0	0	0	0	0	0	0	0	0	0	0	0	0	0	0
0	0	0	0	0	100	0	0	0	0	0	0	0	0	0	0	0	0	0	0	0
0	0	0	0	0	100	0	0	0	0	0	0	0	0	0	0	0	0	0	0	0
0	0	0	0	0	100	0	0	0	0	0	0	0	0	0	0	0	0	0	0	0
0	0	0	0	0	100	0	0	0	0	0	0	0	0	0	0	0	0	0	0	0
0	0	0	0	0	100	0	0	0	0	0	0	0	0	0	0	0	0	0	0	0
0	0	0	0	0	100	0	0	0	0	0	0	0	0	0	0	0	0	0	0	0
0	0	0	0	0	100	0	0	0	0	0	0	0	0	0	0	0	0	0	0	0
0	0	0	0	0	100	0	0	0	0	0	0	0	0	0	0	0	0	0	0	0
0	0	0	0	0	100	0	0	0	0	0	0	0	0	0	0	0	0	0	0	0
0	0	0	0	0	100	0	0	0	0	0	0	0	0	0	0	0	0	0	0	0
0	0	0	0	0	100	0	0	0	0	0	0	0	0	0	0	0	0	0	0	0
0	0	0	0	0	100	0	0	0	0	0	0	0	0	0	0	0	0	0	0	0
0	0	0	0	0	100	0	0	0	0	0	0	0	0	0	0	0	0	0	0	0
0	0	0	0	0	100	0	0	0	0	0	0	0	0	0	0	0	0	0	0	0
0	0	0	0	0	100	0	0	0	0	0	0	0	0	0	0	0	0	0	0	0
0	0	0	0	0	100	0	0	0	0	0	0	0	0	0	0	0	0	0	0	0
0	0	0	0	0	100	0	0	0	0	0	0	0	0	0	0	0	0	0	0	0
0	0	0	0	0	100	0	0	0	0	0	0	0	0	0	0	0	0	0	0	0

0	0	0	0	0	100	0	0	0	0	0	0	0	0	0	0	0	0	0	0	0
0	0	0	0	0	100	0	0	0	0	0	0	0	0	0	0	0	0	0	0	0
0	0	0	0	0	100	0	0	0	0	0	0	0	0	0	0	0	0	0	0	0
0	0	0	0	0	100	0	0	0	0	0	0	0	0	0	0	0	0	0	0	0
0	0	0	0	0	100	0	0	0	0	0	0	0	0	0	0	0	0	0	0	0
0	0	0	0	0	100	0	0	0	0	0	0	0	0	0	0	0	0	0	0	0
0	0	0	0	0	100	0	0	0	0	0	0	0	0	0	0	0	0	0	0	0
0	0	0	0	0	100	0	0	0	0	0	0	0	0	0	0	0	0	0	0	0
0	0	0	0	0	100	0	0	0	0	0	0	0	0	0	0	0	0	0	0	0
0	0	0	0	0	100	0	0	0	0	0	0	0	0	0	0	0	0	0	0	0
0	0	0	0	0	100	0	0	0	0	0	0	0	0	0	0	0	0	0	0	0
0	0	0	0	0	100	0	0	0	0	0	0	0	0	0	0	0	0	0	0	0
0	0	0	0	0	100	0	0	0	0	0	0	0	0	0	0	0	0	0	0	0
0	0	0	0	0	100	0	0	0	0	0	0	0	0	0	0	0	0	0	0	0
0	0	0	0	0	100	0	0	0	0	0	0	0	0	0	0	0	0	0	0	0
0	0	0	0	0	100	0	0	0	0	0	0	0	0	0	0	0	0	0	0	0
0	0	0	0	0	100	0	0	0	0	0	0	0	0	0	0	0	0	0	0	0
0	0	0	0	0	100	0	0	0	0	0	0	0	0	0	0	0	0	0	0	0
0	0	0	0	0	100	0	0	0	0	0	0	0	0	0	0	0	0	0	0	0
0	0	0	0	0	100	0	0	0	0	0	0	0	0	0	0	0	0	0	0	0
0	0	0	0	0	100	0	0	0	0	0	0	0	0	0	0	0	0	0	0	0
0	0	0	0	0	100	0	0	0	0	0	0	0	0	0	0	0	0	0	0	0
0	0	0	0	0	100	0	0	0	0	0	0	0	0	0	0	0	0	0	0	0
0	0	0	0	0	100	0	0	0	0	0	0	0	0	0	0	0	0	0	0	0
0	0	0	0	0	100	0	0	0	0	0	0	0	0	0	0	0	0	0	0	0
0	0	0	0	0	100	0	0	0	0	0	0	0	0	0	0	0	0	0	0	0
0	0	0	0	0	100	0	0	0	0	0	0	0	0	0	0	0	0	0	0	0
0	0	0	0	0	100	0	0	0	0	0	0	0	0	0	0	0	0	0	0	0
0	0	0	0	0	100	0	0	0	0	0	0	0	0	0	0	0	0	0	0	0
0	0	0	0	0	100	0	0	0	0	0	0	0	0	0	0	0	0	0	0	0
0	0	0	0	0	100	0	0	0	0	0	0	0	0	0	0	0	0	0	0	0
0	0	0	0	0	100	0	0	0	0	0	0	0	0	0	0	0	0	0	0	0
0	0	0	0	0	100	0	0	0	0	0	0	0	0	0	0	0	0	0	0	0
0	0	0	0	0	100	0	0	0	0	0	0	0	0	0	0	0	0	0	0	0
0	0	0	0	0	100	0	0	0	0	0	0	0	0	0	0	0	0	0	0	0
0	0	0	0	0	100	0	0	0	0	0	0	0	0	0	0	0	0	0	0	0
0	0	0	0	0	100	0	0	0	0	0	0	0	0	0	0	0	0	0	0	0
0	0	0	0	0	100	0	0	0	0	0	0	0	0	0	0	0	0	0	0	0
0	0	0	0	0	100	0	0	0	0	0	0	0	0	0	0	0	0	0	0	0
0	0	0	0	0	100	0	0	0	0	0	0	0	0	0	0	0	0	0	0	0
0	0	0	0	0	100	0	0	0	0	0	0	0	0	0	0	0	0	0	0	0
0	0	0	0	0	100	0	0	0	0	0	0	0	0	0	0	0	0	0	0	0
0	0	0	0	0	100	0	0	0	0	0	0	0	0	0	0	0	0	0	0	0
0	0	0	0	0	100	0	0	0	0	0	0	0	0	0	0	0	0	0	0	0
0	0	0	0	0	100	0	0	0	0	0	0	0	0	0	0	0	0	0	0	0
0	0	0	0	0	100	0	0	0	0	0	0	0	0	0	0	0	0	0	0	0
0	0	0	0	0	100	0	0	0	0	0	0	0	0	0	0	0	0	0	0	0
0	0	0	0	0	100	0	0	0	0	0	0	0	0	0	0	0	0	0	0	0
0	0	0	0	0	100	0	0	0	0	0	0	0	0	0	0	0	0	0	0	0
0	0	0	0	0	100	0	0	0	0	0	0	0	0	0	0	0	0	0	0	0
0	0	0	0	0	100	0	0	0	0	0	0	0	0	0	0	0	0	0	0	0
0	0	0	0	0	100	0	0	0	0	0	0	0	0	0	0	0	0	0	0	0
0	0	0	0	0	100	0	0	0	0	0	0	0	0	0	0	0	0	0	0	0
0	0	0	0	0	100	0	0	0	0	0	0	0	0	0	0	0	0	0	0	0

0	0	0	0	0	100	0	0	0	0	0	0	0	0	0	0	0	0	0	0	0
0	0	0	0	0	100	0	0	0	0	0	0	0	0	0	0	0	0	0	0	0
0	0	0	0	0	100	0	0	0	0	0	0	0	0	0	0	0	0	0	0	0
0	0	0	0	0	100	0	0	0	0	0	0	0	0	0	0	0	0	0	0	0
0	0	0	0	0	100	0	0	0	0	0	0	0	0	0	0	0	0	0	0	0
0	0	0	0	0	100	0	0	0	0	0	0	0	0	0	0	0	0	0	0	0
0	0	0	0	0	0	0	100	0	0	0	0	0	0	0	0	0	0	0	0	0
0	0	0	0	0	0	0	0	0	0	0	0	0	0	0	0	0	0	0	0	0
0	0	0	0	0	0	0	0	0	0	0	0	0	0	0	0	0	0	0	0	0
0	0	0	0	0	100	0	0	0	0	0	0	0	0	0	0	0	0	0	0	0
0	0	0	0	0	100	0	0	0	0	0	0	0	0	0	0	0	0	0	0	0
0	0	0	0	0	100	0	0	0	0	0	0	0	0	0	0	0	0	0	0	0
0	0	0	0	0	100	0	0	0	0	0	0	0	0	0	0	0	0	0	0	0
0	0	0	0	0	100	0	0	0	0	0	0	0	0	0	0	0	0	0	0	0
0	0	0	0	0	100	0	0	0	0	0	0	0	0	0	0	0	0	0	0	0
0	0	0	0	0	100	0	0	0	0	0	0	0	0	0	0	0	0	0	0	0
0	0	0	0	0	100	0	0	0	0	0	0	0	0	0	0	0	0	0	0	0
0	0	0	0	0	100	0	0	0	0	0	0	0	0	0	0	0	0	0	0	0
0	0	0	0	0	100	0	0	0	0	0	0	0	0	0	0	0	0	0	0	0
0	0	0	0	0	100	0	0	0	0	0	0	0	0	0	0	0	0	0	0	0
0	0	0	0	0	100	0	0	0	0	0	0	0	0	0	0	0	0	0	0	0
0	0	0	0	0	100	0	0	0	0	0	0	0	0	0	0	0	0	0	0	0
0	0	0	0	0	100	0	0	0	0	0	0	0	0	0	0	0	0	0	0	0
0	0	0	0	0	100	0	0	0	0	0	0	0	0	0	0	0	0	0	0	0
0	0	0	0	0	100	0	0	0	0	0	0	0	0	0	0	0	0	0	0	0
0	0	0	0	0	100	0	0	0	0	0	0	0	0	0	0	0	0	0	0	0
0	0	0	0	0	100	0	0	0	0	0	0	0	0	0	0	0	0	0	0	0
0	0	0	0	0	100	0	0	0	0	0	0	0	0	0	0	0	0	0	0	0
0	0	0	0	0	100	0	0	0	0	0	0	0	0	0	0	0	0	0	0	0
0	0	0	0	0	100	0	0	0	0	0	0	0	0	0	0	0	0	0	0	0
0	0	0	0	0	100	0	0	0	0	0	0	0	0	0	0	0	0	0	0	0
0	0	0	0	0	100	0	0	0	0	0	0	0	0	0	0	0	0	0	0	0
0	0	0	0	0	100	0	0	0	0	0	0	0	0	0	0	0	0	0	0	0
0	0	0	0	0	100	0	0	0	0	0	0	0	0	0	0	0	0	0	0	0
0	0	0	0	0	100	0	0	0	0	0	0	0	0	0	0	0	0	0	0	0
0	0	0	0	0	100	0	0	0	0	0	0	0	0	0	0	0	0	0	0	0
0	0	0	0	0	100	0	0	0	0	0	0	0	0	0	0	0	0	0	0	0
0	0	0	0	0	100	0	0	0	0	0	0	0	0	0	0	0	0	0	0	0
0	0	0	0	0	100	0	0	0	0	0	0	0	0	0	0	0	0	0	0	0
0	0	0	0	0	100	0	0	0	0	0	0	0	0	0	0	0	0	0	0	0
0	0	0	0	0	100	0	0	0	0	0	0	0	0	0	0	0	0	0	0	0
0	0	0	0	0	100	0	0	0	0	0	0	0	0	0	0	0	0	0	0	0
0	0	0	0	0	100	0	0	0	0	0	0	0	0	0	0	0	0	0	0	0
0	0	0	0	0	100	0	0	0	0	0	0	0	0	0	0	0	0	0	0	0
0	0	0	0	0	100	0	0	0	0	0	0	0	0	0	0	0	0	0	0	0
0	0	0	0	0	100	0	0	0	0	0	0	0	0	0	0	0	0	0	0	0
0	0	0	0	0	100	0	0	0	0	0	0	0	0	0	0	0	0	0	0	0
0	0	0	0	0	100	0	0	0	0	0	0	0	0	0	0	0	0	0	0	0
0	0	0	0	0	100	0	0	0	0	0	0	0	0	0	0	0	0	0	0	0
0	0	0	0	0	100	0	0	0	0	0	0	0	0	0	0	0	0	0	0	0
0	0	0	0	0	100	0	0	0	0	0	0	0	0	0	0	0	0	0	0	0
0	0	0	0	0	100	0	0	0	0	0	0	0	0	0	0	0	0	0	0	0
0	0	0	0	0	100	0	0	0	0	0	0	0	0	0	0	0	0	0	0	0
0	0	0	0	0	100	0	0	0	0	0	0	0	0	0	0	0	0	0	0	0

0	0	0	0	0	100	0	0	0	0	0	0	0	0	0	0	0	0	0	0	0
0	0	0	0	0	100	0	0	0	0	0	0	0	0	0	0	0	0	0	0	0
0	0	0	0	0	100	0	0	0	0	0	0	0	0	0	0	0	0	0	0	0
0	0	0	0	0	0	0	0	0	0	0	0	0	0	0	0	0	0	0	0	0
0	0	0	0	0	0	0	0	0	0	0	0	0	0	0	0	0	0	0	0	0
0	0	0	0	0	100	0	0	0	0	0	0	0	0	0	0	0	0	0	0	0
0	0	0	0	0	100	0	0	0	0	0	0	0	0	0	0	0	0	0	0	0
0	0	0	0	0	100	0	0	0	0	0	0	0	0	0	0	0	0	0	0	0
0	0	0	0	0	100	0	0	0	0	0	0	0	0	0	0	0	0	0	0	0
0	0	0	0	0	100	0	0	0	0	0	0	0	0	0	0	0	0	0	0	0
0	0	0	0	0	100	0	0	0	0	0	0	0	0	0	0	0	0	0	0	0
0	0	0	0	0	100	0	0	0	0	0	0	0	0	0	0	0	0	0	0	0
0	0	0	0	0	100	0	0	0	0	0	0	0	0	0	0	0	0	0	0	0
0	0	0	0	0	100	0	0	0	0	0	0	0	0	0	0	0	0	0	0	0
0	0	0	0	0	100	0	0	0	0	0	0	0	0	0	0	0	0	0	0	0
0	0	0	0	0	100	0	0	0	0	0	0	0	0	0	0	0	0	0	0	0
0	0	0	0	0	100	0	0	0	0	0	0	0	0	0	0	0	0	0	0	0
0	0	0	0	0	100	0	0	0	0	0	0	0	0	0	0	0	0	0	0	0
0	0	0	0	0	100	0	0	0	0	0	0	0	0	0	0	0	0	0	0	0
0	0	0	0	0	100	0	0	0	0	0	0	0	0	0	0	0	0	0	0	0
0	0	0	0	0	100	0	0	0	0	0	0	0	0	0	0	0	0	0	0	0
0	0	0	0	0	0	0	0	0	0	0	0	0	0	0	0	0	0	0	0	0
0	0	0	0	0	0	0	0	0	0	0	0	0	0	0	0	0	0	0	0	0
0	0	0	0	0	0	0	0	0	0	0	0	0	0	0	0	0	0	0	0	0
0	0	0	0	0	0	0	0	0	0	0	0	0	0	0	0	0	0	0	0	0
0	0	0	0	0	0	0	0	0	0	0	0	0	0	0	0	0	0	0	0	0
0	0	0	0	0	0	0	0	0	0	0	0	0	0	0	0	0	0	0	0	0
0	0	0	0	0	100	0	0	0	0	0	0	0	0	0	0	0	0	0	0	0
0	0	0	0	0	0	0	0	0	0	0	0	0	0	0	0	0	0	0	0	0
0	0	0	0	0	0	0	0	0	0	0	0	0	0	0	0	0	0	0	0	0
0	0	0	0	0	0	0	0	0	0	0	0	0	0	0	0	0	0	0	0	0
0	0	0	0	0	0	0	0	0	0	0	0	0	0	0	0	0	0	0	0	100
0	0	0	0	0	0	0	0	0	0	0	0	0	0	0	0	0	0	0	0	0
0	0	0	0	0	0	0	0	0	0	0	0	0	0	0	0	0	0	0	0	0
0	0	0	0	0	0	0	0	0	0	0	0	0	0	0	0	0	0	0	0	0
0	0	0	0	0	100	0	0	0	0	0	0	0	0	0	0	0	0	0	0	0
0	0	0	0	0	100	0	0	0	0	0	0	0	0	0	0	0	0	0	0	0
0	0	0	0	0	100	0	0	0	0	0	0	0	0	0	0	0	0	0	0	0
0	0	0	0	0	0	0	0	0	0	0	0	0	0	0	0	0	0	0	0	0
0	0	0	0	0	0	0	0	0	0	0	0	0	0	0	0	0	0	0	0	0
0	0	0	0	0	0	0	0	0	0	0	0	0	0	0	0	0	0	0	0	0
0	0	0	0	0	0	0	0	0	0	0	0	0	0	0	0	0	0	0	0	0
0	0	0	0	0	0	0	0	0	0	0	0	0	0	0	0	0	0	0	0	0
0	0	0	0	0	0	0	0	0	0	0	0	0	0	0	0	0	0	0	0	0
0	0	0	0	0	100	0	0	0	0	0	0	0	0	0	0	0	0	0	0	0
0	0	0	0	0	0	0	0	0	0	0	0	0	0	0	0	0	0	0	0	0
0	0	0	0	0	0	0	0	0	0	0	0	0	0	0	0	0	0	0	0	0
0	0	0	0	0	0	0	0	0	0	0	0	0	0	0	0	0	0	0	0	0
0	0	0	0	0	0	0	0	0	0	0	0	0	0	0	0	0	0	0	0	100
0	0	0	0	0	0	0	0	0	0	0	0	0	0	0	0	0	0	0	0	0
0	0	0	0	0	0	0	0	0	0	0	0	0	0	0	0	0	0	0	0	0
0	0	0	0	0	0	0	0	0	0	0	0	0	0	0	0	0	0	0	0	0
0	0	0	0	0	0	0	0	0	0	0	0	0	0	0	0	0	0	0	0	0
0	0	0	0	0	0	0	0	0	0	0	0	0	0	0	0	0	0	0	0	0

0	0	0	0	0	0	0	0	0	0	0	0	0	0	0	0	0	0	0	0	0
0	0	0	0	0	0	0	0	0	0	0	0	0	0	0	0	0	0	0	0	0
0	0	0	0	0	0	0	0	0	0	0	0	0	0	0	0	0	0	0	0	0
0	0	0	0	0	0	0	0	0	0	0	0	0	0	0	0	0	0	0	0	0
0	0	0	0	0	0	0	0	0	0	0	0	100	0	0	0	0	0	0	0	0
0	0	0	0	0	0	0	0	0	0	0	0	100	0	0	0	0	0	0	0	0
0	0	0	0	0	0	0	0	0	0	0	0	0	0	0	0	0	0	0	0	0
0	0	0	0	0	0	0	0	0	0	0	0	0	0	0	0	0	0	0	0	0
0	0	0	0	0	0	0	0	0	0	0	0	0	0	0	0	0	0	0	0	0
0	0	0	0	0	0	0	0	0	0	0	0	0	0	0	0	0	0	0	0	0
0	0	0	0	0	0	0	0	0	0	0	0	100	0	0	0	0	0	0	0	0
0	0	0	0	0	0	0	0	0	0	0	0	0	0	0	0	0	0	0	0	0
0	0	0	0	0	0	0	0	0	0	0	0	100	0	0	0	0	0	0	0	0
0	0	0	0	0	0	0	0	0	0	0	0	0	0	0	0	0	0	0	0	0
0	0	0	0	0	0	0	0	0	0	0	0	0	0	0	0	0	0	0	0	0
0	0	0	0	0	0	0	0	0	0	0	0	0	0	0	0	0	0	0	0	0
0	0	0	0	0	0	0	0	0	0	0	0	100	0	0	0	0	0	0	0	0
0	0	0	0	0	0	0	0	0	0	0	0	0	0	0	0	0	0	0	0	0
0	0	0	0	0	0	0	0	0	0	0	0	100	0	0	0	0	0	0	0	0
0	0	0	0	0	0	0	0	0	0	0	0	100	0	0	0	0	0	0	0	0
0	0	0	0	0	0	0	0	0	0	0	0	0	0	0	0	0	0	0	0	0
0	0	0	0	0	0	0	0	0	0	0	0	0	0	0	0	0	0	0	0	0
0	0	0	0	0	0	0	0	0	0	0	0	100	0	0	0	0	0	0	0	0
0	0	0	0	0	0	0	0	0	0	0	0	0	0	0	0	0	0	0	0	0
0	0	0	0	0	0	0	0	0	0	0	0	100	0	0	0	0	0	0	0	0
0	0	0	0	0	0	0	0	0	0	0	0	100	0	0	0	0	0	0	0	0
0	0	0	0	0	0	0	0	0	0	0	0	0	0	0	0	0	0	0	0	0
0	0	0	0	0	0	0	0	0	0	0	0	0	0	0	0	0	0	0	0	0
0	0	0	0	0	0	0	0	0	0	0	0	0	0	0	0	0	0	0	0	0
0	0	0	0	0	0	0	0	0	0	0	0	0	0	0	0	0	0	0	0	0
0	0	0	0	0	0	0	0	0	0	0	0	0	0	0	0	0	0	0	0	0
0	0	0	0	0	0	0	0	0	0	0	0	0	0	0	0	0	0	0	0	0
0	0	0	0	0	0	0	0	0	0	0	0	0	0	0	0	0	0	0	0	0
0	0	0	0	0	0	0	0	0	0	0	0	0	0	0	0	0	0	0	0	0
0	0	0	0	0	0	0	0	0	0	0	0	0	0	0	0	0	0	0	0	0
0	0	0	0	0	0	0	100	0	0	0	0	0	0	0	0	0	0	0	0	0
0	0	0	0	0	0	0	0	0	0	0	0	0	0	0	0	0	0	0	0	0
0	0	0	0	0	0	0	0	0	0	0	0	0	0	0	0	0	0	0	0	0
0	0	0	0	0	0	0	0	0	0	0	0	0	0	0	0	0	0	0	0	0
0	0	0	0	0	0	0	0	0	0	0	0	0	0	100	0	0	0	0	0	0
0	0	0	0	0	0	0	0	0	0	0	0	0	0	0	0	0	0	0	0	0
0	0	0	0	0	0	0	0	0	0	0	0	0	0	0	0	0	0	0	0	0
0	0	0	0	0	0	0	0	0	0	0	0	0	0	0	0	0	0	0	0	0
0	0	0	0	0	0	0	0	0	0	0	0	0	0	0	0	0	0	0	0	0
0	0	0	0	0	0	0	100	0	0	0	0	0	0	0	0	0	0	0	0	0
0	0	0	0	0	0	0	0	0	0	0	0	0	0	0	0	0	0	0	0	0
0	0	0	0	0	0	0	100	0	0	0	0	0	0	0	0	0	0	0	0	0
0	0	0	0	0	0	0	0	0	0	0	0	0	0	0	0	0	0	0	0	0
0	0	0	0	0	0	0	0	0	0	0	0	0	0	0	0	0	0	0	0	0
0	0	0	0	0	0	0	0	0	0	0	0	0	0	0	0	0	0	0	0	0
0	0	0	0	0	0	0	100	0	0	0	0	0	0	0	0	0	0	0	0	0
0	0	0	0	0	0	0	0	0	0	0	0	0	0	0	0	0	0	0	0	0
0	0	0	0	0	0	0	100	0	0	0	0	0	0	0	0	0	0	0	0	0

0	0	0	0	0	0	0	0	0	0	0	0	0	0	0	0	0	0	0	0	0
0	0	0	0	0	0	0	0	0	0	0	0	0	0	0	0	0	0	0	0	0
0	0	0	0	0	0	0	0	0	0	0	0	0	0	0	0	0	0	0	0	0
0	0	0	0	0	0	0	0	0	0	0	0	0	0	0	0	0	0	0	0	0
0	0	0	0	0	0	0	0	0	0	0	0	0	0	0	0	77.78	0	0	0	0
0	0	0	0	0	0	0	0	0	0	0	0	0	0	0	0	77.78	0	0	0	0
0	0	0	0	0	0	0	0	0	0	0	0	0	0	0	0	0	0	0	0	0
0	0	0	0	0	0	0	0	0	0	0	0	0	0	0	0	0	0	0	0	0
0	0	0	0	0	0	0	0	0	0	0	0	0	0	0	0	0	0	0	0	0
0	0	0	0	0	0	0	0	0	0	0	0	0	0	0	0	0	0	0	0	0
0	0	0	0	0	0	0	0	0	0	0	0	0	0	0	0	0	0	0	0	0
0	0	0	0	0	0	0	0	0	0	0	0	0	0	0	0	0	0	0	0	0
0	0	0	0	0	0	0	0	0	0	0	0	0	0	0	0	0	0	0	0	0
0	0	0	0	0	0	0	0	0	0	0	0	0	0	0	0	0	0	0	0	0
0	0	0	0	0	0	0	0	0	0	0	0	0	0	0	0	0	0	0	0	0
0	0	0	0	0	0	0	0	0	0	0	0	0	0	0	0	0	0	0	0	0
0	0	0	0	0	0	0	0	0	0	0	0	0	0	0	0	0	0	0	0	0
0	0	0	0	0	0	0	0	0	0	0	0	0	0	0	0	0	0	0	0	0
0	0	0	0	0	0	0	0	0	0	0	0	0	0	0	0	0	0	0	0	0
0	0	0	0	0	0	0	0	0	0	0	0	0	0	0	0	0	0	0	0	0
0	0	0	0	0	0	0	0	0	0	0	0	0	0	0	0	0	0	0	0	0
0	0	0	0	0	0	0	0	0	0	0	0	0	0	0	0	77.78	0	0	0	0
0	0	0	0	0	0	0	0	0	0	0	0	0	0	0	0	0	0	0	0	0
0	0	0	0	0	0	0	0	0	0	0	0	0	0	0	0	0	0	0	0	0
0	0	0	0	0	0	0	0	0	0	0	0	0	0	0	0	0	0	0	0	0
0	0	0	0	0	0	0	0	0	0	0	0	0	0	0	0	77.78	0	0	0	0
0	0	0	0	0	0	0	0	0	0	0	0	0	0	0	0	77.78	0	0	0	0
0	0	0	0	0	0	0	0	0	0	0	0	0	0	0	0	77.78	0	0	0	0
0	0	0	0	0	0	0	0	0	0	0	0	0	0	0	0	0	0	0	0	0
0	0	0	0	0	0	0	0	0	0	0	0	0	0	0	0	0	0	0	0	0
0	0	0	0	0	0	0	0	0	0	0	0	0	0	0	0	77.78	0	0	0	0
0	0	0	0	0	0	0	0	0	0	0	0	0	0	0	0	0	0	0	0	0
0	0	0	0	0	0	0	0	0	0	0	0	0	0	0	0	0	0	0	0	0
0	0	0	0	0	0	0	0	0	0	0	0	0	0	0	0	0	0	0	0	0
0	0	0	0	0	0	0	0	0	0	0	0	0	0	0	0	0	0	0	0	0
0	0	0	0	0	0	0	0	0	0	0	0	0	0	0	0	0	0	0	0	0
0	0	0	0	0	0	0	0	0	0	0	0	0	0	0	0	0	0	0	0	0
0	0	0	0	0	0	0	0	0	0	0	0	0	0	0	0	0	0	0	0	0
0	0	0	0	0	0	0	0	0	0	0	0	0	0	0	0	0	0	0	0	0
0	0	0	0	0	0	0	0	0	0	0	0	0	0	0	0	0	0	0	0	0
0	0	0	0	0	0	0	0	0	0	0	0	0	0	0	0	0	0	0	0	0
0	0	0	0	0	0	0	0	0	0	0	0	0	0	0	0	0	0	0	0	0
0	0	0	0	0	0	0	0	0	0	0	0	0	0	0	0	0	0	0	0	0
0	0	0	0	0	0	0	0	0	0	0	0	0	0	0	0	0	0	0	0	0
0	0	0	0	0	0	0	0	0	0	0	0	0	0	0	0	0	0	0	0	0
0	0	0	0	0	0	0	0	0	0	0	0	0	0	0	0	0	0	0	0	0
0	0	0	0	0	0	0	0	0	0	0	0	0	0	0	0	0	0	0	0	0
0	0	0	0	0	0	0	0	0	0	0	0	0	0	0	0	0	0	0	0	0
0	0	0	0	0	0	0	0	0	0	0	0	0	0	0	0	0	0	0	0	0
0	0	0	0	0	0	0	0	0	0	0	0	0	0	0	0	0	0	0	0	0
0	0	0	0	0	0	0	0	0	0	0	0	0	0	0	0	0	0	0	0	0
0	0	0	0	0	0	0	0	0	0	0	0	0	0	0	0	0	0	0	0	0
0	0	0	0	0	0	0	0	0	0	0	0	0	0	0	0	0	0	0	0	0
0	0	0	0	0	0	0	0	0	0	0	0	0	0	0	0	0	0	0	0	0

0	0	0	0	0	0	0	0	0	0	0	0	0	0	0	0	0	0	0	0	0
0	0	0	0	0	0	0	0	0	0	0	0	0	0	0	0	0	0	0	0	0
0	0	0	0	0	0	0	0	0	0	0	0	0	0	0	0	0	0	0	0	0
0	0	0	0	0	0	0	0	0	0	0	0	0	0	0	0	0	0	0	0	0
0	0	0	0	0	0	0	0	0	0	0	0	0	0	0	0	0	0	0	0	0
0	0	0	0	0	0	0	0	0	0	0	0	0	0	0	0	0	0	0	0	0
0	0	0	0	0	0	0	0	0	0	0	0	0	0	0	0	0	0	0	0	0
0	0	0	0	0	0	0	0	0	0	0	0	0	0	0	0	77.78	0	0	0	0
0	0	0	0	0	0	0	0	0	0	0	0	0	0	0	0	0	0	0	0	0
0	0	0	0	0	0	0	0	0	0	0	0	0	0	0	0	0	0	0	0	0
0	0	0	0	0	0	0	0	0	0	0	0	0	0	0	0	0	0	0	0	0
0	0	0	0	0	0	0	0	0	0	0	0	0	0	0	0	0	0	0	0	0
0	0	0	0	0	0	0	0	0	0	0	0	0	0	0	0	0	0	0	0	0
0	0	0	0	0	0	0	0	0	0	0	0	0	0	0	0	0	0	0	0	0
0	0	0	0	0	0	0	0	0	0	0	0	0	0	0	0	0	0	0	0	0
0	0	0	0	0	0	0	0	0	0	0	0	0	0	0	0	0	0	0	0	0
0	0	0	0	0	0	0	0	0	0	0	0	0	0	0	0	0	0	0	0	0
0	0	0	0	0	0	0	0	0	0	0	0	0	0	0	0	0	0	0	0	0
0	0	0	0	0	0	0	0	0	0	0	0	0	0	0	0	0	0	0	0	0
0	0	0	0	0	0	0	0	0	0	0	0	0	0	0	0	0	0	0	0	0
0	0	0	0	0	0	0	0	0	0	0	0	0	0	0	0	0	0	0	0	0
0	0	0	0	0	0	0	0	0	0	0	0	0	0	0	0	0	0	0	0	0
0	0	0	0	0	0	0	0	0	0	0	0	0	0	0	0	0	0	0	0	0
0	0	0	0	0	0	0	0	0	0	0	0	0	0	0	0	0	0	0	0	0
0	0	0	0	0	0	0	0	0	0	0	0	0	0	0	0	0	0	0	0	0
0	0	0	0	0	0	0	0	0	0	0	0	0	0	0	0	0	0	0	0	0
0	0	0	0	0	0	0	0	0	0	0	0	0	0	0	0	0	0	0	0	0
0	0	0	0	0	0	0	0	0	0	0	0	0	0	0	0	0	0	0	0	0
0	0	0	0	0	0	0	0	0	0	0	0	0	0	0	0	77.78	0	0	0	0
0	0	0	0	0	0	0	0	0	0	0	0	0	0	0	0	0	0	0	0	0
0	0	0	0	0	0	0	0	0	0	0	0	0	0	0	0	0	0	0	0	0
0	0	0	0	0	0	0	0	0	0	0	0	0	0	0	0	0	0	0	0	0
0	0	0	0	0	0	0	0	0	0	0	0	0	0	0	0	0	0	0	0	0
0	0	0	0	0	0	0	0	0	0	0	0	0	0	0	0	77.78	0	0	0	0
0	0	0	0	0	0	0	0	0	0	0	0	0	0	0	0	0	0	0	0	0
0	0	0	0	0	0	0	0	0	0	0	0	0	0	0	0	0	0	0	0	0
0	0	0	0	0	0	0	0	0	0	0	0	0	0	0	0	0	0	0	0	0
0	0	0	0	0	0	0	0	0	0	0	0	0	0	0	0	0	0	0	0	0
0	0	0	0	0	0	0	0	0	0	0	0	0	0	0	0	0	0	0	0	0
0	0	0	0	0	0	0	0	0	0	0	0	0	0	0	0	0	0	0	0	0
0	0	0	0	0	0	0	0	0	0	0	0	0	0	0	0	0	0	0	0	0
0	0	0	0	0	0	0	0	0	0	0	0	0	0	0	0	0	0	0	0	0
0	0	0	0	0	0	0	0	0	0	0	0	0	0	0	0	0	0	0	0	0
0	0	0	0	0	0	0	0	0	0	0	0	0	0	0	0	77.78	0	0	0	0
0	0	0	0	0	0	0	0	0	0	0	0	0	0	0	0	0	0	0	0	0
0	0	0	0	0	0	0	0	0	0	0	0	0	0	0	0	0	0	0	0	0

CTX-M-64	CTX-M-65	CTX-M-67	CTX-M-93		OXA-1	OXA-10	OXA-664	TEM-1	TEM-113	TEM-116	TEM-122	TEM-131	TEM-138	TEM-143	TEM-17	TEM-193	TEM-209	TEM-214	TEM-220	TEM-43
0	0	0	0	100	0	0	0	0	0	0	0	0	0	0	0	0	0	0	0	0
0	0	0	0	100	0	0	0	0	0	0	100	0	0	0	0	0	0	0	0	0
0	0	0	0	0	0	0	0	100	0	0	0	0	0	0	0	0	0	0	0	0
0	0	0	0	0	0	0	0	100	0	0	0	0	0	0	0	0	0	0	0	0
0	0	0	0	0	0	0	0	0	0	0	0	0	0	0	0	0	0	0	0	0
0	0	0	0	0	0	0	0	0	0	0	0	0	0	0	0	0	0	0	0	0
0	0	0	0	0	0	0	0	100	0	0	0	0	0	0	0	0	0	0	0	0
0	0	0	0	0	0	0	0	0	0	0	0	0	0	0	0	0	0	0	0	0
0	0	0	0	0	0	0	0	100	0	0	0	0	0	0	0	0	0	0	0	0
0	0	0	0	0	0	0	0	0	0	0	0	0	0	0	0	0	0	0	0	0
0	0	0	0	0	0	0	0	100	0	0	0	0	0	0	0	0	0	0	0	0
0	0	0	0	100	0	0	0	0	0	0	0	0	0	0	0	0	0	0	0	0
0	0	0	0	100	0	0	0	0	0	0	0	0	0	0	0	0	0	0	0	0
0	0	0	0	100	0	0	0	0	0	0	0	0	0	0	0	0	0	0	0	0
0	0	0	0	100	0	0	0	0	0	0	0	0	0	0	0	0	0	0	0	0
0	0	0	0	100	0	0	0	0	0	0	0	0	0	0	0	0	0	0	0	0
0	0	0	0	100	0	0	0	0	0	0	0	0	0	0	0	0	0	0	0	0
0	0	0	0	100	0	0	0	0	0	0	0	0	0	0	0	0	0	0	0	0
0	0	0	0	100	0	0	0	0	0	0	0	0	0	0	0	0	0	0	0	0
0	0	0	0	100	0	0	0	0	0	0	0	0	0	0	0	0	0	0	0	0
0	0	0	0	100	0	0	0	0	0	0	0	0	0	0	0	0	0	0	0	0
0	0	0	0	100	0	0	0	0	0	0	0	0	0	0	0	0	0	0	0	0
0	0	0	0	100	0	0	0	0	0	0	0	0	0	0	0	0	0	0	0	0
0	0	0	0	100	0	0	0	0	0	0	0	0	0	0	0	0	0	0	0	0
0	0	0	0	100	0	0	0	0	0	0	0	0	0	0	0	0	0	0	0	0
0	0	0	0	100	0	0	0	0	0	0	0	0	0	0	0	0	0	0	0	0
0	0	0	0	100	0	0	0	0	0	0	0	0	0	0	0	0	0	0	0	0
0	0	0	0	100	0	0	0	0	0	0	0	0	0	0	0	0	0	0	0	0
0	0	0	0	100	0	0	0	0	0	0	0	0	0	0	0	0	0	0	0	0
0	0	0	0	100	0	0	0	0	0	0	0	0	0	0	0	0	0	0	0	0
0	0	0	0	100	0	0	0	0	0	0	0	0	0	0	0	0	0	0	0	0
0	0	0	0	100	0	0	0	0	0	0	0	0	0	0	0	0	0	0	0	0
0	0	0	0	100	0	0	0	0	0	0	0	0	0	0	0	0	0	0	0	0
0	0	0	0	100	0	0	0	0	0	0	0	0	0	0	0	0	0	0	0	0
0	0	0	0	100	0	0	0	0	0	0	0	0	0	0	0	0	0	0	0	0
0	0	0	0	100	0	0	0	0	0	0	0	0	0	0	0	0	0	0	0	0
0	0	0	0	100	0	0	0	0	0	0	0	0	0	0	0	0	0	0	0	0
0	0	0	0	100	0	0	0	0	0	0	0	0	0	0	0	0	0	0	0	0
0	0	0	0	100	0	0	0	0	0	0	0	0	0	0	0	0	0	0	0	0
0	0	0	0	100	0	0	0	0	0	0	0	0	0	0	0	0	0	0	0	0
0	0	0	0	100	0	0	0	0	0	0	0	0	0	0	0	0	0	0	0	0
0	0	0	0	100	0	0	0	0	0	0	0	0	0	0	0	0	0	0	0	0
0	0	0	0	100	0	0	0	0	0	0	0	0	0	0	0	0	0	0	0	0
0	0	0	0	100	0	0	0	0	0	0	0	0	0	0	0	0	0	0	0	0
0	0	0	0	100	0	0	0	0	0	0	0	0	0	0	0	0	0	0	0	0
0	0	0	0	100	0	0	0	0	0	0	0	0	0	0	0	0	0	0	0	0
0	0	0	0	100	0	0	0	0	0	0	0	0	0	0	0	0	0	0	0	0
0	0	0	0	100	0	0	0	0	0	0	0	0	0	0	0	0	0	0	0	0
0	0	0	0	100	0	0	0	0	0	0	0	0	0	0	0	0	0	0	0	0
0	0	0	0	100	0	0	0	0	0	0	0	0	0	0	0	0	0	0	0	0
0	0	0	0	100	0	0	0	0	0	0	0	0	0	0	0	0	0	0	0	0

0	0	0	0	100	0	0	0	0	0	0	0	100	0	0	0	0	100	0	0	0
0	0	0	0	100	0	0	0	0	0	0	0	0	0	0	0	0	0	0	0	0
0	0	0	0	100	0	0	0	0	0	0	100	0	0	0	0	0	0	0	0	0
0	0	0	0	100	0	0	0	0	0	0	0	100	0	0	0	0	100	0	0	0
0	0	0	0	100	0	0	0	0	0	0	100	0	0	0	0	0	0	0	0	0
0	0	0	0	100	0	0	0	100	0	0	0	0	0	0	0	0	0	0	0	0
0	0	0	0	0	0	0	0	100	0	0	0	0	0	0	0	0	0	0	0	0
0	0	0	0	100	0	0	0	100	0	0	0	0	0	0	0	0	0	0	0	0
0	0	0	0	0	0	0	0	100	0	0	0	0	0	0	0	0	0	0	0	0
0	0	0	0	0	0	0	0	100	0	0	0	0	0	0	0	0	0	0	0	0
0	0	0	0	0	0	0	0	0	0	0	0	0	0	0	0	0	0	0	0	0
0	0	0	0	0	0	0	0	0	0	0	0	0	0	0	0	0	0	0	0	0
0	0	0	0	0	0	0	0	100	0	0	0	0	0	0	0	0	0	0	0	0
100	0	0	0	100	0	0	0	100	0	0	0	0	0	0	0	0	0	0	0	0
0	0	0	0	100	0	0	0	0	0	0	0	0	0	0	0	0	0	0	0	0
0	0	0	0	0	0	0	0	0	0	0	0	0	0	0	0	0	0	0	0	0
0	0	0	0	0	0	0	0	100	0	0	0	0	0	0	0	0	0	0	0	0
0	0	0	0	0	0	0	0	0	0	0	0	0	0	0	0	0	0	0	0	0
0	0	0	0	0	0	0	0	0	0	0	0	0	0	0	0	0	0	0	0	0
0	0	0	0	0	0	0	0	100	0	0	0	0	0	0	0	0	0	0	0	0
0	0	0	0	100	0	0	0	100	0	0	0	0	0	0	0	0	0	0	0	0
0	0	0	0	100	0	0	0	0	0	0	0	0	0	100	0	0	100	0	0	97.56
100	0	0	100	300	0	0	0	100	0	0	0	0	0	0	0	0	0	0	0	0
100	0	0	0	100	0	0	0	100	0	0	0	0	0	0	0	0	0	0	0	0
0	0	0	0	0	0	0	0	100	0	0	0	0	0	0	0	0	0	0	0	0
0	0	0	0	100	0	0	0	0	0	0	0	0	0	0	0	97.22	100	0	100	0
0	0	0	0	100	0	0	0	0	0	0	100	0	0	0	0	0	0	0	0	0
0	0	0	0	100	0	0	0	100	0	0	0	0	0	0	0	0	0	0	0	0
0	0	0	0	0	0	0	0	100	0	0	0	0	0	0	0	0	0	0	0	0
0	0	0	0	0	0	0	0	100	0	0	0	0	0	0	0	0	0	0	0	0
0	0	0	0	0	0	0	0	0	0	0	0	0	0	0	0	0	0	0	0	0
0	0	0	0	0	0	0	0	0	0	0	0	0	0	0	0	0	0	0	0	0
0	0	0	0	100	0	0	0	0	0	0	0	0	0	0	0	0	0	0	0	0
0	0	0	0	100	0	0	0	0	0	0	0	0	0	0	0	0	0	0	0	0
0	0	0	0	100	0	0	0	0	0	0	0	0	0	0	0	0	0	0	0	0
0	0	0	0	0	0	0	0	100	0	0	0	0	0	0	0	0	0	0	0	0
0	0	0	0	100	0	0	0	100	0	0	0	0	0	0	0	0	0	0	0	0
0	0	0	0	100	100	0	0	100	0	0	0	0	0	0	0	0	0	0	0	0
0	0	0	0	100	0	0	0	100	0	0	0	0	0	0	0	0	0	0	0	0
0	0	0	0	100	0	0	0	100	0	0	0	0	0	0	0	0	0	0	0	0
0	0	0	0	100	0	0	0	100	0	0	0	0	0	0	0	0	0	0	0	0
0	0	0	0	0	0	0	0	100	0	0	0	0	0	0	0	0	0	0	0	0
0	0	0	0	100	0	0	0	0	0	0	0	0	0	0	0	0	0	0	0	0
0	0	0	0	100	0	0	0	100	0	0	0	0	0	100	0	0	0	0	0	0
0	0	0	0	100	0	0	0	0	0	0	0	0	0	0	0	0	0	0	0	0
0	0	0	0	100	0	0	0	0	0	0	0	0	0	0	0	0	0	0	0	0
0	0	0	0	100	0	0	0	0	0	0	0	0	0	0	0	0	0	0	0	0
0	0	0	0	100	0	0	0	0	0	0	0	0	0	0	0	0	0	0	0	0
0	0	0	0	0	0	0	0	0	0	0	0	0	0	0	0	0	0	0	0	0
0	0	0	0	0	0	0	0	100	0	0	0	0	0	0	0	0	0	0	0	0
0	0	0	0	0	0	0	0	100	0	0	0	0	0	0	0	0	0	0	0	0
0	0	0	0	0	0	0	0	100	0	0	0	0	0	0	0	0	0	0	0	0
0	0	0	0	0	0	0	0	0	0	0	0	0	0	0	0	0	0	0	0	0
0	0	0	0	400	0	0	0	100	78.95	0	0	0	0	0	0	0	0	0	0	0

0	0	0	0	0	0	0	0	100	0	0	0	0	0	0	0	0	0	0	0	0
0	0	0	0	100	0	0	0	0	0	0	0	0	0	0	0	0	0	0	0	0
0	0	0	0	0	0	0	0	100	0	0	0	0	0	0	0	0	0	0	0	0
0	0	0	0	100	0	0	0	100	0	0	0	0	0	0	0	0	0	0	0	0
0	0	0	0	0	0	0	0	100	0	0	0	0	0	0	0	0	0	0	0	0
0	0	0	0	0	0	0	0	100	0	0	0	0	0	0	0	0	0	0	0	0
0	0	0	0	100	0	0	0	0	0	0	0	0	0	0	0	0	0	0	0	0
0	0	0	0	100	0	0	0	0	0	0	0	0	0	0	0	0	0	0	0	0
0	0	0	0	100	0	0	0	0	0	0	0	0	0	0	0	0	0	0	0	0
0	0	0	0	100	0	0	0	0	0	0	0	0	0	0	0	0	0	0	0	0
0	0	0	0	0	0	0	0	100	0	0	0	0	0	0	0	0	0	0	0	0
0	0	0	0	100	0	0	0	100	0	0	0	0	0	0	0	0	0	0	0	0
0	0	0	0	0	0	0	0	100	0	0	0	0	0	0	0	0	0	0	0	0
0	0	0	0	100	0	0	0	100	0	0	0	0	0	0	0	0	0	0	0	0
0	0	0	0	100	0	0	0	0	0	0	0	0	0	0	0	0	0	0	0	0
0	0	0	0	100	0	0	0	100	0	0	0	0	0	0	0	0	0	0	0	0
0	0	0	0	0	0	0	0	0	0	0	0	0	0	0	0	0	0	0	0	0
0	0	0	0	0	0	0	0	0	0	0	0	0	0	0	0	0	0	0	0	0
0	0	0	0	0	0	0	0	0	0	0	0	0	0	0	0	0	0	0	0	0
0	0	0	0	100	0	0	0	0	0	0	0	0	0	0	0	0	0	0	0	0
0	0	0	0	100	0	0	0	0	0	0	0	0	0	0	0	0	0	0	0	0
0	0	0	0	100	0	0	0	0	0	0	0	0	0	0	0	0	0	0	0	0
0	0	0	0	100	0	0	0	0	0	0	0	0	0	0	0	0	0	0	0	0
0	0	0	0	0	0	0	0	0	0	0	0	0	0	0	0	0	0	0	0	0
0	0	0	0	0	0	0	0	100	0	0	0	0	0	0	0	0	0	0	0	0
0	0	0	0	100	0	0	0	0	0	0	0	0	0	0	0	0	100	0	0	0
0	0	0	0	0	0	0	0	100	0	0	0	0	0	0	0	0	0	0	0	0
0	0	0	0	0	0	0	0	100	0	0	0	0	0	0	0	0	0	0	0	0
0	0	0	0	100	0	0	0	100	0	0	0	0	0	0	0	0	0	0	0	0
0	0	0	0	0	0	0	0	100	0	0	0	0	0	0	0	0	0	0	0	0
0	0	0	0	100	0	0	0	100	78.95	0	0	0	0	0	0	0	0	0	0	0
0	0	0	0	100	0	0	0	0	0	0	0	0	0	0	0	0	0	0	0	0
0	0	0	0	0	0	0	0	100	0	0	0	0	0	0	0	0	0	0	0	0
100	0	0	0	100	0	0	0	100	0	0	0	0	0	0	0	0	0	0	0	0
0	0	0	0	0	0	0	0	100	0	0	0	0	0	0	0	0	0	0	0	0
0	0	0	0	100	0	0	0	100	0	0	0	0	0	0	0	0	0	0	0	0
0	0	0	0	100	0	0	0	100	0	0	0	0	0	0	0	0	0	0	0	0
0	0	0	0	0	0	0	0	0	0	0	0	0	0	0	0	0	0	0	0	0
0	0	0	0	100	0	0	0	100	78.95	0	0	0	0	0	0	0	0	0	0	0
0	0	0	0	0	0	0	0	100	0	0	0	0	0	0	0	0	0	0	0	0
0	0	0	0	100	0	0	0	0	0	0	0	0	0	0	0	0	0	0	0	0
0	0	0	0	100	0	0	0	100	0	0	0	0	0	0	0	0	0	0	0	0
0	0	0	0	0	0	0	0	100	0	0	0	0	0	0	0	0	0	0	0	0
0	0	0	0	0	0	0	0	0	0	0	0	0	0	0	0	0	0	0	0	0
0	0	0	0	100	0	0	0	0	0	0	0	0	0	0	0	0	0	0	0	0
0	0	0	0	100	0	0	0	0	0	0	0	0	0	0	0	0	0	0	0	0
0	0	0	0	0	0	0	0	100	0	0	0	0	0	0	0	0	0	0	0	0
0	0	0	0	100	0	0	0	0	0	0	0	0	0	0	0	0	0	0	0	0
0	0	0	0	0	0	0	0	0	0	0	0	0	0	0	0	0	0	0	0	0
0	0	0	0	0	0	0	0	99.65	0	0	0	0	0	0	0	0	0	0	0	0
0	0	0	0	100	0	0	0	0	0	0	0	0	0	0	0	0	0	0	0	0
0	0	0	0	0	0	0	0	0	0	0	0	0	0	0	0	0	0	0	0	0
0	0	0	0	100	0	0	0	100	0	0	0	0	0	0	0	0	0	0	0	0
0	0	0	0	100	0	0	0	0	0	0	0	0	0	0	0	0	0	0	0	0

0	0	0	0	0	0	0	0	100	0	0	0	0	0	0	0	0	0	0	0	0
0	0	0	0	0	0	0	0	100	0	0	0	0	0	0	0	0	0	0	0	0
0	0	0	0	0	0	0	0	100	0	0	0	0	0	0	0	0	0	0	0	0
0	0	0	0	100	0	0	0	100	0	0	0	0	0	0	0	0	0	0	0	0
0	0	0	0	100	0	0	0	100	0	0	0	0	0	0	0	0	0	0	0	0
0	0	0	0	0	0	0	0	100	0	0	0	0	0	0	0	0	0	0	0	0
0	0	0	0	100	0	0	0	100	78.95	0	0	0	0	0	0	0	0	0	0	0
0	0	0	0	0	0	0	0	100	0	0	0	0	0	0	0	0	0	0	0	0
0	0	0	0	0	0	0	0	0	0	0	0	0	0	0	0	0	0	0	0	0
0	0	0	0	100	0	0	0	0	0	0	0	0	0	0	0	0	0	0	0	0
0	0	0	0	100	0	0	0	100	0	0	0	0	0	0	0	0	0	0	0	0
0	0	0	0	0	0	0	0	0	0	0	0	0	0	0	0	0	0	0	0	0
0	0	0	0	100	0	0	0	0	0	0	0	0	0	0	0	0	0	0	0	0
0	0	0	0	0	0	0	0	100	0	0	0	0	0	0	0	0	0	0	0	0
0	0	0	0	0	0	0	0	100	0	0	0	0	0	0	0	0	0	0	0	0
0	0	0	0	100	0	0	0	100	0	0	0	0	0	0	0	0	0	0	0	0
0	0	0	0	100	0	0	0	0	0	0	0	0	0	0	0	0	0	0	0	0
0	0	0	0	0	0	0	0	0	0	0	0	0	0	0	0	0	0	0	0	0
0	0	0	0	100	0	0	0	0	0	0	0	0	0	0	0	0	0	0	0	0
0	0	0	0	100	0	0	0	0	0	0	0	0	0	0	0	0	0	0	0	0
0	0	0	0	0	0	0	0	100	0	0	0	0	0	0	0	0	0	0	0	0
0	0	0	0	100	0	0	0	100	0	0	0	0	0	0	0	0	0	0	0	0
0	0	0	0	100	0	0	0	100	0	0	0	0	0	0	0	0	0	0	0	0
0	0	0	0	0	0	0	0	100	0	0	0	0	0	0	0	0	0	0	0	0
0	0	0	0	100	0	0	0	100	78.95	0	0	0	0	0	0	0	0	0	0	0
0	0	0	0	0	0	0	0	100	0	0	0	0	0	0	0	0	0	0	0	0
0	0	0	0	100	0	0	0	100	0	0	0	0	0	0	0	0	0	0	0	0
0	0	0	0	100	0	0	0	0	0	0	0	0	0	0	0	0	0	0	0	0
0	0	0	0	100	0	0	0	0	0	0	0	0	0	0	0	0	0	0	0	0
0	0	0	0	200	0	0	0	100	0	0	0	0	0	0	0	0	0	0	0	0
0	0	0	0	100	0	0	0	100	0	0	0	0	0	0	0	0	0	0	0	0
0	0	0	0	0	0	0	0	0	0	0	0	0	0	0	0	0	0	0	0	0
0	0	0	0	0	0	0	0	0	0	0	0	0	0	0	0	0	0	0	0	0
0	0	0	0	0	0	0	0	100	0	0	0	0	0	0	0	0	0	0	0	0
0	0	0	0	100	0	0	0	0	0	0	0	0	0	0	0	0	0	0	0	0
0	0	0	0	100	0	0	0	100	0	0	0	0	0	0	0	0	0	0	0	0
0	0	0	0	0	0	0	0	0	0	0	0	0	0	0	0	0	0	0	0	0
0	0	0	0	0	0	0	0	100	0	0	0	0	0	0	0	0	0	0	0	0
0	0	0	0	100	0	0	0	100	78.95	0	0	0	0	0	0	0	0	0	0	0
0	0	0	0	100	0	0	0	0	0	0	0	0	0	0	0	0	0	0	0	0
0	0	0	0	200	0	0	0	0	0	0	0	0	0	0	0	0	0	0	0	0
0	0	0	0	100	0	0	0	100	0	0	0	0	0	0	0	0	0	0	0	0
100	0	0	0	100	0	0	0	0	0	0	0	0	0	0	0	0	0	0	0	0
100	0	0	0	200	0	0	0	0	0	0	0	0	0	0	0	0	0	0	0	0
0	0	0	0	100	0	0	0	100	0	0	0	0	0	0	0	0	0	0	0	0
0	0	0	0	100	0	0	0	0	0	0	0	0	0	0	0	0	0	0	0	0
0	0	0	0	100	0	0	0	0	0	0	0	0	0	0	0	0	0	0	0	0
0	0	0	0	200	0	0	0	100	0	0	0	0	0	0	0	0	0	0	0	0
0	0	0	0	100	0	0	0	0	0	0	0	0	0	0	0	0	0	0	0	0
100	0	0	0	100	0	0	0	100	0	0	0	0	0	0	0	0	0	0	0	0
100	0	0	0	100	0	0	0	100	0	0	0	0	0	0	0	0	0	0	0	0
0	0	0	0	100	0	0	0	100	0	0	0	0	0	0	0	0	0	0	0	0
0	0	0	0	100	0	0	0	100	0	0	0	0	0	0	0	0	0	0	0	0
0	0	0	0	100	0	0	0	100	78.95	0	0	0	0	0	0	0	0	0	0	0

0	0	0	0	100	0	0	0	100	78.95	0	0	0	0	0	0	0	0	0	0	0
0	0	0	0	0	0	0	0	100	0	0	0	0	0	0	0	0	0	0	0	0
0	0	0	0	100	0	0	0	100	78.95	0	0	0	0	0	0	0	0	0	0	0
0	0	0	0	200	0	0	0	0	0	0	0	0	0	0	0	0	0	0	0	0
0	0	0	0	0	0	0	0	0	0	0	0	0	0	0	0	0	0	0	0	0
0	0	0	0	100	0	0	0	0	0	0	0	0	0	0	0	0	0	0	0	0
0	0	0	0	0	0	0	0	0	0	0	0	0	0	0	0	0	0	0	0	0
0	0	0	0	0	0	0	0	0	0	0	0	0	0	0	0	0	0	0	0	0
0	100	0	0	100	0	0	0	100	0	0	0	0	0	0	0	0	0	0	0	0
0	0	0	0	0	0	0	0	0	0	0	0	0	0	0	0	0	0	0	0	0
0	0	0	0	0	0	0	0	100	0	0	0	0	0	0	0	0	0	0	0	0
0	0	0	0	100	0	0	0	100	0	0	0	0	0	0	0	0	0	0	0	0
0	0	0	0	0	0	0	0	0	0	0	0	0	0	0	0	0	0	0	0	0
0	0	0	0	0	0	0	0	100	0	0	0	0	0	0	0	0	0	0	0	0
0	0	0	0	100	0	0	0	0	0	0	0	0	0	0	0	0	0	0	0	0
0	0	0	0	100	0	0	0	0	0	0	0	0	0	0	0	0	0	0	0	0
0	0	0	0	0	0	0	0	0	0	0	0	0	0	0	0	0	0	0	0	0
0	0	0	0	100	0	0	0	100	78.95	0	0	0	0	0	0	0	0	0	0	0
100	0	0	0	100	0	0	0	100	0	0	0	0	0	0	0	0	0	0	0	0
0	0	0	0	0	0	0	0	100	0	0	0	0	0	0	0	0	0	0	0	0
0	0	0	0	0	0	0	0	0	0	0	0	0	0	0	0	0	0	0	0	0
0	0	0	0	200	0	0	0	0	0	0	0	0	0	0	0	0	0	0	0	0
0	0	0	0	0	0	0	0	100	0	0	0	0	0	0	0	0	0	0	0	0
0	0	0	0	100	0	0	0	100	0	0	0	0	0	0	0	0	0	0	0	0
0	0	0	0	100	0	0	0	0	0	0	0	0	0	0	0	0	0	0	0	0
0	0	0	0	100	0	0	0	100	0	0	0	0	0	0	0	0	0	0	0	0
0	0	0	0	100	0	0	0	0	0	0	0	0	0	0	83.16	0	0	0	0	0
0	0	100	0	200	0	0	0	0	0	0	0	0	0	0	0	0	0	0	0	0
0	0	0	0	0	0	0	0	100	0	0	0	0	0	0	0	0	0	0	0	0
0	0	0	0	0	0	0	0	0	0	0	0	0	0	0	0	0	0	0	0	0
0	0	0	0	100	0	0	0	100	0	0	0	0	0	0	0	0	0	0	0	0
0	0	0	0	100	0	0	0	0	0	0	0	0	0	0	0	97.22	100	0	100	0
0	0	0	0	100	0	0	0	100	0	0	0	0	0	0	0	0	0	0	0	0
0	0	0	0	100	0	0	0	0	0	0	0	0	0	0	0	0	0	0	0	0
0	0	0	0	100	0	0	0	100	0	0	0	0	0	0	0	0	0	0	0	0
0	0	0	0	100	0	0	0	100	0	0	0	0	0	0	0	0	0	0	0	0
0	0	0	0	100	0	0	0	100	0	0	0	0	0	0	0	0	0	0	0	0
0	0	0	0	100	0	0	0	100	0	0	0	0	100	0	0	97.22	0	0	0	0
0	0	0	0	600	0	0	0	0	0	0	0	0	0	0	0	0	0	99.59	0	0
0	0	0	0	100	0	0	0	0	0	0	0	0	0	0	0	0	0	0	0	0
0	0	0	0	100	0	0	0	100	0	0	0	0	0	0	0	0	0	0	0	0
100	0	0	0	500	0	0	0	0	0	0	100	0	0	0	0	0	0	0	0	0
0	0	0	0	100	0	0	0	0	0	0	0	0	0	0	0	0	0	0	0	0
0	0	0	0	100	0	0	0	0	0	0	0	0	0	0	0	0	0	0	0	0
0	0	0	0	100	0	0	0	0	0	0	0	0	0	0	0	0	0	0	0	0
0	0	0	0	100	0	0	0	100	0	0	0	0	0	0	0	0	0	0	0	0
0	0	0	0	0	0	0	0	100	0	0	0	0	0	0	0	0	0	0	0	0
0	0	0	0	100	0	0	0	0	0	0	0	0	0	0	0	0	0	0	0	0
0	0	0	0	0	0	0	0	0	0	0	0	0	0	0	0	0	0	0	0	0
0	0	0	0	100	0	0	0	100	0	0	0	0	0	0	0	0	0	0	0	0
0	0	0	0	100	0	0	0	0	0	0	0	0	0	0	0	0	0	0	0	0
0	0	0	0	300	0	0	0	0	0	0	100	0	0	0	0	0	0	0	0	0
0	0	0	0	100	0	0	0	0	0	0	0	0	0	0	0	0	0	0	0	0
0	0	0	0	100	0	0	0	100	0	0	0	0	0	0	0	0	0	0	0	0

0	0	0	0	100	0	0	0	100	0	0	0	0	0	0	0	0	0	0	0	0
0	0	0	0	100	0	0	0	100	0	0	0	0	0	0	0	0	0	0	0	0
0	0	0	0	0	0	0	0	0	0	0	0	0	0	0	0	0	0	0	0	0
0	0	0	0	100	0	0	0	100	0	0	0	0	0	0	0	0	0	0	0	0
0	0	0	0	100	0	0	0	100	0	0	0	0	0	0	0	0	0	0	0	0
0	0	0	0	100	0	0	0	0	0	0	0	0	0	0	0	0	0	0	0	0
0	0	0	0	100	0	0	0	0	0	0	0	0	0	0	0	0	0	0	0	0
0	0	0	0	100	0	0	0	0	0	0	0	0	0	0	0	0	0	0	0	0
0	0	0	0	100	0	0	0	0	0	0	0	0	0	0	0	0	0	0	0	0
0	0	0	0	100	0	0	0	0	0	0	0	0	0	0	0	0	0	0	0	0
0	0	0	0	100	0	0	0	100	0	0	0	0	0	0	0	0	0	0	0	0
0	0	0	0	100	0	0	0	100	0	0	0	0	0	0	0	0	0	0	0	0
0	0	0	0	100	0	0	0	0	0	0	0	0	0	0	0	0	0	0	0	0
0	0	0	0	100	0	0	0	0	0	0	0	0	0	0	0	0	0	0	0	0
0	0	0	0	100	0	0	0	100	0	0	0	0	0	0	0	0	0	0	0	0
0	0	0	0	100	0	0	0	0	0	0	0	0	0	0	0	0	0	0	0	0
0	0	0	0	100	0	0	0	100	0	0	0	0	0	0	0	0	0	0	0	0
0	0	0	0	100	0	0	0	100	0	0	0	0	0	0	0	0	0	0	0	0
0	0	0	0	100	0	0	0	100	0	0	0	0	0	0	0	0	0	0	0	0
0	0	0	0	100	0	0	0	0	0	0	0	0	0	0	0	0	0	0	0	0
0	0	0	0	100	0	0	0	0	0	0	0	0	0	0	0	0	0	0	0	0
0	0	0	0	100	0	0	0	100	0	0	0	0	0	0	0	0	0	0	0	0
0	0	0	0	100	0	0	0	100	0	0	0	0	0	0	0	0	0	0	0	0
0	0	0	0	100	0	0	0	0	0	0	0	0	0	0	0	0	0	0	0	0
0	0	0	0	100	0	0	0	100	0	0	0	0	0	0	0	0	0	0	0	0
0	0	0	0	100	0	0	0	0	0	0	0	0	0	0	0	0	0	0	0	0
0	0	0	0	100	0	0	0	0	0	0	0	0	0	0	0	0	0	0	0	0
0	0	0	0	0	0	0	0	100	0	0	0	0	0	0	0	0	0	0	0	0
0	0	0	0	100	0	0	0	0	0	0	100	0	0	0	0	0	0	0	0	0
0	0	0	0	100	0	0	0	100	0	0	0	0	0	0	0	0	0	0	0	0
0	0	0	0	100	0	0	0	100	0	0	0	0	0	0	0	0	0	0	0	0
0	0	0	0	100	0	0	0	0	0	0	0	0	0	0	0	0	0	0	0	0
0	0	0	0	0	0	0	0	100	0	0	0	0	0	0	0	0	0	0	0	0
0	0	0	0	100	0	0	0	0	0	0	100	0	0	0	0	0	0	0	0	0
0	0	0	0	100	0	0	0	100	0	0	0	0	0	0	0	0	0	0	0	0
0	0	0	0	0	0	0	0	100	0	0	0	0	0	0	0	0	0	0	0	0
0	0	0	0	0	0	0	0	100	0	0	0	0	0	0	0	0	0	0	0	0
0	0	0	0	0	0	0	0	100	0	0	0	0	0	0	0	0	0	0	0	0
0	0	0	0	0	0	0	0	100	0	0	0	0	0	0	0	0	0	0	0	0
0	0	0	0	0	0	0	0	100	0	0	0	0	0	0	0	0	0	0	0	0
0	0	0	0	100	0	0	0	0	0	0	100	0	0	0	0	0	0	0	0	0
0	0	0	0	100	0	0	0	100	0	0	0	0	0	0	0	0	0	0	0	0
0	0	0	0	0	0	0	0	100	0	0	0	0	0	0	0	0	0	0	0	0
0	0	0	0	0	0	0	0	100	0	0	0	0	0	0	0	0	0	0	0	0
0	0	0	0	100	0	0	0	100	0	0	0	0	0	0	0	0	0	0	0	0
0	0	0	0	0	0	0	0	100	0	0	0	0	0	0	0	0	0	0	0	0
0	0	0	0	0	0	0	0	100	0	0	0	0	0	0	0	0	0	0	0	0
0	0	0	0	100	0	0	0	100	0	0	0	0	0	0	0	0	0	0	0	0
0	0	0	0	100	0	0	0	100	0	0	0	0	0	0	0	0	0	0	0	0
0	100	0	0	100	0	0	0	100	0	0	0	0	0	0	0	0	0	0	0	0
0	0	0	0	100	0	0	0	100	0	0	0	0	0	0	0	0	0	0	0	0
0	0	0	0	0	0	0	0	100	0	0	0	0	0	0	0	0	0	0	0	0
0	0	0	0	100	0	0	0	100	0	0	0	0	0	0	0	0	0	0	0	0
0	0	0	0	100	0	0	0	100	0	0	0	0	0	0	0	0	0	0	0	0

0	0	0	0	100	0	0	0	0	0	0	0	0	0	0	0	0	0	0	0	0
0	0	0	0	100	0	0	0	100	0	0	0	0	0	0	0	0	0	0	0	0
0	0	0	0	100	0	0	0	0	0	0	0	0	0	0	0	0	0	0	0	0
0	0	0	0	100	0	0	0	0	0	0	0	0	0	0	0	0	0	0	0	0
0	0	0	0	100	0	0	0	100	0	0	0	0	0	0	0	0	0	0	0	0
0	0	0	0	100	0	0	0	100	0	0	0	0	0	0	0	0	0	0	0	0
0	0	0	0	100	0	0	0	100	0	0	0	0	0	0	0	0	0	0	0	0
0	0	0	0	100	0	0	0	0	0	0	0	0	0	0	0	0	0	0	0	0
0	0	0	0	100	0	0	0	0	0	0	0	0	0	0	0	0	0	0	0	0
0	0	0	0	100	0	0	0	0	0	0	0	0	0	0	0	0	0	0	0	0
0	0	0	0	100	0	0	0	0	0	0	0	0	0	0	0	0	0	0	0	0
0	0	0	0	100	0	0	0	0	0	0	100	0	0	0	0	0	0	0	0	0
0	0	0	0	100	0	0	0	100	0	0	0	0	0	0	0	0	0	0	0	0
0	0	0	0	100	0	0	0	100	0	0	0	0	0	0	0	0	0	0	0	0
0	0	0	0	100	0	0	0	100	0	0	0	0	0	0	0	0	0	0	0	0
0	0	0	0	100	0	0	0	100	0	0	0	0	0	0	0	0	0	0	0	0
0	0	0	0	100	0	0	0	100	0	0	0	0	0	0	0	0	0	0	0	0
0	0	0	0	100	0	0	0	100	0	0	0	0	0	0	0	0	0	0	0	0
0	0	0	0	100	0	0	0	100	0	0	0	0	0	0	0	0	0	0	0	0
0	0	0	0	100	0	0	0	0	0	0	0	0	0	0	0	0	0	0	0	0
0	0	0	0	100	0	0	0	0	0	0	0	0	0	0	0	0	0	0	0	0
0	0	0	0	200	0	0	0	0	0	0	0	0	0	0	83.16	0	0	0	0	0
0	0	0	0	0	0	0	0	100	0	0	0	0	0	0	0	0	0	0	0	0
0	0	0	0	100	0	0	0	100	0	0	0	0	0	0	0	0	0	0	0	0
0	0	0	0	100	0	0	0	0	0	0	0	0	0	0	0	97.22	100	0	100	0
0	0	0	0	100	0	0	0	100	0	0	0	0	0	0	0	0	0	0	0	0
0	0	0	0	0	0	0	0	100	0	0	0	0	0	0	0	0	0	0	0	0
0	0	0	0	0	0	0	0	100	0	0	0	0	0	0	0	0	0	0	0	0
0	0	0	0	100	0	0	0	0	0	0	0	0	0	0	0	0	0	0	0	0
0	0	0	0	0	0	0	0	100	0	0	0	0	0	0	0	0	0	0	0	0
0	0	0	0	100	0	0	0	0	0	0	0	0	0	0	0	0	0	0	0	0
0	0	0	0	0	0	0	0	0	0	0	0	0	0	0	0	0	0	0	0	0
0	0	0	0	0	0	0	0	100	0	0	0	0	0	0	0	0	0	0	0	0
0	0	0	0	100	0	0	0	100	0	0	0	0	0	0	0	0	0	0	0	0
0	0	0	0	0	0	0	0	0	0	0	0	0	0	0	0	0	0	0	0	0
0	0	0	0	100	0	0	0	0	0	0	0	0	0	0	0	0	0	0	0	0
0	0	0	0	100	0	0	0	0	0	0	0	0	0	0	0	0	0	0	0	0
0	0	0	0	100	0	0	0	0	0	0	0	0	0	0	0	0	0	0	0	0
0	0	0	0	100	0	0	0	0	0	0	0	0	0	0	0	0	0	0	0	0
0	0	0	0	100	0	0	0	0	0	0	0	0	0	0	0	0	0	0	0	0
0	0	0	0	100	0	0	0	0	0	0	0	0	0	0	0	0	0	0	0	0
0	0	0	0	100	0	0	0	0	0	0	0	0	0	0	0	0	0	0	0	0
0	0	0	0	0	0	0	0	100	0	0	0	0	0	0	0	0	0	0	0	0
0	0	0	0	0	0	0	0	0	0	0	0	0	0	0	0	0	0	0	0	0
0	0	0	0	0	0	0	0	0	0	0	0	0	0	0	0	0	0	0	0	0
0	0	0	0	100	0	0	0	0	0	0	100	0	0	0	0	0	0	0	0	0
0	0	0	0	100	0	0	0	0	0	0	0	0	0	0	0	0	0	0	0	0
0	0	0	0	0	0	100	0	0	0	0	0	0	0	0	0	0	0	0	0	0
0	0	0	0	0	0	0	0	100	0	0	0	0	0	0	0	0	0	0	0	0
0	0	0	0	0	0	0	0	100	0	0	0	0	0	0	0	0	0	0	0	0
0	0	0	0	0	0	0	0	0	0	0	0	0	0	0	0	0	0	0	0	0
0	0	0	0	100	0	0	0	0	0	0	0	0	0	0	0	0	0	0	0	0
0	0	0	0	100	0	0	0	100	0	0	0	0	0	0	0	0	0	0	0	0
0	0	0	0	100	0	0	0	100	0	0	0	0	0	0	0	0	0	0	0	0

0	0	0	0	100	0	0	0	0	0	0	0	0	0	0	0	97.22	100	0	100	0
0	0	0	0	0	0	0	0	100	0	0	0	0	0	0	0	0	0	0	0	0
0	0	0	0	0	0	0	0	100	0	0	0	0	0	0	0	0	0	0	0	0
0	0	0	0	0	0	0	0	0	0	0	0	0	0	0	0	0	0	0	0	0
0	0	0	0	100	0	0	0	0	0	0	0	0	0	0	0	0	0	0	0	0
0	0	0	0	0	0	0	0	100	0	0	0	0	0	0	0	0	0	0	0	0
0	0	0	0	0	0	0	0	100	0	0	0	0	0	0	0	0	0	0	0	0
0	0	0	0	100	0	0	0	0	0	0	100	0	0	0	0	0	0	0	0	0
0	0	0	0	0	0	0	0	0	0	0	0	0	0	0	0	0	0	0	0	0
100	0	0	0	200	0	0	0	0	0	0	0	0	0	0	0	0	0	0	0	0
0	0	0	0	100	0	0	0	0	0	0	0	0	0	0	0	0	0	0	0	0
0	0	0	0	500	0	0	0	0	0	0	100	0	0	0	0	0	0	0	0	0
0	0	0	0	100	0	0	0	0	0	0	0	0	0	0	0	0	0	0	0	0
0	0	0	0	0	0	0	0	0	0	0	0	0	0	0	0	0	0	0	0	0
0	0	0	0	0	0	0	0	100	0	0	0	0	0	0	0	0	0	0	0	0
0	0	0	0	0	0	0	0	100	0	0	0	0	0	0	0	0	0	0	0	0
0	0	0	0	100	0	0	0	0	0	0	0	0	0	0	0	0	0	0	0	0
0	0	0	0	0	0	0	0	100	0	0	0	0	0	0	0	0	0	0	0	0
0	0	0	0	0	0	0	0	100	0	0	0	0	0	0	0	0	0	0	0	0
0	0	0	0	0	0	0	0	100	0	0	0	0	0	0	0	0	0	0	0	0
0	0	0	0	100	0	0	0	100	0	0	0	0	0	0	0	0	0	0	0	0
0	0	0	0	0	0	0	0	100	0	0	0	0	0	0	0	0	0	0	0	0
0	0	0	0	0	0	0	0	100	0	0	0	0	0	0	0	0	0	0	0	0
0	0	0	0	0	0	0	0	100	0	0	0	0	0	0	0	0	0	0	0	0
0	0	0	0	100	0	0	0	0	0	0	0	0	0	0	0	0	0	0	0	0
0	0	0	0	0	0	0	0	100	0	0	0	0	0	0	0	0	0	0	0	0
0	0	0	0	100	0	0	0	0	0	0	0	0	0	0	0	0	0	0	0	0
0	0	0	0	100	0	0	0	0	0	0	0	0	0	0	0	0	0	0	0	0
0	0	0	0	100	0	0	0	0	0	0	0	0	0	0	0	0	0	0	0	0
0	0	0	0	100	0	0	0	0	0	0	0	0	0	0	0	0	0	0	0	0
0	0	0	0	100	0	0	0	0	0	0	0	0	0	0	0	0	0	0	0	0
0	0	0	0	100	0	0	0	0	0	0	0	0	0	0	0	0	0	0	0	0
0	0	0	0	100	0	0	0	0	0	0	0	0	0	0	0	0	0	0	0	0
0	0	0	0	100	0	0	0	0	0	0	0	0	0	0	0	0	0	0	0	0
0	0	0	0	100	0	0	0	0	0	0	0	0	0	0	0	0	0	0	0	0
0	0	0	0	100	0	0	0	0	0	0	0	0	0	0	0	0	0	0	0	0
0	0	0	0	100	0	0	0	0	0	0	0	0	0	0	0	0	0	0	0	0
0	0	0	0	100	0	0	0	0	0	0	0	0	0	0	0	0	0	0	0	0
0	0	0	0	100	0	0	0	0	0	0	0	0	0	0	0	0	0	0	0	0
0	0	0	0	100	0	0	0	0	0	0	0	0	0	0	0	0	0	0	0	0
0	0	0	0	100	0	0	0	0	0	0	0	0	0	0	0	0	0	0	0	0
0	0	0	0	100	0	0	0	0	0	0	0	0	0	0	0	0	0	0	0	0
0	0	0	0	100	0	0	0	0	0	0	0	0	0	0	0	0	0	0	0	0
0	0	0	0	100	0	0	0	0	0	0	0	0	0	0	0	0	0	0	0	0
0	0	0	0	100	0	0	0	0	0	0	0	0	0	0	0	0	0	0	0	0
0	0	0	0	100	0	0	0	0	0	0	0	0	0	0	0	0	0	0	0	0
0	0	0	0	100	0	0	0	0	0	0	0	0	0	0	0	0	0	0	0	0
0	0	0	0	100	0	0	0	0	0	0	0	0	0	0	0	0	0	0	0	0
0	0	0	0	100	0	0	0	0	0	0	0	0	0	0	0	0	0	0	0	0
0	0	0	0	100	0	0	0	0	0	0	0	0	0	0	0	0	0	0	0	0
0	0	0	0	100	0	0	0	0	0	0	0	0	0	0	0	0	0	0	0	0
0	0	0	0	100	0	0	0	0	0	0	0	0	0	0	0	0	0	0	0	0
0	0	0	0	100	0	0	0	0	0	0	0	0	0	0	0	0	0	0	0	0
0	0	0	0	100	0	0	0	0	0	0	0	0	0	0	0	0	0	0	0	0

0	0	0	0	100	0	0	0	0	0	0	0	0	0	0	0	0	0	0	0	0
0	0	0	0	100	0	0	0	0	0	0	0	0	0	0	0	0	0	0	0	0
0	0	0	0	100	0	0	0	0	0	0	0	0	0	0	0	0	0	0	0	0
0	0	0	0	100	0	0	0	0	0	0	0	0	0	0	0	0	0	0	0	0
0	0	0	0	100	0	0	0	0	0	0	0	0	0	0	0	0	0	0	0	0
0	0	0	0	100	0	0	0	0	0	0	0	0	0	0	0	0	0	0	0	0
0	0	0	0	100	0	0	0	0	0	0	0	0	0	0	0	0	0	0	0	0
0	0	0	0	100	0	0	0	0	0	0	0	0	0	0	0	0	0	0	0	0
0	0	0	0	100	0	0	0	0	0	0	0	0	0	0	0	0	0	0	0	0
0	0	0	0	100	0	0	0	0	0	0	0	0	0	0	0	0	0	0	0	0
0	0	0	0	100	0	0	0	0	0	0	0	0	0	0	0	0	0	0	0	0
0	0	0	0	100	0	0	0	0	0	0	0	0	0	0	0	0	0	0	0	0
0	0	0	0	100	0	0	0	0	0	0	0	0	0	0	0	0	0	0	0	0
0	0	0	0	100	0	0	0	0	0	0	0	0	0	0	0	0	0	0	0	0
0	0	0	0	100	0	100	0	0	0	0	0	0	0	0	0	0	0	0	0	0
0	0	0	0	100	0	0	0	0	0	0	0	0	0	0	0	0	0	0	0	0
0	0	0	0	100	0	0	0	0	0	0	0	0	0	0	0	0	0	0	0	0
0	0	0	0	100	0	0	0	0	0	0	0	0	0	0	0	0	0	0	0	0
0	0	0	0	100	0	0	0	0	0	0	0	0	0	0	0	0	0	0	0	0
0	0	0	0	100	0	0	0	0	0	0	0	0	0	0	0	0	0	0	0	0
0	0	0	0	100	0	0	0	0	0	99.6	0	0	0	0	0	0	0	0	0	0
0	0	0	0	100	0	0	0	0	0	0	0	0	0	0	0	0	0	0	0	0
0	0	0	0	100	0	0	0	0	0	0	0	0	0	0	0	0	0	0	0	0
0	0	0	0	100	0	0	0	0	0	0	0	0	0	0	0	0	0	0	0	0
0	0	0	0	100	0	0	0	0	0	0	0	0	0	0	0	0	0	0	0	0
0	0	0	0	100	0	0	0	0	0	0	0	0	0	0	0	0	0	0	0	0
0	0	0	0	100	0	0	0	0	0	0	0	0	0	0	0	0	0	0	0	0
0	0	0	0	100	0	0	0	0	0	0	0	0	0	0	0	0	0	0	0	0
0	0	0	0	100	0	0	0	0	0	0	0	0	0	0	0	0	0	0	0	0
0	0	0	0	100	0	0	0	0	0	0	0	0	0	0	0	0	0	0	0	0
0	0	0	0	100	0	0	0	0	0	0	0	0	0	0	0	0	0	0	0	0
0	0	0	0	100	0	0	0	0	0	0	0	0	0	0	0	0	0	0	0	0
0	0	0	0	100	0	0	0	0	0	0	0	0	0	0	0	0	0	0	0	0
0	0	0	0	100	0	0	0	0	0	0	0	0	0	0	0	0	0	0	0	0
0	0	0	0	100	0	0	0	0	0	0	0	0	0	0	0	0	0	0	0	0
0	0	0	0	100	0	0	0	0	0	0	0	0	0	0	0	0	0	0	0	0
0	0	0	0	100	0	0	0	0	0	0	0	0	0	0	0	0	0	0	0	0
0	0	0	0	100	0	0	0	0	0	0	0	0	0	0	0	0	0	0	0	0
0	0	0	0	100	0	0	0	0	0	0	0	0	0	0	0	0	0	0	0	0
0	0	0	0	100	0	0	0	0	0	0	0	0	0	0	0	0	0	0	0	0
0	0	0	0	100	0	0	0	0	0	0	0	0	0	0	0	0	0	0	0	0
0	0	0	0	100	0	0	0	0	0	0	0	0	0	0	0	0	0	0	0	0
0	0	0	0	100	0	0	0	0	0	0	0	0	0	0	0	0	0	0	0	0
0	0	0	0	100	0	0	0	0	0	0	0	0	0	0	0	0	0	0	0	0
0	0	0	0	100	0	0	0	0	0	0	0	0	0	0	0	0	0	0	0	0
0	0	0	0	100	0	0	0	0	0	0	0	0	0	0	0	0	0	0	0	0
0	0	0	0	100	0	0	0	0	0	0	0	0	0	0	0	0	0	0	0	0
0	0	0	0	100	0	0	0	0	0	0	0	0	0	0	0	0	0	0	0	0
0	0	0	0	100	0	0	0	0	0	0	0	0	0	0	0	0	0	0	0	0
0	0	0	0	100	0	0	0	0	0	0	0	0	0	0	0	0	0	0	0	0
0	0	0	0	100	0	0	0	0	0	0	0	0	0	0	0	0	0	0	0	0
0	0	0	0	100	0	0	0	0	0	0	0	0	0	0	0	0	0	0	0	0
0	0	0	0	100	0	0	0	0	0	0	0	0	0	0	0	0	0	0	0	0
0	0	0	0	100	0	0	0	0	0	0	0	0	0	0	0	0	0	0	0	0

0	0	0	0	100	0	0	0	0	0	0	0	0	0	0	0	0	0	0	0	0
0	0	0	0	100	0	0	0	0	0	0	0	0	0	0	0	0	0	0	0	0
0	0	0	0	100	0	0	0	0	0	0	0	0	0	0	0	0	0	0	0	0
0	0	0	0	100	0	0	0	0	0	0	0	0	0	0	0	0	0	0	0	0
0	0	0	0	100	0	0	0	0	0	0	0	0	0	0	0	0	0	0	0	0
0	0	0	0	100	0	0	0	0	0	0	0	0	0	0	0	0	0	0	0	0
0	0	0	0	100	0	0	0	0	0	0	100	0	0	0	0	0	0	0	0	0
0	0	0	0	0	0	0	0	100	0	0	0	0	0	0	0	0	0	0	0	0
0	0	0	0	0	0	0	0	0	0	0	0	0	0	0	0	0	0	0	0	0
0	0	0	0	100	0	0	0	0	0	0	0	0	0	0	0	0	0	0	0	0
0	0	0	0	100	0	0	0	0	0	0	0	0	0	0	0	0	0	0	0	0
0	0	0	0	100	0	0	0	0	0	0	0	0	0	0	0	0	0	0	0	0
0	0	0	0	100	0	0	0	0	0	0	0	0	0	0	0	0	0	0	0	0
0	0	0	0	100	0	0	0	0	0	0	0	0	0	0	0	0	0	0	0	0
0	0	0	0	100	0	0	0	0	0	0	0	0	0	0	0	0	0	0	0	0
0	0	0	0	100	0	0	0	0	0	0	0	0	0	0	0	0	0	0	0	0
0	0	0	0	100	0	0	0	0	0	0	0	0	0	0	0	0	0	0	0	0
0	0	0	0	100	0	0	0	0	0	0	0	0	0	0	0	0	0	0	0	0
0	0	0	0	100	0	0	0	0	0	0	0	0	0	0	0	0	0	0	0	0
0	0	0	0	100	0	0	0	0	0	0	0	0	0	0	0	0	0	0	0	0
0	0	0	0	100	0	0	0	0	0	0	0	0	0	0	0	0	0	0	0	0
0	0	0	0	100	0	0	0	0	0	0	0	0	0	0	0	0	0	0	0	0
0	0	0	0	100	0	0	0	0	0	0	0	0	0	0	0	0	0	0	0	0
0	0	0	0	100	0	0	0	0	0	0	0	0	0	0	0	0	0	0	0	0
0	0	0	0	100	0	0	0	0	0	0	0	0	0	0	0	0	0	0	0	0
0	0	0	0	100	0	0	0	0	0	0	0	0	0	0	0	0	0	0	0	0
0	0	0	0	100	0	0	0	0	0	0	0	0	0	0	0	0	0	0	0	0
0	0	0	0	100	0	0	0	0	0	0	0	0	0	0	0	0	0	0	0	0
0	0	0	0	100	0	0	0	0	0	0	0	0	0	0	0	0	0	0	0	0
0	0	0	0	100	0	0	0	0	0	0	0	0	0	0	0	0	0	0	0	0
0	0	0	0	100	0	0	0	0	0	0	0	0	0	0	0	0	0	0	0	0
0	0	0	0	100	0	0	0	0	0	0	0	0	0	0	0	0	0	0	0	0
0	0	0	0	100	0	0	0	0	0	0	0	0	0	0	0	0	0	0	0	0
0	0	0	0	100	0	0	0	0	0	0	0	0	0	0	0	0	0	0	0	0
0	0	0	0	100	0	0	0	0	0	0	0	0	0	0	0	0	0	0	0	0
0	0	0	0	100	0	0	0	0	0	0	0	0	0	0	0	0	0	0	0	0
0	0	0	0	100	0	0	0	0	0	0	0	0	0	0	0	0	0	0	0	0
0	0	0	0	100	0	0	0	0	0	0	0	0	0	0	0	0	0	0	0	0
0	0	0	0	100	0	0	0	0	0	0	0	0	0	0	0	0	0	0	0	0
0	0	0	0	100	0	0	0	0	0	0	0	0	0	0	0	0	0	0	0	0
0	0	0	0	100	0	0	0	0	0	0	0	0	0	0	0	0	0	0	0	0
0	0	0	0	100	0	0	0	0	0	0	0	0	0	0	0	0	0	0	0	0
0	0	0	0	100	0	0	0	0	0	0	0	0	0	0	0	0	0	0	0	0
0	0	0	0	100	0	0	0	0	0	0	0	0	0	0	0	0	0	0	0	0
0	0	0	0	100	0	0	0	0	0	0	0	0	0	0	0	0	0	0	0	0
0	0	0	0	100	0	0	0	0	0	0	0	0	0	0	0	0	0	0	0	0
0	0	0	0	100	0	0	0	0	0	0	0	0	0	0	0	0	0	0	0	0
0	0	0	0	100	0	0	0	0	0	0	0	0	0	0	0	0	0	0	0	0
0	0	0	0	100	0	0	0	0	0	0	0	0	0	0	0	0	0	0	0	0
0	0	0	0	100	0	0	0	0	0	0	0	0	0	0	0	0	0	0	0	0
0	0	0	0	100	0	0	0	0	0	0	0	0	0	0	0	0	0	0	0	0
0	0	0	0	100	0	0	0	0	0	0	0	0	0	0	0	0	0	0	0	0
0	0	0	0	100	0	0	0	0	0	0	0	0	0	0	0	0	0	0	0	0
0	0	0	0	100	0	0	0	0	0	0	0	0	0	0	0	0	0	0	0	0

0	0	0	0	100	0	0	0	0	0	0	0	0	0	0	0	0	0	0	0	0
0	0	0	0	100	0	0	0	0	0	0	0	0	0	0	0	0	0	0	0	0
0	0	0	0	100	0	0	0	0	0	0	0	0	0	0	0	0	0	0	0	0
0	0	0	0	0	0	0	0	100	0	0	0	0	0	0	0	0	0	0	0	0
0	0	0	0	0	0	0	0	99.65	0	0	0	0	0	0	0	0	0	0	0	0
0	0	0	0	100	0	0	0	100	0	0	0	0	0	0	0	0	0	0	0	0
0	0	0	0	100	0	0	0	100	0	0	0	0	0	0	0	0	0	0	0	0
0	0	0	0	100	0	0	0	100	0	0	0	0	0	0	0	0	0	0	0	0
0	0	0	0	100	0	0	0	100	0	0	0	0	0	0	0	0	0	0	0	0
0	0	0	0	100	0	0	0	100	0	0	0	0	0	0	0	0	0	0	0	0
0	0	0	0	100	0	0	0	100	0	0	0	0	0	0	0	0	0	0	0	0
0	0	0	0	100	0	0	0	100	0	0	0	0	0	0	0	0	0	0	0	0
0	0	0	0	100	0	0	0	100	0	0	0	0	0	0	0	0	0	0	0	0
0	0	0	0	100	0	0	0	100	0	0	0	0	0	0	0	0	0	0	0	0
0	0	0	0	100	0	0	0	100	0	0	0	0	0	0	0	0	0	0	0	0
0	0	0	0	100	0	0	0	100	0	0	0	0	0	0	0	0	0	0	0	0
0	0	0	0	100	0	0	0	100	0	0	0	0	0	0	0	0	0	0	0	0
0	0	0	0	100	0	0	0	100	0	0	0	0	0	0	0	0	0	0	0	0
0	0	0	0	100	0	0	0	100	0	0	0	0	0	0	0	0	0	0	0	0
0	0	0	0	100	0	0	0	100	0	0	0	0	0	0	0	0	0	0	0	0
0	0	0	0	100	0	0	0	100	0	0	0	0	0	0	0	0	0	0	0	0
0	0	0	0	0	0	0	0	100	0	0	0	0	0	0	0	0	0	0	0	0
0	0	0	0	0	0	0	0	0	0	0	0	0	0	0	0	0	0	0	0	0
0	0	0	0	0	0	0	0	0	0	0	0	0	0	0	0	0	0	0	0	0
0	0	0	0	0	0	0	0	0	0	0	0	0	0	0	0	0	0	0	0	0
0	0	0	0	0	0	0	0	0	0	0	0	0	0	0	0	0	0	0	0	0
0	0	0	0	0	0	0	0	0	0	0	0	0	0	0	0	0	0	0	0	0
0	0	0	0	100	0	0	0	0	0	0	0	0	0	0	0	0	0	0	0	0
0	0	0	0	0	0	0	0	0	0	0	0	0	0	0	0	0	0	0	0	0
0	0	0	0	0	0	0	0	100	0	0	0	0	0	0	0	0	0	0	0	0
0	0	0	0	0	0	0	0	100	0	0	0	0	0	0	0	0	0	0	0	0
0	0	0	0	100	0	0	0	100	0	0	0	0	0	0	0	0	0	0	0	0
0	0	0	0	0	0	0	0	0	0	0	0	0	0	0	0	0	0	0	0	0
0	0	0	0	0	0	0	0	0	0	0	0	0	0	0	0	0	0	0	0	0
0	0	0	0	0	0	0	0	100	0	0	0	0	0	0	0	0	0	0	0	0
0	0	0	0	100	0	0	0	0	0	0	0	0	0	0	0	0	0	0	0	0
0	0	0	0	100	0	0	0	0	0	0	0	0	0	0	0	0	0	0	0	0
0	0	0	0	100	0	0	0	100	0	0	0	0	0	0	0	0	0	0	0	0
0	0	0	0	0	0	0	0	100	0	0	0	0	0	0	0	0	0	0	0	0
0	0	0	0	0	0	0	0	100	0	0	0	0	0	0	0	0	0	0	0	0
0	0	0	0	0	0	0	0	100	0	0	0	0	0	0	0	0	0	0	0	0
0	0	0	0	0	0	0	0	0	0	0	0	0	0	0	0	0	0	0	0	0
0	0	0	0	0	0	0	0	0	0	0	0	0	0	0	0	0	0	0	0	0
0	0	0	0	0	0	0	0	0	0	0	0	0	0	0	0	0	0	0	0	0
0	0	0	0	100	0	0	0	0	0	0	0	0	0	0	0	0	0	0	0	0
0	0	0	0	0	0	0	0	0	0	0	0	0	0	0	0	0	0	0	0	0
0	0	0	0	0	0	0	0	0	0	0	0	0	0	0	0	0	0	0	0	0
0	0	0	0	0	0	0	0	100	0	0	0	0	0	0	0	0	0	0	0	0
0	0	0	0	100	0	0	0	0	0	0	0	0	0	0	0	0	0	0	0	0
0	0	0	0	0	0	0	0	100	0	0	0	0	0	0	0	0	0	0	0	0
0	0	0	0	0	0	0	0	0	0	0	0	0	0	0	0	0	0	0	0	0
0	0	0	0	0	0	0	0	100	0	0	0	0	0	0	0	0	0	0	0	0
0	0	0	0	0	0	0	0	100	0	0	0	0	0	0	0	0	0	0	0	0
0	0	0	0	0	0	0	0	100	0	0	0	0	0	0	0	0	0	0	0	0

0	0	0	0	0	0	0	0	100	0	0	0	0	0	0	0	0	0	0	0	0
0	0	0	0	0	0	0	0	100	0	0	0	0	0	0	0	0	0	0	0	0
0	0	0	0	0	0	0	0	100	0	0	0	0	0	0	0	0	0	0	0	0
0	0	0	0	0	0	0	0	100	0	0	0	0	0	0	0	0	0	0	0	0
0	0	0	0	100	0	0	0	100	0	0	0	0	0	0	0	0	0	0	0	0
0	0	0	0	100	0	0	0	100	0	0	0	0	0	0	0	0	0	0	0	0
0	0	0	0	0	0	0	0	100	0	0	0	0	0	0	0	0	0	0	0	0
0	0	0	0	0	0	0	0	100	0	0	0	0	0	0	0	0	0	0	0	0
0	0	0	0	0	0	0	0	100	0	0	0	0	0	0	0	0	0	0	0	0
0	0	0	0	0	0	0	0	100	0	0	0	0	0	0	0	0	0	0	0	0
0	0	0	0	100	0	0	0	100	0	0	0	0	0	0	0	0	0	0	0	0
0	0	0	0	0	0	0	0	100	0	0	0	0	0	0	0	0	0	0	0	0
0	0	0	0	100	0	0	0	100	0	0	0	0	0	0	0	0	0	0	0	0
0	0	0	0	0	0	0	0	100	0	0	0	0	0	0	0	0	0	0	0	0
0	0	0	0	0	0	0	0	100	0	0	0	0	0	0	0	0	0	0	0	0
0	0	0	0	0	0	0	0	100	0	0	0	0	0	0	0	0	0	0	0	0
0	0	0	0	100	0	0	0	100	0	0	0	0	0	0	0	0	0	0	0	0
0	0	0	0	0	0	0	0	100	0	0	0	0	0	0	0	0	0	0	0	0
0	0	0	0	100	0	0	0	100	0	0	0	0	0	0	0	0	0	0	0	0
0	0	0	0	100	0	0	0	100	0	0	0	0	0	0	0	0	0	0	0	0
0	0	0	0	0	0	0	0	100	0	0	0	0	0	0	0	0	0	0	0	0
0	0	0	0	0	0	0	0	100	0	0	0	0	0	0	0	0	0	0	0	0
0	0	0	0	100	0	0	0	100	0	0	0	0	0	0	0	0	0	0	0	0
0	0	0	0	0	0	0	0	100	0	0	0	0	0	0	0	0	0	0	0	0
0	0	0	0	100	0	0	0	100	0	0	0	0	0	0	0	0	0	0	0	0
0	0	0	0	100	0	0	0	100	0	0	0	0	0	0	0	0	0	0	0	0
0	0	0	0	0	0	0	0	100	0	0	0	0	0	0	0	0	0	0	0	0
0	0	0	0	0	0	0	0	100	0	0	0	0	0	0	0	0	0	0	0	0
0	0	0	0	0	0	0	0	100	0	0	0	0	0	0	0	0	0	0	0	0
0	0	0	0	0	0	0	0	100	0	0	0	0	0	0	0	0	0	0	0	0
0	0	0	0	0	0	0	0	100	0	0	0	0	0	0	0	0	0	0	0	0
0	0	0	0	0	0	0	0	100	0	0	0	0	0	0	0	0	0	0	0	0
0	0	0	0	0	0	0	0	100	0	0	0	0	0	0	0	0	0	0	0	0
0	0	0	0	100	0	0	0	100	0	0	0	0	0	0	0	0	0	0	0	0
0	0	0	0	100	100	0	0	100	0	0	0	0	0	0	0	0	0	0	0	0
0	0	0	0	100	0	0	0	100	0	0	0	0	0	0	0	0	0	0	0	0
0	0	0	0	0	0	0	0	100	0	0	0	0	0	0	0	0	0	0	0	0
0	0	0	0	0	0	0	0	100	0	0	0	0	0	0	0	0	0	0	0	0
0	0	0	0	0	0	0	0	100	0	0	0	0	0	0	0	0	0	0	0	0
0	0	0	0	100	0	0	0	0	0	0	0	0	0	0	0	0	0	0	0	0
0	0	0	0	0	0	0	0	0	0	0	0	0	0	0	0	0	0	0	0	0
0	0	0	0	0	0	0	0	0	0	0	0	0	0	0	0	0	0	0	0	0
0	0	0	0	0	0	0	0	0	0	0	0	0	0	0	0	0	0	0	0	0
0	0	0	0	0	0	0	0	0	0	0	0	0	0	0	0	0	0	0	0	0
0	0	0	0	100	0	0	0	100	0	0	0	0	0	0	0	0	0	0	0	0
0	0	0	0	0	0	0	0	0	0	0	0	0	0	0	0	0	0	0	0	0
0	0	0	0	100	0	0	0	100	0	0	0	0	0	0	0	0	0	0	0	0
0	0	0	0	0	0	0	0	0	0	0	0	0	0	0	0	0	0	0	0	0
0	0	0	0	0	0	0	0	0	0	0	0	0	0	0	0	0	0	0	0	0
0	0	0	0	0	0	0	0	0	0	0	0	0	0	0	0	0	0	0	0	0
0	0	0	0	100	0	0	0	100	0	0	0	0	0	0	0	0	0	0	0	0
0	0	0	0	0	0	0	0	100	0	0	0	0	0	0	0	0	0	0	0	0
0	0	0	0	100	0	0	0	100	0	0	0	0	0	0	0	0	0	0	0	0

0	0	0	0	0	0	0	0	0	0	0	0	0	0	0	0	0	0	0	0	0
0	0	0	0	0	0	0	0	100	0	0	0	0	0	0	0	0	0	0	0	0
0	0	0	0	0	0	0	0	0	0	0	0	0	0	0	0	0	0	0	0	0
0	0	0	0	0	0	0	0	0	0	0	0	0	0	0	0	0	0	0	0	0
0	0	0	0	77.78	0	0	0	0	0	0	0	0	0	0	0	0	0	0	0	0
0	0	0	0	77.78	0	0	0	0	0	0	0	0	0	0	0	0	0	0	0	0
0	0	0	0	0	0	0	0	0	0	0	0	0	0	0	0	0	0	0	0	0
0	0	0	0	0	0	0	0	0	0	0	0	0	0	0	0	0	0	0	0	0
0	0	0	0	0	0	0	0	100	0	0	0	0	0	0	0	0	0	0	0	0
0	0	0	0	0	0	0	0	0	0	0	0	0	0	0	0	0	0	0	0	0
0	0	0	0	0	0	0	0	100	0	0	0	0	0	0	0	0	0	0	0	0
0	0	0	0	0	0	0	0	0	0	0	0	0	0	0	0	0	0	0	0	0
0	0	0	0	0	0	0	0	0	0	0	0	0	0	0	0	0	0	0	0	0
0	0	0	0	0	0	0	0	0	0	0	0	0	0	0	0	0	0	0	0	0
0	0	0	0	0	0	0	0	0	0	0	0	0	0	0	0	0	0	0	0	0
0	0	0	0	0	0	0	0	100	0	0	0	0	0	0	0	0	0	0	0	0
0	0	0	0	0	0	0	0	0	0	0	0	0	0	0	0	0	0	0	0	0
0	0	0	0	0	0	0	0	100	0	0	0	0	0	0	0	0	0	0	0	0
0	0	0	0	0	0	0	0	100	0	0	0	0	0	0	0	0	0	0	0	0
0	0	0	0	0	0	0	0	0	0	0	0	0	0	0	0	0	0	0	0	0
0	0	0	0	0	0	0	0	0	0	0	0	0	0	0	0	0	0	0	0	0
0	0	0	0	77.78	0	0	0	0	0	0	0	0	0	0	0	0	0	0	0	0
0	0	0	0	0	0	0	0	0	0	0	0	0	0	0	0	0	0	0	0	0
0	0	0	0	0	100	0	0	0	0	0	0	0	0	0	0	0	0	0	0	0
0	0	0	0	0	0	0	0	0	0	0	0	0	0	0	0	0	0	0	0	0
0	0	0	0	77.78	0	0	0	0	0	0	0	0	0	0	0	0	0	0	0	0
0	0	0	0	77.78	0	0	0	0	0	0	0	0	0	0	0	0	0	0	0	0
0	0	0	0	77.78	0	0	85.71	0	0	0	0	0	0	0	0	0	0	0	0	0
0	0	0	0	0	0	0	0	0	0	0	0	0	0	0	0	0	0	0	0	0
0	0	0	0	0	0	0	0	0	0	0	0	0	0	0	0	0	0	0	0	0
0	0	0	0	77.78	0	0	0	0	0	0	0	0	0	0	0	0	0	0	0	0
0	0	0	0	0	0	0	0	0	0	0	0	0	0	0	0	0	0	0	0	0
0	0	0	0	0	0	0	0	0	0	0	0	0	0	0	0	0	0	0	0	0
0	0	0	0	0	0	0	0	0	0	0	0	0	0	0	0	0	0	0	0	0
0	0	0	0	0	0	0	0	0	0	0	0	0	0	0	0	0	0	0	0	0
0	0	0	0	0	0	0	0	0	0	0	0	0	0	0	0	0	0	0	0	0
0	0	0	0	0	0	0	0	0	0	0	0	0	0	0	0	0	0	0	0	0
0	0	0	0	0	0	0	0	0	0	0	0	0	0	0	0	0	0	0	0	0
0	0	0	0	0	0	0	0	0	0	0	0	0	0	0	0	0	0	0	0	0
0	0	0	0	0	0	0	0	0	0	0	0	0	0	0	0	0	0	0	0	0
0	0	0	0	0	0	0	0	0	0	0	0	0	0	0	0	0	0	0	0	0
0	0	0	0	0	0	0	0	0	0	0	0	0	0	0	0	0	0	0	0	0
0	0	0	0	0	0	0	0	0	0	0	0	0	0	0	0	0	0	0	0	0
0	0	0	0	0	0	0	0	0	0	0	0	0	0	0	0	0	0	0	0	0
0	0	0	0	0	0	0	0	0	0	0	0	0	0	0	0	0	0	0	0	0
0	0	0	0	0	0	0	0	0	0	0	0	0	0	0	0	0	0	0	0	0
0	0	0	0	0	0	0	0	0	0	0	0	0	0	0	0	0	0	0	0	0
0	0	0	0	0	0	0	0	0	0	0	0	0	0	0	0	0	0	0	0	0
0	0	0	0	0	0	0	85.71	0	0	0	0	0	0	0	0	0	0	0	0	0
0	0	0	0	0	0	0	0	0	0	0	0	0	0	0	0	0	0	0	0	0
0	0	0	0	0	0	0	0	0	0	0	0	0	0	0	0	0	0	0	0	0
0	0	0	0	0	0	0	0	0	0	0	0	0	0	0	0	0	0	0	0	0
0	0	0	0	0	0	0	0	0	0	0	0	0	0	0	0	0	0	0	0	0
0	0	0	0	0	0	0	0	0	0	0	0	0	0	0	0	0	0	0	0	0

0	0	0	0	0	0	0	0	0	0	0	0	0	0	0	0	0	0	0	0	0
0	0	0	0	0	0	0	0	0	0	0	0	0	0	0	0	0	0	0	0	0
0	0	0	0	0	0	0	0	0	0	0	0	0	0	0	0	0	0	0	0	0
0	0	0	0	0	0	0	0	0	0	0	0	0	0	0	0	0	0	0	0	0
0	0	0	0	0	0	0	0	0	0	0	0	0	0	0	0	0	0	0	0	0
0	0	0	0	0	0	0	0	0	0	0	0	0	0	0	0	0	0	0	0	0
0	0	0	0	0	0	0	0	0	0	0	0	0	0	0	0	0	0	0	0	0
0	0	0	0	77.78	0	0	0	0	0	0	0	0	0	0	0	0	0	0	0	0
0	0	0	0	0	0	0	0	0	0	0	0	0	0	0	0	0	0	0	0	0
0	0	0	0	0	0	0	0	100	0	0	0	0	0	0	0	0	0	0	0	0
0	0	0	0	0	0	0	0	0	0	0	0	0	0	0	0	0	0	0	0	0
0	0	0	0	0	0	0	0	0	0	0	0	0	0	0	0	0	0	0	0	0
0	0	0	0	0	0	0	0	0	0	0	0	0	0	0	0	0	0	0	0	0
0	0	0	0	0	0	0	0	0	0	0	0	0	0	0	0	0	0	0	0	0
0	0	0	0	0	0	0	0	0	0	0	0	0	0	0	0	0	0	0	0	0
0	0	0	0	0	0	0	0	0	0	0	0	0	0	0	0	0	0	0	0	0
0	0	0	0	0	0	0	0	0	0	0	0	0	0	0	0	0	0	0	0	0
0	0	0	0	0	0	0	0	0	0	0	0	0	0	0	0	0	0	0	0	0
0	0	0	0	0	0	0	0	0	0	0	0	0	0	0	0	0	0	0	0	0
0	0	0	0	0	0	0	0	0	0	0	0	0	0	0	0	0	0	0	0	0
0	0	0	0	0	0	0	0	0	0	0	0	0	0	0	0	0	0	0	0	0
0	0	0	0	0	0	0	0	0	0	0	0	0	0	0	0	0	0	0	0	0
0	0	0	0	0	0	0	0	0	0	0	0	0	0	0	0	0	0	0	0	0
0	0	0	0	0	0	0	0	0	0	0	0	0	0	0	0	0	0	0	0	0
0	0	0	0	0	0	0	0	0	0	0	0	0	0	0	0	0	0	0	0	0
0	0	0	0	0	0	0	0	100	0	0	0	0	0	0	0	0	0	0	0	0
0	0	0	0	0	0	0	0	0	0	0	0	0	0	0	0	0	0	0	0	0
0	0	0	0	0	0	0	0	0	0	0	0	0	0	0	0	0	0	0	0	0
0	0	0	0	77.78	0	0	0	0	0	0	0	0	0	0	0	0	0	0	0	0
0	0	0	0	0	0	0	0	0	0	0	0	0	0	0	0	0	0	0	0	0
0	0	0	0	0	0	0	0	0	0	0	0	0	0	0	0	0	0	0	0	0
0	0	0	0	0	0	0	0	0	100	0	100	0	0	0	0	0	0	0	0	0
0	0	0	0	0	0	0	0	0	0	0	0	0	0	0	0	0	0	0	0	0
0	0	0	0	77.78	0	0	0	0	0	0	0	0	0	0	0	0	0	0	0	0
0	0	0	0	0	0	0	0	0	0	0	0	0	0	0	0	0	0	0	0	0
0	0	0	0	0	0	0	0	0	0	0	0	0	0	0	0	0	0	0	0	0
0	0	0	0	0	0	0	0	0	0	0	0	0	0	0	0	0	0	0	0	0
0	0	0	0	0	0	0	0	0	0	0	0	0	0	0	0	0	0	0	0	0
0	0	0	0	0	0	0	0	0	0	0	0	0	0	0	0	0	0	0	0	0
0	0	0	0	0	0	0	0	100	0	0	0	0	0	0	0	0	0	0	0	0
0	0	0	0	0	0	0	0	0	0	0	0	0	0	0	0	0	0	0	0	0
0	0	0	0	0	0	0	0	0	0	0	0	0	0	0	0	0	0	0	0	0
0	0	0	0	0	100	0	0	0	0	0	0	0	0	0	0	0	0	0	0	0
0	0	0	0	77.78	0	0	0	0	0	0	0	0	0	0	0	0	0	0	0	0
0	0	0	0	0	0	0	0	0	0	0	0	0	0	0	0	0	0	0	0	0
0	0	0	0	0	0	0	0	0	0	0	0	0	0	0	0	0	0	0	0	0

TEM-57	TEM-60	TEM-83	TEM-89	TEM-91	TEM-93	MCR-1	FosA3	FosC2	ErmB	mphA	oleC	catB3	catI	catIII	cmlA1	cmlA5	cmlA6	QnrS5	QnrVC4	sul1
0	0	0	0	0	0	0	0	0	0	100	0	0	0	0	0	0	0	0	0	100
100	0	0	0	0	0	0	0	0	97.96	100	0	0	0	0	0	0	0	0	0	0
0	0	0	0	0	0	0	0	0	0	0	0	0	0	0	0	0	0	0	0	0
0	0	0	0	0	0	0	0	0	0	0	0	0	0	0	0	0	0	0	0	0
0	0	0	0	0	0	0	0	0	0	0	0	0	0	0	0	0	0	0	0	0
0	0	0	0	0	0	0	0	0	0	0	0	0	0	0	0	0	0	0	0	0
0	0	0	0	0	0	0	0	0	0	0	0	0	0	0	0	0	0	0	0	0
0	0	0	0	0	0	0	0	0	0	0	0	0	0	0	0	0	0	0	0	0
0	0	0	0	0	0	0	0	0	0	100	0	0	0	0	0	0	0	0	0	100
0	0	0	0	0	0	0	0	0	97.96	0	0	0	0	0	0	0	0	0	0	0
0	0	0	0	0	0	0	0	0	0	0	0	0	0	0	0	0	0	0	0	0
0	0	0	0	0	0	0	0	0	0	100	0	0	0	0	0	0	0	0	0	100
0	0	0	0	0	0	0	0	0	0	0	0	0	0	0	0	0	0	0	0	0
0	0	0	0	0	0	0	0	0	0	100	0	0	0	0	0	0	0	0	0	0
0	0	0	0	0	0	0	0	0	0	100	0	0	0	0	0	0	0	0	0	0
0	0	0	0	0	0	0	0	0	0	100	0	0	0	0	0	0	0	0	0	0
0	0	0	0	0	0	0	0	0	0	100	0	0	0	0	0	0	0	0	0	0
0	0	0	0	0	0	0	0	0	0	100	0	0	0	0	0	0	0	0	0	0
0	0	0	0	0	0	0	0	0	0	100	0	0	0	0	0	0	0	0	0	0
0	0	0	0	0	0	0	0	0	0	100	0	0	0	0	0	0	0	0	0	0
0	0	0	0	0	0	0	0	0	0	100	0	0	0	0	0	0	0	0	0	0
0	0	0	0	0	0	0	0	0	0	100	0	0	0	0	0	0	0	0	0	0
0	0	0	0	0	0	0	0	0	0	100	0	0	0	0	0	0	0	0	0	0
0	0	0	0	0	0	0	0	0	0	100	0	0	0	0	0	0	0	0	0	0
0	0	0	0	0	0	0	0	0	0	100	0	0	0	0	0	0	0	0	0	0
0	0	0	0	0	0	0	0	0	0	100	0	0	0	0	0	0	0	0	0	0
0	0	0	0	0	0	0	0	0	0	100	0	0	0	0	0	0	0	0	0	0
0	0	0	0	0	0	0	0	0	0	100	0	0	0	0	0	0	0	0	0	0
0	0	0	0	0	0	0	0	0	0	100	0	0	0	0	0	0	0	0	0	0
0	0	0	0	0	0	0	0	0	0	100	0	0	0	0	0	0	0	0	0	0
0	0	0	0	0	0	0	0	0	0	100	0	0	0	0	0	0	0	0	0	0
0	0	0	0	0	0	0	0	0	0	100	0	0	0	0	0	0	0	0	0	0
0	0	0	0	0	0	0	0	0	0	100	0	0	0	0	0	0	0	0	0	0
0	0	0	0	0	0	0	0	0	0	100	0	0	0	0	0	0	0	0	0	0
0	0	0	0	0	0	0	0	0	0	100	0	0	0	0	0	0	0	0	0	0
0	0	0	0	0	0	0	0	0	0	100	0	0	0	0	0	0	0	0	0	0
0	0	0	0	0	0	0	0	0	0	100	0	0	0	0	0	0	0	0	0	0
0	0	0	0	0	0	0	0	0	0	100	0	0	0	0	0	0	0	0	0	0
0	0	0	0	0	0	0	0	0	0	100	0	0	0	0	0	0	0	0	0	0
0	0	0	0	0	0	0	0	0	0	100	0	0	0	0	0	0	0	0	0	0
0	0	0	0	0	0	0	0	0	0	100	0	0	0	0	0	0	0	0	0	0
0	0	0	0	0	0	0	0	0	0	100	0	0	0	0	0	0	0	0	0	0
0	0	0	0	0	0	0	0	0	0	100	0	0	0	0	0	0	0	0	0	0
0	0	0	0	0	0	0	0	0	0	100	0	0	0	0	0	0	0	0	0	0
0	0	0	0	0	0	0	0	0	0	100	0	0	0	0	0	0	0	0	0	0
0	0	0	0	0	0	0	0	0	0	100	0	0	0	0	0	0	0	0	0	0
0	0	0	0	0	0	0	0	0	0	100	0	0	0	0	0	0	0	0	0	0
0	0	0	0	0	0	0	0	0	0	100	0	0	0	0	0	0	0	0	0	0
0	0	0	0	0	0	0	0	0	0	100	0	0	0	0	0	0	0	0	0	0
0	0	0	0	0	0	0	0	0	0	100	0	0	0	0	0	0	0	0	0	0
0	0	0	0	0	0	0	0	0	0	100	0	0	0	0	0	0	0	0	0	0

0	0	0	0	100	0	0	0	0	97.96	100	100	0	0	0	0	0	0	0	0	0
83.16	0	0	0	0	0	0	0	0	97.96	100	0	0	0	0	0	0	0	0	0	0
100	0	0	0	0	0	0	0	0	97.96	100	0	0	0	0	0	0	0	0	0	0
0	0	0	0	100	0	0	0	0	0	0	100	0	0	0	0	0	0	0	0	0
100	0	0	0	0	0	0	0	0	97.96	100	0	0	0	0	0	0	100	0	0	100
0	0	0	0	0	0	0	0	0	0	100	0	0	0	0	0	0	0	0	0	100
0	0	0	0	0	0	0	0	0	0	0	0	0	0	0	0	0	0	0	0	0
0	0	0	0	0	0	0	0	0	0	0	0	0	0	0	0	0	0	0	0	0
0	0	0	0	0	0	0	0	0	0	0	0	0	0	0	0	0	0	0	0	0
0	0	0	0	0	0	0	0	0	0	0	0	0	0	0	0	0	0	0	0	0
0	0	0	0	0	0	0	0	0	0	0	0	0	0	0	0	0	0	0	0	0
0	0	0	0	0	0	0	0	0	0	0	0	0	0	0	0	0	0	0	0	0
0	0	0	0	0	0	0	0	0	0	0	0	0	0	0	0	0	0	0	0	0
0	0	0	0	0	0	0	0	0	0	0	0	0	0	0	0	0	0	0	0	0
0	0	0	0	0	0	0	0	0	0	0	0	0	0	0	0	0	0	0	0	0
0	0	0	0	0	0	0	0	0	0	0	0	0	0	0	0	0	0	0	0	0
0	0	0	0	0	0	0	0	0	0	0	0	0	0	0	0	0	0	0	0	0
0	0	0	0	0	0	0	0	0	0	0	0	0	0	0	0	0	0	0	0	0
0	0	0	0	0	0	0	0	0	0	0	0	0	0	0	0	0	0	0	0	0
0	0	0	0	0	0	0	0	0	0	0	0	0	0	0	0	0	0	0	0	0
0	0	0	0	0	0	0	0	0	0	0	0	0	0	0	0	0	0	0	0	0
0	0	0	0	0	0	0	0	0	97.96	100	0	0	0	0	0	0	0	0	0	0
0	0	0	0	0	0	0	0	0	97.55	100	0	0	0	0	0	0	0	0	0	100
0	0	0	0	0	0	0	0	0	0	0	0	0	0	0	0	0	0	0	0	0
0	0	0	0	0	0	0	0	0	0	100	0	0	0	0	0	0	0	0	0	100
0	0	0	0	0	0	0	0	0	97.96	100	0	0	0	0	0	0	0	0	0	0
100	0	0	0	0	0	0	0	0	0	100	0	0	0	0	0	0	0	0	0	0
0	0	0	0	0	0	0	0	0	0	45	0	0	0	0	0	0	0	0	0	0
0	0	0	0	0	0	0	0	0	0	0	0	0	0	0	0	0	0	0	0	0
0	0	0	0	0	0	0	0	0	0	0	0	0	0	0	0	0	0	0	0	0
0	0	0	0	0	0	0	0	0	0	0	0	0	0	0	0	0	0	0	0	0
0	0	0	0	0	0	0	0	0	0	0	0	0	0	0	0	0	0	0	0	0
0	0	0	0	0	0	0	0	0	0	0	0	0	0	0	0	0	0	0	0	0
0	0	0	0	0	0	0	0	0	0	0	0	0	0	0	0	0	0	0	0	0
0	0	0	0	0	0	0	0	0	0	0	0	0	0	0	0	0	0	0	0	0
0	0	0	0	0	0	0	0	0	0	0	0	0	0	0	0	0	0	0	0	0
0	0	0	0	0	0	0	0	0	0	0	0	0	0	0	0	0	0	0	0	0
0	0	0	0	0	0	0	0	0	0	0	0	100	0	0	0	0	0	0	0	0
0	0	0	0	0	0	0	0	0	0	0	0	0	0	0	0	0	0	0	0	0
0	0	0	0	0	0	0	0	0	0	0	0	0	0	0	0	0	0	0	0	0
0	0	0	0	0	0	0	0	0	0	0	0	0	0	0	0	0	0	0	0	0
0	0	0	0	0	0	0	0	0	0	0	0	0	0	0	0	0	0	0	0	0
0	0	0	0	0	0	0	0	0	0	0	0	0	0	0	0	0	0	0	0	0
0	0	0	0	0	0	0	0	0	97.96	100	0	0	0	0	0	0	0	0	0	0
0	0	0	0	0	0	0	0	0	0	0	0	0	0	0	0	0	0	0	0	0
0	0	0	0	0	0	0	0	0	0	100	0	0	0	0	0	0	0	0	0	0
0	0	0	0	0	0	0	0	0	0	100	0	0	0	0	0	0	0	0	0	0
0	0	0	0	0	0	0	0	0	0	0	0	0	0	0	0	0	0	0	0	0
0	0	0	0	0	0	0	0	0	0	0	0	0	0	0	0	0	0	0	0	0
0	0	0	0	0	0	0	0	0	0	0	0	0	0	0	0	0	0	0	0	0
0	0	0	0	0	0	0	0	0	0	0	0	0	0	0	0	0	0	0	0	0
0	0	0	0	0	0	0	0	0	0	0	0	0	0	0	0	0	0	0	0	0
0	0	0	0	0	0	0	0	0	0	0	0	0	0	0	0	0	0	0	0	0
0	0	0	0	0	0	0	0	0	97.96	100	0	0	0	0	0	0	0	0	0	0

0	0	0	0	0	0	0	0	0	0	0	0	0	0	0	0	0	0	0	0	0
0	0	0	0	0	0	0	0	0	0	0	0	0	0	0	0	0	0	0	0	0
0	0	0	0	0	0	0	0	0	0	0	0	0	0	0	0	0	0	0	0	0
0	0	0	0	0	0	0	0	0	0	0	0	0	0	0	0	0	0	0	0	0
0	0	0	0	0	0	0	0	0	0	0	0	0	0	0	0	0	0	0	0	0
0	0	0	0	0	0	0	0	0	0	0	0	0	0	0	0	0	0	0	0	0
0	0	0	0	0	0	0	0	0	0	0	0	0	0	0	0	0	0	0	0	0
0	0	0	0	0	0	0	0	0	0	100	0	0	0	0	0	0	0	0	0	0
0	0	0	0	0	0	0	0	0	0	0	0	0	0	0	0	0	0	0	0	0
0	0	0	0	0	0	0	0	0	0	0	0	0	0	0	0	0	0	0	0	0
0	0	0	0	0	0	0	0	0	0	0	0	0	0	0	0	0	0	0	0	0
0	0	0	0	0	0	0	0	0	0	0	0	0	0	0	0	0	0	0	0	0
0	0	0	0	0	0	0	0	0	0	100	0	0	0	0	0	0	0	0	0	100
0	0	0	0	0	0	0	0	0	0	0	0	0	0	0	0	0	0	0	0	0
0	0	0	0	0	0	0	0	0	0	0	0	0	0	0	0	0	0	0	0	0
0	0	0	0	0	0	0	0	0	0	0	0	0	0	0	0	0	0	0	0	0
0	0	0	0	0	0	0	0	0	0	0	0	0	0	0	0	0	0	0	0	0
0	0	0	0	0	0	0	0	0	0	0	0	0	0	0	0	0	0	0	0	0
0	0	0	0	0	0	0	0	0	0	0	0	0	0	0	0	0	0	0	0	0
0	0	0	0	0	0	0	0	0	0	0	0	0	0	0	0	0	0	0	0	0
0	0	0	0	0	0	0	0	0	0	0	0	0	0	0	0	0	0	0	0	0
0	0	0	0	0	0	0	0	0	0	0	0	0	0	0	0	0	0	0	0	0
0	0	0	0	0	0	0	0	0	0	0	0	0	0	0	0	0	0	0	0	0
0	0	0	0	0	0	0	0	0	0	0	0	0	0	0	0	0	0	0	0	0
0	0	0	0	100	0	0	0	0	97.96	100	0	0	0	0	0	0	0	0	0	0
0	0	0	0	0	0	0	0	0	0	0	0	0	0	0	0	0	0	0	0	0
0	0	0	0	0	0	0	0	0	0	0	0	0	0	0	0	0	0	0	0	0
0	0	0	0	0	0	0	0	0	0	0	0	0	0	0	0	0	0	0	0	0
0	0	0	0	0	0	0	0	0	0	0	0	0	0	0	0	0	0	0	0	0
0	0	0	0	0	0	0	0	0	0	0	0	0	0	0	0	0	0	0	0	0
0	0	0	0	0	0	0	0	0	0	0	0	0	0	0	0	0	0	0	0	0
0	0	0	0	0	0	0	0	0	0	0	0	0	0	0	0	0	0	0	0	0
0	0	0	0	0	0	0	0	0	0	0	0	0	0	0	0	0	0	0	0	0
0	0	0	0	0	0	0	0	0	0	0	0	0	0	0	0	0	0	0	0	0
0	0	0	0	0	0	0	0	0	0	0	0	0	0	0	0	0	0	0	0	0
0	0	0	0	0	0	0	0	0	0	0	0	0	0	0	0	0	0	0	0	0
0	0	0	0	0	0	0	0	0	0	0	0	0	0	0	0	0	0	0	0	0
0	0	0	0	0	0	0	0	0	0	0	0	0	0	0	0	0	0	0	0	0
0	0	0	0	0	0	0	0	0	0	0	0	0	0	0	0	0	0	0	0	0
0	0	0	0	0	0	0	0	0	0	0	0	0	0	0	0	0	0	0	0	0
0	0	0	0	0	0	0	0	0	0	0	0	0	0	0	0	0	0	0	0	0
0	0	0	0	0	0	0	0	0	0	0	0	0	0	0	0	0	0	0	0	0
0	0	0	0	0	0	0	0	0	0	0	0	0	0	0	0	0	0	0	0	0
0	0	0	0	0	0	0	0	0	97.96	0	0	0	0	0	0	0	0	0	0	0
0	0	0	0	0	0	0	0	0	0	0	0	0	0	0	0	0	0	0	0	0
0	0	0	0	0	0	0	0	0	0	0	0	0	0	0	0	0	0	0	0	0
0	0	0	0	0	0	0	0	0	0	0	0	0	0	0	0	0	0	0	0	0
0	0	0	0	0	0	0	0	0	0	0	0	0	0	0	0	0	0	0	0	0
0	0	0	0	0	0	0	0	0	0	0	0	0	0	0	0	0	0	0	0	0
0	0	0	0	0	0	0	0	0	0	0	0	0	0	0	0	0	0	0	0	0
0	0	0	0	0	0	0	0	0	0	0	0	0	0	0	0	0	0	0	0	0
0	0	0	0	0	0	0	0	0	0	0	0	0	0	0	0	0	0	0	0	0
0	0	0	0	0	0	0	0	0	0	0	0	0	0	0	0	0	0	0	0	0

0	0	0	0	0	0	0	0	0	0	0	0	0	0	0	0	0	0	0	0	0
0	0	0	0	0	0	0	0	0	0	0	0	0	0	0	0	0	0	0	0	0
0	0	0	0	0	0	0	0	0	0	0	0	0	0	0	0	0	0	0	0	0
0	0	0	0	0	0	0	0	0	0	0	0	0	0	0	0	0	0	0	0	0
0	0	0	0	0	0	0	0	0	0	0	0	0	0	0	0	0	0	0	0	0
0	0	0	0	0	0	0	0	0	0	0	0	0	0	0	0	0	0	0	0	0
0	0	0	0	0	0	0	0	0	97.96	100	0	0	0	0	0	0	0	0	0	0
0	0	0	0	0	0	0	0	0	0	0	0	0	0	0	0	0	0	0	0	0
0	0	0	0	0	0	0	0	0	0	0	0	0	0	0	0	0	0	0	0	0
0	0	0	0	0	0	0	0	0	0	0	0	0	0	0	0	0	0	0	0	0
0	0	0	0	0	0	0	0	0	0	0	0	0	0	0	0	0	0	0	0	0
0	0	0	0	0	0	0	0	0	0	0	0	0	0	0	0	0	0	0	0	0
0	0	0	0	0	0	0	0	0	0	0	0	0	0	0	0	0	0	0	0	0
0	0	0	0	0	0	0	0	0	0	0	0	0	0	0	0	0	0	0	0	0
0	0	0	0	0	0	0	0	0	0	0	0	0	0	0	0	0	0	0	0	0
0	0	0	0	0	0	0	0	0	0	0	0	0	0	0	0	0	0	0	0	0
0	0	0	0	0	0	0	0	0	0	0	0	0	0	0	0	0	0	0	0	0
0	0	0	0	0	0	0	0	0	0	0	0	0	0	0	0	0	0	0	0	0
0	0	0	0	0	0	0	0	0	0	0	0	0	0	0	0	0	0	0	0	0
0	0	0	0	0	0	0	0	0	0	0	0	0	0	0	0	0	0	0	0	0
0	0	0	0	0	0	0	0	0	0	0	0	0	0	0	0	0	0	0	0	0
0	0	0	0	0	0	0	0	0	0	0	0	0	0	0	0	0	0	0	0	0
0	0	0	0	0	0	0	0	0	0	0	0	0	0	0	0	0	0	0	0	0
0	0	0	0	0	0	0	0	0	0	0	0	0	0	0	0	0	0	0	0	0
0	0	0	0	0	0	0	0	0	0	0	0	0	0	0	0	0	0	0	0	0
0	0	0	0	0	0	0	0	0	0	0	0	0	0	0	0	0	0	0	0	0
0	0	0	0	0	0	0	0	0	0	0	0	0	0	0	0	0	0	0	0	0
0	0	0	0	0	0	0	0	0	0	0	0	0	0	0	0	0	0	0	0	0
0	0	0	0	0	0	0	0	0	0	0	0	0	0	0	0	0	0	0	0	0
0	0	0	0	0	0	0	0	0	0	100	0	0	0	0	0	0	0	0	0	100
0	0	0	0	0	0	0	0	0	0	100	0	0	0	0	0	0	0	0	0	100
0	0	0	0	0	0	0	0	0	0	0	0	0	0	0	0	0	0	0	0	0
0	0	0	0	0	0	0	0	0	0	0	0	0	0	0	0	0	0	0	0	0
0	0	0	0	0	0	0	0	0	0	0	0	0	0	0	0	0	0	0	0	0
0	0	0	0	0	0	0	0	0	0	0	0	0	0	0	0	0	0	0	0	0
0	0	0	0	0	0	0	0	0	0	0	0	0	0	0	0	0	0	0	0	0
0	0	0	0	0	0	0	0	0	0	0	0	0	0	0	0	0	0	0	0	0
0	0	0	0	0	0	0	0	0	0	0	0	0	0	0	0	0	0	0	0	0
0	0	0	0	0	0	0	0	0	0	100	0	0	0	0	0	0	0	0	0	0
0	0	0	0	0	0	0	0	0	0	0	0	0	0	0	0	0	0	0	0	0
95.09	0	0	0	0	0	0	0	0	0	0	0	0	0	0	0	0	0	0	0	0
0	0	0	0	0	0	0	0	0	0	0	0	0	0	0	0	0	0	0	0	0
0	0	0	0	0	0	0	0	0	0	0	0	0	0	0	0	0	0	0	0	0
0	0	0	0	0	0	0	0	0	0	0	0	0	0	0	0	0	0	0	0	0
0	0	0	0	0	0	0	0	0	0	0	0	0	0	0	0	0	0	0	0	0
0	0	0	0	0	0	0	0	0	0	0	0	0	0	0	0	0	0	0	0	0
0	0	0	0	0	0	0	0	0	0	0	0	0	0	0	0	0	0	0	0	0
0	0	0	0	0	0	0	0	0	0	0	0	0	0	0	0	0	0	0	0	0
0	0	0	0	0	0	0	0	0	0	0	0	0	0	0	0	0	0	0	0	0
0	0	0	0	0	0	0	0	0	0	0	0	0	0	0	0	0	0	0	0	0
0	0	0	0	0	0	0	0	0	0	0	0	0	0	0	0	0	0	0	0	0
0	0	0	0	0	0	0	0	0	0	0	0	0	0	0	0	0	0	0	0	0
0	0	0	0	0	0	0	0	0	0	0	0	0	0	0	0	0	0	0	0	0
0	0	0	0	0	0	0	0	0	0	0	0	0	0	0	0	0	0	0	0	0

0	0	0	0	0	0	0	0	0	0	0	0	0	0	0	0	0	0	0	0	0
0	0	0	0	0	0	0	0	0	0	0	0	0	0	0	0	0	0	0	0	0
0	0	0	0	0	0	0	0	0	0	0	0	0	0	0	0	0	0	0	0	0
0	0	0	0	0	0	0	0	0	0	100	0	0	0	0	100	0	0	0	0	100
0	0	0	0	0	0	0	0	0	0	0	0	0	0	0	0	0	0	0	0	0
0	0	0	0	0	0	0	0	0	0	100	0	0	0	0	0	0	0	0	0	100
0	0	0	0	0	0	0	0	0	0	0	0	0	0	0	0	0	0	0	0	0
0	0	0	0	0	0	0	0	0	0	0	0	0	0	0	0	0	0	0	0	0
0	0	0	0	0	0	0	0	0	0	0	0	0	0	0	0	0	0	0	0	0
0	0	0	0	0	0	0	0	0	0	0	0	0	0	0	0	0	0	0	0	0
0	0	0	0	0	0	0	0	0	0	0	0	0	0	0	0	0	0	0	0	0
0	0	0	0	0	0	0	0	0	0	0	0	0	0	0	0	0	0	0	0	0
0	0	0	0	0	0	0	0	0	0	0	0	0	0	0	0	0	0	0	0	0
0	0	0	0	0	0	0	0	0	0	0	0	0	0	0	0	0	0	0	0	0
0	0	0	0	0	0	0	0	0	0	0	0	0	0	0	0	0	0	0	0	0
0	0	0	0	0	0	0	0	0	0	0	0	0	0	0	0	0	0	0	0	0
0	0	0	0	0	0	0	0	0	0	0	0	0	0	0	0	0	0	0	0	0
0	0	0	0	0	0	0	0	0	0	0	0	0	0	0	0	0	0	0	0	0
0	0	0	0	0	0	0	0	0	0	0	0	0	0	0	0	0	0	0	0	0
0	0	0	0	0	0	0	0	0	0	0	0	0	0	0	0	0	0	0	0	0
0	0	0	0	0	0	0	0	0	0	0	0	0	0	0	0	0	0	0	0	0
0	0	0	0	0	0	0	0	0	0	0	0	0	0	0	0	0	0	0	0	0
0	0	0	0	0	0	0	0	0	0	0	0	0	0	0	0	0	0	0	0	0
0	0	0	0	0	0	0	0	0	0	100	0	0	0	0	0	0	0	0	0	100
0	0	0	0	0	0	0	0	0	0	0	0	0	0	0	0	0	0	0	0	0
0	0	0	0	0	0	0	0	0	0	0	0	0	0	0	0	0	0	0	0	0
0	0	0	0	0	0	0	0	0	97.96	100	0	0	0	0	0	0	0	0	0	0
0	0	0	0	0	0	0	0	0	0	0	0	0	0	0	0	0	0	0	0	0
0	0	0	0	0	0	0	0	0	0	0	0	0	0	0	0	0	0	0	0	0
0	0	0	0	0	0	0	0	0	0	0	0	0	0	0	0	0	0	0	0	0
0	0	0	0	0	0	0	0	0	97.96	0	0	0	0	0	0	0	0	0	0	0
0	0	0	0	0	0	0	0	0	97.96	100	0	0	0	0	0	0	0	0	0	0
0	0	0	0	0	0	0	0	0	0	0	0	0	0	0	0	0	0	0	0	0
0	0	0	0	0	0	0	0	0	0	0	100	0	0	0	0	0	0	0	0	0
0	0	0	0	0	0	0	0	0	0	0	0	0	0	0	0	0	0	0	0	0
0	0	0	0	0	0	100	0	0	0	100	0	0	0	0	0	0	0	0	0	100
0	0	0	0	0	0	0	0	0	0	0	0	0	0	0	0	0	0	0	0	0
0	0	0	0	0	0	0	0	0	97.96	100	0	0	0	0	0	0	0	0	0	0
0	0	0	0	0	0	0	100	0	97.96	100	0	0	0	0	0	0	0	0	0	100
0	0	0	0	0	0	0	0	0	0	0	0	0	0	0	0	0	0	0	0	0
0	0	0	0	0	0	0	0	0	97.96	100	0	0	0	0	0	0	0	0	0	0
100	0	0	0	0	0	0	0	0	97.96	100	0	0	0	0	0	0	0	0	0	0
0	0	0	0	0	0	0	0	0	0	0	0	0	0	0	0	0	0	0	0	0
95.09	0	0	0	0	100	0	0	0	97.96	100	0	0	0	0	0	0	0	0	0	0
0	0	0	0	0	0	0	0	0	0	0	0	0	0	0	0	0	0	0	0	0
0	0	0	0	0	0	0	0	0	97.96	100	0	0	0	0	0	0	0	0	0	0
0	0	0	0	0	0	0	0	0	0	0	0	0	0	0	0	0	0	0	0	0
0	0	0	0	0	0	0	0	0	0	0	0	0	0	0	0	0	0	0	0	0
0	0	0	0	0	0	0	0	0	0	0	0	0	0	0	0	0	0	0	0	0
0	0	0	0	0	0	0	0	0	0	100	0	0	0	0	0	0	0	0	0	0
0	0	0	0	0	0	0	0	0	0	0	0	0	0	0	0	0	0	0	0	0
100	0	0	0	0	0	0	0	0	97.96	100	0	0	0	0	0	0	0	0	0	100
0	0	0	0	0	0	0	0	0	0	0	0	0	0	0	0	0	0	0	0	0
0	0	0	0	0	0	0	0	0	0	0	0	0	0	0	0	0	0	0	0	0

0	0	0	0	0	0	0	0	0	97.96	100	0	0	0	0	0	0	0	0	0	100
0	0	0	0	0	0	0	0	0	0	0	0	0	0	0	0	0	0	0	0	0
0	0	0	0	0	0	0	0	0	0	0	0	0	0	0	0	0	0	0	0	0
0	0	0	0	0	0	0	0	0	0	0	100	0	0	0	0	0	0	0	0	0
0	0	0	0	0	0	0	0	0	0	0	0	0	0	0	0	0	0	0	0	0
0	0	0	0	0	0	0	0	0	0	0	0	0	0	0	0	0	0	0	0	0
0	0	0	0	0	0	0	0	0	0	0	0	0	0	0	0	0	0	0	0	0
0	0	0	0	0	0	0	0	0	0	0	0	0	0	0	0	0	0	0	0	0
0	0	0	0	0	0	0	0	0	0	0	0	0	0	0	0	0	0	0	0	0
0	0	0	0	0	0	0	0	0	0	0	0	0	0	0	0	0	0	0	0	0
0	0	0	0	0	0	0	0	0	0	0	0	0	0	0	0	0	0	0	0	0
0	0	0	0	0	0	0	0	0	0	0	0	0	0	0	0	0	0	0	0	0
0	0	0	0	0	0	0	0	0	0	0	0	0	0	0	0	0	0	0	0	0
0	0	0	0	0	0	0	0	0	97.96	0	0	0	0	0	0	0	0	0	0	0
0	0	0	0	0	0	0	0	0	0	0	0	0	0	0	0	0	0	0	0	0
0	0	0	0	0	0	0	0	0	0	100	0	0	0	0	0	0	0	0	0	100
0	0	0	0	0	0	0	0	0	0	0	0	0	0	0	0	0	0	0	0	0
0	0	0	0	0	0	0	0	0	0	0	0	0	0	0	0	0	0	0	0	0
0	0	0	0	0	0	0	0	0	0	0	0	0	0	0	0	0	0	0	0	0
0	0	0	0	0	0	0	0	0	0	100	0	0	0	0	0	0	0	0	0	100
0	0	0	0	0	0	0	0	0	0	0	0	0	0	0	0	0	0	0	0	0
0	0	0	0	0	0	0	0	0	0	0	0	0	0	0	0	0	0	0	0	0
0	0	0	0	0	0	0	0	0	0	0	0	0	0	0	0	0	0	0	0	0
0	0	0	0	0	0	0	0	0	0	0	0	0	0	0	0	0	0	0	0	0
0	0	0	0	0	0	0	0	0	0	0	0	0	0	0	0	0	0	0	0	0
0	0	0	0	0	0	0	0	0	0	100	0	0	0	0	0	0	0	0	0	100
0	0	0	0	0	0	0	0	0	0	0	0	0	0	0	0	0	0	0	0	0
0	0	0	0	0	0	0	0	0	0	0	0	0	0	0	0	0	0	0	0	0
100	0	0	0	0	0	0	0	0	0	0	0	0	0	0	0	0	0	0	0	0
0	0	0	0	0	0	0	0	0	0	100	0	0	0	0	0	0	0	0	0	100
0	0	0	0	0	0	0	0	0	0	0	0	0	0	0	0	0	0	0	0	0
0	0	0	0	0	0	0	0	0	0	0	0	0	0	0	0	0	0	0	0	0
0	0	0	0	0	0	0	0	0	0	0	0	0	0	0	0	0	0	0	0	0
100	0	0	0	0	0	0	0	0	0	0	0	0	0	0	0	0	0	0	0	0
0	0	0	0	0	0	100	0	0	0	0	0	0	0	0	0	0	0	0	0	0
0	0	0	0	0	0	0	0	0	0	0	0	0	0	0	0	0	0	0	0	0
0	0	0	0	0	0	0	0	0	0	0	0	0	0	0	0	0	0	0	0	0
0	0	0	0	0	0	0	0	0	0	0	0	0	0	0	0	0	0	0	0	0
0	0	0	0	0	0	0	0	0	0	0	0	0	0	0	0	0	0	0	0	0
0	0	0	0	0	0	0	0	0	0	0	0	0	0	0	0	0	0	0	0	0
100	0	0	0	0	0	0	0	0	97.96	100	0	0	0	0	0	0	0	0	0	0
0	0	0	0	0	0	0	0	0	0	0	0	0	0	0	0	0	0	0	0	0
0	0	0	0	0	0	0	0	0	0	0	0	0	0	0	0	0	0	0	0	0
0	0	0	0	0	0	0	0	0	0	100	0	0	0	0	0	0	0	0	0	100
0	0	0	0	0	0	0	0	0	0	0	0	0	0	0	0	0	0	0	0	0
0	0	0	0	0	0	0	0	0	0	0	0	0	0	0	0	0	0	0	0	0
0	0	0	0	0	0	0	0	0	0	0	0	0	0	0	0	0	0	0	0	0
0	0	0	0	0	0	0	0	0	97.96	0	0	0	0	0	0	0	0	0	0	0
0	0	0	0	0	0	0	0	0	97.96	0	0	0	0	0	0	0	0	0	0	0
0	0	0	0	0	0	0	100	0	0	100	0	0	0	0	0	0	0	0	0	0
0	0	0	0	0	0	0	0	0	0	0	0	0	0	0	0	0	0	0	0	0
0	0	0	0	0	0	0	0	0	0	0	0	0	0	0	0	0	0	0	0	0
0	0	0	0	0	0	0	0	0	97.96	0	0	0	0	0	0	0	0	0	0	0
0	0	0	0	0	0	0	0	0	97.96	0	0	0	0	0	0	0	0	0	0	0

0	0	0	0	0	0	0	0	0	0	100	0	0	0	0	0	0	0	0	0	100
0	0	0	0	0	0	0	0	0	97.96	0	0	0	0	0	0	0	0	0	0	0
0	0	0	0	0	0	0	0	0	0	100	0	0	0	0	0	0	0	0	0	100
0	0	0	0	0	0	0	0	0	0	100	0	0	0	0	0	0	0	0	0	100
0	0	0	0	0	0	0	0	0	0	100	0	0	0	0	0	0	0	0	0	100
0	0	0	0	0	0	0	0	0	0	41.67	0	0	0	0	0	0	0	0	0	100
0	0	0	0	0	0	0	0	0	0	0	0	0	0	0	0	0	0	0	0	0
0	0	0	0	0	0	0	0	0	0	100	0	0	0	0	0	0	0	0	0	100
0	0	0	0	0	0	0	0	0	0	100	0	0	0	0	0	0	0	0	0	100
0	0	0	0	0	0	0	0	0	0	100	0	0	0	0	0	0	0	0	0	100
0	0	0	0	0	0	0	0	0	0	100	0	0	0	0	0	0	0	0	0	100
100	0	0	0	0	0	0	0	0	0	0	0	0	0	0	0	0	0	0	0	0
0	0	0	0	0	0	0	0	0	0	0	0	0	0	0	0	0	0	0	0	0
0	0	0	0	0	0	100	0	0	0	0	0	0	0	0	0	0	0	0	0	0
0	0	0	0	0	0	0	0	0	0	0	0	0	0	0	0	0	0	0	0	0
0	0	0	0	0	0	0	0	0	97.96	0	0	0	0	0	0	0	0	0	0	0
0	0	0	0	0	0	0	0	0	0	0	0	0	0	0	0	0	0	0	0	0
0	0	0	0	0	0	100	0	0	0	0	0	0	0	0	0	0	0	0	0	0
0	0	0	0	0	0	0	0	0	0	0	0	0	0	0	0	0	0	0	0	0
0	0	0	0	0	0	0	0	0	0	0	0	0	0	0	0	0	0	0	0	0
0	0	0	0	0	0	0	0	0	0	0	0	0	0	0	0	0	0	0	0	0
0	0	0	0	0	0	0	0	0	0	100	0	0	0	0	0	0	0	0	0	100
0	0	0	0	0	0	0	0	0	0	0	0	0	0	0	0	0	0	0	0	0
0	0	0	0	0	0	0	0	0	97.96	100	0	0	0	0	0	0	0	0	0	100
0	100	0	0	0	0	0	0	0	97.96	100	0	0	0	0	0	0	0	0	0	0
0	0	0	0	0	0	0	0	0	0	0	0	0	0	0	0	0	0	0	0	0
0	0	0	0	0	0	0	0	0	0	100	0	0	0	0	0	0	0	0	0	100
0	0	0	0	0	0	0	0	0	0	100	0	0	0	0	0	0	0	0	0	100
0	0	0	0	0	0	0	0	0	0	0	0	0	0	0	0	0	0	0	0	0
0	0	0	0	0	0	0	0	0	0	0	0	0	0	0	0	0	0	0	0	0
0	0	0	0	0	0	0	0	0	0	0	0	0	0	0	0	0	0	0	0	0
0	0	0	0	0	0	100	0	0	0	0	0	0	0	0	0	0	0	0	0	0
0	0	0	0	0	0	0	0	0	0	0	0	0	0	0	0	0	0	0	0	0
0	0	0	0	0	0	0	0	0	0	0	0	0	0	0	0	0	0	0	0	0
0	0	0	0	0	0	0	0	0	0	0	0	0	0	0	0	0	0	0	0	0
0	0	0	0	0	0	0	0	0	0	0	0	0	0	0	0	0	0	0	0	0
0	0	0	0	0	0	0	0	0	0	0	0	0	0	0	0	0	0	0	0	0
0	0	0	0	0	0	0	0	0	0	0	0	0	0	0	0	0	0	0	0	0
0	0	0	0	0	0	0	0	0	0	0	0	0	0	0	0	0	0	0	0	0
0	0	0	0	0	0	0	0	0	0	0	0	0	0	0	0	0	0	0	0	0
0	0	0	0	0	0	0	0	0	0	0	0	0	0	0	0	0	0	0	0	0
0	0	0	0	0	0	0	0	0	0	0	0	0	0	0	0	0	0	0	0	0
0	0	0	0	0	0	0	0	0	0	0	0	0	0	0	0	0	0	0	0	0
0	0	0	0	0	0	0	0	0	0	0	0	0	0	0	0	0	0	0	0	0
0	0	0	0	0	0	0	0	0	0	0	0	0	0	0	0	0	0	0	0	0
100	0	0	0	0	0	0	0	0	97.96	100	0	0	0	0	0	0	0	0	0	0
0	0	0	0	0	0	0	0	0	0	0	0	0	0	0	0	0	0	0	0	0
0	0	0	0	0	0	0	0	0	0	100	0	0	0	0	0	100	0	0	100	0
0	0	0	0	0	0	0	0	0	0	0	0	0	0	0	0	0	0	0	0	0
0	0	0	0	0	0	0	0	0	0	0	0	0	0	0	0	0	0	0	0	0
0	0	0	0	0	0	0	0	0	0	0	0	0	0	0	0	0	0	0	0	0
0	0	0	0	0	0	0	0	0	0	0	0	0	0	0	0	0	0	0	0	0
0	0	0	0	0	0	0	0	0	0	100	0	0	0	0	0	0	0	0	0	100
0	0	0	0	0	0	0	0	0	0	0	0	0	0	0	0	0	0	0	0	0

0	0	0	0	0	0	0	0	0	0	0	0	0	0	0	0	0	0	0	0	0
0	0	0	0	0	0	0	0	0	0	0	0	0	0	0	0	0	0	0	0	0
0	0	0	0	0	0	0	0	0	0	0	0	0	0	0	0	0	0	0	0	0
0	0	0	0	0	0	0	0	0	0	0	0	0	0	0	0	0	0	0	0	0
0	0	0	0	0	0	0	0	0	0	0	0	0	0	0	0	0	0	0	0	0
0	0	0	0	0	0	0	0	0	97.96	100	0	0	0	0	0	0	0	0	0	100
0	0	0	0	0	0	0	0	0	0	0	0	0	0	0	0	0	0	0	0	0
100	0	0	0	0	0	0	0	0	0	0	0	0	0	0	0	0	0	0	0	0
0	0	0	0	0	0	100	0	0	0	0	0	0	0	0	0	0	0	0	0	0
0	0	0	0	0	0	0	0	0	0	100	0	0	0	0	0	0	0	0	0	100
0	0	0	0	0	0	0	0	0	0	100	0	0	0	0	0	0	0	0	0	100
100	0	0	0	0	0	0	0	0	97.96	100	0	0	0	0	0	0	0	0	0	0
0	0	0	0	0	0	0	0	0	0	100	0	0	0	0	0	0	0	0	0	0
0	0	0	0	0	0	0	0	0	0	0	0	0	0	0	0	0	0	0	0	0
0	0	0	0	0	0	0	0	0	0	0	0	0	0	0	0	0	0	0	0	0
0	0	0	0	0	0	0	0	0	0	100	0	0	0	0	0	0	0	0	0	100
0	0	0	0	0	0	0	0	0	0	0	0	0	0	0	0	0	0	0	0	0
0	0	0	0	0	0	0	0	0	0	0	0	0	0	0	0	0	0	0	0	0
0	0	0	0	0	0	0	0	0	0	0	0	0	0	0	0	0	0	0	0	0
0	0	0	0	0	0	0	0	0	0	0	0	0	0	0	0	0	0	0	0	0
0	0	0	0	0	0	0	0	0	0	0	0	0	0	0	0	0	0	0	0	0
0	0	0	0	0	0	0	0	0	0	0	0	0	0	0	0	0	0	0	0	0
0	0	0	0	0	0	0	0	0	0	100	0	0	0	0	0	0	0	0	0	100
0	0	0	0	0	0	0	0	0	0	100	0	0	0	0	0	0	0	0	0	100
0	0	0	0	0	0	0	0	0	0	0	0	0	0	0	0	0	0	0	0	0
0	0	0	0	0	0	0	0	0	0	100	0	0	0	0	0	0	0	0	0	100
0	0	0	0	0	0	0	0	0	0	100	0	0	0	0	0	0	0	0	0	0
0	0	0	0	0	0	0	0	0	0	100	0	0	0	0	0	0	0	0	0	0
0	0	0	0	0	0	0	0	0	0	100	0	0	0	0	0	0	0	0	0	0
0	0	0	0	0	0	0	0	0	0	100	0	0	0	0	0	0	0	0	0	0
0	0	0	0	0	0	0	0	0	0	100	0	0	0	0	0	0	0	0	0	0
0	0	0	0	0	0	0	0	0	0	100	0	0	0	0	0	0	0	0	0	0
0	0	0	0	0	0	0	0	0	0	100	0	0	0	0	0	0	0	0	0	0
0	0	0	0	0	0	0	0	0	0	100	0	0	0	0	0	0	0	0	0	0
0	0	0	0	0	0	0	0	0	0	100	0	0	0	0	0	0	0	0	0	0
0	0	0	0	0	0	0	0	0	0	100	0	0	0	0	0	0	0	0	0	0
0	0	0	0	0	0	0	0	0	0	100	0	0	0	0	0	0	0	0	0	0
0	0	0	0	0	0	0	0	0	0	100	0	0	0	0	0	0	0	0	0	0
0	0	0	0	0	0	0	0	0	0	100	0	0	0	0	0	0	0	0	0	0
0	0	0	0	0	0	0	0	0	0	100	0	0	0	0	0	0	0	0	0	100
0	0	0	0	0	0	0	0	0	0	100	0	0	0	0	0	0	0	0	0	100
0	0	0	0	0	0	0	0	0	0	100	0	0	0	0	0	0	0	0	0	100
0	0	0	0	0	0	0	0	0	0	100	0	0	0	0	0	0	0	0	0	100
0	0	0	0	0	0	0	0	0	0	100	0	0	0	0	0	0	0	0	0	100
0	0	0	0	0	0	0	0	0	0	100	0	0	0	0	0	0	0	0	0	100
0	0	0	0	0	0	0	0	0	0	100	0	0	0	0	0	0	0	0	0	100
0	0	0	0	0	0	0	0	0	0	100	0	0	0	0	0	0	0	0	0	100
0	0	0	0	0	0	0	0	0	0	100	0	0	0	0	0	0	0	0	0	0
0	0	0	0	0	0	0	0	0	0	100	0	0	0	0	0	0	0	0	0	0
0	0	0	0	0	0	100	0	0	0	100	0	0	0	0	0	0	0	0	0	100
0	0	0	0	0	0	0	0	0	0	100	0	0	0	0	0	0	0	0	0	100
0	0	0	0	0	0	0	0	0	0	100	0	0	0	0	0	0	0	0	0	100
0	0	0	0	0	0	100	0	0	0	100	0	0	0	0	0	0	0	0	0	100
0	0	0	0	0	0	100	0	0	0	100	0	0	0	0	0	0	0	0	0	100

0	0	0	0	0	0	100	0	0	0	100	0	0	0	0	0	0	0	0	0	100
0	0	0	0	0	0	0	0	0	0	100	0	0	0	0	0	0	0	0	0	100
0	0	0	0	0	0	0	0	0	0	100	0	0	0	0	0	0	0	0	0	100
0	0	0	0	0	0	0	0	0	0	100	0	0	0	0	0	0	0	0	0	100
0	0	0	0	0	0	100	0	0	0	100	0	0	0	0	0	0	0	0	0	100
0	0	0	0	0	0	100	0	0	0	100	0	0	0	0	0	0	0	0	0	100
0	0	0	0	0	0	100	0	0	0	100	0	0	0	0	0	0	0	0	0	100
0	0	0	0	0	0	100	0	0	0	100	0	0	0	0	0	0	0	0	0	100
0	0	0	0	0	0	100	0	0	0	100	0	0	0	0	0	0	0	0	0	100
0	0	0	0	0	0	100	0	0	0	100	0	0	0	0	0	0	0	0	0	100
0	0	0	0	0	0	100	0	0	0	100	0	0	0	0	0	0	0	0	0	100
0	0	0	0	0	0	0	0	0	0	100	0	0	0	0	0	0	0	0	0	100
0	0	0	0	0	0	100	0	0	0	100	0	0	0	0	0	0	0	0	0	100
0	0	0	0	0	0	100	0	0	0	100	0	0	0	0	0	0	0	0	0	100
0	0	0	0	0	0	100	0	0	0	100	0	0	0	0	0	100	0	98.62	0	100
0	0	0	0	0	0	100	0	0	0	100	0	0	0	0	0	0	0	0	0	100
0	0	0	0	0	0	100	0	0	0	100	0	0	0	0	0	0	0	0	0	100
0	0	0	0	0	0	100	0	0	0	100	0	0	0	0	0	0	0	0	0	100
0	0	0	0	0	0	100	0	0	0	100	0	0	0	0	0	0	0	0	0	100
0	0	0	0	0	0	100	0	0	0	100	0	0	0	0	0	0	0	0	0	100
0	0	0	0	0	0	100	0	0	0	100	0	0	0	0	0	0	0	0	0	100
0	0	0	0	0	0	100	0	0	0	100	0	0	0	0	0	0	0	0	0	100
0	0	0	0	0	0	100	0	0	0	100	0	0	0	0	0	0	0	0	0	100
0	0	0	0	0	0	100	0	0	0	100	0	0	0	0	0	0	0	0	0	100
0	0	0	0	0	0	0	0	0	0	100	0	0	0	0	0	0	0	0	0	100
0	0	0	0	0	0	100	0	0	0	100	0	0	0	0	0	0	0	0	0	100
0	0	0	0	0	0	100	0	0	0	100	0	0	0	0	0	0	0	0	0	100
0	0	0	0	0	0	0	0	0	0	100	0	0	0	0	0	0	0	0	0	100
0	0	0	0	0	0	100	0	0	0	100	0	0	0	0	0	0	0	0	0	100
0	0	0	0	0	0	0	0	0	0	100	0	0	0	0	0	0	0	0	0	100
0	0	0	0	0	0	0	0	0	0	100	0	0	0	0	0	0	0	0	0	100
0	0	0	0	0	0	100	0	0	0	100	0	0	0	0	0	0	0	0	0	100
0	0	0	0	0	0	100	0	0	0	100	0	0	0	0	0	0	0	0	0	100
0	0	0	0	0	0	100	0	0	0	100	0	0	0	0	0	0	0	0	0	100
0	0	0	0	0	0	100	0	0	0	100	0	0	0	0	0	0	0	0	0	100
0	0	0	0	0	0	0	0	0	0	100	0	0	0	0	0	0	0	0	0	0
0	0	0	0	0	0	0	0	0	0	100	0	0	0	0	0	0	0	0	0	100
0	0	0	0	0	0	0	0	0	0	100	0	0	0	0	0	0	0	0	0	100
0	0	0	0	0	0	0	0	0	0	100	0	0	0	0	0	0	0	0	0	0
0	0	0	0	0	0	0	0	0	0	100	0	0	0	0	0	0	0	0	0	0
0	0	0	0	0	0	0	0	0	0	100	0	0	0	0	0	0	0	0	0	100
0	0	0	0	0	0	0	0	0	0	100	0	0	0	0	0	0	0	0	0	100
0	0	0	0	0	0	0	0	0	0	100	0	0	0	0	0	0	0	0	0	100
0	0	0	0	0	0	0	0	0	0	100	0	0	0	0	0	0	0	0	0	100
0	0	0	0	0	0	0	0	0	0	100	0	0	0	0	0	0	0	0	0	100
0	0	0	0	0	0	0	0	0	0	100	0	0	0	0	0	0	0	0	0	100
0	0	0	0	0	0	0	0	0	0	100	0	0	0	0	0	0	0	0	0	100
0	0	0	0	0	0	0	0	0	0	100	0	0	0	0	0	0	0	0	0	100
0	0	0	0	0	0	0	0	0	0	100	0	0	0	0	0	0	0	0	0	100
0	0	0	0	0	0	0	0	0	0	100	0	0	0	0	0	0	0	0	0	100
0	0	0	0	0	0	0	0	0	0	100	0	0	0	0	0	0	0	0	0	100
0	0	0	0	0	0	0	0	0	0	100	0	0	0	0	0	0	0	0	0	100
0	0	0	0	0	0	0	0	0	0	100	0	0	0	0	0	0	0	0	0	100
0	0	0	0	0	0	0	0	0	0	100	0	0	0	0	0	0	0	0	0	100

0	0	0	0	0	0	0	0	0	0	100	0	0	0	0	0	0	0	0	0	100
0	0	0	0	0	0	0	0	0	0	100	0	0	0	0	0	0	0	0	0	100
0	0	0	0	0	0	0	0	0	0	100	0	0	0	0	0	0	0	0	0	100
0	0	0	0	0	0	0	0	0	0	100	0	0	0	0	0	0	0	0	0	100
0	0	0	0	0	0	0	0	0	0	100	0	0	0	0	0	0	0	0	0	100
0	0	0	0	0	0	0	0	0	0	100	0	0	0	0	0	0	0	0	0	100
100	0	0	0	0	0	0	0	0	97.96	100	0	0	0	0	0	0	0	0	0	0
0	0	0	0	0	0	0	0	0	0	0	0	0	0	0	0	0	0	0	0	0
0	0	0	0	0	0	0	0	0	0	0	0	0	0	0	0	0	0	0	0	0
0	0	0	0	0	0	0	0	0	0	100	0	0	0	0	0	0	0	0	0	100
0	0	0	0	0	0	0	0	0	0	100	0	0	0	0	0	0	0	0	0	100
0	0	0	0	0	0	0	0	0	0	100	0	0	0	0	0	0	0	0	0	100
0	0	0	0	0	0	0	0	0	0	100	0	0	0	0	0	0	0	0	0	100
0	0	0	0	0	0	0	0	0	0	100	0	0	0	0	0	0	0	0	0	100
0	0	0	0	0	0	0	0	0	0	100	0	0	0	0	0	0	0	0	0	100
0	0	0	0	0	0	0	0	0	0	100	0	0	0	0	0	0	0	0	0	100
0	0	0	0	0	0	0	0	0	0	100	0	0	0	0	0	0	0	0	0	100
0	0	0	0	0	0	0	0	0	0	100	0	0	0	0	0	0	0	0	0	100
0	0	0	0	0	0	0	0	0	0	100	0	0	0	0	0	0	0	0	0	100
0	0	0	0	0	0	0	0	0	0	100	0	0	0	0	0	0	0	0	0	100
0	0	0	0	0	0	0	0	0	0	100	0	0	0	0	0	0	0	0	0	100
0	0	0	0	0	0	0	0	0	0	100	0	0	0	0	0	0	0	0	0	100
0	0	0	0	0	0	0	0	0	0	100	0	0	0	0	0	0	0	0	0	100
0	0	0	0	0	0	0	0	0	0	100	0	0	0	0	0	0	0	0	0	100
0	0	0	0	0	0	0	0	0	0	100	0	0	0	0	0	0	0	0	0	100
0	0	0	0	0	0	0	0	0	0	100	0	0	0	0	0	0	0	0	0	100
0	0	0	0	0	0	0	0	0	0	100	0	0	0	0	0	0	0	0	0	100
0	0	0	0	0	0	0	0	0	0	100	0	0	0	0	0	0	0	0	0	100
0	0	0	0	0	0	0	0	0	0	100	0	0	0	0	0	0	0	0	0	100
0	0	0	0	0	0	0	0	0	0	100	0	0	0	0	0	0	0	0	0	100
0	0	0	0	0	0	0	0	0	0	100	0	0	0	0	0	0	0	0	0	100
0	0	0	0	0	0	0	0	0	0	100	0	0	0	0	0	0	0	0	0	100
0	0	0	0	0	0	0	0	0	0	100	0	0	0	0	0	0	0	0	0	100
0	0	0	0	0	0	0	0	0	0	100	0	0	0	0	0	0	0	0	0	100
0	0	0	0	0	0	0	0	0	0	100	0	0	0	0	0	0	0	0	0	100
0	0	0	0	0	0	0	0	0	0	100	0	0	0	0	0	0	0	0	0	100
0	0	0	0	0	0	0	0	0	0	100	0	0	0	0	0	0	0	0	0	100
0	0	0	0	0	0	0	0	0	0	100	0	0	0	0	0	0	0	0	0	100
0	0	0	0	0	0	0	0	0	0	100	0	0	0	0	0	0	0	0	0	100
0	0	0	0	0	0	0	0	0	0	100	0	0	0	0	0	0	0	0	0	100
0	0	0	0	0	0	0	0	0	0	100	0	0	0	0	0	0	0	0	0	100
0	0	0	0	0	0	0	0	0	0	100	0	0	0	0	0	0	0	0	0	100
0	0	0	0	0	0	0	0	0	0	100	0	0	0	0	0	0	0	0	0	100
0	0	0	0	0	0	0	0	0	0	100	0	0	0	0	0	0	0	0	0	100
0	0	0	0	0	0	0	0	0	0	100	0	0	0	0	0	0	0	0	0	100
0	0	0	0	0	0	0	0	0	0	100	0	0	0	0	0	0	0	0	0	100
0	0	0	0	0	0	0	0	0	0	100	0	0	0	0	0	0	0	0	0	100
0	0	0	0	0	0	0	0	0	0	100	0	0	0	0	0	0	0	0	0	100
0	0	0	0	0	0	0	0	0	0	100	0	0	0	0	0	0	0	0	0	100
0	0	0	0	0	0	0	0	0	0	100	0	0	0	0	0	0	0	0	0	100
0	0	0	0	0	0	0	0	0	0	100	0	0	0	0	0	0	0	0	0	100
0	0	0	0	0	0	0	0	0	0	100	0	0	0	0	0	0	0	0	0	100
0	0	0	0	0	0	0	0	0	0	100	0	0	0	0	0	0	0	0	0	100
0	0	0	0	0	0	0	0	0	0	100	0	0	0	0	0	0	0	0	0	100

0	0	0	0	0	0	0	0	0	0	100	0	0	0	0	0	0	0	0	0	100
0	0	0	0	0	0	0	0	0	0	100	0	0	0	0	0	0	0	0	0	100
0	0	0	0	0	0	0	0	0	0	100	0	0	0	0	0	0	0	0	0	100
0	0	0	0	0	0	0	0	0	0	0	0	0	0	0	0	0	0	0	0	0
0	0	0	0	0	0	0	0	0	0	0	0	0	0	0	0	0	0	0	0	0
0	0	0	0	0	0	0	0	0	0	0	0	0	0	0	0	0	0	0	0	0
0	0	0	0	0	0	0	0	0	0	0	0	0	0	0	0	0	0	0	0	0
0	0	0	0	0	0	0	0	0	0	0	0	0	0	0	0	0	0	0	0	0
0	0	0	0	0	0	0	0	0	0	0	0	0	0	0	0	0	0	0	0	0
0	0	0	0	0	0	0	0	0	0	0	0	0	0	0	0	0	0	0	0	0
0	0	0	0	0	0	0	0	0	0	0	0	0	0	0	0	0	0	0	0	0
0	0	0	0	0	0	0	0	0	0	0	0	0	0	0	0	0	0	0	0	0
0	0	0	0	0	0	0	0	0	0	0	0	0	0	0	0	0	0	0	0	0
0	0	0	0	0	0	0	0	0	0	0	0	0	0	0	0	0	0	0	0	0
0	0	0	0	0	0	0	0	0	0	0	0	0	0	0	0	0	0	0	0	0
0	0	0	0	0	0	0	0	0	0	0	0	0	0	0	0	0	0	0	0	0
0	0	0	0	0	0	0	0	0	0	0	0	0	0	0	0	0	0	0	0	0
0	0	0	0	0	0	0	0	0	0	0	0	0	0	0	0	0	0	0	0	0
0	0	0	0	0	0	0	0	0	0	0	0	0	0	0	0	0	0	0	0	0
0	0	0	0	0	0	0	0	0	0	0	0	0	0	0	0	0	0	0	0	0
0	0	0	0	0	0	0	0	0	0	0	0	0	0	0	0	0	0	0	0	0
0	0	0	0	0	0	0	0	0	0	0	0	0	0	0	0	0	0	0	0	0
0	0	0	0	0	0	0	0	0	0	0	0	0	0	0	0	0	0	0	0	0
0	0	0	0	0	0	0	0	0	0	0	0	0	0	0	0	0	0	0	0	0
0	0	0	0	0	0	0	0	0	0	0	0	0	0	0	0	0	0	0	0	0
0	0	0	0	0	0	0	0	0	0	0	0	0	0	0	0	0	0	0	0	0
0	0	0	0	0	0	0	0	0	0	0	0	0	0	0	0	0	0	0	0	0
0	0	0	0	0	0	0	0	0	0	0	0	0	0	0	0	0	0	0	0	0
0	0	0	0	0	0	0	0	0	0	0	0	0	0	0	0	0	0	0	0	0
0	0	0	0	0	0	0	0	0	0	0	0	0	0	0	0	0	0	0	0	0
0	0	0	0	0	0	0	0	0	0	0	0	0	0	0	0	0	0	0	0	0
0	0	0	0	0	0	0	0	0	0	0	0	0	0	0	0	0	0	0	0	0
0	0	0	0	0	0	0	0	0	0	0	0	0	0	0	0	0	0	0	0	0
0	0	0	0	0	0	0	0	0	0	0	0	0	0	0	0	0	0	0	0	0
0	0	0	0	0	0	0	0	0	0	0	0	0	0	0	0	0	0	0	0	0
0	0	0	0	0	0	0	0	0	0	0	0	0	0	0	0	0	0	0	0	0
0	0	0	0	0	0	0	0	0	0	0	0	0	0	0	0	0	0	0	0	0
0	0	0	0	0	0	0	0	0	0	0	0	0	0	0	0	0	0	0	0	0
0	0	0	0	0	0	0	0	0	0	0	0	0	0	0	0	0	0	0	0	0
0	0	0	0	0	0	0	0	0	0	100	0	0	0	0	0	0	0	0	0	100
0	0	0	0	0	0	0	0	0	0	0	0	0	0	0	0	0	0	0	0	0
0	0	0	0	0	0	0	0	0	0	0	0	0	0	0	0	0	0	0	0	0
0	0	0	0	0	0	0	0	0	0	0	0	0	0	0	0	0	0	0	0	0
0	0	0	0	0	0	0	0	0	0	0	0	0	0	0	0	0	0	0	0	0
0	0	0	0	0	0	0	0	0	0	0	0	0	0	0	0	0	0	0	0	0
0	0	0	0	0	0	0	0	0	0	0	0	0	0	0	0	0	0	0	0	0
0	0	0	0	0	0	0	0	0	0	0	0	0	0	0	0	0	0	0	0	0
0	0	0	0	0	0	0	0	0	0	0	0	0	0	0	0	0	0	0	0	0
0	0	0	0	0	0	0	0	0	0	0	0	0	0	0	0	0	0	0	0	0
0	0	0	0	0	0	0	0	0	0	0	0	0	0	0	0	0	0	0	0	0
0	0	0	0	0	0	0	0	0	0	0	0	0	0	0	0	0	0	0	0	0
0	0	0	0	0	0	0	0	0	97.96	100	0	0	0	0	0	0	0	0	0	0
0	0	0	0	0	0	0	0	0	97.96	100	0	0	0	0	0	0	0	0	0	0
0	0	0	0	0	0	0	0	0	97.96	100	0	0	0	0	0	0	0	0	0	0

0	0	0	0	0	0	0	0	0	97.96	100	0	0	0	0	0	0	0	0	0	0
0	0	0	0	0	0	0	0	0	97.96	100	0	0	0	0	0	0	0	0	0	0
0	0	0	0	0	0	0	0	0	97.96	100	0	0	0	0	0	0	0	0	0	0
0	0	0	0	0	0	0	0	0	97.96	100	0	0	0	0	0	0	0	0	0	0
0	0	0	0	0	0	0	0	0	97.96	100	0	0	0	0	0	0	0	0	0	100
0	0	0	0	0	0	0	0	0	97.96	100	0	0	0	0	0	0	0	0	0	100
0	0	0	0	0	0	0	0	0	97.96	100	0	0	0	0	0	0	0	0	0	0
0	0	0	0	0	0	0	0	0	97.96	100	0	0	0	0	0	0	0	0	0	0
0	0	0	0	0	0	0	0	0	97.96	100	0	0	0	0	0	0	0	0	0	0
0	0	0	0	0	0	0	0	0	97.96	100	0	0	0	0	0	0	0	0	0	0
0	0	0	0	0	0	0	0	0	97.96	100	0	0	0	0	0	0	0	0	0	100
0	0	0	0	0	0	0	0	0	97.96	100	0	0	0	0	0	0	0	0	0	0
0	0	0	0	0	0	0	0	0	97.96	100	0	0	0	0	0	0	0	0	0	100
0	0	0	0	0	0	0	0	0	97.96	100	0	0	0	0	0	0	0	0	0	0
0	0	0	0	0	0	0	0	0	97.96	100	0	0	0	0	0	0	0	0	0	0
0	0	0	0	0	0	0	0	0	97.96	100	0	0	0	0	0	0	0	0	0	0
0	0	0	0	0	0	0	0	0	97.96	100	0	0	0	0	0	0	0	0	0	100
0	0	0	0	0	0	0	0	0	97.96	100	0	0	0	0	0	0	0	0	0	0
0	0	0	0	0	0	0	0	0	97.96	100	0	0	0	0	0	0	0	0	0	100
0	0	0	0	0	0	0	0	0	97.96	100	0	0	0	0	0	0	0	0	0	100
0	0	0	0	0	0	0	0	0	97.96	100	0	0	0	0	0	0	0	0	0	0
0	0	0	0	0	0	0	0	0	97.96	100	0	0	0	0	0	0	0	0	0	0
0	0	0	0	0	0	0	0	0	97.96	100	0	0	0	0	0	0	0	0	0	100
0	0	0	0	0	0	0	0	0	97.96	100	0	0	0	0	0	0	0	0	0	0
0	0	0	0	0	0	0	0	0	97.96	100	0	0	0	0	0	0	0	0	0	100
0	0	0	0	0	0	0	0	0	97.96	100	0	0	0	0	0	0	0	0	0	100
0	0	0	0	0	0	0	0	0	97.96	100	0	0	0	0	0	0	0	0	0	0
0	0	0	0	0	0	0	0	0	97.96	100	0	0	0	0	0	0	0	0	0	0
0	0	0	0	0	0	0	0	0	97.96	100	0	0	0	0	0	0	0	0	0	0
0	0	0	0	0	0	0	0	0	97.96	100	0	0	0	0	0	0	0	0	0	0
0	0	0	0	0	0	0	0	0	97.96	100	0	0	0	0	0	0	0	0	0	0
0	0	0	0	0	0	0	0	0	97.96	100	0	0	0	0	0	0	0	0	0	0
0	0	0	0	0	0	0	0	0	97.96	100	0	0	0	0	0	0	0	0	0	0
0	0	0	0	0	0	0	0	0	0	0	0	0	0	0	0	0	0	0	0	0
0	0	0	0	0	0	0	0	0	0	0	0	0	100	0	0	0	0	0	0	0
0	0	0	0	0	0	0	0	0	0	0	0	0	0	0	0	0	0	0	0	0
0	0	0	0	0	0	0	0	0	97.96	100	0	0	0	0	0	0	0	0	0	100
0	0	0	0	0	0	0	0	0	97.96	100	0	0	0	0	0	0	0	0	0	0
0	0	0	0	0	0	0	0	0	97.96	100	0	0	0	0	0	0	0	0	0	0
0	0	0	0	0	0	0	0	0	0	0	0	0	0	0	0	0	0	0	0	0
0	0	0	0	0	0	0	0	0	0	99.67	0	0	0	0	0	0	0	0	0	100
0	0	0	0	0	0	0	0	0	0	99.67	0	0	0	0	0	0	0	0	0	100
0	0	0	0	0	0	0	0	0	0	99.67	0	0	0	0	0	0	0	0	0	100
0	0	0	0	0	0	0	0	0	0	99.67	0	0	0	0	0	0	0	0	0	100
0	0	0	0	0	0	0	0	0	0	0	0	0	0	0	0	0	0	0	0	0
0	0	0	0	0	0	0	0	0	0	99.67	0	0	0	0	0	0	0	0	0	100
0	0	0	0	0	0	0	0	0	0	0	0	0	0	0	0	0	0	0	0	0
0	0	0	0	0	0	0	0	0	0	99.67	0	0	0	0	0	0	0	0	0	100
0	0	0	0	0	0	0	0	0	0	99.67	0	0	0	0	0	0	0	0	0	100
0	0	0	0	0	0	0	0	0	0	99.67	0	0	0	0	0	0	0	0	0	100
0	0	0	0	0	0	0	0	0	0	0	0	0	0	0	0	0	0	0	0	0
0	0	0	0	0	0	0	0	0	97.96	100	0	0	0	0	0	0	0	0	0	100
0	0	0	0	0	0	0	0	0	0	0	0	0	0	0	0	0	0	0	0	0

0	0	0	0	0	0	0	0	0	0	99.67	0	0	0	0	0	0	0	0	0	100
0	0	0	0	0	0	0	0	0	0	100	0	0	0	0	0	0	0	0	0	100
0	0	0	0	0	0	0	0	0	0	0	0	0	0	0	0	0	0	0	0	0
0	0	0	0	0	0	0	0	0	0	0	0	0	0	0	0	0	0	0	0	0
0	0	0	0	0	0	0	0	0	0	0	0	0	0	0	0	0	0	0	0	0
0	0	0	0	0	0	0	0	0	0	0	0	0	0	0	0	0	0	0	0	0
0	0	0	0	0	0	0	0	0	0	0	0	0	0	0	0	0	0	0	0	0
0	0	0	0	0	0	0	0	0	0	0	0	0	0	0	0	0	0	0	0	0
0	0	0	0	0	0	0	0	0	0	0	0	0	0	0	0	0	0	0	0	0
0	0	0	0	0	0	0	0	0	0	0	0	0	0	0	0	0	0	0	0	0
0	0	0	0	0	0	0	0	0	0	0	0	0	0	0	0	0	0	0	0	0
0	0	0	0	0	0	0	0	0	0	0	0	0	0	0	0	0	0	0	0	0
0	0	0	0	0	0	0	0	0	0	0	0	0	0	0	0	0	0	0	0	0
0	0	0	0	0	0	0	0	0	0	0	0	0	0	0	0	0	0	0	0	0
0	0	0	0	0	0	0	0	0	0	0	0	0	0	0	0	0	0	0	0	0
0	0	0	0	0	0	0	0	0	0	0	0	0	0	0	0	0	0	0	0	0
0	0	0	0	0	0	0	0	0	0	0	0	0	0	0	0	0	0	0	0	0
0	0	0	0	0	0	0	0	0	0	0	0	0	0	0	0	0	0	0	0	0
0	0	0	0	0	0	0	0	0	0	0	0	0	0	0	0	0	0	0	0	0
0	0	0	0	0	0	0	0	0	0	0	0	0	0	0	0	0	0	0	0	0
100	0	0	0	0	100	0	0	0	0	0	0	0	0	0	0	0	0	0	0	0
0	0	0	0	0	0	0	0	0	0	0	0	0	0	0	0	0	0	0	0	100
0	0	0	0	0	0	0	0	0	0	0	0	0	0	0	0	0	0	0	0	0
0	0	0	0	0	0	0	0	0	0	0	0	0	100	0	0	0	0	0	0	0
0	0	0	0	0	0	0	0	0	0	0	0	0	0	0	0	0	0	0	0	0
0	0	0	0	0	0	0	0	0	0	0	0	0	0	0	0	0	0	0	0	0
0	0	0	0	0	0	0	0	0	0	0	0	0	0	0	0	0	0	0	0	0
0	0	0	0	0	0	0	0	0	0	0	0	0	0	0	0	0	0	0	0	0
0	0	0	0	0	0	0	0	0	0	0	0	0	0	0	0	0	0	0	0	0
0	0	0	0	0	0	0	0	0	0	0	0	0	0	0	0	0	0	0	0	0
0	0	0	0	0	0	0	0	0	0	0	0	0	0	0	0	0	0	0	0	0
0	0	0	0	0	0	0	0	0	0	0	0	0	0	0	0	0	0	0	0	0
0	0	0	0	0	0	0	0	0	0	0	0	0	0	0	0	0	0	0	0	0
0	0	0	0	0	0	0	0	0	0	0	0	0	0	0	0	0	0	0	0	0
0	0	0	0	0	0	0	0	0	0	0	0	0	0	0	0	0	0	0	0	0
0	0	0	0	0	0	0	0	0	0	0	0	0	0	0	0	0	0	0	0	0
0	0	0	0	0	0	0	0	0	0	0	0	0	0	0	0	0	0	0	0	0
0	0	0	0	0	0	0	0	0	0	0	0	0	0	0	0	0	0	0	0	0
0	0	0	0	0	0	0	0	0	0	0	0	0	0	0	0	0	0	0	0	0
0	0	0	0	0	0	0	0	0	0	0	0	0	0	0	0	0	0	0	0	0
0	0	0	0	0	0	0	0	0	0	0	0	0	0	0	0	0	0	0	0	0
0	0	0	0	0	0	0	0	0	0	0	0	0	0	0	0	0	0	0	0	0
0	0	0	0	0	0	0	0	0	0	0	0	0	0	0	0	0	0	0	0	0
0	0	0	0	0	0	0	0	0	0	0	0	0	0	0	0	0	0	0	0	0
0	0	0	0	0	0	0	0	0	0	0	0	0	0	0	0	0	0	0	0	0
0	0	0	0	0	0	0	0	0	0	0	0	0	0	0	0	0	0	0	0	0
0	0	0	0	0	0	0	0	0	0	0	0	0	0	0	0	0	0	0	0	0
0	0	0	0	0	0	0	0	100	0	0	0	0	0	0	0	0	0	0	0	0
0	0	0	0	0	0	0	0	0	0	0	0	0	0	0	0	0	0	0	0	0
0	0	0	0	0	0	0	0	0	0	0	0	0	0	0	0	0	0	0	0	0
0	0	0	0	0	0	0	0	0	0	0	0	0	0	0	0	0	0	0	0	0
0	0	0	0	0	0	0	0	0	0	0	0	0	0	0	0	0	0	0	0	0
0	0	0	0	0	0	0	0	0	0	0	0	0	0	0	0	0	0	0	0	0

0	0	0	0	0	0	0	0	0	0	0	0	0	0	44.44	0	0	0	0	0	0
0	0	0	0	0	0	0	0	0	0	0	0	0	0	0	0	0	0	0	0	0
0	0	0	0	0	0	0	0	0	0	0	0	0	0	0	0	0	0	0	0	0
0	0	0	0	0	0	0	0	0	0	0	0	0	0	0	0	0	0	0	0	0
0	0	0	0	0	0	0	0	0	0	0	0	0	0	0	0	0	0	0	0	0
0	0	0	0	0	0	0	0	0	0	0	0	0	0	44.44	0	0	0	0	0	0
0	0	0	0	0	0	0	0	100	0	0	0	0	0	0	0	0	0	0	0	0
0	0	0	0	0	0	0	0	0	0	0	0	0	0	0	0	0	0	0	0	0
0	0	0	0	0	0	0	0	0	0	0	0	0	0	0	0	0	0	0	0	0
0	0	0	0	0	0	0	0	0	0	100	0	0	0	0	0	0	0	0	0	0
0	0	0	0	0	0	0	0	0	0	0	0	0	0	0	0	0	0	0	0	0
0	0	0	0	0	0	0	0	0	0	0	0	0	0	0	0	0	0	0	0	0
0	0	0	0	0	0	0	0	0	0	0	0	0	0	0	0	0	0	0	0	0
0	0	0	0	0	0	0	0	0	0	0	0	0	0	0	0	0	0	0	0	0
0	0	0	0	0	0	0	0	0	0	0	0	0	100	0	0	0	0	0	0	100
0	0	0	0	0	0	0	0	0	0	0	0	0	0	0	0	0	0	0	0	0
0	0	0	0	0	0	0	0	0	0	0	0	0	0	0	0	0	0	0	0	0
0	0	0	0	0	0	0	0	0	0	0	0	0	100	0	0	0	0	0	0	100
0	0	0	0	0	0	0	0	0	0	0	0	0	100	0	0	0	0	0	0	100
0	0	0	0	0	0	0	0	0	0	0	0	0	100	0	0	0	0	0	0	100
0	0	0	0	0	0	0	0	0	0	0	0	0	0	0	0	0	0	0	0	0
0	0	0	0	0	0	0	0	0	0	0	0	0	0	0	0	0	0	0	0	0
0	0	0	0	0	0	0	0	0	0	0	0	0	100	0	0	0	0	0	0	100
0	0	0	0	0	0	0	0	0	0	0	0	0	0	0	0	0	0	0	0	0
0	0	0	0	0	0	0	0	0	0	0	0	0	0	0	0	0	0	0	0	0
0	0	0	0	0	0	0	0	0	0	100	0	0	0	0	0	0	0	0	0	99.64
0	0	0	0	0	0	0	0	0	0	0	0	0	0	0	0	0	0	0	0	0
0	0	0	0	0	0	0	0	0	0	0	0	0	0	44.44	0	0	0	0	0	100
0	0	0	0	0	0	0	0	0	0	0	0	0	100	0	0	0	0	0	0	100
0	0	0	0	0	0	0	0	0	0	0	0	0	0	44.44	0	0	0	0	0	100
0	0	0	0	0	0	0	0	0	0	0	0	0	0	44.44	0	0	0	0	0	0
0	0	100	100	0	0	0	0	0	0	100	0	0	0	0	0	0	0	0	0	100
0	0	0	0	0	0	0	0	0	0	0	0	0	0	0	0	0	0	0	0	0
0	0	0	0	0	0	0	0	0	0	0	0	0	0	0	0	0	0	0	0	0
0	0	0	0	0	0	0	0	0	0	0	0	0	0	0	0	0	0	0	0	0
0	0	0	0	0	0	0	0	0	0	0	0	0	0	0	0	0	0	0	0	0
0	0	0	0	0	0	0	0	0	0	0	0	0	0	44.44	0	0	0	0	0	0
0	0	0	0	0	0	0	0	0	0	0	0	0	0	44.44	0	0	0	0	0	0
0	0	0	0	0	0	0	0	0	0	0	0	0	0	0	0	0	0	0	0	0
0	0	0	0	0	0	0	0	0	0	0	0	0	0	0	0	0	0	0	0	0
0	0	0	0	0	0	0	0	0	0	0	0	0	0	0	0	0	0	0	0	0
0	0	0	0	0	0	0	0	0	0	0	0	0	0	0	0	0	0	0	0	0
0	0	0	0	0	0	0	0	0	0	0	0	0	100	0	0	0	0	0	0	0
0	0	0	0	0	0	0	0	0	0	0	0	0	0	44.44	0	0	0	0	0	0
0	0	0	0	0	0	0	0	0	0	0	0	0	0	0	0	0	0	0	0	0
0	0	0	0	0	0	0	0	0	0	0	0	0	0	0	0	0	0	0	0	0

sul2	tet(A)	tetR	dfrA1	dfrA12	dfrA14	dfrA17	dfrA5	dfrA7	dfrA8	dfrK
100	100	0	99.36	0	0	100	0	0	0	0
100	100	0	99.36	100	0	0	0	0	0	0
100	100	0	99.36	100	0	0	0	0	0	0
100	100	0	99.36	100	0	0	0	0	0	0
100	100	0	99.36	0	0	0	0	0	0	0
0	0	0	0	0	0	0	0	0	0	0
100	100	0	99.36	0	0	0	0	0	0	0
100	100	0	99.36	0	0	100	0	0	0	0
0	100	0	99.36	0	0	0	0	0	0	0
0	0	0	99.36	0	0	0	0	0	0	0
100	100	0	99.36	100	0	0	0	0	0	0
100	100	0	99.36	0	0	0	0	0	0	0
0	0	0	99.36	0	0	0	0	0	0	0
100	100	0	99.36	0	0	0	0	0	0	0
100	100	0	99.36	0	0	0	0	0	0	0
100	100	0	99.36	0	0	0	0	0	0	0
100	100	0	99.36	0	0	0	0	0	0	0
100	100	0	99.36	0	0	0	0	0	0	0
100	100	0	99.36	0	0	0	0	0	0	0
100	100	0	99.36	0	0	0	0	0	0	0
100	100	0	99.36	0	0	0	0	0	0	0
100	100	0	99.36	0	0	0	0	0	0	0
100	100	0	99.36	0	0	0	0	0	0	0
100	100	0	99.36	0	0	0	0	0	0	0
100	100	0	99.36	0	0	0	0	0	0	0
100	100	0	99.36	0	0	0	0	0	0	0
100	100	0	99.36	0	0	0	0	0	0	0
100	100	0	99.36	0	0	0	0	0	0	0
100	100	0	99.36	0	0	0	0	0	0	0
100	100	0	99.36	0	0	0	0	0	0	0
100	100	0	99.36	0	0	0	0	0	0	0
100	100	0	99.36	0	0	0	0	0	0	0
100	100	0	99.36	0	0	0	0	0	0	0
100	100	0	99.36	0	0	0	0	0	0	0
100	100	0	99.36	0	0	0	0	0	0	0
100	100	0	99.36	0	0	0	0	0	0	0
100	100	0	99.36	0	0	0	0	0	0	0
100	100	0	99.36	0	0	0	0	0	0	0
100	100	0	99.36	0	0	0	0	0	0	0
100	100	0	99.36	0	0	0	0	0	0	0
100	100	0	99.36	0	0	0	0	0	0	0
100	100	0	99.36	0	0	0	0	0	0	0
100	100	0	99.36	0	0	0	0	0	0	0
100	100	0	99.36	0	0	0	0	0	0	0
100	100	0	99.36	0	0	0	0	0	0	0
100	100	0	99.36	0	0	0	0	0	0	0
100	100	0	99.36	0	0	0	0	0	0	0
100	100	0	99.36	0	0	0	0	0	0	0
100	100	0	99.36	0	0	0	0	0	0	0
100	100	0	99.36	0	0	0	0	0	0	0
100	100	0	99.36	0	0	0	0	0	0	0

100	100	0	99.36	100	0	0	0	0	0	0
0	0	0	99.36	0	0	0	0	0	0	0
100	100	0	99.36	100	0	0	0	0	0	0
100	100	0	99.36	100	0	100	0	0	0	0
100	100	0	99.36	0	0	100	0	0	0	0
100	100	0	98.09	100	0	0	0	0	0	0
100	100	0	99.36	100	0	0	0	0	0	0
100	100	0	99.36	0	0	0	0	0	0	0
100	100	0	99.36	100	0	0	0	0	0	0
100	100	0	99.36	100	0	0	0	0	0	0
0	0	0	99.36	0	0	0	0	0	0	0
0	0	0	0	0	0	0	0	0	0	0
100	100	0	99.36	100	0	0	0	0	0	0
100	100	0	99.36	100	0	0	0	0	0	0
100	100	0	99.36	0	0	0	0	0	0	0
100	100	0	99.36	0	0	0	0	0	0	0
100	100	0	99.36	100	0	0	0	0	0	0
100	100	0	99.36	0	0	0	0	0	0	0
100	100	0	99.36	0	0	0	0	0	0	0
100	100	0	99.36	100	0	0	0	0	0	0
0	0	0	99.36	0	0	0	0	0	0	0
100	100	0	99.36	100	0	100	0	0	0	0
100	100	0	99.36	100	0	0	0	0	0	0
100	100	0	99.36	100	0	100	0	0	0	0
0	100	0	99.36	0	0	0	0	0	0	0
100	0	0	99.36	0	0	0	0	0	0	0
100	100	0	99.36	100	0	0	0	0	0	0
100	100	0	99.36	100	0	0	0	0	0	0
100	100	0	99.36	100	0	0	0	0	0	0
100	100	0	99.36	100	0	0	0	0	0	0
100	100	0	99.36	0	0	0	0	0	0	0
100	100	0	99.36	0	0	0	0	0	0	0
100	100	0	99.36	0	0	0	0	0	0	0
100	100	100	99.36	0	0	0	0	0	0	0
100	100	0	99.36	0	0	0	0	0	0	0
100	100	0	99.36	100	0	0	0	0	0	0
100	100	0	99.36	100	0	100	0	0	0	0
100	100	0	99.36	0	0	0	0	0	0	0
100	100	0	99.36	0	0	0	0	0	0	0
100	100	0	99.36	0	0	0	0	0	0	0
100	100	0	99.36	0	0	0	0	0	0	0
100	100	0	99.36	100	0	0	0	0	0	0
100	100	0	99.36	0	0	0	0	0	0	0
100	100	0	99.36	100	0	0	0	0	0	0
100	100	0	99.36	0	0	0	0	0	0	0
100	100	0	99.36	0	0	0	0	0	0	0
100	0	0	99.36	0	0	0	0	0	0	0
100	100	0	99.36	0	0	0	0	0	0	0
100	100	0	99.36	0	0	0	0	0	0	0
100	100	0	99.36	100	0	0	0	0	0	0
100	100	0	99.36	100	0	0	0	0	0	0
100	100	0	99.36	100	0	0	0	0	0	0
100	100	0	99.36	0	0	0	0	0	0	0
100	100	0	99.36	100	0	0	0	0	0	0

100	100	0	99.36	100	0	0	0	0	0	0
100	100	0	99.36	0	0	0	0	0	0	0
100	100	0	99.36	100	0	0	0	0	0	0
100	100	0	99.36	0	0	0	0	0	0	0
100	100	0	99.36	100	0	0	0	0	0	0
100	100	0	99.36	100	0	0	0	0	0	0
0	0	0	99.36	0	0	0	0	0	0	0
100	100	0	99.36	0	0	0	0	0	0	0
0	0	0	0	0	0	0	0	0	0	0
100	100	0	99.36	0	0	0	0	0	0	0
100	100	0	99.36	100	0	0	0	0	0	0
100	100	0	99.36	0	0	0	0	0	0	0
100	100	0	99.36	0	0	0	0	0	0	0
100	0	0	99.36	0	0	0	0	0	100	0
0	0	0	99.36	0	0	0	0	0	0	0
100	0	0	99.36	0	0	0	0	0	100	0
0	0	0	99.36	0	0	0	0	0	0	0
100	100	0	99.36	0	0	0	0	0	0	0
100	100	0	99.36	0	0	0	0	0	0	0
100	100	0	99.36	0	0	0	0	0	0	0
100	100	0	99.36	0	0	0	0	0	0	0
100	0	0	99.36	0	0	0	0	0	0	0
100	100	0	99.36	0	0	0	0	0	0	0
100	100	0	99.36	0	0	0	0	0	0	0
100	100	0	99.36	100	0	0	0	0	0	0
0	0	0	99.36	0	0	0	0	0	0	0
100	100	0	0	100	0	0	0	0	0	0
100	100	0	0	100	0	0	0	0	0	0
100	100	0	99.36	100	0	0	0	0	0	0
100	100	0	99.36	100	0	0	0	0	0	0
100	100	0	99.36	100	0	0	0	0	0	0
100	100	0	99.36	0	0	0	0	0	0	0
100	100	0	99.36	100	0	0	0	0	0	0
100	100	0	99.36	100	0	0	0	0	0	0
100	100	0	99.36	100	0	0	0	0	0	0
100	100	0	99.36	100	0	0	0	0	0	0
100	100	0	99.36	0	0	0	0	0	0	0
0	0	0	99.36	0	0	0	0	0	0	0
100	100	0	99.36	100	0	0	0	0	0	0
100	100	0	99.36	100	0	0	0	0	0	0
100	100	0	99.36	0	0	0	0	0	0	0
100	100	0	99.36	100	0	0	0	0	0	0
100	100	0	99.36	100	0	0	0	0	0	0
100	0	100	99.36	0	0	0	0	0	0	0
0	0	0	99.36	0	0	0	0	0	0	0
100	100	0	99.36	0	0	0	0	0	0	0
100	100	0	99.36	100	0	0	0	0	0	0
0	0	0	99.36	0	0	0	0	0	0	0
100	100	0	99.36	0	0	0	0	0	0	0
100	100	0	99.36	100	0	0	0	0	0	0
0	0	0	99.36	0	0	0	0	0	0	0
100	100	0	99.36	0	0	0	0	0	0	0
100	100	0	99.36	0	0	0	0	0	0	0
100	100	0	99.36	0	0	0	0	0	0	0

100	100	0	99.36	100	0	0	0	0	0	0
100	100	0	99.36	100	0	0	0	0	0	0
100	100	0	99.36	100	0	0	0	0	0	0
100	100	0	99.36	100	0	0	0	0	0	0
100	100	0	99.36	0	0	0	0	0	0	0
100	100	0	0	100	0	0	0	0	0	0
100	100	0	99.36	100	0	0	0	0	0	0
100	100	0	99.36	100	0	0	0	0	0	0
0	0	0	99.36	0	0	0	0	0	0	0
100	100	0	99.36	0	0	0	0	0	0	0
100	100	0	99.36	100	0	0	0	0	0	0
100	100	0	99.36	0	0	0	0	0	0	0
0	0	0	0	0	0	0	0	0	0	0
100	100	0	99.36	100	0	0	0	0	0	0
100	100	0	99.36	100	0	0	0	0	0	0
100	100	0	99.36	100	0	0	0	0	0	0
100	0	0	99.36	0	0	0	0	0	0	0
100	0	0	99.36	0	0	0	0	0	0	0
100	100	0	99.36	0	0	0	0	0	0	0
100	0	0	99.36	0	0	0	0	0	0	0
100	100	0	99.36	0	0	0	0	0	0	0
100	100	0	99.36	0	0	0	0	0	0	0
100	100	0	99.36	0	0	0	0	0	0	0
100	100	0	99.36	100	0	0	0	0	0	0
100	100	0	99.36	100	0	0	0	0	0	0
100	100	0	99.36	100	0	0	0	0	0	0
100	100	0	99.36	0	0	0	0	0	0	0
100	100	0	99.36	0	0	0	0	0	0	0
100	100	0	99.36	0	0	0	0	0	0	0
100	100	0	99.36	0	0	0	0	0	0	0
100	100	0	99.36	0	0	100	0	0	0	0
100	0	100	99.36	0	0	0	0	0	0	0
100	100	0	0	0	0	0	0	0	0	0
100	100	0	99.36	100	0	0	0	0	0	0
100	0	100	99.36	0	0	0	0	0	0	0
100	100	0	99.36	100	0	0	0	0	0	0
0	0	0	99.36	0	0	0	0	0	0	0
100	100	0	99.36	100	0	0	0	0	0	0
100	100	0	99.36	100	0	0	0	0	0	0
0	0	0	99.36	0	0	0	0	0	0	0
0	0	0	99.36	0	0	0	0	0	0	0
100	100	0	99.36	100	0	0	0	0	0	0
0	0	0	99.36	0	0	0	0	0	0	0
0	0	0	99.36	0	0	0	0	0	0	0
100	100	0	99.36	100	0	0	0	0	0	0
0	0	0	99.36	0	0	0	0	0	0	0
100	100	0	99.36	0	0	0	0	0	0	0
100	100	0	99.36	100	0	0	0	0	0	0
0	0	0	99.36	0	0	0	0	0	0	0
100	100	0	99.36	100	0	0	0	0	0	0
100	100	0	99.36	100	0	0	0	0	0	0
100	100	0	99.36	100	0	0	0	0	0	0
100	100	0	99.36	100	0	0	0	0	0	0
100	100	0	99.36	100	0	0	0	0	0	0

100	100	0	99.36	100	0	0	0	0	0	0
100	100	0	99.36	100	0	0	0	0	0	0
100	100	0	99.36	100	0	0	0	0	0	0
0	0	0	99.36	0	0	0	0	0	0	0
0	0	0	99.36	0	0	0	0	0	0	0
0	0	0	99.36	0	0	0	0	0	0	0
100	100	0	99.36	0	0	0	0	0	0	0
100	100	0	99.36	0	0	0	0	0	0	0
100	100	0	99.36	100	0	0	0	0	0	0
100	100	0	99.36	0	0	0	0	0	0	0
100	100	0	99.36	100	0	0	0	0	0	0
100	100	0	99.36	100	0	0	0	0	0	0
100	100	0	99.36	0	0	0	0	0	0	0
100	100	0	99.36	100	0	0	0	0	0	0
0	0	0	0	0	0	0	0	0	0	0
0	0	0	0	0	0	0	0	0	0	0
100	100	0	99.36	0	0	0	0	0	0	0
100	100	0	99.36	100	0	0	0	0	0	0
100	100	0	99.36	100	0	0	0	0	0	0
100	100	0	99.36	100	0	0	0	0	0	0
0	0	0	99.36	0	0	0	0	0	0	0
100	100	0	99.36	0	0	0	0	0	0	0
100	100	0	99.36	100	0	0	0	0	0	0
100	100	0	99.36	0	0	100	0	0	0	0
0	0	0	98.73	0	0	0	0	0	0	0
100	100	0	99.36	100	0	0	0	0	0	0
0	0	0	99.36	0	0	0	0	0	0	0
0	0	0	99.36	0	0	0	0	0	0	0
100	100	0	99.36	100	0	0	0	0	0	0
100	100	0	99.36	0	0	0	0	0	0	0
0	0	0	99.36	0	0	0	0	0	0	0
100	100	0	99.36	100	0	0	0	0	0	0
100	100	0	99.36	100	0	0	0	0	0	0
0	0	0	99.36	0	0	0	0	0	0	0
100	100	0	99.36	100	0	0	0	0	0	0
100	100	0	99.36	100	0	100	0	0	0	0
100	100	0	99.36	100	0	0	0	0	0	0
100	100	0	99.36	100	0	0	0	0	0	0
0	0	0	99.36	0	0	100	0	0	0	0
0	0	0	0	0	0	0	0	0	0	0
100	100	0	99.36	100	0	0	0	0	0	0
100	100	0	99.36	100	0	0	0	0	0	0
0	0	0	99.36	0	0	0	0	0	0	0
100	100	0	99.36	100	0	0	0	0	0	0
0	0	0	0	0	0	0	0	0	0	0
100	100	0	99.36	100	0	0	0	0	0	0
100	100	0	99.36	100	0	0	0	0	0	0
0	0	0	0	0	0	0	0	0	0	0
0	0	0	99.36	0	0	0	0	0	0	0
100	100	0	99.36	100	0	0	0	0	0	0
0	0	0	99.36	0	0	0	0	0	0	0
100	100	0	99.36	0	0	100	0	0	0	0
0	0	0	99.36	0	0	0	0	0	0	0
100	100	0	99.36	100	0	0	0	0	0	0

100	100	0	99.36	0	0	100	0	0	0	0
100	100	0	99.36	100	0	0	0	0	0	0
0	0	0	99.36	0	0	0	0	0	0	0
100	100	0	99.36	100	0	0	0	0	0	0
0	0	0	99.36	0	0	0	0	0	0	0
0	0	0	99.36	0	0	0	0	0	0	0
0	0	0	99.36	0	0	0	0	0	0	0
0	0	0	99.36	0	0	0	0	0	0	0
0	0	0	99.36	0	0	0	0	0	0	0
0	0	0	99.36	0	0	0	0	0	0	0
100	100	0	99.36	100	0	0	0	0	0	0
100	100	0	99.36	0	0	0	0	0	0	0
0	0	0	99.36	0	0	0	0	0	0	0
0	0	0	99.36	0	0	0	0	0	0	0
100	100	0	99.36	100	0	0	0	0	0	0
0	0	0	99.36	0	0	100	0	0	0	0
100	100	0	99.36	100	0	0	0	0	0	0
100	100	0	99.36	100	0	0	0	0	0	0
100	100	0	99.36	100	0	0	0	0	0	0
0	0	0	99.36	0	0	100	0	0	0	0
0	0	0	99.36	0	0	0	0	0	0	0
100	100	0	99.36	100	0	0	0	0	0	0
100	100	0	99.36	100	0	0	0	0	0	0
100	100	0	99.36	0	0	0	0	0	0	0
100	100	0	99.36	0	0	0	0	0	0	0
0	0	0	99.36	0	0	100	0	0	0	0
0	0	0	99.36	0	0	0	0	0	0	0
100	100	0	99.36	100	0	0	0	0	0	0
100	100	0	99.36	100	0	0	0	0	0	0
100	100	0	99.36	0	0	100	0	0	0	0
100	100	0	99.36	0	0	0	0	0	0	0
0	0	0	99.36	0	0	0	0	0	0	0
100	100	0	99.36	100	0	0	0	0	0	0
100	100	0	99.36	100	0	0	0	0	0	0
100	100	0	99.36	0	0	0	0	0	0	0
100	100	0	99.36	100	0	0	0	0	0	0
100	100	0	99.36	100	0	0	0	0	0	0
100	100	0	99.36	100	0	0	0	0	0	0
100	100	0	99.36	100	0	0	0	0	0	0
100	100	0	99.36	100	0	0	0	0	0	0
100	100	0	99.36	100	0	0	0	0	0	0
100	100	0	99.36	0	0	0	0	0	0	0
100	100	0	99.36	100	0	0	0	0	0	0
100	100	0	99.36	0	0	100	0	0	0	0
100	100	0	99.36	0	0	0	0	0	0	0
100	100	0	99.36	100	0	0	0	0	0	0
100	100	0	99.36	100	0	0	0	0	0	0
0	0	0	99.36	0	0	0	0	0	0	0
0	0	0	99.36	0	0	0	0	0	0	0
100	100	0	99.36	100	0	0	0	0	0	0
100	100	0	99.36	0	0	0	0	0	0	0
100	100	0	99.36	100	0	0	0	0	0	0
0	0	0	99.36	0	0	0	0	0	0	0
0	0	0	99.36	0	0	0	0	0	0	0

0	0	0	99.36	0	0	100	0	0	0	0
0	0	0	99.36	0	0	0	0	0	0	0
0	0	0	99.36	0	0	100	0	0	0	0
0	0	0	99.36	0	0	100	0	0	0	0
100	100	0	99.36	100	0	100	0	0	0	0
100	0	0	0	0	0	100	0	99.36	0	0
100	100	0	99.36	100	0	0	0	0	0	0
0	0	0	99.36	0	0	100	0	0	0	0
0	0	0	99.36	0	0	100	0	0	0	0
0	0	0	99.36	0	0	100	0	0	0	0
0	0	0	99.36	0	0	100	0	0	0	0
100	100	0	99.36	100	0	0	0	0	0	0
100	100	0	99.36	100	0	0	0	0	0	0
100	100	0	99.36	0	0	0	0	0	0	0
100	100	0	99.36	100	0	0	0	0	0	0
0	0	0	99.36	0	0	0	0	0	0	0
100	100	0	99.36	100	0	0	0	0	0	0
100	100	0	99.36	0	0	0	0	0	0	0
0	0	0	99.36	0	0	0	0	0	0	0
0	0	0	99.36	0	0	0	0	0	0	0
0	0	0	99.36	0	0	0	0	0	0	0
0	0	0	99.36	0	0	100	0	0	0	0
100	100	0	99.36	100	0	0	0	0	0	0
0	100	0	99.36	0	0	100	0	0	0	0
100	0	0	99.36	0	0	0	0	0	0	0
100	100	0	99.36	100	0	0	0	0	0	0
0	100	0	99.36	0	0	100	0	0	0	0
0	100	0	99.36	0	0	100	0	0	0	0
0	0	0	99.36	0	0	0	0	0	0	0
100	100	0	99.36	100	0	0	0	0	0	0
0	0	0	99.36	0	0	0	0	0	0	0
0	0	0	99.36	0	0	0	0	0	0	0
100	100	0	99.36	100	0	0	0	0	0	0
100	100	0	99.36	100	0	0	0	0	0	0
0	0	0	99.36	0	0	0	0	0	0	0
0	0	0	99.36	0	0	0	0	0	0	0
0	0	0	99.36	0	0	0	0	0	0	0
0	0	0	99.36	0	0	0	0	0	0	0
0	0	0	99.36	0	0	0	0	0	0	0
0	0	0	99.36	0	0	0	0	0	0	0
0	0	0	99.36	0	0	0	0	0	0	0
0	0	0	99.36	0	0	0	0	0	0	0
100	100	0	99.36	100	0	0	0	0	0	0
0	0	0	99.36	0	0	0	0	0	0	0
0	0	0	99.36	0	0	0	0	0	0	0
100	100	0	99.36	100	0	0	0	0	0	0
0	0	0	99.36	0	0	0	0	0	0	0
100	100	0	99.36	0	98.73	0	0	0	0	0
100	100	0	99.36	0	0	0	0	0	0	0
100	100	0	99.36	100	0	0	0	0	0	0
0	0	0	99.36	0	0	0	0	0	0	0
0	0	0	99.36	0	0	0	0	0	0	0
100	100	0	99.36	100	0	100	0	0	0	0
100	100	0	99.36	0	0	0	0	0	0	0

100	100	0	99.36	100	0	0	0	0	0	0
100	100	0	99.36	100	0	0	0	0	0	0
100	100	0	99.36	99.34	0	0	0	0	0	0
0	0	0	99.36	0	0	0	0	0	0	0
0	0	0	99.36	0	0	0	0	0	0	0
0	100	0	99.36	0	0	100	0	0	0	0
100	100	0	0	100	0	0	0	0	0	0
100	100	0	99.36	100	0	0	0	0	0	0
0	0	0	99.36	0	0	0	0	0	0	0
100	100	0	99.36	0	0	100	0	0	0	0
100	100	0	99.36	0	0	100	0	0	0	0
100	100	0	99.36	100	0	0	0	0	0	0
100	100	0	99.36	0	0	0	0	0	0	0
0	0	0	99.36	0	0	0	0	0	0	0
100	100	0	99.36	100	0	0	0	0	0	0
0	100	0	99.36	0	0	100	0	0	0	0
0	0	0	99.36	0	0	0	0	0	0	0
100	100	0	99.36	100	0	0	0	0	0	0
100	100	0	99.36	100	0	0	0	0	0	0
100	100	0	99.36	100	0	0	0	0	0	0
100	100	0	99.36	100	0	0	0	0	0	0
100	100	0	99.36	100	0	0	0	0	0	0
0	100	0	99.36	0	0	100	0	0	0	0
0	100	0	99.36	0	0	100	0	0	0	0
100	100	0	99.36	0	0	0	0	0	0	0
0	100	0	99.36	0	0	100	0	0	0	0
100	100	0	99.36	0	0	0	0	0	0	0
100	100	0	99.36	0	0	0	0	0	0	0
100	100	0	99.36	0	0	0	0	0	0	0
100	100	0	99.36	0	0	0	0	0	0	0
100	100	0	99.36	0	0	0	0	0	0	0
100	100	0	99.36	0	0	0	0	0	0	0
100	100	0	99.36	0	0	0	0	0	0	0
100	100	0	99.36	0	0	0	0	0	0	0
100	100	0	99.36	0	0	0	0	0	0	0
100	100	0	99.36	0	0	0	0	0	0	0
100	100	0	99.36	0	0	0	0	0	0	0
100	100	0	99.36	0	0	0	0	0	0	0
100	100	0	99.36	0	0	0	0	0	0	0
100	100	0	99.36	0	0	100	0	0	0	0
100	100	0	99.36	0	0	100	0	0	0	0
100	100	0	99.36	0	0	100	0	0	0	0
100	100	0	99.36	0	0	100	0	0	0	0
100	100	0	99.36	0	0	100	0	0	0	0
100	100	0	99.36	0	0	100	0	0	0	0
100	100	0	99.36	0	0	100	0	0	0	0
100	100	0	99.36	0	0	100	0	0	0	0
0	0	0	99.36	0	0	0	0	0	0	0
0	0	0	99.36	0	0	0	0	0	0	0
100	100	0	99.36	0	0	100	0	0	0	0
100	100	0	99.36	0	0	100	0	0	0	0
100	100	0	99.36	0	0	100	0	0	0	0
100	100	0	99.36	0	0	100	0	0	0	0
100	100	0	99.36	0	0	100	0	0	0	0

100	100	0	99.36	0	0	100	0	0	0	0
100	100	0	99.36	0	0	100	0	0	0	0
100	100	0	99.36	0	0	100	0	0	0	0
100	100	0	99.36	0	0	100	0	0	0	0
100	100	0	99.36	0	0	100	0	0	0	0
100	100	0	99.36	0	0	100	0	0	0	0
100	100	0	99.36	0	0	100	0	0	0	0
100	100	0	99.36	0	0	100	0	0	0	0
100	100	0	99.36	0	0	100	0	0	0	0
100	100	0	99.36	0	0	100	0	0	0	0
100	100	0	99.36	0	0	100	0	0	0	0
100	100	0	99.36	0	0	100	0	0	0	0
100	100	0	99.36	0	0	100	0	0	0	0
100	100	0	99.36	0	0	100	0	0	0	0
100	100	0	99.36	0	0	100	0	0	0	0
100	100	0	99.36	0	0	100	0	0	0	0
100	100	0	99.36	0	0	100	0	0	0	0
100	100	0	99.36	0	0	100	0	0	0	0
100	100	0	99.36	0	0	100	0	0	0	0
100	100	0	99.36	0	0	100	0	0	0	0
100	100	0	99.36	0	0	100	0	0	0	0
100	100	0	99.36	0	0	100	0	0	0	0
100	100	0	99.36	0	0	100	0	0	0	0
100	100	0	99.36	0	0	100	0	0	0	0
100	100	0	99.36	0	0	100	0	0	0	0
100	100	0	99.36	0	0	100	0	0	0	0
100	100	0	99.36	0	0	100	0	0	0	0
100	100	0	99.36	0	0	100	0	0	0	0
100	100	0	99.36	0	0	100	0	0	0	0
100	100	0	99.36	0	0	100	0	0	0	0
100	100	0	99.36	0	0	100	0	0	0	0
100	100	0	99.36	0	0	100	0	0	0	0
100	100	0	99.36	0	0	100	0	0	0	0
100	100	0	99.36	0	0	100	0	0	0	0
100	100	0	0	0	0	100	0	0	0	0
100	100	0	99.36	0	0	0	0	0	0	0
100	100	0	99.36	0	0	100	0	0	0	0
100	100	0	99.36	0	0	100	0	0	0	0
100	100	0	99.36	0	0	0	0	0	0	0
100	100	0	99.36	0	0	0	0	0	0	0
0	0	0	99.36	0	0	100	0	0	0	0
100	100	0	99.36	0	0	100	0	0	0	0
100	100	0	99.36	0	0	100	0	0	0	0
100	100	0	99.36	0	0	100	0	0	0	0
100	100	0	99.36	0	0	100	0	0	0	0
100	0	0	99.36	0	0	100	0	0	0	0
100	100	0	99.36	0	0	100	0	0	0	0
100	100	0	99.36	0	0	100	0	0	0	0
0	0	0	99.36	0	0	100	0	0	0	0
100	100	0	99.36	0	0	100	0	0	0	0
100	100	0	99.36	0	0	100	0	0	0	0
100	100	0	99.36	0	0	100	0	0	0	0
100	100	0	99.36	0	0	100	0	0	0	0
100	100	0	99.36	0	0	100	0	0	0	0

100	100	0	99.36	0	0	100	0	0	0	0
100	100	0	99.36	0	0	100	0	0	0	0
0	0	0	99.36	0	0	100	0	0	0	0
100	100	0	99.36	0	0	100	0	0	0	0
100	100	0	99.36	0	0	100	0	0	0	0
100	100	0	99.36	0	0	100	0	0	0	0
100	100	0	99.36	100	0	0	0	0	0	0
100	100	0	99.36	100	0	0	0	0	0	0
100	100	0	99.36	0	0	0	0	0	0	0
100	100	0	99.36	0	0	100	0	0	0	0
100	100	0	99.36	0	0	100	0	0	0	0
100	100	0	99.36	0	0	100	0	0	0	0
100	100	0	99.36	0	0	100	0	0	0	0
100	100	0	99.36	0	0	100	0	0	0	0
100	100	0	99.36	0	0	100	0	0	0	0
100	100	0	99.36	0	0	100	0	0	0	0
100	100	0	99.36	0	0	100	0	0	0	0
100	100	0	99.36	0	0	100	0	0	0	0
100	100	0	99.36	0	0	100	0	0	0	0
100	100	0	99.36	0	0	100	0	0	0	0
100	100	0	99.36	0	0	100	0	0	0	0
100	100	0	99.36	0	0	100	0	0	0	0
100	100	0	99.36	0	0	100	0	0	0	0
100	100	0	99.36	0	0	100	0	0	0	0
100	100	0	99.36	0	0	100	0	0	0	0
100	100	0	99.36	0	0	100	0	0	0	0
100	100	0	99.36	0	0	100	0	0	0	0
100	100	0	99.36	0	0	100	0	0	0	0
100	100	0	99.36	0	0	100	0	0	0	0
100	100	0	99.36	0	0	100	0	0	0	0
100	100	0	99.36	0	0	100	0	0	0	0
100	100	0	99.36	0	0	100	0	0	0	0
100	100	0	99.36	0	0	100	0	0	0	0
100	100	0	99.36	0	0	100	0	0	0	0
100	100	0	99.36	0	0	100	0	0	0	0
100	100	0	99.36	0	0	100	0	0	0	0
100	100	0	99.36	0	0	100	0	0	0	0
100	100	0	99.36	0	0	100	0	0	0	0
100	100	0	99.36	0	0	100	0	0	0	0
100	100	0	99.36	0	0	100	0	0	0	0
0	0	0	99.36	0	0	100	0	0	0	0
100	100	0	99.36	0	0	100	0	0	0	0
100	100	0	99.36	0	0	100	0	0	0	0
100	100	0	99.36	0	0	100	0	0	0	0
100	100	0	99.36	0	0	100	0	0	0	0
100	100	0	99.36	0	0	100	0	0	0	0
100	100	0	99.36	0	0	100	0	0	0	0
100	100	0	99.36	0	0	100	0	0	0	0
100	100	0	99.36	0	0	100	0	0	0	0
100	100	0	99.36	0	0	100	0	0	0	0
100	100	0	99.36	0	0	100	0	0	0	0
100	100	0	99.36	0	0	100	0	0	0	0
100	100	0	99.36	0	0	100	0	0	0	0
100	100	0	99.36	0	0	100	0	0	0	0

100	100	0	99.36	0	0	100	0	0	0	0
100	100	0	99.36	0	0	100	0	0	0	0
100	100	0	99.36	0	0	100	0	0	0	0
100	100	0	99.36	100	0	0	0	0	0	0
100	100	0	99.36	100	0	0	0	0	0	0
100	100	0	99.36	0	0	0	0	0	0	0
100	100	0	99.36	0	0	0	0	0	0	0
100	100	0	99.36	0	0	0	0	0	0	0
100	100	0	99.36	0	0	0	0	0	0	0
100	100	0	99.36	0	0	0	0	0	0	0
100	100	0	99.36	0	0	0	0	0	0	0
100	100	0	99.36	0	0	0	0	0	0	0
100	100	0	99.36	0	0	0	0	0	0	0
100	100	0	99.36	0	0	0	0	0	0	0
100	100	0	99.36	0	0	0	0	0	0	0
100	100	0	99.36	0	0	0	0	0	0	0
100	100	0	99.36	0	0	0	0	0	0	0
100	100	0	99.36	0	0	0	0	0	0	0
100	100	0	99.36	0	0	0	0	0	0	0
100	100	0	99.36	0	0	0	0	0	0	0
100	100	0	99.36	0	0	0	0	0	0	0
100	100	0	0	100	0	0	0	0	0	0
100	100	0	99.36	0	0	0	0	0	0	0
100	100	0	99.36	0	0	0	0	0	0	0
100	100	0	99.36	0	0	0	0	0	0	0
100	100	0	99.36	0	0	0	0	0	0	0
100	100	0	99.36	0	0	0	0	0	0	0
100	100	0	99.36	0	0	0	0	0	0	0
100	100	0	99.36	0	0	0	0	0	0	0
100	100	0	99.36	100	0	0	0	0	0	0
100	100	0	99.36	100	0	0	0	0	0	0
100	100	0	99.36	100	0	0	0	0	0	0
100	100	0	99.36	0	0	0	0	0	0	0
100	100	0	99.36	0	0	0	0	0	0	0
100	100	0	99.36	100	0	0	0	0	0	0
0	0	0	99.36	0	0	0	0	0	0	0
0	0	0	99.36	0	0	0	0	0	0	0
100	100	0	99.36	0	0	0	0	0	0	0
100	100	0	99.36	100	0	0	0	0	0	0
100	100	0	99.36	0	0	100	0	0	0	0
100	100	0	99.36	100	0	0	0	0	0	0
100	100	0	99.36	0	0	0	0	0	0	0
100	100	0	99.36	0	0	0	0	0	0	0
100	100	0	99.36	0	0	0	0	0	0	0
100	100	0	99.36	0	0	0	0	0	0	0
100	100	0	99.36	0	0	0	0	0	0	0
100	0	0	99.36	0	0	0	0	0	0	0
100	100	0	99.36	100	0	0	0	0	0	0
0	0	0	99.36	0	0	0	0	0	0	0
100	100	0	99	100	0	0	0	0	0	0
100	0	0	99.36	0	0	0	0	0	0	0
100	100	0	99.36	0	0	0	0	0	0	0
0	0	0	99.36	0	0	0	0	0	0	0
100	0	100	99.36	0	0	0	0	0	0	0

100	100	0	99.36	0	0	0	0	0	0	0
100	0	100	99.36	0	0	0	0	0	0	0
100	0	100	99.36	0	0	0	0	0	0	0
100	100	0	99.36	0	0	0	0	0	0	0
0	0	0	99.36	0	0	0	0	0	0	0
100	100	0	99.36	0	0	100	0	0	0	0
100	100	100	99.36	0	0	100	0	0	0	0
100	100	100	99.36	0	0	0	0	0	0	0
100	100	100	99.36	0	0	0	0	0	0	0
100	100	100	99.36	0	0	0	0	0	0	0
100	100	0	99.36	0	0	0	0	0	0	0
100	0	100	99.36	0	0	100	0	0	0	0
0	0	0	99.36	0	0	0	0	0	0	0
100	100	100	99.36	0	0	100	0	0	0	0
100	0	100	99.36	0	0	0	0	0	0	0
100	100	100	99.36	0	0	0	0	0	0	0
100	100	0	99.36	0	0	0	0	0	0	0
100	0	100	99.36	0	0	100	0	0	0	0
100	100	0	99.36	0	0	0	0	0	0	0
100	100	0	99.36	0	0	100	0	0	0	0
100	0	100	99.36	0	0	100	0	0	0	0
0	0	0	99.36	0	0	0	0	0	0	0
100	100	0	99.36	0	0	0	0	0	0	0
100	100	0	99.36	0	0	100	0	0	0	0
0	0	0	99.36	0	0	0	0	0	0	0
100	100	0	99.36	0	0	100	0	0	0	0
100	100	0	99.36	0	0	100	0	0	0	0
100	100	0	99.36	0	0	0	0	0	0	0
100	0	100	99.36	0	0	0	0	0	0	0
100	100	100	99.36	0	0	0	0	0	0	0
100	100	100	99.36	0	0	0	0	0	0	0
100	0	100	99.36	0	0	0	0	0	0	0
100	0	100	99.36	0	0	0	0	0	0	0
100	100	0	99.36	0	0	0	0	0	0	0
100	100	100	99.36	0	100	0	0	0	0	0
100	100	0	98.73	0	0	0	0	0	0	0
0	0	0	99.36	0	0	0	0	0	0	0
100	100	0	99.36	0	0	0	0	0	0	0
100	100	0	99.36	0	0	0	0	0	0	0
100	100	0	99.36	0	0	0	0	0	0	0
100	100	0	99.36	0	0	0	100	0	0	0
100	100	0	99.36	0	0	0	100	0	0	0
0	0	0	99.36	0	0	0	100	0	0	0
100	100	0	99.36	0	0	0	100	0	0	0
100	100	0	99.36	0	0	0	0	0	0	0
100	100	0	99.36	0	0	0	100	0	0	0
100	0	0	99.36	0	0	0	0	0	0	0
100	100	0	99.36	0	0	0	100	0	0	0
100	100	0	99.36	0	0	0	100	0	0	0
0	0	0	99.36	0	0	0	100	0	0	0
100	100	0	99.36	0	0	0	0	0	0	0
100	100	0	99.36	0	0	0	0	0	0	0
100	100	0	99.36	0	0	100	0	0	0	0

100	100	0	99.36	0	0	0	100	0	0	0
100	100	0	99.36	100	0	0	0	0	0	0
0	0	0	0	0	0	0	0	0	0	0
100	100	0	99.36	0	0	0	0	0	0	0
100	0	100	99.36	0	0	0	0	0	0	0
100	0	100	99.36	0	0	0	0	0	0	0
0	0	0	99.36	0	0	0	0	0	0	0
100	100	0	99.36	0	0	0	0	0	0	0
100	100	0	99.36	0	0	0	0	0	0	0
0	0	0	0	0	0	0	0	0	0	0
0	0	0	0	0	0	0	0	0	0	0
100	100	0	99.36	0	0	0	0	0	0	0
100	100	0	99.36	0	0	0	0	0	0	0
0	0	0	0	0	0	0	0	0	0	0
100	0	100	99.36	0	0	0	0	0	0	0
0	0	0	99.36	0	0	0	0	0	0	0
100	100	0	99.36	0	0	0	0	0	0	0
0	0	0	0	0	0	0	0	0	0	0
100	0	100	0	0	0	0	0	0	100	0
100	100	0	99.36	0	0	0	0	0	0	0
100	0	0	99.36	0	0	0	0	0	0	0
0	0	0	0	0	0	0	100	0	0	0
0	0	0	0	0	0	0	0	0	0	100
0	0	100	0	0	0	0	0	0	0	0
0	0	0	0	0	0	0	0	0	0	0
0	0	0	0	0	0	0	0	0	0	0
0	0	0	0	0	0	0	0	0	0	0
100	0	100	0	0	0	0	0	0	0	0
100	0	0	0	0	0	0	0	0	0	0
0	0	0	0	0	0	0	0	0	0	0
0	0	0	0	0	0	0	0	0	0	0
0	0	0	0	0	0	0	0	0	0	0
0	0	0	99.36	0	0	0	0	0	0	0
0	0	0	0	0	0	0	0	0	0	0
0	0	0	0	0	0	0	0	0	0	0
0	0	0	0	0	0	0	0	0	0	0
0	0	0	0	0	0	0	0	0	0	0
0	0	0	0	0	0	0	0	0	0	0
100	0	0	0	0	0	0	0	0	0	100
0	0	0	0	0	0	0	0	0	0	0
0	0	0	0	0	0	0	0	0	0	0
0	0	0	0	0	0	0	0	0	0	0
0	0	0	0	0	0	0	0	0	0	0
0	0	0	0	0	0	0	0	0	0	0
0	0	0	0	0	0	0	0	0	0	100
100	100	0	99.36	0	0	0	0	0	0	0
100	100	0	99.36	0	0	0	0	0	0	0
100	100	0	99.36	0	0	0	0	0	0	0
100	100	0	99.36	0	0	0	0	0	0	0
0	0	0	0	0	0	0	0	0	0	0
0	100	0	0	0	0	0	0	0	0	0
0	0	0	99.36	0	0	0	0	0	0	0
0	0	0	99.36	0	0	0	0	0	0	0
0	0	0	99.36	0	0	0	0	0	0	0

100	100	0	99.36	0	0	0	0	0	0	0
100	100	0	99.36	0	0	0	0	0	0	0
100	100	0	99.36	0	0	0	0	0	0	0
100	100	0	99.36	0	0	0	0	0	0	0
0	0	0	99.36	0	0	0	0	0	0	0
0	0	0	99.36	0	0	0	0	0	0	0
0	0	0	99.36	0	0	0	0	0	0	0
100	100	0	99.36	0	0	0	0	0	0	0
0	0	0	99.36	0	0	0	0	0	0	0
100	100	0	99.36	0	99.34	0	0	0	0	0
100	0	0	99.36	0	0	0	0	0	0	0
100	100	0	99.36	0	0	0	0	0	0	0
100	100	0	99.36	0	0	0	0	0	0	0
100	100	0	99.36	0	0	0	0	0	0	0
0	0	100	0	0	0	0	0	0	0	0
0	0	0	0	0	0	0	0	0	0	100
0	0	0	0	0	0	0	0	0	0	100
0	0	100	0	0	0	0	0	0	0	0
0	0	100	0	0	0	0	0	0	0	0
0	0	99.52	0	0	0	0	0	0	0	100
0	0	0	99.36	0	0	0	0	0	0	0
0	0	0	99.36	0	0	0	0	0	0	0
0	0	100	0	0	0	0	0	0	0	0
100	100	0	99.36	0	0	0	0	0	0	0
100	100	0	99.36	0	0	0	0	0	0	0
0	0	0	99.36	100	0	0	0	0	0	0
100	100	0	99.36	0	0	0	0	0	0	0
100	0	0	99.36	0	0	0	100	0	0	0
0	0	100	0	0	0	0	0	0	0	100
100	100	0	99.36	100	0	0	0	0	0	0
100	100	0	99.36	0	0	0	0	0	0	0
100	100	0	99.36	100	0	0	0	0	0	0
0	0	0	99.36	0	0	0	0	0	0	0
0	0	0	99.36	0	0	0	0	0	0	0
0	0	0	99.36	0	0	0	0	0	0	0
0	0	0	99.36	0	0	0	0	0	0	0
99.63	100	0	99.36	0	0	0	0	0	0	0
100	100	0	99.36	0	0	0	0	0	0	0
0	0	0	0	0	0	0	0	0	0	0
100	0	0	0	0	100	0	0	0	0	0
0	0	0	0	0	0	0	0	0	0	0
0	0	0	0	0	0	0	0	0	0	0
0	0	100	0	0	0	0	0	0	0	0
100	0	100	99.36	0	0	0	0	0	0	0
0	0	100	0	0	0	0	0	0	0	0
100	100	0	99.36	0	0	0	0	0	0	0

Table S9. Details of assembly statistics of the strains

Assembly strains Id	11sh13D152	18	1943	1977	20062H025	20110812H	20112H151	20122H050	2020SXFB0(	2020SXFB0(	2020SXFB0(	2020SXFB0(	2020SXFB0(	2020SXFB0(	2020SXFB01	2020SXFB01	2020SXFB01	2020SXFB01	2020SXFB01	2020SXFB01
# contigs (>= 0 bp)	569	624	882	604	875	800	801	960	614	624	629	619	605	627	616	611	602	606	607	617
# contigs (>= 1000 bp)	316	325	363	318	341	329	325	355	323	323	323	321	323	321	322	322	323	321	323	322
# contigs (>= 5000 bp)	198	206	211	203	204	202	205	209	206	203	203	203	203	203	203	204	203	203	205	204
# contigs (>= 10000 bp)	140	148	147	143	143	144	144	145	144	143	144	143	145	144	143	144	145	143	145	145
# contigs (>= 25000 bp)	59	63	54	57	56	52	56	57	58	58	58	58	58	58	58	58	58	58	57	58
# contigs (>= 50000 bp)	16	15	11	15	13	13	13	14	14	15	14	15	14	14	15	14	14	15	14	14
Total length (>= 0 bp)	4630084	4817633	4707706	4625201	4658402	4551159	4593463	4812543	4669852	4676538	4677769	4675405	4673807	4676883	4669477	4674400	4673319	4674007	4674447	4675662
Total length (>= 1000 bp)	4544989	4721886	4599068	4534052	4547827	4451817	4495737	4691413	4575980	4579945	4579269	4580428	4580372	4578515	4574705	4578767	4578527	4579390	4581014	4579410
Total length (>= 5000 bp)	4218807	4396388	4181068	4222996	4161310	4098160	4158116	4283932	4249138	4244529	4243630	4249729	4243745	4242846	4239335	4248277	4242663	4249024	4249725	4248880
Total length (>= 10000 bp)	3784566	3964336	3695798	3778011	3698055	3648940	3688884	3799226	3785003	3791666	3795990	3797056	3806062	3796082	3790645	3795639	3805397	3796378	3798184	3806103
Total length (>= 25000 bp)	2484530	2604355	2204224	2415745	2316288	2181206	2289449	2413489	2420498	2447118	2432337	2452178	2432034	2431903	2446589	2431899	2431097	2451549	2406407	2431758
Total length (>= 50000 bp)	1025175	945395	704767	991749	837638	848454	823566	937127	921837	981473	922355	985891	922308	922725	981412	922280	922055	985890	922418	922165
# contigs	370	381	418	372	391	379	373	406	379	380	378	378	378	378	381	381	384	379	381	380
Largest contig	88310	88310	79706	101509	79708	84201	79707	107569	95298	91258	95660	95660	95660	95660	91258	95506	95298	95660	95660	95297
Total length	4583049	4761671	4638009	4572182	4582498	4486165	4529938	4727229	4616312	4620582	4618325	4621273	4620229	4619888	4616771	4620939	4622230	4620844	4622161	4620881
GC (%)	50.75	50.9	50.56	50.84	50.52	50.85	50.75	50.39	50.87	50.85	50.85	50.85	50.85	50.85	50.86	50.85	50.85	50.85	50.85	50.85
N50	27048	27081	23365	25873	25211	24338	25230	25257	25720	25872	25720	25872	25720	25755	25872	25755	25755	25873	25720	25755
N75	14086	14086	11864	13983	12002	12157	12162	11931	13222	13222	13222	13222	13222	13222	13222	13222	13222	13222	13222	13222
L50	52	55	59	52	56	55	56	56	54	53	54	53	54	54	53	54	54	53	54	54
L75	111	116	128	113	120	119	119	123	116	115	116	115	116	116	115	116	116	115	116	116
# N's per 100 kbp	0	0	0	0	0	0	0	0	0	0	0	0	0	0	0	0	0	0	0	0

2020SXFB01	2020SXFB02	2020SXFB02	2020SXFB02	2020SXFB02	2020SXFB02	2020SXFB02	2020SXFB02	2020SXFB02	2020SXFB02	2020SXFB02	2020SXFB03	2020SXFB03	2020SXFB03	2020SXFB03	2020SXFB03	2020SXFB04	2020SXFB04	2020SXFB04	2020SXFB04	2020SXFB04
610	612	658	614	599	614	633	622	605	603	621	601	620	624	626	608	611	617	621	615	611
323	323	332	323	322	322	321	321	322	324	324	323	323	321	321	321	323	322	323	323	322
203	203	207	203	205	205	202	203	205	202	203	201	203	204	203	203	203	203	203	205	204
145	143	146	144	144	143	143	143	146	143	144	142	144	144	143	143	144	144	144	144	144
58	58	59	58	58	58	58	58	58	58	58	58	58	58	58	58	58	58	57	58	58
14	14	15	14	14	15	15	15	14	14	14	15	14	14	15	15	14	14	15	15	14
4674410	4675050	4754267	4669585	4673703	4669434	4677651	4675740	4673467	4673717	4676223	4673646	4676051	4676887	4676624	4674287	4667103	4676227	4676156	4674759	4675615
4580570	4580027	4648472	4575864	4581372	4574694	4580772	4579170	4578779	4580373	4580145	4580448	4580132	4580199	4580184	4580096	4572899	4581046	4581198	4580737	4581532
4244873	4242461	4296873	4237106	4252311	4248427	4246607	4248342	4249991	4235214	4239618	4233691	4238363	4245137	4249531	4246530	4235215	4246408	4244551	4249975	4248378
3806139	3789092	3842422	3794578	3795873	3780979	3799843	3795353	3807141	3786634	3792063	3785902	3791087	3792448	3796040	3797544	3791368	3799973	3798825	3795813	3796091
2431694	2432369	2466566	2430773	2432080	2436418	2455269	2450845	2420985	2431902	2427965	2450482	2426983	2428640	2451760	2451116	2431463	2431419	2424672	2446661	2432166
922419	922433	957366	922297	922417	980909	990528	985311	922361	921702	918032	985388	918032	919355	985735	985733	922072	922527	985532	981333	922418
381	380	395	380	379	381	377	380	381	382	381	381	380	378	378	379	378	377	379	381	379
95660	95660	88310	95506	95660	91258	100453	95297	95298	95297	91258	95297	91258	91258	95660	95660	95298	95660	95660	91258	95660
4621590	4620804	4692730	4616609	4622122	4616612	4620744	4621372	4621500	4622270	4620570	4622016	4621471	4621059	4620554	4621445	4612725	4620413	4620888	4622010	4621923
50.85	50.85	50.84	50.87	50.85	50.87	50.85	50.85	50.85	50.85	50.85	50.85	50.86	50.85	50.85	50.85	50.85	50.85	50.85	50.85	50.86
25755	25720	25755	25755	25755	25872	25873	26002	25755	25720	25720	25873	25755	25755	25873	25873	25755	25755	25755	25872	25755
13222	13487	12727	13222	13222	12727	13487	13222	13222	13222	13222	13222	13222	13222	13222	13222	13222	13487	13222	12711	13222
54	54	55	54	54	53	53	53	54	54	54	53	54	54	53	53	54	54	53	53	54
116	115	118	116	116	116	114	115	116	116	116	115	116	116	115	115	115	115	115	116	116
0	0	0	0	0	0	0	0	0	0	0	0	0	0	0	0	0	0	0	0	0

2020SXFB0	2020SXLYX	2020SXWbk	2020SXWSZ	2020SXXXj	21535	21679	230603	230608	41sh10D162	41sh10D181	41sh10D187	41sh11D062	41sh13D174	AH17	BJ20072H0	BJ20092H1	BJ2010074	BJ20102H03	BJ20122H07	ERR024076
615	645	604	629	636	757	783	939	784	852	630	819	669	691	1227	583	570	717	674	672	1617
321	334	324	324	326	321	300	352	323	358	328	373	325	345	856	333	320	364	353	326	435
204	206	206	206	205	199	189	212	201	214	206	223	202	209	287	211	204	210	221	201	256
145	146	144	144	144	143	145	147	142	151	146	150	146	147	106	145	142	142	147	142	152
58	58	57	58	58	53	54	56	52	59	60	61	59	58	4	60	59	56	60	57	33
14	14	15	14	15	12	15	11	12	14	14	14	14	15	0	14	15	15	13	13	1
4675927	4707173	4667998	4676707	4683349	4461322	4553555	4696680	4445219	4888451	4728271	4997170	4698028	4739630	4459343	4762026	4656535	4758045	4841146	4597219	4448870
4582202	4608499	4574223	4578414	4586293	4370758	4452780	4583219	4349208	4751199	4630272	4855091	4598009	4631016	4311622	4669622	4570646	4642247	4729241	4487832	4316297
4252405	4252151	4244358	4248012	4249562	4027603	4159004	4184732	4006302	4354059	4292966	4451288	4261000	4265759	2819194	4336868	4248074	4236445	4371358	4150859	3837095
3809963	3801831	3781000	3781311	3789547	3592208	3828856	3697049	3553048	3883024	3847678	3931662	3841991	3806562	1551890	3838889	3787353	3727250	3830222	3704525	3060074
2432317	2417380	2408122	2414980	2434483	2164749	2338144	2258833	2138938	2423333	2475578	2514054	2454617	2396660	108192	2476025	2484246	2366682	2439720	2343218	1169438
922281	908639	985389	922101	968578	764985	975087	712031	765627	879947	884007	880620	912776	944119	0	898668	1004509	974774	826693	827993	53306
378	394	384	385	383	368	356	402	373	427	386	447	384	409	962	392	367	434	413	383	494
95506	88310	95298	95297	88310	79708	76421	79708	79705	88270	88310	88310	88270	88310	29961	88342	113830	88311	88342	88343	53306
4622628	4651098	4617096	4621056	4626610	4403767	4491572	4618524	4384242	4800569	4670649	4907012	4639960	4676554	4393476	4710361	4603796	4692628	4771700	4527718	4358881
50.86	50.84	50.87	50.85	50.85	50.81	50.87	50.49	50.79	50.57	50.82	50.6	50.87	50.85	51.3	50.87	50.78	50.73	50.6	50.81	50.84
25755	25720	25720	25755	25720	24359	25257	24338	24015	25021	25874	25357	25833	25357	7175	25752	26034	25061	25389	25787	16047
13222	12675	12711	12727	12727	12162	14284	11932	12001	12222	13983	12101	13943	12459	3849	12745	13256	12032	12134	12743	8580
54	55	54	54	54	55	51	59	55	59	55	59	54	56	183	56	52	56	58	54	85
116	119	117	117	116	118	109	127	119	127	117	128	115	121	396	119	113	124	125	116	175
0	0	0	0	0	0	0	0	0	0	0	0	0	0	0	0	0	0	0	0	0

ERR024078	ERR024079	ERR024085	ERR024604	ERR024605	ERR024606	ERR024607	ERR024608	ERR024609	ERR024610	ERR024611	ERR024612	ERR024614	ERR024616	ERR024617	ERR024618	ERR024619	ERR024620	ERR024622	ERR024625	ERR024626
434	1682	2010	426	981	1138	1028	1254	1171	1012	1236	1096	1050	1479	1121	1563	1205	1489	1106	1220	1391
354	490	456	365	320	334	328	354	327	345	382	336	354	370	355	394	367	352	345	372	373
213	265	264	223	203	208	208	202	204	210	220	207	213	220	221	232	230	219	213	223	217
142	163	150	157	141	147	146	143	143	148	142	139	145	146	144	152	162	151	147	142	141
54	35	36	50	55	62	53	52	51	50	44	52	50	50	51	53	43	48	47	51	46
15	3	4	6	13	12	10	9	9	7	7	9	8	10	9	9	9	8	6	9	10
4628677	4690718	4614257	4516360	4539267	4728438	4565650	4503898	4417963	4407576	4410538	4385837	4492646	4634117	4539342	4792276	4684289	4566016	4425552	4540944	4558384
4581525	4514034	4434905	4481357	4439642	4610583	4457722	4382834	4307351	4311392	4296436	4277239	4381848	4482095	4426092	4637602	4545942	4434754	4324980	4414375	4425877
4209996	3927567	3929391	4105948	4121740	4281016	4138319	3974054	3959718	3940068	3884474	3925931	3995412	4072620	4045741	4207172	4180704	4064994	3966356	4017666	4019367
3680557	3187379	3095174	3627562	3657327	3840767	3689361	3532745	3490869	3484234	3308124	3402375	3485033	3523696	3464066	3614330	3685411	3541146	3464811	3403912	3433169
2301353	1262603	1317927	1929375	2260592	2476932	2186062	2076905	2011474	1921397	1737531	2036788	1960835	1982456	2015158	2048584	1782891	1894544	1867095	1959736	1894643
985353	172549	239771	381037	813637	717285	656397	562409	564395	453508	469995	573542	531605	620581	576484	545653	566330	507788	405583	580049	659844
409	585	537	409	368	385	378	407	370	386	431	385	406	432	404	456	446	396	384	435	425
108321	70339	68786	87929	76901	69056	87357	79694	80092	76513	79660	80158	80085	79790	80161	75149	76202	76619	88269	88163	79832
4619383	4580869	4493397	4512889	4474159	4646842	4493974	4422361	4338947	4340476	4330791	4312630	4419341	4527926	4461631	4683813	4603678	4466818	4352909	4459335	4462878
50.74	50.59	50.72	50.83	50.78	50.86	50.77	50.59	50.82	50.82	50.85	50.81	50.81	50.86	50.79	50.55	50.71	50.86	50.84	50.9	50.82
24549	15284	16298	22076	25051	25963	24257	23203	22675	22022	20424	23321	22214	21274	21726	21405	21071	21635	21268	21938	21152
11934	8294	8407	11624	13342	13342	13078	11859	11898	11623	10798	11348	11324	11080	11048	11080	11625	11438	11390	10924	11004
55	90	84	64	55	57	56	58	58	61	64	57	61	63	61	66	66	63	61	63	61
123	191	180	135	115	117	119	124	122	128	137	124	130	135	134	143	141	134	129	137	133
98.37	0	0	0	0	0	0	0	0	0	0	0	0	0	0	0	0	0	0	0	0

ERR024627	ERR025682	ERR025683	ERR025685	ERR025686	ERR025687	ERR025690	ERR025691	ERR025692	ERR025693	ERR025695	ERR025697	ERR025698	ERR025699	ERR025700	ERR025701	ERR025703	ERR025704	ERR025705	ERR025706	ERR025708
1132	1583	555	1345	2019	581	697	1589	1493	1629	582	644	1387	1387	1463	1505	1317	1365	1523	1184	552
346	425	449	383	360	442	554	387	378	390	464	479	380	404	454	395	390	403	408	389	453
213	242	247	215	221	253	285	223	224	231	243	240	217	228	245	223	227	230	229	231	252
144	171	146	144	153	163	148	158	155	157	137	140	146	146	151	149	142	146	149	138	152
47	40	36	50	50	42	33	45	49	49	33	37	51	46	41	44	46	44	44	42	30
8	5	4	9	10	8	1	11	6	9	5	4	10	10	7	6	4	6	6	9	2
4399138	4649953	4486945	4524309	4677274	4719195	4637368	4760500	4561743	4781041	4311343	4360930	4599162	4581191	4646513	4521573	4429416	4563694	4563447	4432879	4375263
4288169	4492339	4438942	4392623	4505951	4652897	4559738	4595216	4424697	4608831	4247257	4304133	4466853	4436909	4511649	4369643	4305658	4418246	4404119	4317407	4321660
3920042	4032149	3922916	3934026	4150630	4156970	3851689	4182057	4018369	4198887	3656067	3669290	4019824	3978287	3964364	3917684	3881502	3963442	3935546	3893705	3785587
3392267	3498730	3185495	3401097	3648462	3502296	2890347	3712962	3536088	3660393	2888340	2939254	3493223	3374222	3264020	3370322	3252662	3339354	3346555	3202692	3065978
1837186	1465474	1420797	1900776	2052486	1648541	1123023	1908029	1836337	1933162	1240521	1340355	2002695	1818795	1574642	1720807	1743408	1715199	1678432	1683227	1122446
500624	297290	264629	556910	620625	505116	51281	708409	351678	550784	281601	228663	633493	627153	433997	387574	240469	372118	372886	543794	131371
395	511	504	440	434	523	646	468	452	475	540	540	435	479	529	467	449	470	486	451	517
79789	75811	87840	76905	70405	87509	51281	79789	64369	76206	65642	59624	79806	79766	76536	77883	70674	82925	76322	79885	70286
4324922	4553743	4477791	4432386	4560001	4710936	4626075	4655339	4477799	4668499	4301413	4348612	4506540	4491382	4565247	4419740	4347911	4466984	4458568	4361650	4367885
50.77	50.79	50.58	50.58	50.77	50.45	50.68	50.42	50.78	50.53	50.98	50.69	50.48	50.58	50.7	50.56	50.89	50.58	50.54	50.83	50.85
21635	17441	18039	21373	21613	17528	13625	21087	21270	21270	16098	15350	22080	20228	17530	19489	20218	20093	20086	19174	15968
11085	10606	8828	10982	11592	9738	6758	11859	11620	11354	7797	7859	10981	10177	8984	10767	9932	9928	10219	9492	8464
62	80	76	64	60	77	100	64	67	67	80	80	62	66	76	67	66	68	70	65	84
131	163	165	137	132	167	220	138	139	143	177	177	136	146	168	144	143	148	149	146	175
0	0	0	0	0	0	0	0	0	0	0	0	0	0	0	0	0	0	0	0	0

ERR025709	ERR025710	ERR025711	ERR025712	ERR025713	ERR025714	ERR025715	ERR025716	ERR025717	ERR025718	ERR025719	ERR025721	ERR025724	ERR025725	ERR025726	ERR025729	ERR025731	ERR025732	ERR025734	ERR025737	ERR025742
1508	1358	1318	1535	1392	1249	525	1428	2096	1340	1085	572	1005	559	1200	1105	1353	1388	1531	1268	1442
421	416	375	368	376	366	409	385	388	371	342	457	347	425	393	445	430	349	370	364	352
228	225	211	211	218	212	235	220	222	214	207	256	212	247	230	247	235	207	219	225	220
148	153	144	140	141	138	156	146	147	142	139	150	147	151	153	147	155	148	152	156	148
45	47	52	50	50	45	44	48	50	51	51	33	49	39	47	35	45	53	45	46	46
5	4	8	12	11	10	4	9	6	11	10	3	11	4	10	2	7	10	9	10	7
4569398	4579437	4562424	4592034	4648948	4401306	4563513	4578190	4597647	4573846	4440516	4411952	4487630	4469377	4671079	4417494	4676843	4583564	4595853	4689014	4441073
4425442	4446817	4430652	4451610	4508784	4280720	4524439	4434875	4433804	4443263	4334446	4355151	4384354	4416264	4539868	4306279	4542933	4452911	4443517	4570681	4318519
3930111	3947616	4000193	4033504	4078414	3874594	4059366	4002318	4014256	4009061	3960433	3860550	4027837	3952500	4135070	3801388	4043925	4072720	4054599	4216065	3984460
3329113	3411997	3493714	3506201	3495897	3321098	3480537	3464877	3460698	3460848	3442214	3090948	3564931	3240119	3581277	3080551	3478192	3634315	3555495	3704315	3443518
1692104	1728396	2007619	2060878	2051716	1851343	1683122	1907410	1936690	2033688	2056666	1245257	1998672	1450146	1885565	1268989	1757841	2129039	1857257	1871715	1813964
344540	238240	530571	780068	696698	632075	288986	563268	399324	672651	635657	187238	687247	261492	615950	132535	434158	637802	612072	652694	443922
489	475	425	421	441	427	451	447	446	424	387	525	404	478	471	513	498	402	452	424	404
79931	64365	80015	88166	88166	79756	87849	76619	88167	79790	88165	77813	80133	87930	88166	76293	71647	79893	81704	80792	72535
4471633	4487872	4467093	4490456	4555403	4324572	4555267	4479899	4473214	4481514	4367358	4403653	4426367	4454256	4594973	4355295	4592091	4490675	4504133	4614440	4356052
50.56	50.55	50.6	50.59	50.43	50.82	50.85	50.56	50.59	50.53	50.83	50.84	50.83	50.59	50.87	50.96	50.93	50.77	50.78	50.85	50.87
18926	20051	22315	22145	21940	21272	19341	21089	21159	22214	23203	15685	21928	17658	21091	16569	19451	23326	21689	21148	21422
9899	10767	11348	11389	10924	10867	10535	11004	10879	10982	11393	8716	11858	9460	11611	8906	10388	11892	11327	12157	11510
70	71	62	58	60	59	72	63	64	60	57	81	59	77	65	80	70	58	62	65	62
151	149	131	128	134	131	150	137	138	133	124	173	126	162	141	167	152	125	136	135	132
0	0	0	0	0	0	0	0	0	0	0	0	0	0	0	0	0	0	0	0	0

ERR025743	ERR025746	ERR025747	ERR025748	ERR025749	ERR025750	ERR025751	ERR025753	ERR025754	ERR025755	ERR025756	ERR025758	ERR025761	ERR025762	ERR025765	ERR025767	ERR025768	ERR028672	ERR028675	ERR028676	ERR028679
853	1115	1140	601	1096	1780	534	1145	2097	1243	1389	1177	1403	1618	2014	1415	1535	1073	1536	1368	1267
309	306	363	438	321	364	401	317	357	359	364	355	344	359	358	378	372	332	340	331	356
193	198	220	229	198	216	238	200	230	211	215	219	213	218	219	223	227	198	203	206	220
137	148	140	133	131	139	148	143	152	139	143	141	138	145	144	147	144	149	143	139	146
50	55	44	40	48	44	39	50	50	54	48	49	47	52	55	52	42	50	56	53	50
14	12	8	5	10	8	7	12	11	8	8	10	12	12	11	10	6	15	15	12	7
4432003	4472888	4282700	4293039	4218449	4378258	4463826	4443132	4754798	4649878	4547324	4488922	4451703	4714093	4773575	4674176	4406773	4711606	4686214	4552814	4524619
4334461	4364496	4170760	4247157	4113848	4226893	4414853	4334473	4576542	4523900	4418530	4375663	4322469	4556615	4591808	4546193	4278647	4585013	4530790	4419529	4388575
4036575	4084395	3800555	3722633	3786656	3854602	3974939	4022899	4260759	4138958	4039279	4009724	3966788	4204390	4225497	4141030	3879978	4216419	4183088	4075080	4031272
3630878	3718069	3203101	3018039	3282450	3286398	3320651	3597480	3719770	3606531	3510149	3422510	3412560	3670420	3686665	3573661	3249613	3854567	3736994	3568160	3484452
2188297	2236208	1671793	1544991	1952077	1785101	1584384	2082863	2013640	2174747	1929158	1973590	1952249	2191929	2222908	2038043	1649604	2207818	2332348	2172767	1945510
901233	758838	494450	304999	645462	504984	447200	788473	678886	536249	510149	634180	755913	781252	657908	630566	390200	979396	929597	752172	464666
372	362	416	488	371	420	457	371	422	422	420	409	393	426	428	428	421	390	398	376	411
88164	80126	76437	76302	87942	88132	87838	88164	72720	84058	84014	79869	88167	79988	70676	80362	79831	103646	76253	79696	88197
4380263	4404313	4211128	4282034	4150200	4266469	4456549	4374210	4624031	4569657	4459358	4414125	4358263	4605762	4644407	4583103	4315004	4627170	4573302	4451482	4427126
50.91	50.81	50.89	50.66	50.85	50.85	50.84	50.88	50.97	50.8	50.85	50.84	50.81	50.52	50.97	50.76	50.81	50.79	50.89	50.7	50.87
24867	25116	19755	18297	23237	20464	18594	23622	23306	24032	23387	22231	22214	23622	23889	22003	18880	23872	25218	23963	21758
13935	13650	10839	8470	11860	11080	9795	13596	12159	12418	11426	11080	11398	11812	12046	11438	10389	13818	13264	12150	11470
51	54	64	69	54	60	69	55	63	59	61	59	57	57	60	64	66	55	55	56	62
109	114	136	155	116	131	151	116	130	123	128	131	125	126	126	135	143	117	117	119	131
0	0	0	0	0	0	0	0	0	0	0	0	0	0	0	0	0	0	0	0	0

ERR028685	ERR028688	ERR028689	ERR028690	ERR028691	ERR028692	ERR028694	ERR028695	ERR028699	ERR028703	ERR028704	ERR028705	ERR028706	ERR136417	ERR136419	ERR136419	ERR136419	ERR136420	ERR136420	ERR136421	ERR136422
1472	1561	1639	1609	937	823	1980	1510	1929	1258	1619	1407	981	744	655	687	701	653	580	668	671
362	332	354	358	380	303	359	357	364	357	368	332	354	319	312	317	315	316	319	313	319
213	201	208	208	222	183	213	212	214	219	216	207	199	210	204	201	202	205	204	205	204
145	140	140	144	144	136	142	141	145	151	146	144	139	151	143	142	141	148	141	143	143
56	54	51	55	48	61	53	54	52	52	56	53	55	55	56	54	55	55	56	56	57
10	10	11	12	8	17	12	12	10	8	12	9	15	11	14	15	14	11	15	14	15
4754344	4504819	4631422	4718305	4533667	4628976	4749593	4715335	4729801	4672126	4816691	4528999	4671378	4605031	4645431	4648590	4560029	4589165	4626109	4646505	4754058
4596276	4355941	4468281	4543416	4427710	4525560	4558579	4555696	4536383	4526065	4640678	4395317	4551311	4502583	4557826	4557058	4462409	4502940	4544791	4555163	4664945
4200588	4001669	4077543	4149155	4011760	4215320	4161635	4164702	4122439	4151350	4229611	4067810	4155570	4196688	4243314	4230839	4142940	4202167	4226880	4243459	4336513
3679936	3547996	3560106	3665875	3437130	3864276	3635224	3620576	3608286	3642725	3700846	3588794	3728800	3745685	3775919	3780668	3676783	3761802	3741269	3770730	3866888
2252788	2170454	2105234	2220949	1897902	2621726	2197689	2220486	2123132	2032740	2233979	2120608	2354977	2255273	2389704	2363848	2292582	2260674	2376454	2387773	2497783
657732	655354	717766	744404	519552	1085089	771963	769474	670832	535796	766896	604343	961386	719279	926889	1021506	889371	743162	976953	926207	1036751
415	385	404	428	429	350	415	411	420	415	431	378	414	369	360	362	366	363	377	365	368
88199	81834	88199	88801	81292	85577	88199	88199	88199	88190	88199	88197	88193	88246	89608	92762	88253	88244	89616	88875	109195
4633776	4393895	4504383	4594335	4463100	4559392	4598923	4594810	4577308	4568353	4686703	4427023	4593958	4539413	4593620	4589360	4499206	4536282	4586588	4593172	4701335
50.56	50.84	50.53	50.8	50.71	50.96	50.61	50.51	50.52	50.54	50.5	50.81	50.61	50.85	50.74	50.83	50.82	50.76	50.73	50.73	50.61
23962	24347	23265	24014	20829	28267	23353	23353	22349	22740	23352	23592	25218	24997	25656	25592	25293	24997	25592	25656	26858
11891	12592	11892	11944	11347	15108	11891	11680	11470	11852	11891	12072	13374	12120	13158	13919	12649	13919	13158	12649	13158
59	56	58	59	63	48	58	58	60	63	61	57	53	56	53	52	54	56	53	53	52
127	119	124	125	136	102	126	126	129	132	130	121	115	121	115	113	115	117	115	115	114
0	0	0	0	0	0	0	0	0	0	0	0	0	0	0	0	0	0	0	0	0

ERR136423[illegible]	ERR136424[illegible]	ERR136425[illegible]	ERR136425[illegible]	ERR136426[illegible]	ERR136427[illegible]	ERR136427[illegible]	ERR136429[illegible]	ERR136432[illegible]	ERR136432[illegible]	ERR151607[illegible]	ERR154157[illegible]	ERR154158[illegible]	ERR154159[illegible]	GIMI239[illegible]	HN08149	HN08160	HN08184	HN09187	HN09221	HN09238
656	656	691	649	645	662	703	651	786	736	666	589	878	653	865	744	747	565	606	717	607
314	314	318	315	309	314	319	315	353	353	339	330	372	355	333	364	369	323	333	360	334
201	205	201	206	202	204	206	202	214	210	208	204	224	216	208	215	215	202	205	213	205
144	144	144	144	144	144	145	143	145	146	140	141	147	145	151	144	145	141	139	142	139
55	56	54	57	56	57	58	55	56	55	55	55	59	58	59	59	61	58	56	60	56
13	14	13	16	14	15	16	13	13	12	13	11	13	14	14	15	15	15	15	14	15
4574704	4671126	4616116	4751851	4631098	4755655	4760129	4596374	4750098	4729022	4586720	4538374	4942360	4742950	4839149	4825783	4869570	4661119	4598936	4784636	4600747
4485999	4584364	4520843	4666667	4546676	4669879	4668598	4510694	4638385	4627359	4487767	4459498	4814844	4644756	4731400	4702150	4747084	4569681	4507184	4665355	4509936
4165947	4272030	4196424	4355317	4241636	4352458	4341555	4190753	4257150	4237567	4139920	4124078	4422883	4282524	4374340	4308109	4338558	4236647	4164563	4270595	4167509
3732753	3807865	3747433	3880474	3798356	3895018	3881575	3743053	3742771	3755578	3623681	3652718	3843456	3749441	3936982	3789518	3833641	3776885	3673570	3748076	3677447
2316054	2394953	2298159	2496011	2395643	2505351	2497754	2350612	2322236	2286540	2270721	2299139	2417935	2386973	2480225	2445696	2518433	2446308	2362407	2450625	2367847
848511	926444	868283	1084769	926976	1035735	1052364	889299	824581	774996	830201	760289	823927	885747	934693	944761	951651	978780	978291	870231	977718
361	361	367	362	356	361	366	360	412	410	404	387	440	427	387	430	434	382	388	424	391
88607	88931	92104	109195	89616	108730	108730	119269	87499	87499	88255	93822	88244	88247	103226	88311	88311	88342	88311	88311	88311
4519793	4618781	4556731	4701152	4581328	4703946	4702698	4542909	4681858	4668962	4534729	4499678	4863943	4698033	4767912	4748302	4792787	4610963	4545892	4711159	4548752
50.77	50.7	50.86	50.61	50.68	50.61	50.62	50.81	50.45	50.45	50.79	50.8	50.51	50.79	50.75	50.59	50.49	50.87	50.77	50.57	50.78
25592	25656	25266	26858	25938	26858	26858	25656	24997	24912	25266	25293	24997	25293	25902	25357	25755	25905	25720	25545	25720
13158	13422	13422	13422	13422	13955	13422	13158	11900	12037	11968	12197	11703	11968	13122	12102	12032	13254	12262	12101	12261
53	53	54	52	52	52	53	52	57	57	55	54	60	57	56	57	57	53	53	57	53
115	115	116	114	113	113	115	114	125	124	120	117	130	125	120	124	124	114	116	123	116
0	0	0	0	0	0	0	0	0	0	0	0	0	0	0	0	0	0	0	0	0

HN10108	HN10159	HN10208	HN11128	HN12099	HN12187	HN12202	HN12237	HN13068	HN13118	HN13136	HN1	HN6	lj06041	lj06057	lj06058	lj07024	lj07026	lj10020	lj11005	lj11023
639	668	599	799	747	648	769	639	854	735	736	626	623	542	553	707	552	544	655	610	563
334	351	331	380	366	339	368	341	359	368	364	329	332	321	322	415	321	339	361	373	328
201	205	203	218	211	208	214	204	202	215	212	205	205	203	204	214	205	207	217	213	211
140	141	143	146	145	144	144	141	136	143	143	141	141	141	142	142	140	141	145	148	141
57	58	60	63	60	60	61	58	57	59	58	55	55	57	57	57	57	60	61	57	58
15	16	16	13	16	14	15	16	13	14	15	14	15	14	14	14	14	15	15	13	14
4616761	4806653	4755003	4981301	4855185	4733533	4889761	4679331	4664571	4808147	4770345	4525577	4613073	4527155	4601556	4896006	4598285	4696665	4917643	4786950	4657997
4515509	4697300	4656321	4840745	4730847	4632687	4762273	4578205	4492011	4690696	4650516	4428872	4518687	4448627	4514418	4782982	4514531	4615588	4810644	4694549	4568090
4160665	4315411	4304229	4409277	4320827	4281107	4351041	4215086	4081273	4274730	4242259	4097318	4184160	4128847	4192920	4257387	4196984	4260613	4433405	4277133	4242263
3709386	3843817	3850834	3880248	3835642	3809035	3831037	3750583	3593846	3743733	3732363	3626283	3709487	3662969	3724610	3718932	3708290	3757252	3896656	3797374	3718170
2398362	2535741	2537119	2556474	2494337	2480453	2509259	2445747	2336573	2424444	2385558	2267881	2334661	2344992	2367072	2371802	2385331	2481088	2550172	2366651	2411005
974703	1111288	1040617	827055	1005998	890465	944486	1020822	840078	884977	944631	876812	977941	893299	896006	899465	923117	962089	987433	827180	902445
392	416	389	455	434	397	436	401	449	427	426	388	385	367	378	491	370	394	429	435	386
88311	113906	88340	88311	88311	88311	88311	88311	88311	88311	88311	88311	88311	88343	88406	88406	94728	88342	101470	88342	88342
4557093	4743000	4698510	4894938	4778520	4673823	4810200	4620983	4556476	4732131	4695301	4470834	4556097	4481501	4554484	4838694	4550488	4656404	4857518	4739106	4609931
50.83	50.71	50.88	50.52	50.42	50.84	50.58	50.88	50.88	50.59	50.56	50.79	50.77	50.82	50.83	50.49	50.85	50.84	50.58	50.77	50.77
25755	26003	26034	25720	25755	25873	25755	26003	25357	25357	25330	25330	25357	25752	25752	23809	25752	25905	25752	24491	25752
12711	12676	13519	12032	12261	12711	12102	12459	11767	11654	12031	12459	12676	12707	12745	11100	12708	12290	12290	11799	12135
53	52	53	59	56	55	57	53	55	57	57	54	53	53	54	59	53	54	57	58	54
115	116	115	128	123	118	125	116	120	126	125	117	116	115	116	134	115	118	123	126	118
0	0	0	0	0	0	0	0	0	0	0	0	0	0	0	0	0	0	0	0	0

lj11031	lj11042	lj12024	lj13015	lj13016	lj14023	lj16007	lj16011	lj16015	R01	R02	R03	R04	R05	R06	R07	R08	R09	R100	R101	R102
576	559	651	520	559	566	1145	521	539	823	675	822	797	652	811	695	672	706	675	808	675
326	326	395	314	332	365	540	321	319	347	323	350	354	319	340	328	318	327	327	348	319
202	204	216	203	206	212	285	204	205	208	199	212	215	203	207	201	201	204	202	208	202
140	139	144	140	145	141	155	140	142	143	143	145	148	142	151	143	141	145	143	146	144
58	58	58	59	59	59	30	59	57	56	54	56	57	53	59	54	57	55	55	57	55
14	14	14	15	15	15	2	14	14	14	14	14	12	12	12	13	15	13	13	13	13
4656326	4651740	4836529	4629721	4744259	4764621	4799652	4652182	4627222	4708438	4584815	4786943	4788730	4488650	4850730	4579726	4620650	4589363	4580830	4760869	4602664
4564141	4564393	4737592	4553971	4660614	4684296	4602809	4576182	4549643	4588003	4494926	4669786	4677284	4402450	4735022	4486003	4531427	4497764	4492555	4645582	4512549
4218961	4227444	4276292	4242267	4322760	4264859	3922987	4253471	4231621	4198497	4147103	4280459	4283012	4071692	4358426	4125315	4194381	4147500	4148424	4252664	4172070
3746784	3730579	3752930	3756543	3861639	3731173	2968093	3762091	3747942	3708193	3718786	3779884	3780375	3606205	3936488	3684481	3741286	3701168	3695675	3785586	3729949
2439768	2438400	2397274	2467255	2490685	2445175	1018347	2479985	2403767	2324356	2296950	2370183	2333820	2178884	2441115	2277708	2416175	2271604	2281981	2364382	2308288
942296	942549	883678	973586	977092	948650	115590	926752	915178	878249	913602	912105	759401	766110	771676	850194	978364	822357	836307	820702	847943
383	381	463	360	384	419	620	371	364	408	372	406	411	367	400	378	366	373	374	406	368
113167	113830	88342	88342	88342	88342	65220	98780	88341	79744	84237	82132	79744	79744	79768	84229	88246	79744	84262	79744	94436
4604578	4604179	4786642	4586922	4698020	4724708	4662634	4611923	4582147	4631642	4530625	4711293	4718869	4436902	4776986	4522561	4566330	4531722	4527248	4688127	4547524
50.78	50.78	50.7	50.86	50.92	50.57	50.86	50.9	50.86	50.5	50.8	50.57	50.58	50.79	50.75	50.82	50.84	50.84	50.84	50.52	50.84
26035	25905	25093	26034	25905	25689	14100	25905	25905	25266	25293	25266	24912	24575	25592	25266	25938	25266	25266	25266	25293
12567	12491	11614	13254	13519	11697	7372	12708	12708	11968	12647	12038	11968	12193	13421	12193	12612	12395	12612	12037	12649
53	53	58	53	54	56	101	53	53	56	53	56	59	55	57	54	52	55	55	57	54
115	115	129	113	116	124	216	114	115	122	115	123	127	118	121	118	114	119	117	123	117
0	0	0	0	0	0	0	0	0	0	0	0	0	0	0	0	0	0	0	0	0

R103	R104	R105	R106	R107	R108	R109	R10	R110	R111	R112	R113	R114	R115	R116	R117	R118	R119	R11	R120	R121
699	729	797	685	692	701	883	724	866	794	659	795	674	764	685	712	675	686	733	662	674
318	297	348	323	324	323	352	323	355	343	312	342	317	341	321	326	316	327	348	328	329
201	196	215	205	206	204	217	204	215	208	200	208	202	205	200	202	198	204	211	202	204
143	144	147	145	144	146	147	143	149	143	143	142	144	143	144	145	144	145	146	145	145
55	59	54	56	57	56	56	55	55	54	53	53	54	54	55	55	55	55	56	56	55
15	17	14	15	13	15	14	15	14	13	12	13	13	14	14	14	13	13	13	12	13
4630553	4801077	4732235	4690003	4614317	4706316	4912799	4616239	4811295	4644420	4470768	4652001	4563686	4715394	4621633	4629039	4578824	4626791	4718776	4594702	4594698
4536321	4693863	4620213	4595231	4520557	4612297	4780676	4516238	4684059	4531415	4379298	4539832	4471767	4607472	4525807	4530956	4488141	4535985	4622216	4509181	4506286
4199591	4433703	4244752	4257386	4181747	4268637	4404490	4170706	4294915	4145031	4066367	4157539	4140224	4220756	4172411	4178140	4160009	4190821	4228717	4149967	4156053
3756523	4049734	3735093	3799687	3708565	3825880	3876525	3702410	3796692	3656336	3628190	3658889	3690627	3744007	3739733	3736435	3741985	3743205	3739839	3714653	3710467
2352706	2640145	2251252	2390248	2328129	2373664	2407003	2308524	2292025	2238823	2186187	2232723	2242145	2311211	2319456	2298874	2311724	2294894	2309890	2284214	2267665
978326	1159920	881842	970980	826957	958695	953220	967213	881393	829909	766020	841806	832596	933293	913233	891297	871563	845107	815990	758276	822795
369	351	404	377	373	375	413	378	414	397	363	397	366	396	374	377	365	376	397	375	377
98675	89344	79744	86471	79744	89942	107373	87880	79744	79744	79744	88246	79744	86722	84895	79743	107247	84271	79744	79744	79744
4573009	4732669	4660718	4634064	4555492	4649367	4824434	4555360	4726425	4571073	4416367	4579257	4507527	4647349	4564089	4568181	4522940	4571458	4657033	4543161	4540834
50.85	50.92	50.49	50.76	50.76	50.83	50.39	50.76	50.48	50.48	50.79	50.49	50.77	50.66	50.76	50.76	50.7	50.86	50.47	50.83	50.83
25592	27940	24051	25592	25293	25293	24997	25293	24575	24828	24997	24031	24997	24997	25293	25266	25293	25266	24997	25266	24997
12647	14922	12037	12612	12198	13158	12193	12198	11968	11814	12649	11939	12647	12038	12636	12638	13422	12614	11968	12612	12612
53	49	58	54	56	55	57	54	58	56	54	56	55	55	54	55	54	55	57	56	56
115	105	126	117	119	117	124	118	127	123	116	122	117	121	117	118	114	118	124	119	119
0	0	0	0	0	0	0	0	0	0	0	0	0	0	0	0	0	0	0	0	0

R122	R123	R124	R125	R126	R127	R128	R129	R12	R130	R131	R132	R133	R134	R135	R136	R137	R138	R13	R14	R15
693	680	725	688	687	680	688	704	904	639	694	690	673	670	672	700	652	693	707	687	668
324	322	330	321	321	323	331	322	363	315	323	325	320	327	325	321	317	323	323	322	320
207	207	208	206	202	204	207	202	216	202	202	205	205	203	201	202	201	207	204	201	200
146	146	148	147	145	147	148	147	147	144	145	146	144	140	142	144	145	148	144	143	145
56	56	55	52	55	59	57	56	55	54	55	56	54	57	51	53	55	52	55	56	54
15	13	12	12	14	14	12	13	13	12	14	13	13	14	14	12	14	13	14	14	12
4701730	4683529	4655773	4583235	4625748	4793674	4624618	4701739	4794010	4508374	4652170	4631272	4548865	4647903	4543977	4571792	4568634	4583168	4622608	4630279	4573097
4606470	4592461	4560038	4490944	4533254	4701278	4535620	4605230	4661495	4425826	4561084	4541196	4459674	4559510	4456591	4476007	4482037	4491310	4526066	4537442	4485549
4277475	4266266	4218350	4160218	4195213	4364075	4190669	4258743	4253983	4107282	4213409	4200114	4131895	4207961	4109446	4136069	4155812	4160894	4175887	4196024	4141100
3810948	3801858	3765267	3708147	3760488	3933715	3737343	3835597	3736724	3669563	3781464	3754039	3669764	3738761	3662269	3693210	3726468	3711150	3714036	3755766	3719145
2371487	2348980	2275374	2172223	2336904	2524509	2278247	2370465	2269278	2222065	2337482	2295835	2221673	2401710	2189040	2225165	2313246	2170208	2299516	2376383	2254139
961260	851696	765222	777773	916015	959165	726135	857746	828026	766410	906134	817592	813457	907546	913392	794405	901739	819102	891864	912906	786591
377	370	380	369	370	376	379	375	426	361	369	372	369	377	373	373	364	371	374	374	366
79744	87185	79744	90547	86480	108225	79744	86455	79741	79744	79744	79744	79744	84278	84254	95474	79744	76594	79744	82867	85850
4644406	4627632	4595781	4525841	4568814	4739487	4570516	4643446	4705997	4458991	4594634	4575507	4495203	4595419	4491025	4514391	4516058	4525776	4562463	4575085	4518995
50.74	50.75	50.86	50.77	50.86	50.66	50.79	50.88	50.47	50.74	50.86	50.86	50.67	50.66	50.85	50.84	50.79	50.77	50.76	50.86	50.75
25293	25293	24997	24233	25592	26881	24997	25293	23401	24997	25293	25266	24912	25938	24616	24576	25592	24233	25266	25656	24997
12648	12648	12612	12490	12648	13955	12395	13422	11899	12647	13158	13158	12491	12649	12491	12610	12649	12395	12198	12610	12649
55	55	56	56	53	53	58	55	59	55	54	56	56	53	54	55	53	56	55	53	55
118	118	120	120	116	115	121	117	129	116	116	118	118	115	117	118	116	121	119	115	117
0	0	0	0	0	0	0	0	0	0	0	0	0	0	0	0	0	0	0	0	0

R16	R17	R18	R19	R20	R21	R22	R23	R24	R25	R26	R27	R28	R29	R30	R31	R32	R33	R34	R35	R36
685	665	687	812	672	681	911	858	793	817	690	778	833	829	738	834	784	690	955	703	841
322	320	318	347	319	319	360	359	351	356	324	315	361	355	326	349	332	313	352	323	361
204	202	199	213	201	202	216	215	210	212	208	200	218	212	200	212	206	198	203	204	216
144	144	143	146	144	143	146	146	146	147	148	147	150	145	137	144	147	142	140	145	147
53	55	54	56	54	56	57	57	55	57	54	59	60	55	55	56	56	53	59	55	58
13	14	14	14	12	15	14	14	13	14	13	17	14	13	13	14	14	12	20	13	14
4581961	4625266	4572783	4777500	4598317	4636738	4834869	4811303	4735736	4821869	4613621	4846236	4942774	4741613	4523735	4738096	4778680	4468857	5025489	4629407	4877827
4489239	4537230	4481721	4662663	4507232	4542700	4700414	4686379	4623883	4707523	4518848	4731077	4826542	4623618	4420643	4621172	4665367	4375462	4875830	4535417	4759283
4154687	4201481	4137412	4285421	4169466	4208434	4307273	4288774	4220020	4304256	4181508	4428634	4423828	4227476	4062733	4235120	4307874	4051595	4507338	4198974	4351956
3692394	3759261	3709031	3782278	3732283	3756850	3782470	3769629	3731860	3812815	3723132	4043051	3918817	3730848	3593927	3725664	3863250	3621600	4046934	3752855	3843558
2214846	2338809	2298461	2337243	2282114	2372314	2357299	2346657	2278205	2372050	2233478	2558972	2490618	2285777	2273000	2325440	2399800	2192434	2722824	2318140	2436204
830255	917924	914404	881659	811529	964805	884048	883582	819618	882541	830893	1096328	869721	835554	827697	879398	923868	766072	1333778	851243	904450
371	369	367	405	368	371	422	419	411	414	377	366	414	416	378	401	386	361	424	372	417
79744	88184	85850	79744	112220	87272	79744	79744	79744	79744	79744	87443	79744	84245	79744	79744	87422	79744	87227	86495	79744
4524511	4572570	4516819	4704520	4542514	4579943	4745069	4729764	4667524	4749398	4556942	4767822	4865652	4667316	4457876	4658800	4704965	4410388	4926083	4570884	4799326
50.76	50.86	50.75	50.5	50.76	50.65	50.51	50.52	50.55	50.4	50.75	50.95	50.6	50.54	50.5	50.54	50.83	50.79	50.53	50.85	50.56
24575	25592	25293	24997	25266	25656	24997	24997	24463	24997	24114	26880	25656	24912	25293	24988	25592	24997	27940	25293	25293
12395	12649	12612	12037	13158	12647	11939	11900	11968	12037	12471	14531	12193	11939	12198	11940	13158	13158	13811	12612	12037
55	53	53	57	54	53	58	58	58	58	56	53	58	57	54	57	55	54	50	54	57
118	116	115	124	116	115	126	126	126	125	121	110	127	125	115	124	118	115	111	117	126
0	0	0	0	0	0	0	0	0	0	0	0	0	0	0	0	0	0	0	0	0

R37	R38	R39	R40	R41	R42	R43	R44	R45	R47	R48	R49	R50	R51	R52	R53	R54	R55	R56	R57	R58
707	733	686	689	810	677	674	681	677	683	680	690	805	701	794	716	678	686	658	742	716
359	330	319	320	351	319	322	319	317	328	320	317	351	325	334	329	320	304	316	334	339
211	206	202	202	212	204	207	203	201	206	203	203	214	204	208	206	200	196	202	207	209
148	145	143	142	146	144	145	143	144	148	145	141	143	142	151	148	141	141	146	150	148
59	57	54	54	56	54	55	56	55	56	55	55	55	56	57	55	54	58	55	55	56
15	14	12	15	11	13	13	14	14	11	13	15	14	15	12	13	14	18	14	11	14
4890699	4656304	4524124	4551387	4724690	4607423	4606648	4610105	4624612	4652877	4626310	4611537	4728046	4587923	4734859	4646752	4569507	4739130	4625266	4691346	4724157
4799040	4555388	4434289	4459915	4612487	4513892	4514395	4517380	4533570	4563036	4533870	4516276	4616578	4494406	4617177	4548379	4477204	4640182	4538961	4586226	4627950
4397027	4200664	4096243	4125271	4220633	4179543	4183092	4182612	4198531	4212617	4200531	4186651	4224979	4143413	4261679	4199618	4136777	4353065	4218698	4220536	4271574
3929661	3736968	3645311	3668549	3722257	3718511	3709922	3721016	3763312	3772659	3757925	3712528	3698821	3676305	3825405	3758293	3685990	3960941	3795268	3787321	3811479
2506126	2344488	2234094	2264376	2262586	2270949	2272989	2329343	2344543	2280470	2311197	2346730	2305005	2319324	2321042	2290641	2298559	2566519	2350855	2246354	2356371
987656	879673	765616	941963	689354	862586	826785	889667	916102	697141	851437	994011	901025	938401	764310	851312	922354	1184560	931266	706787	911537
413	383	365	368	403	371	374	369	364	376	372	371	406	376	392	379	372	355	363	392	390
108319	79744	79744	79744	79744	111107	79744	79744	86455	79744	86455	114264	84903	79744	79744	87665	95252	87443	101414	79744	82148
4839002	4593590	4467457	4494709	4649770	4551674	4551673	4553892	4568186	4598157	4571142	4554889	4656042	4531132	4659000	4584960	4515613	4676201	4572763	4628651	4665746
50.37	50.79	50.81	50.67	50.53	50.76	50.76	50.76	50.86	50.86	50.86	50.76	50.48	50.73	50.83	50.8	50.84	50.9	50.87	50.81	50.87
25656	25293	25266	25266	24233	24997	24997	25592	25592	24997	25293	25592	24997	25592	24997	24997	25293	27269	25592	24576	25266
12395	12198	12395	12534	11968	12471	12471	12471	12649	13158	12647	12395	11897	12194	12649	12569	12610	14922	13422	12612	12534
56	56	54	54	59	55	56	54	53	57	54	53	56	54	58	56	53	50	53	58	56
122	120	117	116	126	118	119	117	115	120	117	115	125	118	122	120	115	105	115	123	121
0	0	0	0	0	0	0	0	0	0	0	0	0	0	0	0	0	0	0	0	0

R59	R60	R61	R62	R63	R64	R65	R66	R67	R68	R69	R70	R71	R72	R73	R74	R75	R76	R77	R78	R79
666	683	776	666	856	895	799	723	798	651	693	751	888	781	782	811	715	829	698	679	842
314	324	340	319	355	366	349	329	359	304	326	343	364	341	345	357	342	354	324	327	355
202	200	208	204	215	217	207	206	215	193	207	210	215	206	209	214	212	212	204	205	214
145	142	148	147	148	150	142	146	151	140	145	149	144	146	144	150	148	146	146	146	148
54	56	59	54	56	55	57	54	58	49	55	56	55	55	56	58	56	58	57	55	57
11	13	14	13	13	12	13	13	13	10	13	12	14	13	13	12	13	14	13	11	12
4521206	4596394	4817358	4593204	4792850	4819624	4706057	4622547	4814111	4253881	4555945	4762256	4791727	4695850	4750716	4846381	4746566	4823907	4696218	4587368	4810216
4432036	4503631	4709208	4503889	4671136	4686885	4595571	4528136	4703327	4166587	4461958	4653081	4663643	4583378	4639132	4734714	4649222	4708295	4604979	4496975	4692009
4110093	4149181	4334502	4177374	4272121	4273693	4194208	4184811	4308014	3842705	4120030	4284467	4259924	4206522	4251722	4340615	4287572	4307992	4261908	4154757	4295985
3678148	3711174	3883571	3745953	3772555	3774449	3705398	3728141	3825615	3438720	3654253	3822279	3732084	3752763	3767479	3858993	3801059	3813283	3820455	3709965	3802583
2214993	2344289	2464911	2252443	2315803	2270042	2352019	2257229	2363749	1981674	2216012	2333554	2320663	2303786	2347804	2378450	2315562	2413569	2403856	2245014	2353267
701622	862899	898999	826490	816691	777886	828312	832669	822577	649126	806970	802316	917206	849224	846399	762363	816232	883667	848469	711622	776934
363	375	395	369	414	428	405	378	417	351	380	406	423	397	407	414	396	409	375	376	413
79744	88246	79768	79744	79744	79744	79744	83991	79744	79744	79744	101421	87386	84254	82867	79744	79744	79744	82148	79744	79744
4467675	4541038	4747867	4540411	4713791	4731215	4635775	4563860	4745039	4200485	4500515	4697427	4705895	4623631	4683795	4775965	4688193	4748530	4642154	4532708	4734155
50.8	50.83	50.78	50.67	50.54	50.53	50.33	50.69	50.52	50.8	50.65	50.72	50.51	50.76	50.47	50.47	50.88	50.57	50.88	50.84	50.58
24997	25592	25592	24997	24912	24051	25293	24997	24997	23401	24973	24997	24912	24997	25266	24997	24114	25293	25656	24997	24997
12647	12614	12614	12649	11939	11900	12038	12395	12027	12198	12198	12491	11703	12182	12193	12198	12612	12037	12646	12608	12037
55	54	56	55	58	59	56	55	59	54	57	57	57	56	56	59	58	57	54	56	58
117	116	120	118	127	130	122	120	128	115	121	123	126	121	122	126	123	124	117	119	126
0	0	0	0	0	0	0	0	0	0	0	0	0	0	0	0	0	0	0	0	0

R80	R81	R82	R83	R84	R85	R86	R87	R88	R89	R90	R91	R92	R93	R94	R95	R96	R97	R98	R99	RY184
688	626	721	775	674	839	685	651	798	690	701	784	776	722	829	927	719	656	705	810	716
325	318	322	347	319	354	319	318	346	319	324	314	313	306	352	359	327	313	325	363	335
202	200	204	209	203	216	200	200	213	201	203	196	197	198	210	212	204	199	207	212	205
145	140	147	144	145	148	141	144	148	141	143	145	145	142	143	148	144	142	145	147	142
55	51	54	57	56	57	53	56	58	54	56	59	61	58	57	58	56	55	56	57	58
14	12	13	14	12	13	13	14	14	12	13	18	18	18	14	14	12	13	14	14	14
4665703	4420325	4678457	4755134	4600157	4834002	4486922	4594282	4852237	4489782	4631109	4847263	4853286	4757069	4823793	4860298	4647523	4519650	4679179	4807406	4725585
4571682	4339153	4577363	4648025	4509800	4716656	4399016	4509437	4737158	4397908	4537158	4724414	4732883	4654866	4704041	4734547	4546330	4431821	4583327	4696336	4612381
4219345	4005570	4242830	4262199	4181893	4330013	4057217	4178395	4363473	4058564	4197738	4423191	4435366	4372618	4305283	4314004	4207852	4106958	4238139	4284110	4250620
3784777	3553344	3808641	3767346	3739946	3824247	3605261	3758389	3877821	3598794	3744071	4057498	4065488	3973077	3800060	3830000	3749072	3672008	3761167	3787388	3778521
2342981	2116943	2309566	2385832	2315143	2370144	2202917	2360523	2435474	2208131	2347082	2612807	2664039	2561794	2430506	2388207	2341768	2288240	2330913	2360953	2425332
919177	765890	866551	911213	782939	849668	829716	911927	883497	764552	851200	1160108	1162042	1162208	929394	881688	787721	829451	871919	871924	913143
380	365	376	397	368	412	362	364	406	365	373	377	368	354	411	420	383	363	376	419	395
90466	79744	93404	81851	81792	83259	79744	82148	79744	79744	86471	88000	87443	87443	101271	79744	84244	79744	79744	79744	88310
4610617	4372776	4616973	4684563	4546088	4759470	4430330	4543133	4780962	4431052	4572509	4768128	4771338	4689224	4746479	4778658	4586362	4467253	4620710	4737476	4653879
50.72	50.77	50.86	50.44	50.71	50.56	50.79	50.84	50.47	50.79	50.86	50.86	50.84	50.89	50.58	50.42	50.82	50.79	50.66	50.57	50.8
25293	23587	25266	25266	25266	24997	24997	25656	25293	24997	25592	27494	28149	27465	25592	24997	25293	25592	25266	24997	25720
12649	12197	13421	12037	13158	12037	12198	13158	12239	12194	12647	14294	14629	14124	12037	12038	12649	12647	12569	11968	12713
54	54	54	56	55	58	54	53	57	55	54	51	51	50	55	59	55	53	56	58	55
117	116	117	122	116	126	116	114	123	117	116	107	106	106	122	127	117	114	120	126	117
0	0	0	0	0	0	0	0	0	0	0	0	0	0	0	0	0	0	0	0	0

sh05sh293	sh05sh574	sh08sh072	sh08sh212	sh08sh242	sh09sh134	sh09sh171	sh10D164	sh10sh001	sh10sh003	sh10sh012	sh10sh016	sh10sh019	sh10sh053	sh10sh098	sh10sh108	sh10sh111	sh10sh112	sh10sh121	sh10sh122	sh10sh126
556	727	650	639	769	771	618	793	744	680	664	784	762	632	822	614	808	756	611	841	608
359	352	323	331	319	320	327	359	360	337	359	365	363	327	365	303	338	363	331	340	302
219	219	202	211	196	200	205	217	222	208	222	212	223	205	220	191	205	222	208	204	192
134	150	145	142	142	143	143	149	152	144	151	142	152	146	147	137	148	150	144	146	138
49	58	58	57	54	53	58	61	62	57	60	58	62	59	58	60	56	63	58	55	60
6	13	15	14	14	13	15	15	14	12	13	15	14	15	13	20	14	17	14	13	20
4331617	4885670	4636539	4654598	4506423	4509161	4729721	4913117	4944564	4615757	4863570	4838932	4947650	4738100	4817205	4781336	4682036	5012069	4671887	4643500	4780748
4263987	4766453	4536118	4557773	4412727	4414515	4635079	4781502	4823703	4516007	4765285	4681865	4824249	4636716	4677929	4678079	4583722	4886441	4577596	4536413	4679522
3883752	4408888	4202026	4230099	4068656	4078123	4299319	4401596	4454654	4165963	4400161	4285348	4449743	4304276	4285757	4384680	4217203	4509885	4239843	4155269	4390775
3253714	3894496	3778766	3719714	3650814	3640415	3836651	3903221	3941993	3683551	3879582	3759359	3931414	3862054	3756028	3985384	3780103	3989316	3767252	3708545	4000123
1904539	2425208	2398344	2366392	2266368	2218505	2478782	2509843	2515040	2301440	2438185	2415770	2505839	2477098	2354734	2693339	2311426	2625411	2385781	2257818	2701770
389089	875658	947882	892095	903264	846208	1002004	944038	878902	765741	826562	942224	878902	976641	826650	1310672	892179	1070576	878849	839664	1310940
401	415	377	384	367	368	382	424	422	392	419	463	426	388	436	366	391	435	389	396	363
75806	107232	88310	88310	79708	80653	107234	88310	88309	88269	88309	88342	88309	88310	88310	87507	79708	88310	88309	79708	87507
4293236	4810660	4574811	4594947	4446220	4448075	4673633	4827967	4868564	4554144	4807207	4751008	4869037	4680308	4728510	4721424	4620938	4936664	4618865	4576463	4722257
51.35	50.47	50.82	50.8	50.88	50.84	50.7	50.59	50.6	50.81	50.58	50.77	50.61	50.87	50.53	50.92	50.83	50.46	50.82	50.84	50.92
21320	25061	25755	25357	25257	24961	25873	25720	25658	25021	25330	25362	25357	25872	24459	28097	25230	26000	25720	24359	30017
10502	12256	13486	12257	12359	12359	13487	12262	12257	12222	12257	12035	12102	14004	11964	14986	12574	12257	12708	12157	14986
60	58	54	55	53	54	53	58	59	57	59	57	60	54	59	48	56	57	55	57	48
131	125	115	118	115	116	115	124	127	121	127	125	128	116	128	102	121	125	118	122	102
0	0	0	0	0	0	0	0	0	0	0	0	0	0	0	0	0	0	0	0	0

sh10sh144	sh10sh145	sh10sh147	sh10sh151	sh10sh159	sh10sh166	sh10sh167	sh10sh172	sh10sh173	sh10sh176	sh10sh187	sh10sh214	sh10sh217	sh10sh229	sh10sh234	sh10sh235	sh10sh236	sh10sh237	sh10sh249	sh10sh253	sh10sh256
734	803	595	626	749	735	1086	720	601	745	718	834	716	633	710	718	707	710	736	606	621
357	330	306	324	363	355	420	346	321	359	359	363	358	332	351	349	341	348	356	326	327
215	200	194	205	218	214	252	213	201	220	215	214	216	205	217	214	212	216	221	206	206
148	144	139	141	149	146	163	146	142	151	147	149	143	140	143	145	147	144	149	145	144
58	52	63	55	59	57	52	59	58	62	62	60	57	57	58	57	60	57	60	58	57
16	13	19	14	15	13	11	15	15	14	16	14	14	15	14	14	14	14	14	13	14
4865189	4557578	4826551	4558882	4910033	4789331	5117257	4821123	4643435	4936413	4995231	4880878	4839479	4652745	4777059	4783372	4801521	4691522	4894136	4647169	4658751
4739141	4460443	4727107	4457924	4788639	4670004	4927957	4704725	4552224	4814312	4879074	4752752	4721584	4552313	4662909	4666661	4684925	4576529	4773415	4554385	4562689
4351591	4104169	4435129	4128589	4399838	4284330	4478256	4348245	4228612	4436699	4485800	4349516	4336268	4206605	4299726	4299791	4336313	4221570	4409087	4218541	4231473
3861176	3673204	4034735	3657937	3898906	3780844	3835311	3858491	3783947	3929585	3987781	3867946	3792733	3724657	3759338	3792895	3864563	3694845	3884613	3755677	3764286
2424436	2204024	2764899	2288869	2467231	2356004	2045743	2487522	2458856	2518112	2628188	2449697	2417585	2406685	2416040	2382491	2483428	2330612	2471570	2376474	2390678
999235	862079	1225552	890099	954278	826511	696316	976535	991534	878262	1050933	880973	939831	977173	913704	913185	906946	889794	889934	841279	914356
430	379	368	390	427	417	503	406	374	423	423	427	425	391	411	409	403	409	420	382	383
88310	88210	87507	88310	88310	88310	88310	88310	101438	88310	107469	88269	113363	88310	88310	88308	88310	88310	88310	88310	88310
4791919	4494498	4770340	4504083	4833587	4713555	4985603	4747326	4589856	4859416	4924836	4797229	4768451	4593989	4705467	4709356	4728445	4619515	4819082	4594105	4602015
50.56	50.85	50.85	50.77	50.62	50.51	50.58	50.49	50.76	50.54	50.4	50.57	50.48	50.8	50.53	50.52	50.45	50.47	50.6	50.76	50.79
25330	24359	30300	25330	25720	24459	21231	25658	26002	25720	26002	25317	25330	25873	25357	25330	25755	25061	25357	25658	25720
12256	12533	15329	12262	12256	12102	10602	12459	13487	12262	12712	12222	12032	12459	12042	12262	12713	12003	12257	12711	12535
57	54	49	54	57	58	72	55	52	59	56	58	56	53	56	56	56	57	58	55	54
125	118	103	117	125	125	154	120	113	126	122	125	124	116	123	122	120	124	125	118	117
0	0	0	0	0	0	0	0	0	0	0	0	0	0	0	0	0	0	0	0	0

sh10sh266	sh10sh273	sh10sh274	sh10sh285	sh10sh286	sh10sh288	sh10sh294	sh10sh296	sh10sh297	sh10sh299	sh10sh2	sh10sh304	sh10sh62	sh11sh002	sh11sh041	sh11sh071	sh11sh074	sh11sh079	sh11sh103	sh11sh105	sh11sh108
702	773	609	744	738	770	604	715	730	740	795	620	724	718	795	721	749	914	805	732	776
352	361	326	362	356	370	320	352	359	356	323	328	356	350	328	352	354	335	328	357	330
208	222	205	221	218	221	204	216	221	221	198	207	217	214	204	213	217	205	208	215	203
145	149	143	149	150	149	142	147	150	151	143	141	146	145	146	146	146	147	145	148	147
57	61	58	59	60	61	57	59	59	59	53	57	60	58	57	59	60	56	54	56	55
14	14	14	13	13	14	15	13	14	13	12	14	14	15	12	14	16	13	13	14	12
4682983	4947309	4617006	4892284	4886647	4937417	4621183	4791042	4887739	4882401	4467079	4662123	4831984	4795548	4591454	4787628	4858598	4692237	4589733	4798946	4604746
4566987	4818483	4522576	4769506	4765941	4807481	4528380	4676740	4769937	4763488	4370211	4565508	4713991	4678365	4495652	4667722	4737303	4578011	4489978	4675710	4512350
4175405	4446512	4193743	4388653	4391153	4400642	4210063	4308435	4402318	4401667	4018855	4229952	4344013	4305955	4151779	4291227	4367807	4212346	4152808	4297523	4157684
3715422	3912796	3731270	3860899	3886943	3869568	3754784	3806830	3885491	3888129	3592707	3733040	3822528	3799815	3705771	3799694	3851637	3767385	3666517	3806495	3727749
2335877	2500856	2380587	2404691	2455586	2482353	2407904	2417643	2423954	2406482	2169991	2390047	2466442	2431878	2292224	2412223	2494990	2316388	2229350	2343387	2265390
880948	878902	880677	826149	826515	876282	983814	826056	890042	826782	766203	914280	894563	983156	742400	880499	996803	849958	845119	911187	773906
418	426	382	427	420	438	376	412	422	417	372	385	421	412	377	416	417	386	378	428	378
88310	88309	88310	88310	88310	88310	93584	88310	88310	88310	79706	88310	88310	93583	79708	88310	88310	84319	80622	88310	82864
4613129	4864252	4562131	4815495	4811020	4855239	4568659	4719322	4814371	4806772	4404439	4605732	4759533	4723097	4529749	4713744	4782452	4613105	4524637	4725919	4545890
50.54	50.61	50.85	50.61	50.62	50.6	50.84	50.53	50.61	50.63	50.75	50.81	50.47	50.55	50.77	50.53	50.59	50.81	50.77	50.52	50.87
25330	25357	25658	24996	25330	25535	25755	25357	25061	25061	24359	25720	25720	25357	25257	25357	25755	25230	24359	24459	24961
12101	12262	12713	12102	12102	12003	13222	12102	12257	12262	12162	12535	12102	12101	12533	12262	12257	12359	12001	12102	12533
56	59	55	60	59	59	53	57	59	59	55	54	57	56	56	57	56	56	56	57	56
122	126	116	127	126	129	114	124	126	126	118	117	124	122	119	123	123	120	121	125	119
0	0	0	0	0	0	0	0	0	0	0	0	0	0	0	0	0	0	0	0	0

sh11sh109	sh11sh125	sh11sh133	sh11sh179	sh11sh201	sh11sh202	sh11sh209	sh11sh210	sh11sh211	sh11sh220	sh11sh221	sh11sh225	sh11sh241	sh11sh244	sh11sh270	sh11sh272	sh11sh277	sh11sh290	sh11sh293	sh11sh297	sh11sh301
736	575	880	798	943	1015	863	615	622	869	624	621	823	555	614	612	659	617	800	600	606
356	325	339	330	359	361	333	328	323	331	324	321	333	309	329	330	342	325	327	328	328
219	209	205	201	212	211	204	203	208	203	208	207	200	196	208	208	208	209	200	207	206
147	146	147	143	146	144	147	146	147	144	146	143	146	140	145	146	146	149	142	144	143
59	60	52	54	56	54	53	59	58	53	61	58	54	57	59	59	59	59	53	59	59
15	17	12	13	13	12	12	16	15	14	14	15	12	15	15	14	14	15	14	16	16
4853697	4830354	4598805	4568455	4723342	4697911	4600133	4761182	4756197	4597918	4774763	4651729	4587550	4546911	4713715	4718541	4745560	4760072	4537776	4717252	4718706
4734014	4741821	4487693	4472707	4606687	4565547	4492144	4665824	4658570	4487580	4677917	4552924	4485736	4462407	4619903	4624591	4640361	4664507	4444528	4626094	4625590
4364375	4419059	4119695	4119623	4209660	4155364	4130421	4324609	4340196	4133798	4356842	4236881	4115986	4153669	4290072	4291033	4279284	4342105	4090821	4296478	4289116
3838134	3946126	3674009	3674486	3715614	3642874	3691352	3899640	3877933	3683591	3895267	3758402	3697133	3736867	3826959	3829228	3817745	3889355	3641615	3829219	3817952
2442057	2584248	2163804	2258166	2287149	2192320	2188109	2508710	2472032	2234217	2541928	2411652	2235471	2416359	2459821	2441404	2426243	2479002	2226018	2479851	2479678
932959	1131568	796221	849758	819647	747692	791493	1039161	1015509	913543	908242	967190	796089	991922	958887	895202	889678	1000920	913543	1017446	1017444
419	374	391	377	413	420	384	385	382	382	382	382	385	364	386	385	402	383	371	381	387
88310	107429	84201	84201	79708	79706	84201	88310	109046	84201	88310	88310	84193	101469	88310	88310	88311	109046	84201	88310	88310
4778297	4777270	4524113	4505601	4644263	4606920	4527387	4706164	4699866	4523436	4719489	4596274	4522291	4501308	4659372	4663118	4683117	4704773	4475924	4663603	4667132
50.59	50.59	50.83	50.81	50.54	50.57	50.84	50.73	50.66	50.83	50.76	50.75	50.82	50.85	50.63	50.61	50.78	50.66	50.86	50.61	50.61
25357	26882	23365	25230	24359	23365	23764	26002	25720	24359	27048	25755	24359	26002	25872	25720	25720	25755	24961	25873	25873
12102	14015	12157	12533	11903	11667	12359	14019	13224	12162	14004	13224	12533	14004	13486	13487	12713	13223	12162	13487	13487
57	53	57	54	58	59	57	53	54	55	54	54	56	51	54	55	56	55	54	54	54
125	115	122	117	126	128	121	115	117	119	116	116	119	110	117	118	120	118	117	116	116
0	0	0	0	0	0	0	0	0	0	0	0	0	0	0	0	0	0	0	0	0

sh11sh306	sh11sh324	sh11sh347	sh11sh353	sh11sh359	sh11sh374	sh11sh376	sh11sh385	sh11sh386	sh11sh387	sh11sh393	sh11sh398	sh11sh417	sh11sh418	sh11sh458	sh11sh459	sh11sh483	sh11sh487	sh11sh488	sh11sh492	sh11sh516
723	603	732	606	895	650	751	754	758	703	722	730	563	578	724	622	710	579	615	584	710
351	329	355	325	389	316	355	357	353	352	355	355	314	322	357	332	352	331	323	320	343
211	207	216	202	217	203	217	219	219	214	212	217	198	208	216	209	213	213	207	201	211
144	145	142	143	146	143	149	147	148	146	144	146	141	144	147	145	147	146	146	144	144
58	59	60	58	59	60	59	60	60	59	59	59	58	59	61	59	61	57	58	59	58
16	15	14	15	14	21	15	13	13	15	15	16	14	16	15	15	17	16	13	14	14
4790385	4717487	4809199	4633704	5015733	4817114	4862137	4859102	4859710	4800811	4803325	4857730	4579165	4695687	4874136	4720345	4918673	4717147	4638904	4640060	4774644
4673839	4624363	4687692	4539916	4844883	4703216	4736578	4731996	4730525	4688374	4686955	4739043	4495132	4609854	4757797	4624546	4801970	4624655	4543577	4549750	4655232
4295278	4291934	4301137	4195768	4382271	4421628	4361849	4355085	4362211	4311252	4294419	4364350	4182181	4287310	4383318	4286883	4423063	4307845	4215977	4222645	4287782
3800492	3829682	3761117	3759380	3860573	3988722	3860254	3829874	3844956	3814174	3793083	3843553	3755092	3798934	3873768	3810563	3933003	3800001	3756130	3797773	3795731
2437443	2462439	2455138	2406810	2473437	2625551	2430469	2443267	2448745	2442249	2441105	2460709	2425832	2469863	2511466	2448408	2563060	2381081	2365326	2444846	2424828
1013392	955161	889380	943642	921156	1321232	938746	826056	826479	951464	951135	996562	913439	1028891	944243	949942	1070509	1004388	838504	900782	921033
410	384	415	382	483	379	423	421	418	411	415	416	366	368	417	388	415	386	382	378	407
88310	88310	88310	88310	95007	82612	88310	88310	88310	88310	88310	88310	88310	88342	88310	88310	88310	88342	88310	88310	93946
4716222	4663517	4729560	4580467	4912190	4748502	4784330	4777274	4777293	4730821	4730042	4782639	4531878	4642264	4800875	4664093	4847862	4665131	4584767	4590490	4701432
50.54	50.61	50.6	50.82	50.55	50.94	50.54	50.57	50.57	50.54	50.54	50.58	50.86	50.74	50.5	50.62	50.54	50.67	50.75	50.76	50.49
25720	25873	25499	25755	25061	27203	25330	25357	25357	25658	25658	25357	26002	25905	25720	25755	25873	25362	25357	25762	25357
12387	13487	12101	13487	11767	14593	12256	12102	12262	12102	12102	12102	14086	12294	12262	12711	12562	12491	12563	14019	12674
55	54	57	54	59	51	58	58	58	56	56	57	52	54	57	55	56	56	56	54	56
121	117	123	115	130	109	125	126	125	123	123	124	111	116	123	118	122	120	119	114	121
0	0	0	0	0	0	0	0	0	0	0	0	0	0	0	0	0	0	0	0	0

sh11sh529	sh11sh78	sh12D205	sh12D215	sh12sh010	sh12sh046	sh12sh051	sh12sh059	sh12sh100	sh12sh112	sh12sh113	sh12sh123	sh12sh133	sh12sh144	sh12sh169	sh12sh177	sh12sh190	sh12sh191	sh12sh196	sh12sh197	sh12sh200
726	799	611	632	992	644	641	562	941	597	572	966	626	890	606	618	782	628	705	608	586
358	330	322	327	368	324	320	318	357	317	319	333	334	345	317	322	327	320	343	317	315
216	203	207	207	215	209	205	203	209	202	204	205	210	207	205	203	203	204	215	202	203
146	147	143	146	151	145	144	144	146	144	142	147	145	145	145	144	146	146	147	145	144
59	56	59	59	56	60	59	59	57	59	58	54	59	54	59	56	52	57	58	56	57
15	12	16	14	12	16	16	14	14	15	15	13	15	13	14	15	13	15	14	14	15
4865650	4611895	4732810	4723911	4800327	4793083	4735482	4641496	4771867	4633896	4630472	4674677	4740468	4658050	4672143	4630670	4576033	4660014	4794622	4593870	4627244
4747499	4513975	4637879	4624072	4664377	4695125	4637914	4554132	4652902	4542530	4539878	4549511	4642335	4544047	4580325	4535397	4481773	4563067	4679941	4500408	4536112
4360983	4157219	4318806	4295737	4249536	4377701	4316958	4232047	4250160	4224534	4224519	4192190	4303290	4155256	4272253	4205288	4135220	4241836	4330074	4182959	4225459
3847274	3728872	3843598	3841130	3761093	3906480	3864898	3793130	3775473	3790031	3759406	3746997	3823466	3682566	3827278	3770384	3694073	3809898	3837450	3758442	3789460
2451649	2291336	2510553	2447928	2258651	2559332	2521469	2431073	2358230	2442289	2420868	2279943	2455007	2235203	2455505	2360466	2196534	2397337	2415106	2340223	2400510
953317	773307	1044904	913507	748982	1055815	1055391	890989	888000	943465	950520	860637	964928	819476	913273	972064	849719	983049	912273	919283	976071
419	380	382	386	437	380	375	372	412	373	372	386	393	401	371	377	375	375	405	370	370
88310	82104	95984	88310	79708	90614	90623	88342	88210	88310	88342	95107	88309	79708	88310	88310	84081	92810	88310	92501	88310
4790946	4549005	4679602	4666412	4712875	4734954	4676935	4592558	4691206	4581928	4577253	4586375	4684058	4583113	4618789	4574825	4514777	4601930	4723616	4538443	4575436
50.57	50.86	50.9	50.86	50.46	50.73	50.84	50.7	50.53	50.79	50.7	50.87	50.89	50.42	50.72	50.69	50.69	50.69	50.41	50.81	50.68
25357	25230	26002	25755	23281	27048	27048	25752	25230	25872	25905	24961	25720	24338	25872	25357	24338	25720	25357	25357	25755
12257	12359	13486	13983	11796	13983	13983	14036	12002	13983	13771	12162	12713	11932	14004	13487	12533	13487	12676	13872	13983
57	56	53	55	61	53	52	54	57	54	53	55	55	57	54	54	55	54	56	54	53
124	119	115	117	131	115	114	115	124	114	114	120	118	123	115	115	119	116	121	115	114
0	0	0	0	0	0	0	0	0	0	0	0	0	0	0	0	0	0	0	0	0

sh12sh212	sh12sh213	sh12sh215	sh12sh216	sh12sh217	sh12sh220	sh12sh252	sh12sh256	sh12sh257	sh12sh260	sh12sh266	sh12sh284	sh12sh295	sh12sh30	sh12sh45	sh12sh50	sh12sh55	sh13D178	sh13sh002	sh13sh037	sh13sh069
797	768	758	739	604	720	574	785	761	825	953	622	741	805	783	828	822	672	491	624	546
324	322	324	348	314	345	329	323	408	354	413	326	358	327	328	356	327	330	347	328	318
200	198	197	209	202	213	207	200	241	211	216	208	219	199	202	211	205	207	220	204	206
146	143	144	146	145	147	143	144	150	148	143	145	149	142	145	146	147	146	133	143	144
54	53	51	58	56	59	58	54	43	54	57	58	59	54	53	54	55	61	50	59	58
13	12	12	15	14	15	14	13	6	13	12	12	14	13	13	13	13	18	10	16	14
4570200	4454722	4454908	4780619	4597803	4823056	4643272	4544446	4575812	4716128	4966779	4617123	4889454	4533528	4549254	4667847	4620297	4892486	4376606	4729125	4627069
4469477	4361934	4361221	4650500	4503624	4704090	4557298	4447099	4458476	4618168	4756135	4514680	4764734	4436275	4454312	4568078	4517829	4778225	4326569	4632663	4542113
4123215	4012448	4003252	4279985	4193159	4344606	4217316	4104425	4027423	4229831	4244804	4187689	4389068	4087977	4098355	4176677	4176364	4435721	3961476	4299169	4237423
3707677	3587279	3588600	3813782	3768784	3865901	3739074	3676010	3366775	3746702	3696556	3716640	3875841	3654563	3660270	3688661	3729596	3975770	3316743	3843329	3770371
2255741	2171885	2117535	2415580	2344997	2476603	2386370	2255178	1642589	2245123	2315904	2333488	2432109	2255856	2201553	2228014	2267553	2631657	2029718	2504453	2404052
849758	764946	764630	946382	918442	978636	875784	849165	398432	836746	748842	776427	886885	850288	849277	838802	834882	1165172	640426	1035061	888188
375	371	372	427	370	412	374	373	481	401	544	391	424	375	378	408	378	396	375	383	370
84201	79708	79706	88310	91661	98163	88342	83525	81622	79705	88343	88310	88310	84209	84209	79708	79708	88310	75879	88311	88342
4505922	4395613	4395142	4706297	4543449	4751615	4590233	4481996	4512771	4651361	4848347	4560612	4810628	4469653	4489595	4604379	4553401	4825200	4345543	4671559	4579384
50.82	50.78	50.78	50.92	50.69	50.55	50.79	50.85	50.79	50.41	50.58	50.76	50.58	50.85	50.85	50.54	50.68	50.75	51.37	50.88	50.71
25230	24438	23365	25357	25357	25720	25688	25230	19082	24270	23413	25357	25330	25230	24359	23764	24961	27067	22253	26003	25787
12533	12002	12157	12678	14004	12459	12708	12533	9888	12001	10888	12535	12257	12574	12162	11903	12533	13983	10609	14004	13518
54	55	55	56	54	56	55	54	72	58	62	56	58	54	55	58	56	53	57	53	54
118	118	118	121	114	121	118	117	152	125	138	119	126	116	119	125	120	116	128	114	115
0	0	0	0	0	0	0	0	0	0	0	0	0	0	0	0	0	0	0	0	0

sh13sh134	sh13sh135	sh13sh142	sh13sh147	sh13sh150	sh13sh152	sh13sh178	sh13sh180	sh13sh33	sh13sh47	sh13sh51	sh13sh61	sh13sh65	sh14sh003	sh14sh004	sh14sh014	sh14sh038	sh14sh043	sh14sh045	sh15sh100	sh15sh101
574	609	856	611	620	601	614	603	928	683	955	843	933	620	622	695	617	590	604	1624	651
325	323	376	325	335	335	322	318	355	301	360	334	357	326	324	353	326	324	334	668	320
205	204	213	204	205	201	203	202	212	186	211	202	207	205	203	211	204	201	200	355	199
143	141	146	144	142	142	144	142	143	135	146	146	146	142	143	145	142	140	139	152	143
59	57	58	60	53	55	59	58	55	55	55	55	56	59	59	56	60	57	58	58	58
15	15	17	16	13	14	16	15	11	12	13	13	14	16	16	14	17	15	15	16	15
4701108	4642929	4907535	4724406	4476935	4552351	4725633	4637527	4604428	4364090	4721814	4698150	4721156	4736723	4741889	4764344	4788355	4632048	4666009	6477520	4643706
4610354	4546498	4764693	4630448	4380708	4459709	4627677	4545302	4488425	4280143	4602283	4594421	4603940	4641504	4643562	4650921	4690128	4541102	4571234	6127802	4543711
4280314	4212303	4324738	4290589	4029069	4100164	4292938	4223862	4099427	3961006	4198426	4226060	4191695	4307736	4308075	4267511	4352306	4194472	4199157	5298413	4200702
3807956	3737285	3826661	3837685	3561538	3662744	3850165	3773206	3574626	3567067	3707632	3793131	3727711	3839622	3856620	3780985	3889151	3735822	3730250	3961898	3777264
2448643	2402872	2431877	2502239	2160874	2294407	2509567	2443390	2171990	2270040	2262096	2347863	2307731	2521895	2522634	2358560	2575806	2417472	2438377	2496160	2445824
944426	983231	1058301	1005834	836825	920955	1043537	976350	704176	793773	819775	901017	883718	1056174	1055679	927158	1109726	983809	969732	1069392	984342
379	382	443	380	393	391	381	371	409	348	416	389	411	381	382	416	389	378	397	862	377
88342	93583	88311	88310	88311	88311	91220	88310	79708	84218	79705	111776	79708	91011	91011	88310	88779	93946	93962	93946	94181
4649102	4588025	4812381	4669887	4422531	4500639	4669751	4582852	4526764	4313326	4641052	4632193	4641804	4680508	4684488	4694986	4734446	4579671	4615381	6253580	4584840
50.75	50.82	50.5	50.75	50.7	50.84	50.83	50.77	50.55	50.92	50.54	50.7	50.55	50.84	50.82	50.7	50.82	50.84	50.89	49.08	50.84
25752	25755	25330	26002	23864	25330	26002	25873	23764	25902	24338	25257	24961	27048	27048	25061	27067	25873	26019	17519	25963
13256	12535	12004	13487	12102	12262	13487	13222	11576	13883	11903	12574	11932	13487	13983	12262	14019	12711	12599	6276	13447
55	53	57	54	56	54	53	53	59	51	58	54	57	52	52	56	52	52	53	89	52
116	116	127	116	120	117	114	114	127	107	126	119	124	114	114	122	114	114	116	247	114
0	0	0	0	0	0	0	0	0	0	0	0	0	0	0	0	0	0	0	0	0

sh15sh102	sh15sh103	sh15sh104	sh15sh105	sh15sh106	sh15sh107	sh15sh108	sh15sh98	sh15sh99	sh16sh001	sh16sh003	sh16sh004	sh16sh005	sh16sh006	sh16sh007	sh16sh008	sh16sh009	sh16sh255	sh16sh258	sh16sh262	sh16sh264
636	605	629	613	618	609	612	633	624	673	615	566	614	619	612	585	598	557	558	572	565
331	327	325	325	330	328	328	329	328	326	331	327	344	343	332	326	330	324	329	330	322
205	201	203	200	203	201	204	204	201	208	201	204	212	211	209	208	209	201	199	209	205
144	142	141	142	144	142	141	140	141	144	141	143	144	143	143	143	140	141	140	144	144
58	58	58	58	56	58	58	58	58	57	59	59	59	58	58	58	59	59	58	60	58
15	15	15	15	15	15	15	15	15	14	14	14	11	11	14	14	14	15	15	15	15
4677591	4669545	4670150	4666542	4665731	4668858	4672242	4672874	4669437	4678398	4685585	4682683	4679631	4672739	4685016	4677578	4676988	4665373	4663045	4748905	4677329
4573941	4574787	4569700	4569686	4570176	4575384	4574403	4570582	4569223	4574592	4589463	4597019	4590921	4579358	4591570	4589515	4583289	4578862	4576479	4660000	4590593
4228437	4217309	4229866	4221441	4218763	4221779	4233153	4224758	4213025	4248054	4227208	4254381	4224836	4216453	4249660	4256356	4247136	4234985	4213323	4324953	4262598
3771822	3772921	3764940	3780812	3773552	3776399	3761550	3746660	3758382	3777634	3764329	3794399	3714447	3707304	3758143	3766668	3726757	3778709	3763127	3833001	3806219
2417531	2448992	2448974	2448700	2380528	2452586	2448992	2449281	2448562	2397712	2460233	2466239	2367666	2370105	2425906	2419949	2442744	2476770	2453076	2494083	2451306
952819	984057	983805	983828	984070	983743	983822	984218	983658	919934	921898	927656	705643	739132	927304	911420	910726	984648	985043	950401	981990
396	382	383	382	385	383	387	389	387	385	387	374	391	395	384	376	387	378	382	382	371
88311	94181	93946	93946	94181	93946	93946	93584	93792	93583	93615	99893	88342	93616	93616	93616	93615	93615	93615	88340	93615
4620244	4614806	4611068	4610227	4608596	4614434	4616821	4613651	4611759	4616555	4630170	4631518	4624981	4618152	4629504	4626033	4624229	4617874	4614681	4697674	4626336
50.86	50.82	50.87	50.84	50.86	50.83	50.83	50.87	50.83	50.87	50.89	50.88	50.9	50.92	50.88	50.9	50.9	50.88	50.88	50.78	50.89
25755	25762	25873	25762	25720	26002	25762	25873	25873	25720	25905	25905	25389	25389	25752	25750	25905	25905	26034	25905	25905
12676	13222	13222	13222	12711	13222	12711	12711	12676	12676	12707	12743	12133	12050	12491	12486	12289	13254	12743	12743	12743
54	53	53	53	53	53	53	53	53	54	54	54	57	56	54	54	54	53	53	55	53
118	115	115	114	117	115	115	115	115	117	116	116	123	122	118	117	118	114	114	117	115
0	0	0	0	0	0	0	0	0	0	0	0	0	0	0	0	0	0	0	0	0

sh16sh265	sh16sh266	sh16sh267	sh16sh268	sh16sh269	sh16sh270	sh16sh271	sh16sh272	sh16sh273	sh16sh274	sh16sh275	sh16sh276	sh16sh277	sh16sh278	sh16sh279	sh16sh280	sh16sh281	sh16sh283	sh16sh284	sh16sh285	sh16sh286
638	550	593	592	608	546	596	568	569	582	611	572	572	578	597	566	569	908	577	576	568
366	328	330	328	353	330	326	326	330	328	363	326	327	324	326	326	325	650	324	330	327
221	211	209	207	210	204	202	205	206	206	214	208	210	207	205	208	208	421	209	208	208
140	146	144	143	141	141	139	144	142	144	144	148	143	143	143	143	143	263	145	143	144
55	61	60	60	59	58	59	61	61	60	59	60	60	61	59	61	60	91	60	61	61
13	15	15	15	14	14	15	16	15	16	16	15	16	16	14	16	16	23	15	15	15
4681448	4741561	4749315	4742968	4762751	4672194	4671151	4784876	4746004	4739734	4880638	4740392	4739699	4740735	4679980	4737844	4741997	8446288	4734746	4747947	4739467
4581182	4655456	4658169	4646548	4670443	4585950	4580621	4691686	4655611	4648982	4791464	4650618	4646644	4649933	4582946	4649256	4651750	8346323	4647036	4657253	4649200
4186353	4334715	4321104	4304106	4269321	4232158	4228996	4349701	4309111	4308157	4374559	4320266	4326318	4320492	4241594	4325179	4323117	7691954	4326922	4315452	4318668
3590984	3841672	3832682	3819307	3755071	3754018	3752541	3887875	3822366	3842790	3844193	3870305	3826458	3842037	3774881	3842875	3835240	6536472	3840048	3826727	3835904
2282289	2508934	2493578	2492605	2457901	2433690	2477776	2563365	2518257	2507969	2501007	2481784	2504931	2538155	2432565	2539155	2515984	3812006	2492393	2519244	2517965
818843	950740	951460	950346	921626	922196	982364	1039055	950391	1008961	1033308	938524	1014508	1012748	889849	1012838	1015170	1491903	951184	949391	951449
425	381	382	385	408	391	383	387	387	383	418	380	384	378	385	380	379	718	374	388	382
88342	88342	88342	88342	93615	93616	93808	89163	88342	88342	88342	88342	88342	88342	88342	88340	88342	89406	88342	88342	88342
4622770	4694704	4695604	4687655	4710446	4629753	4620726	4736154	4696201	4687618	4830542	4690230	4687737	4688620	4625385	4688482	4690989	8396871	4683208	4698045	4689806
51.08	50.76	50.78	50.77	50.72	50.89	50.88	50.76	50.77	50.78	50.52	50.78	50.78	50.78	50.88	50.78	50.78	51.25	50.79	50.77	50.78
24175	26034	25905	26032	25688	25905	26019	26239	26034	26034	25688	25905	26034	26275	25905	26275	26034	22395	25905	26034	25905
11006	12708	13254	12743	12035	12293	12692	13519	12743	13254	12035	13254	12743	13254	12743	13254	13254	11257	12708	12708	13254
57	55	55	55	55	54	53	54	55	54	56	55	54	54	55	54	54	108	55	55	55
129	118	117	117	121	117	114	115	117	116	125	118	116	115	117	115	116	241	117	117	117
0	0	0	0	0	0	0	0	0	0	0	0	0	0	0	0	0	0	0	0	0

sh16sh287	sh16sh288	sh16sh289	sh16sh290	sh16sh291	sh16sh292	sh16sh293	sh16sh294	sh16sh295	sh16sh296	sh16sh297	sh16sh298	sh16sh299	sh16sh300	sh16sh301	sh16sh302	sh16sh303	sh16sh306	sh16sh308	sh16sh309	sh16sh336
570	575	561	582	579	579	576	628	575	548	581	560	580	593	561	579	612	569	581	678	630
324	326	330	325	325	349	330	330	326	324	327	328	326	326	326	337	422	330	326	360	331
207	207	205	208	207	216	204	209	209	209	209	205	207	209	211	207	241	203	205	215	203
145	145	143	145	145	148	141	148	145	147	144	142	143	146	148	142	142	141	140	143	141
60	60	60	60	60	62	58	61	60	59	60	59	61	60	56	60	37	60	59	59	57
16	16	15	16	16	16	14	17	16	15	15	14	16	15	15	16	3	15	14	14	15
4739835	4739295	4738795	4742406	4741959	4874013	4682055	4848931	4740620	4702940	4741975	4671373	4727107	4744171	4696129	4737916	4261262	4791099	4678430	4844086	4678492
4649095	4646765	4653001	4647256	4648651	4789734	4589899	4738558	4648090	4620171	4645592	4583870	4635585	4649567	4610684	4645865	4186916	4699522	4584924	4731485	4577901
4323727	4315484	4300216	4325959	4319527	4417244	4236809	4405846	4321163	4291448	4319067	4247729	4310480	4321596	4277714	4287402	3674940	4347998	4244295	4331276	4218897
3858495	3847751	3829530	3851432	3857739	3912526	3759449	3944075	3846859	3819865	3829384	3765118	3821063	3851520	3794041	3797478	2982970	3876758	3752646	3797330	3751574
2512793	2510100	2493660	2511074	2511171	2554800	2430190	2588205	2500688	2437365	2490559	2460898	2522782	2484365	2356962	2500513	1351312	2582748	2457953	2467959	2419115
1012672	1012692	950410	1013559	1012703	1001993	918765	1091514	1002187	940692	948769	922471	1005456	950222	953675	1001495	194494	1029823	920611	920661	984673
379	386	381	386	384	403	387	399	384	371	390	382	379	382	375	398	468	390	384	425	392
88342	88342	88342	88342	88342	88342	93616	88342	88340	88340	88342	93615	88342	88342	88342	88343	75758	107807	93615	93615	93808
4688398	4689631	4690277	4691011	4690983	4828203	4630513	4788309	4689335	4654973	4690224	4623234	4674057	4689770	4647003	4690278	4222376	4742700	4626173	4777101	4620980
50.78	50.78	50.77	50.78	50.78	50.52	50.87	50.76	50.77	50.87	50.77	50.9	50.81	50.77	50.91	50.78	51.9	50.77	50.89	50.61	50.86
26034	26034	25905	26034	26034	25905	25905	26700	26034	25752	25905	25752	26402	25905	25362	26035	16872	27071	26034	25752	25889
13254	12743	13254	13254	13519	12293	12708	12743	13254	12494	13254	12615	12743	13519	12134	12707	8534	12743	12708	12035	12475
54	54	55	54	54	57	54	54	54	55	55	54	54	55	55	54	74	52	54	56	53
116	116	117	116	116	123	117	118	117	118	117	116	116	117	121	118	162	114	116	124	116
0	0	0	0	0	0	0	0	0	0	0	0	0	0	0	0	0	0	0	0	0

sh16sh506	sh16sh529	sh16sh534	sh16sh540	sh16sh543	sh16sh546	sh16sh549	sh16sh550	sh16sh552	sh16sh564	sh16sh568	sh16sh573	sh16sh574	sh16sh575	sh16sh576	sh16sh577	sh16sh578	sh16sh580	sh16sh581	sh16sh587	sh16sh588
578	549	597	580	613	593	697	515	590	596	657	633	685	584	623	492	672	677	686	593	680
327	322	328	329	340	321	516	312	329	381	331	323	327	329	336	469	329	327	328	331	335
203	202	207	206	212	205	289	200	205	222	208	204	202	206	205	278	208	205	204	205	208
139	141	141	140	143	141	161	139	142	146	144	142	142	142	143	147	145	143	141	142	143
60	59	61	57	60	59	29	58	57	53	57	58	58	58	59	31	57	59	58	58	59
15	14	14	14	13	15	1	15	13	10	14	15	15	14	14	3	14	15	15	14	14
4674859	4650810	4713897	4654641	4727374	4651584	4646404	4572588	4662047	4626585	4675754	4667248	4679754	4677172	4668433	4520483	4677947	4670177	4681398	4685394	4682350
4583254	4565300	4619227	4563659	4625089	4557876	4570681	4494407	4570268	4542142	4575353	4568951	4573240	4585703	4569224	4511555	4573742	4564281	4572622	4589674	4573279
4234962	4220044	4286948	4216305	4271037	4229955	3955875	4184377	4223938	4116680	4239150	4242479	4221926	4247867	4199792	3970933	4236752	4222479	4224581	4241394	4218954
3752259	3752111	3783825	3712654	3757937	3750539	3010691	3728197	3754781	3563778	3752359	3783085	3774505	3767761	3731696	2993475	3764615	3758548	3756091	3763977	3742894
2481350	2434894	2500328	2401378	2440414	2448614	943600	2451189	2382729	2105682	2360804	2449868	2449166	2432150	2404888	1172756	2371373	2428774	2439975	2435195	2416623
953087	883389	881873	921444	822345	943440	63385	980773	862513	670036	909924	983676	983252	921092	864924	207202	904508	924440	984707	921576	880217
386	377	383	383	404	375	567	364	387	439	390	382	382	383	400	476	385	386	388	389	398
88343	88342	88342	93616	88343	88342	63385	93615	93599	93615	89560	93584	93584	93615	88327	93600	93584	88310	93583	93616	88310
4625190	4605304	4658644	4602900	4670544	4596651	4608475	4532066	4612170	4584948	4615996	4611129	4612715	4624001	4613770	4517092	4614313	4606135	4615780	4631428	4619048
50.89	50.91	50.8	50.88	50.87	50.89	50.85	50.91	50.9	50.87	50.9	50.88	50.87	50.89	50.87	51.02	50.87	50.86	50.87	50.88	50.86
26153	26034	26164	25752	25787	26034	13790	27080	25673	22251	25373	25762	25762	25752	25736	15324	25357	25755	25873	25752	25720
12743	13254	12743	12294	12491	12743	7891	12743	12691	11289	12681	13487	12711	12491	12278	8471	12459	12676	12676	12617	12261
54	54	55	54	56	54	101	51	54	61	55	53	53	54	56	87	55	55	53	54	55
115	116	116	117	121	115	211	111	117	135	119	114	115	116	120	191	119	117	116	117	119
0	0	0	0	0	0	0	0	0	0	0	0	0	0	0	0	0	0	0	0	0

sh16sh589	sh188	sh19sh001	sh19sh002	sh19sh003	sh19sh004	sh19sh005	sh19sh006	sh19sh007	sh19sh008	sh19sh009	sh19sh010	sh19sh011	sh19sh012	sh19sh013	sh19sh014	sh19sh015	sh19sh016	sh19sh017	sh19sh018	sh19sh019
652	795	621	754	743	739	733	752	764	742	748	755	754	752	711	750	735	740	771	750	759
328	329	326	359	358	359	352	358	360	357	362	360	359	356	354	356	358	361	367	357	365
205	200	204	213	213	213	213	214	213	213	214	212	214	211	215	213	214	214	219	217	215
147	143	142	145	142	144	144	143	142	143	144	145	142	141	145	143	145	143	146	144	146
59	54	57	59	59	59	59	59	59	59	59	59	59	59	59	59	59	59	59	59	59
16	14	13	15	15	15	15	15	15	15	14	14	15	15	14	15	15	15	14	15	14
4769114	4565058	4568281	4845815	4840352	4844922	4844764	4841414	4843820	4840796	4839821	4841027	4842084	4787198	4833184	4840429	4847338	4850957	4896795	4846062	4858697
4668384	4467846	4471911	4720097	4715189	4722414	4721512	4712138	4714808	4714601	4715143	4710654	4714315	4660696	4719224	4716193	4725918	4726232	4766869	4717883	4728387
4329578	4115193	4138637	4315393	4312255	4317758	4334129	4317363	4305509	4311495	4309129	4298213	4310287	4262699	4328577	4318512	4323275	4324709	4354491	4328864	4319138
3896023	3675025	3676433	3806273	3784128	3805566	3818384	3794806	3778562	3795899	3794070	3800723	3778252	3748054	3803444	3799797	3808282	3796833	3812763	3790151	3804260
2498904	2265304	2335261	2462621	2462503	2462034	2463857	2462776	2463837	2462510	2441377	2443468	2463297	2463108	2444166	2462753	2464248	2462843	2431272	2462135	2442988
1040657	902524	826219	962390	962184	962500	963228	962459	962805	962196	898896	898851	962337	963080	899383	962183	963227	962758	887424	962196	899183
387	381	382	422	421	422	418	424	425	423	427	430	422	424	417	417	422	429	435	424	434
88310	79708	88307	88307	88307	88307	88307	88307	88307	88307	88307	88307	88307	88307	88306	88306	88306	88306	88307	88307	88307
4709788	4504443	4511515	4765215	4761020	4767517	4768770	4759747	4761006	4762137	4762246	4760399	4759837	4708757	4764720	4760523	4770778	4774546	4814781	4765847	4777210
50.76	50.82	50.8	50.57	50.59	50.58	50.57	50.59	50.59	50.59	50.59	50.59	50.59	50.59	50.59	50.59	50.58	50.58	50.59	50.57	50.57
25873	25230	25720	25720	25720	25720	25720	25720	25720	25720	25357	25357	25720	25755	25357	25720	25720	25357	25061	25720	25357
13983	12533	12676	12032	12101	12101	12102	12102	12032	12101	12101	12032	12032	12003	12032	12101	12003	12032	12032	11963	12004
54	54	54	56	56	56	56	56	56	56	57	57	56	55	57	56	56	57	59	56	57
116	117	117	124	123	124	123	123	124	124	125	125	124	122	125	123	125	124	128	125	126
0	0	0	0	0	0	0	0	0	0	0	0	0	0	0	0	0	0	0	0	0

sh19sh020	sh19sh021	sh19sh022	sh19sh023	sh19sh024	sh19sh025	sh19sh026	sh19sh027	sh19sh028	sh19sh029	sh19sh030	sh19sh031	sh19sh032	sh19sh033	sh19sh034	sh19sh035	sh19sh036	sh19sh037	sh19sh038	sh19sh039	sh19sh040
753	759	738	732	766	737	726	755	620	608	623	606	605	617	616	623	622	634	612	613	615
360	359	357	355	363	356	352	362	326	330	330	328	323	324	326	328	328	329	324	323	330
217	215	213	215	214	214	209	214	207	206	206	209	205	206	206	206	207	205	206	205	205
144	143	144	143	143	144	142	145	142	143	141	142	143	143	143	144	143	142	142	143	141
59	59	59	59	59	59	59	59	58	58	58	58	58	58	58	58	58	58	58	58	58
15	15	15	14	14	15	15	14	15	15	15	14	15	15	14	14	15	15	15	15	15
4846102	4842263	4839671	4838144	4861688	4844479	4788385	4846776	4663901	4671521	4663732	4666424	4667215	4660303	4663596	4673910	4673243	4665151	4663476	4668847	4668029
4720069	4717621	4715857	4718267	4730167	4720134	4666616	4723273	4566945	4576017	4565552	4571673	4573117	4562992	4567913	4577723	4575374	4565558	4569048	4573656	4570889
4330433	4322828	4318550	4324191	4315177	4327454	4271588	4317171	4235875	4230419	4225210	4239247	4245551	4232946	4226710	4234029	4238819	4222089	4244051	4245579	4224662
3788843	3789585	3805378	3789486	3785990	3811002	3778339	3798763	3749607	3761325	3735666	3737093	3775057	3760522	3755866	3764812	3755915	3750393	3763413	3775827	3741664
2463228	2461868	2461836	2446546	2443266	2462347	2461664	2443161	2410617	2417977	2419076	2398982	2418972	2410753	2399100	2399497	2410530	2409696	2418408	2419183	2425692
962616	962481	962178	898867	899253	962184	962304	899273	952162	959982	959983	896693	960363	952010	896938	896867	951725	952079	960257	960239	960071
424	420	421	416	433	419	412	423	384	387	388	386	378	380	383	383	387	389	377	377	387
88307	88307	88307	88307	88306	88307	88307	88306	88311	88311	88311	88311	88311	88311	88310	88310	88311	88311	88311	88310	88311
4765403	4761756	4762543	4761820	4779774	4765858	4710499	4766449	4607934	4616886	4606473	4612594	4612320	4603061	4608464	4616564	4616884	4608170	4606542	4612402	4611665
50.57	50.59	50.59	50.59	50.58	50.57	50.61	50.57	50.87	50.85	50.87	50.86	50.86	50.85	50.87	50.85	50.84	50.87	50.87	50.86	50.86
25729	25720	25720	25357	25357	25720	25755	25357	25755	25755	25755	25720	25873	25873	25720	25720	25873	25873	25755	25873	25755
11963	12003	12032	12101	12032	12102	12257	12003	12711	12711	12676	12676	13222	12711	13222	12711	12676	12711	13222	13222	12676
56	56	56	57	57	56	55	57	54	54	54	55	54	54	55	55	54	54	54	54	54
125	124	124	124	125	123	121	125	117	117	117	118	116	117	117	118	118	117	116	116	117
0	0	0	0	0	0	0	0	0	0	0	0	0	0	0	0	0	0	0	0	0

sh19sh041	sh19sh042	sh19sh043	sh19sh044	sh19sh045	sh19sh046	sh19sh047	sh19sh048	sh19sh049	sh236	sn0603	sn0701	sn0707	sn0713	sn0714	sn0716	sn0717	sn0719	sn0720	sn0722	sn0723
628	613	614	623	622	630	634	617	605	779	710	753	597	591	596	582	692	708	577	603	583
329	331	329	330	334	324	329	328	329	325	354	316	314	315	315	314	346	345	318	315	327
207	207	208	207	207	206	205	208	205	202	215	198	197	199	198	197	206	205	202	199	202
142	142	143	143	143	144	143	144	142	147	144	145	141	142	142	139	143	143	143	142	139
58	58	58	58	58	58	58	58	58	56	60	54	57	57	57	58	58	58	59	57	56
15	15	14	14	14	14	15	14	15	13	15	13	15	14	14	15	14	14	15	14	15
4670275	4667973	4663548	4664191	4674836	4670972	4674686	4672453	4656927	4610859	4834139	4562166	4613880	4613094	4613843	4617531	4765349	4767417	4694880	4614941	4627479
4571686	4571213	4565077	4566173	4577585	4571438	4576168	4574506	4561099	4515598	4714584	4469782	4520665	4520569	4520583	4525641	4653804	4651512	4603808	4520218	4532424
4232762	4231414	4226215	4226877	4226683	4240584	4227836	4237232	4217489	4176634	4334793	4141928	4197749	4202018	4199399	4198967	4277294	4269523	4282929	4198469	4192533
3746979	3745416	3737526	3752006	3745481	3774727	3762293	3763029	3744812	3751899	3813783	3729931	3773565	3772373	3774498	3760086	3807923	3806373	3833800	3768423	3719093
2418223	2418492	2391593	2397481	2398714	2399484	2418436	2399776	2416726	2310374	2479319	2289435	2431369	2410820	2412470	2459349	2446622	2445348	2500354	2412111	2402569
960010	961028	889466	896680	896737	897140	959939	897697	959926	840288	945963	878065	1002401	937567	939170	1003412	938200	938499	1000003	940147	1003170
386	389	389	388	392	380	384	387	386	372	425	362	369	369	371	370	404	402	369	368	386
88311	88311	88311	88311	88311	88311	88311	88311	88311	79708	88326	112640	112747	111232	112747	112755	112296	112506	109829	112747	112314
4612368	4612876	4608249	4607697	4619260	4611119	4615430	4617076	4602019	4548192	4764278	4502140	4559422	4559172	4559548	4565305	4694340	4691984	4640062	4557792	4573917
50.86	50.86	50.86	50.87	50.85	50.86	50.85	50.85	50.88	50.88	50.6	50.8	50.8	50.8	50.8	50.81	50.55	50.56	50.83	50.8	50.81
25755	25755	25720	25720	25720	25755	25755	25720	25755	25257	25673	25257	25873	25873	25873	26143	25664	25720	26275	25873	25752
12676	12676	12459	12711	12459	13222	12711	12711	12676	12576	12058	13122	14019	14019	14060	14035	12713	12713	14036	14004	12734
54	54	55	55	55	55	54	55	54	55	57	53	52	52	52	52	55	55	52	52	52
117	117	119	118	119	117	117	118	117	118	123	115	112	113	113	111	119	119	113	113	114
0	0	0	0	0	0	0	0	0	0	0	0	0	0	0	0	0	0	0	0	0

sn0724	sn0729	sn0730	sn0741	sn0742	sn0743	sn0801	sn0806	sn0815	sn0932	sn0947	sn1004	sn1016	sn1017	sn1103	sn1104	sn1201	SRR3530393	SRR3530435	SRR3530549	SRR3530555
586	694	708	600	698	697	586	763	913	779	796	770	601	798	796	782	810	829	892	964	890
318	341	344	316	345	345	359	318	348	323	320	322	323	326	332	329	329	396	383	365	406
200	209	209	198	204	204	217	197	211	200	198	201	203	203	199	200	204	216	221	216	213
141	143	143	142	142	142	138	141	143	142	140	141	140	144	145	144	146	145	149	145	146
57	58	59	57	58	58	53	53	54	53	53	53	57	54	55	53	54	52	52	54	53
15	14	15	14	15	15	10	13	12	12	13	13	15	13	13	13	14	11	10	13	11
4612442	4768537	4786048	4614106	4766050	4765832	4551424	4440126	4614986	4457799	4440321	4456973	4605504	4543731	4560335	4557958	4614109	4760166	4788864	4820964	4774677
4522709	4655200	4667589	4520439	4651666	4652925	4467126	4350425	4501296	4362512	4344583	4363989	4513509	4446991	4464934	4460770	4515729	4646129	4667932	4681265	4651140
4198961	4290995	4300251	4195168	4270205	4271130	4070103	4005971	4116546	4012675	3998212	4024858	4180636	4100821	4095725	4099289	4169003	4173245	4236252	4263188	4135739
3757035	3801718	3807101	3770839	3807761	3807021	3486845	3573315	3611473	3564439	3550192	3568301	3711616	3649825	3674314	3667644	3718924	3637570	3690374	3724148	3630643
2431787	2446027	2465089	2411623	2465676	2465927	2141597	2183566	2211277	2163751	2183762	2183456	2400510	2237790	2255512	2230530	2254716	2135556	2096885	2230948	2116957
1002345	938273	951625	938957	1002224	1002041	643066	829533	778177	765557	828819	828902	977537	839368	821746	849946	894673	692563	641864	829344	682713
371	401	407	370	404	404	417	363	398	373	367	370	378	375	379	382	377	465	444	429	473
112747	112296	88310	112747	112506	112747	79407	79705	88210	79708	79708	79708	88310	79708	79708	84961	84201	88237	88237	88226	88238
4560206	4697713	4711681	4558326	4692896	4694022	4506772	4381325	4536652	4396739	4377367	4397526	4552340	4480699	4498086	4497787	4549143	4695607	4709568	4726310	4698424
50.8	50.55	50.52	50.8	50.56	50.56	51.22	50.8	50.51	50.8	50.8	50.8	50.82	50.78	50.84	50.85	50.73	50.59	50.57	50.57	50.66
25873	25720	25726	25762	25761	25761	23497	24961	23902	24359	24961	24961	25755	24961	25230	24961	24961	22142	22142	22847	22142
13487	12678	12678	14019	13096	12711	11060	12157	11796	11932	12001	12002	12711	12001	12162	12162	12533	11029	11495	11952	11346
52	55	55	52	54	54	58	54	57	55	54	54	53	55	55	54	55	62	63	60	63
113	119	120	113	117	118	128	116	124	119	117	117	114	119	119	118	119	135	135	130	137
0	0	0	0	0	0	0	0	0	0	0	0	0	0	0	0	0	0	0	0	0

SRR3530636	SRR3530638	SRR3530640	SRR3530773	SRR3530795	SRR3530807	SRR3531022	SRR3531029	SRR3531087	SRR3531231	SRR3531253	SRR3531721	SRR3531782	SRR3531866	SRR3531874	SRR3531882	SRR3531887	SRR3531897	SRR3531903	SRR3531909	SRR4192106
1028	845	950	967	784	943	924	927	1037	915	945	826	748	961	959	927	763	1034	927	989	1211
405	379	370	365	340	358	360	362	374	375	374	353	340	375	377	393	340	398	361	369	398
226	211	217	217	208	217	217	215	216	215	219	208	208	219	211	217	207	221	218	218	226
154	145	142	144	141	144	145	144	143	139	138	145	141	150	143	144	141	148	144	146	149
52	55	55	53	52	55	53	57	53	53	53	54	52	56	56	51	52	52	53	55	53
7	10	11	12	12	11	12	12	12	12	12	12	12	12	12	12	11	11	11	12	11
4814863	4728462	4756450	4731639	4564541	4759128	4730976	4831759	4812905	4720650	4729634	4657162	4568022	4930040	4797720	4806450	4561828	4816675	4728765	4825544	5010246
4666315	4609655	4612818	4591917	4457872	4620996	4596895	4694857	4672576	4583204	4591928	4545363	4467827	4791550	4657344	4671046	4460250	4661840	4590254	4685008	4811651
4173019	4157974	4189994	4179516	4086733	4229824	4204195	4282714	4234565	4139412	4162538	4146527	4098561	4353284	4199296	4197408	4090928	4172191	4190107	4265749	4337215
3632150	3651669	3633941	3631254	3576214	3681560	3656673	3753376	3675443	3563069	3556080	3660114	3581874	3833757	3678362	3642616	3581375	3620782	3637915	3721632	3754226
2026367	2217666	2254682	2189506	2155167	2261653	2189143	2372443	2200797	2164088	2196678	2204798	2146237	2312198	2272348	2107704	2135565	2090324	2169091	2232514	2198679
459836	660268	710224	776929	776601	725642	774892	791107	778381	760136	776569	760195	776022	801825	762156	763493	710500	680702	710560	746023	756038
475	445	442	427	392	429	424	430	434	441	436	406	390	433	443	465	390	469	423	425	493
88231	87486	87485	88239	88240	87485	88240	87485	88229	88240	88240	88238	88239	107542	88238	88238	88239	80288	88239	88237	94115
4715338	4655483	4663702	4635638	4494766	4670038	4642339	4742176	4714921	4631251	4634946	4582702	4502943	4833071	4703732	4722279	4495620	4711725	4634788	4724864	4877158
50.57	50.49	50.47	50.53	50.81	50.46	50.53	50.5	50.56	50.55	50.53	50.84	50.83	50.45	50.56	50.57	50.82	50.57	50.53	50.56	50.45
20861	22847	23301	22381	23301	22847	22381	25250	22373	22278	22381	23301	22847	23386	23301	21790	23301	21151	22381	22847	22002
11045	11415	11414	11442	11687	11502	11502	11687	11566	11388	11192	11848	12021	11952	11502	11455	11952	11045	11455	11883	11135
67	60	59	59	56	59	59	57	60	60	59	58	57	61	60	62	57	64	60	61	64
146	131	130	130	123	128	130	127	131	131	131	125	123	132	130	135	123	140	130	131	141
0	0	0	0	0	0	0	0	0	0	0	0	0	0	0	0	0	0	0	0	0

SRR4195487	SRR4195740	SRR4786326	SRR4787219	SRR4787278	SRR4788222	SRR5005299	SRR5005327	SRR5006072	SRR5023700	SRR5029673	SY230422	SY230719	WH13	WH25	WH52	WH7	WH82	WH88	X8	XJ12140	XJ12212
1115	983	1011	785	1006	1017	1032	785	1063	1114	871	655	589	826	808	586	634	753	574	796	811	734
375	372	368	334	383	382	370	338	374	346	368	349	346	335	332	331	326	367	327	324	324	358
216	216	221	205	222	216	214	207	221	209	212	212	205	202	206	208	211	213	207	201	196	209
146	145	141	144	147	144	144	143	149	146	146	146	138	146	146	138	145	145	146	146	137	140
53	54	52	52	53	54	55	54	54	52	54	58	57	53	52	59	58	59	60	53	54	57
12	12	12	12	9	12	12	10	12	10	11	14	13	12	13	14	14	16	16	13	12	14
4833746	4822204	4716859	4558535	4751197	4805759	4816712	4593911	4871100	4616968	4721872	4788790	4601309	4560865	4545160	4645941	4721083	4845948	4806391	4568909	4481455	4703024
4675466	4679050	4575014	4458602	4610968	4657578	4673363	4484305	4727783	4464041	4601518	4680978	4511913	4461299	4446314	4552052	4621529	4722194	4716620	4473507	4379082	4585698
4232473	4246620	4168154	4100780	4173608	4193591	4239072	4120940	4304533	4074028	4182809	4304949	4123043	4099590	4098346	4221213	4305425	4313273	4387126	4129824	4009967	4178712
3702144	3707478	3578297	3632094	3620279	3648298	3709321	3635194	3770525	3597257	3682645	3815981	3613243	3665881	3638070	3698469	3804764	3815654	3922885	3705707	3569586	3668506
2187188	2203036	2156333	2160607	2119988	2195228	2267072	2198920	2219104	2079752	2215021	2421293	2321026	2187308	2149124	2447569	2430938	2457265	2564925	2230852	2227152	2349313
762960	761996	775726	775577	583465	757103	762072	640339	764792	602120	710503	915772	823640	773059	823788	896562	918370	1002025	1047555	850515	778302	890882
437	434	427	382	450	444	432	396	432	411	434	408	404	384	381	383	380	432	379	372	374	415
88237	88237	88232	88232	87478	88226	88230	87477	88229	87478	87477	88342	88343	79708	76340	88391	88567	88311	90337	84961	88209	88311
4718930	4723093	4617256	4493107	4657959	4702968	4717177	4524762	4768328	4511145	4648130	4722792	4551955	4495781	4480382	4589413	4660095	4768081	4754440	4507155	4414575	4626256
50.56	50.57	50.57	50.81	50.49	50.55	50.56	50.75	50.47	50.74	50.48	50.51	50.76	50.85	50.76	50.75	50.55	50.43	50.75	50.83	50.55	50.44
22381	23180	22373	23293	22270	23166	23293	23987	22373	22270	23256	25389	25362	24338	23764	26034	25752	25357	27080	24961	25230	25330
11687	11883	11340	12173	11184	11338	11875	12013	11679	11839	11435	12289	11912	12162	12001	12275	12567	12101	13518	12574	12157	12032
61	61	59	56	62	61	59	57	61	60	59	56	56	56	56	54	55	57	53	54	54	56
132	131	131	121	136	133	129	122	132	127	129	122	121	120	122	116	118	124	115	118	115	123
0	0	0	0	0	0	0	0	0	0	0	0	0	0	0	0	0	0	0	0	0	0

Table S10. The primers used in this study

Target	Primer sequence (5' to 3')	Amplicon size (bp)
mhp operon		
mhpB	F: CGGTAATAACGCCCTTCG<br>R: GCACGCTTATCTTCACTGTCTT	885
mhpA	F: CGTATTGCCCAGGGTTTG<br>R: GGCTGATGATGGCGAACTA	1514
mhpR	F: CAATGAGCAGACGGAATACA<br>R: TAGCCTGCGATTCAACCC	785
***lac* operon**		
lacI	F: CCAACCGCGTGGCACAACA<br>R: AGGCGGTTTGCGTATTGG	867
lacZ	F: TCTTCCTGAGGCCGATACTGT<br>R: TTCAGCCATGTGCCTTCTT	2724
lacY	F: TTTGGATGTTCGGTTTATTC<br>R: TAAGTTGGCAGCATCACC	263
**Construction of the Δ *lac* operon mutant**		
MR-U	F: CAATGAGCAGACGGAATACA<br>R:	850
LacY/yahN-D	F: TGGACCATGGCTAATTCCCATGGTG<br>R: GCAAGACCAAACAAACCCA	980
pKD4-kana	F: GTGTAGGCTGGAGCTGCTTC<br>R: ATGGGAATTAGCCATGGTCC	1512
inside	F: CCAGACACCCATCAACAG<br>R: GGCGCAAACATCACTAAC	4242
outside	F: GCCTCGTCATCAATACCAA<br>R: AAGAAAGACTCGCCAGAT	7051

Table S11. Abbreviations and their full names used in this study.

Number	Abbreviations	Full names
1	*S. sonnei*	*Shigella sonnei*
2	$cef^{R}azi^{R}$	Coresistance to ceftriaxone and azithromycin
3	ONPG	O-nitrophenyl-β-D-galactopyranoside
4	MDR	Multidrug resistance
5	AMR	Antimicrobial resistance
6	SNP	Single-nucleotide polymorphism
7	QRDR	Quinolone resistance-determining region
8	MSM	Men who have sex with men
9	WGS	Whole-genome sequencing
10	MIC	Minimal inhibitory concentration
11	CLSI	Clinical and Laboratory Standards Institute
12	BEAST	Bayesian Evolutionary Analysis Sampling Tree
13	MCMC	Markov chain Monte Carlo
14	MCC	Maximum clade credibility
15	RGI	Resistance Gene Identifier
16	CARD	Comprehensive Antibiotic Resistance Database
17	FDR	False discovery rate
18	GWAS	Genome-wide association study
19	COGs	Clusters of Orthologous Groups
20	PCR	Polymerase chain reaction
21	MRCA	Most recent common ancestor
22	IS	Insertion sequence
