# Supplementary figures and images for: Divergent Evolution and Local Establishment of Multidrug‐Resistant Shigella sonnei in China
Source: MedComm (2020). 2026 Jan 7;7(1):e70569. doi: 10.1002/mco2.70569 (PMC12778403; doi:10.1002/mco2.70569)

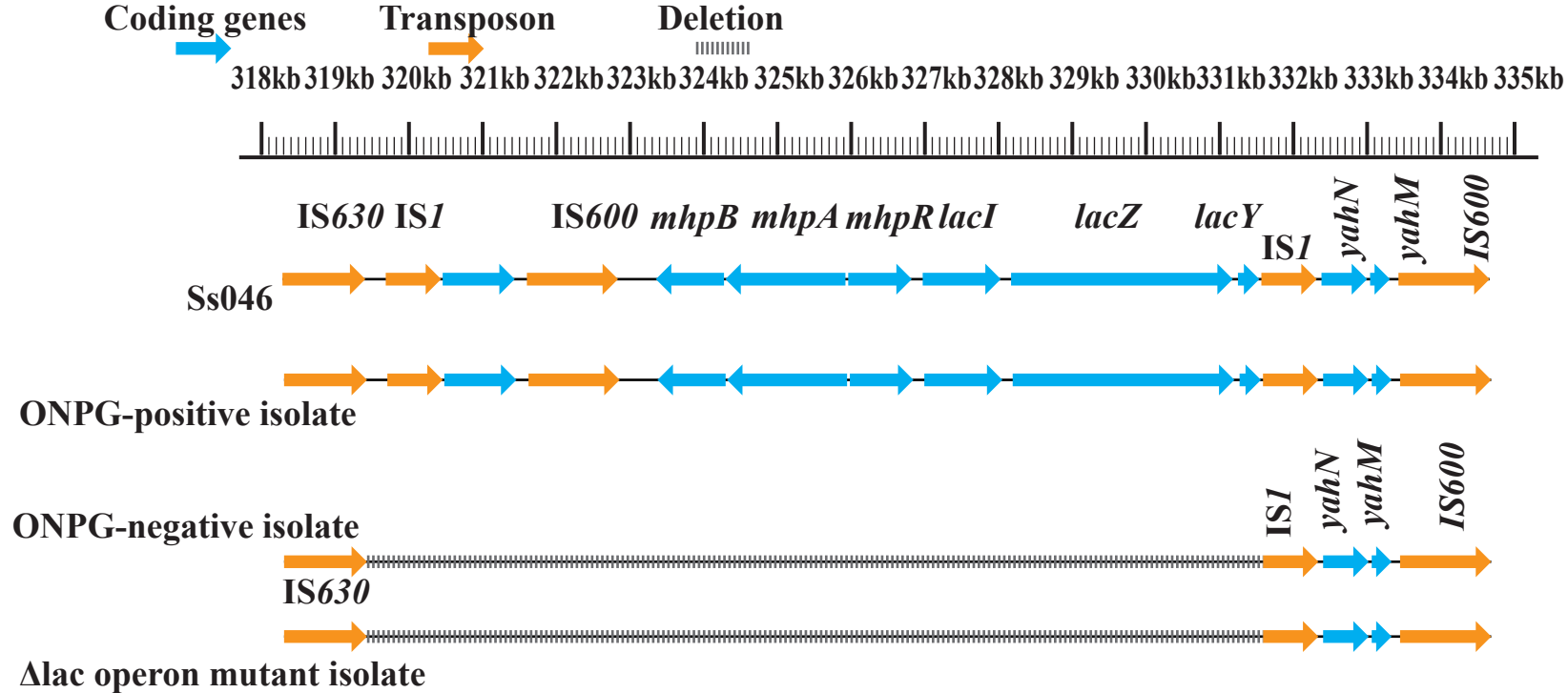

Supplement: Supplementary file 2 — Supporting File 2: mco270569‐sup‐0002‐FigureS4.pdf [file MCO2-7-e70569-s009.pdf]

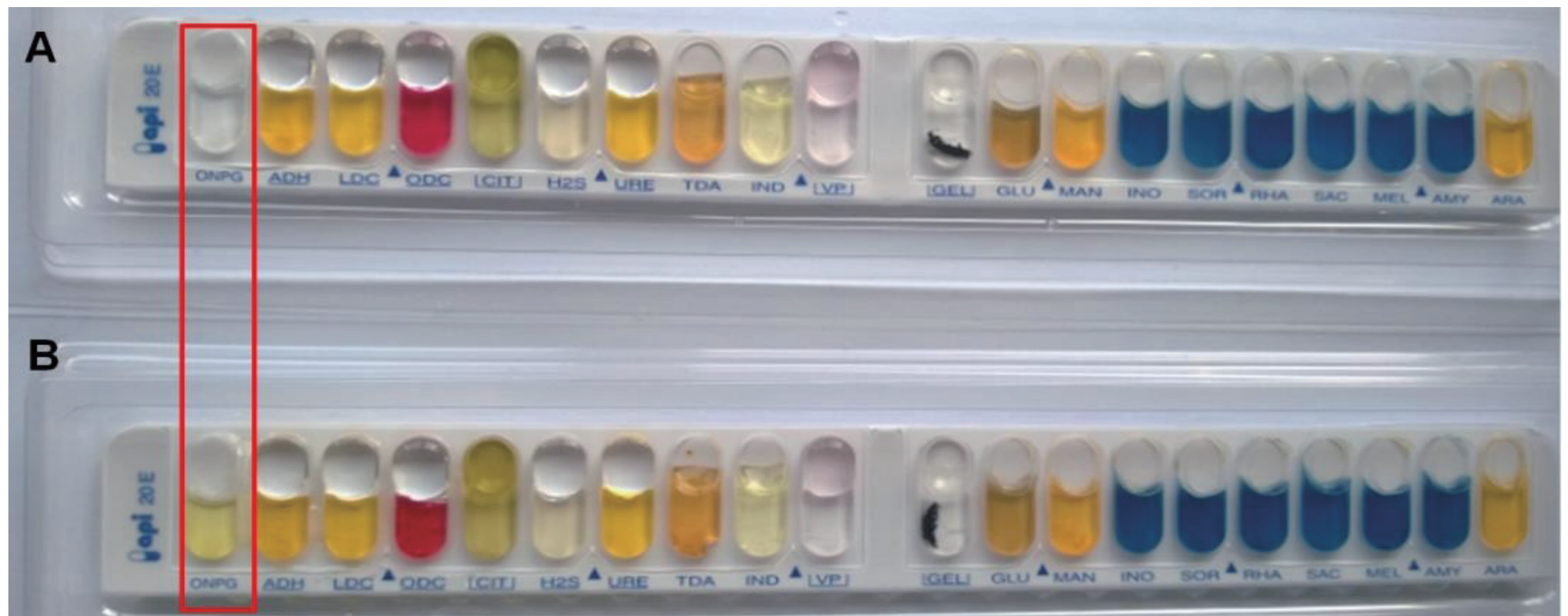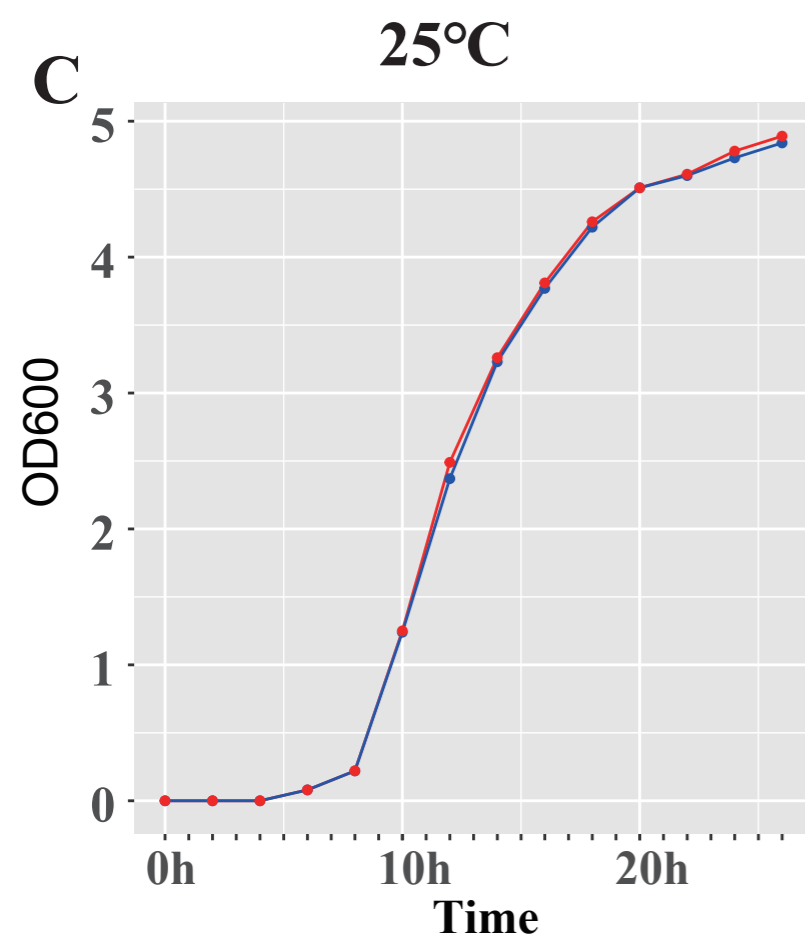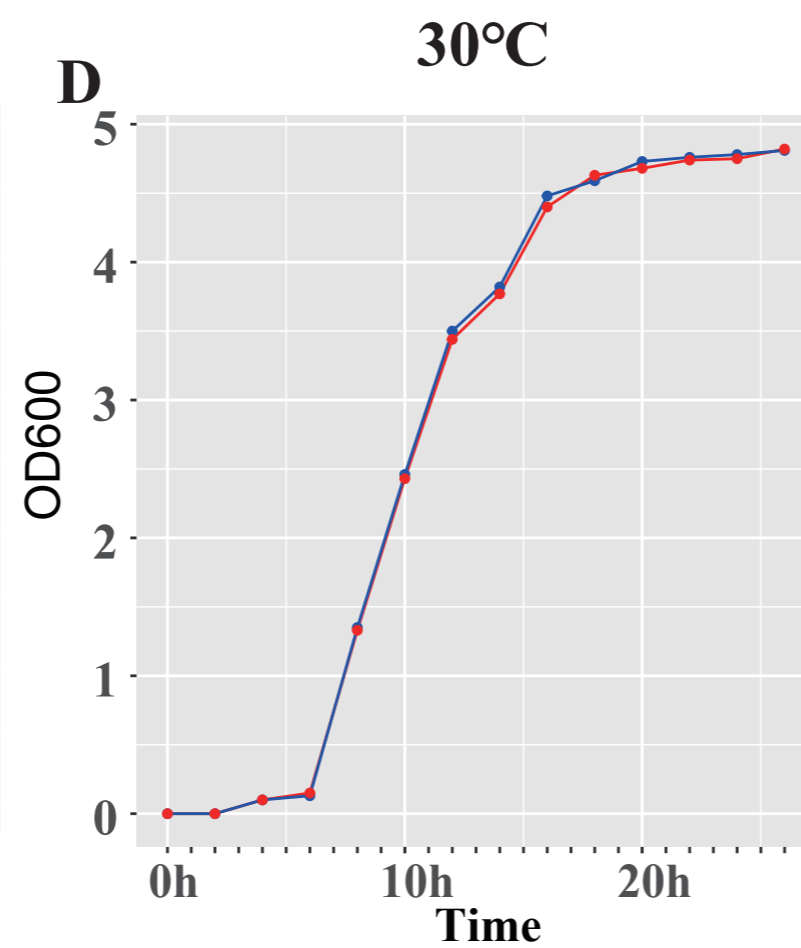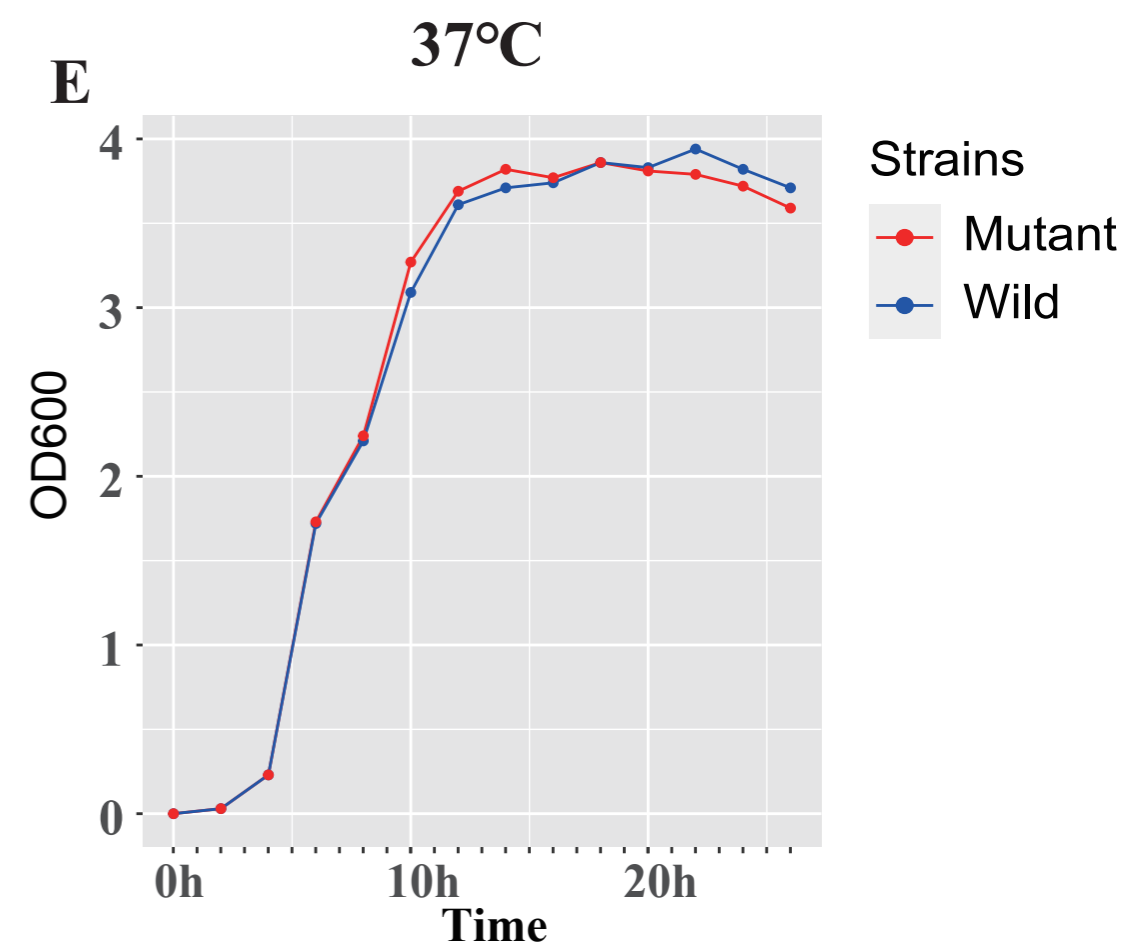

Supplement: Supplementary file 3 — Supporting File 3: mco270569‐sup‐0003‐FigureS5.pdf [file MCO2-7-e70569-s010.pdf]

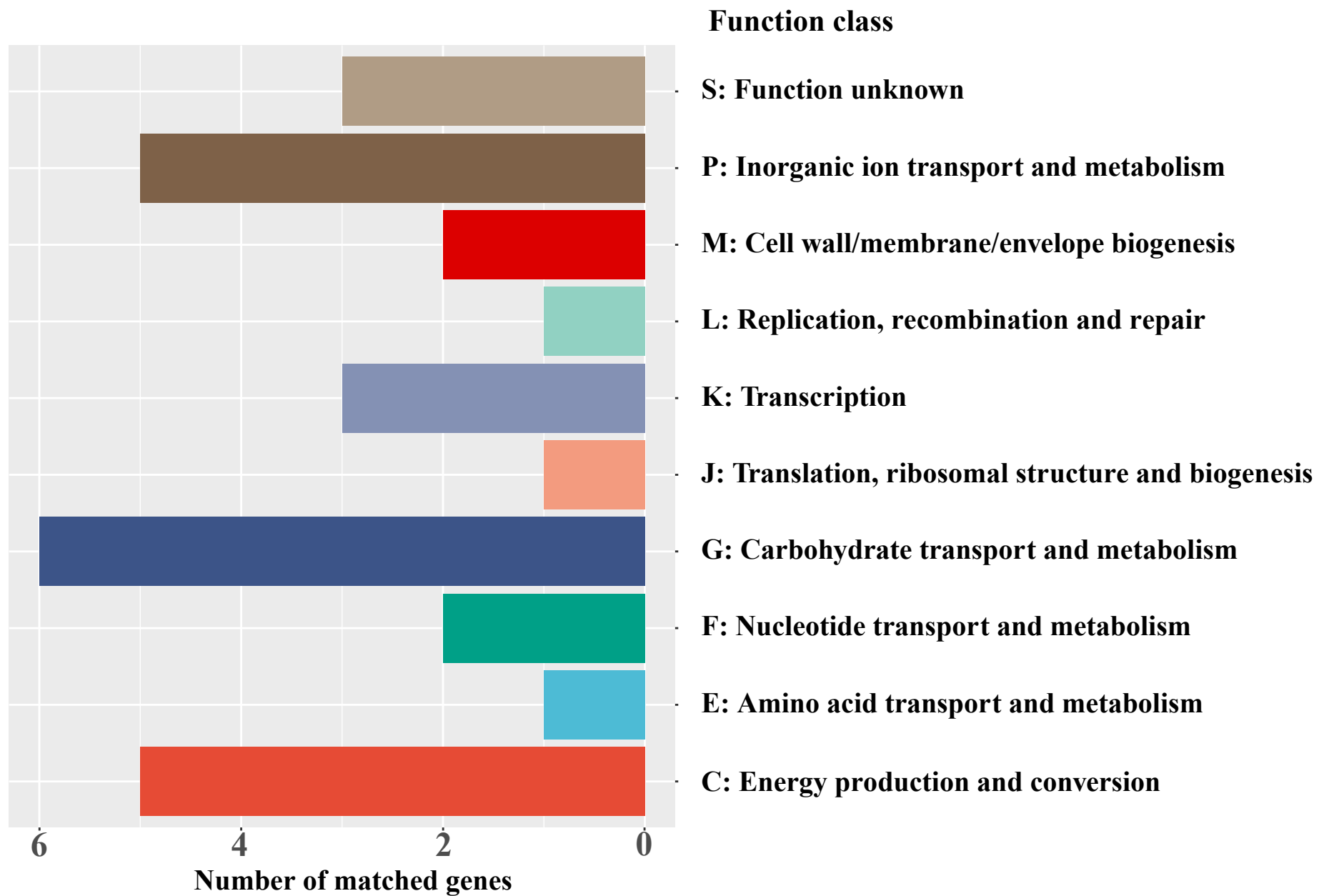

Supplement: Supplementary file 4 — Supporting File 4: mco270569‐sup‐0004‐FigureS6.pdf [file MCO2-7-e70569-s002.pdf]

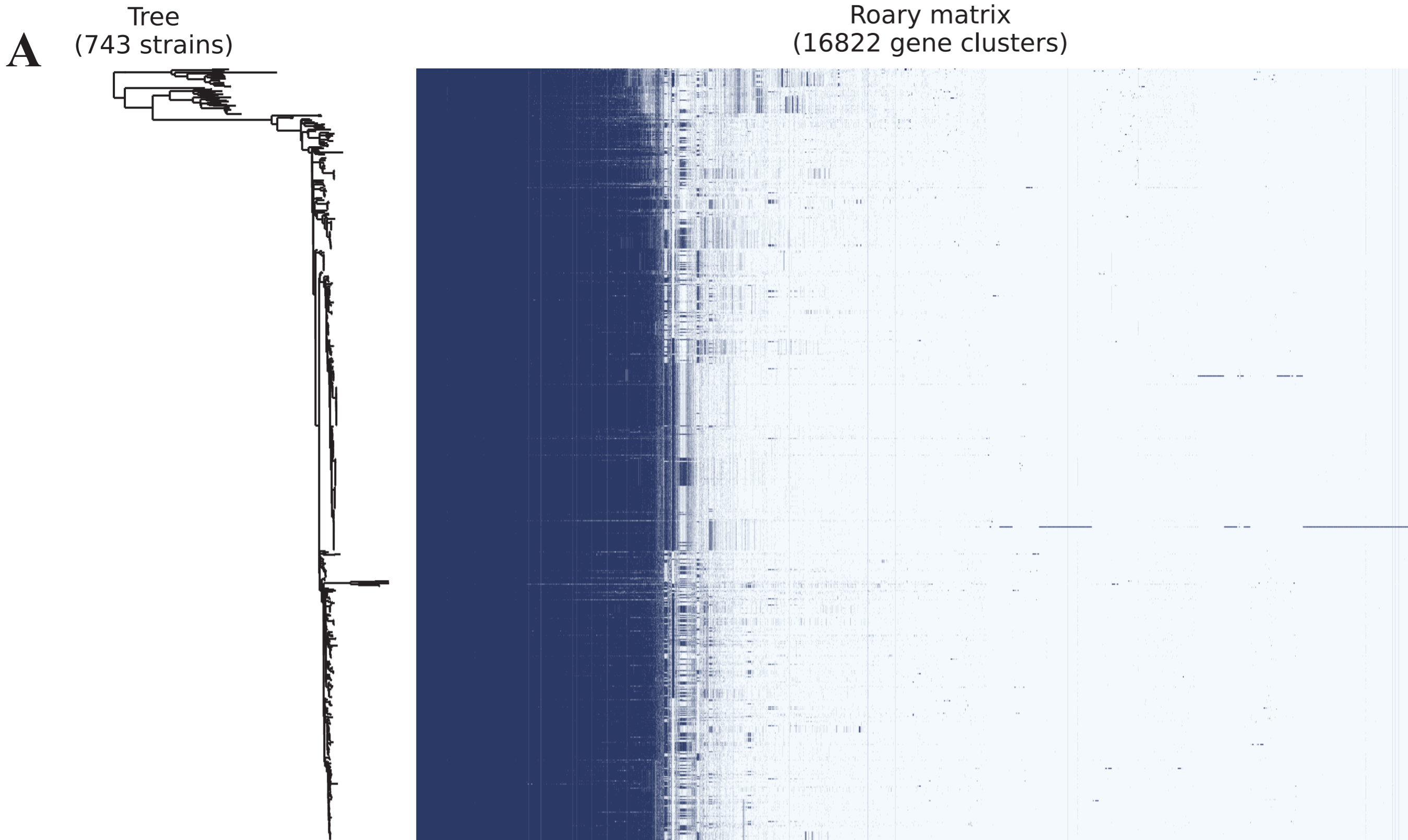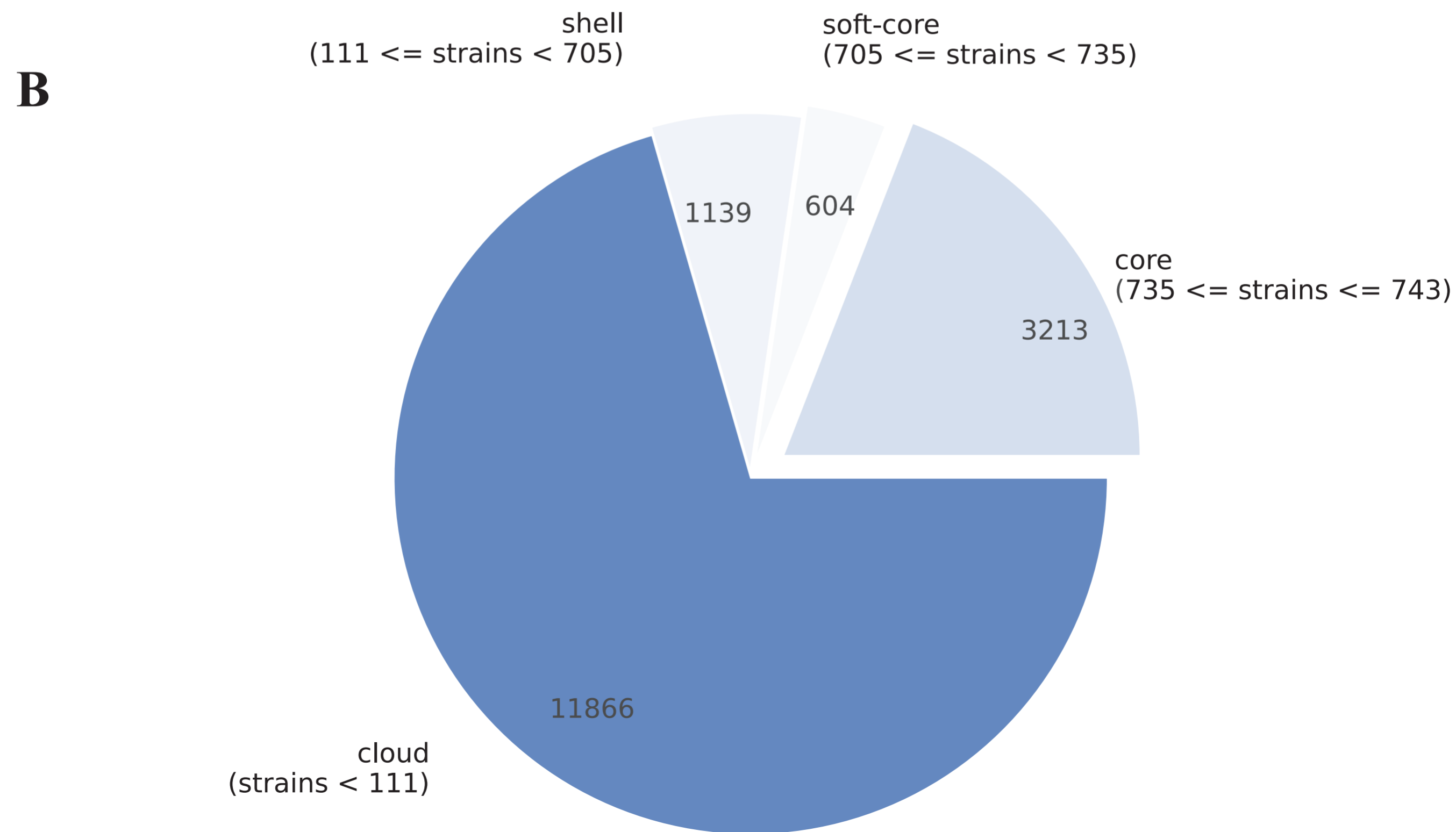

Supplement: Supplementary file 5 — Supporting File 5: mco270569‐sup‐0005‐FigureS7.pdf [file MCO2-7-e70569-s012.pdf]
